# Total Syntheses of (+)-Waixenicin A, (+)-9-Deacetoxy-14,15-deepoxyxeniculin, and (−)-Xeniafaraunol A

**DOI:** 10.1021/jacs.3c03366

**Published:** 2023-05-16

**Authors:** Christian Steinborn, Tatjana Huber, Julian Lichtenegger, Immanuel Plangger, Klaus Wurst, Thomas Magauer

**Affiliations:** † Institute of Organic Chemistry and Center for Molecular Biosciences, University of Innsbruck, Innrain 80−82, 6020 Innsbruck, Austria; ‡ Institute of General, Inorganic & Theoretical Chemistry, University of Innsbruck, Innrain 80−82, 6020 Innsbruck, Austria

## Abstract

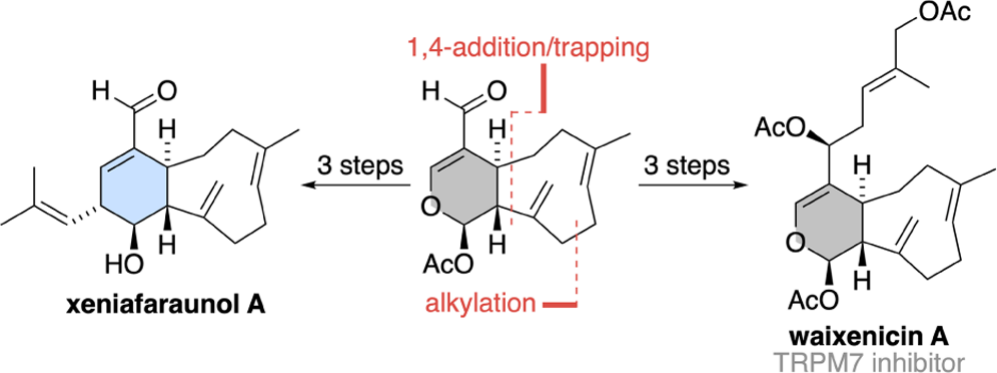

The first asymmetric total synthesis of the *Xenia* diterpenoid waixenicin A, a potent and highly selective TRPM7 inhibitor, is reported. The characteristic *trans*-fused oxabicyclo[7.4.0]tridecane ring system was constructed via a diastereoselective conjugate addition/trapping sequence, followed by an intramolecular alkylation to forge the 9-membered ring. While a β-keto sulfone motif enabled efficient ring-closure, the subsequent radical desulfonylation suffered from (*E*)/(*Z*)-isomerization of the C7/C8-alkene. Conducting the sequence with a trimethylsilylethyl ester allowed for a fluoride-mediated decarboxylation that proceeded without detectable isomerization. The acid-labile enol acetal of the delicate dihydropyran core was introduced at an early stage and temporarily deactivated by a triflate function. The latter was critical for the introduction of the side chain. Diverting from a common late-stage intermediate provided access to waixenicin A and 9-deacetoxy-14,15-deepoxyxeniculin. A high-yielding base-mediated dihydropyran-cyclohexene rearrangement of 9-deacetoxy-14,15-deepoxyxeniculin led to xeniafaraunol A in one step.

## Introduction

**Figure 1:**
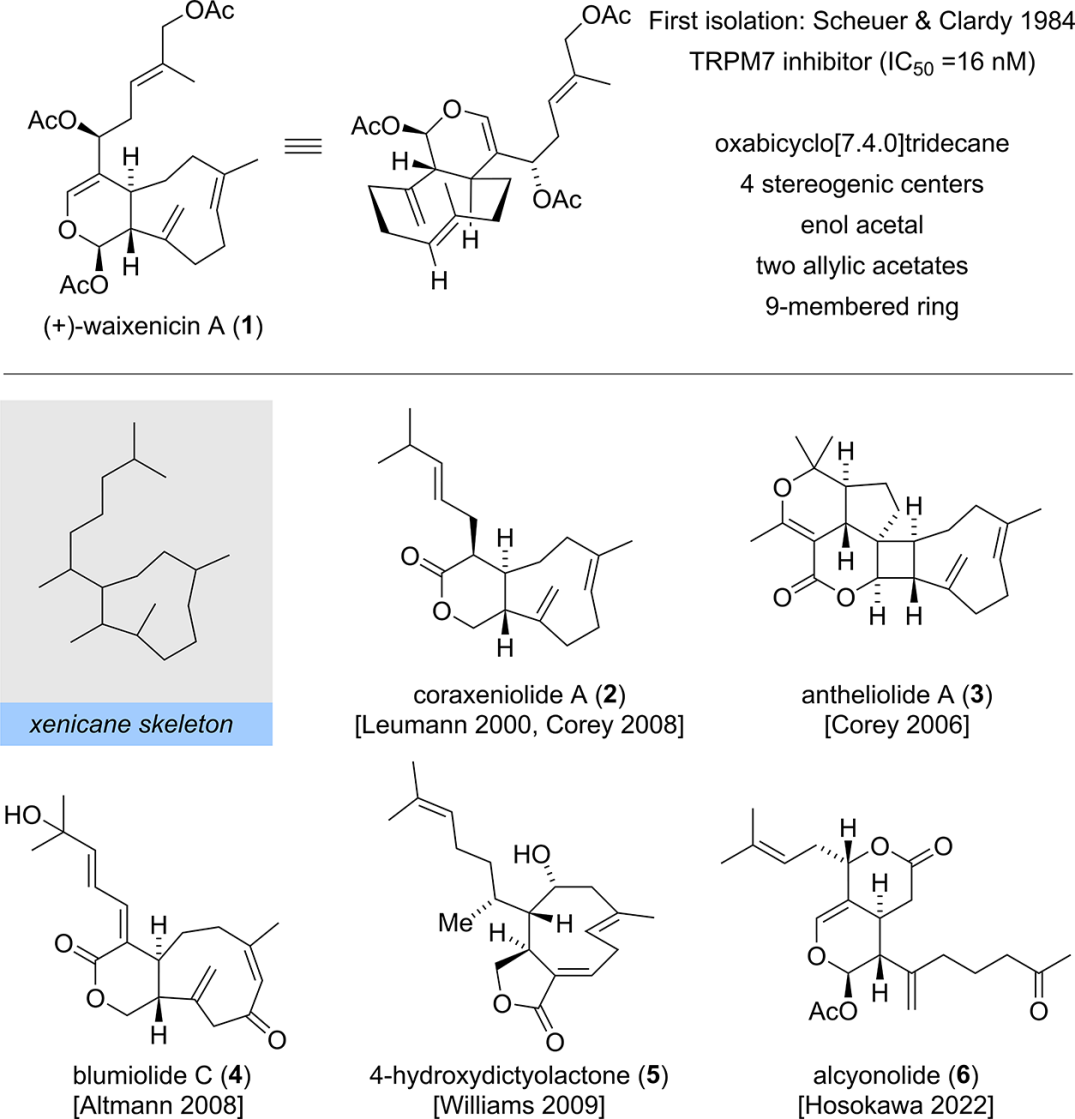
General features and selected structures of *Xenia* diterpenoids.

In 1984, Scheuer and Clardy reported the isolation of waixenicin A (**1**) from an extract of the marine soft coral *Sarcothelia edmondsoni* harvested along the Hawaiian coast (Figure 1).^1^**1** stands out due to its unique biological profile^2^ and has been intensively investigated for its potential to act as a specific inhibitor of transient receptor potential melastatin 7 (TRPM7) channels, blocking cell proliferation with an Mg^2+^-dependent IC_50_ value of 16 nM. Interestingly, **1** displayed no inhibitory activity for TRPM6, which represents the closest homologue of TRPM7.^3^ This makes **1** a highly attractive natural product for targeting TRPM7-related pathophysiologies such as cancer. Structurally, waixenicin A (**1**) belongs to the xenicin subclass of *Xenia* diterpenoids, a unique family of natural products that was first described in 1977.^4^ Since then, several challenging structures have been isolated and identified from marine organisms.^5^ Their oxidized polycyclic framework, along with their diverse biological activities, has stimulated the chemical community to initiate several synthesis campaigns.^6^ To date, only the total syntheses of coraxeniolide A (**2**),^7^ antheliolide A (**3**),^8^ blumiolide C (**4**),^9^ the related *Dictyo* diterpenoid 4-hydroxydictyolactone (**5**),^10^ and *seco*-xenicin alcyonolide (**6**)^11^ have been accomplished. The foundation for the first synthesis of a *Xenia* diterpenoid was laid in seminal work by Corey in 1963 (caryophyllene)^12^ and in 1995 by Pfander’s studies toward coraxeniolide A (**2**).^13^ Analysis of the molecular architecture of **1** reveals several structural challenges including a *trans*-fused oxabicyclo[7.4.0]tridecane ring system and four stereogenic centers, three of which are embedded within the delicate dihydropyran core. The latter, which contains an acid-labile enol acetal and is linked to a side chain carrying two base-labile allylic acetates, clearly distinguishes **1** from the lactone motifs found in **2**–**5**. The inherent strain of the 9-membered ring,^14^ an additional synthetic challenge that is missing for alcyonolide (**6**), is further augmented by the trisubstituted (*E*)-configured alkene. Notably, increased reactivity toward atmospheric oxygen was observed by Higuchi for a closely related system.^15^ For other xenicins, additional complexity and structural variation arise from the total degree of unsaturation and the oxidation pattern along the periphery.^16^ Their synthesis has remained elusive for more than four decades. Here, we report the total synthesis of (+)-waixenicin A (**1**) and (+)-9-deacetoxy-14,15-deepoxyxeniculin (**30**),^17^ as well as the one-step conversion to (−)-xeniafaraunol A (**31**).^18^

## Results and Discussion

### Retrosynthetic Analysis

**Scheme 1:**
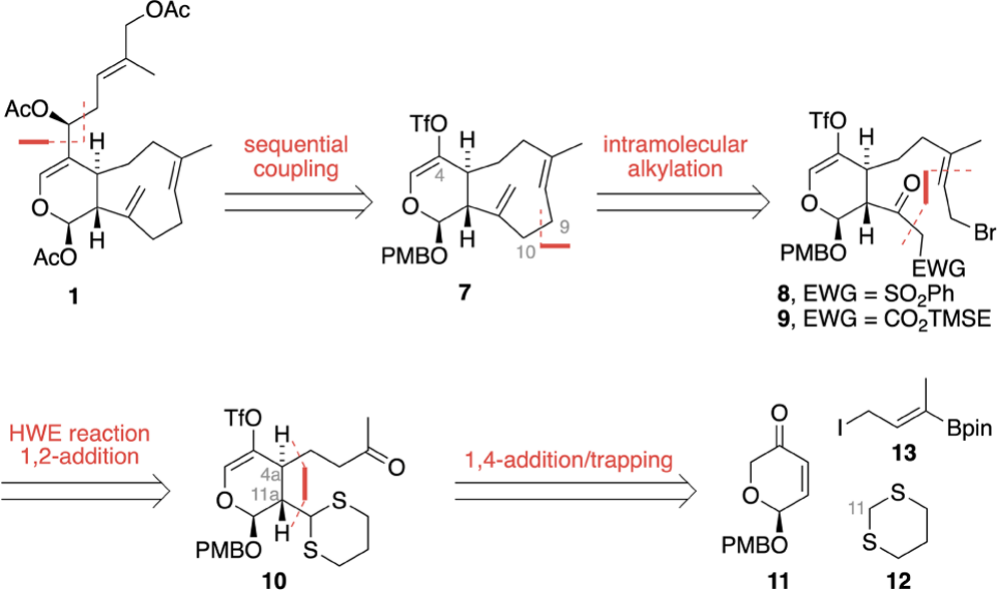
Retrosynthetic Strategy

Our retrosynthetic analysis aimed for maximum side chain flexibility to access different xenicin natural products at a late stage and was initially guided by the 9-membered ring for which several disconnections were evaluated (Scheme 1). Inspiration came from the intramolecular Pd-catalyzed Tsuji–Trost reaction that Corey has employed in the synthesis of antheliolide A (**3**)^8^ as well as the intramolecular alkylation developed by us to install the 9-membered ring of cornexistin.^19^ For the adaption of this maneuver to **1**, we dissected **1** into two fragments: the linear and functionalized side chain attached to C4 and the 6,9-*trans*-fused building block **7** with a triflate functionality which was foreseen to serve a dual purpose. First, the triflate was considered as an ideal handle to enable attachment of various side chains at C4, and second, it was envisioned to deactivate the labile enol acetal. For the installation of the *trans*-fused oxabicyclo[7.4.0]tridecane, the 9-membered ring of **7** was disconnected between C9 and C10 to reveal allylic bromides **8** (EWG = SO_2_Ph) and **9** (EWG = CO_2_TMSE; TMSE = trimethylsilylethyl). Further simplification via removal of the activated ketone function and the trisubstituted alkene unit revealed **10**. For **10**, we envisioned setting the stereocenters at C11a and C4a by conjugate addition of dithiane **12** to enone **11** and in situ trapping of the enolate with the methyl vinyl ketone equivalent **13**.

### Initial Studies toward the Bicyclic System

**Scheme 2:**
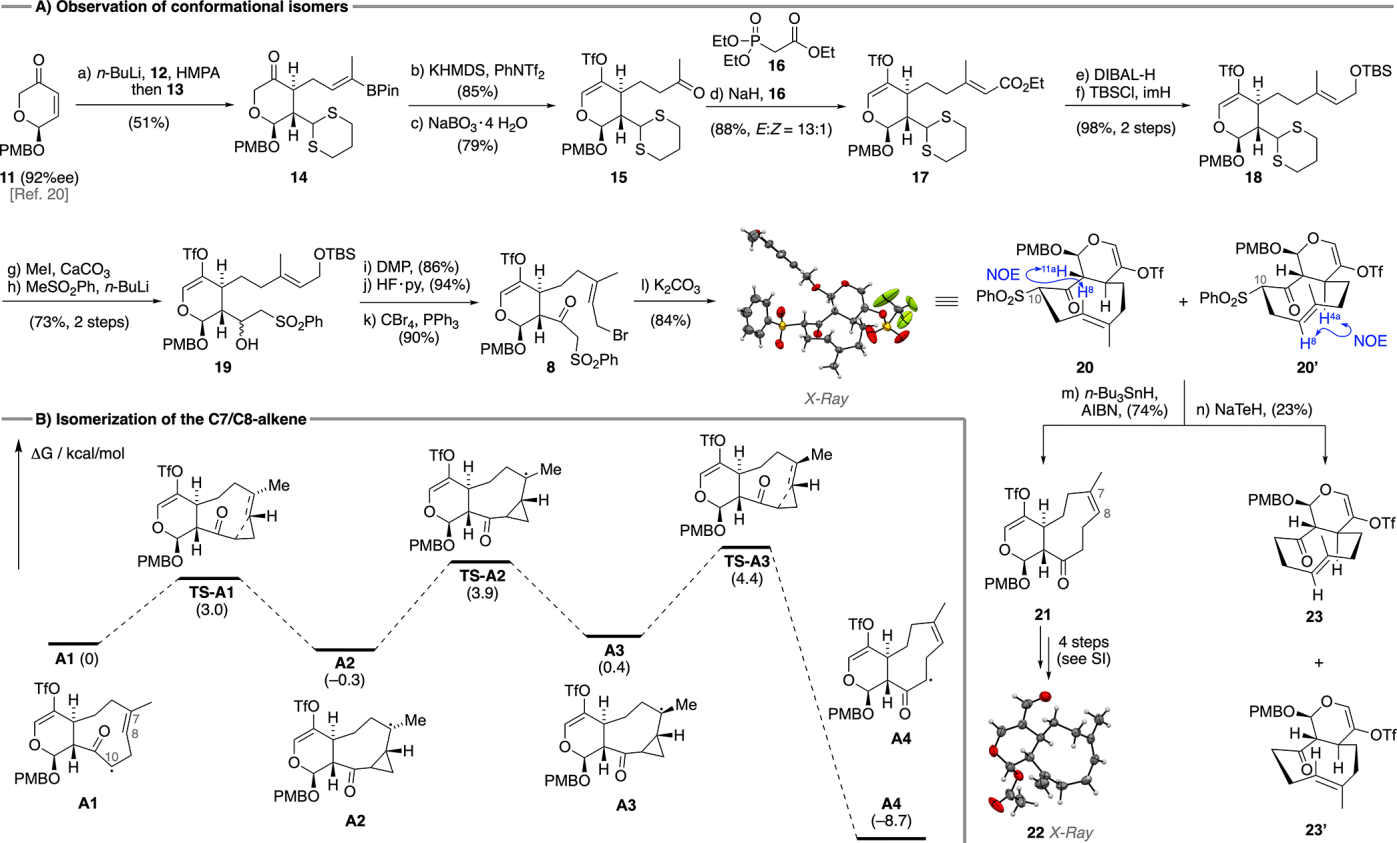
Asymmetric Synthesis of the Oxabicyclo[7.4.0]tridecane System and Isomerization of the C7/C8-Alkene

The synthesis (Scheme 2A) began with the conjugate addition of lithiated dithiane **12** to readily available enone **11** (92% ee, three steps from furfuryl alcohol).^20^ We found that the presence of hexamethyl phosphoramide (HMPA) as a cosolvent was crucial to suppress the competing 1,2-addition.^21^ Under the optimized conditions, the addition of **13** to the enolate was performed at −78 °C, followed by slow warming of the reaction mixture to −35 °C to furnish ketone **14** in 51% yield as a single diastereomer.

Iodide **13** (three steps from ethyl 2-butynoate) represents a previously unknown but valuable alternative to the classical Stork–Jung vinylsilane.^22^ Treating a mixture of **14** and phenyl bistriflimide (PhNTf_2_) with potassium hexamethyldisilazide (KHMDS) gave the corresponding enol triflate in excellent yield (85%). Oxidative cleavage of the vinyl boronate using sodium perborate tetrahydrate unmasked the ketone function of **15**. A subsequent Horner–Wadsworth–Emmons (HWE) reaction with phosphonate **16** affected two-carbon elongation of the side chain to give (*E*)-**17** in 83% yield. Reduction of ester **17** and silylation provided **18** in 98% over two steps. With **18** in hand, we turned our focus toward the introduction of the β-keto sulfone moiety. To this end, we first had to investigate the cleavage of dithiane to liberate the aldehyde function. While methods relying on mercury salts either led to no reaction or to the decomposition of **18**, treatment with methyl iodide and calcium carbonate in a mixture of acetonitrile and water at 55 °C allowed us to liberate the delicate aldehyde function. The obtained product was found to be unstable and underwent rapid decomposition upon purification by flash column chromatography on silica gel. Conducting the reaction at 23 °C suppressed the decomposition of the aldehyde and allowed its utilization without further purification. Since direct conversion of the aldehyde to a β-keto sulfone via the Roskamp reaction (diazomethyl phenyl sulfone, SnCl_2_)^23^ was met with failure, we opted for its synthesis through a 1,2-addition/oxidation procedure. Exposure of the aldehyde to lithiated methyl phenyl sulfone delivered **19** as an inconsequential mixture of diastereomers (73%, 2 steps). A subsequent high-yielding three-step sequence (oxidation, desilylation, and Appel reaction) enabled the conversion of **19** to the cyclization precursor **8**. Gratifyingly, treatment of **8** with potassium carbonate induced smooth cyclization to the 9-membered ring giving the oxabicyclo[7.4.0]tridecane system **20** as an inseparable 2:1 mixture of two compounds in 84% yield. We initially attributed this to the configuration of the sulfone moiety residing in the α-position C10 of the ketone. However, careful analysis of the 2D-NMR nuclear Overhauser effect spectroscopy (NOESY) data of both components revealed two key NOE correlations: between H8 and H11a for the major component **20** and between H8 and H4a for **20′**. With H4a and H11a residing on different sides of the 9-membered ring, we concluded that conformational isomers (atropisomers) with regard to the spatial orientation of the C7/C8-alkene unit were formed. Previously, Guella^24^ made similar observations for closely related *Xenia* diterpenoids, and Williams^10^ was also confronted with conformational isomers in his efforts toward 4-hydroxydictyolactone (**5**). Finally, we were able to crystallize the major conformer **20** from the obtained mixture, giving crystals suitable for single-crystal X-ray analysis. Comparison of the obtained structure with the single-crystal X-ray structure of (+)-waixenicin B allowed us to validate its non-natural product-like conformation.^1^

### Isomerization of the C7/C8-Alkene

Having accomplished the construction of the *trans*-fused oxabicyclo[7.4.0]tridecane ring system, we investigated the removal of the sulfone moiety of **20**. While standard methods resulted in decomposition (e.g., Al/Hg)^8^ or complex mixtures (e.g., SmI_2_), radical desulfonylation utilizing tributyltin hydride and azobisisobutyronitrile (AIBN) resulted in clean transformation to a single product.^25^ To our surprise, careful analysis of the NOESY data showed that the C7/C8-alkene unit underwent complete (*E*)/(*Z*)-isomerization to furnish **21** as a single conformer. We hypothesized that the isomerization was a result of a transient cyclopropyl radical emerging from the attack of a C10 radical at C8 of the alkene. This was also plausible from the single-crystal X-ray structure of **20** showing close proximity between C10 and C8 (2.44 Å). As illustrated in Scheme 2B, further support was obtained when the potential isomerization pathway was investigated by computational studies at the B3LYP-D3/6-311++G(2d,2p) level of theory. As the starting point, we chose the α-keto radical **A1**, which should arise through radical desulfonylation from **20**. We found that **A1** undergoes facile 3-*exo*-trig cyclization (Δ*G*^⧧^ = 3.0 kcal/mol, **TS-A1**) via C8 to give the tertiary radical **A2**. Low-barrier rotation of the methyl group allows for rapid interconversion of the isomeric radicals **A2** and **A3** via **TS-A2**. Cyclopropane opening from **A3** to **A4** leads to the observed (*Z*)-substituted alkene. **A4** represents the thermodynamically most stable intermediate along the reaction pathway for which facile conversion of all radical intermediates was observed (Δ*G*^⧧^ < 5.0 kcal/mol). The reported photoisomerization of a structurally related xenicane system to the crenulatane framework also corroborates the intermediacy of a cyclopropyl radical.^26^ The depicted structure of **21** was finally validated by single-crystal X-ray analysis of acetate **22** (see the Supporting Information (SI) for details). Efforts to avoid the detrimental isomerization showed that sodium hydrogen telluride^27^ affects anionic desulfonylation of **20** to ketone **23** (**23:23′** = 1.2:1) without detectable alkene isomerization. The relative energies of **23**, **23′**, and **21** are in good agreement with the results obtained for **A1** and **A4** (Scheme 2B), favoring the (*Z*)-alkene by 5.0–6.2 kcal/mol (see the SI for details). We additionally calculated the barrier for the interconversion of the two conformers **23** and **23′**. The obtained value of 20.3 kcal/mol supports the values obtained in a previous study.^24^ Trying to obtain further support for an intramolecular radical isomerization pathway, we exposed **23** to AIBN/*n*-Bu_3_SnH. However, isomerization of the (*E*)-alkene was not observed under these conditions. While the use of sodium hydrogen telluride prevented the detrimental isomerization, the reaction suffered from low yields (23%) and limited scalability. A survey of possible alternatives for the sulfone unit ultimately revealed a TMSE ester as the best option.

### Completion of the Total Syntheses

**Scheme 3:**
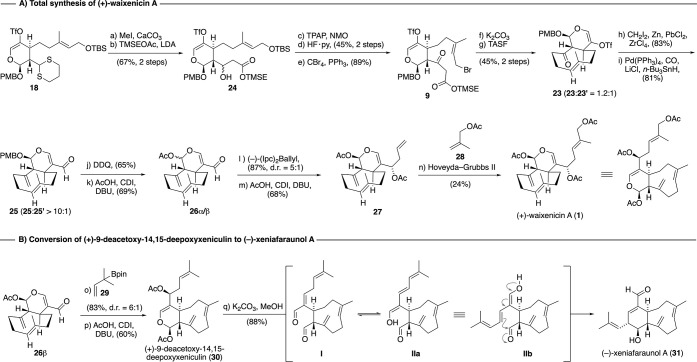
Completion of the Syntheses

The synthesis of the required alkylation precursor commenced with deprotection of dithiane **18**, followed by the addition of the enolate derived from 2-(trimethylsilyl)ethyl acetate (TMSEOAc), to furnish **24** in 67% yield over two steps (Scheme 3A). Oxidation employing Ley conditions,^28^ desilylation, and Appel reaction delivered the modified cyclization precursor **9**. Exposure of **9** to the previously established conditions (K_2_CO_3_, MeCN) induced smooth cyclization to close the 9-membered ring. The obtained crude product was pure enough to be used in the following fluoride-mediated decarboxylation, for which tris(dimethylamino)sulfonium difluorotrimethylsilicate (TASF) proved most efficient. The ketone **23** was formed in 45% yield over two steps as an inseparable mixture of two conformers (**23**:**23′** = 1.2:1). Surprisingly, the following methenylation of the ketone proved to be highly challenging as standard methods failed (see the SI for details).

We eventually found that Zr-based Takai–Lombardo conditions (Zn, CH_2_I_2_, PbCl_2_, ZrCl_4_) were able to deliver the product in 83% yield.^29^ These conditions were crucial as the use of the titanium-based protocol (Zn, CH_2_Br_2_, TiCl_4_) resulted in partial (*E*)/(*Z*)-isomerization of the C7/C8-alkene. Interestingly, installation of the *exo*-methylene was accompanied by equilibration of the conformational isomers, with the natural product-like conformation now constituting the prevalent species (ratio = 10:1). With the manipulations on the *trans*-fused bicyclic core being completed, we turned our focus toward the side chain introduction. To begin with, the enol triflate was subjected to one-carbon elongation via carbonylation to give aldehyde **25** in 81% yield (**25:25′** > 10:1). The generated push–pull system was essential for the ensuing *p*-methoxybenzyl (PMB) ether cleavage as decomposition was otherwise observed (see the SI for details). Surprisingly, the anomeric alcohols (d.r. = 1.8:1) were exceptionally reluctant to undergo acetylation, and only a combination of 1,1′-carbonyldiimidazole (CDI, Staab’s reagent), acetic acid, and catalytic 1,8-diazabicyclo[5.4.0]undec-7-ene (DBU) was found to give **26α** and **26β** in equimolar amounts. The undesired acetate **26α** was recycled via saponification (72%) and acetylation (69%). For the completion of the synthesis of (+)-waixenicin A (**1**), installation of the oxidized prenyl side chain was required. Initial studies with a model system were disappointing as the attempted Mukaiyama aldol reaction or reductive addition of isoprene monoxide (2-methyl-2-vinyloxirane) was met with failure (see the SI for details). Therefore, we continued with the Brown allylation^30^ of **26β** to deliver a 5:1 mixture of diastereomers in 87% yield. Acetylation of the major diastereomer gave allylic acetate **27**, which was subjected to a challenging type I/type III olefin cross-metathesis^31^ with methallyl acetate (**28**). Waixenicin A (**1**) was isolated in 24% yield from the complex product mixture. To our delight, the spectral data of synthetic **1** were in full agreement with the NMR data obtained by the Horgen group and the isolation reports.^1^ It is noteworthy that rapid ^13^C NMR data acquisition for shift comparison was required as a solution of waixenicin A (**1**) in CD_2_Cl_2_ showed substantial decomposition to unidentified byproducts after 1 h. However, **1** proved to be stable in CDCl_3_ or C_6_D_6_ for at least 72 h. With aldehyde **26β** in hand, the stage was set for further diversification. As illustrated in Scheme 3B, prenylation of **26β** employing boronate **29**(32) proceeded in 83% yield (d.r. = 6:1), and subsequent acetylation of the major diastereomer gave access to 9-deacetoxy-14,15-deepoxyxeniculin (**30**). Finally, treatment of **30** with potassium carbonate induced rapid rearrangement to xeniafaraunol A (**31**), which was first isolated by *Kashman* in 1994 and displayed moderate cytotoxicity against P388 cells (IC_50_ = 3.9 μM).^18^ A plausible mechanistic scenario for the rearrangement involves deacetylation/elimination to dialdehyde **I**, γ-deprotonation to give trienol **IIa**, followed by the vinylogous aldol reaction via conformer **IIb**. This rearrangement could be of potential interest with regard to the stability as well as the mode of action of waixenicin A (**1**). While **1** was shown to irreversibly inhibit the TRPM7 channel, the mode of binding is yet unknown.^33^ Long-term cell growth assays, however, revealed a reduced inhibitory effect of **1**.^3^ Whether this is due to binding to serum proteins or a result of degradation via a similar rearrangement is under debate. In initial model experiments, 9-deacetoxy-14,15-deepoxyxeniculin (**30**) was exposed to lysine or cysteine as biological nucleophiles. Under physiological conditions (pH 7 buffer, 37 °C), neither rearrangement to **31** was induced nor was the formation of covalent adducts observed. For both nucleophiles, only unreacted **30** was recovered from the reaction mixture. Further experimentation in cellular systems will be required to elucidate the binding to the TRPM7 channel in detail.

## Conclusions

In conclusion, we have completed the first total syntheses of the xenicin natural products waixenicin A (**1**) and 9-deacetoxy-14,15-deepoxyxeniculin (**30**). For the installation of the stereocenters at C11a and C4a, a highly diastereoselective conjugate addition/trapping sequence was employed. This step also introduces a valuable alternative to the classical Stork–Jung vinylsilane. The characteristic 9-membered carbocycle of the natural products was constructed by a powerful intramolecular alkylation reaction. Deactivation of the labile enol acetal by incorporating a triflate at an early stage of the synthesis enabled the late-stage introduction of the side chain. In addition to providing access to **30**, we accomplished its base-mediated one-step rearrangement to xeniafaraunol A (**31**). The ease of this transformation raises further questions about the biosynthesis of **31** and related natural products such as xenibecin. The developed strategy represents the first synthetic entry to the xenicin class of *Xenia* diterpenoids. Current work in our laboratory focuses on late-stage diversification to access additional members of this natural product family as well as fully synthetic analogues for a broad bioactivity screening campaign against TRPM channels.

## Supporting Information Available

Experimental procedures, computational details, and characterization data for all compounds and X-ray crystallographic data for **20** and **22** (PDF)
