# Supplementary material for: Total Syntheses of (+)-Waixenicin A, (+)-9-Deacetoxy-14,15-deepoxyxeniculin, and (−)-Xeniafaraunol A
Source: J Am Chem Soc. 2023 May 16;145(21):11811–7. doi: 10.1021/jacs.3c03366 (PMC7614607; doi:10.1021/jacs.3c03366)
Supplement: Supplementary file 1 — ja3c03366_si_001.pdf [file ja3c03366_si_001.pdf]

Supporting Information

Total Syntheses of (+)-Waixenicin A, (+)-9-Deacetoxy-14,15-deepoxyxeniculin and (–)-Xeniafaraunol A

Christian Steinborn[†], Tatjana Huber[†], Julian Lichtenegger[†], Immanuel Plangger[†], Klaus Wurst[§], Thomas Magauer[*,†]

[†]Institute of Organic Chemistry and Center for Molecular Biosciences, University of Innsbruck, Innrain 80–82, 6020 Innsbruck, Austria

[§]Institute of General, Inorganic & Theoretical Chemistry, University of Innsbruck, Innrain 80–82, 6020 Innsbruck, Austria
Corresponding author: thomas.magauer@uibk.ac.at

Table of Contents

1 General Experimental Details 3

2 Experimental section 5

2.1 Synthesis of dithiane 18 5

Pyranone *rac*-S2 5
Enantioenriched Pyranone S2 5
Enantioenriched Pyranone 11 7
Ethyl Ester S4 8
Iodide 13 9
Ketone 14 10
Triflate S6 11
Ketone 15 12
Ethyl Esters 17 and S7 13
Silyl ether 18 15

2.2 Sulfone approach 17

Sulfone 19 17
Sulfone S10 19
Alcohol S11 20
Bromide 8 21
Sulfone 20 22
Ketone 21 24
Alkene S12 25
Aldehyde S13 26
Acetates 22 and S15 27

2.3 Total synthesis of waixenicin A, 9-deacetoxy-14,15-deepoxyxeniculin and xeniafaraunol A 30

Acetate S17 30
Alcohols 24a and 24b 31
Alcohol S19 33
Bromide 9 35
Ketones 23 36
Alkene S21 38
Aldehyde 25 40
Hemiacetal S22α/β 41
Acetates 26α and 26β 42
Recycling of S26α 43
Alcohols S23a and S23b 44
Allylic acetate 27 45
Waixenicin A (1) 46
Key insights for the side chain introduction 50
Model substrate S24 53
Allylic chloride S35 54
Sulfone S32 55
Allylic chloride S33 56
Sulfone S31 57
Alcohol S38a and S38b 59
9-deacetoxy-14,15-deepoxyxeniculin 30 60
Xeniafaraunol A 31 63

3 Computational Studies 66

3.1 Computational Methodology 66

3.2 Alkene isomerization 66

3.3 Nine-membered ring conformations 67

3.4 Cartesian Coordinates 68

4 X-ray data 90

4.1 Sulfone 20 90

4.2 Acetate 22 91

5 References 92

^{1}H and ^{13}C NMR Spectra 94

1 General Experimental Details

All reactions were carried out with magnetic stirring, and if moisture or air sensitive, under nitrogen or argon atmosphere using standard Schlenk techniques in oven-dried glassware (120 °C oven temperature). If required glassware was further dried under vacuum with a heat-gun at 650 °C. External bath thermometers were used to record all reaction temperatures. Low temperature reactions were carried out in a Dewar vessel filled with acetone/dry ice (T between –78 °C and 0 °C) or distilled water/ice (0 °C). High temperature reactions were conducted using a heated silicon oil bath or a metal block in reaction vessels equipped with a reflux condenser or in a pressure tube. Tetrahydrofuran (THF) was distilled over sodium/potassium alloy prior to use. All other solvents were purchased from Acros Organics as 'extra dry' reagents. All other reagents with a purity >95% were obtained from commercial sources (Sigma Aldrich, Acros, Alfa Aesar and others) and used without further purification unless otherwise stated.

Flash column chromatography (FCC) was carried out with Merck silica gel 60 (0.040–0.063 mm). Analytical thin layer chromatography (TLC) was carried out using Merck silica gel 60 F254 glass-backed plates or aluminum foils and visualized under UV-light at 254 nm. Staining was performed with ceric ammonium molybdate (CAM), an aqueous potassium permanganate solution or by staining with an aqueous *para*-anisaldehyde solution and subsequent heating.

NMR spectra (^{1}H NMR, ^{13}C NMR and ^{19}F NMR) were recorded in deuterated chloroform ($CDCl_3$), deuterated methanol (CD_3OD) or deuterated benzene (C_6D_6) on a Bruker Avance III HD 400 MHz spectrometer equipped with a CryoProbe™, a Bruker Avance Neo 400 MHz spectrometer, an Agilent 500 DD2 500 MHz spectrometer or a Bruker Avance II 600 MHz spectrometer and are reported as follows: chemical shift δ in ppm (multiplicity, coupling constant *J* in Hz, number of protons) for ^{1}H NMR spectra and chemical shift δ in ppm for ^{13}C NMR spectra. Multiplicities are abbreviated as follows: s = singlet, d = doublet, t = triplet, q = quartet, p = quintet, br = broad, m = multiplet, or combinations thereof. Residual solvent peaks of $CDCl_3$ (δ_H = 7.26 ppm, δ_C = 77.16 ppm), CD_3OD (δ_H = 3.31 ppm, δ_C = 49.00 ppm) and C_6D_6 (δ_H = 7.16 ppm, δ_C = 128.06ppm) were used as internal reference. NMR spectra were assigned using information ascertained from 2D–NMR experiments. ^{19}F–NMR spectra were externally referenced ($CFCl_3$).

High resolution mass spectra (HRMS) were recorded on Thermo Scientific™ LTQ Orbitrap XL™ Hybrid Ion Trap-Orbitrap Mass Spectrometer at the Institute of Organic Chemistry and Center for Molecular Biosciences, University of Innsbruck.

Infrared spectra (IR) were recorded from 4000 cm^{-1} to 450 cm^{-1} on a Bruker™ ALPHA FT-IR Spectrometer from Bruker. Samples were prepared as a neat film or a film by evaporation of a solution in $CDCl_3$.

Optical rotation values were recorded on a Schmidt+Haensch UniPol L1000 Peltier polarimeter. The specific rotation is calculated as follows: $[\alpha]_\lambda^T = \frac{\alpha \times 100}{c \times d}$. Thereby, the wavelength λ is reported in nm and the measuring temperature in °C. α represents the recorded optical rotation, *c* the concentration of the analyte in 10 mg/mL and *d* the length of the cuvette in dm. Thus, the specific rotation is given in $10^{-1}\cdot deg\cdot cm^2\, g^{-1}$. Use of the sodium *D* line (λ = 589 nm) is indicated by *D* instead of the wavelength in nm. The sample concentration as well as the solvent is reported in the relevant section of the experimental part.

For **X-ray diffraction analysis**, data collections were performed on a Bruker D8Quest using MoK_αradiation (λ = 0.71073 Å, Incoatec Microfocus). The Bruker Apex III software was applied for the integration, scaling and multi-scan absorption correction of the data. Structures were solved by direct methods with SHELXTL-XT-2014. Structure refinement was performed by least-squares methods against F2 with SHELXL-2014/7. All non-hydrogen atoms were refined anisotropically. The hydrogen atoms were placed in ideal geometry riding on their parent atoms. Relevant details of the data collection and evaluation are listed in tables at the corresponding sections. Supplementary crystallographic data for **20** (CCDC 2244638) and **22** (CCDC 2244639), can be obtained from the Cambridge Crystallographic Data Centre CCDC deposition service via www.ccdc.cam.ac.uk/structures on quoting the deposition number CCDC 2244638-2244639. Further details are summarized in the tables at the corresponding sections. Plotting of thermal ellipsoids in this document and in the main text was carried out using MERCURY for Windows at 50% probability level.

All yields are isolated, unless otherwise specified.

2 Experimental section

2.1 Synthesis of dithiane 18

Pyranone *rac*-S2

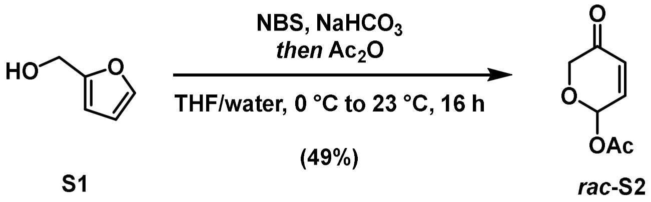

N-bromosuccinimide (96.3 g, 536 mmol, 1.05 equiv) was added in small portions to a suspension of furfuryl alcohol **S1** (45.2 mL, 510 mmol, 1 equiv) and sodium bicarbonate (86.6 g, 1.02 mol, 2.00 equiv) in a mixture of tetrahydrofuran (300 mL) and water (75 mL) at 0 °C. After 30 min, acetic anhydride (95.8 mL, 1.02 mol, 2.00 equiv) was added and the reaction mixture was allowed to warm to 23 °C. After 16 h, solid sodium bicarbonate and saturated aqueous solution of sodium bicarbonate were added and the reaction mixture was poured into dichloromethane (450 mL). The layers were separated and the aqueous phase was extracted with dichloromethane (2 × 300 mL). The combined organic phases were dried over magnesium sulfate and the dried solution was filtrated. The filtrate was concentrated and the residue was purified by flash column chromatography on silica gel (30% diethyl ether in pentane) to give acetate **S1** (38.9 g, 49%) as an orange oil.

^{1}H–NMR (400 MHz, $CDCl_3$): δ = 6.92 (dd, *J* = 10.4, 3.6 Hz, 1H), 6.49 (d, *J* = 3.6 Hz, 1H), 6.27 (d, *J* = 10.4 Hz, 1H), 4.51 (d, *J* = 17.0 Hz, 1H), 4.22 (d, *J* = 17.0 Hz, 1H), 2.14 (s, 3H) ppm.
The obtained analytical data were in full agreement with those reported in the literature.[1]

Enantioenriched Pyranone S2

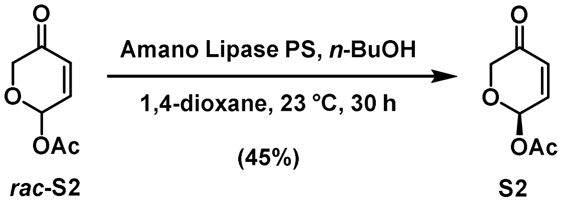

Amano Lipase PS (immobilized on diatomite, 11.4 g) was added to a solution of acetate ***rac*-S2** (54.8 g, 351 mmol, 1 equiv) in a mixture of *n*-butanol (160 mL, 1.75 mol, 5.00 equiv) and 1,4-dioxane (1.5 L) at 23 °C. After 30 h, the reaction mixture was filtered through a plug of Celite. The filtercake was thoroughly washed with diethyl ether (500 mL). The filtrate was concentrated and the residue was purified by flash column chromatography on silica gel (30% diethyl ether in pentane grading to 50% diethyl ether in pentane) to give enantioenriched acetate **S2** (24.6 g, 45%) as a colorless oil.[2]

¹H–NMR (400 MHz, $CDCl_3$): δ = 6.92 (dd, *J* = 10.4, 3.6 Hz, 1H), 6.49 (d, *J* = 3.6 Hz, 1H), 6.27 (d, *J* = 10.4 Hz, 1H), 4.51 (d, *J* = 17.0 Hz, 1H), 4.22 (d, *J* = 17.0 Hz, 1H), 2.14 (s, 3H) ppm.

The obtained analytical data were in full agreement with those reported in the literature.[2]

An *ee* of around 91% (close elution of both enantiomers, made a more accurate determination not possible; the *ee* of the Tsuji–Trost product was determined to be 92%; see below) was determined by Liquid Chromatography conducted on a Daicel Chiralpak IB column (Shimadzu LC-2030, 1 mL/min flow rate, isocratic elution, 2% isopropanol in *n*-hexane, λ = 210 nm, tR (minor) = 17.4 min, tR (major) = 16.2 min).

***rac*-S2**.

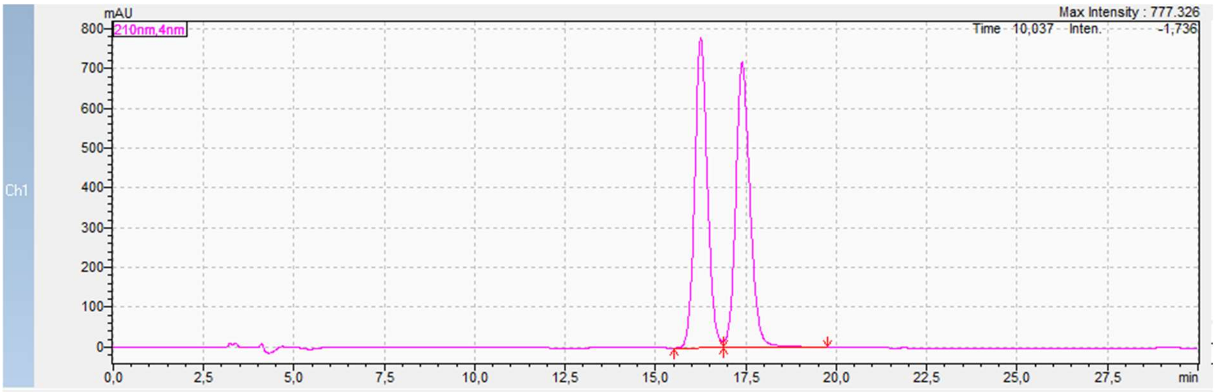

Results View - Peak Table

Peak Table | Compound | Group | Calibration Curve

Peak#	Ret. Time	Area	Height	Mark	Area%
1	16,250	18955181	779343		49,089
2	17,395	19659092	717570	V	50,911
Total		38614274	1496913		100,000

Figure S1: LC-trace of ***rac*-S2**.

S2:

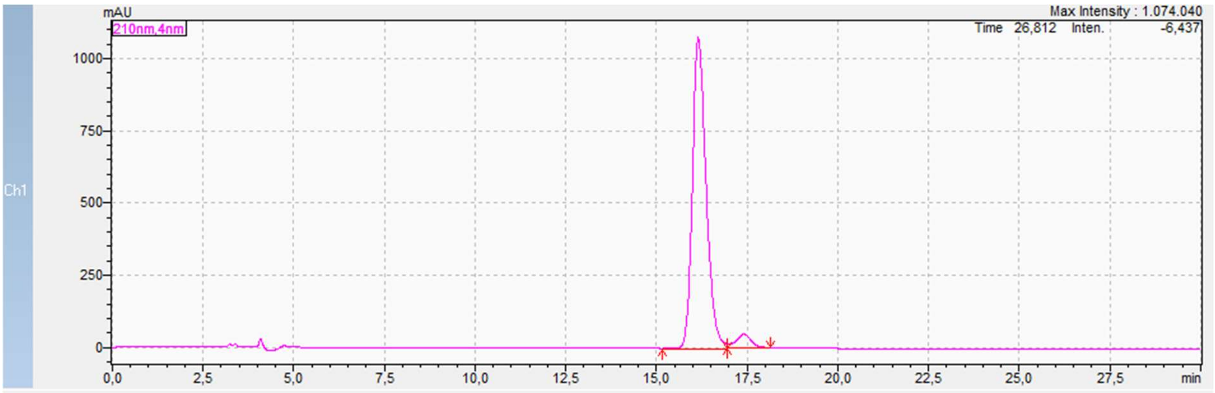

Results View - Peak Table

Peak Table | Compound | Group | Calibration Curve

Peak#	Ret. Time	Area	Height	Mark	Area%
1	16,150	28282109	1078273	M	95,421
2	17,412	1357028	46627	M	4,579
Total		29639138	1124900		100,000

Figure S2: LC-trace of enantioenriched **S2**.

Enantioenriched Pyranone 11

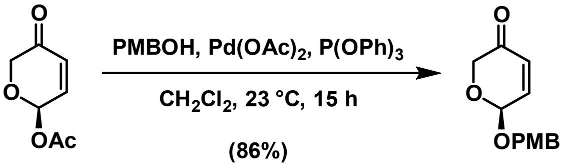

S2 11

Triphenyl phosphite (9.08 mL, 34.7 mmol, 0.220 equiv) was added to a suspension of palladium(II) acetate (3.54 g, 15.8 mmol, 0.100 equiv) in dichloromethane (270 mL) at 23 °C. After 10 min, *p*-methoxybenzyl alcohol (29.4 mL, 236 mmol, 1.50 equiv) was added to the reaction mixture. After 10 min, the black reaction mixture was cooled to –30 °C, a solution of acetate **S2** (24.6 g, 158 mmol, 1 equiv) in dichloromethane (140 mL) was added and the flask was wrapped with aluminum foil. After 15 h, the reaction mixture was concentrated and the residue was purified by flash column chromatography on silica gel (20% diethyl ether in pentane) to give enone **11** (31.8 g, 86%) as a yellow oil.

^{1}H–NMR (400 MHz, $CDCl_3$): δ = 7.30 (d, *J* = 8.5 Hz, 2H), 6.90 (d, *J* = 8.6 Hz, 2H), 6.87 (dd, *J* = 10.4, 3.4 Hz, 1H), 6.14 (d, *J* = 10.3 Hz, 1H), 5.27 (d, *J* = 3.4 Hz, 1H), 4.79 (d, *J* = 11.4 Hz, 1H), 4.60 (d, *J* = 11.4 Hz, 1H), 4.48 (d, *J* = 16.8 Hz, 1H), 4.12 (d, *J* = 16.8 Hz, 1H), 3.82 (s, 3H) ppm.

The obtained analytical data were in full agreement with those reported in the literature.[2]

An *ee* of 92% was determined by Liquid Chromatography conducted on a Daicel Chiralpak IB column (Shimadzu LC-2030, 1 mL/min flow rate, isocratic elution, 2% isopropanol in *n*-hexane, λ = 210 nm, tR (minor) = 17.1 min, tR (major) = 20.9 min). A racemic reference sample was prepared through Tsuji–Trost reaction of racemic acetate ***rac*-S2**.

***rac*-11**:

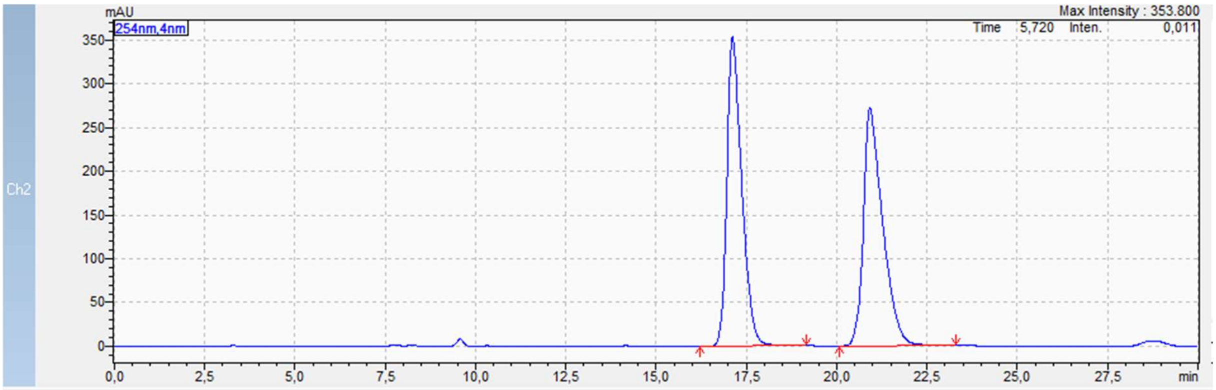

Results View - Peak Table

Peak Table | Compound | Group | Calibration Curve

Peak#	Ret. Time	Area	Height	Mark	Area%
1	17,116	10139432	354167		50,164
2	20,918	10073018	271681		49,836
Total		20212450	625848		100,000

Figure S3: LC-trace of racemic **11**.

enantioenriched **11**:

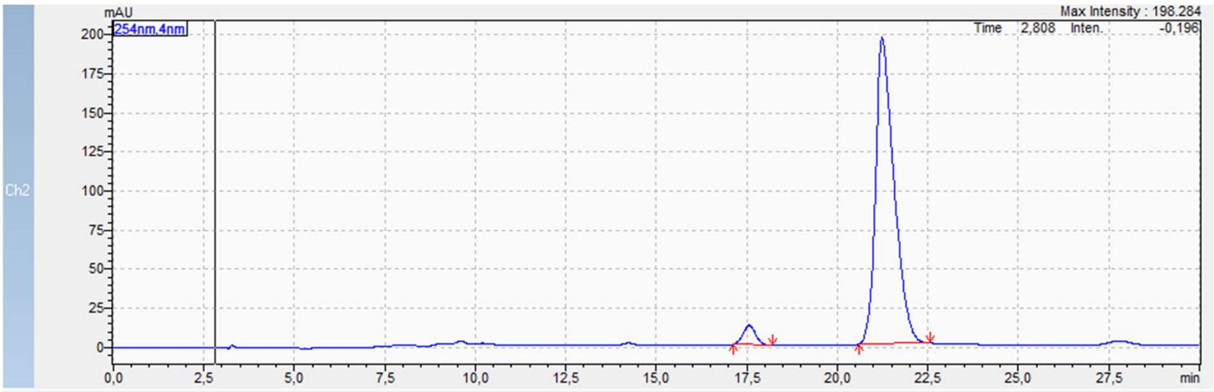

Results View - Peak Table

Peak Table | Compound | Group | Calibration Curve

Peak#	Ret. Time	Area	Height	Mark	Area%
1	17,567	292480	12388	M	4,053
2	21,237	6924276	195943	M	95,947
Total		7216756	208332		100,000

Figure S4: LC-trace of enantioenriched **11**.

Ethyl Ester S4

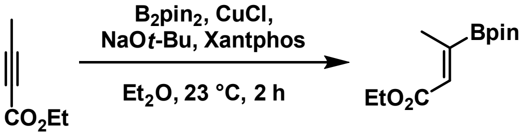

S3 **S4**

Xantphos (3.91 g, 6.55 mmol, 3.00 mol%) was added to a suspension of copper(I) chloride (655 mg, 6.55 mmol, 3.00 mol%) and sodium *tert*-butoxide (1.29 g, 13.1 mmol, 6.00 mol%) in tetrahydrofuran (80 mL) at 23 °C. After 30 min, a solution of bis(pinacolato)diboron (61.0 g, 240 mmol, 1.10 equiv) in tetrahydrofuran (50 mL) was added to the brown reaction mixture. After 10 min, ethyl 2-butynoate **S3** (25.0 g, 218 mmol, 1 equiv) and methanol (17.7 mL, 437 mmol, 2.00 equiv) were added to the black reaction mixture and it was cooled to 0 °C. After 20 min, the reaction mixture was allowed to warm to 23 °C. After 1.5 h the reaction mixture was filtered through a plug of Celite, which was flushed with diethyl ether (200 mL). The filtrate was concentrated and the residue was purified by flash column chromatography on silica gel (10% diethyl ether in pentane) to give ester **S4** (51.8 g, 99%) as a colorless oil.

^{1}H–NMR (400 MHz, $CDCl_3$): δ = 6.44 (q, *J* = 1.8 Hz, 1H), 4.17 (q, *J* = 7.2 Hz, 2H), 2.16 (d, *J* = 1.8 Hz, 3H), 1.29 – 1.25 (m, 15H) ppm.
The obtained analytical data were in full agreement with those reported in the literature.[3]

Iodide 13

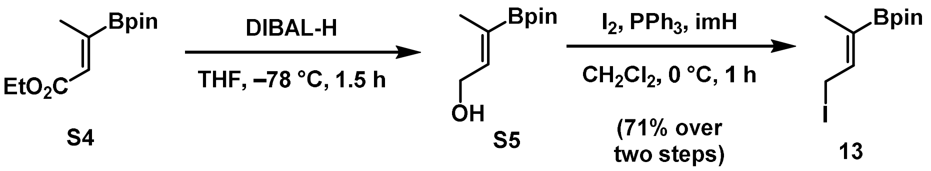

Note: The reduction was carried out in two parallel batches, which were combined during the Rochelle salt work-up.

A solution of diisobutylaluminium hydride (1.00 M in dichloromethane, 154 mL, 154 mmol, 2.20 equiv) was added slowly to a solution of ester **S4** (16.8 g, 70.0 mmol, 1 equiv) in dichloromethane (400 mL) at –78 °C. After 1.5 h, the reaction mixture of both on parallel conducted reaction runs was poured into an aqueous saturated sodium potassium tartrate solution (900 mL) and the mixture was stirred vigorously for 1 h. The layers were separated and the aqueous phase was extracted with dichloromethane (2 × 300 mL). The combined organic phases were dried over magnesium sulfate and the dried solution was filtrated. The filtrate was concentrated to give crude alcohol **S5** (23.7 g), which was used in the following oxidation step without further purification as a colorless oil.

Triphenylphosphine (47.5 g, 179 mmol, 1.50 equiv) was added to a solution of iodine (46.4 g, 179 mmol, 1.50 equiv) in dichloromethane (700 mL) at 0 °C. After 20 min, imidazole (20.4 g, 299 mmol, 2.50 equiv) was added to the reaction mixture. After 10 min, a solution of alcohol **S5** (assumed pure 23.7 g, 120 mmol, 1 equiv) in dichloromethane (200 mL) was added to the reaction mixture. After 1 h, aqueous saturated sodium thiosulfate solution (500 mL) was added to the reaction mixture. The layers were separated and the aqueous phase was extracted with dichloromethane (2 × 400 mL). The combined organic phases were dried over magnesium sulfate and the dried solution was filtrated. The filtrate was concentrated and the residue was treated with a mixture of pentane and diethylether (9:1, 400 mL). The precipitated triphenylphosphine oxide was removed by filtration over a plug of Celite. The filter cake was thoroughly washed with a mixture of pentane and diethyl ether (9:1, 200 mL). The filtrate was concentrated and the residue was treated with a mixture of pentane and diethylether (19:1, 400 mL). The precipitated triphenylphosphine oxide was removed by filtration over a plug of Celite. The filter cake was thoroughly washed with a mixture of pentane and diethyl ether (19:1, 200 mL). The filtrate was concentrated to give iodide **13** (30.7 g, 71%) as a yellowish oil, which was clean enough to be used in the alkylation step.

TLC (30% diethyl ether in pentane): R_f = 0.71 (UV, Permanganate)

^{1}H–NMR (400 MHz, $CDCl_3$): δ = 6.55 (tq, *J* = 8.6, 1.8 Hz, 1H), 3.94 (d, *J* = 8.6 Hz, 2H), 1.71 (d, *J* = 1.8 Hz, 3H), 1.26 (s, 12H) ppm.

^{13}C–NMR (101 MHz, $CDCl_3$): δ = 139.8, 131.8 (assigned by HMBC), 83.3, 24.5, 12.9, 0.00 ppm.

IR (Diamond-ATR, neat): 2977, 1620, 1366, 1326, 1138, 1085, 860, 850, 670, 542 cm^{-1}.

HRMS (ESI) calc. for $C_{10}H_{18}BINaO_2^+$ $[M+Na]^+$: 331.0337; found: mass not found.

Ketone 14

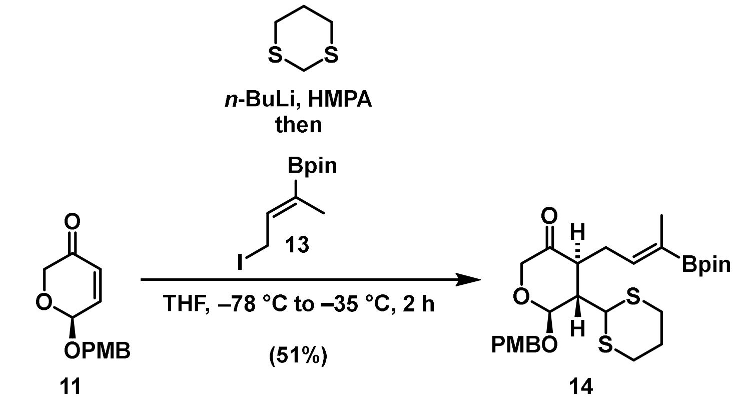

A solution of *n*-butyllithium (2.50 M in hexanes, 24.9 mL, 62.3 mmol, 1.10 equiv) was added dropwise to a solution of 1,3-dithiane (8.07 g, 65.1 mmol, 1.15 equiv) in tetrahydrofuran (500 mL) at –78 °C. After 45 min, hexamethylphosphoramide (19.9 mL, 113 mmol, 2.00 equiv) was added to the reaction mixture. After 15 min, a solution of enone **11** (13.3 g, 56.6 mmol, 1 equiv) in tetrahydrofuran (30 mL) was added to the reaction mixture. After 30 min, iodide **13** (36.6 g, 119 mmol, 2.10 equiv) was added and the yellow reaction mixture was allowed to gradually warm to –35 °C. After 2 h, aqueous saturated ammonium chloride solution (600 mL) and dichloromethane (600 mL) were added to the yellow reaction mixture. The layers were separated and the aqueous phase was extracted with dichloromethane (2 × 400 mL). The combined organic phases were dried over magnesium sulfate and the dried solution was filtrated. The filtrate was concentrated and the residue was purified by flash column chromatography on silica gel (25% diethyl ether in pentane) to give ketone **14** (15.4 g, 51%) as a colorless oil.

TLC (20% diethyl ether in pentane): R_f = 0.20 (UV, CAM).
^{1}H–NMR (400 MHz, $CDCl_3$): δ = 7.33 – 7.27 (m, 2H), 6.92 – 6.83 (m, 2H), 6.26 (tt, *J* = 6.9, 1.8 Hz, 1H), 5.26 (d, *J* = 3.8 Hz, 1H), 4.63 (d, *J* = 11.0 Hz, 1H), 4.55 (d, *J* = 11.0 Hz, 1H), 4.26 (d, *J* = 3.8 Hz, 1H), 4.17 (d, *J* = 17.7 Hz, 1H), 3.97 (d, *J* = 17.7 Hz, 1H), 3.79 (s, 3H), 3.02 (ddd, *J* = 11.0, 6.2, 4.9 Hz, 1H), 2.93 – 2.75 (m, 4H), 2.62 – 2.52 (m, 1H), 2.50 – 2.40 (m, 1H), 2.19 (dt, *J* = 10.8, 3.8 Hz, 1H), 2.15 – 2.07 (m, 1H), 1.94 – 1.78 (m, 1H), 1.74 (d, *J* = 1.8 Hz, 3H), 1.23 (s, 12H) ppm.
^{13}C–NMR (101 MHz, $CDCl_3$): δ = 210.5, 159.4, 142.5, 129.8, 129.5, 128.8 (assigned by HMBC), 113.9, 98.8, 83.3, 70.0, 66.4, 55.4, 50.0, 48.8, 45.2, 31.5, 30.8, 26.1, 25.7, 25.0, 24.9, 14.3 ppm.
IR (Diamond-ATR, neat): 2975, 2931, 1732, 1613, 1515, 1371, 1304, 1248, 1146, 1035 cm^{-1}.
HRMS (ESI) calc. for $C_{27}H_{39}BNaO_6S_2^+$ $[M+Na]^+$: 557.2173; found: 557.2166.
$[\alpha]_D^{20}$: 78.2 (c = 0.45, CH_2Cl_2).

Triflate S6

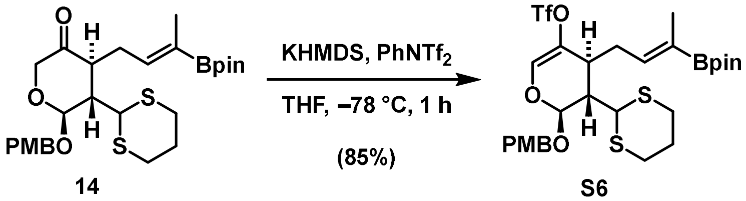

A solution of potassium bis(trimethylsilyl)amide (1.00 M in tetrahydrofuran, 29.3 mL, 29.3 mmol, 1.35 equiv) was added dropwise to a solution of ketone **14** (11.6 g, 21.7 mmol, 1 equiv) and phenyl bistriflimide (14.0 g, 39.1 mmol, 1.80 equiv) in tetrahydrofuran (280 mL) at –78 °C. After 1 h, water (350 mL) and dichloromethane (350 mL) were added to the reaction mixture. The layers were separated and the aqueous phase was extracted with dichloromethane (2 × 200 mL). The combined organic phases were dried over magnesium sulfate and the dried solution was filtrated. The filtrate was concentrated and the residue was purified by flash column chromatography on silica gel (20% diethyl ether in pentane) to give triflate **S6** (12.3 g, 85%) as a colorless oil.

TLC (20% diethyl ether in pentane): R_f = 0.36 (UV, CAM).
^{1}H–NMR (400 MHz, $CDCl_3$): δ = 7.24 (d, *J* = 8.7 Hz, 2H), 6.87 (d, *J* = 8.7 Hz, 2H), 6.69 – 6.62 (m, 1H), 6.37 – 6.23 (m, 1H), 5.57 (d, *J* = 1.5 Hz, 1H), 4.72 (d, *J* = 11.3 Hz, 1H), 4.48 (d, *J* = 11.3 Hz, 1H), 3.80 (s, 3H), 3.57 (d, *J* = 10.7 Hz, 1H), 3.17 (dd, *J* = 10.5, 4.3 Hz, 1H), 2.93 – 2.76 (m, 3H), 2.71 – 2.59 (m, 2H), 2.51 – 2.41 (m, 2H), 2.09 – 1.98 (m, 1H), 1.98 – 1.85 (m, 1H), 1.59 (s, 3H), 1.25 (s, 6H), 1.24 (s, 6H) ppm.
^{13}C–NMR (101 MHz, $CDCl_3$): δ = 159.5, 142.4, 137.2, 135.4, 130.5 (assigned by HMBC), 129.8, 129.1, 118.6 (q, *J* = 320.5 Hz), 114.0, 97.1, 83.4, 70.6, 55.4, 45.6, 42.0, 36.6, 29.6, 27.7, 27.7, 25.8, 25.1, 24.8, 14.1.ppm.
^{19}F–NMR (376 MHz, $CDCl_3$) δ = 73.8 ppm.
IR (Diamond-ATR, neat): 2975, 2931, 1732, 1613, 1515, 1371, 1304, 1248, 1146, 1035 cm^{-1}.
HRMS (ESI) calc. for $C_{28}H_{38}BF_3NaO_8S_3^+$ $[M+Na]^+$: 689.1666; found: 698.1659.
$[\alpha]_D^{20}$: 76.4 (c = 0.55, CH_2Cl_2).

Ketone 15

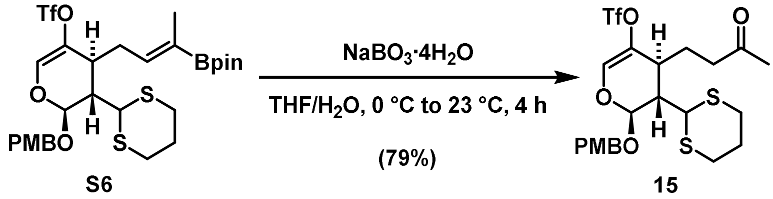

Sodium perborate tetrahydrate (18.8 g, 119 mmol, 9.00 equiv) was added to a solution of triflate **S6** (8.78 g, 13.2 mmol, 1 equiv) in a mixture of tetrahydrofuran (120 mL) and water (15 mL) at 23 °C. After 6 h, water (200mL) and dichloromethane (200 mL) were added to the reaction mixture. The layers were separated and the aqueous phase was extracted with dichloromethane (2 × 150 mL). The combined organic phases were dried over magnesium sulfate and the dried solution was filtrated. The filtrate was concentrated and the residue was purified by flash column chromatography on silica gel (40% diethyl ether in pentane) to give ketone **15** (5.78 g, 79%) as a colorless oil.

TLC (40% diethyl ether in pentane): R_f = 0.40 (UV, CAM).
^{1}H–NMR (400 MHz, $CDCl_3$): δ = 7.22 (d, *J* = 8.7 Hz, 2H), 6.87 (d, *J* = 8.7 Hz, 2H), 6.64 (d, *J* = 1.1 Hz, 1H), 5.49 (d, *J* = 1.7 Hz, 1H), 4.71 (d, *J* = 11.5 Hz, 1H), 4.49 (d, *J* = 11.5 Hz, 1H), 3.81 – 3.78 (m, 4H), 2.97 (dd, *J* = 8.3, 5.4 Hz, 1H), 2.92 – 2.70 (m, 4H), 2.50 (t, *J* = 7.5 Hz, 2H), 2.36 (dt, *J* = 10.2, 1.6 Hz, 1H), 2.21 – 2.00 (m, 6H), 1.97 – 1.83 (m, 1H) ppm.
^{13}C–NMR (101 MHz, $CDCl_3$): δ = 207.7, 159.4, 137.0, 135.5, 129.4, 128.9, 118.5 (q, *J* = 320.5 Hz), 113.9, 96.3, 70.4, 55.3, 47.0, 43.7, 40.7, 36.0, 29.8, 29.4, 29.4, 25.7, 24.8 ppm.
^{19}F–NMR (376 MHz, $CDCl_3$) δ = 73.7 ppm.
IR (Diamond-ATR, neat): 2936, 1716, 1613, 1515, 1247, 1211, 1140, 1070, 825, 608 cm^{-1}.
HRMS (ESI) calc. for $C_{22}H_{27}F_3NaO_7S_3^+$ $[M+Na]^+$: 579.0763; found: 579.0758.
$[\alpha]_D^{20}$: 68.9 (c = 0.53, CH_2Cl_2).

Ethyl Esters 17 and S7

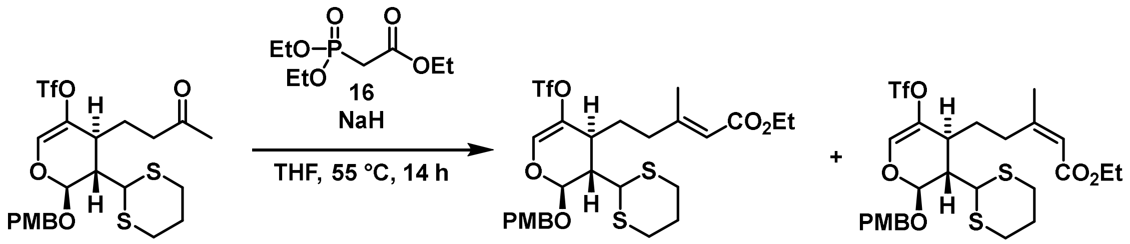

15 17 S7

(83%) (5%)

Sodium hydride (2.79 g, 69.7 mmol, 2.00 equiv) was added in small portions to a solution of phosphonate **16** (14.8 mL, 73.2 mmol, 2.10 equiv) in tetrahydrofuran (360 mL) at 0 °C. After 20 min, a solution of ketone **15** (19.4 g, 34.9 mmol, 1 equiv) in tetrahydrofuran (45 mL) was added to the reaction mixture and it was allowed to warm to 23 °C. After 14 h, water (500 mL) and dichloromethane (600 mL) were added to the reaction mixture. The layers were separated and the aqueous phase was extracted with dichloromethane (2 × 200 mL). The combined organic phases were dried over magnesium sulfate and the dried solution was filtrated. The filtrate was concentrated and the residue was purified by flash column chromatography on silica gel (30% diethyl ether in pentane) to give ester **17** (18.2 g, 83%) and ester **S7** (1.12 g, 5%) as yellowish oils.

17

TLC (40% diethyl ether in pentane): R_f = 0.48 (UV, CAM).

^{1}H–NMR (400 MHz, $CDCl_3$): δ = 7.21 (d, *J* = 8.7 Hz, 2H), 6.87 (d, *J* = 8.7 Hz, 2H), 6.65 (d, *J* = 1.0 Hz, 1H), 5.70 – 5.63 (m, 1H), 5.51 (d, *J* = 1.8 Hz, 1H), 4.72 (d, *J* = 11.4 Hz, 1H), 4.49 (d, *J* = 11.4 Hz, 1H), 4.14 (q, *J* = 7.1 Hz, 2H), 3.84 (d, *J* = 10.1 Hz, 1H), 3.80 (s, 3H), 2.97 (dd, *J* = 8.5, 4.6 Hz, 1H), 2.90 – 2.72 (m, 4H), 2.41 (dt, *J* = 10.1, 1.7 Hz, 1H), 2.28 – 2.17 (m, 1H), 2.17 – 1.99 (m, 6H), 1.99 – 1.83 (m, 2H), 1.27 (t, *J* = 7.1 Hz, 3H) ppm.

^{13}C–NMR (101 MHz, $CDCl_3$): δ = 166.7, 159.4, 158.7, 136.9, 135.5, 129.3, 128.9, 118.5 (q, *J* = 320.5 Hz), 116.2, 113.9, 96.5, 70.5, 59.5, 55.3, 47.1, 43.2, 37.9, 36.4, 29.5 (two carbons), 28.3, 25.7, 18.6, 14.3 ppm.

^{19}F–NMR (376 MHz, $CDCl_3$) δ = 73.8 ppm.

IR (Diamond-ATR, neat): 2935, 1714, 1558, 1418, 1248, 1214, 1142, 1070, 825, 608 cm^{-1}.

HRMS (ESI) calc. for $C_{26}H_{33}F_3NaO_8S_3^+$ $[M+Na]^+$: 649.1182; found: 649.1175.

$[\alpha]_D^{20}$: 49.6 (c = 0.60, CH_2Cl_2).

S7

TLC (40% diethyl ether in pentane): R_f = 0.50 (UV, CAM).

^{1}H–NMR (400 MHz, $CDCl_3$): δ = 7.23 (d, *J* = 8.7 Hz, 2H), 6.85 (d, *J* = 8.7 Hz, 2H), 6.64 (d, *J* = 1.1 Hz, 1H), 5.67 – 5.60 (m, 1H), 5.54 (d, *J* = 1.7 Hz, 1H), 4.72 (d, *J* = 11.5 Hz, 1H), 4.51 (d, *J* = 11.5 Hz, 1H), 4.13 (dqt, *J* = 10.9, 7.4, 3.5 Hz, 2H), 3.85 – 3.75 (m, 4H), 2.99 (dd, *J* = 9.7, 4.2 Hz, 1H), 2.96 – 2.82 (m, 2H), 2.83 – 2.72 (m, 3H), 2.70 – 2.57 (m, 2H), 2.14 – 1.99 (m, 2H), 1.98 – 1.85 (m, 2H), 1.80 (d, *J* = 1.3 Hz, 3H), 1.25 (t, *J* = 7.1 Hz, 3H) ppm.

^{13}C–NMR (101 MHz, $CDCl_3$): δ = 166.2, 159.4, 159.2, 137.3, 135.5, 129.3, 129.3, 118.6 (q, J = 320.4 Hz), 116.8, 113.9, 97.0, 70.5, 59.6, 55.4, 47.1, 42.7, 37.0, 30.6, 29.2 (two carbons), 28.6, 25.9, 25.0, 14.5 ppm.

^{19}F–NMR (376 MHz, $CDCl_3$) δ = 73.8 ppm.

IR (Diamond-ATR, neat): 2935, 1708, 1515, 1417, 1247, 1209, 1171, 1140, 1069, 825 ppm.

HRMS (ESI) calc. for $C_{26}H_{33}F_3NaO_8S_3^+$ $[M+Na]^+$: 649.1182; found: 649.1175.

$[\alpha]_D^{20}$: 45.1 (c = 1.84, CH_2Cl_2)

Silyl ether 18

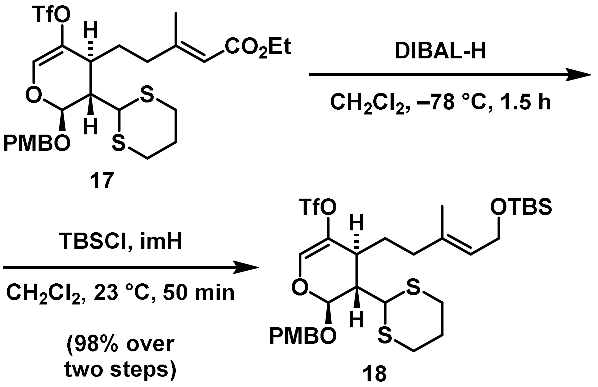

Note: The reduction was conducted in three parallel runs, which were combined in the work-up!

A solution of diisobutylaluminum hydride (1.00 M in tetrahydrofuran, 22.3 mL, 22.3 mmol, 2.30 equiv) was added slowly to a solution of ester **17** (6.07 g, 9.69 mmol, 1 equiv) in dichloromethane (100 mL) at –78 °C. After 1.5 h, the reaction mixtures of the three in parallel conducted reaction runs were poured into an aqueous saturated sodium potassium tartrate solution (500 mL) and the mixture was stirred vigorously for 1 h. The layers were separated and the aqueous phase was extracted with dichloromethane (2 × 200 mL). The combined organic phases were dried over magnesium sulfate and the dried solution was filtrated. The filtrate was concentrated to give crude alcohol **S8** which was used in the following oxidation step without further purification.

Tert-butyl dimethyl silyl chloride (6.07 g, 35.0 mmol, 1.20 equiv) was added to a solution of crude alcohol **S8** (assumed 29.3 mmol, 1 equiv) and imidazole (3.58 g, 52.6 mmol, 1.80 equiv) in dichloromethane (300 mL) at 23 °C. After 50 min, water (400 mL) was added to the reaction mixture. The layers were separated and the aqueous phase was extracted with dichloromethane (2 × 200 mL). The combined organic phases were dried over magnesium sulfate and the dried solution was filtrated. The filtrate was concentrated and the residue was purified by flash column chromatography on silica gel (10% diethyl ether in pentane) to give silyl ether **18** (20.0 g, 98% over two steps) as a colorless oil.

Alcohol **S8**

TLC (70% diethyl ether in pentane): R_f = 0.30 (UV, Permanganate)

^{1}H–NMR (400 MHz, $CDCl_3$): δ = 7.22 (d, *J* = 8.6 Hz, 2H), 6.88 (d, *J* = 8.7 Hz, 2H), 6.66 – 6.61 (m, 1H), 5.51 (d, *J* = 1.8 Hz, 1H), 5.45 (tq, *J* = 6.8, 1.3 Hz, 1H), 4.71 (s, 1H), 4.49 (d, *J* = 11.4 Hz, 1H), 4.17 – 4.10 (m, 2H), 3.87 (d, *J* = 10.2 Hz, 1H), 3.81 (s, 3H), 2.99 – 2.93 (m, 1H), 2.92 – 2.77 (m, 4H), 2.45 (dt, *J* = 10.2, 1.6 Hz, 1H), 2.17 – 1.98 (m, 4H), 1.95 – 1.84 (m, 2H), 1.62 (s, 3H), 1.26 (t, *J* = 5.5 Hz, 1H) ppm.

^{13}C–NMR (101 MHz, $CDCl_3$): δ = 159.5, 138.6, 137.6, 135.3, 129.4, 129.2, 124.8, 118.6 (q, *J* = 320.5 Hz) 114.0, 97.0, 70.6, 59.6, 55.4, 47.7, 43.2, 36.5, 36.0, 30.0, 29.9, 28.1, 25.9, 16.1.

^{19}F–NMR (376 MHz, $CDCl_3$) δ = –73.7 ppm.

IR (Diamond-ATR, neat): 3393, 2935, 1515, 1417, 1247, 1212, 1172, 1140, 1070, 825.

HRMS (ESI) calc. for $C_{24}H_{31}F_3NaO_7S_3^+$ $[M+Na]^+$: 607.1076; found: 607.1072.

$[\alpha]_D^{20}$: 49.3 (c = 0.25, CH_2Cl_2).

Silyl ether **18**

TLC (40% diethyl ether in pentane): R_f = 0.70 (UV, CAM)

^{1}H–NMR (400 MHz, $CDCl_3$): δ = 7.22 (d, *J* = 8.7 Hz, 2H), 6.87 (d, *J* = 8.7 Hz, 2H), 6.67 – 6.60 (m, 1H), 5.50 (d, *J* = 1.8 Hz, 1H), 5.38 – 5.30 (m, 1H), 4.72 (d, *J* = 11.5 Hz, 1H), 4.50 (d, *J* = 11.5 Hz, 1H), 4.17 (d, *J* = 6.2 Hz, 2H), 3.84 (d, *J* = 10.0 Hz, 1H), 3.81 (s, 3H), 2.94 (dd, *J* = 8.4, 4.5 Hz, 1H), 2.82 (tdd, *J* = 17.1, 9.0, 3.7 Hz, 4H), 2.45 (dt, *J* = 10.1, 1.7 Hz, 1H), 2.17 – 1.81 (m, 6H), 1.56 (s, 3H), 0.90 (s, 9H), 0.06 (s, 6H) ppm.

^{13}C–NMR (101 MHz, $CDCl_3$): δ = 159.4, 137.4, 136.0, 135.2, 129.3, 129.0, 125.3, 118.5 (q, *J* = 320.4 Hz), 113.9, 96.6, 70.4, 60.3, 55.3, 47.3, 43.0, 36.5, 36.3, 29.6 (two carbons), 28.3, 26.0, 25.8, 18.4, 16.1, –5.1, –5.1.

^{19}F–NMR (376 MHz, $CDCl_3$) δ = 73.8 ppm.

IR (Diamond-ATR, neat): 2931, 2857, 1733, 1515, 1420, 1249, 1213, 1141, 1073, 834 cm^{-1}.

HRMS (ESI) calc. for $C_{30}H_{45}F_3NaO_7S_3Si^+$ $[M+Na]^+$: 721.1941; found: 721.1934.

$[\alpha]_D^{20}$: 53.2 (c = 0.26, CH_2Cl_2).

2.2 Sulfone approach

Sulfone 19

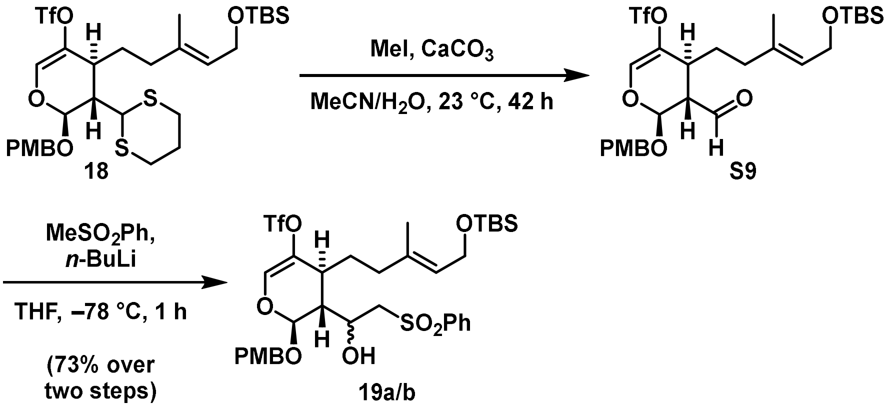

Note: The deprotection of the dithiane was conducted in three parallel runs which were combined for purification.

Methyl iodide (2.67 mL, 42.9 mmol, 25.0 equiv) and calcium carbonate (687 mg, 6.87 mmol, 4.00 equiv) were added to a solution of dithiane **18** (1.21 g, 1.72 mmol, 1 equiv) in a mixture of acetonitrile and water (4:1, 12.5 mL) at 23 °C. After 42 h, the reaction mixtures of all three parallel reaction runs were poured into a mixture of water (120 mL) and dichloromethane (120 mL). The layers were separated and the aqueous phase was extracted with dichloromethane (2 × 75 mL). The combined organic phases were dried over magnesium sulfate and the dried solution was filtrated. The filtrate was concentrated to give crude aldehyde **S9**, which was used in the following step without further purification.

A solution of *n*-butyllithium (2.50 M in hexanes, 2.88 mL, 7.21 mmol, 1.40 equiv) was added dropwise to a solution of methyl phenyl sulfone (1.24 g, 7.73 mmol, 1.50 equiv) in tetrahydrofuran (70 mL) at –78 °C. After 45 min, a solution of crude **S9** (assumed 5.15 mmol, 1 equiv) in tetrahydrofuran (15 mL) was added to the reaction mixture. After 1 h, the reaction mixture was allowed to warm to –45 °C. After 10 min, water (150 mL) and dichloromethane (150 mL) were added to the yellow reaction mixture. The layers were separated and the aqueous phase was extracted with dichloromethane (2 × 100 mL). The combined organic phases were dried over magnesium sulfate and the dried solution was filtrated. The filtrate was concentrated and the residue was purified by flash column chromatography on silica gel (25% ethyl acetate in cyclohexane) to give an inseparable, diastereomeric mixture of alcohols **19a/b** (2.89 g, 73% over two steps) as a yellowish oil.

*Analytical data for aldehyde **S9** were recorded from a small aliquot of the crude reaction mixture.*

TLC (10% ethyl acetate in cyclohexane): R_f = 0.25 (UV, CAM).

^{1}H–NMR (400 MHz, $CDCl_3$): δ = 9.55 (s, 1H), 7.22 (d, *J* = 8.6 Hz, 2H), 6.89 (d, *J* = 8.7 Hz, 2H), 6.73 – 6.68 (m, 1H), 5.35 – 5.27 (m, 2H), 4.79 (d, *J* = 11.5 Hz, 1H), 4.54 (d, *J* = 11.5 Hz, 1H), 4.16 (d, *J* = 6.2 Hz, 2H), 3.81 (s, 3H), 3.05 – 2.93 (m, 2H), 2.07 – 1.99 (m, 2H), 1.95 – 1.86 (m, 2H), 1.58 (s, 3H), 0.89 (s, 9H), 0.06 (s, 6H) ppm.

^{13}C–NMR (101 MHz, $CDCl_3$): δ = 198.1, 159.6, 137.1, 136.0, 135.4, 129.6, 128.3, 125.9, 116.89, 114.0, 95.7, 70.6, 60.1, 55.3, 51.7, 36.1, 33.5, 27.2, 26.0, 25.7, 18.4, 15.9, –5.1 ppm.

^{19}F–NMR (376 MHz, $CDCl_3$) δ = –73.3 ppm.

IR (Diamond-ATR, neat): 2928, 2856, 1420, 1248, 1211, 1141, 1070, 833, 776, 607 cm^{-1}.

HRMS (ESI) calc. for $C_{27}H_{39}F_3NaO_8SSi^+$ $[M+Na]^+$: 631.1979; found: 631.1975.

$[\alpha]_D^{20}$: 29.0 (c = 0.19, CH_2Cl_2).

19a

TLC (20% ethyl acetate in cyclohexane): R_f = 0.15 (UV, CAM).

^{1}H–NMR (400 MHz, $CDCl_3$): δ = 7.93 – 7.85 (m, 2H), 7.71 – 7.64 (m, 1H), 7.61 – 7.54 (m, 2H), 7.22 – 7.16 (m, 2H), 6.88 – 6.82 (m, 2H), 6.58 (d, *J* = 1.2 Hz, 1H), 5.30 – 5.24 (m, 2H), 4.71 (d, *J* = 11.4 Hz, 1H), 4.51 – 4.45 (m, 1H), 4.20 – 4.03 (m, 3H), 3.81 – 3.78 (m, 4H), 3.44 – 3.24 (m, 2H), 2.34 – 2.30 (m, 1H), 2.18 (dt, *J* = 6.4, 3.2 Hz, 1H), 2.11 – 1.68 (m, 4H), 1.54 (s, 3H), 0.91 (s, 9H), 0.07 (s, 6H) ppm.

^{13}C–NMR (101 MHz, $CDCl_3$): δ = 159.4, 138.7, 137.2, 136.3, 135.8, 134.2, 129.5, 129.2, 128.8, 127.9, 125.7, 118.4 (d, *J* = 320.8 Hz), 113.9, 97.1, 70.8, 65.3, 60.1, 60.1, 55.3, 44.8, 36.0, 34.5, 27.6, 26.0, 18.4, 16.0, –5.1 (two carbons) ppm.

^{19}F–NMR (376 MHz, $CDCl_3$) δ = –73.3 ppm.

19b

^{1}H–NMR (400 MHz, $CDCl_3$): δ = 7.93 – 7.85 (m, 2H), 7.71 – 7.64 (m, 1H), 7.61 – 7.54 (m, 2H), 7.22 – 7.16 (m, 2H), 6.88 – 6.82 (m, 2H), 6.52 (d, *J* = 0.9 Hz, 1H), 5.30 – 5.24 (m, 2H), 4.66 (d, *J* = 11.5 Hz, 1H), 4.51 – 4.45 (m, 1H), 4.20 – 4.03 (m, 3H), 3.81 – 3.78 (m, 4H), 3.44 – 3.24 (m, 2H), 2.50 (dd, *J* = 5.4, 1.6 Hz, 1H), 2.11 – 1.68 (m, 5H), 1.53 (s, 3H), 0.91 (s, 9H), 0.07 (s, 6H) ppm.

^{13}C–NMR (101 MHz, $CDCl_3$): δ = 159.4, 138.5, 137.7, 136.0, 135.7, 134.2, 129.5, 129.4, 128.9, 128.1, 125.7, 118.4 (d, *J* = 320.4 Hz), 113.9, 95.5, 70.3, 67.0, 59.9, 57.9, 55.3, 44.5, 36.4, 35.0, 28.3, 26.0, 18.4, 15.9, –5.1 (two carbons) ppm.

^{19}F–NMR (376 MHz, $CDCl_3$) δ = –73.8 ppm.

HRMS (ESI) calc. for $C_{34}H_{47}F_3NaO_{10}S_2Si^+$ $[M+Na]^+$: 787.2224; found: 787.2210.

Sulfone S10

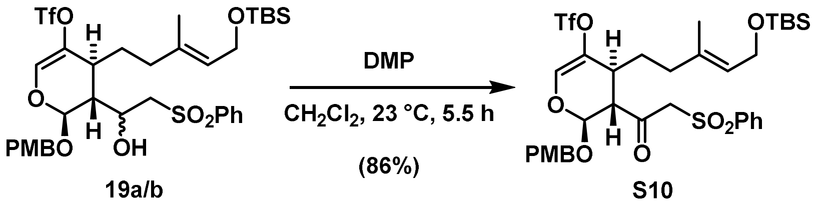

Dess–Martin periodinane (3.63 g, 8.31 mmol, 2.20 equiv) was added to a solution of alcohols **19a/b** (2.89 g, 3.78 mmol, 1 equiv) in dichloromethane (50 mL) at 23 °C. After 5.5 h, water (100 mL) was added to the reaction mixture. The layers were separated and the aqueous phase was extracted with dichloromethane (2 × 75 mL). The combined organic phases were dried over magnesium sulfate and the dried solution was filtrated. The filtrate was concentrated and the residue was purified by flash column chromatography on silica gel (35% diethyl ether in pentane) to give sulfone **S10** (2.49 g, 86%) as a colorless oil.

TLC (45% diethyl ether in pentane): R_f = 0.59 (UV, CAM).
^{1}H–NMR (400 MHz, $CDCl_3$): δ = 7.87 – 7.82 (m, 2H), 7.67 (tt, *J* = 6.9, 1.1 Hz, 1H), 7.56 (t, *J* = 7.7 Hz, 2H), 7.15 (d, *J* = 8.6 Hz, 2H), 6.87 (d, *J* = 8.7 Hz, 2H), 6.76 (d, *J* = 1.5 Hz, 1H), 5.39 – 5.33 (m, 1H), 4.87 – 4.79 (m, 2H), 4.50 (d, *J* = 6.6 Hz, 1H), 4.47 (d, *J* = 3.8 Hz, 1H), 4.21 – 4.15 (m, 3H), 3.81 (s, 3H), 3.47 (dd, *J* = 8.4, 6.9 Hz, 1H), 3.21 (dddd, *J* = 7.8, 5.5, 3.6, 1.3 Hz, 1H), 2.10 (td, *J* = 12.5, 12.0, 5.8 Hz, 1H), 1.87 (td, *J* = 13.5, 12.8, 4.0 Hz, 1H), 1.80 – 1.63 (m, 2H), 1.62 (s, 3H), 0.89 (s, 9H), 0.06 (s, 6H) ppm.
^{13}C–NMR (101 MHz, $CDCl_3$): δ = 195.4, 159.8, 138.8, 137.6, 135.8, 135.6, 134.3, 129.8, 129.4, 128.3, 127.7, 125.4,118.5 (q, *J* = 320.8 Hz), 114.1, 98.4, 71.5, 67.5, 60.2, 55.3, 53.6, 35.5, 34.2, 26.4, 26.0, 18.4, 16.2, –5.1, –5.2 ppm.
^{19}F–NMR (376 MHz, $CDCl_3$) δ = –73.1 ppm.
IR (Diamond-ATR, neat): 2955, 2929, 2856, 1422, 1250, 1214, 1142, 1069, 836, 777 cm^{-1}.
HRMS (ESI) calc. for $C_{34}H_{45}F_3NaO_{10}S_2Si^+$ $[M+Na]^+$: 785.2068; found: 785.2071.
$[\alpha]_D^{20}$: 43.0 (c = 0.16, CH_2Cl_2).

Alcohol S11

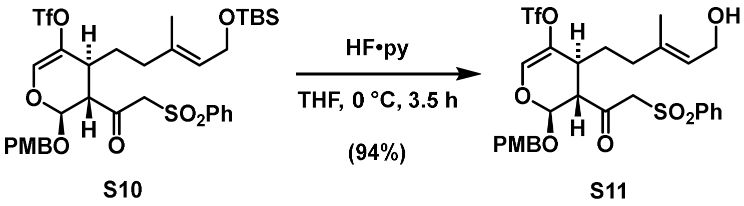

Pyridine hydrofluoride (≈70% hydrogen fluoride, ≈30% pyridine, 2.43 mL, 93.6 mmol, 30.0 equiv) was added to a solution of sulfone **S10** (2.38 g, 3.12 mmol, 1 equiv) in tetrahydrofuran (40 mL) at 0 °C. After 3.5 h, aqueous saturated sodium hydrogen carbonate solution (50 mL) and dichloromethane (50 mL) were added to the reaction mixture. The layers were separated and the aqueous phase was extracted with dichloromethane (2 × 50 mL). The combined organic phases were dried over magnesium sulfate and the dried solution was filtrated. The filtrate was concentrated and the residue was purified by flash column chromatography on silica gel (90% diethyl ether in pentane) to give alcohol **S11** (1.91 g, 94%) as a colorless oil.

TLC (60% diethyl ether in pentane): R_f = 0.14 (UV, CAM).
^{1}H–NMR (400 MHz, $CDCl_3$): δ = 7.87 – 7.81 (m, 2H), 7.68 (tt, *J* = 6.9, 1.2 Hz, 1H), 7.59 – 7.53 (m, 2H), 7.15 (d, *J* = 8.7 Hz, 2H), 6.87 (d, *J* = 8.7 Hz, 2H), 6.75 (d, *J* = 1.5 Hz, 1H), 5.52 – 5.43 (m, 1H), 4.88 (d, *J* = 6.4 Hz, 1H), 4.81 (d, *J* = 11.2 Hz, 1H), 4.49 (d, *J* = 2.4 Hz, 1H), 4.46 (s, 1H), 4.19 (d, *J* = 13.9 Hz, 1H), 4.14 (d, *J* = 6.8 Hz, 2H), 3.81 (s, 3H), 3.50 (dd, *J* = 7.8, 6.5 Hz, 1H), 3.20 (ddt, *J* = 6.4, 4.6, 2.2 Hz, 1H), 2.15 (ddd, *J* = 13.7, 10.4, 6.5 Hz, 1H), 1.94 (td, *J* = 13.9, 12.2, 4.4 Hz, 1H), 1.86 – 1.68 (m, 2H), 1.66 (s, 3H), 1.50 (bs, 1H).
^{13}C–NMR (101 MHz, $CDCl_3$): δ = 195.3, 159.8, 138.8, 138.3, 137.5, 135.6, 134.4, 129.8, 129.4, 128.2, 127.7, 124.8, 118.5 (q, *J* = 320.9 Hz), 114.1, 98.1, 71.4, 67.3, 59.3, 55.3, 53.6, 35.2, 34.3, 26.3, 16.0ppm.
^{19}F–NMR (376 MHz, $CDCl_3$) δ = –73.0 ppm.
IR (Diamond–ATR, neat): 3460, 2933, 1515, 1419, 1248, 1214, 1141, 1121, 1082, 831 cm^{-1}.
HRMS (ESI) calc. for $C_{28}H_{32}F_3NaO_{10}S_2^+$ $[M+Na]^+$: 649.1383; found: 649.1372.
$[\alpha]_D^{20}$: 51.3 (c = 0.37, CH_2Cl_2).

Bromide 8

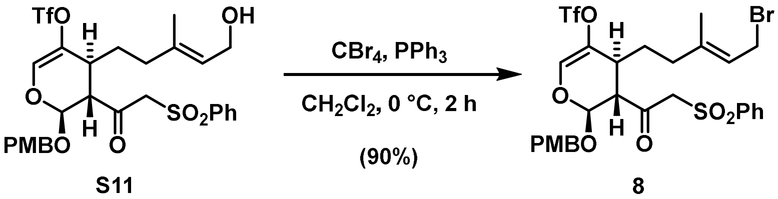

Carbon tetrabromide (1.23 g, 3.68 mmol, 1.25 equiv) was added to a solution of alcohol **S11** (1.91 g, 2.94 mmol, 1 equiv) and triphenylphosphine (975 mg, 3.68 mmol, 1.25 equiv) in dichloromethane (30 mL) at 0 °C. After 2 h, water (30 mL) was added to the reaction mixture. The layers were separated and the aqueous phase was extracted with dichloromethane (2 × 30 mL). The combined organic phases were dried over magnesium sulfate and the dried solution was filtrated. The filtrate was concentrated and the residue was purified by flash column chromatography on silica gel (50% diethyl ether in pentane) to give bromide **8** (1.88 g, 90%) as a pale yellow oil.

TLC (45% diethyl ether in pentane): R_f = 0.45 (UV, CAM).
^{1}H–NMR (400 MHz, $CDCl_3$): δ = 7.90 – 7.79 (m, 2H), 7.69 (t, *J* = 7.4 Hz, 1H), 7.57 (t, *J* = 7.6 Hz, 2H), 7.15 (d, *J* = 8.6 Hz, 2H), 6.87 (d, *J* = 8.6 Hz, 2H), 6.77 (d, *J* = 1.2 Hz, 1H), 5.69 – 5.53 (m, 1H), 4.92 – 4.78 (m, 2H), 4.53 – 4.44 (m, 2H), 4.14 (d, *J* = 13.7 Hz, 1H), 4.00 (d, *J* = 8.4 Hz, 2H), 3.81 (s, 3H), 3.57 – 3.45 (m, 1H), 3.34 – 3.18 (m, 1H), 2.20 (ddd, *J* = 13.5, 11.2, 6.0 Hz, 1H), 1.93 (td, *J* = 13.6, 12.7, 4.4 Hz, 1H), 1.85 – 1.58 (m, 5H) ppm.
^{13}C–NMR (101 MHz, $CDCl_3$): δ = 195.2, 159.8, 142.3, 138.7, 137.7, 135.4, 134.4, 129.8, 129.5, 128.2, 127.6, 121.6, 118.5 (q, *J* = 320.8 Hz), 114.1, 98.4, 71.5, 67.6, 55.3, 53.6, 35.3, 34.1, 29.2, 26.2, 15.7 ppm.
^{19}F–NMR (376 MHz, $CDCl_3$) δ = –73.0 ppm.
IR (Diamond–ATR, neat): 2929, 1515, 1419, 1325, 1248, 1213, 1141, 1074, 865, 831 cm^{-1}.
HRMS (ESI) calc. for $C_{28}H_{30}BrF_3NaO_9S_2^+$ $[M+Na]^+$: 733.0359; found: 733.0347.
$[\alpha]_D^{20}$: 42.1 (c = 0.17, CH_2Cl_2).

Sulfone 20

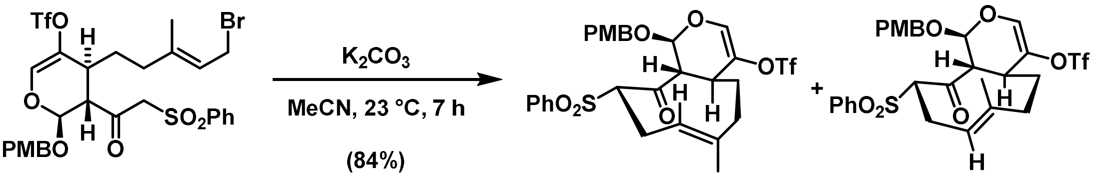

Potassium carbonate (922 mg, 6.61 mmol, 2.50 equiv) was added to a solution of bromide **8** (1.88 g, 2.64 mmol, 1 equiv) in acetonitrile (144 mL) at 23 °C. After 7 h, aqueous saturated ammonium chloride solution (150 mL) and dichloromethane (150 mL) were added to the reaction mixture. The layers were separated and the aqueous phase was extracted with dichloromethane (2 × 100 mL). The combined organic phases were dried over magnesium sulfate and the dried solution was filtrated. The filtrate was concentrated and the residue was purified by flash column chromatography on silica gel (20% diethyl ether in pentane) to give the cyclized sulfone as an inseparable mixture of confomartional diastereomers **20** and **20'** (ratio = 2:1, 1.40 g, 84%) as a colorless solid.
Crystals of **20** that were suitable for single crystal X-ray analysis were obtained by vapour diffusion (diethyl ether/ pentane).

TLC (30% ethyl acetate in cyclohexane): R_f = 0.44 (UV, CAM).

20

^{1}H–NMR (700 MHz, $CDCl_3$): δ = 7.74 – 7.71 (m, 2H), 7.63 – 7.60 (m, 1H), 7.47 – 7.45 (m, 2H), 7.42 (d, J = 8.6 Hz, 2H), 6.93 – 6.91 (m, 2H)*, 6.71 (s, 1H), 5.40 – 5.36 (m, 1H), 4.79 (d, J = 10.3 Hz, 1H), 4.69 – 4.65 (m, 2H), 4.55 (dd, J = 11.2, 7.9 Hz, 1H), 3.83 (s, 3H)*, 3.11 – 3.05 (m, 2H), 2.63 – 2.60 (m, 1H), 2.60 – 2.52 (m, 1H)*, 2.44 (ddd, J = 12.7, 7.9, 4.6 Hz, 1H), 2.18 – 2.13 (m, 1H), 1.70 – 1.59 (m, 2H), 1.37 (s, 3H) ppm.

* marks signals in which protons from both conformational isomers overlap.

^{13}C–NMR (176 MHz, $CDCl_3$): δ = 199.0, 159.8, 140.7, 138.4, 138.3, 137.4, 133.9, 131.0, 129.7, 129.0, 128.7, 119.5, 118.7 (q, *J* = 320.9 Hz), 113.9, 100.8, 74.1, 72.2, 64.1, 55.5, 37.2, 34.1, 33.8, 28.3, 21.7 ppm.

^{19}F–NMR (376 MHz, $CDCl_3$) δ = –73.1 ppm.

20'

^{1}H–NMR (700 MHz, $CDCl_3$): δ = 7.79 – 7.77 (m, 2H), 7.66 – 7.63 (m, 1H), 7.51 – 7.48 (m, 2H), 7.40 (d, J = 8.6 Hz, 2H), 6.93 – 6.91 (m, 2H)*, 6.73 (s, 1H), 5.05 – 5.02 (m, 1H), 4.74 (d, J = 10.3 Hz, 1H), 4.71 (d, J = 8.0 Hz, 1H), 4.64 (d, J = 10.3 Hz, 1H), 4.60 (t, J = 9.4 Hz, 1H), 3.83 (s, 3H)*, 2.85 – 2.77 (m, 2H), 2.60 – 2.52 (m, 2H)*, 2.23 – 2.19 (m, 1H), 2.08 – 2.01 (m, 2H), 1.73 (s, 3H), 1.57 – 1.45 (m, 1H) ppm.

* marks signals in which protons from both conformational isomers overlap.

^{13}C–NMR (176 MHz, $CDCl_3$): δ = 202.1, 159.8, 141.3, 140.3, 138.0, 137.3, 134.1, 130.9, 129.9, 129.1, 128.8, 119.4, 118.7 (q, *J* = 321.0 Hz), 114.0, 101.0, 72.2, 71.4, 62.6, 55.5, 39.4, 38.9, 32.5, 24.6, 16.6 ppm.

^{19}F–NMR (376 MHz, $CDCl_3$) δ = –73.2 ppm.

IR (Diamond–ATR, neat): 2925, 2853, 1718, 1613, 1420, 1309, 1248, 1212, 1140, 862 cm^{-1}.

HRMS (ESI) calc. for $C_{28}H_{29}F_3NaO_9S_2^+$ $[M+Na]^+$: 653.1097; found: 653.1087.
$[\alpha]_D^{20}$: 51.1 (c = 0.21, CH_2Cl_2).

Ketone 21

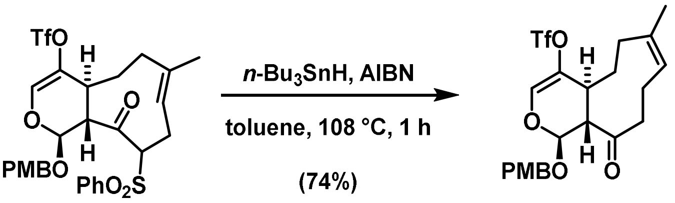

20 21

Azobisisobutyronitrile (365 mg, 2.22 mmmol, 1.00 equiv) was added to a solution of sulfones **20** and **20'**(1.40 g, 2.22 mmol, 1 equiv) and tributyltin hydride (2.47 mL, 8.88 mmol, 4.00 equiv) in toluene (30 mL) and the resulting solution was heated to 108 °C. After 1 h, the reaction mixture was cooled to room temperature, evaporated and the residue was purified by flash column chromatography on silica gel (2% diethyl ether in pentane until tin impurities were removed, then 7% diethyl ether in pentane) to give ketone **21** (800 mg, 74%) as a colorless oil.

TLC (25% diethyl ether in pentane): R_f = 0.59 (UV, CAM).
^{1}H–NMR (400 MHz, $CDCl_3$): δ = 7.16 (d, *J* = 8.6 Hz, 2H), 6.87 (d, *J* = 8.7 Hz, 2H), 6.70 (s, 1H), 5.44 (t, *J* = 7.4 Hz, 1H), 4.79 (d, *J* = 11.3 Hz, 1H), 4.73 (d, *J* = 7.7 Hz, 1H), 4.47 (d, *J* = 11.3 Hz, 1H), 3.81 (s, 3H), 3.23 – 3.08 (m, 2H), 2.61 – 2.47 (m, 1H), 2.39 – 2.23 (m, 3H), 2.12 – 1.99 (m, 3H), 1.68 (s, 3H), 1.48 – 1.37 (m, 1H) ppm.
^{13}C–NMR (101 MHz, $CDCl_3$) δ = 212.4, 159.8, 138.3, 138.0, 136.8, 129.8, 128.3, 124.0, 118.7 (q, *J* = 320.6 Hz), 114.1, 100.3, 71.5, 55.4, 54.6, 43.2, 36.8, 27.8, 27.1, 24.9, 22.5 ppm.
^{19}F–NMR (376 MHz, $CDCl_3$) δ = –73.2 ppm.
IR (Diamond–ATR, neat): 2938, 1708, 1515, 1419, 1247, 1210, 1139, 1120, 1092, 861 cm^{-1}.
HRMS (ESI) calc. for $C_{22}H_{25}F_3NaO_7S^+$ $[M+Na]^+$: 513.1165; found: 513.1165.
$[\alpha]_D^{20}$: 114.3 (c = 0.37, CH_2Cl_2).

Alkene S12

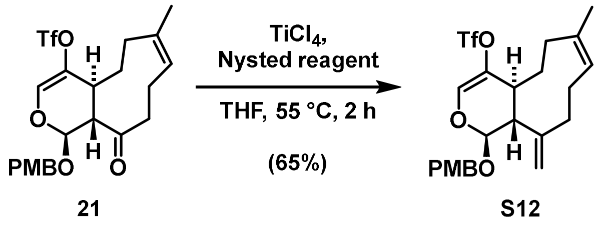

Titanium tetrachloride (1.44 mL, 13.0 mmol, 8.00 equiv) was added dropwise to the commercially available suspension of Nysted reagent (20% suspension in tetrahydrofuran, 56.2 mL, 16.2 mmol, 10.0 equiv) in tetrahydrofuran (20 mL) at 0 °C. After 20 min, a solution of ketone **21** (800 mg, 1.63 mmol, 1 equiv) in tetrahydrofuran (5 mL) was added and the reaction mixture was heated to 55 °C. After 2 h, the reaction mixture was cooled to 0 °C and aqueous saturated ammonium chloride solution (150 mL) and dichloromethane (150 mL) were added. The layers were separated and the aqueous phase was extracted with dichloromethane (2 × 50 mL). The combined organic phases were dried over magnesium sulfate and the dried solution was filtrated. The filtrate was concentrated and the residue was purified by flash column chromatography on silica gel (10% diethyl ether in pentane) to give alkene **S12** (519 mg, 65%) as a colorless oil.

TLC (2% diethyl ether in pentane): R_f = 0.37 (UV, CAM).
^{1}H–NMR (400 MHz, $CDCl_3$): δ = 7.21 (d, *J* = 8.7 Hz, 2H), 6.87 (d, *J* = 8.7 Hz, 2H), 6.71 (d, *J* = 1.7 Hz, 1H), 5.38 (t, *J* = 7.8 Hz, 1H), 4.98 (s, 1H), 4.93 (s, 1H), 4.74 (d, *J* = 11.6 Hz, 1H), 4.69 (d, *J* = 5.6 Hz, 1H), 4.47 (d, *J* = 11.6 Hz, 1H), 3.81 (s, 3H), 2.73 (t, *J* = 5.7 Hz, 1H), 2.65 – 2.44 (m, 2H), 2.41 – 2.26 (m, 2H), 2.22 – 2.11 (m, 1H), 2.10 – 1.96 (m, 3H), 1.65 (s, 3H), 1.62 – 1.51 (m, 1H) ppm.
^{13}C–NMR (101 MHz, $CDCl_3$) δ = 159.5, 150.3, 138.6, 136.3, 136.3, 129.5, 129.2, 126.0, 118.7 (q, *J* = 320.6 Hz), 114.7, 113.9, 100.5, 70.4, 55.4, 45.9, 38.3, 35.5, 28.1, 27.8, 26.9, 22.8 ppm.
^{19}F–NMR (376 MHz, $CDCl_3$) δ = –73.4 ppm.
IR (Diamond–ATR, neat): 2931, 2861, 1541, 1614, 1419, 1248, 1211, 1142, 1068, 832 cm^{-1}.
HRMS (ESI) calc. for $C_{23}H_{27}F_3NaO_6S^+$ $[M+Na]^+$: 511.1373; found: 511.1376.
$[\alpha]_D^{20}$: 127.2 (c = 0.13, CH_2Cl_2).

Aldehyde S13

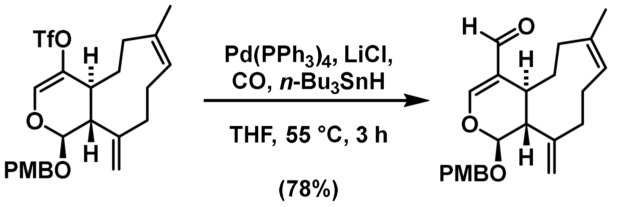

S12 S13

Note: Tetrahydrofuran was degassed via freeze-pump-thaw (three cycles) prior to use. Tetrakis(triphenylphosphine)palladium(0) (122 mg, 104 µmol, 20.0 mol%), and flame dried lithium chloride (224 mg, 5.22 mmol, 10.0 equiv) were added to a solution of alkene **S12** (255 mg, 522 µmol, 1 equiv) in tetrahydrofuran (10 mL). The flask was fitted with a balloon filled with carbon monoxide gas, and the solution was sparged with carbon monoxide for 10 minutes. The yellow reaction mixture was then heated to 55 °C under a carbon monoxide atmosphere. A solution of tributyltin hydride (203 µL, 731 µmol, 1.40 equiv) in tetrahydrofuran (0.8 mL) was added dropwise over a period of 1.5 h. After 1.5 h, the reaction mixture was cooled to 23 °C and water (30 mL) and dichloromethane (30 mL) were added. The layers were separated, and the aqueous phase was extracted with dichloromethane (2 × 30 mL). The combined organic phases were dried over magnesium sulfate and the dried solution was filtrated. The filtrate was concentrated, and the residue was purified by flash column chromatography on silica gel (2% diethyl ether in pentane until tin impurities were removed, then 30% diethyl ether in pentane) to give aldehyde **S13** (150 mg, 78%) as a colorless oil.

TLC (30% diethyl ether in pentane): R_f = 0.57 (UV, CAM).
^{1}H–NMR (400 MHz, $CDCl_3$): δ = 9.25 (s, 1H), 7.22 (d, *J* = 8.7 Hz, 2H), 7.18 (d, *J* = 1.2 Hz, 1H), 6.88 (d, *J* = 8.7 Hz, 2H), 5.35 (t, *J* = 7.9 Hz, 1H), 4.87 (s, 1H), 4.85 (d, *J* = 4.7 Hz, 1H), 4.79 (d, *J* = 11.7 Hz, 1H), 4.76 (s, 1H), 4.54 (d, *J* = 11.7 Hz, 1H), 3.81 (s, 3H), 2.77 (t, *J* = 4.8 Hz, 1H), 2.61 – 2.44 (m, 2H), 2.43 – 1.96 (m, 6H), 1.76 (s, 3H), 1.48 – 1.34 (m, 1H) ppm.
^{13}C–NMR (101 MHz, $CDCl_3$) δ = 190.6, 161.6, 159.4, 150.6, 137.0, 129.3, 129.0, 125.5, 123.9, 113.8, 113.0, 101.9, 70.4, 55.3, 43.3, 35.8, 32.6, 28.7, 27.7, 27.0, 23.3 ppm.
IR (Diamond–ATR, neat): 2931, 1374, 1632, 1514, 1249, 1173, 1150, 1084, 1036, 857 cm^{-1}.
HRMS (ESI) calc. for $C_{23}H_{29}O_4^+$ [M+H]$^+$: 369.2060; found: 369.2053.
$[\alpha]_D^{20}$: 198.6 (c = 0.35, CH_2Cl_2).

Acetates 22 and S15

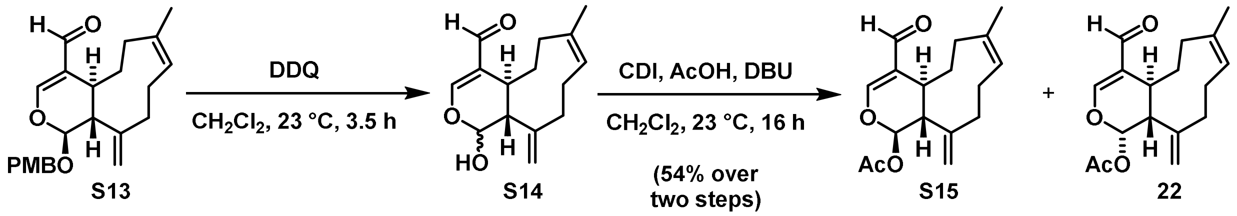

Water (0.3 mL) and 2,3-dichloro-5,6-dicyano-1,4-benzoquinone (102 mg, 434 µmol, 1.60 equiv) were sequentially added to a solution of aldehyde **S13** (100 mg, 271 µmol, 1 equiv) in dichloromethane (3.5 mL) at 0 °C. After 3.5 h, saturated aqueous sodium hydrogencarbonate solution (30 mL) and dichloromethane (25 mL) were added to the reaction mixture. The layers were separated and the aqueous phase was extracted with dichloromethane (2 × 25 mL). The combined organic phases were dried over magnesium sulfate and the dried solution was filtrated. The filtrate was concentrated and the residue was purified by flash column chromatography on silica gel (35% diethyl ether in pentane) to give alcohol **S14** (73.1 mg) as a mixture of inseparable diastereomers together with small amounts of inseparable impurities. The obtained impure product was used in the following step without further purification.

Acetic acid (329 µL, 5.74 mmol, 19.5 mmol) and 1,8-diazabicyclo[5.4.0]undec-7-ene (8.98 µL, 58.9 µmol, 0.200 equiv) were added sequentially to a solution of CDI (902 mg, 5.40 mmol, 18.3 equiv) in dichloromethane (4 mL) at 23 °C. After 20 min, a solution of alcohol **S14** (assumed clean: 73.1 mg, 294 µmol, 1 equiv) in dichloromethane (1.5 mL) was added at 23 °C. After 16 h, water (20 mL) and dichloromethane (10 mL) were added to the reaction mixture. The layers were separated, and the aqueous phase was extracted with dichloromethane (2 × 10 mL). The combined organic phases were dried over magnesium sulfate and the dried solution was filtrated. The filtrate was concentrated, and the residue was purified by flash column chromatography on silica gel (30% diethyl ether in pentane) to give an inseparable mixture of acetates **S15** and **22** (42.2 mg, 54% over two steps) as a colorless oil. Separation of **S15** and **22** was achieved by normal-phase semi-preparative HPLC purification using 8% ethyl acetate in *n*-hexane as eluent initially, with a gradient to 15% ethyl acetate in *n*-hexane as eluent over 30 min (flow rate: 20 mL/min; column: Microsorb 60-8 Si Dynamax 250 × 21.4mm (R00083121C); detection: 254 nm; retention time: **S15** 15.9 min; **22** 17.4 min).

Crystals of **22** that were suitable for single crystal X-ray analysis were obtained by vapour diffusion (diethyl ether/ pentane).

S15

TLC (40% diethyl ether in pentane): R_f = 0.43 (UV, CAM).

^{1}H–NMR (400 MHz, $CDCl_3$): δ = 9.28 (s, 1H), 7.18 (d, *J* = 1.4 Hz, 1H), 6.10 (d, *J* = 4.1 Hz, 1H), 5.43 – 5.36 (m, 1H), 4.91 – 4.89 (m, 1H), 4.78 (s, 1H), 2.81 (t, *J* = 4.1 Hz, 1H), 2.66 – 2.16 (m, 7H), 2.10 (s, 3H), 2.09 – 2.00 (m, 1H), 1.79 (s, 3H), 1.46 – 1.37 (m, 1H) ppm.

^{13}C–NMR (101 MHz, $CDCl_3$): δ = 190.5, 169.1, 160.7, 149.3, 136.5, 126.2, 123.3, 113.7, 93.4, 41.1, 35.8, 31.7, 28.9, 27.6, 26.7, 23.4, 21.0 ppm.

IR (Diamond–ATR, neat): 2932, 2863, 2726, 1758, 1677, 1637, 1217, 1164, 1014, 913 cm^{-1}.

HRMS (ESI) calc. for $C_{17}H_{22}NaO_4^+$ $[M+Na]^+$: 313.1410; found: 313.1407.
$[\alpha]_D^{20}$: 231.9 (c = 0.56, CH_2Cl_2).

22
TLC (40% diethyl ether in pentane): R_f = 0.43 (UV, CAM).
^{1}H–NMR (700 MHz, C_6D_6, 70 °C): δ = 9.00 (s, 1H), 6.49 (s, 1H), 6.16 (s, 1H), 5.48 – 5.43 (m, 1H), 4.83 (s, 1H), 4.83 (s, 1H), 2.86 (t, *J* = 10.2 Hz, 1H), 2.75 – 2.68 (m, 1H), 2.50 – 2.42 (m, 1H), 2.40 (d, *J* = 10.0 Hz, 1H), 2.14 – 2.09 (m, 2H), 2.07 – 2.00 (m, 1H), 1.98 – 1.93 (m, 1H), 1.92 (s, 3H), 1.83 – 1.78 (m, 1H), 1.53 (s, 3H), 1.10 – 1.03 (m, 1H) ppm.
^{13}C–NMR (176 MHz, C_6D_6, 70 °C): δ = 189.0, 168.3, 160.7, 151.0, 138.3, 125.6, 124.7, 117.3, 93.3, 49.3, 33.6, 30.5, 30.1 (two overlapping carbons), 27.6, 22.7, 20.1 ppm.
IR (Diamond–ATR, neat): 2964, 2925, 2862, 1764, 1678, 1630, 1212, 1184, 1164, 939 cm^{-1}.
HRMS (ESI) calc. for $C_{17}H_{22}NaO_4^+$ $[M+Na]^+$: 313.1410; found: 313.1406.
$[\alpha]_D^{20}$: –46.8 (c = 0.49, CH_2Cl_2).

Ketone 23

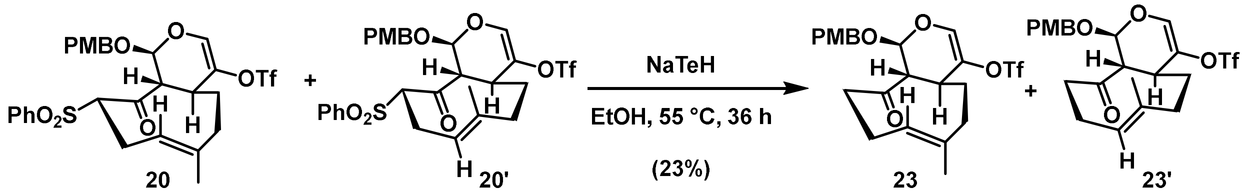

Sodium borohydride (3.7 mg, 96 µmol, 6.4 equiv) was added to a suspension of tellurium powder (9.3 mg, 72 µmol, 4.8 equiv) in ethanol (0.2 mL) and the resulting mixture was heated to 55 °C. After 2 h, a solution of sulfones **20** and **20’** (9.5 mg, 15 µmol, 1 equiv) in ethanol (0.15 mL) was added to the violet reaction mixture and stirring was continued at 55 °C. After 36 h, the reaction mixture was cooled to 23 °C and water (10 mL) and dichloromethane (10 mL) were added. The layers were separated and the aqueous phase was extracted with dichloromethane (2 × 10 mL). The combined organic phases were dried over magnesium sulfate and the dried solution was filtrated. The filtrate was concentrated and the residue was purified by flash column chromatography on silica gel (5% diethyl ether in pentane) to give conformational isomers **23** and **23’** (ratio = 1:1.2, 1.7 mg, 23%) as a colorless oil.

TLC (20% diethyl ether in pentane): R_f = 0.49+0.54 (two spots on TLC; UV, CAM).

23

^{1}H–NMR (700 MHz, $CDCl_3$): δ = 7.14 (d, *J* = 8.7 Hz, 2H), 6.87 – 6.85 (m, 2H)*, 6.69 – 6.69 (m, 1H), 5.58 (ddt, *J* = 12.1, 3.7, 1.6 Hz, 1H), 4.79 – 4.73 (m, 1H)*, 4.60 (d, *J* = 8.8 Hz, 1H), 4.46 – 4.43 (m, 1H)*, 3.80 (s, 3H), 3.09 (t, *J* = 8.4 Hz, 1H), 2.87 (ddd, *J* = 12.2, 11.1, 7.2 Hz, 1H), 2.83 – 2.74 (m, 1H)*, 2.59 (t, *J* = 7.9 Hz, 1H) 2.57 – 2.51 (m, 1H)*, 2.35 (dd, *J* = 11.1, 6.9 Hz, 1H), 2.23 – 2.16 (m, 2H)*, 1.71 – 1.62 (m, 2H), 1.52 (s, 3H) ppm.

* marks signals in which protons from both conformational diastereomers overlap.

^{13}C–NMR (176 MHz, $CDCl_3$): δ = 209.5, 159.7, 138.5, 137.5, 136.4, 129.7, 128.4, 124.7, 118.7 (d, *J* = 321.0 Hz), 114.0, 101.8, 72.0, 63.2, 55.4, 44.3, 37.8, 34.5, 34.3, 25.7, 21.6, ppm.

^{19}F–NMR (376 MHz, $CDCl_3$) δ = –73.2 ppm.

23’

^{1}H–NMR (700 MHz, $CDCl_3$): δ = 7.15 (d, J = 8.7 Hz, 2H), 6.87 – 6.85 (m, 2H)*, 6.72 (s, 1H), 5.32 – 5.27 (m, 1H), 4.79 – 4.73 (m, 2H)*, 4.46 – 4.43 (m, 1H)*, 3.81 (s, 3H), 2.83 – 2.74 (m, 2H)*, 2.72 – 2.69 (m, 1H), 2.57 – 2.51 (m, 1H)*, 2.27 (ddd, J = 11.6, 8.2, 3.5 Hz, 1H), 2.23 – 2.16 (m, 2H)*, 2.08 (dtd, J = 14.6, 3.9, 1.5 Hz, 1H), 2.01 (td, J = 12.9, 3.9 Hz, 1H), 1.74 (d, J = 1.4 Hz, 3H), 1.54 – 1.50 (m, 1H)* ppm.

* marks signals in which protons from both conformational diastereomers overlap.

^{13}C–NMR (176 MHz, $CDCl_3$): δ = 210.7, 159.8, 139.3, 137.4, 137.1, 129.8, 128.4, 125.2, 118.7 (d, *J* = 320.7 Hz), 114.1, 101.1, 71.7, 61.3, 55.4, 40.4, 39.5, 38.9, 31.5, 21.7, 16.5 ppm.

^{19}F–NMR (376 MHz, $CDCl_3$) δ = –73.1 ppm.

IR (Diamond–ATR, neat): 2936, 1710, 1613, 1515, 1421, 1248, 1212, 1141, 862, 832 cm^{-1}.

HRMS (ESI) calc. for $C_{22}H_{25}F_3NaO_7S^+$ $[M+Na]^+$: 513.1165; found: 513.1152.

$[\alpha]_D^{20}$: 65.9 (c = 0.39, CH_2Cl_2).

2.3 Total synthesis of waixenicin A, 9-deacetoxy-14,15-deepoxyxeniculin and xeniafaraunol A

Acetate S17

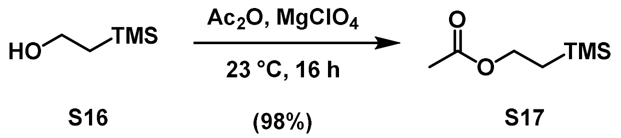

Magnesium perchlorate (247 mg, 1.11 mmol, 10.0 mol%), was added to a mixture of 2-(trimethylsilyl)ethanol **S16** (16.5 mL, 111 mmol, 1 equiv) and acetic anhydride (11.0 mL, 116 mmol, 1.05 equiv) at 23 °C. After 16 h, the reaction mixture was poured into a mixture of saturated aqueous sodium hydrogen carbonate solution (300 mL) and diethyl ether (200 mL). The layers were separated, and the aqueous phase was extracted with diethyl ether (2 × 150 mL). The combined organic phases were dried over magnesium sulfate and the dried solution was filtrated. The filtrate was concentrated to give acetate **S17** (17.3 g, 98%) as a colorless liquid.

The obtained analytical data were in full agreement with those reported in the literature.[4]

^{1}H–NMR (400 MHz, $CDCl_3$): δ = 4.18 – 4.12 (m, 2H), 2.03 (s, 3H), 1.00 – 0.95 (m, 2H), 0.04 (s, 9H) ppm.

Alcohols 24a and 24b

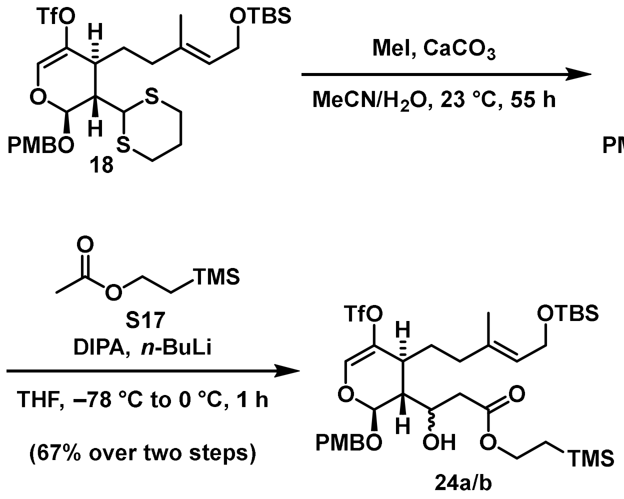

Note: The deprotection of the dithiane was conducted in three parallel batches, which were combined for purification.

Methyl iodide (11.1 mL, 179 mmol, 25.0 equiv) and calcium carbonate (2.86 g, 28.6 mmol, 4.00 equiv) were added to a solution of dithiane **18** (5.00 g, 7.15 mmol, 1 equiv) in a mixture of acetonitrile and water (4:1, 50 mL) at 23 °C. After 55 h, the reaction mixtures of all three parallel reaction runs were poured into a mixture of water (300 mL) and dichloromethane (250 mL). The layers were separated and the aqueous phase was extracted with dichloromethane (2 × 250 mL). The combined organic phases were dried over magnesium sulfate and the dried solution was filtrated. The filtrate was concentrated to give crude aldehyde **S9**, which was used in the following step without further purification.

A solution of *n*-butyllithium (1.60 M in hexanes, 32.3 mL, 51.6 mmol, 2.40 equiv) was added dropwise to a solution of diisopropylamine (7.60 mL, 53.8 mmol, 2.50 equiv) in tetrahydrofuran (300 mL) at –78 °C. After 20 min, the reaction was warmed to 0 °C. After 5 min, the reaction was cooled to –78 °C and acetate **S17** (8.62 g, 53.8 mmol, 2.50 equiv) was added. After 1 h, a solution of crude **S9** (assumed 21.5 mmol, 1 equiv) in tetrahydrofuran (30 mL) was added to the reaction mixture. After 45 min, the reaction mixture was allowed to warm to 0 °C. After 10 min, water (600 mL) and dichloromethane (600 mL) were added to the yellow reaction mixture. The layers were separated and the aqueous phase was extracted with dichloromethane (2 × 400 mL). The combined organic phases were dried over magnesium sulfate and the dried solution was filtrated. The filtrate was concentrated and the residue was purified by flash column chromatography on silica gel (30% diethyl ether in pentane) to give a diastereomeric mixture of alcohols **24a/b** (11.1 g, 67% over two steps) as a yellowish oil.

24a

TLC (40% diethyl ether in pentane): R_f = 0.37 (UV, CAM).

^{1}H–NMR (400 MHz, $CDCl_3$): δ = 7.21 (d, *J* = 8.7 Hz, 2H), 6.87 (d, *J* = 8.7 Hz, 2H), 6.63 – 6.60 (m, 1H), 5.36 – 5.30 (m, 1H), 5.13 (d, *J* = 1.8 Hz, 1H), 4.71 (d, *J* = 11.5 Hz, 1H), 4.48 (d, *J* = 11.5 Hz, 1H), 4.24 – 4.13 (m, 4H), 3.98 – 3.90 (m, 1H), 3.81 (s, 3H), 3.40 (d, *J* = 4.5 Hz, 1H), 2.56 (dd, *J* = 16.5, 2.8 Hz, 1H), 2.50 – 2.41 (m, 2H), 2.36 – 2.31 (m, 1H), 2.14 – 1.80 (m, 4H), 1.58 (s, 3H), 1.02 – 0.96 (m, 2H),

0.89 (s, 9H), 0.06 (s, 6H), 0.04 (s, 9H) ppm. *Quartet of the CF_3 carbon cannot be observed due to insufficient amount of material.*

^{13}C–NMR (101 MHz, $CDCl_3$): δ = 173.1, 159.5, 138.4, 136.2, 135.6, 129.4, 129.1, 125.6, 118.6 (q, *J* = 320.8 Hz), 114.0, 96.6, 70.2, 68.0, 63.5, 60.4, 55.4, 44.9, 37.7, 36.6, 34.9, 28.4, 26.2, 18.6, 17.5, 16.1, –1.4, –5.0 ppm.

^{19}F–NMR (376 MHz, $CDCl_3$) δ = –73.7 ppm.

IR (Diamond-ATR, neat): 3483, 2955, 2929, 1726, 1515, 1419, 1249, 1213, 1069, 835 ppm.

HRMS (ESI) calc. for $C_{34}H_{55}F_3NaO_{10}SSi_2^+$ $[M+Na]^+$: 791.2899; found: 791.2890.

$[\alpha]_D^{20}$: 30.0 (c = 0.14, CH_2Cl_2).

24b

TLC (40% diethyl ether in pentane): R_f = 0.33 (UV, CAM).

^{1}H–NMR (400 MHz, $CDCl_3$): δ = 7.22 (d, *J* = 8.7 Hz, 2H), 6.87 (d, *J* = 8.7 Hz, 2H), 6.65 (s, 1H), 5.42 (d, *J* = 2.0 Hz, 1H), 5.32 (ddt, *J* = 6.5, 3.8, 1.2 Hz, 1H), 4.73 (d, *J* = 11.5 Hz, 1H), 4.50 (d, *J* = 11.5 Hz, 1H), 4.24 – 4.14 (m, 4H), 3.94 – 3.86 (m, 1H), 3.80 (s, 3H), 3.48 (d, *J* = 4.2 Hz, 1H), 2.61 (dd, *J* = 16.8, 2.6 Hz, 1H), 2.46 (dd, *J* = 16.8, 9.3 Hz, 1H), 2.29 – 2.18 (m, 2H), 2.13 – 1.91 (m, 3H), 1.89 – 1.81 (m, 1H), 1.56 (s, 3H), 1.03 – 0.97 (m, 2H), 0.90 (s, 9H), 0.06 (s, 6H), 0.04 (s, 9H) ppm.

^{13}C–NMR (101 MHz, $CDCl_3$): δ = 173.3, 159.4, 137.6, 136.1, 136.1, 129.4, 129.3, 125.7, 118.6 (q, *J* = 320.5 Hz), 114.0, 97.2, 70.6, 67.2, 63.6, 60.3, 55.4, 44.4, 38.7, 36.6, 34.9, 28.1, 26.2, 18.6, 17.5, 16.1, –1.4, –5.0 ppm.

^{19}F–NMR (376 MHz, $CDCl_3$) δ = –73.5 ppm.

IR (Diamond-ATR, neat): 3487, 2955, 2929, 1719, 1515, 1419, 1249, 1212, 1017, 834 ppm.

HRMS (ESI) calc. for $C_{34}H_{55}F_3NaO_{10}SSi_2^+$ $[M+Na]^+$: 791.2899; found: 791.2892.

$[\alpha]_D^{20}$: 53.4 (c = 0.51, CH_2Cl_2).

Alcohol S19

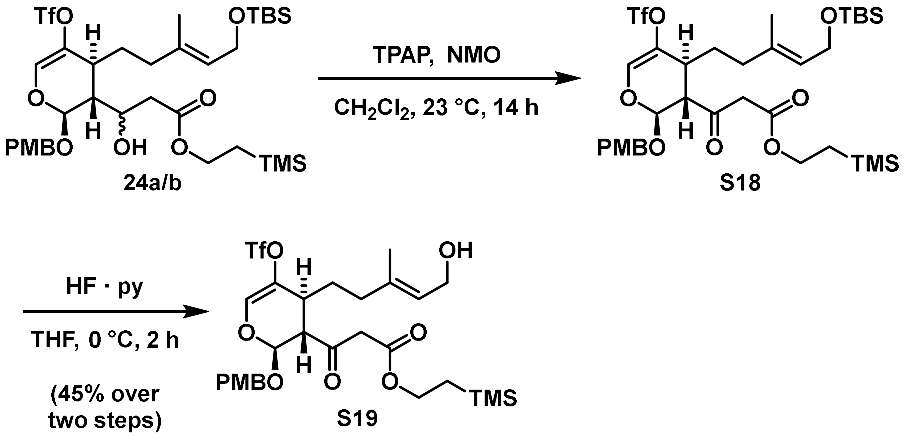

Note: The Ley oxidation was conducted in three parallel batches, which were combined after filtration over silica gel.

Tetrapropylammonium perruthenate (436 mg, 1.20 mmol, 0.250 equiv) was added to a solution of alcohol **24a/b** (3.70 g, 4.81 mmol, 1 equiv) and *N*-methylmorpholine *N*-oxide (1.74 g, 14.4 mmol, 3.00 equiv) in dichloromethane (45 mL) at 0 °C. The reaction mixture was allowed to slowly warm to 23 °C. After 14 h, the reaction mixture was filtered through a plug of silica gel. After flushing with diethyl ether (100 mL), the filtrates of all three in parallel conducted reaction runs were combined and concentrated to give crude ketoester **S18** (7.82 g) as a mixture of keto-enol tautomers, which was used in the following step without further purification.

Pyridine hydrofluoride (≈70% hydrogen fluoride, ≈30% pyridine, 7.93 mL, 305 mmol, 30.0 equiv) was added to a solution of crude ketoester **S18** (assumed pure 7.82 g, 10.2 mmol, 1 equiv) in a mixture of tetrahydrofuran (100 mL) and pyridine (8.31 mL, 102 mmol, 10.0 equiv) at 0 °C. After 2 h, aqueous saturated sodium hydrogen carbonate solution (200 mL) and dichloromethane (150 mL) were added to the reaction mixture. The layers were separated and the aqueous phase was extracted with dichloromethane (2 × 100 mL). The combined organic phases were dried over magnesium sulfate and the dried solution was filtrated. The filtrate was concentrated and the residue was purified by flash column chromatography on silica gel (70% diethyl ether in pentane) to give alcohol **S19** (4.28 g, 45% over two steps) as a colorless oil.

An analytically pure sample of **S18** was obtained by purification of a small aliquot of crude **S18** by flash column chromatography on silica gel (15% diethyl ether in pentane).

S18 *was obtained as a mixture of keto/enol-tautomers; only signals of the major keto form are reported.*

TLC (40% diethyl ether in pentane): R_f = 0.73 (UV, CAM).

^{1}H–NMR (400 MHz, $CDCl_3$): δ = 7.23 – 7.18 (m, 2H)*, 6.90 – 6.84 (m, 2H)*, 6.76 – 6.74 (m, 1H)*, 5.34 – 5.27 (m, 1H)*, 4.91 (d, *J* = 6.7 Hz, 1H), 4.84 – 4.78 (m, 1H)*, 4.53 (d, *J* = 11.2 Hz, 1H), 4.22 – 4.13 (m, 4H)*, 3.81 (s, 3H), 3.57 (s, 2H), 3.26 – 3.17 (m, 2H), 2.03 – 1.83 (m, 2H)*, 1.80 – 1.63 (m, 2H)*, 1.60 (s, 3H), 1.06 – 0.95 (m, 2H)*, 0.89 (s, 9H)*, 0.05 (s, 9H)*, 0.03 (s, 6H)* ppm.

* marks signals in which protons of the keto/enol-tautomers overlap.

^{13}C–NMR (101 MHz, $CDCl_3$): δ = 200.4, 166.7, 159.8, 137.7, 135.9, 135.7, 129.9, 128.1, 125.6, 118.7 (q, *J* = 321.0 Hz), 114.1, 98.9, 71.6, 64.0, 60.3, 55.4, 53.0, 51.1, 36.2, 34.9, 26.8, 26.2, 18.6, 17.5, 16.3, –1.4, –4.9 ppm.
^{19}F–NMR (376 MHz, $CDCl_3$) δ = –73.1 ppm.
IR (Diamond-ATR, neat): 2954, 2932, 2857, 1743, 1717, 1515, 1142, 859, 836 ppm.
HRMS (ESI) calc. for $C_{34}H_{53}F_3NaO_{10}SSi_2^+$ $[M+Na]^+$: 789.2742; found: 789.2719.
$[\alpha]_D^{20}$: 17.4 (c = 0.29, CH_2Cl_2).

S19 *was obtained as a mixture of keto/enol-tautomers; only signals of the major keto form are reported.*
TLC (70% diethyl ether in pentane): R_f = 0.36 (UV, CAM).
^{1}H–NMR (400 MHz, $CDCl_3$): δ = 7.23 – 7.18 (m, 2H)*, 6.90 – 6.85 (m, 2H)*, 6.76 – 6.74 (m, 1H)*, 5.43 – 5.38 (m, 1H)*, 4.93 (d, *J* = 6.3 Hz, 1H), 4.84 – 4.78 (m, 1H)*, 4.52 (d, *J* = 11.2 Hz, 1H), 4.22 – 4.16 (m, 2H), 4.15 – 4.10 (m, 2H)*, 3.81 (s, 3H), 3.58 (s, 2H), 3.26 – 3.17 (m, 2H), 2.06 – 1.89 (m, 2H)*, 1.86 – 1.69 (m, 2H)*, 1.64 (s, 3H), 1.31 – 1.26 (m, 1H), 1.06 – 0.95 (m, 2H)*, 0.03 (s, 9H) ppm.
* marks signals in which protons of the keto/enol-tautomers overlap.
^{13}C–NMR (101 MHz, $CDCl_3$): δ = 200.3, 166.8, 159.8, 138.3, 137.6, 136.0, 129.9, 128.0, 124.8, 118.6 (q, *J* = 320.9 Hz), 114.1, 98.7, 71.6, 64.1, 59.4, 55.4, 53.1, 50.8, 35.9, 35.0, 26.8, 17.5, 16.2, –1.4 ppm.
^{19}F–NMR (376 MHz, $CDCl_3$) δ = –73.1 ppm.
IR (Diamond-ATR, neat): 3405, 2955, 1742, 1716, 1515, 1249, 1213, 1141, 859, 837 ppm.
HRMS (ESI) calc. for $C_{28}H_{39}F_3NaO_{10}SSi^+$ $[M+Na]^+$: 675.1878; found: 675.1857.
$[\alpha]_D^{20}$: 25.8 (c = 0.62, CH_2Cl_2).

Bromide 9

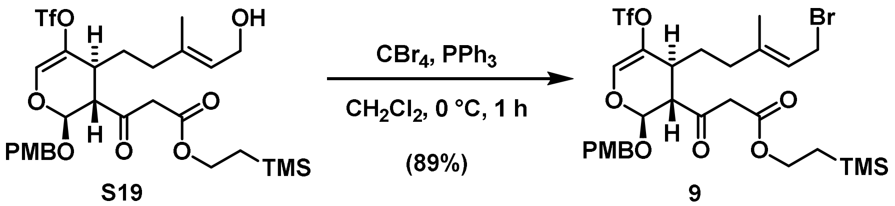

S19 **9**

Carbon tetrabromide (2.64 g, 7.87 mmol, 1.20 equiv) was added to a solution of alcohol **S19** (4.28 g, 6.56 mmol, 1 equiv) and triphenylphosphine (2.08 g, 7.87 mmol, 1.20 equiv) in dichloromethane (65 mL) at 0 °C. After 1 h, water (75 mL) was added to the reaction mixture. The layers were separated and the aqueous phase was extracted with dichloromethane (2 × 70 mL). The combined organic phases were dried over magnesium sulfate and the dried solution was filtrated. The filtrate was concentrated and the residue was purified by flash column chromatography on silica gel (15% diethyl ether in pentane) to give bromide **9** (4.17 g, 89%) as a mixture of keto-enol tautomers.

***9** was obtained as a mixture of keto/enol-tautomers; only signals of the major keto form are reported.*
TLC (40% diethyl ether in pentane): R_f = 0.67 (UV, CAM).
^{1}H–NMR (400 MHz, $CDCl_3$): δ = 7.25 – 7.16 (m, 2H)*, 6.92 – 6.83 (m, 2H)*, 6.76 (d, *J* = 1.4 Hz, 1H), 5.58 – 5.47 (m, 1H)*, 4.92 (d, *J* = 6.6 Hz, 1H), 4.82 (d, *J* = 11.2 Hz, 1H), 4.52 (d, *J* = 11.2 Hz, 1H), 4.24 – 4.15 (m, 2H), 3.99 (s, 1H), 3.96 (s, 1H), 3.81 (s, 3H), 3.61 (d, *J* = 16.0 Hz, 1H), 3.54 (d, *J* = 16.0 Hz, 1H), 3.27 – 3.17 (m, 2H), 2.00 (tdd, *J* = 23.0, 13.8, 6.9 Hz, 2H)*, 1.88 – 1.64 (m, 5H)*, 1.02 – 0.96 (m, 2H)*, 0.04 (s, 9H) ppm.
* marks signals in which protons of the keto/enol-tautomers overlap.
^{13}C–NMR (101 MHz, $CDCl_3$): δ = 200.2, 166.8, 159.9, 142.2, 137.7, 135.8, 129.9, 128.0, 121.7, 118.7 (q, *J* = 320.7 Hz), 114.2, 98.8, 71.6, 64.1, 55.4, 53.0, 50.9, 36.0, 35.0, 29.2, 26.7, 17.5, 15.9, –1.4 ppm.
^{19}F–NMR (376 MHz, $CDCl_3$) δ = –73.1 ppm.
IR (Diamond–ATR, neat): 2955, 1716, 1656, 1515, 1421, 1249, 1213, 1141, 859, 837 cm^{-1}.
HRMS (ESI) calc. for $C_{28}H_{38}BrF_3NaO_9SSi^+$ $[M+Na]^+$: 737.1033; found: 737.1011.
$[\alpha]_D^{20}$: 19.4 (c = 0.34, CH_2Cl_2).

Ketones 23

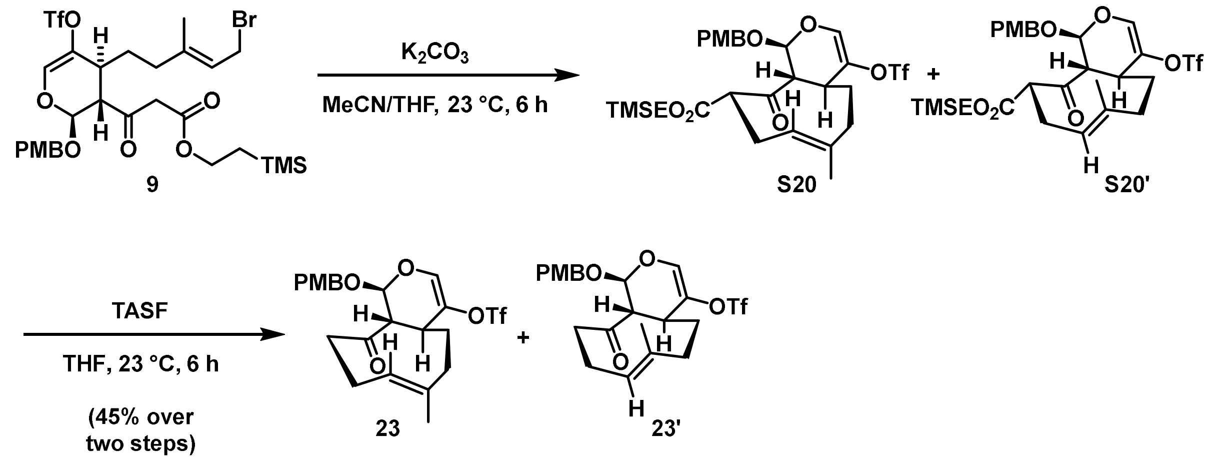

Potassium carbonate (593 mg, 4.25 mmol, 2.50 equiv) was added to a solution of bromide **9** (1.27 g, 1.74 mmol, 1 equiv) in a mixture of acetonitrile (80 mL) and tetrahydrofuran (8 mL) at 23 °C. After 6 h, aqueous saturated ammonium chloride solution (100 mL) and dichloromethane (150 mL) were added to the reaction mixture. The layers were separated and the aqueous phase was extracted with dichloromethane (2 × 100 mL). The combined organic phases were dried over magnesium sulfate and the dried solution was filtrated. The filtrate was concentrated to give the cyclized ketoester as a mixture of conformational diastereomers **S20** and **S20'** (ratio 1.4:1. 1.06 g), which was pure enough to be used in the following step without further purification.

For the following decarboxylation step the obtained crude product was split into two batches, which were run in parallel and combined for purification.

Tris(dimethylamino)sulfonium difluorotrimethylsilicate (262 mg, 951 µmol, 1.20 equiv) was added to a solution of conformational isomers **S20** and **S20'** (assumed pure: 503 mg, 792 µmol, 1 equiv) in tetrahydrofuran (16 mL) at 23 °C. After 6 h, an aqueous saturated solution of sodium hydrogen carbonate (75 mL) and dichloromethane (75 mL) were added to the reaction mixture. The layers were separated and the aqueous phase was extracted with dichloromethane (2 × 70 mL). The combined organic phases were dried over magnesium sulfate and the dried solution was filtrated. The filtrate was concentrated and the residue obtained from both in parallel conducted reaction batches was purified by flash column chromatography on silica gel (10% diethyl ether in pentane) to give ketones **23** and **23'** (379 mg, 45% over two steps) as a mixture of conformational isomers (ratio 1.2:1).

TLC (20% diethyl ether in pentane): R_f = 0.49 (UV, CAM).

S20

^{1}H–NMR (700 MHz, $CDCl_3$): δ = 7.22 (d, J = 8.6 Hz, 2H), 6.87 – 6.84 (m, 2H)*, 6.69 (d, *J* = 1.1 Hz, 1H), 5.52 (ddt, *J* = 12.6, 4.3, 1.8 Hz, 1H), 4.77 – 4.73 (m, 1H)*, 4.62 (d, *J* = 8.8 Hz, 1H), 4.58 (d, *J* = 11.2 Hz, 1H), 4.14 – 4.01 (m, 3H)*, 3.80 (s, 3H), 3.18 – 3.11 (m, 2H), 2.63 – 2.59 (m, 1H)*, 2.57 (d, *J* = 10.2 Hz, 1H), 2.43 – 2.48 (m, 1H), 2.21 – 2.15 (m, 1H), 1.73 – 1.63 (m, 2H), 1.55 (s, 3H), 0.94 – 0.84 (m, 2H)*, 0.01 (s, 9H) ppm.

* marks signals in which protons from both conformational diastereomers overlap.

^{13}C–NMR (176 MHz, $CDCl_3$): δ = 203.4, 169.0, 159.6, 138.6, 138.6, 137.4, 129.6, 128.6, 122.3, 118.7 (d, *J* = 320.6 Hz), 114.0, 101.3, 71.8, 63.9, 63.5, 58.4, 55.4, 37.6, 34.3, 34.2, 29.1, 21.7, 17.6, –1.5 ppm.
^{19}F–NMR (376 MHz, $CDCl_3$) δ = –73.2 ppm.

S20'

^{1}H–NMR (700 MHz, $CDCl_3$): δ = 7.23 (d, *J* = 8.6 Hz, 2H), 6.87 – 6.83 (m, 2H)*, 6.71 (s, 1H), 5.22 – 5.19 (m, 1H), 4.77 – 4.73 (m, 1H)*, 4.71 (d, *J* = 8.0 Hz, 1H), 4.53 (d, *J* = 11.0 Hz, 1H), 4.14 – 4.01 (m, 3H)*, 3.80 (s, 3H), 2.89 (dd, *J* = 8.0, 5.1 Hz, 1H), 2.80 – 2.74 (m, 1H), 2.69 – 2.65 (m, 1H), 2.63 – 2.59 (m, 1H)*, 2.26 – 2.22 (m, 1H), 2.10 – 2.04 (m, 2H), 1.81 (s, 3H), 1.57 – 1.53 (m, 1H)*, 0.94 – 0.84 (m, 2H)*, 0.01 (s, 9H) ppm.
* marks signals in which protons from both conformational diastereomers overlap.
^{13}C–NMR (176 MHz, $CDCl_3$): δ = 205.9, 169.3, 159.7, 140.4, 139.4, 137.3, 129.7, 128.6, 122.3, 118.7 (d, *J* = 320.7 Hz), 114.0, 101.3, 71.9, 63.7, 61.9, 55.4, 55.4, 39.5, 39.1, 32.4, 25.1, 17.6, 16.5, –1.5 ppm.
^{19}F–NMR (376 MHz, $CDCl_3$) δ = –73.1 ppm.
IR (Diamond–ATR, neat): 2953, 1737, 1713, 1515, 1421, 1249, 1212, 1142, 857, 838 cm^{-1}.
HRMS (ESI) calc. for $C_{28}H_{37}F_3NaO_9SSi^+$ $[M+Na]^+$: 657.1772; found: 657.1754.
$[\alpha]_D^{20}$:51.3 (c = 0.52, CH_2Cl_2).

The obtained analytical data for **23** were in full agreement with our previously observed data.

Alkene S21

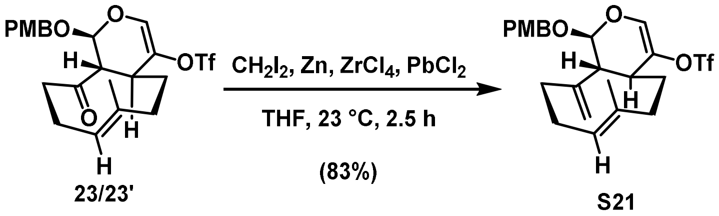

Diiodomethane (927 µL, 11.4 mmol, 12.0 equiv) was added to a suspension of zinc dust (2.28 g, 34.1 mmol, 36.0 equiv) and lead(II) chloride (399 mg, 1.42 mmol, 1.50 equiv) in tetrahydrofuran (10 mL) at 23 °C. After 10 min, the reaction mixture started boiling and was cooled to 0 °C. After 10 min, the reaction mixture was allowed to rewarm to 23 °C. After 1 h, zirconium (IV) chloride (1.00 g, 4.27 mmol, 4.50 equiv) was added to the reaction mixture. After 1 h, a solution conformational diastereomers **23** and **23'** (465 mg, 948 µmol, 1 equiv) in tetrahydrofuran (2 mL) was added to the grey/green reaction mixture. After 2.5 h, the reaction mixture was poured into a mixture of an aqueous saturated sodium hydrogen carbonate solution (60 mL) and dichloromethane (30 mL). The layers were separated and the aqueous phase was extracted with dichloromethane (2 × 30 mL). The combined organic phases were dried over magnesium sulfate and the dried solution was filtrated. The filtrate was concentrated and the residue was purified by flash column chromatography on silica gel (8% diethyl ether in pentane) to give alkene **S21** (383 mg, 83%) as a mixture of conformational isomers (ratio 10:1).

Note: Only the signals of the major conformational diastereomer are reported.

TLC (10% diethyl ether in pentane): R_f = 0.68 (UV, CAM).

^{1}H–NMR (400 MHz, $CDCl_3$): δ = 7.20 (d, *J* = 8.7 Hz, 2H), 6.88 (d, *J* = 8.7 Hz, 2H), 6.66 (d, *J* = 2.0 Hz, 1H), 5.37 – 5.30 (m, 1H), 4.94 (s, 1H), 4.92 (d, *J* = 0.8 Hz, 1H), 4.71 (d, *J* = 2.6 Hz, 1H), 4.66 (d, *J* = 11.8 Hz, 1H), 4.42 (d, *J* = 11.8 Hz, 1H), 3.81 (s, 3H), 2.51 – 2.40 (m, 2H), 2.24 – 2.06 (m, 5H), 1.99 (td, *J* = 12.6, 4.9 Hz, 1H), 1.91 – 1.85 (m, 1H), 1.76 – 1.65 (m, 4H) ppm.

^{13}C–NMR (101 MHz, $CDCl_3$): δ = 159.4, 150.2, 139.2, 135.9, 135.5, 129.5, 129.2, 124.8, 118.7 (d, *J* = 320.4 Hz), 114.4, 113.9, 98.5, 69.6, 55.4, 52.6, 40.9, 39.4, 35.2, 28.3, 25.3, 17.0 ppm.

^{19}F–NMR (376 MHz, $CDCl_3$) δ = –73.7 ppm.

IR (Diamond–ATR, neat): 2932, 2858, 1587, 1419, 1248, 1211, 1142, 1071, 869, 822 cm^{-1}.

HRMS (ESI) calc. for $C_{23}H_{27}F_3NaO_6S^+$ $[M+Na]^+$: 511.1373; found: 511.1360.

$[\alpha]_D^{20}$: 95.0 (c = 0.40, CH_2Cl_2).

***Table S1*: Unsuccessful conditions for the methenylation of ketones **23** and **23’**.**

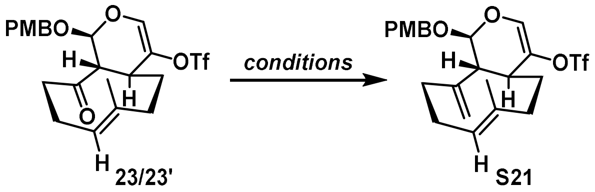

	**conditions**	**result**
1	CH_3PPh_3Br (2.1 equiv.), KHMDS (2 equiv.), THF, 0 °C	decomposition
2	Tebbe reagent (2.5 equiv.), THF, –78 °C to 55 °C	no conversion
3	$Cp_2Ti(CH_3)_2$, toluene, 60 °C	no conversion
4	$[RhCl(PPh_3)_3]$, PPh_3, $TMSCHN_2$, *i*-PrOH, THF, 60 °C	no conversion
5	$TMSCH_2MgCl$ (3 equiv.), THF, –78 °C to 23 °C	slow decomposition
6	CH_2Br_2, Zn, $TiCl_4$, THF, 50 °C	olefination and double bond isomerization

Aldehyde 25

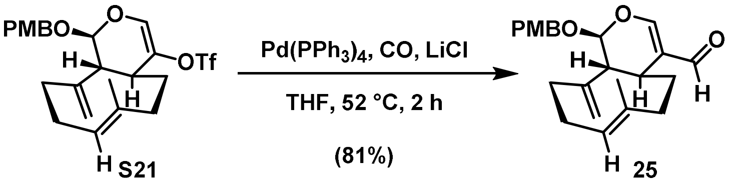

Note: Tetrahydrofuran was degassed via freeze-pump-thaw (three cycles) prior to use.

Tetrakis(triphenylphosphine)palladium(0) (125 mg, 107 µmol, 15.0 mol%), and flame dried lithium chloride (307 mg, 7.16 mmol, 10.0 equiv) were added to a solution of alkene **S21** (350 mg, 716 µmol, 1 equiv) in tetrahydrofuran (10 mL). The flask was fitted with a balloon filled with carbon monoxide gas, and the solution was sparged with carbon monoxide for 10 minutes. The yellow reaction mixture was then heated to 52 °C under a carbon monoxide atmosphere. A solution of tributyltin hydride (299 µL, 1.07 mmol, 1.40 equiv) in tetrahydrofuran (0.7 mL) was added dropwise over a period of 1.3 h via syringe pump. After 30 min, the reaction mixture was cooled to 23 °C and water (30 mL) and dichloromethane (30 mL) were added. The layers were separated, and the aqueous phase was extracted with dichloromethane (2 × 30 mL). The combined organic phases were dried over magnesium sulfate and the dried solution was filtrated. The filtrate was concentrated, and the residue was purified by flash column chromatography on silica gel (2% diethyl ether in pentane until tin impurities were removed, then 30% diethyl ether in pentane) to give aldehyde **25** (214 mg, 81%) as a colorless oil.

TLC (20% diethyl ether in pentane): R_f = 0.23 (UV, CAM).

^{1}H–NMR (400 MHz, $CDCl_3$): δ = 9.29 (s, 1H), 7.20 (d, *J* = 8.6 Hz, 2H), 7.17 (d, *J* = 1.5 Hz, 1H), 6.88 (d, *J* = 8.6 Hz, 2H), 5.45 – 5.35 (m, 1H), 4.92 (d, *J* = 2.3 Hz, 1H), 4.83 (s, 1H), 4.71 (d, *J* = 11.9 Hz, 1H), 4.67 – 4.64 (m, 1H), 4.50 (d, *J* = 11.9 Hz, 1H), 3.81 (s, 3H), 2.50 – 2.41 (m, 2H), 2.29 – 2.06 (m, 6H), 1.84 – 1.77 (m, 1H), 1.70 (d, *J* = 1.4 Hz, 3H), 1.67 – 1.57 (m, 1H) ppm.

^{13}C–NMR (101 MHz, $CDCl_3$): δ = 191.4, 161.0, 159.4, 151.2, 136.4, 129.4, 129.1, 125.2, 124.4, 114.0, 113.3, 100.7, 70.1, 55.4, 49.8, 40.0, 35.7, 34.7, 30.8, 25.6, 17.3 ppm.

IR (Diamond–ATR, neat): 2934, 2855, 1674, 1634, 1514, 1249, 1216, 1171, 1085, 859 cm^{-1}.

HRMS (ESI) calc. for $C_{23}H_{28}NaO_4^+$ $[M+Na]^+$: 391.1880; found: 391.1869.

$[\alpha]_D^{20}$: 252.3 (c = 0.15, CH_2Cl_2).

Hemiacetal S22α/β

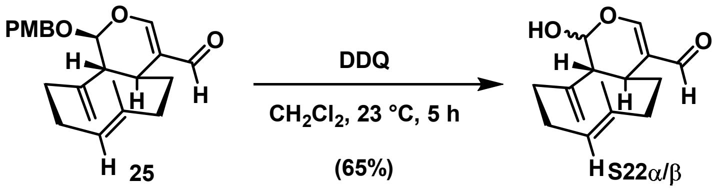

Water (0.125 mL) and 2,3-dichloro-5,6-dicyano-1,4-benzoquinone (123 mg, 528 µmol, 1.80 equiv) were sequentially added to a solution of aldehyde **25** (108 mg, 293 µmol, 1 equiv) in dichloromethane (4 mL) at 0 °C. After 5 h, an aqueous saturated solution of sodium hydrogencarbonate (30 mL) and dichloromethane (25 mL) were added to the reaction mixture. The layers were separated and the aqueous phase was extracted with dichloromethane (2 × 25 mL). The combined organic phases were dried over magnesium sulfate and the dried solution was filtrated. The filtrate was concentrated and the residue was purified by flash column chromatography on silica gel (45% diethyl ether in pentane) to give an inseparable mixture of hemiacetal **S22α/β** (47.0 mg, 65%, d.r. =1.8:1).

S22α

TLC (20% ethyl acetate in cyclohexane): R_f = 0.18 (UV, CAM).

^{1}H–NMR (600 MHz, $CDCl_3$): δ = 9.30 (s, 1H), 7.23 (s, 1H), 5.48 (t, *J* = 8.3 Hz, 1H), 5.05 (d, *J* = 1.6 Hz, 1H), 5.03 (br, 1H), 4.72 (s, 1H), 3.26 (br, 1H), 2.61 (d, *J* = 9.3 Hz, 1H), 2.53 – 2.44 (m, 1H)*, 2.39 – 2.34 (m, 1H), 2.33 – 2.09 (m, 4H)*, 2.02 (d, *J* = 4.0 Hz, 1H), 1.98 – 1.92 (m, 1H), 1.71 (s, 3H), 1.10 (tdd, *J* = 13.0, 9.3, 4.0 Hz, 1H) ppm.

* marks signals in which protons from both epimers overlap.

^{13}C–NMR (151 MHz, $CDCl_3$): δ = 191.0, 161.9, 148.7, 136.0, 124.3, 123.3, 115.5, 95.5, 51.6, 39.6, 37.3, 36.7, 32.7, 25.5, 17.3 ppm.

S22β

^{1}H–NMR (600 MHz, $CDCl_3$): δ = 9.29 (s, 1H), 7.18 (s, 1H), 5.41 (dd, *J* = 10.3, 5.4 Hz, 1H), 5.17 (d, *J* = 3.6 Hz, 1H), 4.94 (s, 1H), 4.78 (s, 1H), 3.02 (br s, 1H), 2.59 – 2.56 (m, 1H), 2.53 – 2.44 (m, 1H)*, 2.33 – 2.10 (m, 5H)*, 2.09 – 2.06 (m, 1H), 1.86 – 1.82 (m, 1H), 1.68 (s, 3H), 1.59 – 1.47 (m, 1H) ppm.

* marks signals in which protons from both epimers overlap.

^{13}C–NMR (151 MHz, $CDCl_3$): δ = 191.0, 161.0, 150.0, 136.5, 125.2, 124.6, 115.5, 96.6, 52.5, 40.0, 34.7, 34.0, 31.6, 26.5, 17.6 ppm.

IR (Diamond–ATR, neat): 3399, 2926, 2855, 1662, 1621, 1261, 1174, 1158, 897, 856 cm^{-1}.

HRMS (ESI) calc. for $C_{15}H_{20}NaO_3^+$ $[M+Na]^+$: 271.1305; found: 271.1302.

$[\alpha]_D^{20}$: 87.7 (c = 0.17, CH_2Cl_2).

Acetates 26α and 26β

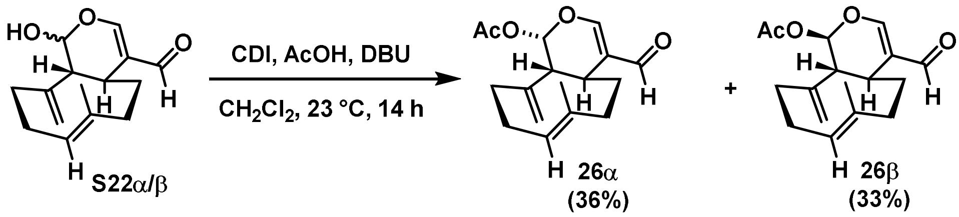

Acetic acid (188 µL, 3.27 mmol, 19.5 mmol) and 1,8-diazabicyclo[5.4.0]undec-7-ene (5.12 µL, 33.6 µmol, 0.200 equiv) were added sequentially to a solution of 1,1'-carbonyldiimidazole (515 mg, 3.08 mmol, 18.3 equiv) in dichloromethane (2.5 mL) at 23 °C. After 20 min, a solution of hemiacetal **S22α/β** (41.7 mg, 168 µmol, 1 equiv) in dichloromethane (1 mL) was added at 23 °C. After 16 h, water (15 mL) and dichloromethane (10 mL) were added to the reaction mixture. The layers were separated, and the aqueous phase was extracted with dichloromethane (2 × 10 mL). The combined organic phases were dried over magnesium sulfate and the dried solution was filtrated. The filtrate was concentrated, and the residue was purified by flash column chromatography on silica gel (25% diethyl ether in pentane) to give acetate **26β** (16.1 mg, 33%) as a colorless oil and acetate **26α** (17.7 mg, 36%) as a colorless oil.

26α

TLC (40% diethyl ether in pentane): R_f = 0.39 (UV, CAM).

^{1}H–NMR (700 MHz, C_6D_6, 70 °C): δ = 9.05 (s, 1H), 6.52 (s, 1H), 6.04 (d, *J* = 3.5 Hz, 1H), 5.35 (dd, *J* = 10.3, 6.0 Hz, 1H), 4.82 (s, 1H), 4.71 (s, 1H), 2.75 – 2.72 (m, 1H), 2.47 – 2.41 (m, 1H), 2.31 – 2.22 (m, 1H), 2.13 – 2.08 (m, 1H), 2.07 – 1.99 (m, 4H), 1.95 – 1.88 (m, 1H), 1.59 (s, 3H), 1.48 (s, 3H), 1.04 – 0.97 (m, 1H) ppm.

^{13}C–NMR (176 MHz, C_6D_6, 70 °C): δ = 189.3, 168.3, 159.8, 148.4, 135.7, 125.5, 125.0, 115.9, 93.5, 51.0, 40.0, 35.4, 34.9, 33.8, 27.2, 20.2, 17.4 ppm.

IR (Diamond–ATR, neat): 2928, 2858, 1761, 1675, 1629, 1214, 1183, 1118, 1073, 947 cm^{-1}.

HRMS (ESI) calc. for $C_{17}H_{22}NaO_4^+$ $[M+Na]^+$: 313.1410; found: 313.1400.

$[\alpha]_D^{20}$: –29.7 (c = 1.31, CH_2Cl_2).

26β

TLC (40% diethyl ether in pentane): R_f = 0.34 (UV, CAM).

^{1}H–NMR (400 MHz, $CDCl_3$): δ = 9.33 (s, 1H), 7.19 (d, *J* = 1.7 Hz, 1H), 6.11 (d, *J* = 1.5 Hz, 1H), 5.48 – 5.39 (m, 1H), 4.89 (s, 1H), 4.68 (s, 1H), 2.54 – 2.40 (m, 2H), 2.32 – 2.10 (m, 5H), 2.09 (s, 3H), 2.04 – 2.02 (m, 1H), 1.93 – 1.85 (m, 1H), 1.66 (d, *J* = 1.5 Hz, 3H), 1.53 – 1.44 (m, 1H) ppm.

^{13}C–NMR (101 MHz, $CDCl_3$): δ = 191.0, 169.2, 160.2, 150.4, 136.0, 124.8, 124.4, 113.9, 92.9, 48.1, 39.9, 35.7, 34.0, 30.6, 25.4, 21.0, 17.1 ppm.

IR (Diamond–ATR, neat): 2928, 2855, 1759, 1678, 1639, 1217, 1183, 1163, 981, 906 cm^{-1}.

HRMS (ESI) calc. for $C_{17}H_{22}NaO_4^+$ $[M+Na]^+$: 313.1410; found: 313.1400.

$[\alpha]_D^{20}$: 198.6 (c = 0.48, CH_2Cl_2).

Recycling of S26α

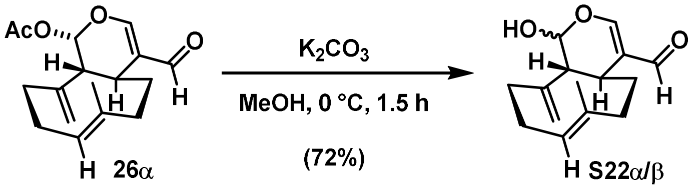

Potassium carbonate (86.3 mg, 618 µmol, 3.00 equiv) was added to a solution of acetate **26α** (59.8 mg, 206 µmol, 1 equiv) in methanol (2.5 mL) at 0 °C. After 1 h, aqueous saturated ammonium chloride solution (10 mL) and dichloromethane (10 mL) were added to the reaction mixture. The layers were separated and the aqueous phase was extracted with dichloromethane (2 × 10 mL). The combined organic phases were dried over magnesium sulfate and the dried solution was filtrated. The filtrate was concentrated and the residue was purified by flash column chromatography on silica gel (50% diethyl ether in pentane) to give hemiacetal **S22α/β** (37.2 mg, 72%) as a colorless oil.

Alcohols S23a and S23b

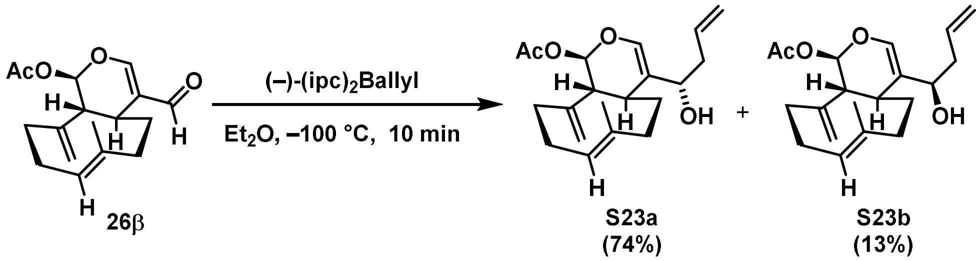

A solution of (–)-(ipc)$_2$B(allyl)borane (1.00 M in pentane, 101 µL, 101 µmol, 1.40 equiv) diluted with diethyl ether (0.4 mL) was added slowly to a solution of acetate **26β** (21.0 mg, 72.3 µmol, 1 equiv) in diethyl ether (1.5 mL) at –100 °C. After 10 min, the reaction mixture was warmed to 23 °C and sodium perborate tetrahydrate (206 mg 1.30 mmol, 18.0 equiv), water (1 mL) and tetrahydrofuran (1.5 mL) were added sequentially. After 5 h, water (15 mL) and dichloromethane (15 mL) were added to the reaction mixture. The layers were separated and the aqueous phase was extracted with dichloromethane (2 × 10 mL). The combined organic phases were dried over magnesium sulfate and the dried solution was filtrated. The filtrate was concentrated and the residue was purified by flash column chromatography on silica gel (25% diethyl ether in pentane) to give alcohol **S23b** (3.1 mg, 13%) as a colorless oil and alcohol **S23a** (17.9 mg, 74%) as a colorless oil.

S23a

TLC (40% diethyl ether in pentane): R_f = 0.20 (CAM).

^{1}H–NMR (400 MHz, $CDCl_3$): δ = 6.42 (d, *J* = 1.9 Hz, 1H), 5.89 (d, *J* = 1.8 Hz, 1H), 5.85 – 5.70 (m, 1H), 5.43 – 5.36 (m, 1H), 5.20 – 5.11 (m, 2H), 4.87 (s, 1H), 4.80 (d, *J* = 1.0 Hz, 1H), 4.16 (td, *J* = 7.7, 7.1, 2.9 Hz, 1H), 2.52 – 2.34 (m, 3H), 2.33 – 2.03 (m, 10H), 1.97 (s, 1H), 1.67 (s, 3H), 1.60 – 1.48 (m, 2H) ppm.

^{13}C–NMR (101 MHz, $CDCl_3$): δ = 169.9, 151.5, 138.6, 136.1, 134.7, 124.5, 119.7, 118.2, 113.3, 92.2, 72.7, 49.6, 40.2, 39.6, 36.9, 35.7, 31.0, 25.2, 21.3, 16.9 ppm.

IR (Diamond–ATR, neat): 3456, 2932, 2855, 1737, 1664, 1229, 1156, 1019, 927, 873 cm^{-1}.

HRMS (ESI) calc. for $C_{20}H_{28}NaO_4^+$ [M+Na]$^+$: 355.1880; found: 355.1874.

$[\alpha]_D^{20}$: 142.3 (c = 0.22, CH_2Cl_2).

S23b

TLC (40% diethyl ether in pentane): R_f = 0.25 (CAM).

^{1}H–NMR (400 MHz, $CDCl_3$): δ = 6.52 (t, *J* = 1.6 Hz, 1H), 5.87 (d, *J* = 2.2 Hz, 1H), 5.86 – 5.76 (m, 1H), 5.40 – 5.34 (m, 1H), 5.22 – 5.20 (m, 1H), 5.19 – 5.16 (m, 1H), 4.89 (s, 1H), 4.85 (d, *J* = 1.1 Hz, 1H), 4.29 (dt, *J* = 8.1, 4.5 Hz, 1H), 2.53 – 2.40 (m, 2H), 2.33 – 2.19 (m, 4H), 2.17 – 2.08 (m, 1H), 2.06 (s, 3H), 2.05 – 1.97 (m, 3H), 1.83 – 1.76 (m, 1H), 1.67 (s, 3H), 1.64 (d, *J* = 4.4 Hz, 1H), 1.63 – 1.51 (m, 1H) ppm.

^{13}C–NMR (101 MHz, $CDCl_3$): δ = 169.9, 151.5, 136.5, 135.7, 134.1, 124.8, 119.9, 119.2, 113.4, 92.2, 69.6, 49.7, 41.6, 40.4, 38.1, 35.7, 30.4, 25.3, 21.3, 16.9 ppm.

IR (Diamond–ATR, neat): 3458, 292, 2854, 1735, 1670, 1229, 1155, 1018, 929, 870 cm^{-1}.

HRMS (ESI) calc. for $C_{20}H_{28}NaO_4^+$ [M+Na]$^+$: 355.1880; found: 355.1871.

$[\alpha]_D^{20}$: 150.0 (c = 0.09, CH_2Cl_2).

Allylic acetate 27

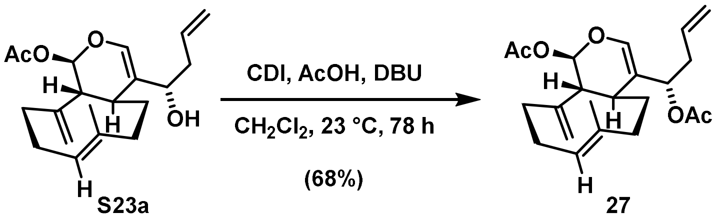

Acetic acid (70.6 µL, 1.23 mmol, 39.0 mmol) and 1,8-diazabicyclo[5.4.0]undec-7-ene (0.1 M stock solution in dichloromethane, 126 µL, 12.6 µmol, 0.400 equiv) were added sequentially to a solution of CDI (193 mg, 1.19 mmol, 37.6 equiv) in dichloromethane (0.8 mL) at 23 °C. After 20 min, a solution of alcohol **S23a** (10.5 mg, 31.6 µmol, 1 equiv) in dichloromethane (0.5 mL) was added at 23 °C. After 78 h, water (10 mL) and dichloromethane (10 mL) were added to the reaction mixture. The layers were separated, and the aqueous phase was extracted with dichloromethane (2 × 10 mL). The combined organic phases were dried over magnesium sulfate and the dried solution was filtrated. The filtrate was concentrated, and the residue was purified by flash column chromatography on silica gel (20% diethyl ether in pentane) to give acetate **27** (8.0 mg, 68%) as a colorless oil.

TLC (40% diethyl ether in pentane): R_f = 0.56 (CAM).
^{1}H–NMR (400 MHz, $CDCl_3$): δ = 6.51 (d, *J* = 1.9 Hz, 1H), 5.87 (d, *J* = 1.9 Hz, 1H), 5.75 – 5.64 (m, 1H), 5.40 – 5.33 (m, 2H), 5.15 – 5.05 (m, 2H), 4.87 (s, 1H), 4.79 (d, *J* = 1.2 Hz, 1H), 2.53 – 2.36 (m, 3H), 2.32 – 1.99 (m, 12H), 1.99 – 1.92 (m, 2H), 1.66 (d, *J* = 1.3 Hz, 3H), 1.58 – 1.47 (m, 1H) ppm.
^{13}C–NMR (101 MHz, $CDCl_3$): δ = 170.5, 169.8, 151.2, 140.7, 135.9, 133.5, 124.6, 118.1, 116.2, 113.5, 91.9, 73.8, 49.4, 40.1, 37.2, 36.9, 35.6, 30.6, 25.2, 21.5, 21.2, 16.9 ppm.
IR (Diamond–ATR, neat): 2933, 2855, 1736, 1664, 1372, 1233, 1163, 1015, 948, 872 cm^{-1}.
HRMS (ESI) calc. for $C_{22}H_{30}NaO_5^+$ $[M+Na]^+$: 397.1985; found: 397.1978.
$[\alpha]_D^{20}$: 66.4 (c = 0.23, CH_2Cl_2).

Waixenicin A (1)

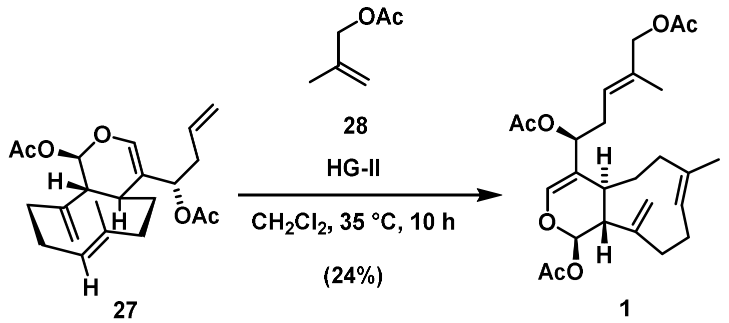

Note: The reaction was carried out in two parallel batches, which were combined for purification by column chromatography.

A solution of Hoveyda–Grubbs 2nd generation catalyst (431 µg, 668 nmol, 10 mol%) in degassed (three cycles of freeze-pump-thaw) dichloromethane (0.4 mL) was added to acetate **27** (2.5 mg, 6.7 µmol, 1 equiv) and alkene **28** (25.4 µL, 200 µmol, 30.0 equiv) and the resulting green reaction mixture was heated to 35 °C. After 10 h, the reaction mixture was allowed to cool to 23 °C and evaporated. The residues of both in parallel conducted reaction runs were combined and purified by flash column chromatography on silica gel (25% diethyl ether in pentane) to give waixenicin A (**1**) (1.5 mg, 24%) as a colorless oil.

TLC (40% diethyl ether in pentane): R_f = 0.44 (CAM).

^{1}H–NMR (700 MHz, $CDCl_3$): δ = 6.50 (d, *J* = 1.8 Hz, 1H), 5.88 (d, *J* = 1.9 Hz, 1H), 5.37 – 5.32 (m, 3H), 4.87 (s, 1H), 4.77 (s, 1H), 4.43 (s, 2H), 2.54 – 2.45 (m, 2H), 2.41 – 2.36 (m, 1H), 2.30 – 2.19 (m, 3H), 2.15 – 2.01 (m, 12H), 1.98 – 1.94 (m, 2H), 1.70 (s, 3H), 1.66 (s, 3H), 1.53 (s, 1H) ppm.

^{1}H–NMR (700 MHz, C_6D_6): δ = 6.56 (d, *J* = 2.0 Hz, 1H), 6.25 (d, *J* = 2.0 Hz, 1H), 5.52 (t, *J* = 7.4 Hz, 1H), 5.46 – 5.41 (m, 1H), 5.33 (t, *J* = 6.9 Hz 1H), 4.99 (d, *J* = 1.4 Hz, 1H), 4.85 (s, 1H), 4.41 (s, 2H), 2.46 (dt, *J* = 14.5, 7.2 Hz, 1H), 2.35 – 2.23 (m, 3H), 2.19 – 2.05 (m, 6H), 1.95 (dq, *J* = 12.8, 8.2 Hz, 1H), 1.71 (s, 3H), 1.69 (s, 3H), 1.68 – 1.60 (m, 1H), 1.58 (s, 3H), 1.56 (d, *J* = 1.3 Hz, 3H), 1.53 (d, *J* = 1.3 Hz, 3H) ppm.

^{13}C–NMR (176 MHz, $CDCl_3$): δ = 170.9, 170.3, 169.6, 151.2, 140.7, 135.8, 133.0, 124.4, 123.5, 115.8, 113.2, 91.7, 74.2, 69.6, 49.2, 40.0, 36.8, 35.5, 30.9, 30.5, 25.0, 21.4, 21.0, 21.0, 16.8, 14.4 ppm.

^{13}C–NMR (176 MHz, CD_2Cl_2): δ = 171.0, 170.5, 169.9, 152.0, 141.0, 136.4, 133.4, 124.5, 123.8, 116.2, 113.1, 91.8, 74.6, 69.8, 49.5, 40.3, 37.1, 35.8, 31.2, 30.7, 25.3, 21.6, 21.2, 21.1, 16.9, 14.5 ppm.

IR (Diamond–ATR, neat): 2924, 2854, 1737, 1665, 1447, 1373, 1232, 1153, 1018, 948 cm^{-1}.

HRMS (ESI) calc. for $C_{26}H_{36}NaO_7^+$ $[M+Na]^+$: 483.2353; found: 483.2347.

$[\alpha]_D^{20}$: 59.6 (c = 0.02, MeOH).

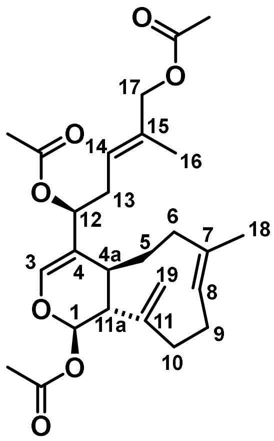

Waixenicin A

***Table S2**: Comparison of ^{1}H–NMR shifts in $CDCl_3$ for natural and synthetic waixenicin A (**1**).[5,6]*

No	^{1}H–NMR ($CDCl_3$) <u>isolated</u> waixenicin A **ppm**	^{1}H–NMR (700 MHz, $CDCl_3$) <u>synthetic</u> waixenicin A **ppm**	Δ ppm
H-1	5.84 (d, *J* = 1.6 Hz, 1H)	5.88 (d, *J* = 1.9 Hz, 1H)	+0.04
H-3	6.47 (d, *J* = 1.8 Hz, 1H)	6.50 (d, *J* = 1.8 Hz, 1H)	+0.03
H-8	5.31 (m, 1H)	5.33 (m, 3H)	+0.02
H-12	5.31 (m, 1H)	5.33 (m, 3H)	+0.02
H-14	5.31 (m, 1H)	5.33 (m, 3H)	+0.02
H-16	1.69 (br s, 3H)	1.70 (br s, 3H)	+0.01
H-17	4.41 (s, 2H)	4.43 (s, 2H)	+0.02
H-18	1.66 (br s, 3H)	1.66 (br s, 3H)	±0
H-19	4.84 (s, 1H), 4.74 (s, 1H)	4.87 (s, 1H), 4.77 (s, 1H)	+0.03, +0.03
OAc	2.07 (s, 3H); 2.05 (s, 3H); 2.03 (s, 3H)	2.07 (s, 3H); 2.05 (s, 3H); 2.04 (s, 3H)	±0, ±0+0.01

***Table S3**: Comparison of ^{1}H–NMR shifts in C_6D_6 for natural and synthetic waixenicin A (**1**).[5,6]*

No	^{1}H–NMR (MHz, C_6D_6) <u>isolated</u> waixenicin A **ppm**	^{1}H–NMR (400 MHz, C_6D_6) <u>synthetic</u> waixenicin A **ppm**	Δ ppm
H-1	6.22 (d, *J* = 1.7 Hz 1H)	6.25 (d, *J* = 2.0 Hz, 1H)	+0.03
H-3	6.54 (d, *J* = 1.7 Hz 1H)	6.56 (d, *J* = 2.0 Hz 1H)	+0.02
H-8	5.42 (dd, *J* = 7.7 Hz 1H)	5.44 (m, 1H)	+0.02
H-12	5.50 (dd, *J* = 7.4 Hz 1H)	5.52 (t, *J* = 7.4 Hz 1H)	+0.02
H-14	5.32 (dd, *J* = 6.9 Hz 1H)	5.33 (t, *J* = 6.9 Hz 1H)	+0.01
H-16	1.52 (br s, 3H)	1.53 (d, *J* = 1.3 Hz, 3H)	+0.01
H-17	4.40 (s, 2H)	4.41 (s, 2H)	+0.01
H-18	1.54 br s, 3H)	1.56 (d, *J* = 1.3 Hz, 3H)	+0.02
H-19	4.96 (s, 1H), 4.84 (s, 1H)	4.99 (s, 1H), 4.85 (s, 1H)	+0.03, +0.01
OAc	1.70 (s, 3H); 1.68 (s, 3H); 1.57 (s, 3H)	1.71 (s, 3H); 1.69 (s, 3H); 1.58 (s, 3H)	+0.01, +0.01 +0.01

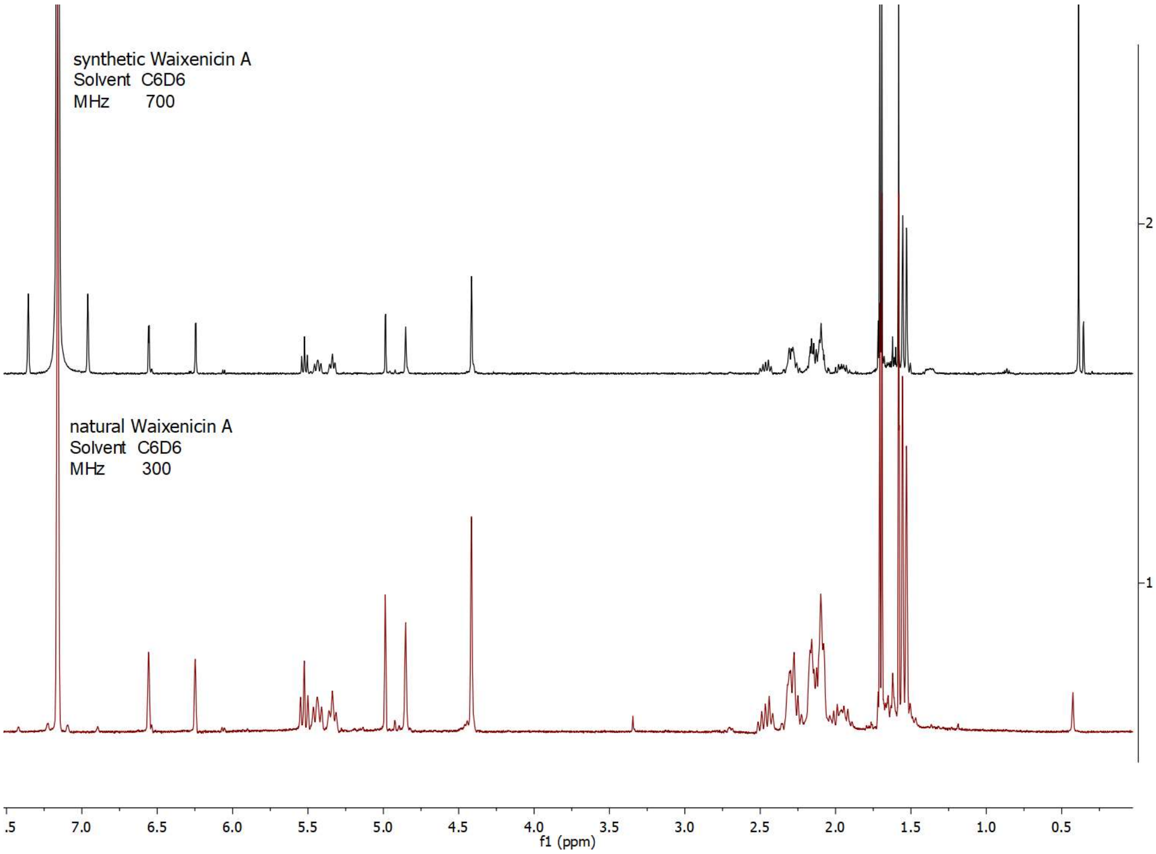

Figure S5: Overlap of the ^{1}H–NMR spectra (C_6D_6) of synthesized (top) and natural waixenicin A (**1**) (bottom, spectrum provided by Prof. F. David Horgen).

Table S4*: Comparison of ^{13}C–NMR shifts in CD_2Cl_2 for natural and synthetic waixenicin A (**1**).*[5,6]

No	^{13}C–NMR (MHz, CD_2Cl_2) isolated Waixenicin A **ppm**	^{13}C–NMR (151 MHz, CD_2Cl_2) synthetic Waixenicin A **ppm**	Δ ppm
C-1	91.8	91.8	±0
C-3	140.8	141.0	+0.2
C-4	116.1	116.2	+0.1
C-4a	37.2	37.1	–0.1
C-5	31.2/30.8	31.2/30.7	±0/–0.1
C-6	40.3	40.3	±0
C-7	136.1/133.2	136.4/133.4	+0.3/+0.2
C-8	124.4/123.7	124.5/123.8	+0.1/+0.1
C-9	25.4	25.3	–0.1
C-10	35.8	35.8	±0
C-11	151.8	152.0	+0.2
C-11a	49.6	49.5	–0.1
C-12	74.4	74.6	+0.2
C-13	31.2/30.8	31.2/30.7	0/–0.1
C-14	124.4/123.7	124.5/123.8	+0.1/+0.1
C-15	136.1/133.2	136.4/133.4	+0.3/+0.2
C-16	14.4	14.5	+0.1

C-17	69.7	69.8	+0.1
C-18	16.9	16.9	±0
C-19	113.0	113.1	+0.1
OAc	170.6, 170.1, 169.5, 21.5, 21.0, 21.0	171.0, 170.5, 169.9, 21.6, 21.2, 21.1	+0.4, +0.4, +0.4, +0.1, +0.2+, +0.1

Key insights for the side chain introduction

With large amounts of sulfones **20/20'** in hand and the desulfonylation without concomitant double bond isomerization being problematic, we decided to convert all of the material to aldehyde **S13**, which we treated as elaborate model substrate for different end game scenarios. Initially, we envisioned the removal of the PMB group and the following acetylation of the acetal as the last steps of our synthesis. However, during our studies we found that the presence of a push/pull system was crucial for the PMB-ether cleavage. In initial experiments, advanced substrates containing an allylic alcohol with parts of the side chain already preinstalled only decomposed when subjected to deprotection conditions. To further investigate the removal, we prepared **S24** as model substrate.

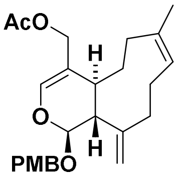

S24

Subjection of **S24** to standard oxidative deprotection conditions (Table **SXX**, entries 1 and 2) thereby led to complete decomposition of the starting material. The attempted cleavage with BCl_3•SMe_2 (entry 3) resulted in the formation of multiple unknown products. Attempting to activate the acetal by treatment with TMSOTf (entry 4) then again resulted in decomposition. Similar results were obtained upon treatment with acid (entry 5) and under photochemical conditions (entry 6)

Table S5: Investigation of the PMB ether cleavage**.**

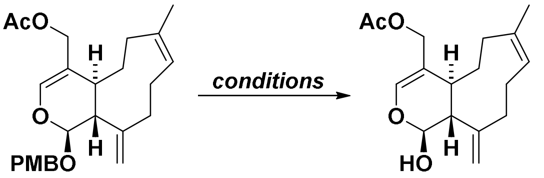

	conditions	result
1	DDQ, CH_2Cl_2/H_2O, 0 °C	decomposition
2	CAN $MeCN/H_2O$, 0 °C	decomposition
3	BCl_3•SMe_2, CH_2Cl_2, 0 °C	unknown products
4	TMSOTf, CH_2Cl_2, –78 °C to 23 °C	decomposition
5	TFA, CH_2Cl_2, 23 °C	decomposition
6	$(Ir[dF(CF_3)ppy]_2(dtbpy))PF_6$,$BrCCl_3$, H2O, blue LEDs	decomposition

We also obtained similar results, when we attempted the deprotection on the stage of triflate **S12**. Given these results, we had to adapt our envisioned end game strategies. With the presence of a push/pull system being necessary to effect removal of the PMB group, two different scenarios for the end game were examined. On one hand, we investigated approaches in which we tried to install the side chain directly on **S13**. This would then require the reoxidation of the allylic alcohol towards a ketone to be able to exchange the PMB group as well as a re-reduction step to access to the natural product. On the other hand we examined strategies, which would allow the installation of the side chain with the acetyl group already installed on the aldehyde stage.

Scheme **S1** thereby shows initially investiagated unsuccessful vinylogous aldol reactions between **S13** and vinylketene silyl acetals **S26** and **S28**.

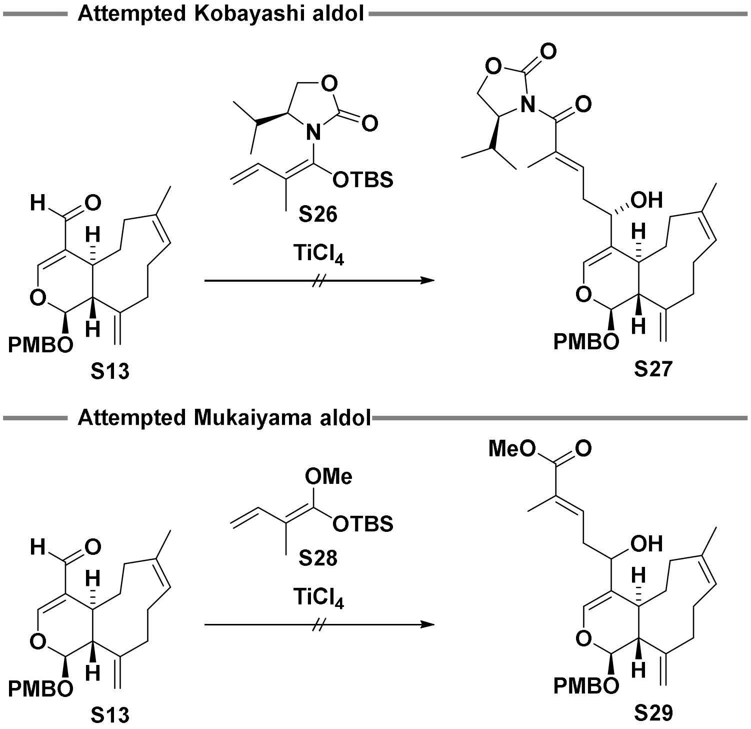

Scheme S1: Unsuccessful vinylogous aldol reactions with **S13**.

We then turned our attention towards strategies which involved methyl vinyl oxirane **S30** or building blocks derived from **S30** as side chain precursors (Figure **S2**). Unfortunately, the reductive opening of **S30** (SmI_2; Cp_2TiCl_2/Zn) and attempted direct 1,2-addition of the resulting carbanion into the aldehyde were thereby met with failure. Attempts to utilize deprotonated sulfones **S31** and **S32** were then thwarted by decomposition of the side chain precursors upon treatment with base. The introduction via 1,2 addition of **S33** was unsuccessful as complete γ-selectivity of the addition under Nozaki–Hiyama–Kishi conditions was observed. Applying alternative conditions, we were never able to observe the formation of the desired 1,2 addition product.

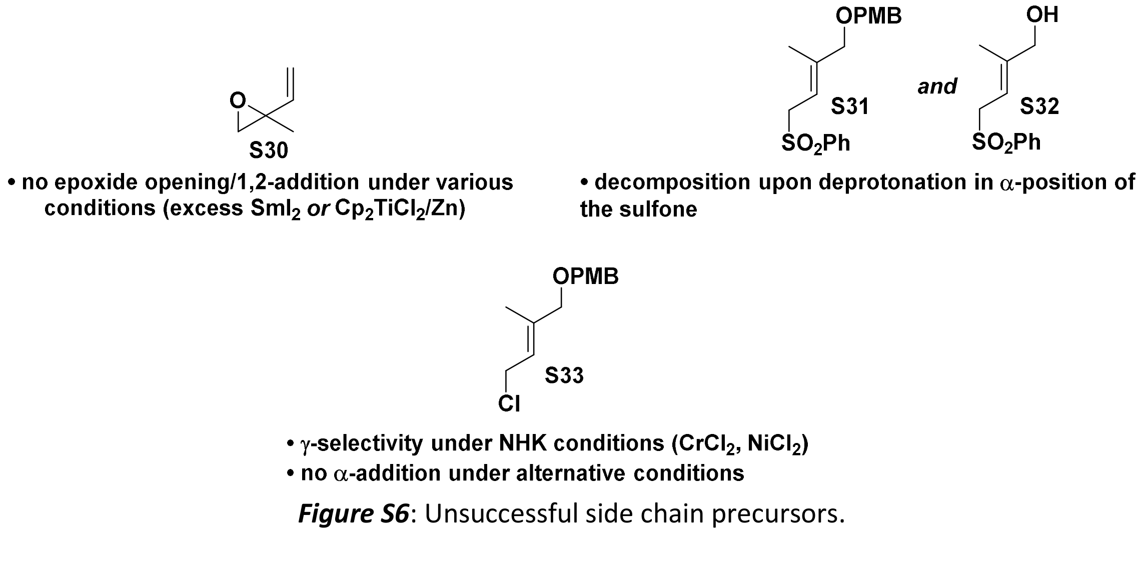

Figure S6: Unsuccessful side chain precursors.

Given these unsuccesful attempts, we ultimately found the allylation chemistry of boron compunds to be the most reliable to further build up the side chain. In addition it was compazible with the acetylated acetal, saving the oxidation/reduction steps otherwise necessary for the PMB removal.

Model substrate S24

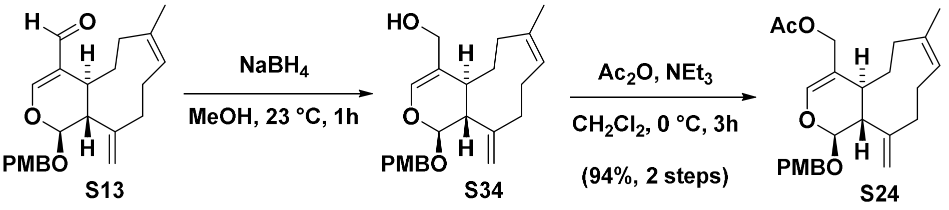

Sodium borohydride (1.1 mg, 30 µmol, 1.0 equiv) was added to a solution of **S13** (11 mg, 30 µmol, 1 equiv) in methanol (0.6 mL) at 23 °C. After 1 h, aqueous saturated ammonium chloride solution (10 mL) and dichloromethane (10 mL) were added to the reaction mixture. The layers were separated and the aqueous phase was extracted with dichloromethane (2 × 10 mL). The combined organic phases were dried over magnesium sulfate and the dried solution was filtrated. The filtrate was concentrated to give crude S34 (11 mg) which was used in the following step without further purification.

Triethylamine (17 µL, 0.12 mmol, 4.0 equiv) was added to a solution of crude **S34** (assumed pure: 11 mg, 30 µmol, 1 equiv) and acetic anhydride (8.5 µL, 85 µmol, 3.0 equiv) in dichloromethane (1 mL) at 23 °C. After 3 h, water (10 mL) and dichloromethane (10 mL) were added to the reaction mixture. The layers were separated, and the aqueous phase was extracted with dichloromethane (2 × 10 mL). The combined organic phases were dried over magnesium sulfate and the dried solution was filtrated. The filtrate was concentrated, and the residue was purified by flash column chromatography on silica gel (25% diethyl ether in pentane) to give **S24** (11.5 mg, 94%) as a colorless oil.

TLC (40% diethyl ether in pentane): R_f = 0.68 (UV, CAM).

^{1}H–NMR (400 MHz, $CDCl_3$): δ = 7.22 (d, *J* = 8.6 Hz, 2H), 6.86 (d, *J* = 8.7 Hz, 2H), 6.46 (s, 1H), 5.34 (t, *J* = 7.9 Hz, 1H), 4.92 (d, *J* = 10.5 Hz, 2H), 4.76 – 4.65 (m, 3H), 4.47 (d, *J* = 11.8 Hz, 1H), 4.35 (d, *J* = 12.3 Hz, 1H), 3.81 (s, 3H), 2.63 – 2.53 (m, 2H), 2.40 – 2.24 (m, 3H), 2.18 – 2.10 (m, 1H), 2.06 – 1.86 (m, 6H), 1.63 (s, 3H), 1.48 – 1.39 (m, 1H) ppm.

^{13}C–NMR (101 MHz, $CDCl_3$): δ = 171.2, 159.3, 151.6, 141.4, 136.9, 129.8, 129.4, 125.6, 113.9, 113.8, 113.3, 100.5, 69.8, 64.2, 55.4, 45.9, 35.5, 35.0, 28.5, 28.0, 27.8, 22.9, 21.3 ppm.

IR (Diamond–ATR, neat): 2935, 2854, 1663, 1453, 1374, 1227, 11769, 1014, 950, 789cm^{-1}.

HRMS (ESI) calc. for $C_{25}H_{32}NaO_5^+$ [M+Na]$^+$: 435.2142; found: 435.2143.

$[\alpha]_D^{20}$: 199.6 (c = 0.11, CH_2Cl_2).

Allylic chloride S35

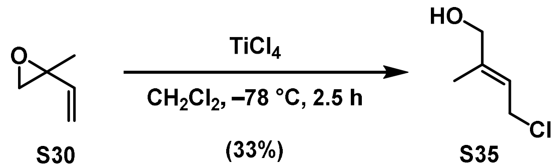

A solution of titanium tetrachloride (1.68 g, 15.2 mmol, 1.50 equiv) in dichloromethane (30 mL) was added over 20 mins via syringe pump to a solution of epoxide **S30** (854 mg, 10.2 mmol, 1 equiv) at –78 °C. After 2.5 h, aqueous hydrochloric acid (1M, 15 mL) was added and the reaction mixture was allowed to warm to 23 °C. The layers were separated and the aqueous phase was extracted with dichloromethane (2 × 20 mL). The combined organic phases were dried over magnesium sulfate and the dried solution was filtrated. The filtrate was concentrated and the residue was purified by flash column chromatography on silica gel (30% diethyl ether in pentane) to give chloride **S35** (398 mg, 22%) as a colorless oil.

TLC (15% ethyl acetate in cyclohexane): R_f = 0.53 (UV, Permanganate)
^{1}H-NMR (400 MHz, $CDCl_3$): δ = δ 5.75 (tq, 1H, J = 8.0, 1.5 Hz), 4.15 (d, 2H, J = 8.0 Hz), 4.09 (s, 2H), 1.74 (s, 3H) ppm.
The obtained analytical data were in full agreement with those reported in the literature.[7]

Sulfone S32

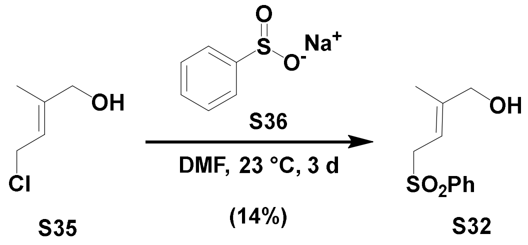

Chloride **S35** (137 mg, 1.14 mmol, 1 equiv) was added to a suspension of benzenesulfonic acid sodium salt **S36** (373 mg, 2.27 mmol, 2.00 equiv) in *N*,*N*-dimethylformamide (14.8 mL) at 23 °C. After 3 d, water (20 mL) and diethyl ether (10 mL) were added. The layers were separated and the aqueous phase was extracted with diethyl ether (2 × 10 mL) The combined organic phases were washed with 10% aqueous lithium chloride solution (10 mL), dried over magnesium sulfate and the dried solution was filtrated. The filtrate was concentrated and the residue was purified by flash column chromatography on silica gel (100% diethyl ether in pentane) to give sulfone **S32** (35.0 mg, 14%) as a yellowish oil.

TLC (50% diethyl ether in pentane): R_f = 0.10 (UV, CAM).
^{1}H-NMR (400 MHz, $CDCl_3$): δ = 7.90 – 7.87 (m, 2H), 7.68 – 7.62 (m, 1H), 7.58 – 7.52 (m, 2H), 5.52 (tq, *J* = 8.0, 1.4 Hz, 1H), 4.01 (d, *J* = 6.2 Hz, 2H), 3.86 (d, *J* = 8.0 Hz, 2H), 1.40 (s, 3H) ppm.
^{13}C-NMR (101 MHz, $CDCl_3$): δ = 145.5, 138.9, 133.9, 129.3, 128.6, 110.4, 67.6, 55.8, 13.8 ppm.
IR (Diamond-ATR, neat): 3496, 2920, 1447, 1302, 1148, 1131, 1084, 741, 689, 531 cm^{-1}.
HRMS (ESI) calc. for $C_{11}H_{14}NaO_3S^+$ $[M+Na]^+$: 249.0556; found: 249.0551.

Allylic chloride S33

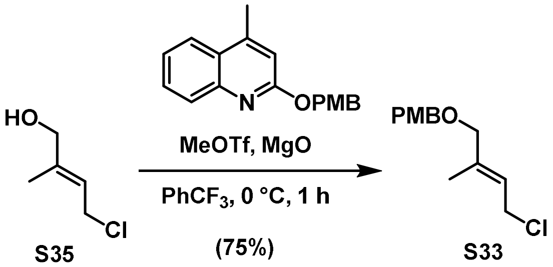

Methyl triflate (192 µL, 1.66 mmol, 2.00 equiv) was added to a suspension of alcohol **S35** (100 mg, 82.9 µmol, 1 equiv), 2-(4-Methoxybenzyloxy)-4-methylquinoline (478 mg, 1.66 mmol, 2.00 equiv) and magnesium oxide (68.9 mg, 1.66 mmol, 2.00 equiv) in benzotrifluoride (10 mL) at 0 °C. After 1 h, the reaction mixture was poured into a mixture of water (25 mL) and dichloromethane (25 mL). The layers were separated and the aqueous phase was extracted with dichloromethane (2 × 20 mL). The combined organic phases were dried over magnesium sulfate and the dried solution was filtrated. The filtrate was concentrated and the residue was purified by flash column chromatography on silica gel (10% diethyl ether in pentane) to give iodide **S33** (150 mg, 75%) as a colorless oil.

TLC (10% ethyl acetate in hexanes): R_f = 0.43 (UV, CAM).
^{1}H-NMR (400 MHz, $CDCl_3$): δ = 7.27 (d, *J* = 8.9 Hz, 2H), 6.88 (d, *J* = 8.7 Hz, 2H), 5.73 (tq, *J* = 7.9, 1.4 Hz, 1H), 4.41 (s, 2H), 4.13 (d, *J* = 7.9 Hz, 2H), 3.91 (s, 2H), 3.81 (s, 3H), 1.75 (s, 3H).
^{13}C-NMR (101 MHz, $CDCl_3$): δ = 159.4, 138.9, 130.4, 129.5, 122.5, 114.0, 74.6, 71.8, 55.4, 40.3, 13.9 ppm.
IR (Diamond-ATR, neat): 2933, 1612, 1512, 1246, 1173, 1108, 1075, 1035, 848, 820 cm^{-1}.
HRMS (ESI) calc. for $C_{13}H_{17}ClNaO_2^+$ $[M+Na]^+$: 263.0809; found: 263.0800.

Sulfone S31

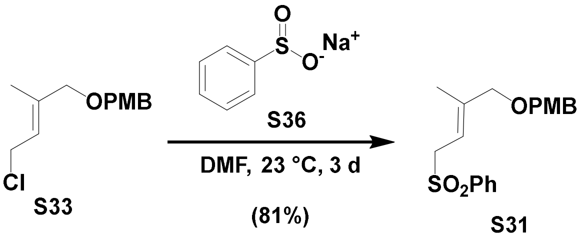

Chloride **S33** (560 mg, 2.33 mmol, 1 equiv) was added to a suspension of benzenesulfonic acid sodium salt **S36** (1.15 mg, 6.98 µmol, 3.00 equiv) in *N*,*N*-dimethylformamid (4.65 mL) at 23 °C. After 3 d, water (5 mL) and diethyl ether (5 mL) were added. The layers were separated and the aqueous phase was extracted with diethyl ether (2 × 5 mL) The combined organic phases were washed with 10% aqueous lithium chloride solution (10 mL), dried over magnesium sulfate and the dried solution was filtrated. The filtrate was concentrated and the residue was purified by flash column chromatography on silica gel (70% diethyl ether in pentane) to give sulfone **S31** (654 mg, 81%) as a yellowish oil.

TLC (70% ethyl acetate in hexanes): R_f = 0.48 (UV, CAM).
^{1}H-NMR (400 MHz, $CDCl_3$): δ = 7.88 (d, *J* = 7.1 Hz, 2H), 7.63 (t, *J* = 7.5 Hz, 1H), 7.52 (t, *J* = 7.7 Hz, 2H), 7.21 (d, *J* = 8.7 Hz, 2H), 6.88 (d, *J* = 8.7 Hz, 2H), 5.51 (tq, *J* = 7.9, 1.3 Hz, 1H), 4.33 (s, 2H), 3.87 (d, *J* = 8.0 Hz, 2H), 3.84 (s, 3H), 3.81 (s, 2H), 1.38 (s, 3H) ppm.
^{13}C-NMR (101 MHz, $CDCl_3$): δ = 159.4, 143.1, 138.8, 133.9, 130.2, 129.4, 129.2, 128.6, 113.9, 112.7, 74.3, 71.7, 55.8, 55.4, 14.0 ppm.
IR (Diamond-ATR, neat): 2924, 1513, 1405, 1305, 1247, 1151, 1084, 1033, 742, 532 cm^{-1}.
HRMS (ESI) calc. for $C_{19}H_{12}NaO_4S^+$ $[M+Na]^+$: 369.1131; found: 369.1125.

Allyl boronate 29

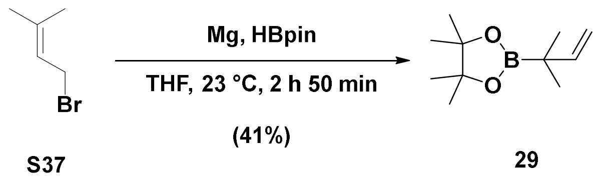

Pincolborane (2.93 mL, 20.0 mmol, 1 equiv) and 1 bromo-3-methyl-2-ene **S37** (4.75 mL, 40.5 mmol, 2.00 equiv) were added dropwise to a suspension of freshly activated magnesium turnings (590 mg, 24.3 mmol, 1.20 equiv) in tetrahydrofuran (30 mL) at 23 °C. After 50 min, additional pinacolatoborane (2.93 mL, 20.2 mmol, 1 equiv) was added to the reaction mixture. After 2.75 h, water (30 mL) and hexanes (15 mL) were added to the reaction mixture. After 5 min, aqueous hydrochloric acid (1 M, 10 mL) was added dropwise. The layers were separated, and the aqueous phase was extracted with hexanes (2 × 30 mL). The combined organic phases were dried over magnesium sulfate and the dried solution was filtrated. The filtrate was concentrated, and the residue was purified by flash column chromatography on silica gel (5% diethyl ether in pentane) to give boronate **29** (1.64 g, 41%) as a colorless oil.

^{1}H–NMR (400 MHz, $CDCl_3$): δ = 5.99 – 5.91 (m, 1H), 4.92 (s, 1H), 4.89 (d, *J* = 3.1 Hz, 1H), 1.22 (s, 12H), 1.06 (s, 6H) ppm.
The obtained analytical data were in full agreement with those reported in the literature.[8]

Alcohol S38a and S38b

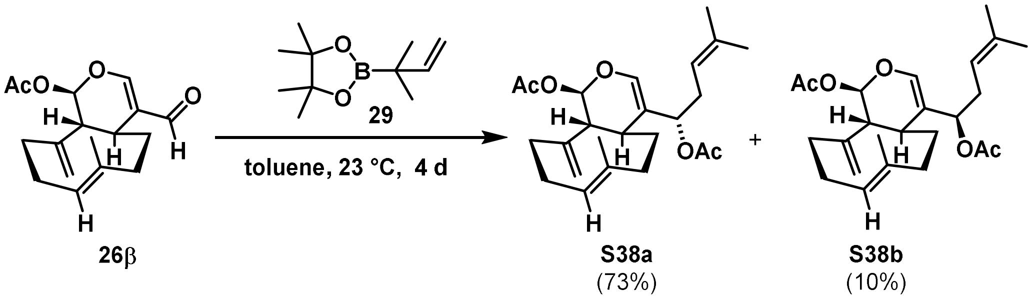

Pinacolboronate **29** (40.5 mg, 207 µmol, 6.00 equiv) was added to a solution of acetate **26β** (10.0 mg, 34.4 µmol, 1 equiv) in toluene (0.44 mL) at 23 °C. After 84 h, the reaction mixture was concentrated and the residue was purified by flash column chromatography on silica gel (8% diethyl ether in pentane) to give alcohol **S38b** (1.2 mg, 10%) as a colorless oil and alcohol **S38a** (9.1 mg, 73%) as a colorless oil.

S38a

TLC (40% diethyl ether in pentane): R_f = 0.17 (CAM).

^{1}H–NMR (400 MHz, $CDCl_3$): δ = 6.41 (d, *J* = 1.9 Hz, 1H), 5.89 (d, *J* = 1.8 Hz, 1H), 5.43 – 5.34 (m, 1H), 5.07 (dddd, *J* = 8.5, 5.7, 2.9, 1.5 Hz, 1H), 4.87 (s, 1H), 4.80 (d, *J* = 1.4 Hz, 1H), 4.11 (td, *J* = 7.2, 2.6 Hz, 1H), 2.52 – 2.36 (m, 2H), 2.33 – 2.15 (m, 6H), 2.16 – 2.03 (m, 5H), 1.97 – 1.95 (m, 1H), 1.71 (d, *J* = 1.4 Hz, 3H), 1.67 (s, 6H), 1.59 – 1.48 (m, 2H) ppm.

^{13}C–NMR (101 MHz, $CDCl_3$): δ = 167.0, 151.6, 138.6, 136.1, 134.9, 124.3, 120.0, 119.7, 113.2, 92.2, 73.6, 49.6, 40.2, 36.8, 35.6, 33.7, 31.1, 26.0, 25.2, 21.3, 18.4, 16.9 ppm.

IR (Diamond–ATR, neat): 3440, 2926, 2854, 1736, 1664, 1375, 1233, 1184, 1017, 949 cm^{-1}.

HRMS (ESI) calc. for $C_{22}H_{32}NaO_4^+$ $[M+Na]^+$: 383.2193; found: 383.2187.

$[\alpha]_D^{20}$: 72.8 (c = 0.18, CH_2Cl_2).

S38b

TLC (40% diethyl ether in pentane): R_f = 0.24 (CAM).

^{1}H–NMR (400 MHz, $CDCl_3$): δ = 6.53 – 6.52 (m, 1H), 5.87 (d, *J* = 2.2 Hz, 1H), 5.36 (t, *J* = 8.4 Hz, 1H), 5.18 – 5.13 (m, 1H), 4.89 (s, 1H), 4.86 (d, *J* = 1.3 Hz, 1H), 4.26 (dt, *J* = 8.1, 4.8 Hz, 1H), 2.53 – 2.42 (m, 1H), 2.42 – 2.33 (m, 1H), 2.31 – 2.18 (m, 4H), 2.16 – 2.08 (m, 1H), 2.08 – 1.92 (m, 6H), 1.82 – 1.75 (m, 1H), 1.75 – 1.74 (m, 3H), 1.68 – 1.66 (m, 6H), 1.61 – 1.50 (m, 2H) ppm.

^{13}C–NMR (101 MHz, $CDCl_3$): δ = 170.0, 151.5, 136.3, 136.1, 135.8, 124.7, 120.3, 119.4, 113.3, 92.2, 70.4, 49.9, 40.4, 38.1, 35.9, 35.7, 30.5, 26.2, 25.3, 21.3, 18.3, 16.9 ppm.

IR (Diamond–ATR, neat): 3443, 2925, 2855, 1736, 1672, 1376, 1231, 1160, 1017, 941 cm^{-1}.

HRMS (ESI) calc. for $C_{22}H_{32}NaO_4^+$ $[M+Na]^+$: 383.2193; found: 383.2183.

$[\alpha]_D^{20}$: 150.0 (c = 0.05, CH_2Cl_2).

9-deacetoxy-14,15-deepoxyxeniculin 30

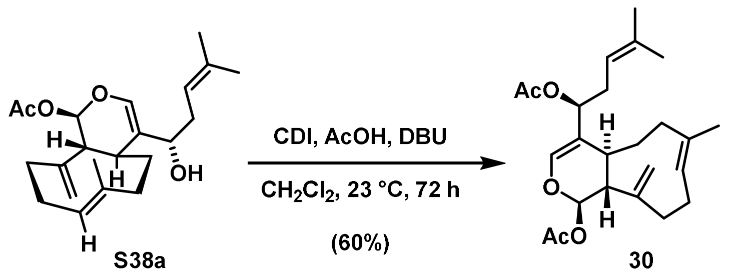

Acetic acid (40.9 µL, 714 µmol, 39.0 mmol) and 1,8-diazabicyclo[5.4.0]undec-7-ene (0.1 M stock solution in dichloromethane, 73.2 µL, 7.32 µmol, 0.400 equiv) were added sequentially to a solution of CDI (112 mg, 690 µmol, 37.6 equiv) in dichloromethane (0.5 mL) at 23 °C. After 20 min, a solution of alcohol **S38a** (6.6 mg, 18 µmol, 1 equiv) in dichloromethane (0.5 mL) was added at 23 °C. After 72 h, water (10 mL) and dichloromethane (10 mL) were added to the reaction mixture. The layers were separated, and the aqueous phase was extracted with dichloromethane (2 × 10 mL). The combined organic phases were dried over magnesium sulfate and the dried solution was filtrated. The filtrate was concentrated, and the residue was purified by flash column chromatography on silica gel (20% diethyl ether in pentane) to give 9-deacetoxy-14,15-deepoxyxeniculin **30** (4.4 mg, 60%) as an amorphous colorless solid.

TLC (40% diethyl ether in pentane): R_f = 0.58 (CAM).

^{1}H–NMR (400 MHz, $CDCl_3$): δ = 6.49 (d, *J* = 1.6 Hz, 1H), 5.87 (d, *J* = 1.4 Hz, 1H), 5.36 (t, *J* = 8.4 Hz, 1H), 5.28 (t, *J* = 7.5 Hz, 1H), 4.98 (t, *J* = 6.5 Hz, 1H), 4.87 (s, 1H), 4.78 (s, 1H), 2.52 – 2.38 (m, 2H), 2.37 – 1.94 (m, 9H), 2.05 (s, 3H), 2.03 (s, 3H), 1.68 (s, 3H), 1.66 (s, 3H), 1.64 (s, 3H), 1.54 (d, *J* = 0.8 Hz, 1H) ppm.

^{13}C–NMR (101 MHz, $CDCl_3$): δ = 170.5, 169.8, 151.3, 140.8, 136.0, 134.6, 124.5, 119.0, 116.1, 113.3, 91.9, 75.1, 49.4, 40.2, 36.9, 35.6, 31.3, 30.7, 25.9, 25.2, 21.7, 21.2, 18.3, 16.9 ppm.

IR (Diamond–ATR, neat): 2927, 2854, 1736, 1664, 1453, 1372, 1239, 1151, 1015, 950 cm^{-1}.

HRMS (ESI) calc. for $C_{24}H_{34}NaO_5^+$ $[M+Na]^+$: 425.2298; found: 425.2287.

$[\alpha]_D^{20}$: 35.6 (c = 0.16, CH_2Cl_2).

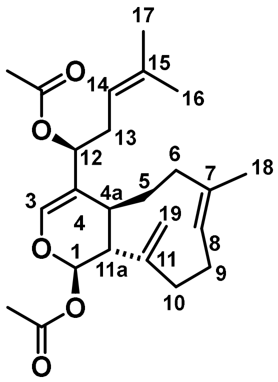

9-deacetoxy-14,15-deepoxyxeniculin

Table S6: *Comparison of ^{1}H–NMR shifts for natural and synthetic 9-deacetoxy-14,15-deepoxyxeniculin (**30**).*[9]

No	^{1}H–NMR (400 MHz, $CDCl_3$) isolated 9-deacetoxy-14,15-deepoxyxeniculin **ppm**	^{1}H–NMR (400 MHz, $CDCl_3$) synthetic 9-deacetoxy-14,15-deepoxyxeniculin **ppm**	Δ ppm
H-1	5.87, d	5.87 (d, *J* = 1.4 Hz, 1H)	±0
H-3	6.49, d	6.49 (d, *J* = 1.6 Hz, 1H)	±0
H-8	5.36, br t	5.36 (t, *J* = 8.4 Hz, 1H)	±0
H-12	5.28 t	5.28 (t, *J* = 7.5 Hz, 1H)	±0
H-14	4.99, br t	4.98 (t, *J* = 6.5 Hz, 1H)	–0.01
CH_3-16	1.68, s	1.68 (s, 3H)	±0
CH_3-17	1.66, s	1.66 (s, 3H)	±0
CH_3-18	1.65, s	1.64 (s, 3H)	–0.01
H-19	4.87, s	4.87 (s, 1H)	±0
H-19’	4.79, s	4.78 (s, 1H)	+0.1
OAc	2.05, s; 2.03, s	2.05, s; 2.03, s	±0; ±0

Table S7: *Comparison of ^{13}C–NMR shifts for natural and synthetic 9-deacetoxy-14,15-deepoxyxeniculin (**30**).*[9]

No	^{13}C–NMR (101 MHz, $CDCl_3$) isolated 9-deacetoxy-14,15-deepoxyxeniculin **ppm**	^{13}C–NMR (176 MHz, $CDCl_3$) synthetic 9-deacetoxy-14,15-deepoxyxeniculin **ppm**	Δ ppm
C-1	91.8	91.9	+0.1
C-3	140.7	140.8	+0.1
C-4	115.9	116.1	+0.2
C-4a	36.7	36.9	+0.2
C-5	30.5	30.7	+0.2
C-6	40.0	40.2	+0.2
C-7	134.3	134.6	+0.3
C-8	124.3	124.5	+0.2
C-9	25.0	25.2	+0.2
C-10	35.4	35.6	+0.2
C-11	151.2	151.3	+0.1
C-11a	49.3	49.4	+0.1
C-12	74.9	75.1	+0.2
C-13	31.3	31.3	±0
C-14	119.0	119.0	±0

C-15	135.8	136.0	+0.2
C-16	18.1	18.3	+0.2
C-17	25.7	25.9	+0.2
C-18	16.7	16.9	+0.2
C-19	113.1	113.3	+0.2
OAc	170.2, 169.6, 21.3, 20.9	170.5, 169.8, 21.7, 21.2	+0.3, +0.2, +0.4, +0.3

Xeniafaraunol A 31

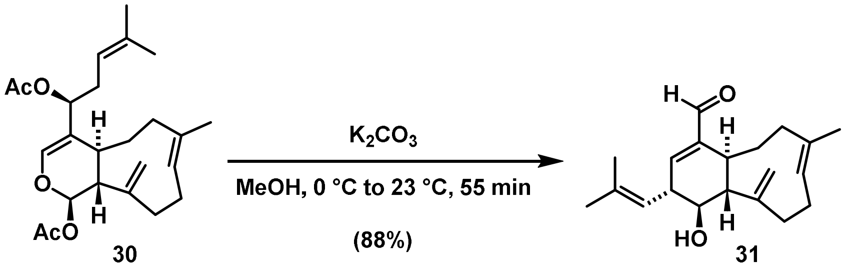

Potassium carbonate (4.0 mg, 28 µmol, 6.0 equiv) was added to a solution of **30** (1.9 mg, 4.7 µmol, 1 equiv) in methanol (0.6 mL) at 0 °C. After 45 min, the reaction mixture was allowed to warm to 23 °C and started to turn yellow. After 10 min, aqueous saturated ammonium chloride solution (10 mL) and dichloromethane (10 mL) were added to the reaction mixture. The layers were separated and the aqueous phase was extracted with dichloromethane (2 × 10 mL). The combined organic phases were dried over magnesium sulfate and the dried solution was filtrated. The filtrate was concentrated and the residue was purified by flash column chromatography on silica gel (20% diethyl ether in pentane) to give xeniafaraunol A **31** (1.2 mg, 88%) as a colorless oil.

TLC (40% diethyl ether in pentane): R_f = 0.51 (UV, CAM).

^{1}H–NMR (700 MHz, C_6D_6): δ = 9.26 (s, 1H), 5.91 (s, 1H), 5.39 (dd, *J* = 11.5, 5.0 Hz, 1H), 4.94 (d, *J* = 8.8 Hz, 1H), 4.82 (s, 1H), 4.79 (s, 1H), 3.08 – 3.03 (m, 2H), 2.82 – 2.79 (m, 1H), 2.72 (td, *J* = 12.4, 3.4 Hz, 1H), 2.30 (tdd, *J* = 11.7, 10.0, 5.4 Hz, 1H), 2.12 (dt, *J* = 12.0, 3.2 Hz, 1H), 2.06 (dt, *J* = 12.9, 4.8 Hz, 1H), 1.91 – 1.84 (m, 2H), 1.69 – 1.62 (m, 2H), 1.67 (d, *J* = 1.5 Hz, 3H), 1.60 (d, *J* = 1.4 Hz, 3H), 1.56 (d, *J* = 1.8 Hz, 1H), 1.54 (s, 3H), 1.23 – 1.18 (m, 1H) ppm.

^{13}C–NMR (176 MHz, C_6D_6): δ = 192.9, 150.0, 146.9, 146.0 (assigned by HMBC), 135.9, 135.6, 125.6, 124.9, 118.0, 73.3, 61.0, 44.0, 40.7, 36.6, 36.3, 32.2, 28.5, 26.0, 18.5, 18.5 ppm.

IR (Diamond–ATR, neat): 3451, 2923, 2855, 1686, 1449, 1260, 1086, 1057, 1023, 801 cm^{-1}.

HRMS (ESI) calc. for $C_{20}H_{28}NaO_2^+$ $[M+Na]^+$: 323.1982; found: 323.1977.

$[\alpha]_D^{20}$: –127.5 (c = 0.08, CH_2Cl_2).

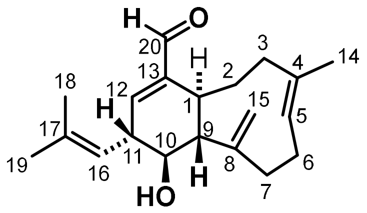

Xeniafaraunol A

Table S8: *Comparison of* [1]*H–NMR shifts for natural and synthetic xeniafaraunol A (**31**).*[10]

No	^{1}H–NMR (500 MHz, C_6D_6)<br>isolated xeniafaraunol A<br>**ppm**	^{1}H–NMR (700 MHz, C_6D_6)<br>synthetic xeniafaraunol A<br>**ppm**	Δ ppm
H-1	2.80 (m, 1H)	2.82 – 2.79 (m, 1H)	±0
H-2	1.20 (ddt, *J* = 3, 1.5, 13.5 Hz, 1H)	1.23 – 1.18 (m, 1H)	±0
H-2’	1.65 (dq, *J* = 13.5, 3.0 Hz, 1H)	1.69 – 1.62 (m, 2H), *1.67 by HSQC*	+0.02
H-3	2.10 (dt, *J* = 12.0, 3.0 Hz, 1H)	2.12 (dt, *J* = 12.0, 3.2 Hz, 1H)	+0.02
H-3’	2.72 (dt, *J* = 3.5, 12.0 Hz, 1H)	2.72 (td, *J* = 12.4, 3.4 Hz, 1H)	±0
H-4	-	-	-
H-5	5.40 (dd, *J* = 11.0, 4.5 Hz, 1H)	5.39 (dd, *J* = 11.5, 5.0 Hz, 1H)	–0.01
H-6	1.88 (dq, *J* = 12.5, 5.0 Hz, 1H)	1.91 – 1.84 (m, 2H), *1.89 by HSQC*	+0.01
H-6’	2.30 (dq, *J* = 5.5, 12.0 Hz, 1H)	2.30 (tdd, *J* = 11.7, 10.0, 5.4 Hz, 1H)	±0
H-7	1.62 (dd, *J* = 13.0, 10.0 Hz, 1H)	1.69 – 1.62 (m, 2H), *1.64 by HSQC*	+0.02
H-7’	2.06 (dt, *J* = 4.7, 13.0 Hz, 1H)	2.06 (dt, *J* = 12.9, 4.8 Hz, 1H)	±0
H-8	-	-	-
H-9	1.87 (t, *J* = 9.0 Hz, 1H)	1.91 – 1.84 (m, 2H), *1.87 by HSQC*	±0
H-10	3.03 (t, *J* = 9.0 Hz, 1H)	3.08 – 3.03 (m, 2H), *3.03 by HSQC*	±0
H-11	3.06 (m, 1H)	3.08 – 3.03 (m, 2H), *3.05 by HSQC*	–0.01
H-12	5.90 (s, 1H)	5.91 (s, 1H)	+0.01
H-13	-	-	-
H-14	1.53 (s, 3H)	1.54 (s, 3H)	+0.01
H-15	4.80 (s, 2H)	4.82 (s, 1H), 4.79 (s, 1H)	-
H-16	4.94 (d, *J* = 7.5 Hz 1H)	4.94 (d, *J* = 8.8 Hz, 1H)	±0
H-17	-	-	-
H-18	1.60 (s, 3H)	1.60 (d, *J* = 1.4 Hz, 3H)	±0
H-19	1.67 (s, 3H)	1.67 (d, *J* = 1.5 Hz, 3H)	±0
H-20	9.26 (s, 1H)	9.26 (s, 1H)	±0

Table S9: *Comparison of* [13]*C–NMR shifts for natural and synthetic xeniafaraunol A (**31**).*[10]

No	^{13}C–NMR (125 MHz, C_6D_6)<br>isolated xeniafaraunol A<br>**ppm**	^{13}C–NMR (176 MHz, C_6D_6)<br>synthetic xeniafaraunol A<br>**ppm**	Δ ppm
C-1	36.6	36.6	±0
C-3	36.3	36.3	±0
C-4	40.6	40.7	+0.1
C-4a	136.0	135.9	–0.1
C-5	125.1	124.9	–0.2

C-6	28.5	28.5	±0
C-7	32.2	32.2	±0
C-8	146.9	146.9	±0
C-9	61.0	61.0	±0
C-10	73.3	73.3	±0
C-11	44.0	44.0	±0
C-12	150.1	150.0	–0.1
C-13	146.0	146.0 (assigned by HMBC)	±0
C-14	18.4	18.5	+0.1
C-15	118.0	118.0	±0
C-16	125.6	125.6	±0
C-17	135.6	135.6	±0
C-18	18.5	18.5	±0
C-19	26.0	26.0	±0
C-20	192.0	192.9	+0.9

3 Computational Studies

3.1 Computational Methodology

All calculations were carried out with the Gaussian 16 package.[11] Investigated structures were fully optimized in implicit solvent with the B3LYP hybrid density functional[12–15] and 6-311G++(2d,2p)[16] basis set on all atoms. Bulk solvent effects were (implicitly) modelled by Integral Equation Formalism Polarizable Continuum Model (IEFPCM) of Tomasi and Pascual-Ahuir[17–19] as implemented in Gaussian 16. The internally stored parameters for toluene (ε = 2.3741) and tetrahydrofuran (ε = 7.4257) were used. Empirical dispersion correction of the D3-generation with Becke-Johnson damping[20] were added as implemented in Gaussian 16. Frequency analyses were carried out at the same level as the geometry optimizations and the nature of the stationary points was determined by analyses of the Hessian matrix. (Local) energy minima were confirmed to show only real eigenvalues, whereas transition states were confirmed to have one imaginary eigenvalue, while the corresponding eigenvector coincided with the change in the reaction coordinate. Zero-point energy and thermal corrections were calculated using the standard rigid-rotator/harmonic oscillator model to obtain Gibbs free energies at the indicated temperatures, no scaling of the frequencies was applied. The possibility of different conformations was considered for all structures. Structures were visualized by using CylView.[21]

3.2 Alkene isomerization

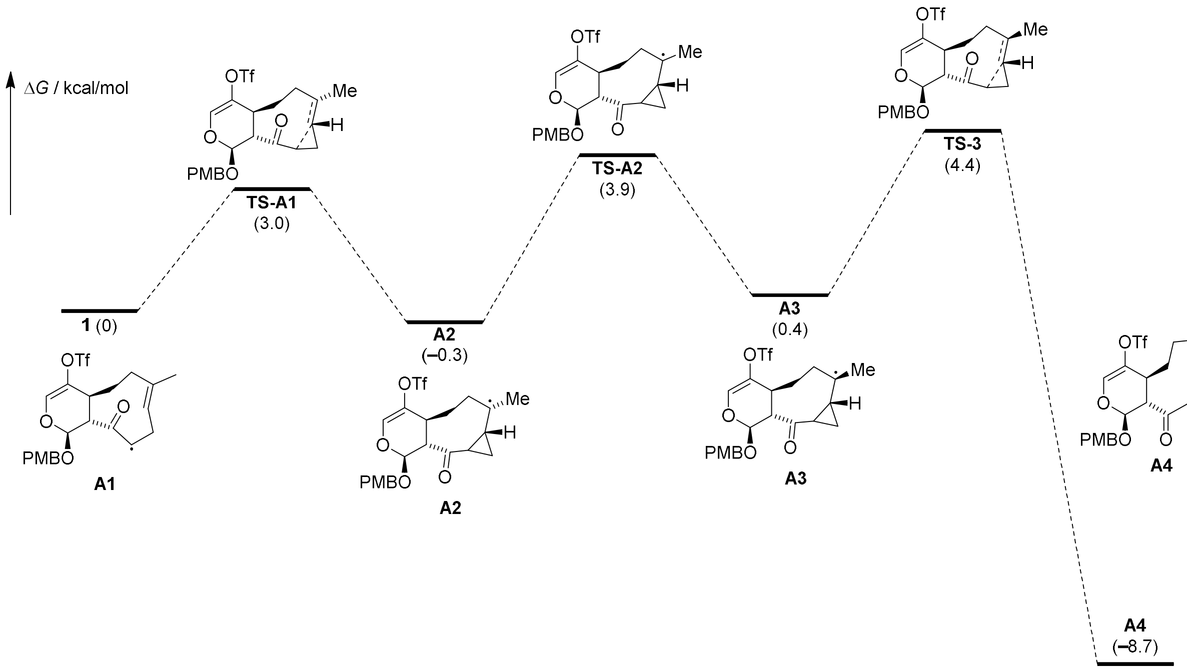

Scheme S2: Calculated pathway of the isomerization of the C7/C8-alkene.

Proposed reaction pathway for the (E)/(*Z*)-isoimerization of the C7/C8 alkene unit as calculated with B3LYP-D3/6-311++G(2d,2p) in toluene treated as the implicit solvent. Relative Gibbs free energies at 381.15 K are given in kcal/mol, whereas the energy of the α-keto radical **A1** was arbitrarily set to zero.

3.3 Nine-membered ring conformations

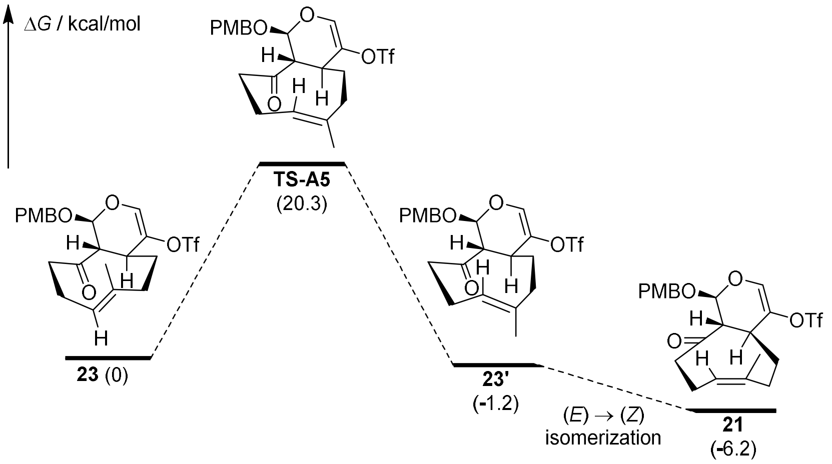

Scheme S3: Calculated barrier for the interconversion of conformers **23** and **23'** and comparison with the relative energy of **21**containing the isomerized alkene.

The proposed reaction pathway for the interconversion between the nine-membered ring conformations **23** and **23'** as well as comparison of closed-shell nine-membered ring conformations bearing either an (*E*) (i.e., **23** and **23'**) or a (*Z*) (i.e., **21**) double bond geometry is depicted. In accordance with the proposed alkene isomerization pathway, the closed-shell compound **21** is thermodynamically favored over the (*E*)-conformers **23** and **23'**. All calculations were performed with B3LYP-D3/6-311++G(2d,2p) in tetrahydrofuran treated as the implicit solvent. Relative Gibbs free energies at 298.15 K are given in kcal/mol, whereas the energy of conformer **23** was arbitrarily set to zero.

3.4 Cartesian Coordinates

α-Keto radical A1

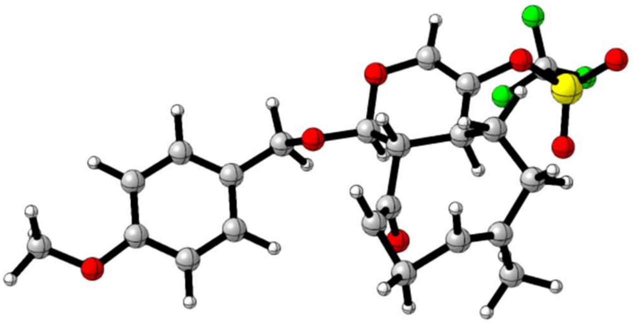

IEFPCM(toluene)B3LYP-D3/6-311++G(2d,2p) Electronic Energy = –2077.582853

IEFPCM(toluene)B3LYP-D3/6-311++G(2d,2p) Free Energy = –2077.243302 (T = 381.15 K)

Number of imaginary frequencies = 0

S	4.50274000	-0.62540700	0.13591800
O	-0.68002100	1.56788400	1.26672100
O	0.12216400	-1.73465300	-1.25890700
O	-1.86076900	-0.74038900	-0.70461500
O	-7.95753700	-0.16710800	0.57332500
O	3.55400900	-0.61737100	-1.16016200
O	3.97262400	0.20121000	1.18363500
O	5.83606600	-0.48767900	-0.36732000
C	-0.69248800	1.72049800	0.04547900
C	-1.20904600	2.91130900	-0.58585700
H	-1.41608000	2.87416000	-1.64687200
C	-0.93773300	4.24544600	0.03980200
H	-1.54335600	5.04729100	-0.37309500
H	-1.03715300	4.21964700	1.11998700
C	0.50413900	4.26501900	-0.42929500
H	0.61585200	4.42021700	-1.49732700
C	1.55635300	3.74326000	0.23529200
C	2.69597300	3.15858000	-0.57039100
H	3.50194300	2.84431500	0.09209200
H	3.11486000	3.90063800	-1.25328200
C	2.22316600	1.95342800	-1.41867300
H	3.08601700	1.56718000	-1.95754100
H	1.52538300	2.30766700	-2.17590900
C	1.54920100	0.79025000	-0.62472500
H	1.71657700	0.94759200	0.43943400
C	0.00594500	0.68509800	-0.83909700
H	-0.22557300	0.85211300	-1.89013400
C	-0.50852600	-0.69733500	-0.46358100
H	-0.27634000	-0.92050400	0.58170400
C	2.14050000	-0.53649700	-0.99691300
C	1.46672000	-1.63898800	-1.31590300
H	1.95547400	-2.53936900	-1.65656500
C	1.61352800	3.55358200	1.71888100
H	0.76084800	3.98397400	2.23481500
H	2.52446600	4.02106400	2.10264200
H	1.67117500	2.49718300	1.98646000
C	-2.57315600	-1.79930500	-0.03284900
H	-2.11411400	-1.94894200	0.94826900
H	-2.47718500	-2.72359700	-0.60271200
C	-4.00947600	-1.39747500	0.10397900
C	-4.35303500	-0.26286300	0.84883000
H	-3.57335000	0.32372700	1.31586200
C	-5.67205600	0.12308600	0.98524700
H	-5.94232100	0.99825100	1.55831400
C	-6.68871700	-0.62454300	0.37941100
C	-6.36368600	-1.75596000	-0.36444800

H	-7.12720300	-2.34676900	-0.84417700
C	-5.02543200	-2.12622500	-0.49731500
H	-4.77973000	-3.00118000	-1.08461000
C	-9.02723500	-0.89413900	-0.02023800
H	-8.93569900	-0.91303400	-1.10778300
H	-9.93490800	-0.36803900	0.25767000
H	-9.06786000	-1.91652400	0.35998100
F	5.29563400	-2.65533900	1.59061900
F	4.43838400	-3.23611900	-0.31884700
F	3.14621600	-2.59123700	1.30302000
C	4.32506800	-2.40813400	0.7162870

Transition state TS-A1

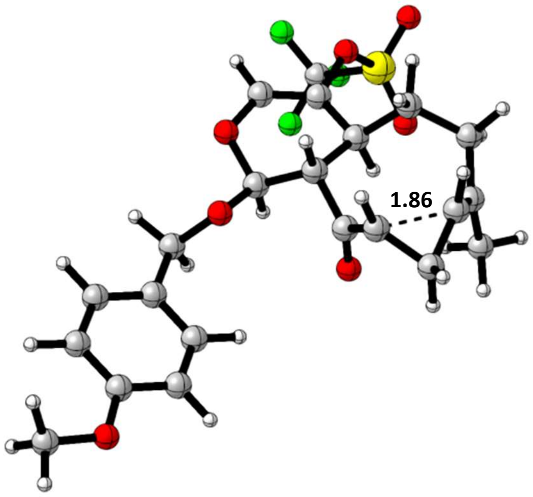

IEFPCM(toluene)B3LYP-D3/6-311++G(2d,2p) Electronic Energy = –2077.578391

IEFPCM(toluene)B3LYP-D3/6-311++G(2d,2p) Free Energy = –2077.238455 (T = 381.15 K)

Number of imaginary frequencies = 1 (423.25i cm^{-1})

S	4.55135800	-0.57222900	0.09216900	H	3.09364400	1.60772300	-1.86706500
O	-0.75275700	1.58016600	1.21414200	H	1.50901000	2.26366500	-2.16039500
O	0.15420800	-1.79925400	-1.16436400	C	1.54324200	0.75979700	-0.59849600
O	-1.84672200	-0.80743300	-0.67474300	H	1.69507200	0.91662800	0.46936400
O	-7.95757700	-0.18999600	0.51056500	C	0.00789400	0.63118100	-0.83442300
O	3.56603200	-0.61450700	-1.17511000	H	-0.20393000	0.75539500	-1.89577400
O	4.03234800	0.26531700	1.13681700	C	-0.49912200	-0.73882400	-0.41455600
O	5.86653800	-0.41570600	-0.45208600	H	-0.28176600	-0.92283200	0.64097500
C	-0.69670900	1.70368300	-0.00817500	C	2.15628100	-0.55596100	-0.97300900
C	-1.08206000	2.90739600	-0.70185500	C	1.49426500	-1.67669300	-1.25211000
H	-1.27147700	2.85358900	-1.76185500	H	1.99215700	-2.57460100	-1.58612200
C	-1.13096100	4.22428000	-0.00820100	C	1.44112000	3.70048900	1.75268900
H	-1.75104700	4.98321600	-0.46844300	H	0.58562900	4.18856900	2.21128300
H	-1.27356000	4.15707100	1.06221400	H	2.35196000	4.18800100	2.11336800
C	0.28444900	4.14974200	-0.47706900	H	1.47400700	2.67138000	2.11981800
H	0.45470600	4.43382700	-1.50745700	C	-2.56310800	-1.83499200	0.03789700
C	1.40305500	3.75076000	0.26171300	H	-2.11507100	-1.93689800	1.03021600
C	2.56650200	3.16682700	-0.49664500	H	-2.45940100	-2.78553700	-0.48588500
H	3.34915000	2.87371800	0.20267600	C	-4.00212200	-1.43086400	0.13988500
H	3.00492000	3.91800800	-1.16138600	C	-4.35291300	-0.24720000	0.80035400
C	2.18770800	1.94725900	-1.36956300	H	-3.57670400	0.37540400	1.22450700

C	-5.67432300	0.14049400	0.90567200
H	-5.95044600	1.05339300	1.41341800
C	-6.68575000	-0.65389800	0.35298000
C	-6.35328700	-1.83394700	-0.30717800
H	-7.11252200	-2.46113400	-0.74584000
C	-5.01272800	-2.20591000	-0.41011200
H	-4.76114600	-3.11920200	-0.93308300
C	-9.02163400	-0.96281900	-0.03282900
H	-8.92567000	-1.05952400	-1.11584700
H	-9.93280900	-0.42246200	0.20318700
H	-9.05924200	-1.95547500	0.41970900
F	5.43347700	-2.55080300	1.56669900
F	4.53465900	-3.19342000	-0.30342000
F	3.27560100	-2.54130900	1.34147400
C	4.43230200	-2.34499600	0.71622400

Cyclopropane A2

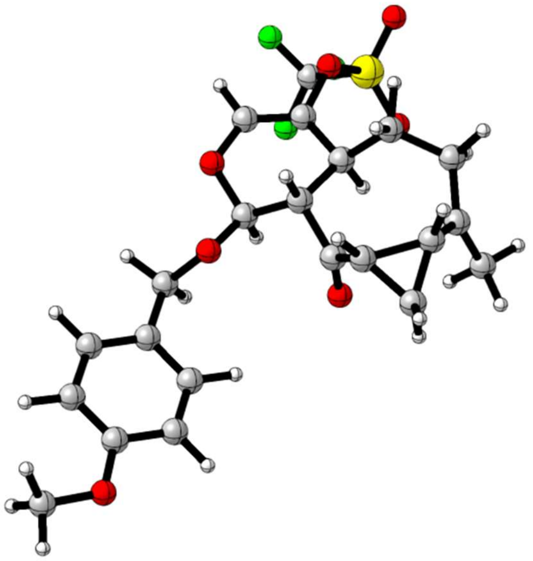

IEFPCM(toluene)B3LYP-D3/6-311++G(2d,2p) Electronic Energy = –2077.582599

IEFPCM(toluene)B3LYP-D3/6-311++G(2d,2p) Free Energy = –2077.243785 (T = 381.15 K)

Number of imaginary frequencies = 0

S	4.59206500	-0.54877500	0.07547500	H	2.93956700	3.92829400	-1.09134600
O	-0.84914500	1.57223000	1.18336800	C	2.17274200	1.94927800	-1.32560700
O	0.18527700	-1.82763100	-1.09676800	H	3.11508700	1.64671900	-1.77674100
O	-1.82209600	-0.83933200	-0.62650500	H	1.51747500	2.22310800	-2.15212600
O	-7.94934200	-0.25417500	0.47832900	C	1.55573600	0.74738200	-0.56316700
O	3.58379400	-0.60615000	-1.17413700	H	1.70874400	0.90613100	0.50533400
O	4.07990300	0.28347700	1.12745100	C	0.02760200	0.60141200	-0.80484900
O	5.89469800	-0.37668300	-0.49327700	H	-0.17480000	0.70282200	-1.87047700
C	-0.69546600	1.68780300	-0.01853600	C	-0.47548200	-0.76292100	-0.36252700
C	-1.01407600	2.91729900	-0.76615400	H	-0.26120800	-0.93149700	0.69616600
H	-1.36064600	2.78333200	-1.77989300	C	2.17881400	-0.56174600	-0.94569000
C	-1.30650500	4.18919600	-0.03513600	C	1.52292700	-1.69052300	-1.20663800
H	-1.97237300	4.89185800	-0.51321800	H	2.02351700	-2.58523800	-1.54480800
H	-1.42823300	4.09659100	1.03374500	C	1.29169100	4.03966200	1.70342900
C	0.07659300	4.04432400	-0.58520600	H	0.59730600	4.82720000	1.99528700
H	0.26597100	4.53676300	-1.53404600	H	2.29282300	4.32600700	2.03200800
C	1.26702900	3.79303100	0.23344700	H	1.01555200	3.14395300	2.27414200
C	2.45742900	3.18244800	-0.44379700	C	-2.53741500	-1.86228200	0.09592800
H	3.20093800	2.90896800	0.30572700	H	-2.09845100	-1.94362500	1.09406900

H	-2.41922900	-2.81958200	-0.41198300
C	-3.98043300	-1.46915100	0.17596900
C	-4.35108000	-0.29185900	0.83699300
H	-3.58721300	0.33265600	1.28034900
C	-5.67648100	0.08752900	0.91930200
H	-5.96784300	0.99538800	1.42751700
C	-6.67227000	-0.70936700	0.34224400
C	-6.32005100	-1.88343600	-0.31855800
H	-7.06703100	-2.51251900	-0.77516600
C	-4.97569300	-2.24689200	-0.39792000
H	-4.70861900	-3.15562500	-0.92107900
C	-8.99840100	-1.02971600	-0.09017800
H	-8.88120500	-1.11865700	-1.17173200
H	-9.91701400	-0.49617700	0.13198000
H	-9.03856000	-2.02544300	0.35524700
F	5.52710100	-2.51464900	1.53453900
F	4.59905900	-3.17094300	-0.31641900
F	3.36518400	-2.53120000	1.35217100
C	4.50705700	-2.32210500	0.70384000

Transition state TS-A2

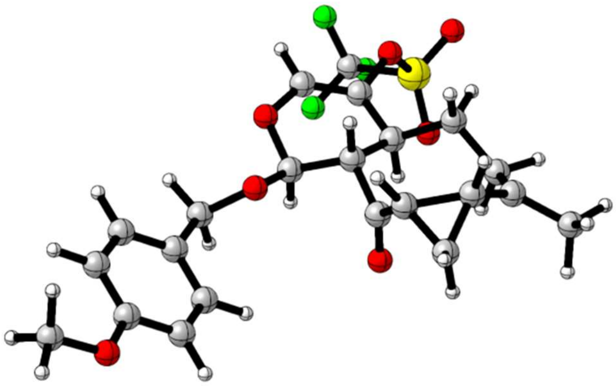

IEFPCM(toluene)B3LYP-D3/6-311++G(2d,2p) Electronic Energy = –2077.575938

IEFPCM(toluene)B3LYP-D3/6-311++G(2d,2p) Free Energy = –2077.237081 (T = 381.15 K)

Number of imaginary frequencies = 1 (43.30i cm^{-1})

S	4.67158500	-0.63616500	0.07559900	H	1.77191400	2.24068000	-1.74270900
O	-0.68298300	1.73190700	1.47381700	C	1.58914600	0.71925900	-0.20865300
O	0.18872000	-1.79236400	-0.86228100	H	1.72880900	0.72594400	0.87404100
O	-1.80441900	-0.79297400	-0.34464100	C	0.07688700	0.61644200	-0.49007500
O	-8.01486200	-0.55434200	0.31003800	H	-0.08982300	0.72774000	-1.56109900
O	3.57790400	-0.54744200	-1.10083800	C	-0.45484100	-0.74684000	-0.08412100
O	4.19832100	-0.01005300	1.27766500	H	-0.23980100	-0.95736900	0.96760300
O	5.92364100	-0.31126400	-0.53831100	C	2.19636700	-0.53384500	-0.76480800
C	-0.64336100	1.72507100	0.25971900	C	1.51590000	-1.64025200	-1.05989600
C	-1.17108900	2.80845100	-0.59947400	H	1.98263100	-2.50675400	-1.50307200
H	-1.66034000	2.45591100	-1.49658100	C	1.22824200	5.65802600	0.40924600
C	-1.59133700	4.11634800	-0.01086600	H	0.67701800	6.40788700	-0.16082400
H	-2.43515800	4.62213700	-0.45549300	H	2.28498500	5.93077200	0.40892200
H	-1.51058000	4.19622100	1.06300300	H	0.88062500	5.73959200	1.44902700
C	-0.29455400	4.04954100	-0.79157200	C	-2.51379000	-1.87571100	0.29535600
H	-0.37356300	4.46265500	-1.79490700	H	-2.14079000	-1.96665900	1.31933400
C	1.01030800	4.28288000	-0.13493300	H	-2.30921900	-2.80710600	-0.23228100
C	2.04025300	3.24286200	0.18473100	C	-3.97665500	-1.55942000	0.28089000
H	1.88970800	2.86924000	1.20797800	C	-4.46956000	-0.47352300	1.01382500
H	3.00261100	3.75710600	0.22900100	H	-3.78583200	0.13422100	1.59116700
C	2.19418200	2.02483800	-0.76020900	C	-5.81493300	-0.16141000	1.00289000
H	3.25750000	1.86709200	-0.91841800	H	-6.19980800	0.67590100	1.56686600

C	-6.70890000	-0.93725900	0.25578500
C	-6.23503100	-2.02199000	-0.47825600
H	-6.90233000	-2.63381300	-1.06359400
C	-4.87230800	-2.31731500	-0.46025200
H	-4.51011300	-3.15648200	-1.03930700
C	-8.96430900	-1.31055300	-0.43329800
H	-8.74087700	-1.28271500	-1.50127100
H	-9.92590900	-0.84128300	-0.25193900
H	-8.99518100	-2.34742200	-0.09377500
F	5.77962900	-2.76104700	1.13831400
F	4.76783200	-3.15493600	-0.74287100
F	3.61295200	-2.85984000	1.07204900
C	4.70336100	-2.48896800	0.40692400

Cyclopropane A3

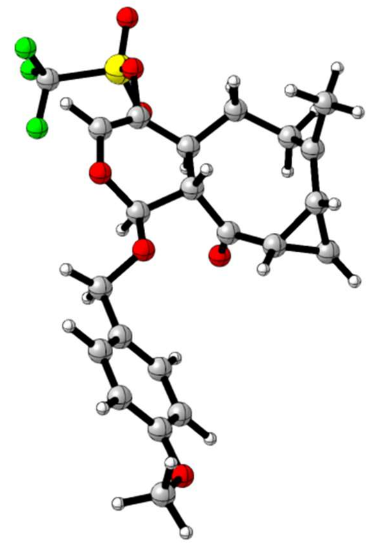

IEFPCM(toluene)B3LYP-D3/6-311++G(2d,2p) Electronic Energy = –2077.580616

IEFPCM(toluene)B3LYP-D3/6-311++G(2d,2p) Free Energy = –2077.242601 (T = 381.15 K)

Number of imaginary frequencies = 0

S	4.72280300	-0.45586600	0.30289400	H	2.20364400	4.32868400	0.30379100
O	-0.49374400	1.19143900	2.05360400	C	2.23650900	2.28241500	-0.31139900
O	0.35311000	-1.39501100	-1.26175200	H	3.25915500	2.31377300	0.06104600
O	-1.67491900	-0.68046000	-0.47783200	H	2.30258800	2.35737400	-1.39760100
O	-7.84082000	-0.75924500	0.46617900	C	1.64038600	0.91403700	0.06995800
O	3.71296200	-0.11323100	-0.89923300	H	1.78175500	0.76897100	1.14207900
O	4.15979200	-0.10989300	1.57714700	C	0.12622100	0.80899800	-0.20932000
O	6.00835000	-0.00334600	-0.13594300	H	-0.11407600	1.24086100	-1.17912100
C	-0.66092000	1.51036300	0.89504800	C	-0.32259100	-0.64787100	-0.22495700
C	-1.61508400	2.57717700	0.47631200	H	-0.07621700	-1.13240200	0.72534200
H	-2.54172400	2.17049700	0.08837600	C	2.30892400	-0.19862700	-0.67878100
C	-1.62058000	3.88823800	1.17879100	C	1.68987000	-1.19766200	-1.30367900
H	-2.56037200	4.39860700	1.32707700	H	2.21483500	-1.91983400	-1.91011800
H	-0.90435700	4.01681400	1.97719100	C	0.64908000	4.47802000	-1.98277400
C	-1.07663200	3.81801900	-0.24942500	H	-0.09362800	5.19718200	-2.33172400
H	-1.75885100	4.19964800	-0.99686400	H	0.66967800	3.66738000	-2.72523800
C	0.33272400	3.98503500	-0.60751400	H	1.62953500	4.95682400	-2.01927800
C	1.47423200	3.51469100	0.24278600	C	-2.33771200	-1.92018300	-0.14642900
H	1.16606400	3.32176300	1.27087500	H	-1.91605600	-2.29066000	0.79186400

H	-2.14615000	-2.65552200	-0.92748000
C	-3.80282900	-1.64599800	-0.00891900
C	-4.27068900	-0.83202700	1.02945400
H	-3.56765300	-0.41178600	1.73620100
C	-5.61666500	-0.55205800	1.16154400
H	-5.98209500	0.07455700	1.96222300
C	-6.53678400	-1.08821100	0.25292700
C	-6.08802700	-1.90168200	-0.78488300
H	-6.77583100	-2.32570300	-1.49850600
C	-4.72440900	-2.16702200	-0.90610400
H	-4.38237800	-2.79255700	-1.71996400
C	-8.81720200	-1.28275400	-0.42776200
H	-8.64068000	-0.93985900	-1.44880800
H	-9.77272700	-0.90477200	-0.07873500
H	-8.82677900	-2.37392900	-0.40558200
F	5.78347500	-2.75954300	0.96297700
F	4.92887800	-2.73525500	-1.03377300
F	3.63081500	-2.84481300	0.70344700
C	4.76236200	-2.33582600	0.22436300

Transition state TS-A3

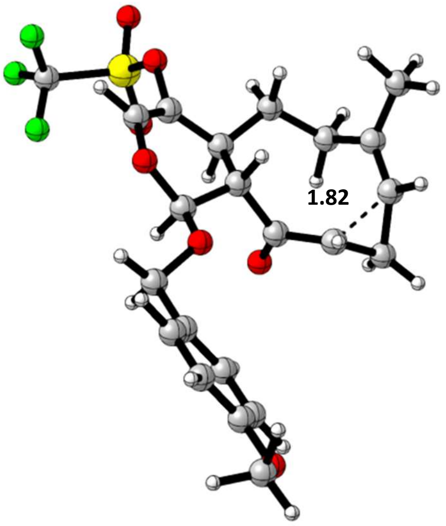

IEFPCM(toluene)B3LYP-D3/6-311++G(2d,2p) Electronic Energy = –2077.572914

IEFPCM(toluene)B3LYP-D3/6-311++G(2d,2p) Free Energy = –2077.236222 (T = 381.15 K)

Number of imaginary frequencies = 1 (553.89i cm^{-1})

S	4.67181200	-0.60881100	0.24275400	C	2.28606200	2.19160700	-0.37249000
O	-0.29483200	1.49476200	2.02036200	H	3.33884600	2.12141700	-0.10698400
O	0.23348300	-1.47538500	-1.12370500	H	2.25614200	2.27945600	-1.45859000
O	-1.75553900	-0.64448100	-0.35991400	C	1.62425800	0.86146000	0.04232000
O	-7.96356700	-0.70075200	0.33102000	H	1.76500100	0.74031900	1.11682700
O	3.63864000	-0.27679100	-0.94215800	C	0.10137700	0.79979200	-0.21811200
O	4.15331500	-0.20963600	1.52024300	H	-0.13043800	1.16940500	-1.21641600
O	5.95644600	-0.20398600	-0.24382500	C	-0.39930000	-0.63453700	-0.12668400
C	-0.59396800	1.64857400	0.83841300	H	-0.15305400	-1.06558400	0.84949300
C	-1.48459900	2.70619800	0.39462700	C	2.23929300	-0.30153500	-0.67676500
H	-2.23045100	2.47958100	-0.35083800	C	1.57141000	-1.31707300	-1.22020600
C	-1.42439800	4.00751200	1.08477800	H	2.05661200	-2.08871000	-1.79839000
H	-2.33305800	4.58694700	1.19111600	C	1.10048400	4.72404900	-1.86519000
H	-0.82244900	3.99914300	1.98384900	H	0.28409100	5.21812300	-2.38828200
C	-0.69479600	4.22445500	-0.21511200	H	1.49745800	3.94722600	-2.52625900
H	-1.33320200	4.63104500	-0.98830500	H	1.90693100	5.45170500	-1.73404700
C	0.66506400	4.17583400	-0.53907200	C	-2.43298500	-1.85792900	0.03002300
C	1.72433400	3.49199500	0.27694300	H	-2.05083200	-2.16049800	1.00916600
H	1.39110600	3.28773900	1.29220600	H	-2.21048400	-2.64734200	-0.68764800
H	2.56755600	4.18199400	0.36696100	C	-3.90317200	-1.58148900	0.08604500

C	-4.41672700	-0.68736500	1.03259500	C	-8.90062700	-1.30866800	-0.55121900
H	-3.74398400	-0.20014000	1.72549900	H	-8.68805600	-1.05131400	-1.59043700
C	-5.76898500	-0.41166800	1.08925300	H	-9.87192500	-0.91343200	-0.27165600
H	-6.16963600	0.27725200	1.81876400	H	-8.90238200	-2.39416500	-0.43714300
C	-6.64949400	-1.03273500	0.19578300	F	5.69550400	-2.91811800	0.94172800
C	-6.15516700	-1.92631700	-0.75134900	F	4.78216700	-2.93147600	-1.02897200
H	-6.81163200	-2.41662700	-1.45193000	F	3.53470500	-2.95697300	0.74811900
C	-4.78588600	-2.18622600	-0.79770300	C	4.66314700	-2.49067100	0.22090200
H	-4.40805200	-2.87488300	-1.54176900				

α-Keto radical A4

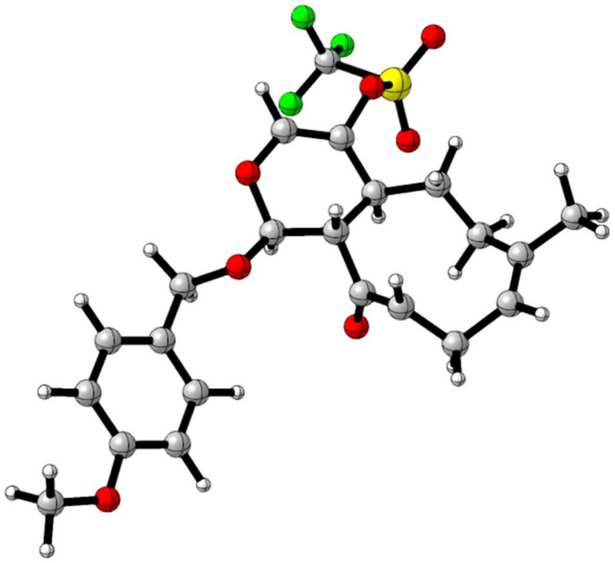

IEFPCM(toluene)B3LYP-D3/6-311++G(2d,2p) Electronic Energy = –2077.596203

IEFPCM(toluene)B3LYP-D3/6-311++G(2d,2p) Free Energy = –2077.257125 (T = 381.15 K)

Number of imaginary frequencies = 0

S	4.51871100	-0.84439500	0.16312500	H	1.30373700	2.80580800	1.23039400
O	-0.89363200	1.51198900	1.56022300	H	2.98631700	3.16043000	1.01276700
O	0.03663200	-1.77325500	-1.03558900	C	2.17219500	1.94302700	-0.57904200
O	-1.94062400	-0.75596000	-0.49687300	H	3.22564100	1.76386700	-0.77469600
O	-8.11070700	-0.18163000	0.36469800	H	1.73845900	2.25867600	-1.52820100
O	3.47479500	-0.64949000	-1.04456100	C	1.49999200	0.62535000	-0.13558000
O	4.02643500	-0.26108600	1.37917700	H	1.60151600	0.54893900	0.94904200
O	5.80454000	-0.53946600	-0.38818300	C	-0.01604300	0.60325100	-0.47223600
C	-0.69694000	1.68237100	0.35314500	H	-0.14092100	0.80802800	-1.53530700
C	-0.92403700	2.93915200	-0.30130500	C	-0.60138900	-0.76994400	-0.19407400
H	-0.98318800	2.95206500	-1.38144100	H	-0.42529400	-1.07149100	0.84196100
C	-0.79582100	4.23827500	0.40477800	C	2.08154600	-0.60642800	-0.76103900
H	-1.65657600	4.87861700	0.20430200	C	1.37403900	-1.65899900	-1.16869000
H	-0.75362600	4.07222800	1.48103700	H	1.82833600	-2.50575000	-1.66077900
C	0.45813400	4.93278900	-0.12029700	C	2.84779800	5.17550500	-0.71830500
H	0.30272600	5.89871400	-0.58546900	H	2.53343200	6.12070000	-1.15676600
C	1.69561400	4.42479400	-0.11156000	H	3.32793300	4.57920800	-1.49878900
C	2.03848700	3.07799900	0.47885600	H	3.61426800	5.37841400	0.03344800

C	-2.70937700	-1.84088000	0.06233900
H	-2.32398600	-2.04888100	1.06444100
H	-2.58007000	-2.73387600	-0.54930700
C	-4.14820000	-1.42932500	0.11606100
C	-4.53908600	-0.34226200	0.90693800
H	-3.79301100	0.20164100	1.47057900
C	-5.86158800	0.05110200	0.96788400
H	-6.16835100	0.88948800	1.57645400
C	-6.83444800	-0.64139700	0.23749300
C	-6.46205100	-1.72504300	-0.55389000
H	-7.19110600	-2.27258600	-1.12904200
C	-5.12063100	-2.10318600	-0.60875100
H	-4.83729600	-2.94047500	-1.23283900
C	-9.13755500	-0.85406800	-0.35554800
H	-8.96346400	-0.80191100	-1.43186300
H	-10.05970900	-0.33603700	-0.11234900
H	-9.21481800	-1.89876900	-0.04885900
F	5.49274000	-3.06257000	1.16389300
F	4.55990200	-3.31965100	-0.78071900
F	3.32954500	-3.07024900	0.99107100
C	4.46444300	-2.71096400	0.39814900

Conformer 23

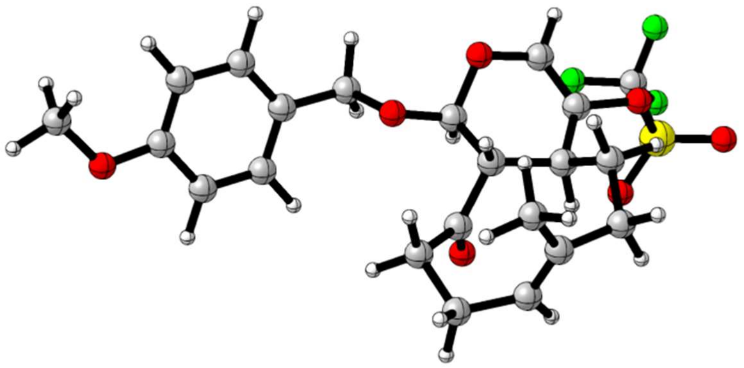

IEFPCM(tetrahydrofuran)B3LYP-D3/6-311++G(2d,2p) Electronic Energy = –2078.251947

IEFPCM(tetrahydrofuran)B3LYP-D3/6-311++G(2d,2p) Free Energy = –2077.867425 (T = 298.15 K)

Number of imaginary frequencies = 0

S	4.52778600	-0.59061000	0.44175500
O	-0.15664300	1.02947600	2.01368900
O	0.30730600	-1.21086600	-1.54112900
O	-1.67739100	-0.54163100	-0.61059000
O	-7.78878400	-0.80321900	0.61894000
O	3.71565700	-0.21535100	-0.89170400
O	3.78821500	-0.26042400	1.62685800
O	5.87197200	-0.15062800	0.21024600
C	-0.54813100	1.44671100	0.94267400
C	-1.71431200	2.41625700	0.84986900
H	-2.08292900	2.44508500	-0.17232600
C	-1.22895000	3.82855800	1.28376000
H	-1.87916100	4.58274400	0.84298100
H	-1.30962300	3.92362600	2.36425000
C	0.21274100	3.97080700	0.86556800
H	0.94192800	3.75810200	1.63881600
C	0.65806000	4.06091700	-0.38883000
C	2.08304500	3.68801100	-0.68876000
H	2.68126300	3.75136400	0.22100600
H	2.52560500	4.36908300	-1.41963800
C	2.19465800	2.25864600	-1.25660000
H	3.24897700	2.07593300	-1.45829900
H	1.68487200	2.19012100	-2.21880900
C	1.67582600	1.10541600	-0.35089700
H	2.00452900	1.27446900	0.67465200
C	0.12383100	0.95098300	-0.34920900
H	-0.30071000	1.45700000	-1.21125900
C	-0.31202100	-0.51417600	-0.43728600
H	-0.00533100	-1.04658300	0.46857800
C	2.29239900	-0.16664600	-0.85472100
C	1.65953100	-1.15646400	-1.47539400
H	2.16684800	-1.97559500	-1.96208100
C	-0.21763300	4.36569600	-1.57187600
H	-1.23739700	4.61037300	-1.28619800
H	-0.25598700	3.52984900	-2.27350000
H	0.19226300	5.21278600	-2.12664700
C	-2.30352900	-1.81575500	-0.32496000
H	-1.82943400	-2.23421900	0.56585700
H	-2.13651800	-2.49402500	-1.16067500
C	-3.76395700	-1.58184400	-0.09993200
C	-4.20464600	-0.95469400	1.07108600
H	-3.48372100	-0.65845500	1.82133100

C	-5.54712500	-0.70584600	1.28316900
H	-5.89007700	-0.22378000	2.18733300
C	-6.49055300	-1.08566300	0.32112500
C	-6.06929000	-1.71360900	-0.84943100
H	-6.77604600	-2.01650300	-1.60494800
C	-4.70982500	-1.95118400	-1.04728300
H	-4.38988600	-2.43483500	-1.96066400
C	-8.78872100	-1.17416700	-0.32811200
H	-8.63490300	-0.66524200	-1.28066300
H	-9.73485500	-0.86238200	0.10189300
H	-8.79753400	-2.25344500	-0.48685500
F	5.40824900	-2.92357800	1.24173500
F	4.96777700	-2.83841600	-0.88354500
F	3.34849100	-2.96408800	0.55787200
C	4.55950300	-2.46690600	0.32556800
H	-2.51548900	2.05987100	1.49557500

Transition state TS-A5

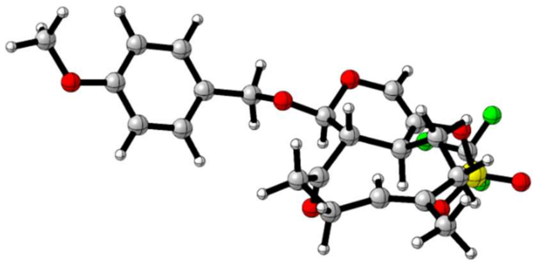

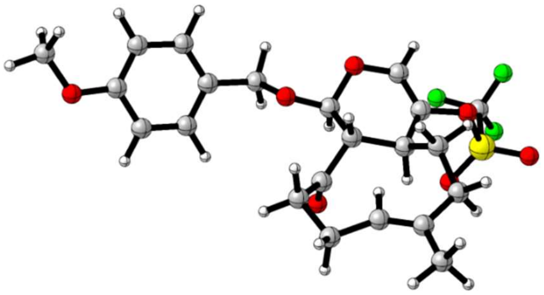

IEFPCM(tetrahydrofuran)B3LYP-D3/6-311++G(2d,2p) Electronic Energy = –2078.218854

IEFPCM(tetrahydrofuran)B3LYP-D3/6-311++G(2d,2p) Free Energy = –2077.835054 (T = 298.15 K)

Number of imaginary frequencies = 1 (195.62i cm^{-1})

S	4.34711900	-0.88668700	0.39587700
O	-0.48512400	1.14656700	1.88011300
O	0.00164400	-1.49651800	-1.33253300
O	-1.91699300	-0.56482200	-0.50296500
O	-8.08400100	-0.28968600	0.42793400
O	3.48672700	-0.63343000	-0.93722300
O	3.62253100	-0.50645800	1.57551700
O	5.67014400	-0.41902200	0.10471000
C	-0.66650700	1.56468500	0.75608100
C	-1.49738100	2.82936200	0.51467800
H	-1.86577200	2.83393300	-0.51000800
C	-0.60120300	4.08150500	0.75716600
H	-1.07524800	4.97777800	0.35817000
H	-0.48460300	4.23131700	1.83287100
C	0.71121000	3.74149800	0.11749200
H	0.74766500	2.69597800	-0.02913000
C	1.84955400	4.26447400	-0.31578700
C	2.81885900	3.24021300	-0.89551800
H	3.59235000	3.00691100	-0.15552700
H	3.35228900	3.69249000	-1.73569700
C	2.19313400	1.92664800	-1.43382100
H	2.98929100	1.42405700	-1.97776300
H	1.43508200	2.18150700	-2.17479600
C	1.56492000	0.85592800	-0.44623000
H	1.90325200	1.05698400	0.57056800
C	-0.01453400	0.80809500	-0.43986400
H	-0.38353100	1.21820000	-1.37739000
C	-0.55113600	-0.61598600	-0.32794400
H	-0.28087800	-1.04455800	0.64131100
C	2.07178500	-0.49634600	-0.84791600
C	1.35451000	-1.51079500	-1.31905400
H	1.79481600	-2.40922600	-1.72388100
C	2.26307300	5.70190000	-0.27256600
H	1.49556000	6.32776700	0.17971500
H	2.47069500	6.07908300	-1.27689600
H	3.18415000	5.82161900	0.30371800
C	-2.63279900	-1.73243900	-0.03486600
H	-2.22372500	-2.01234900	0.93891200
H	-2.47428300	-2.55607900	-0.72999800
C	-4.08448500	-1.38511700	0.06821000
C	-4.53099500	-0.50900900	1.06425900
H	-3.82145000	-0.09597500	1.76873000
C	-5.86467900	-0.16023500	1.15914900
H	-6.21218400	0.51477500	1.92800700

C	-6.79337700	-0.68780800	0.25409000
C	-6.36622100	-1.56384800	-0.74175400
H	-7.06118200	-1.98430700	-1.45044600
C	-5.01539500	-1.89890600	-0.82497600
H	-4.69052600	-2.57521100	-1.60445400
C	-9.06889000	-0.80084400	-0.46810300
H	-8.85360400	-0.50786200	-1.49665300
H	-10.01078200	-0.36243000	-0.15512200
H	-9.13257200	-1.88796400	-0.40279400
F	5.24944500	-3.14196200	1.37451800
F	4.91827600	-3.18914100	-0.77161200
F	3.22909100	-3.27881800	0.59057000
C	4.43849600	-2.76213500	0.39143200
H	-2.34706100	2.81175100	1.19387300

Conformer 23’

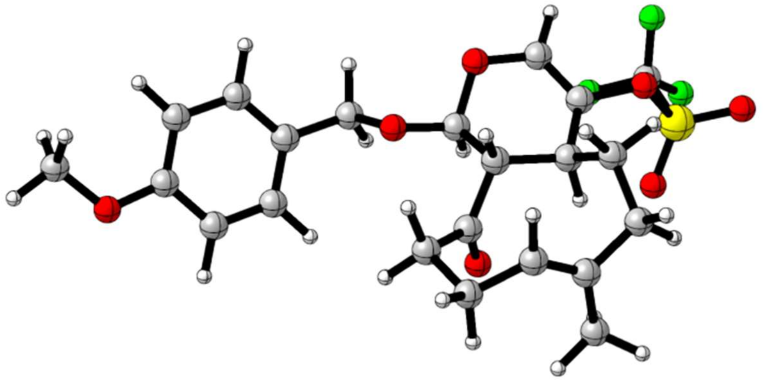

IEFPCM(tetrahydrofuran)B3LYP-D3/6-311++G(2d,2p) Electronic Energy = –2078.252642

IEFPCM(tetrahydrofuran)B3LYP-D3/6-311++G(2d,2p) Free Energy = –2077.869359 (T = 298.15 K)

Number of imaginary frequencies = 0

S	4.51219600	-0.64010100	0.26979800	C	2.22144100	2.04012300	-1.49975400
O	-0.33874800	1.51257900	1.59868500	H	3.17089800	1.69203700	-1.90199800
O	0.18774800	-1.52661200	-1.35466200	H	1.55655200	2.15931000	-2.35474600
O	-1.76861900	-0.62712100	-0.58164800	C	1.62995600	0.93724200	-0.56906800
O	-7.89483300	-0.45490700	0.58053900	H	1.90467500	1.15119600	0.46376000
O	3.62801800	-0.47342600	-1.05853300	C	0.08044600	0.83397900	-0.64420000
O	3.90271300	0.00002100	1.40130700	H	-0.25919200	1.13011500	-1.63358100
O	5.86239100	-0.38379700	-0.13660100	C	-0.40200700	-0.60074500	-0.41607800
C	-0.65193400	1.64868400	0.43357800	H	-0.10620300	-0.94044800	0.58196700
C	-1.78680300	2.56855900	0.01720300	C	2.20845000	-0.39226000	-0.95044400
H	-2.14933000	2.27958200	-0.96719400	C	1.53914700	-1.46153200	-1.37049100
C	-1.25982300	4.02925200	-0.01071400	H	2.02193800	-2.34711000	-1.75504700
H	-1.97400400	4.65605100	-0.54453700	C	1.49280300	4.16905700	1.39864500
H	-1.19206900	4.40059000	1.00844600	H	0.62819100	4.59203700	1.90144500
C	0.08440800	3.99721500	-0.67844900	H	2.33216000	4.85718400	1.52344500
H	0.05442900	3.79364500	-1.74472100	H	1.76420900	3.24479800	1.91386600
C	1.26635600	3.91142700	-0.06307700	C	-2.43118800	-1.79037000	-0.03127300
C	2.47287500	3.39831100	-0.82505100	H	-1.97682400	-2.01336100	0.93699600
H	3.31192100	3.29698700	-0.13560700	H	-2.27652200	-2.64117300	-0.69369700
H	2.79000300	4.11191700	-1.58878000	C	-3.88664900	-1.47513400	0.11441800

C	-4.31715400	-0.57038000	1.09175600	C	-8.89955300	-1.02267300	-0.25760000
H	-3.59261200	-0.11395400	1.75282800	H	-8.72890600	-0.76567900	-1.30397000
C	-5.65417600	-0.24758100	1.22279700	H	-9.83941500	-0.59319500	0.07333200
H	-5.98921200	0.44945300	1.97738900	H	-8.93506400	-2.10750100	-0.14799500
C	-6.60252900	-0.83091200	0.37401700	F	5.32646600	-2.83813300	1.44160900
C	-6.19136100	-1.73583500	-0.60258000	F	4.58355200	-3.14966700	-0.57542100
H	-6.90158300	-2.19911200	-1.26812000	F	3.19132200	-2.80631300	1.05573300
C	-4.83694300	-2.04414200	-0.72303700	C	4.38690500	-2.49567400	0.56533300
H	-4.52467000	-2.74309100	-1.48754400	H	-2.60193800	2.46477700	0.73172600

Conformer 21

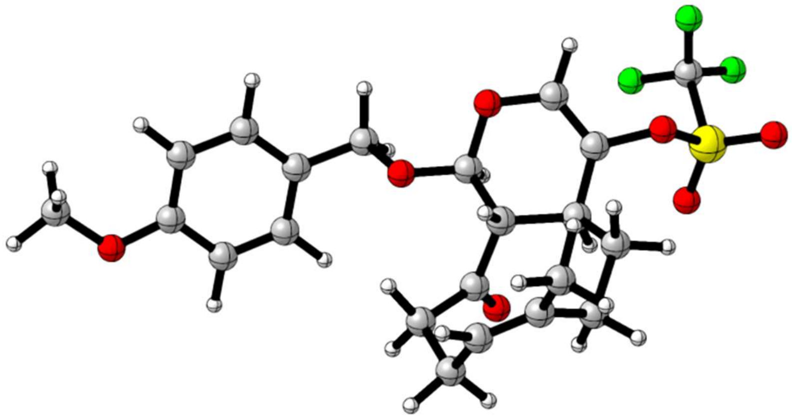

IEFPCM(tetrahydrofuran)B3LYP-D3/6-311++G(2d,2p) Electronic Energy = –2078.261514

IEFPCM(tetrahydrofuran)B3LYP-D3/6-311++G(2d,2p) Free Energy = –2077.877351 (T = 298.15 K)

Number of imaginary frequencies = 0

S	4.64365400	-0.63946300	0.38507200	C	2.30242100	2.33149400	-0.28492000
O	-0.23827900	1.09197200	2.26572300	H	3.33084300	2.32844500	0.07115800
O	0.31190900	-1.14554500	-1.40420200	H	2.36160400	2.37196900	-1.37227400
O	-1.68087900	-0.54043000	-0.46253600	C	1.67026000	0.98344500	0.12806400
O	-7.83360000	-0.86068100	0.50154200	H	1.85850100	0.82498000	1.19005600
O	3.71777600	-0.10255700	-0.80964800	C	0.14108100	0.91937100	-0.07589900
O	4.04906100	-0.39530900	1.66899400	H	-0.16155100	1.47556900	-0.95981200
O	5.97493900	-0.22687600	0.05121100	C	-0.32022600	-0.52968200	-0.26152100
C	-0.61596400	1.42068300	1.15785300	H	-0.03347300	-1.12710300	0.61096800
C	-1.82429500	2.29729500	0.97244900	C	2.29992200	-0.12449200	-0.66032200
H	-2.33987000	2.03908000	0.04920100	C	1.65996700	-1.03007700	-1.39425100
C	-1.41158900	3.79239700	0.95254100	H	2.16627200	-1.72961000	-2.04154700
H	-2.31650300	4.37912500	1.10640500	C	0.99920400	4.64280800	-2.00513600
H	-0.76727700	3.98698200	1.81015700	H	0.18028600	5.06746300	-2.58232700
C	-0.76674700	4.22515300	-0.33486400	H	1.44938100	3.84484300	-2.59863700
H	-1.44844200	4.62533900	-1.07790800	H	1.76768500	5.40997300	-1.88064800
C	0.52445500	4.14619400	-0.66454600	C	-2.31633400	-1.82952400	-0.29001700
C	1.60437900	3.60298000	0.24380100	H	-1.87676000	-2.30909300	0.58790100
H	1.22965800	3.42918700	1.25115200	H	-2.11780100	-2.45014800	-1.16279700
H	2.37106100	4.37635800	0.33678400	C	-3.78442900	-1.60560400	-0.10715000

C	-4.26571900	-1.01530000	1.06706700	C	-8.80045200	-1.20528400	-0.48883000
H	-3.57094800	-0.73803300	1.84851800	H	-8.61727500	-0.66719900	-1.41991800
C	-5.61532700	-0.77686400	1.24192500	H	-9.76136500	-0.90926300	-0.08103100
H	-5.98964900	-0.32245800	2.14796400	H	-8.80005500	-2.27924300	-0.68038100
C	-6.52525300	-1.13059800	0.23841200	F	5.55648600	-3.06018500	0.80149600
C	-6.06322000	-1.72143300	-0.93594700	F	4.74057700	-2.76839400	-1.19014100
H	-6.74334000	-2.00281300	-1.72355800	F	3.40748400	-2.98710800	0.51035000
C	-4.69686700	-1.94831000	-1.09614400	C	4.57443900	-2.50018200	0.10154300
H	-4.34508300	-2.40165800	-2.01333000	H	-2.49093000	2.11850100	1.81409300

4 X-ray data

4.1 Sulfone 20

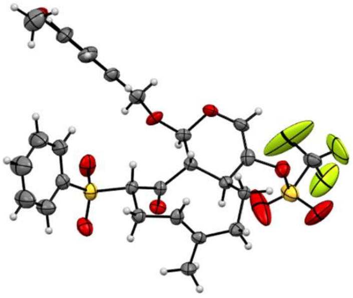

Thermal ellipsoids are shown at the 50% probability level.

Table S10: Crystal data and structure refinement for sulfone **20**.

Identification code	**20**
Empirical formula	$C_{28}H_{29}F_3O_9S_2$
Formula weight	630.63
Temperature	173.00 K
Wavelength	0.71073 Å
Crystal system	Monoclinic
Space group	$P2_1$ (no. 4)
Unit cell dimensions	a = 15.939(4) Å α= 90°.<br>b = 5.5097(13) Å β= 112.860(7)°.<br>c = 18.388(4) Å γ = 90°.
Volume	1488.0(6) Å^3
Z	2
Density (calculated)	1.408 Mg/m^3
Absorption coefficient	0.249 mm^{-1}
F(000)	656
Crystal size	0.18 x 0.09 x 0.02 mm^3
Theta range for data collection	2.159 to 25.024°.
Index ranges	-18<=h<=18, -6<=k<=6, -21<=l<=21
Reflections collected	4957
Independent reflections	4957 [R(int) = ?]
Completeness to theta = 25.242°	97.2 %
Absorption correction	Semi-empirical from equivalents
Max. and min. transmission	0.958 and 0.716
Refinement method	Full-matrix least-squares on F^2
Data / restraints / parameters	4957 / 1 / 419
Goodness-of-fit on F^2	1.053
Final R indices [I>2sigma(I)]	R1 = 0.0496, wR2 = 0.0990
R indices (all data)	R1 = 0.0724, wR2 = 0.1135
Absolute structure parameter	-0.03(9)
Extinction coefficient	0.014(2)
Largest diff. peak and hole	0.249 and -0.273 e.Å^{-3}

4.2 Acetate 22

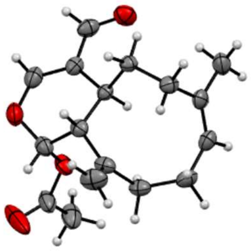

Thermal ellipsoids are shown at the 50% probability level.

Table S11: Crystal data and structure refinement for acetate **22**.

Identification code	**22**
Empirical formula	$C_{17}H_{22}O_4$
Formula weight	290.34
Temperature	173.00 K
Wavelength	0.71073 Å
Crystal system	Orthorhombic
Space group	$P2_12_12_1$ (no. 19)
Unit cell dimensions	a = 6.2787(2) Å α = 90°.<br>b = 14.8443(6) Å β= 90°.<br>c = 17.1662(6) Å γ = 90°.
Volume	1599.94(10) $Å^3$
Z	4
Density (calculated)	1.205 Mg/m^3
Absorption coefficient	0.085 mm^{-1}
F(000)	624
Crystal size	0.17 x 0.1 x 0.1 mm^3
Theta range for data collection	2.373 to 26.542°.
Index ranges	-7<=h<=7, -18<=k<=18, -21<=l<=21
Reflections collected	22659
Independent reflections	3319 [R(int) = 0.0385]
Completeness to theta = 25.242°	99.8 %
Absorption correction	Semi-empirical from equivalents
Max. and min. transmission	0.9585 and 0.9163
Refinement method	Full-matrix least-squares on F^2
Data / restraints / parameters	3319 / 0 / 193
Goodness-of-fit on F^2	1.039
Final R indices [I>2sigma(I)]	R1 = 0.0320, wR2 = 0.0790
R indices (all data)	R1 = 0.0369, wR2 = 0.0827
Absolute structure parameter	0.0(4)
Extinction coefficient	0.013(2)
Largest diff. peak and hole	0.155 and -0.170 e.$Å^{-3}$

5 References

(1) Guindeuil S. Study of new radicalar reactions. Application to the synthesis of polycyclic compounds. Ph.D. Dissertation, École polytechnique, Palaiseau, France, **2006**.

(2) Comely, A. C.; Eelkema, R.; Minnaard, A.J.; Feringa, B. L. De Novo Asymmetric Bio- and Chemocatalytic Synthesis of Saccharides − Stereoselective Formal O-Glycoside Bond Formation Using Palladium Catalysis. *J. Am. Chem. Soc.* **2003**, *125*, 8714–8715.

(3) Jung, H.-Y.; Feng, X.; Kim, H.; Yun, J. Copper-catalyzed boration of activated alkynes. Chiral boranes via a one-pot copper-catalyzed boration and reduction protocol. *Tetrahedron* **2012**, *68*, 3444-3449.

(4) Knobloch, E.; Brückner, R. β-Keto Esters Derived from 2-(Trimethylsilyl)ethanol: An Orthogonal Protective Group for β-Keto Esters. *Synthesis* **2008**, 2229–2246.

(5) Coval, S. J.; Scheuer, P. J.; Matsumoto, G. K.; Clardy, J. Two new xenicin diterpenoids from the octocoral *anthelia edmondsoni*. *Tetrahedron* **1984**, *40*, 3823–3828.

(6) Corval, S. J. Secondary Metabolites of Four Marine Invertebrates. Ph.D Dissertation, University of Hawaii, Honolulu, USA, **1985**.

(7) Fox, D. T.; Poulter, C. D. Synthesis of (*E*)-4-Hydroxydimethylallyl Diphosphate. An Intermediate in the Methyl Erythritol Phosphate Branch of the Isoprenoid Pathway. *J. Org. Chem.* **2002**, 67, 14, 5009–5010.

(8) Ardolino, M. J.; Morken, J. P. Congested C–C Bonds by Pd-Catalyzed Enantioselective Allyl–Allyl Cross-Coupling, a Mechanism-Guided Solution. *J. Am. Chem. Soc.* **2014**, *136*, 7092–7100.

(9) Kashman, Y.; Groweiss, A. New diterpenoids from the soft corals *Xenia macrospiculata* and *Xenia obscuronata*. *J. Org. Chem.* **1980**, *45*, 3814–3824.

(10) Kashman, Y.; Saltoun, M.; Rudi, A.; Benayahu, Y. Xeniafaraunol A and B, and faraunatin; three new cytotoxic diterpenes from the soft coral *xenia faraunensis*. *Tetrahedron Lett.* **1994**, *35*, 8855–8858.

(11) Gaussian 16, Revision C.01, Frisch, M. J.; Trucks, G. W.; Schlegel, H. B.; Scuseria, G. E.; Robb, M. A.; Cheeseman, J. R.; Scalmani, G.; Barone, V.; Petersson, G. A.; Nakatsuji, H.; Li, X.; Caricato, M.; Marenich, A. V.; Bloino, J.; Janesko, B. G.; Gomperts, R.; Mennucci, B.; Hratchian, H. P.; Ortiz, J. V.; Izmaylov, A. F.; Sonnenberg, J. L.; Williams-Young, D.; Ding, F.; Lipparini, F.; Egidi, F.; Goings, J.; Peng, B.; Petrone, A.; Henderson, T.; Ranasinghe, D.; Zakrzewski, V. G.; Gao, J.; Rega, N.; Zheng, G.; Liang, W.; Hada, M.; Ehara, M.; Toyota, K.; Fukuda, R.; Hasegawa, J.; Ishida, M.; Nakajima, T.; Honda, Y.; Kitao, O.; Nakai, H.; Vreven, T.; Throssell, K.; Montgomery, J. A., Jr.; Peralta, J. E.; Ogliaro, F.; Bearpark, M. J.; Heyd, J. J.; Brothers, E. N.; Kudin, K. N.; Staroverov, V. N.; Keith, T. A.; Kobayashi, R.; Normand, J.; Raghavachari, K.; Rendell, A. P.; Burant, J. C.; Iyengar, S. S.; Tomasi, J.; Cossi, M.; Millam, J. M.; Klene, M.; Adamo, C.; Cammi, R.; Ochterski, J. W.; Martin, R. L.; Morokuma, K.; Farkas, O.; Foresman, J. B.; Fox, D. J. Gaussian, Inc., Wallingford CT, 2016.

(12) Becke, A. D. Density-functional thermochemistry. III. The role of exact exchange. *J. Chem. Phys.* **1993**, *98*, 5648–5652.

(13) Lee, C., Yang, W.; Parr, R. G. Development of the Colle-Salvetti correlation-energy formula into a functional of the electron density. *Phys. Rev. B* **1988**, *37*, 785–789.

(14) Vosko, S. H., Wilk, L.; Nusair, M. Accurate spin-dependent electron liquid correlation energies for local spin density calculations: a critical analysis. *Can. J. Phys.* **1980**, *58*, 1200–1211.

(15) Stephens, P. J., Devlin, F. J., Chabalowski, C. F.; Frisch, M. J. Ab Initio Calculation of Vibrational Absorption and Circular Dichroism Spectra Using Density Functional Force Fields. *J. Phys. Chem.* **1994**, *98*, 11623–11627.

(16) Ditchfield, R., Hehre W. J.; Pople, J. A. Self-Consistent Molecular-Orbital Methods. IX. An Extended Gaussian-Type Basis for Molecular-Orbital Studies of Organic Molecules. *J. Chem. Phys.* **1971**, *54*, 724–728.

(17) Miertuš, S.; Tomasi, J. Approximate evaluations of the electrostatic free energy and internal energy changes in solution processes. *Chem. Phys.* **1982**, *65*, 239–245.

(18) Miertuš, S., Scrocco, E.; Tomasi, J. Electrostatic interaction of a solute with a continuum. A direct utilization of AB initio molecular potentials for the prevision of solvent effects. *Chem. Phys.* **1981**, *55*, 117–129.

(19) Pascual-Ahuir, L., Silla, E.; Tuñón, I. GEPOL: An improved description of molecular surfaces. III. A new algorithm for the computation of a solvent-excluding surface. *J. Comp. Chem.* **1994**, *15*, 1127–1138.

(20) Grimme, S., Ehrlich, S.; Goerigk, L. Effect of the damping function in dispersion corrected density functional theory. *J. Comp. Chem.* **2011**, *32*, 1456–1465.

(21) Legault, C.: CylView. http://www.cylview.org.

^{1}H and ^{13}C NMR Spectra

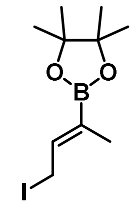

13

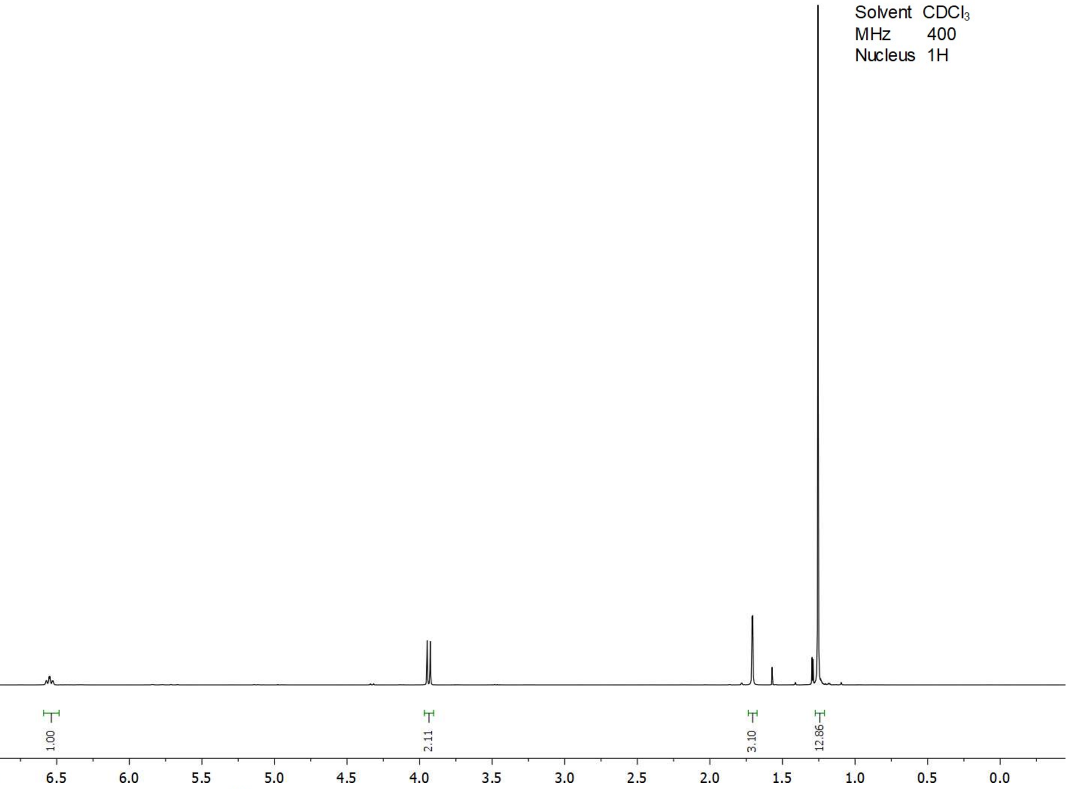

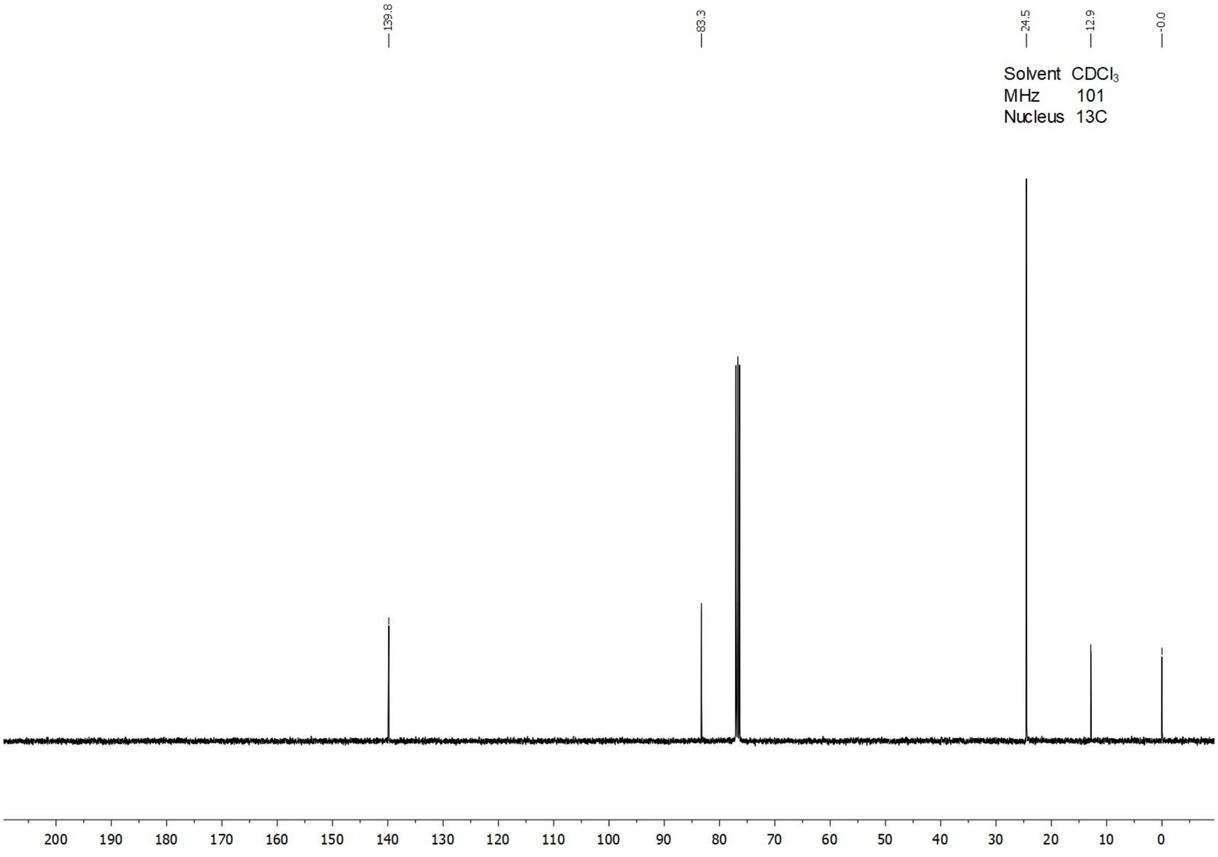

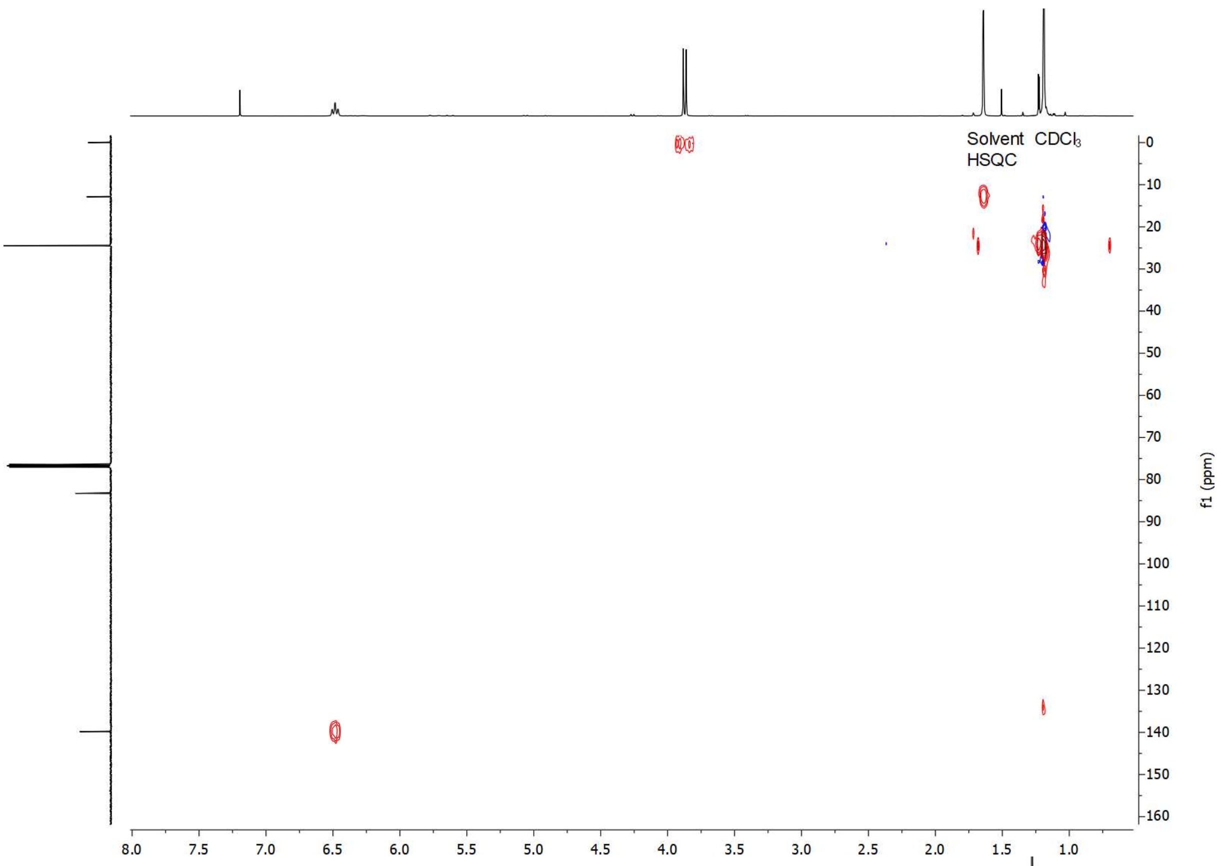
Solvent CDCl3
HSQC
f1 (ppm)

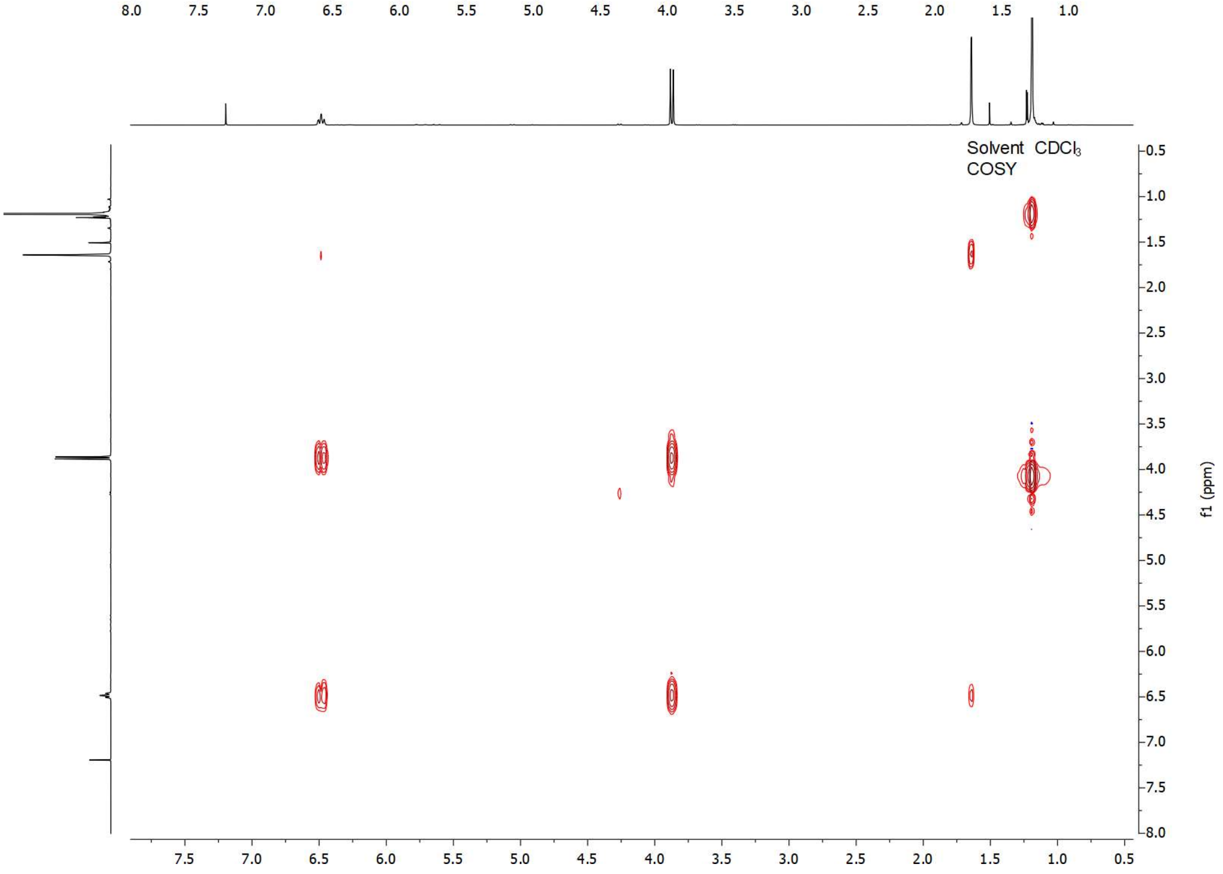
Solvent CDCl3
COSY
f1 (ppm)

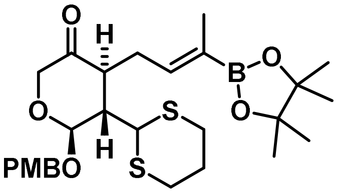

14

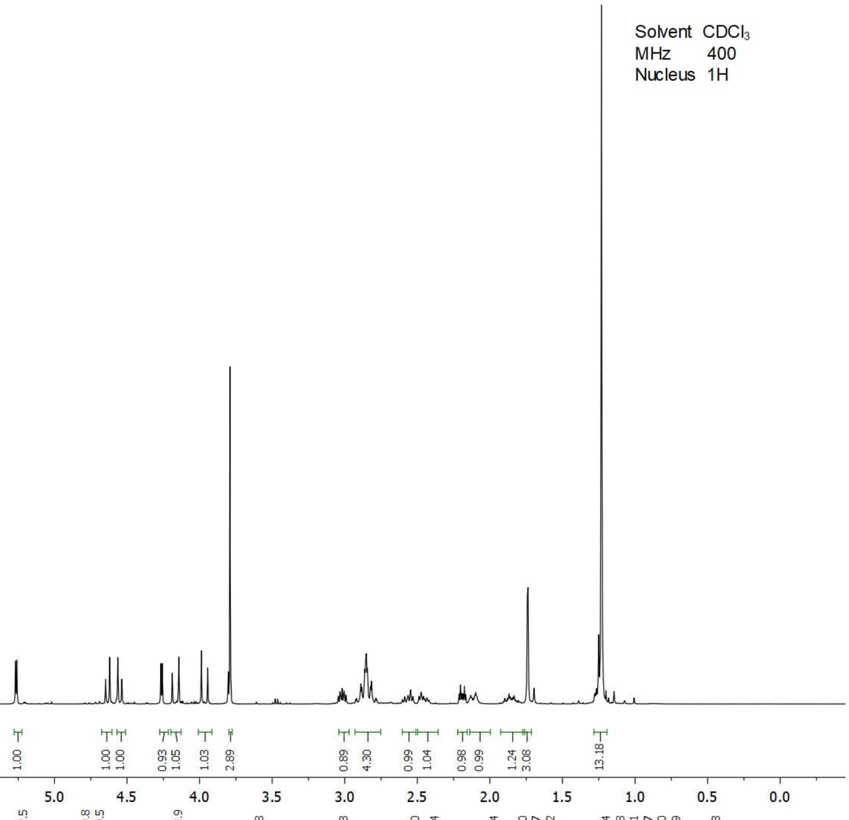

Solvent CDCl3
MHz 400
Nucleus 1H

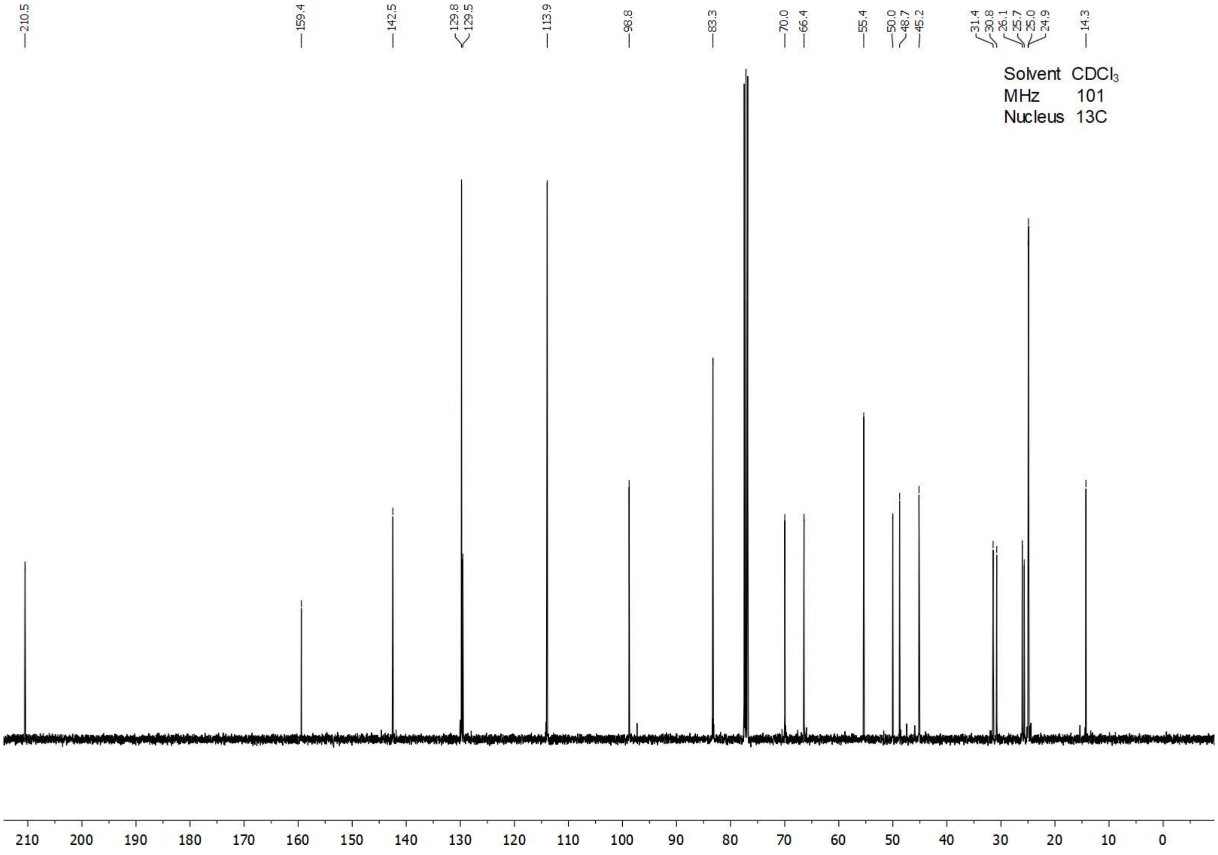

Solvent CDCl3
MHz 101
Nucleus 13C

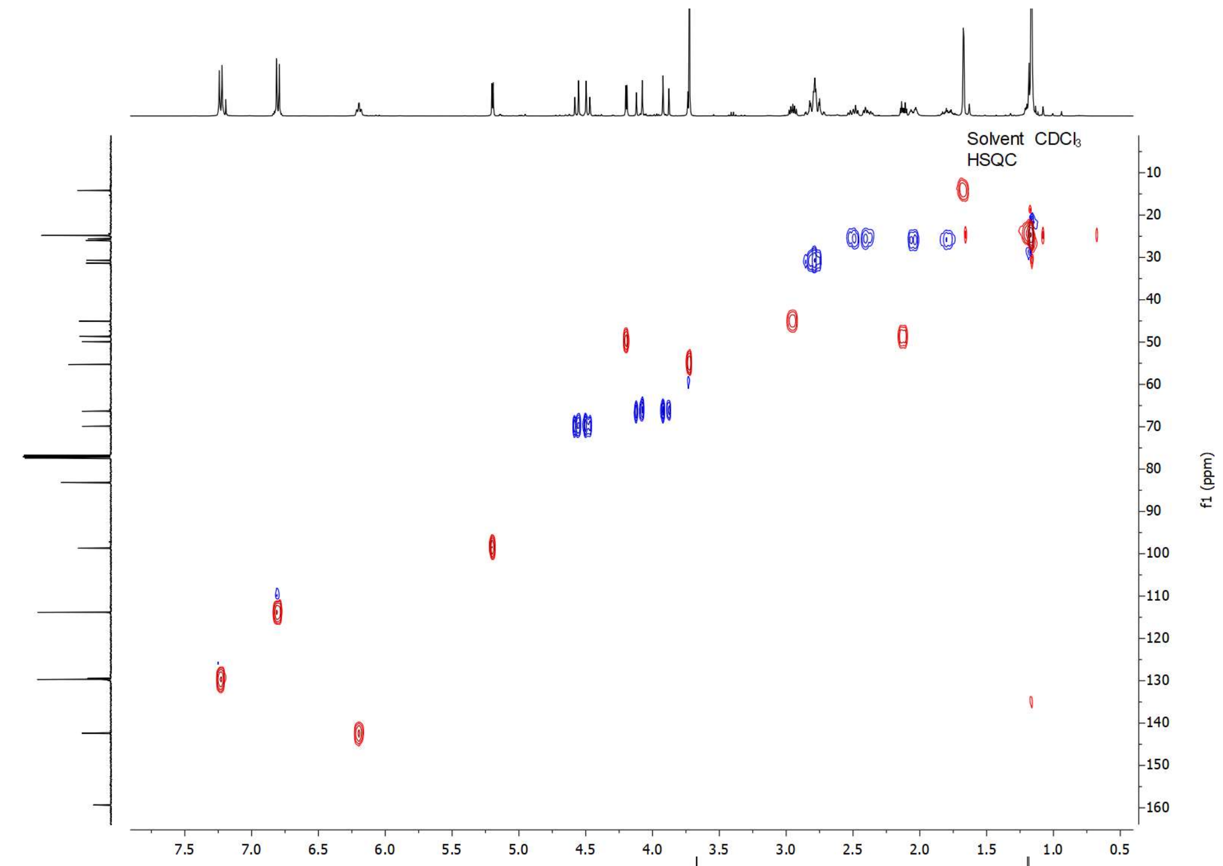
Solvent CDCl3
HSQC
f1 (ppm)

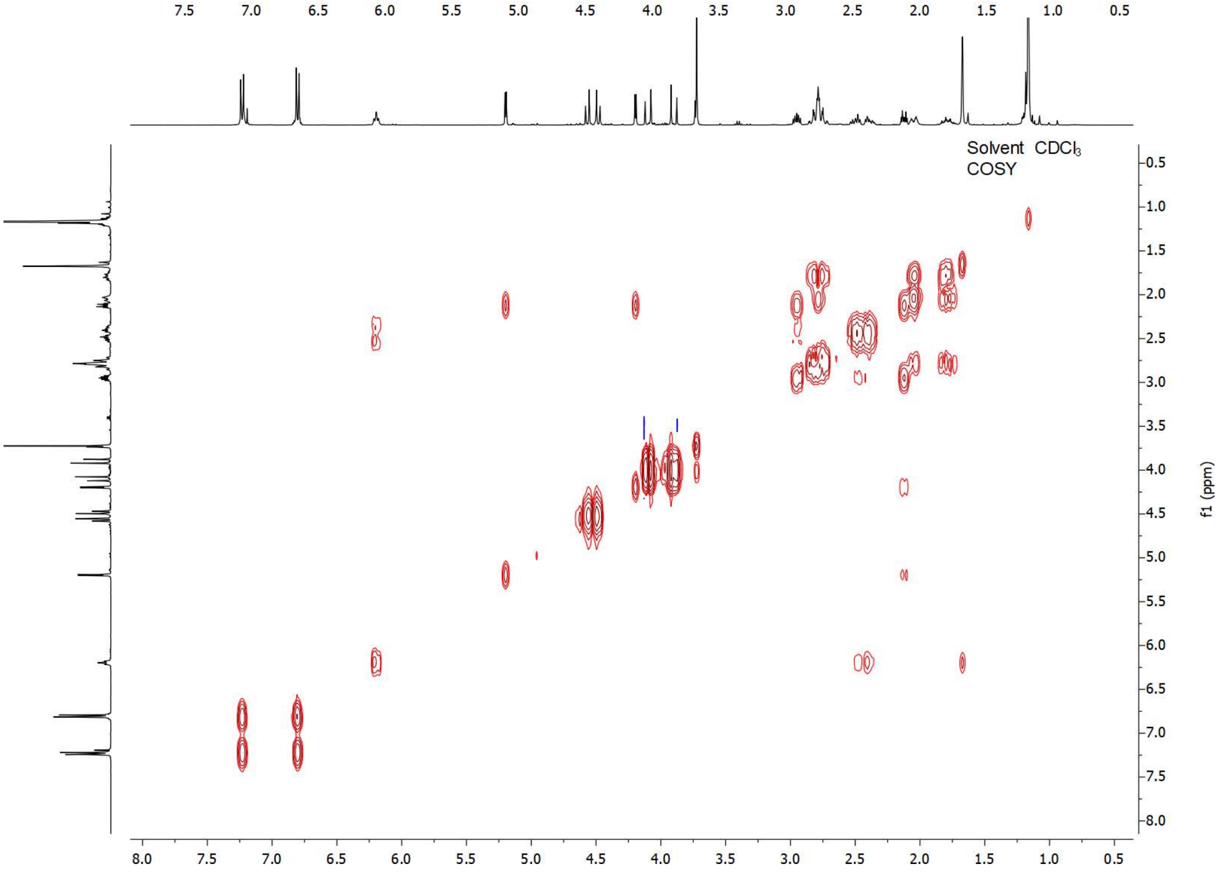
Solvent CDCl3
COSY
f1 (ppm)

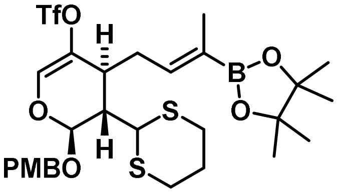
TfO
H
O
PMBO
H
S
S
B
O
O

S6

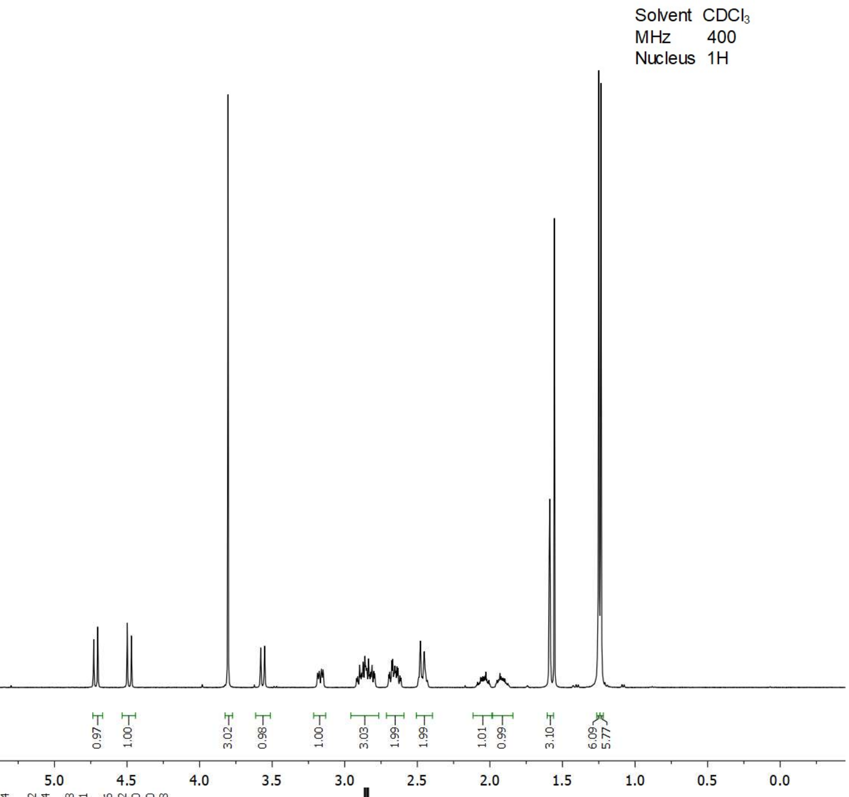
Solvent CDCl3
MHz 400
Nucleus 1H
1.93
2.02
0.97
1.00
0.99
0.97
1.00
3.02
0.98
1.00
3.03
1.99
1.99
1.01
0.99
3.10
6.09
5.77
7.5 7.0 6.5 6.0 5.5 5.0 4.5 4.0 3.5 3.0 2.5 2.0 1.5 1.0 0.5 0.0

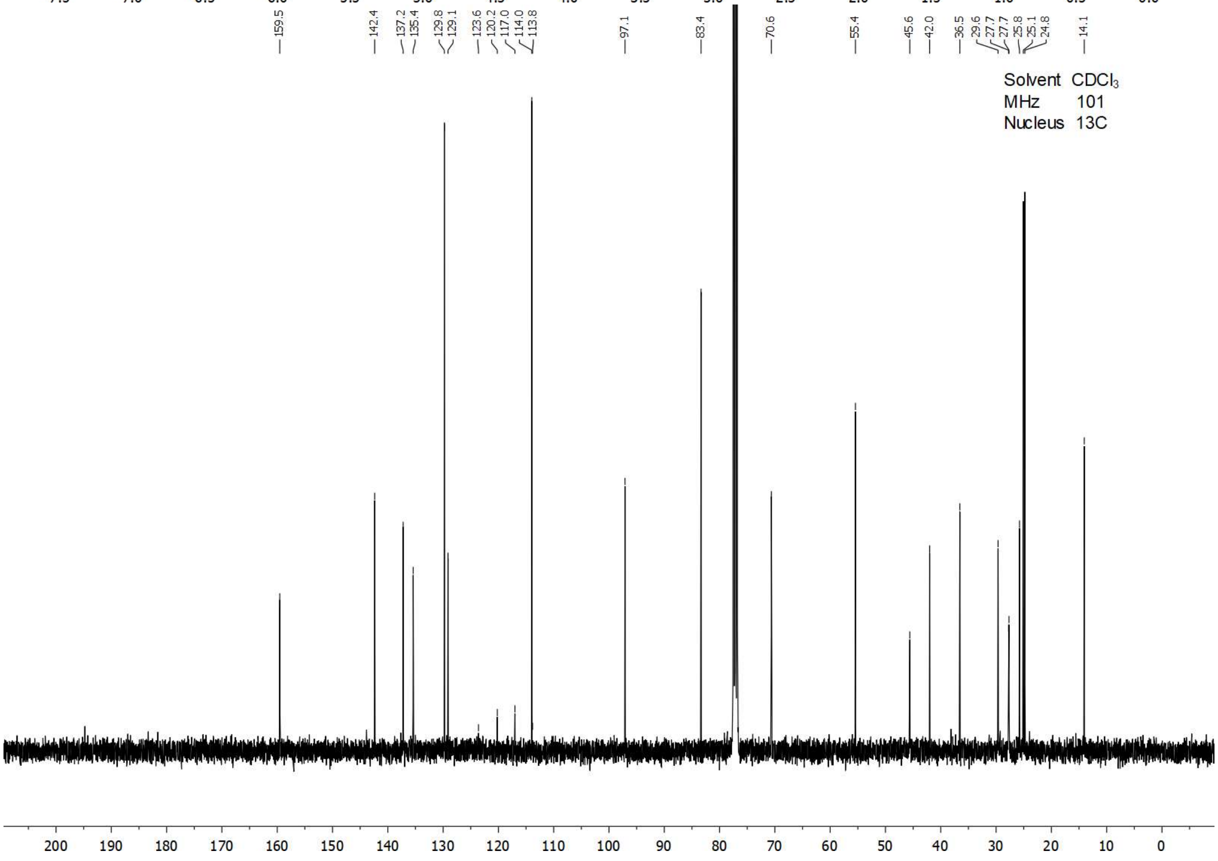
159.5
142.4
137.2
135.4
129.8
129.1
123.6
120.2
117.0
114.0
113.8
97.1
83.4
70.6
55.4
45.6
42.0
36.5
29.6
27.7
27.7
25.8
25.1
24.8
14.1
Solvent CDCl3
MHz 101
Nucleus 13C
200 190 180 170 160 150 140 130 120 110 100 90 80 70 60 50 40 30 20 10 0

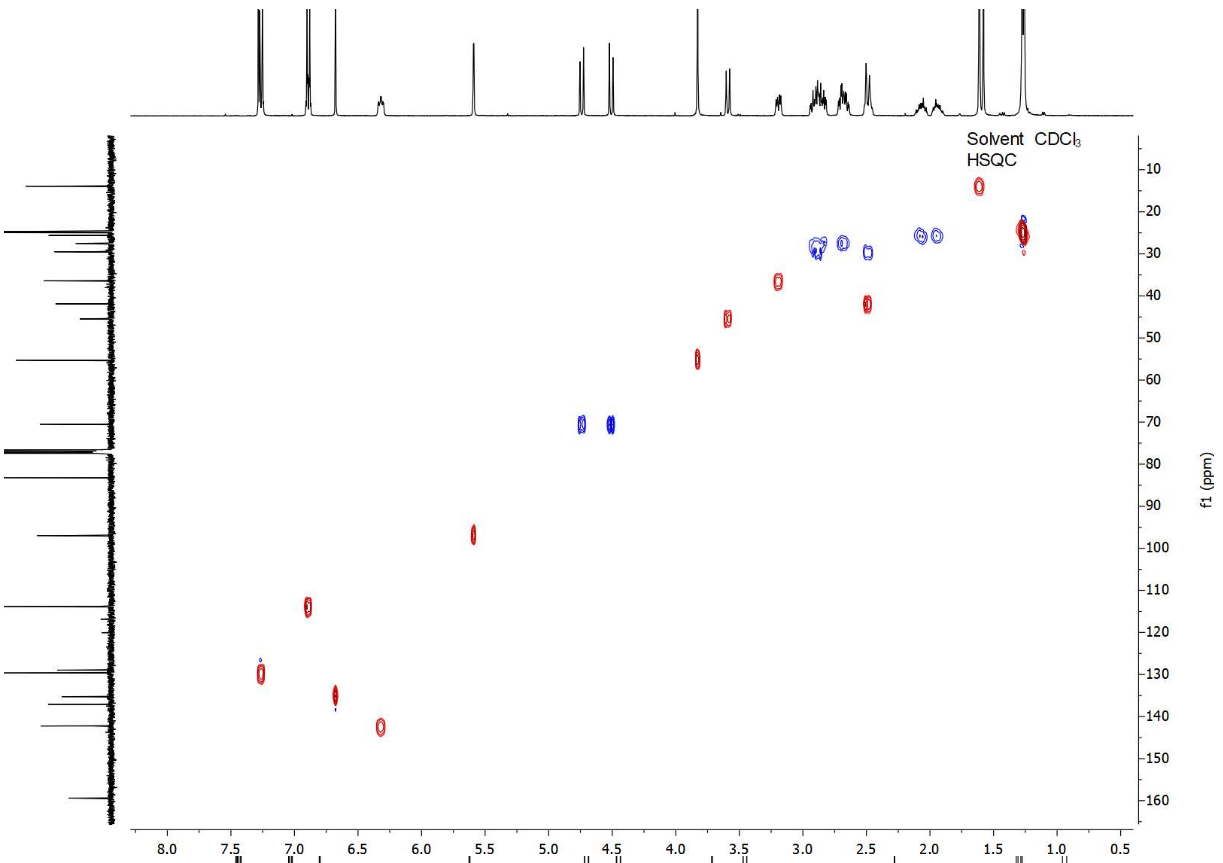
Solvent CDCl3
HSQC
f1 (ppm)

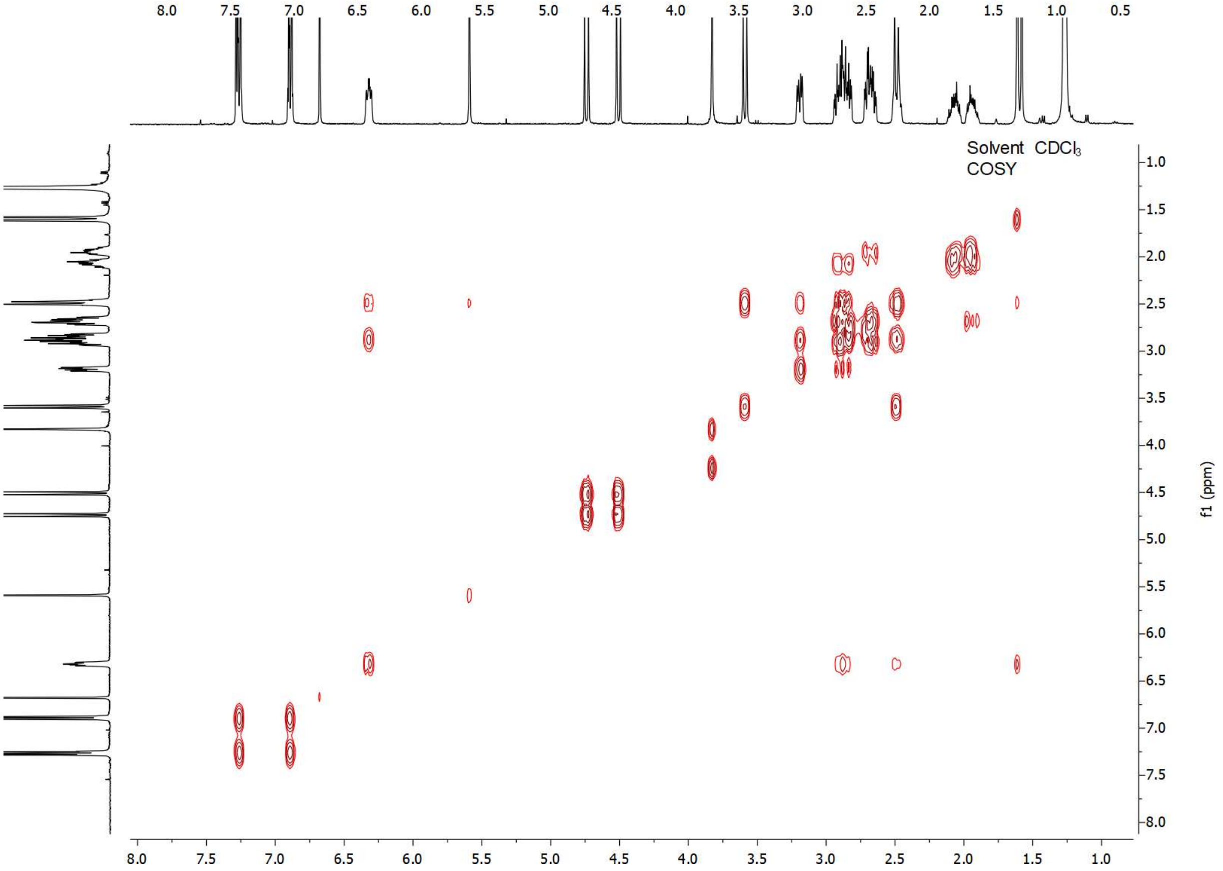
Solvent CDCl3
COSY
f1 (ppm)

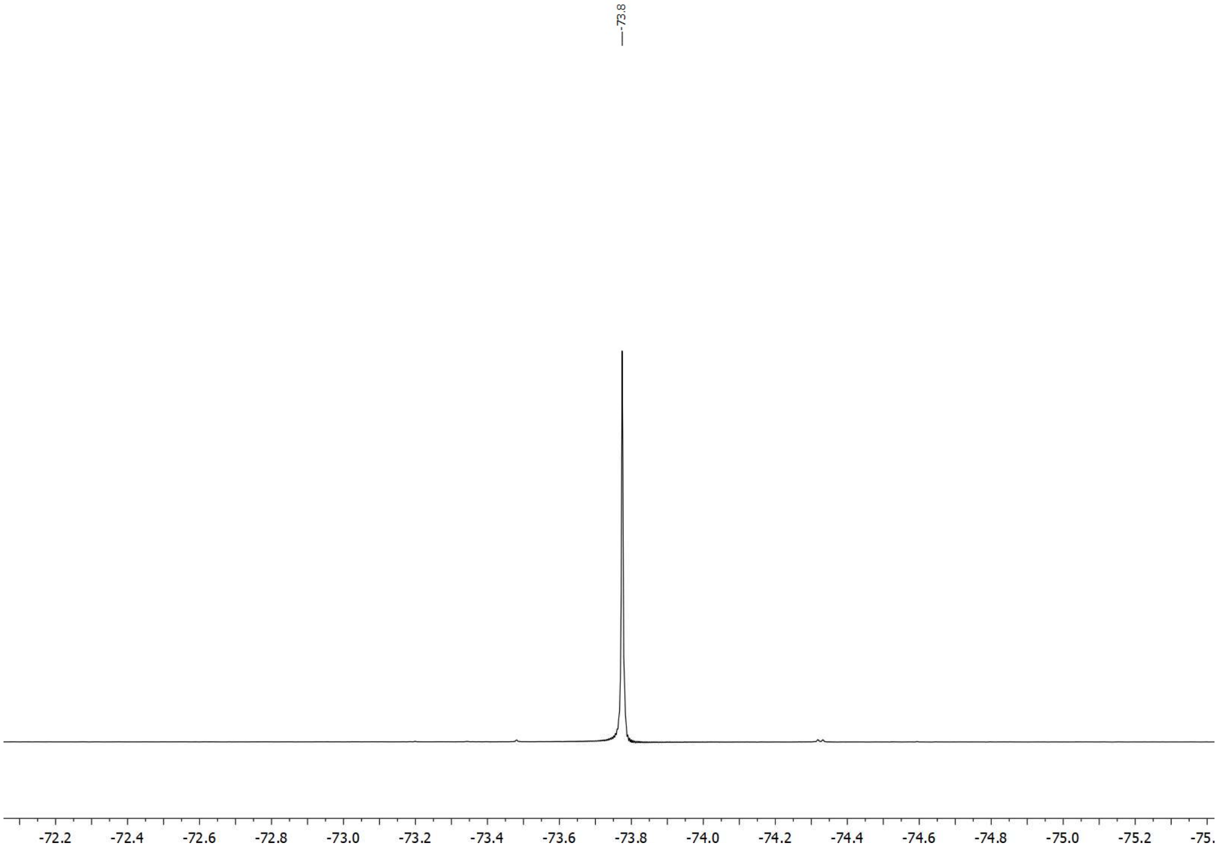
-73.8
-72.2
-72.4
-72.6
-72.8
-73.0
-73.2
-73.4
-73.6
-73.8
-74.0
-74.2
-74.4
-74.6
-74.8
-75.0
-75.2

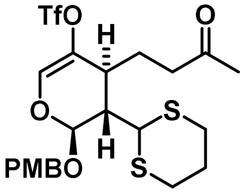
TfO
H
O
O
H
S
PMBO
S

15

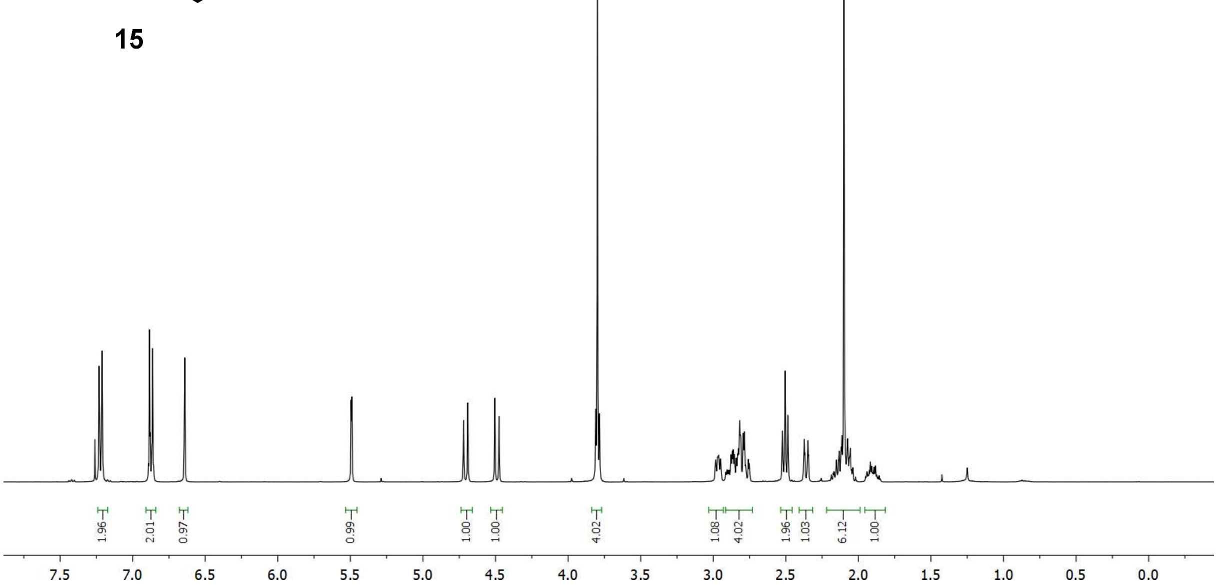
Solvent CDCl3
MHz 400
Nucleus 1H
1.96
2.01
0.97
0.99
1.00
1.00
4.02
1.08
4.02
1.96
1.03
6.12
1.00
7.5 7.0 6.5 6.0 5.5 5.0 4.5 4.0 3.5 3.0 2.5 2.0 1.5 1.0 0.5 0.0

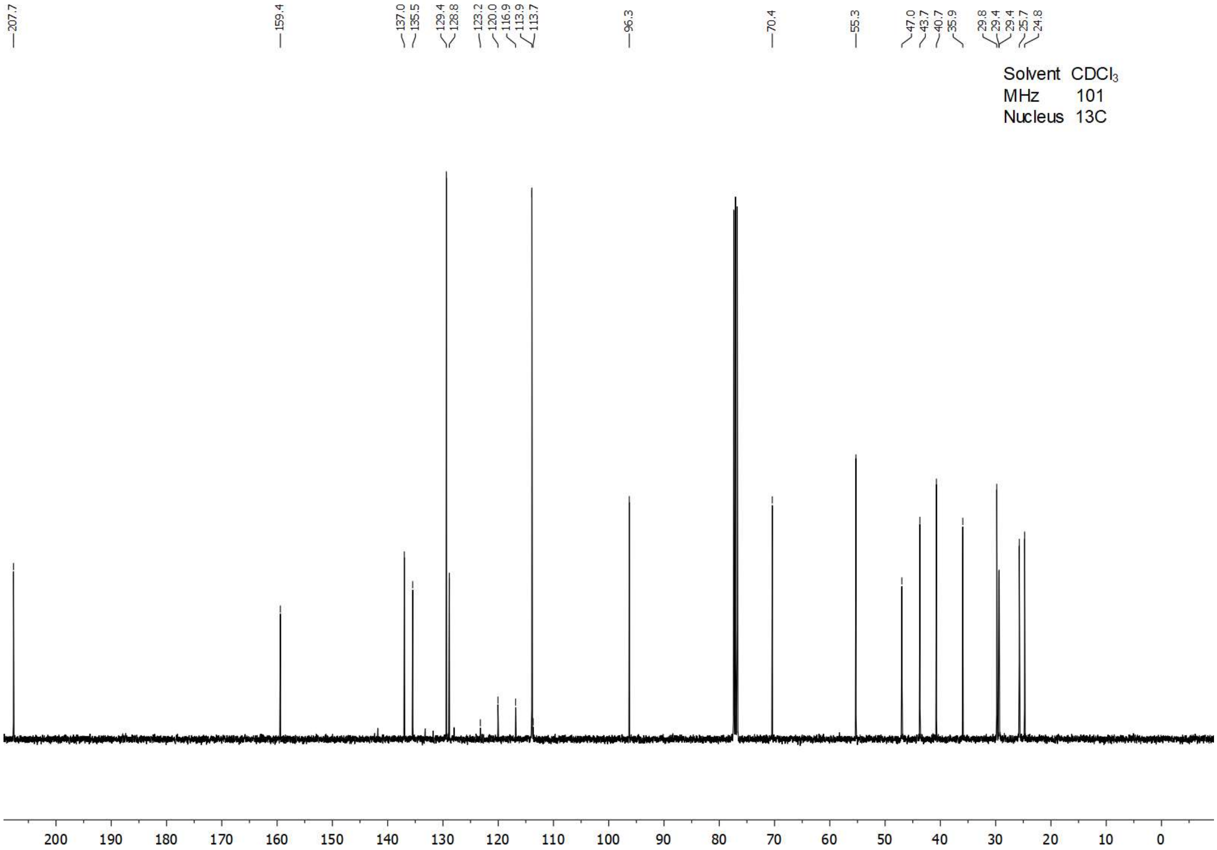
207.7
159.4
137.0
135.5
129.4
128.8
123.2
120.0
116.9
113.9
113.7
96.3
70.4
55.3
47.0
43.7
40.7
35.9
29.8
29.4
29.4
25.7
24.8
Solvent CDCl3
MHz 101
Nucleus 13C
200 190 180 170 160 150 140 130 120 110 100 90 80 70 60 50 40 30 20 10 0

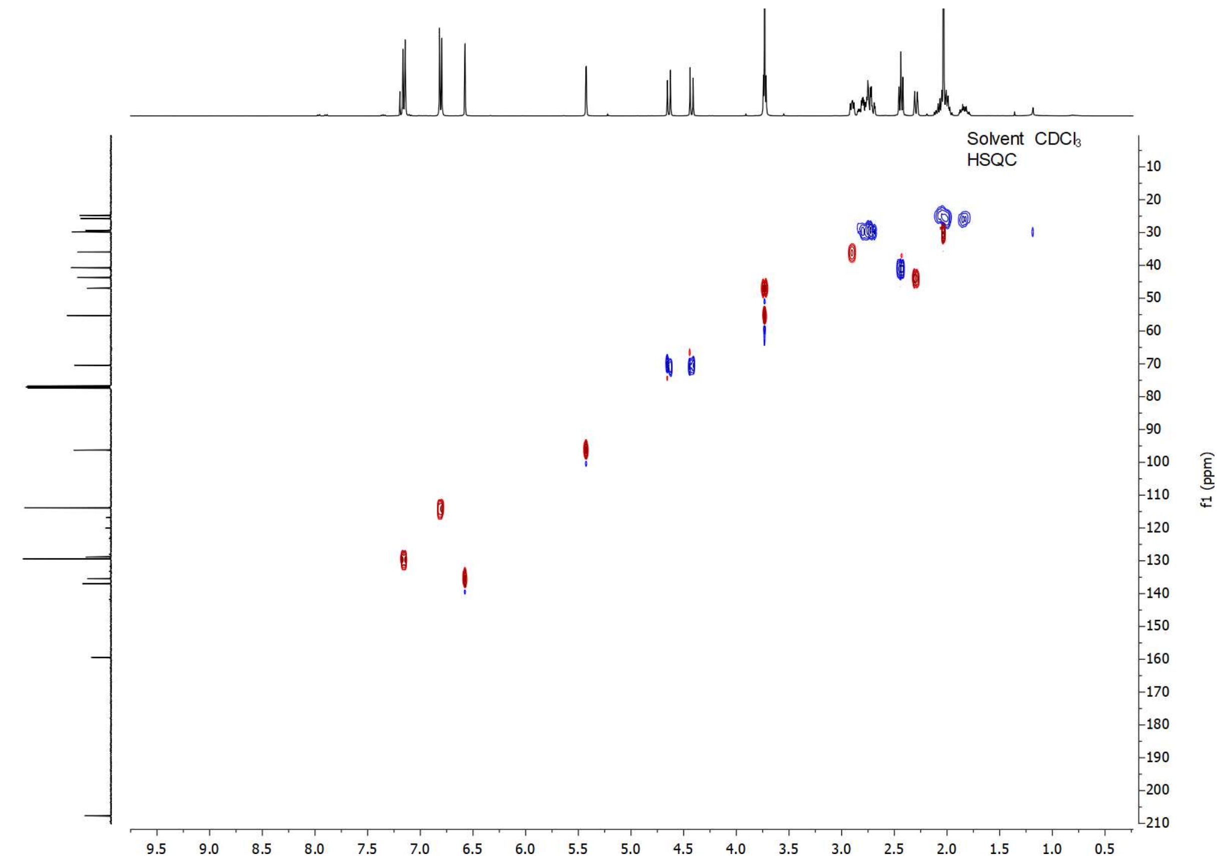

Solvent CDCl3
HSQC
f1 (ppm)

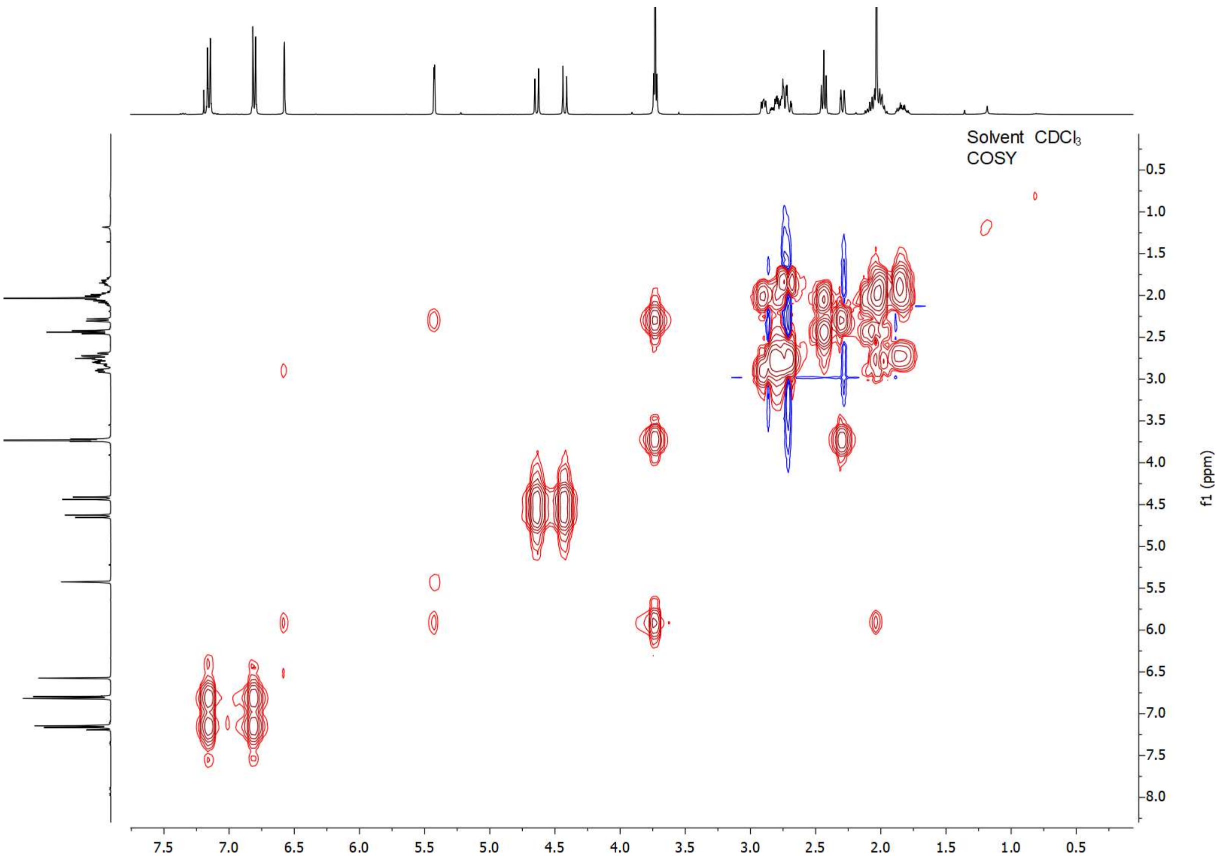

Solvent CDCl3
COSY
f1 (ppm)

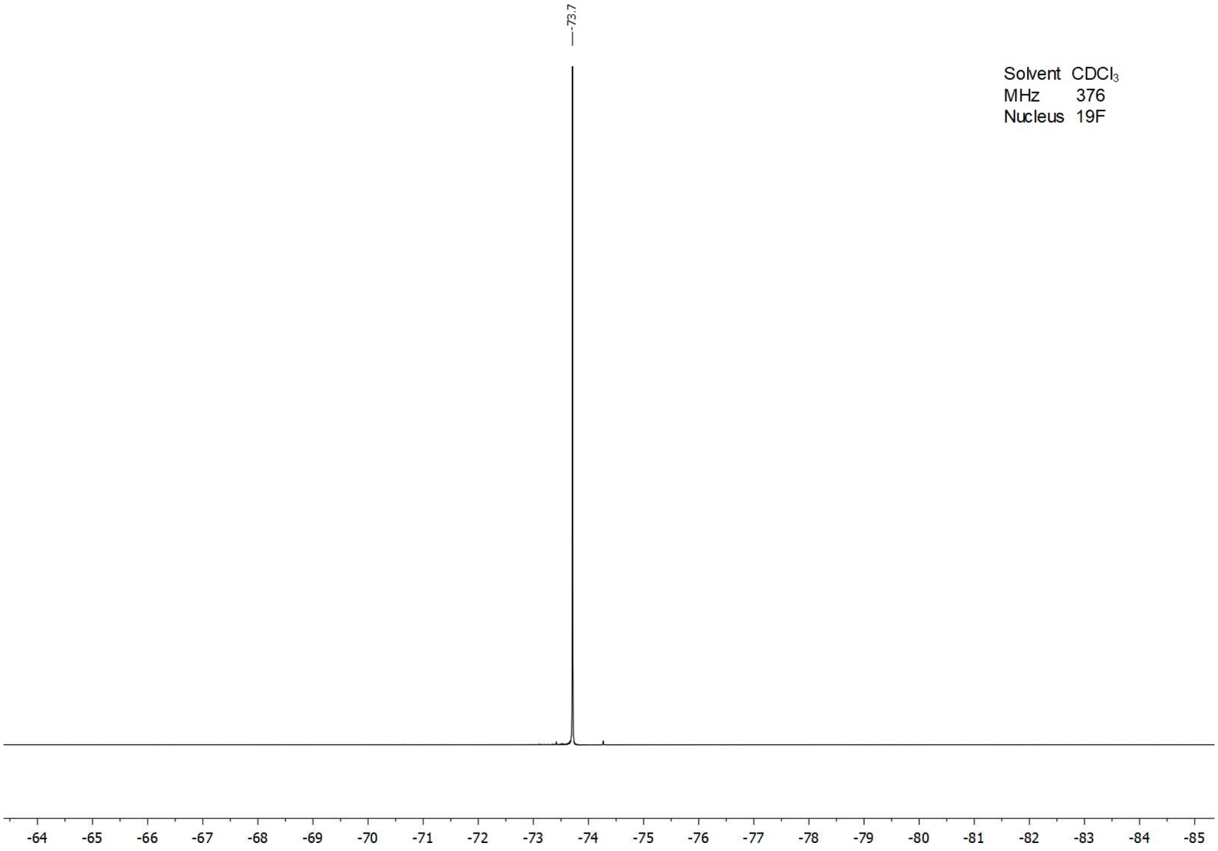
-73.7
Solvent CDCl3
MHz 376
Nucleus 19F
-64
-65
-66
-67
-68
-69
-70
-71
-72
-73
-74
-75
-76
-77
-78
-79
-80
-81
-82
-83
-84
-85

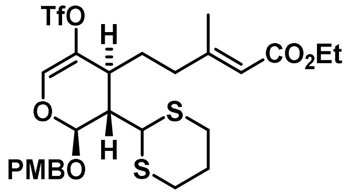
TfO
H
CO2Et
O
S
H
S
PMBO

17

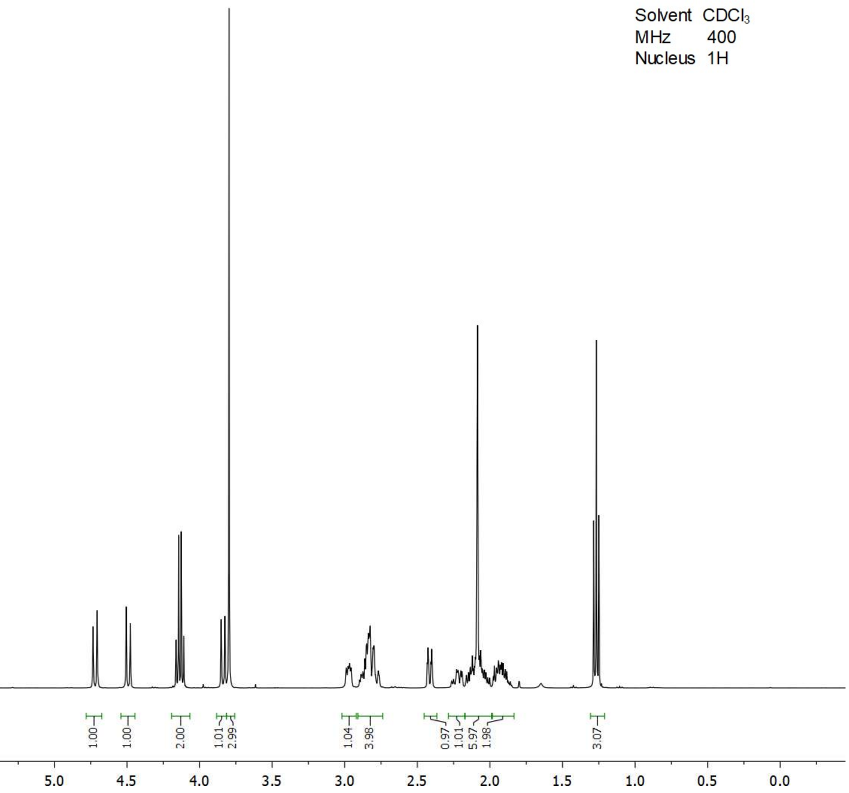
Solvent CDCl3
MHz 400
Nucleus 1H
1.97
1.98
0.97
0.98
0.99
1.00
1.00
2.00
1.01
2.99
1.04
3.98
0.97
1.01
5.97
1.98
3.07
7.5
7.0
6.5
6.0
5.5
5.0
4.5
4.0
3.5
3.0
2.5
2.0
1.5
1.0
0.5
0.0

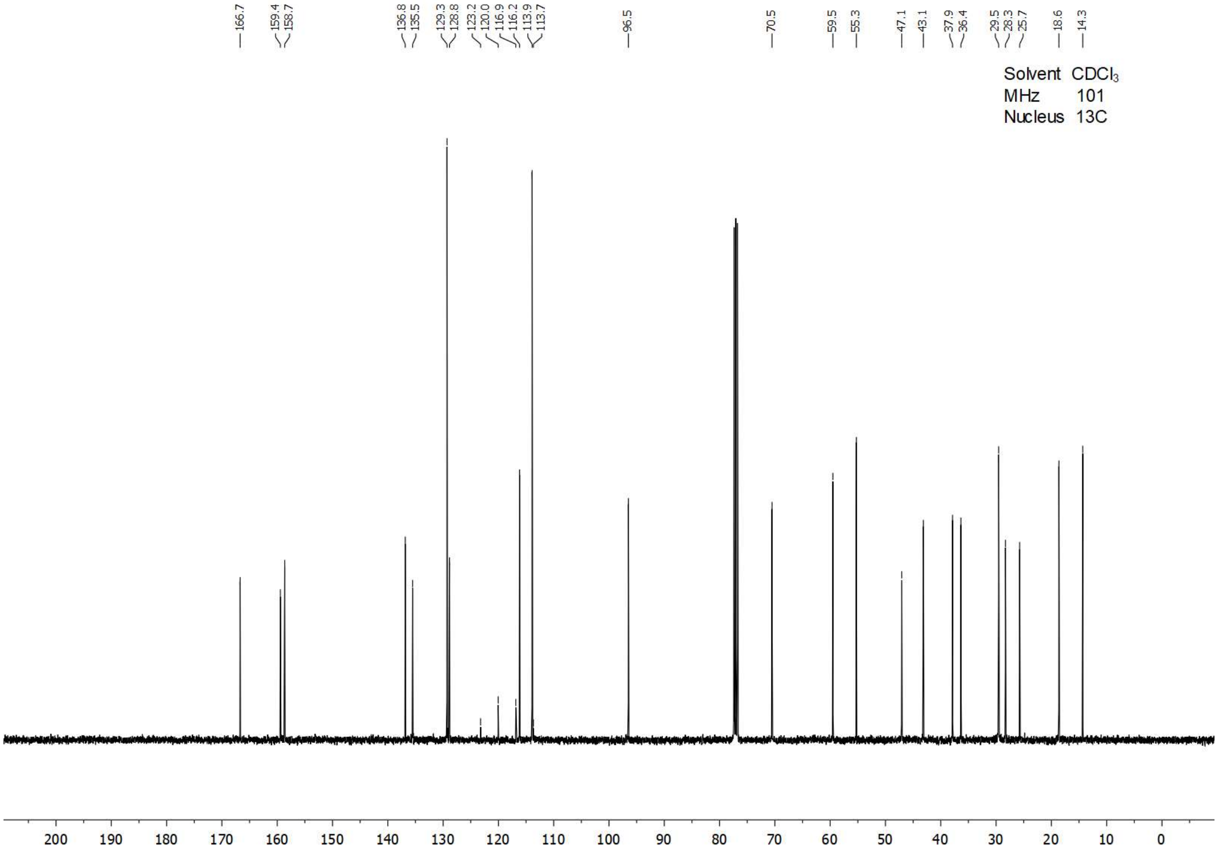
166.7
159.4
158.7
136.8
135.5
129.3
128.8
123.2
120.0
116.9
116.2
113.9
113.7
96.5
70.5
59.5
55.3
47.1
43.1
37.9
36.4
29.5
28.3
25.7
18.6
14.3
Solvent CDCl3
MHz 101
Nucleus 13C
200
190
180
170
160
150
140
130
120
110
100
90
80
70
60
50
40
30
20
10
0

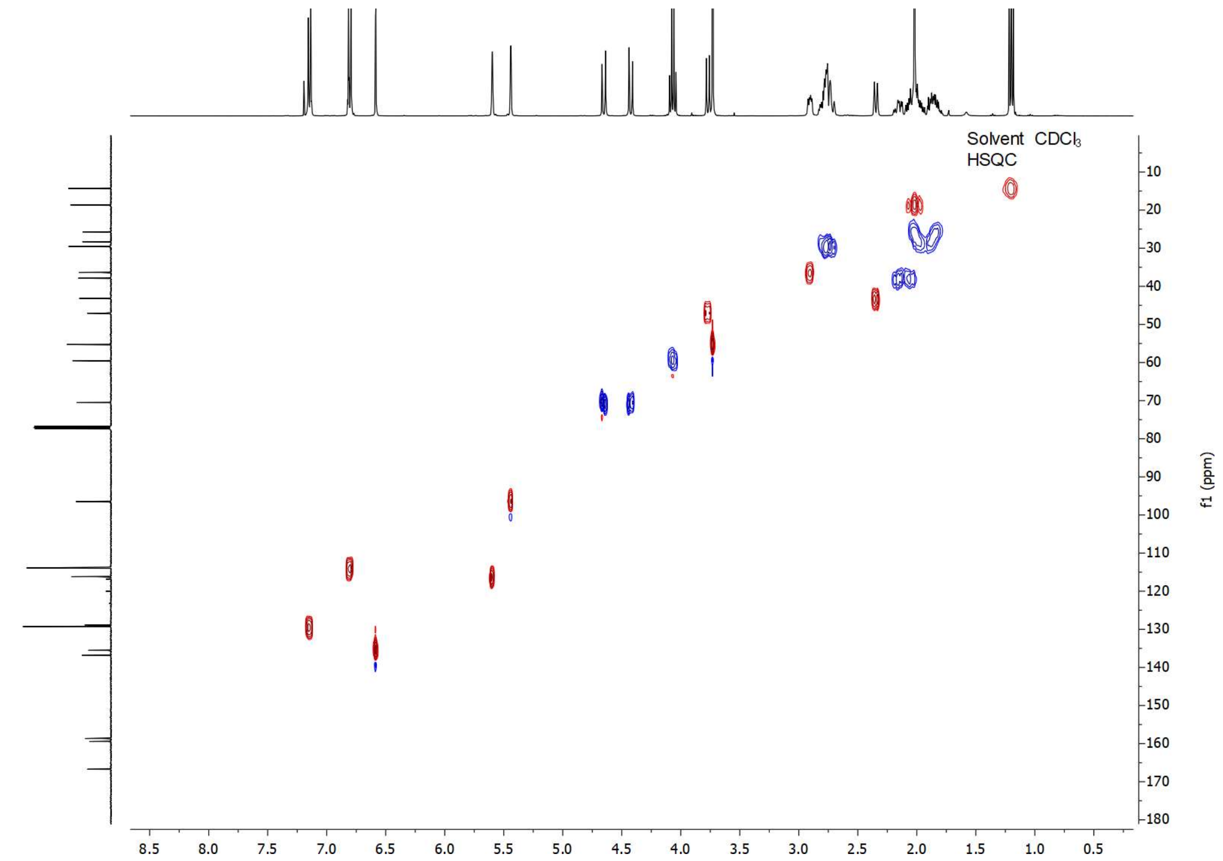

Solvent CDCl3
HSQC
f1 (ppm)

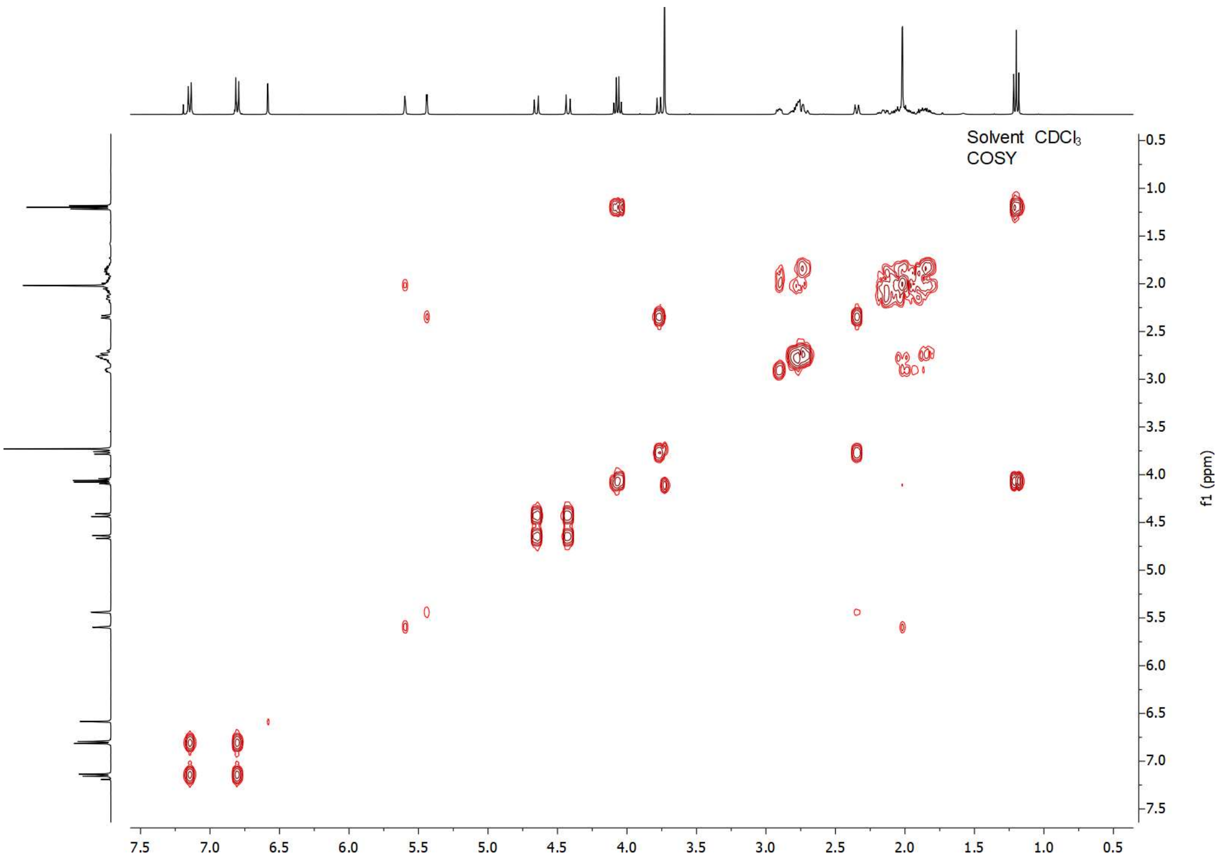

Solvent CDCl3
COSY
f1 (ppm)

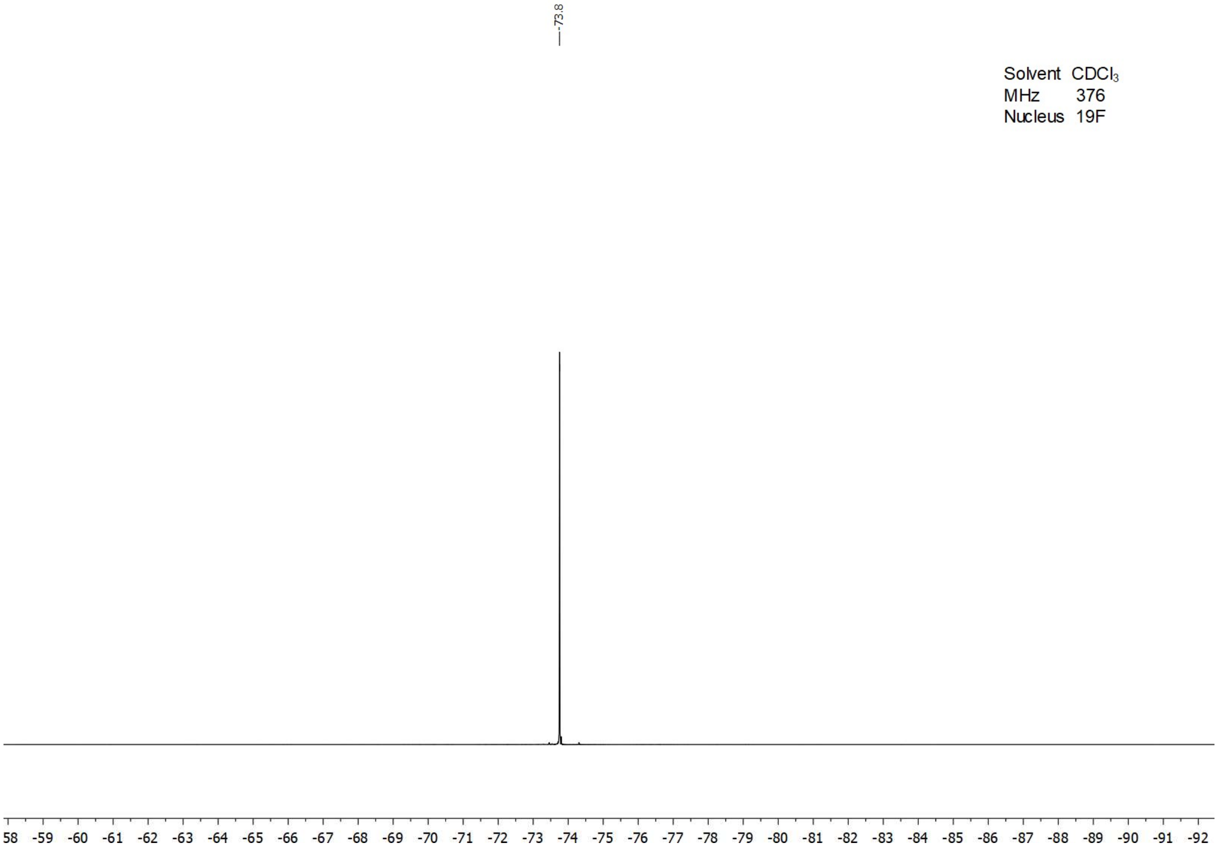
-73.8
Solvent CDCl3
MHz 376
Nucleus 19F
58 -59 -60 -61 -62 -63 -64 -65 -66 -67 -68 -69 -70 -71 -72 -73 -74 -75 -76 -77 -78 -79 -80 -81 -82 -83 -84 -85 -86 -87 -88 -89 -90 -91 -92

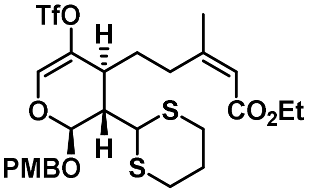
TfO
H
O
H
S
S
CO2Et
PMBO

S7

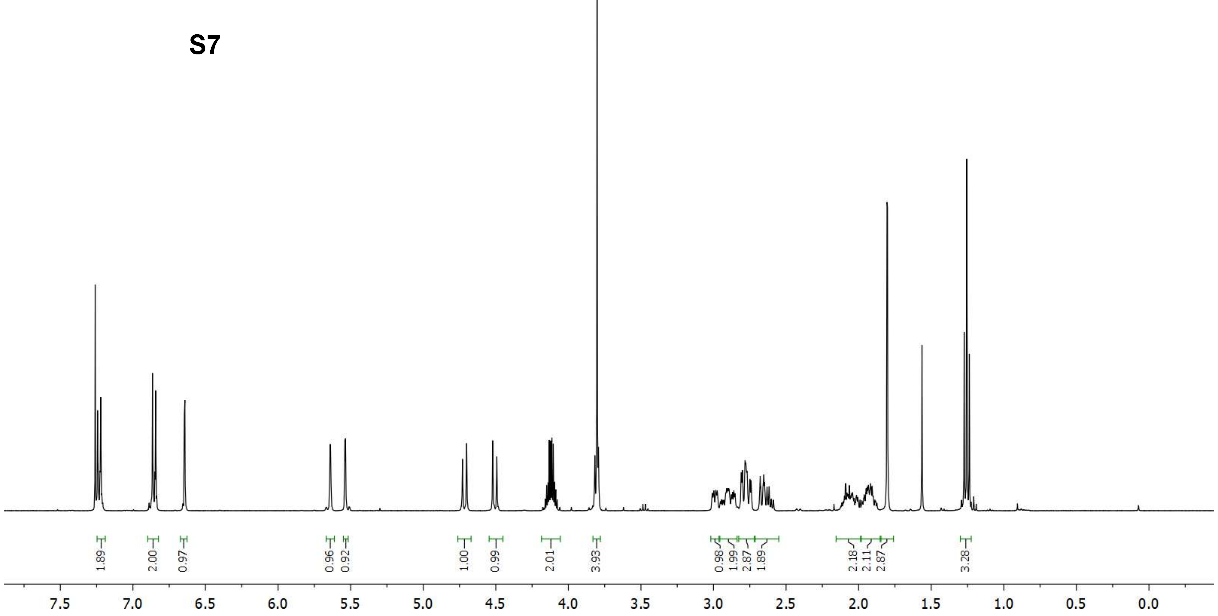
Solvent CDCl3
MHz 400
Nucleus 1H
1.89
2.00
0.97
0.96
0.92
1.00
0.99
2.01
3.93
0.98
1.99
2.87
1.89
2.18
2.11
2.87
3.28
7.5
7.0
6.5
6.0
5.5
5.0
4.5
4.0
3.5
3.0
2.5
2.0
1.5
1.0
0.5
0.0

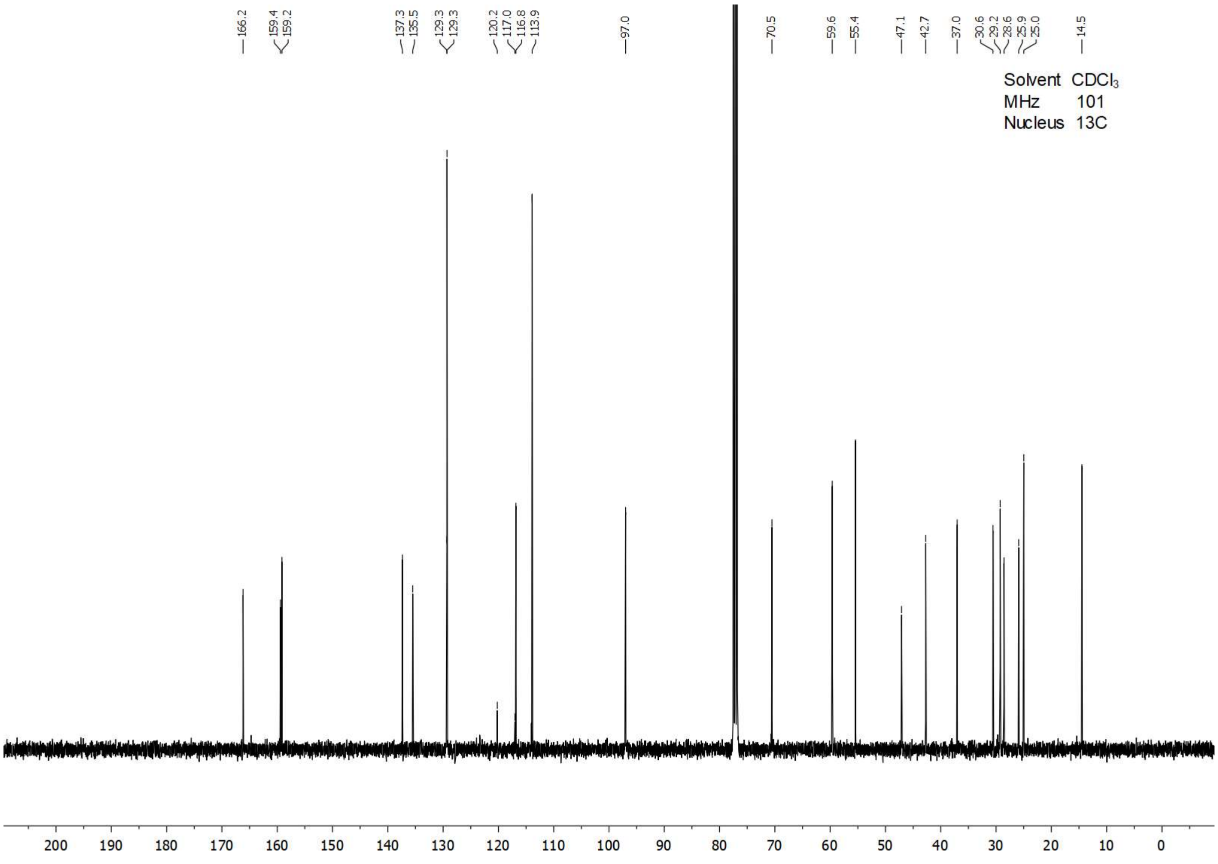
166.2
159.4
159.2
137.3
135.5
129.3
129.3
120.2
117.0
116.8
113.9
97.0
70.5
59.6
55.4
47.1
42.7
37.0
30.6
29.2
28.6
25.9
25.0
14.5
Solvent CDCl3
MHz 101
Nucleus 13C
200
190
180
170
160
150
140
130
120
110
100
90
80
70
60
50
40
30
20
10
0

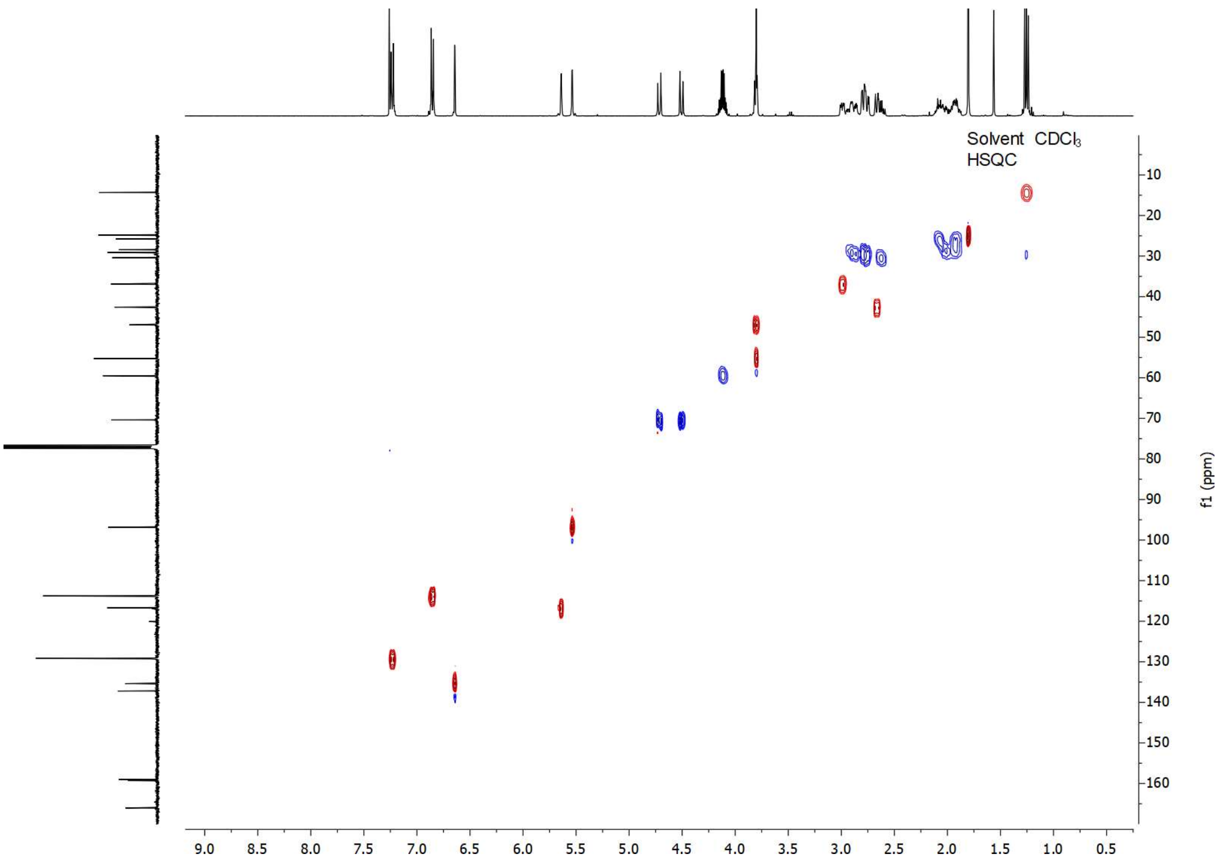
Solvent CDCl3
HSQC
f1 (ppm)

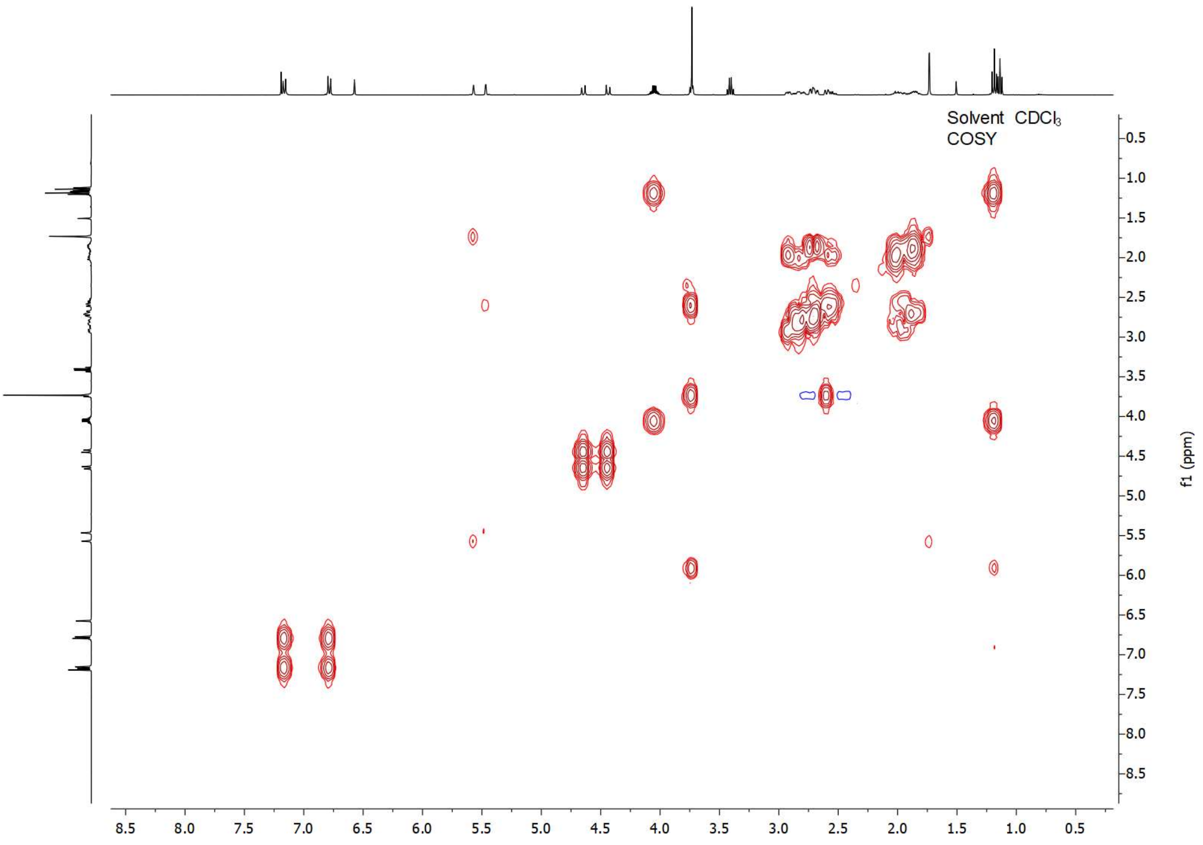
Solvent CDCl3
COSY
f1 (ppm)

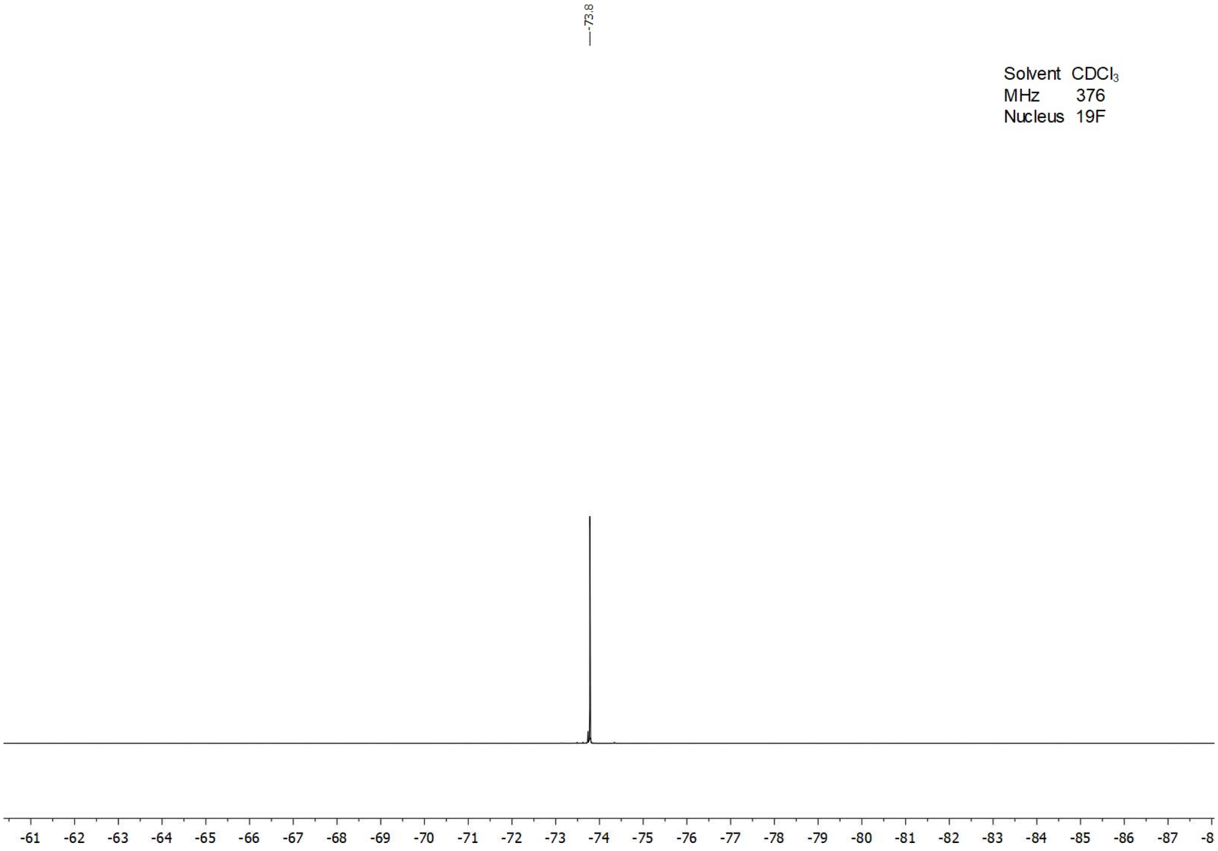
-73.8
Solvent CDCl3
MHz 376
Nucleus 19F
-61 -62 -63 -64 -65 -66 -67 -68 -69 -70 -71 -72 -73 -74 -75 -76 -77 -78 -79 -80 -81 -82 -83 -84 -85 -86 -87 -8

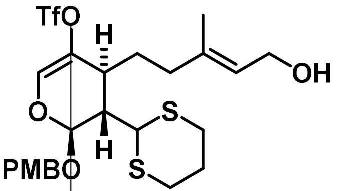
TfO
H
OH
O
S
H
S
PMBO

S8

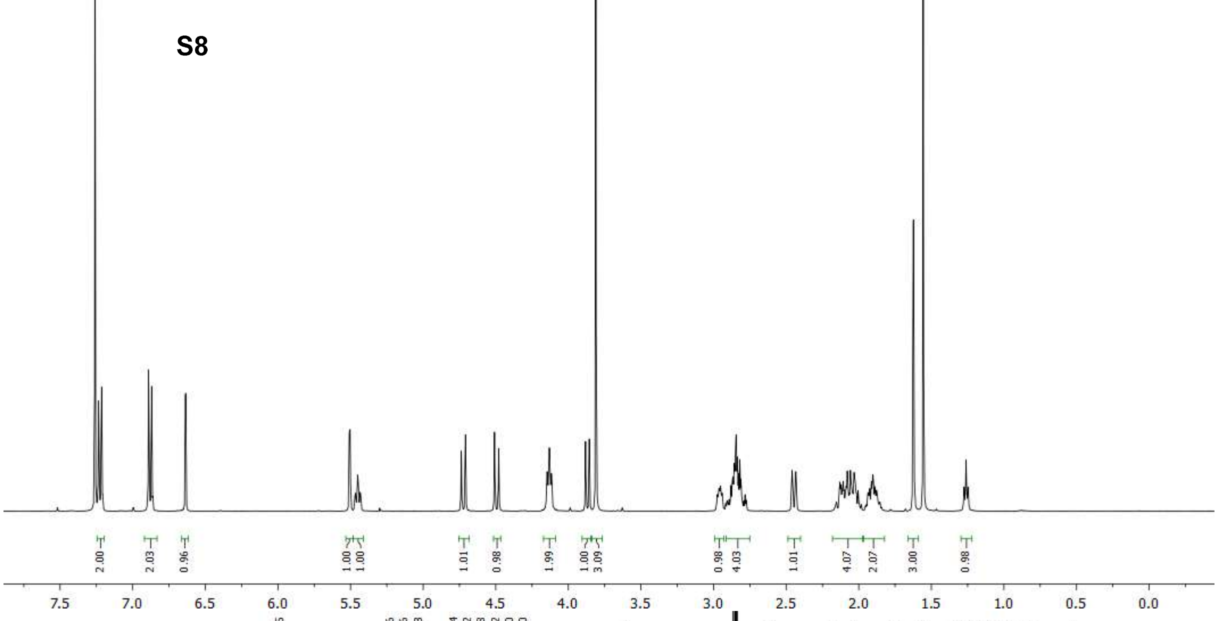
Solvent CDCl3
MHz 400
Nucleus 1H
2.00
2.03
0.96
1.00
1.00
1.01
0.98
1.99
1.00
3.09
0.98
4.03
1.01
4.07
2.07
3.00
0.98
7.5
7.0
6.5
6.0
5.5
5.0
4.5
4.0
3.5
3.0
2.5
2.0
1.5
1.0
0.5
0.0

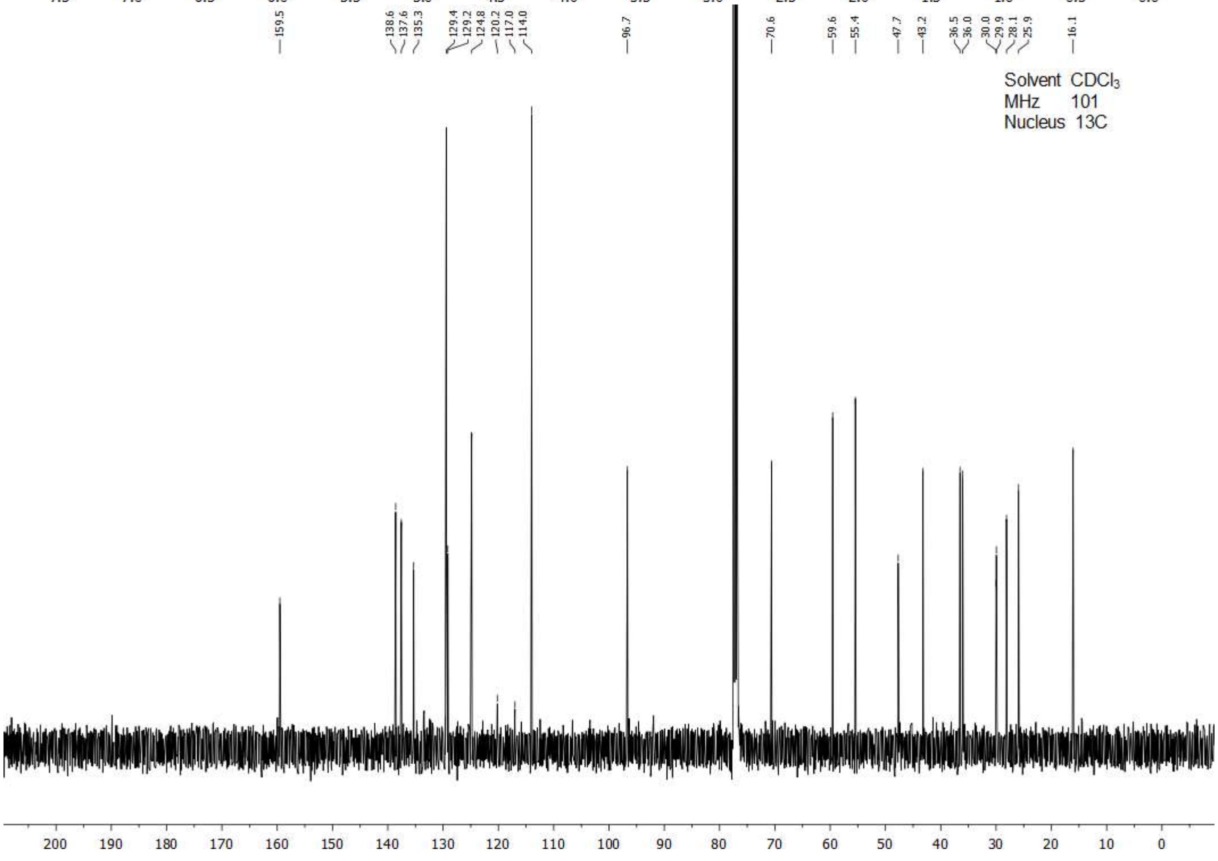
159.5
138.6
137.6
135.3
129.4
129.2
124.8
120.2
117.0
114.0
96.7
70.6
59.6
55.4
47.7
43.2
36.5
36.0
30.0
29.9
28.1
25.9
16.1
Solvent CDCl3
MHz 101
Nucleus 13C
200
190
180
170
160
150
140
130
120
110
100
90
80
70
60
50
40
30
20
10
0

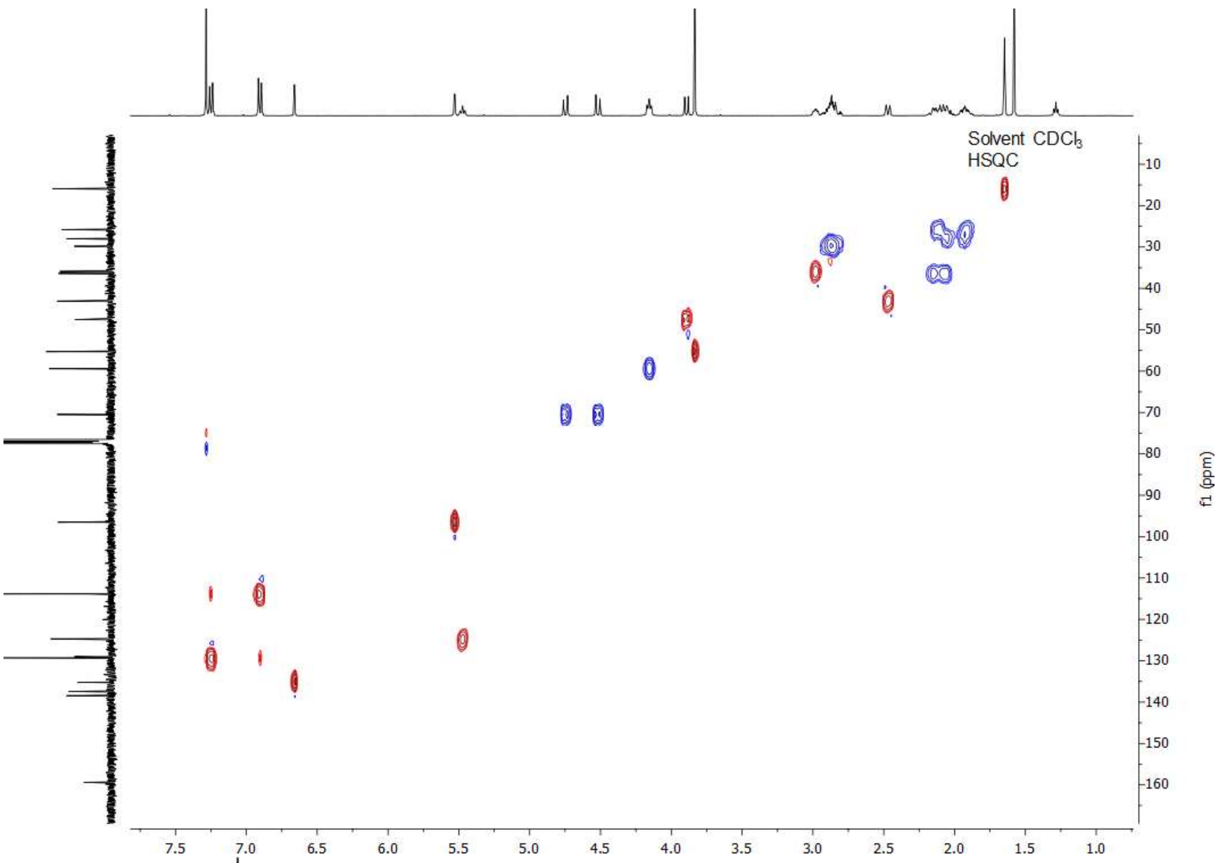
Solvent CDCl3
HSQC
f1 (ppm)

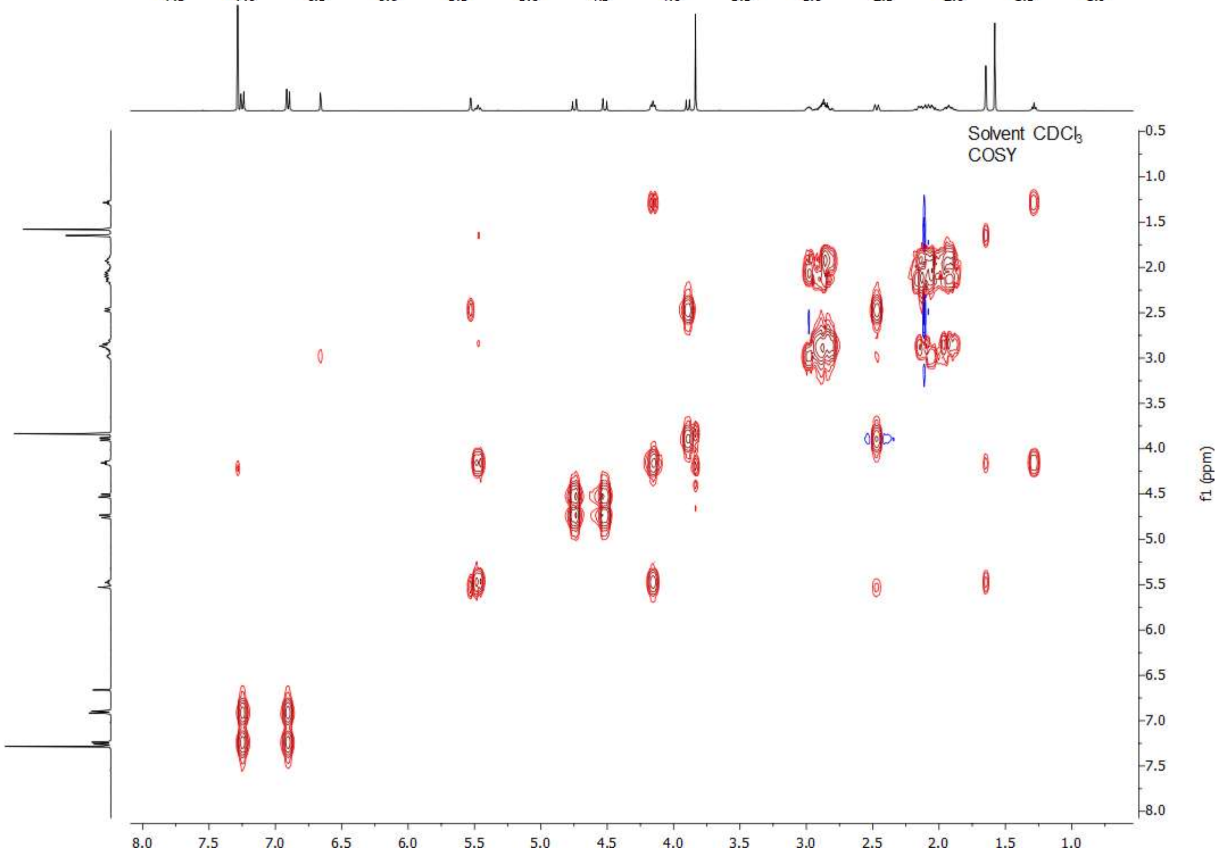
Solvent CDCl3
COSY
f1 (ppm)

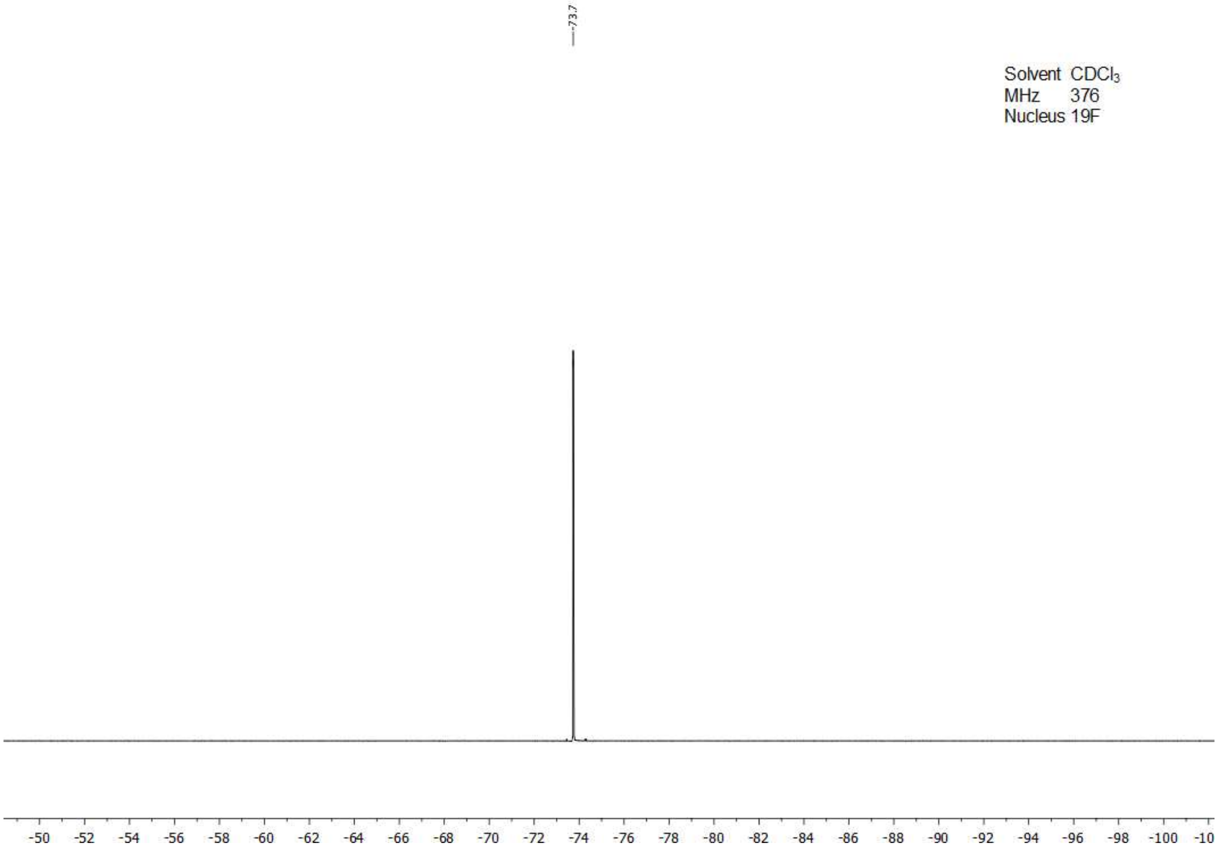
-73.7
Solvent CDCl3
MHz 376
Nucleus 19F
-50 -52 -54 -56 -58 -60 -62 -64 -66 -68 -70 -72 -74 -76 -78 -80 -82 -84 -86 -88 -90 -92 -94 -96 -98 -100 -10

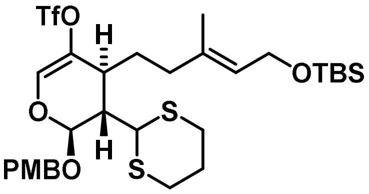
TfO
H
OTBS
O
S
PMBO
H
S

18

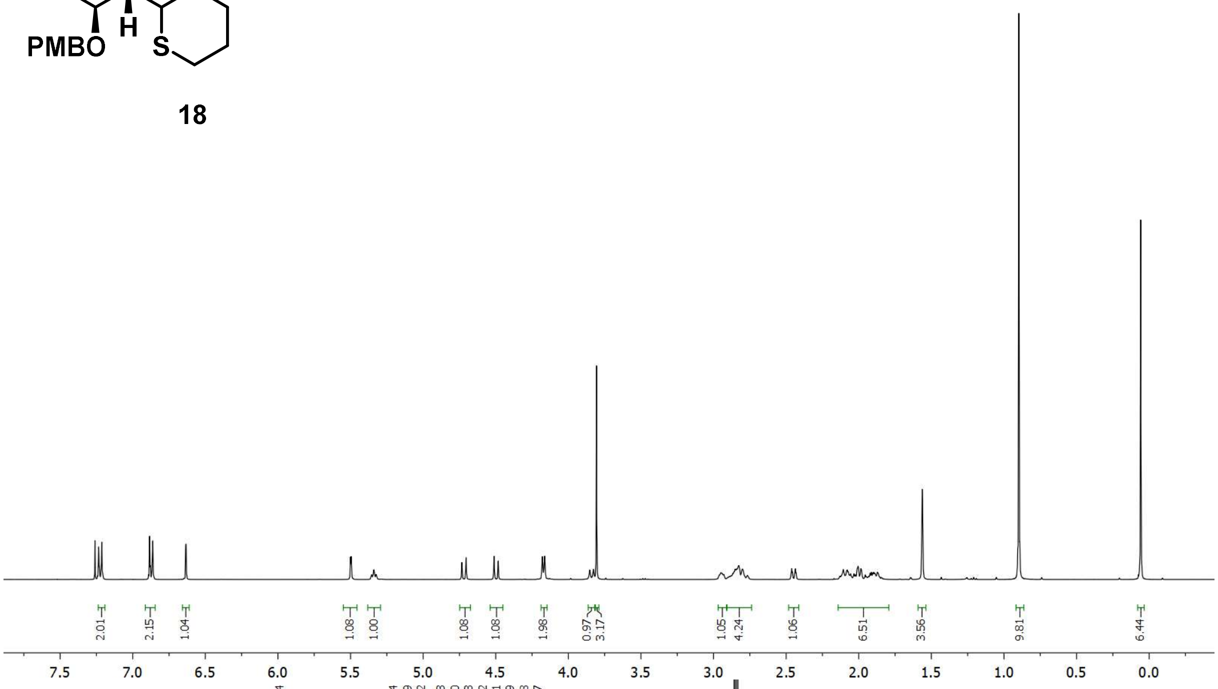
Solvent CDCl$_3$
MHz 400
Nucleus 1H

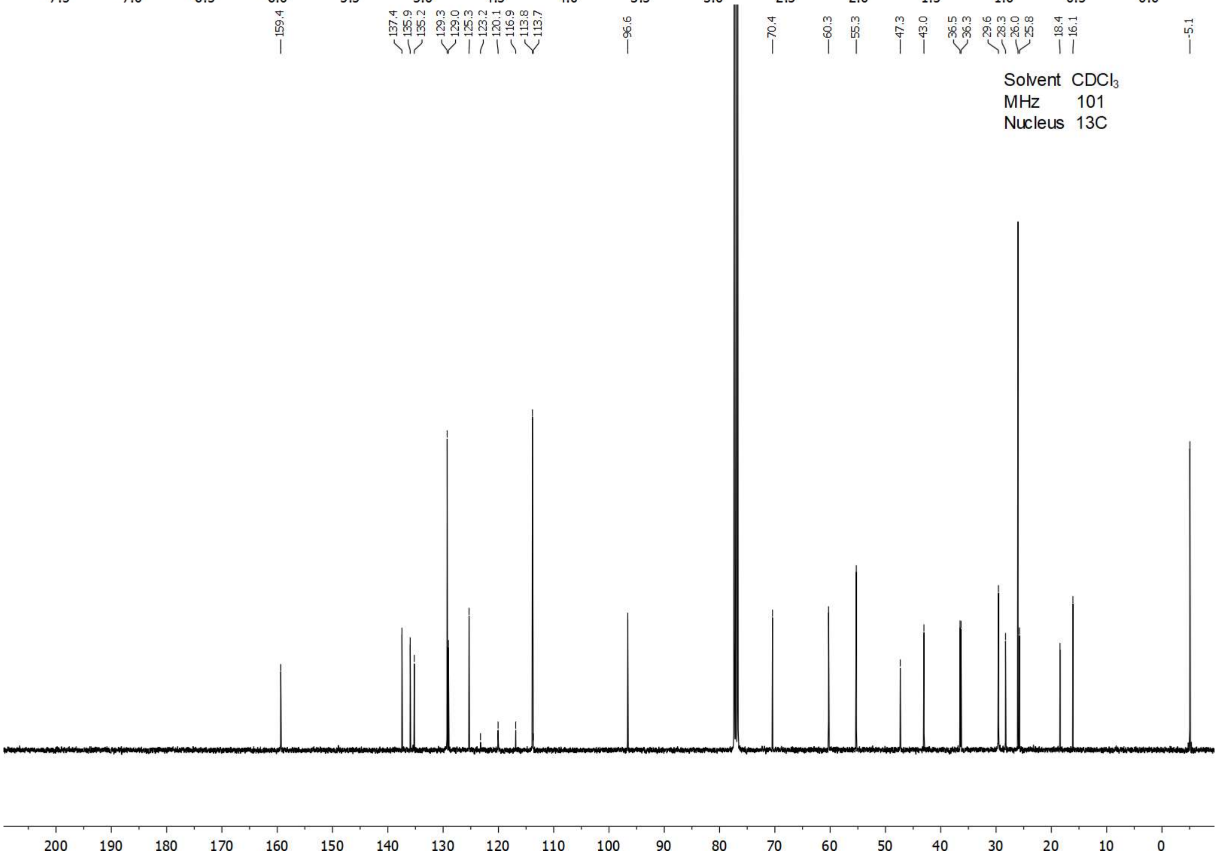
Solvent CDCl$_3$
MHz 101
Nucleus 13C

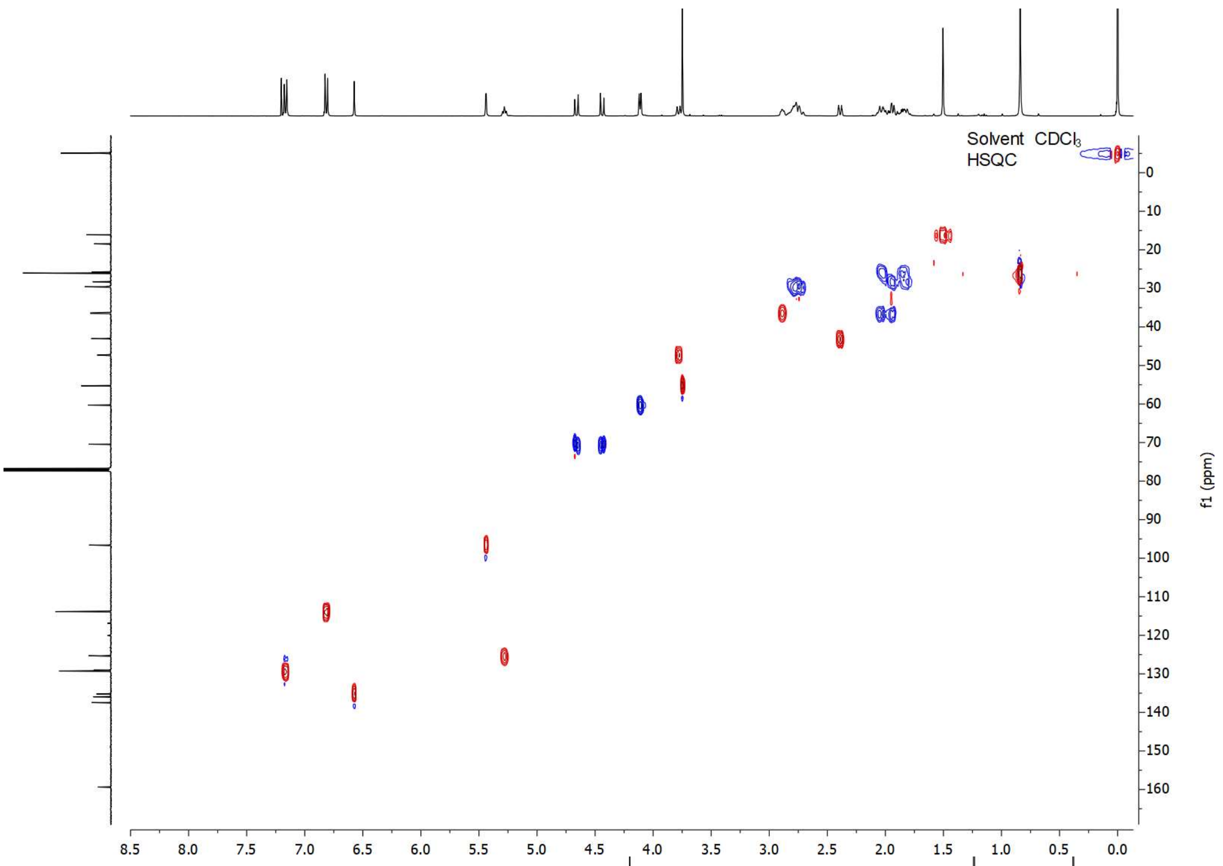
Solvent CDCl3
HSQC
f1 (ppm)

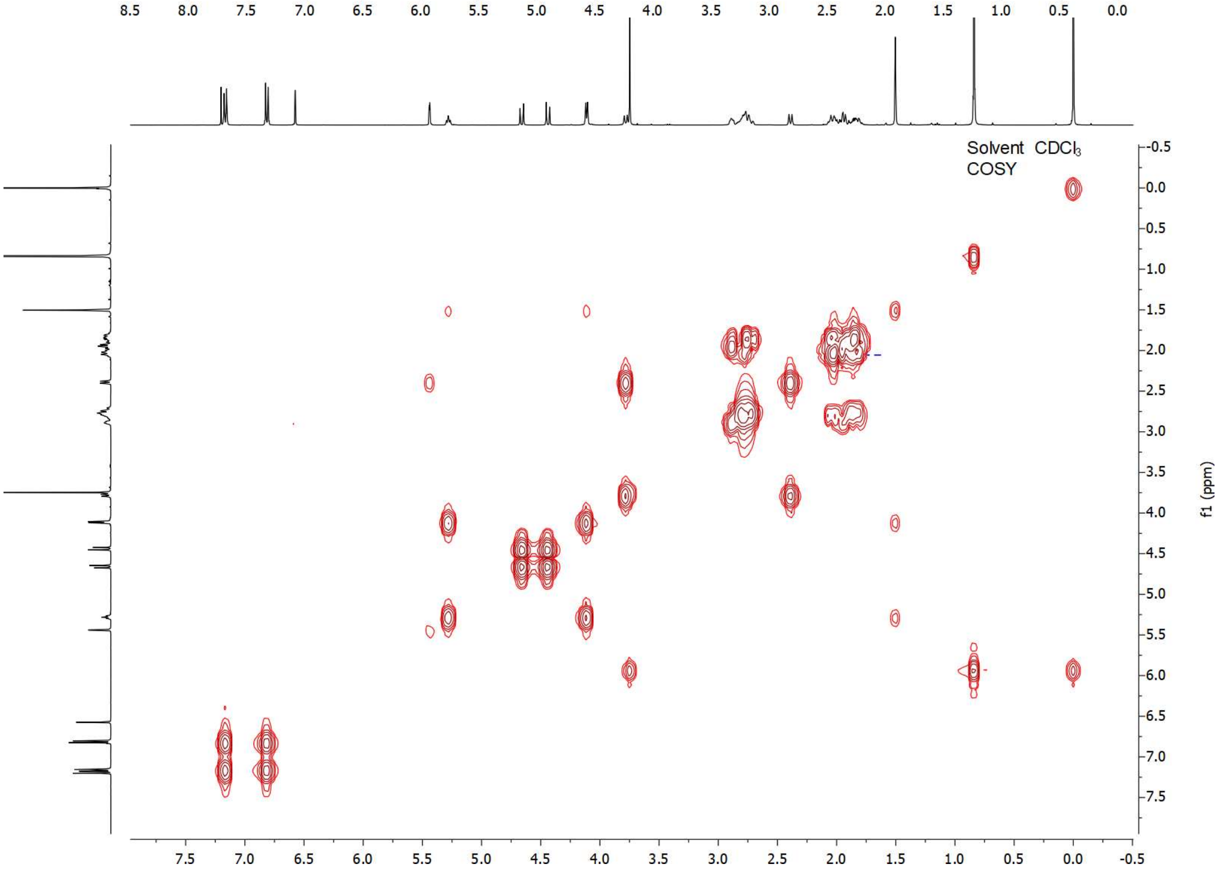
Solvent CDCl3
COSY
f1 (ppm)

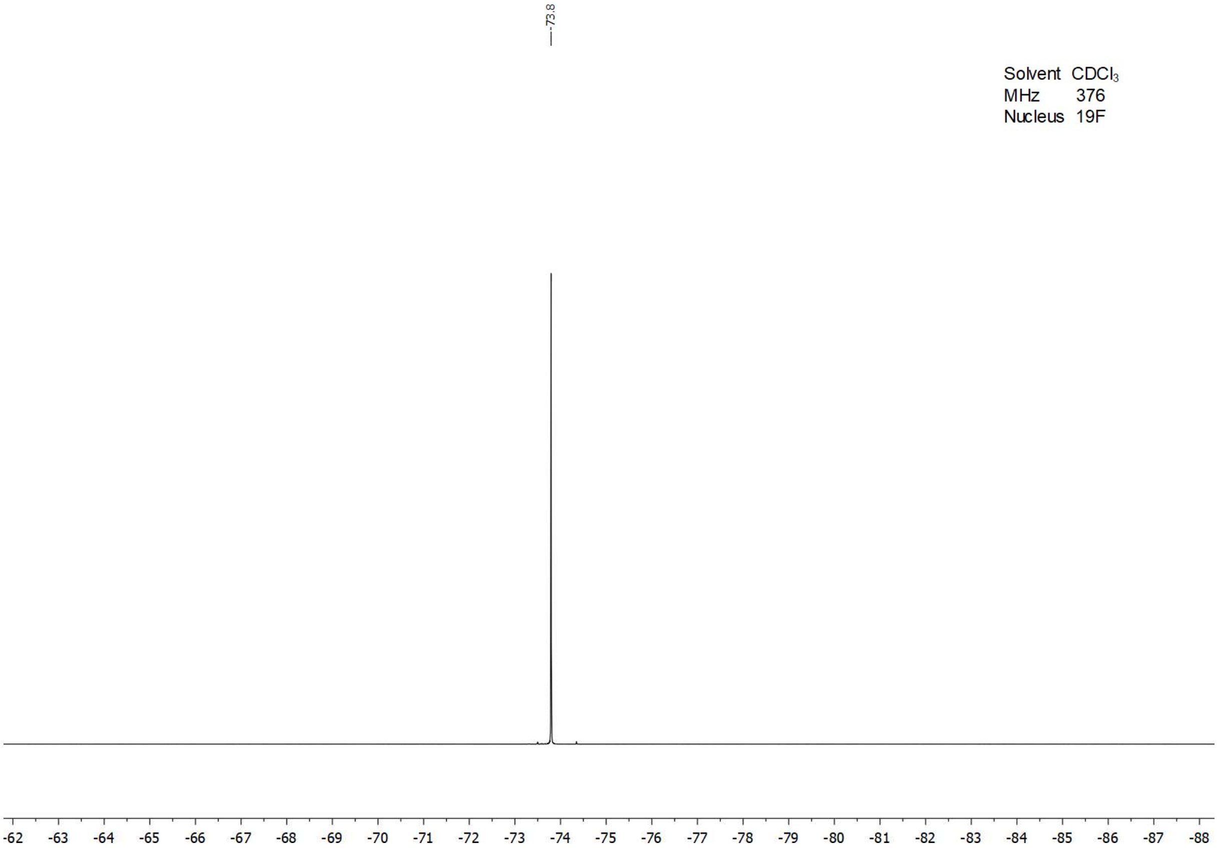
-73.8
Solvent CDCl3
MHz 376
Nucleus 19F
-62 -63 -64 -65 -66 -67 -68 -69 -70 -71 -72 -73 -74 -75 -76 -77 -78 -79 -80 -81 -82 -83 -84 -85 -86 -87 -88

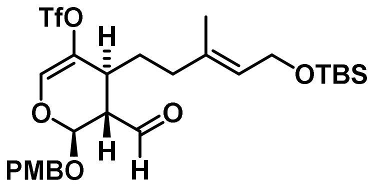

S9

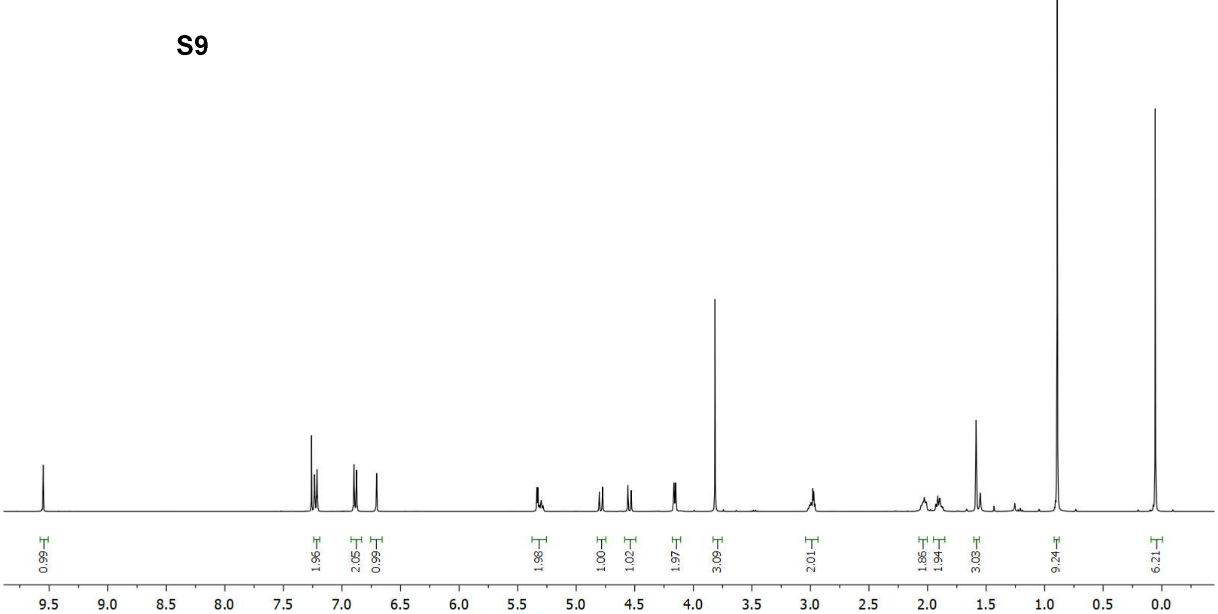
Solvent CDCl$_3$
MHz 400
Nucleus 1H

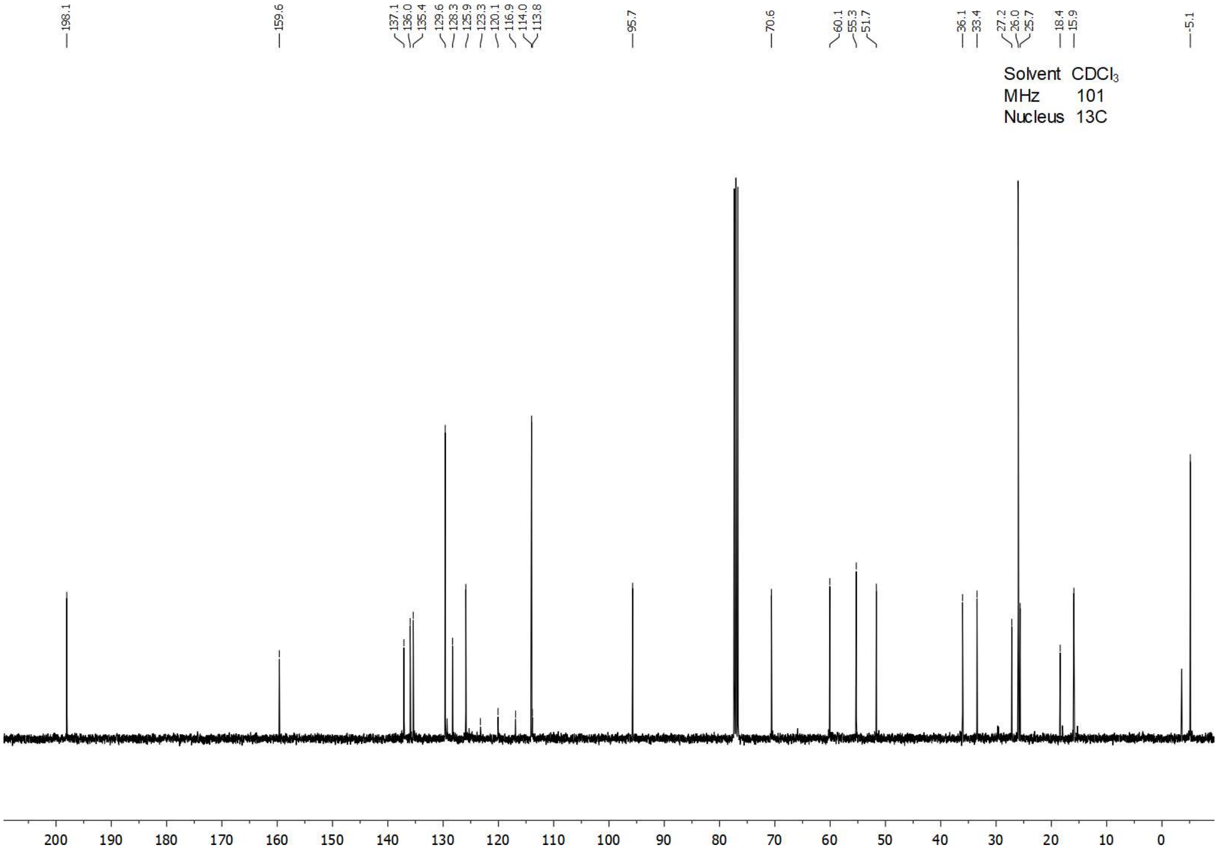
Solvent CDCl$_3$
MHz 101
Nucleus 13C

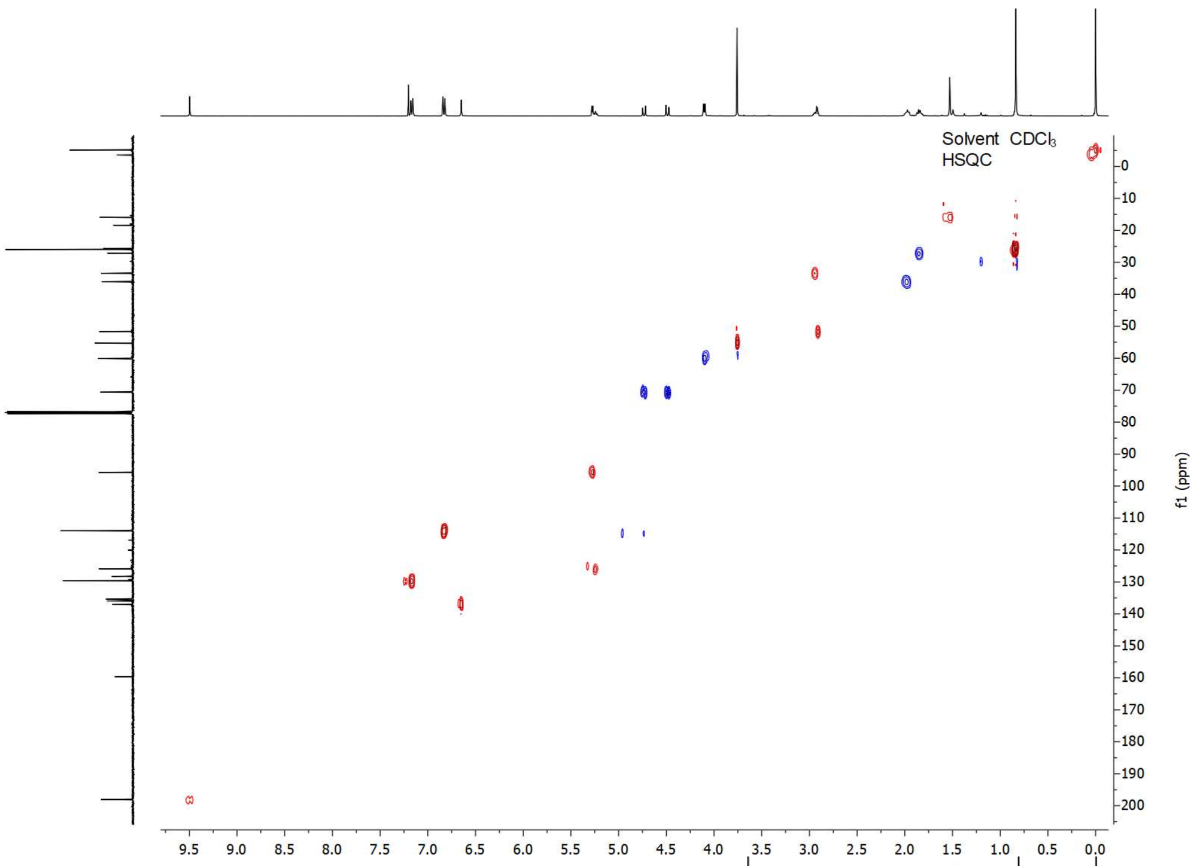

Solvent CDCl3
HSQC
f1 (ppm)

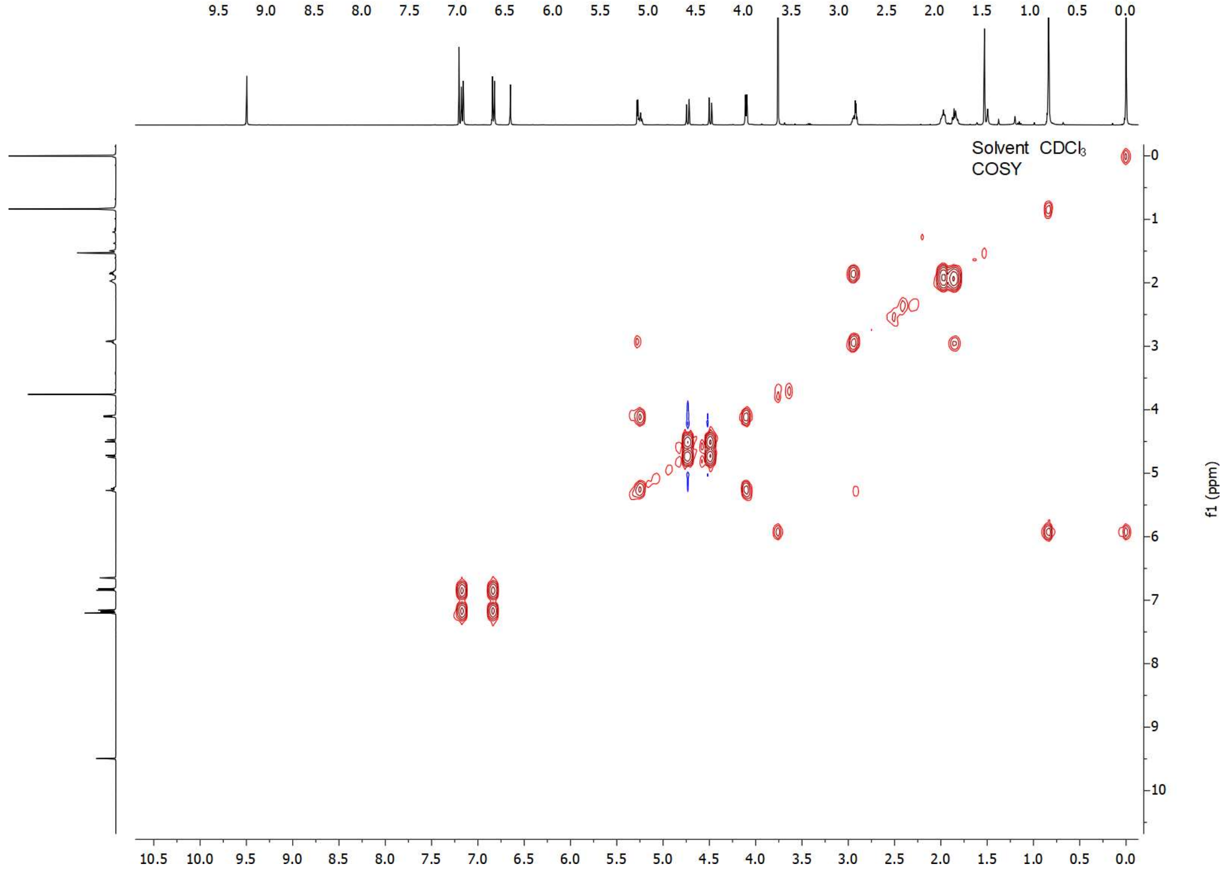

Solvent CDCl3
COSY
f1 (ppm)

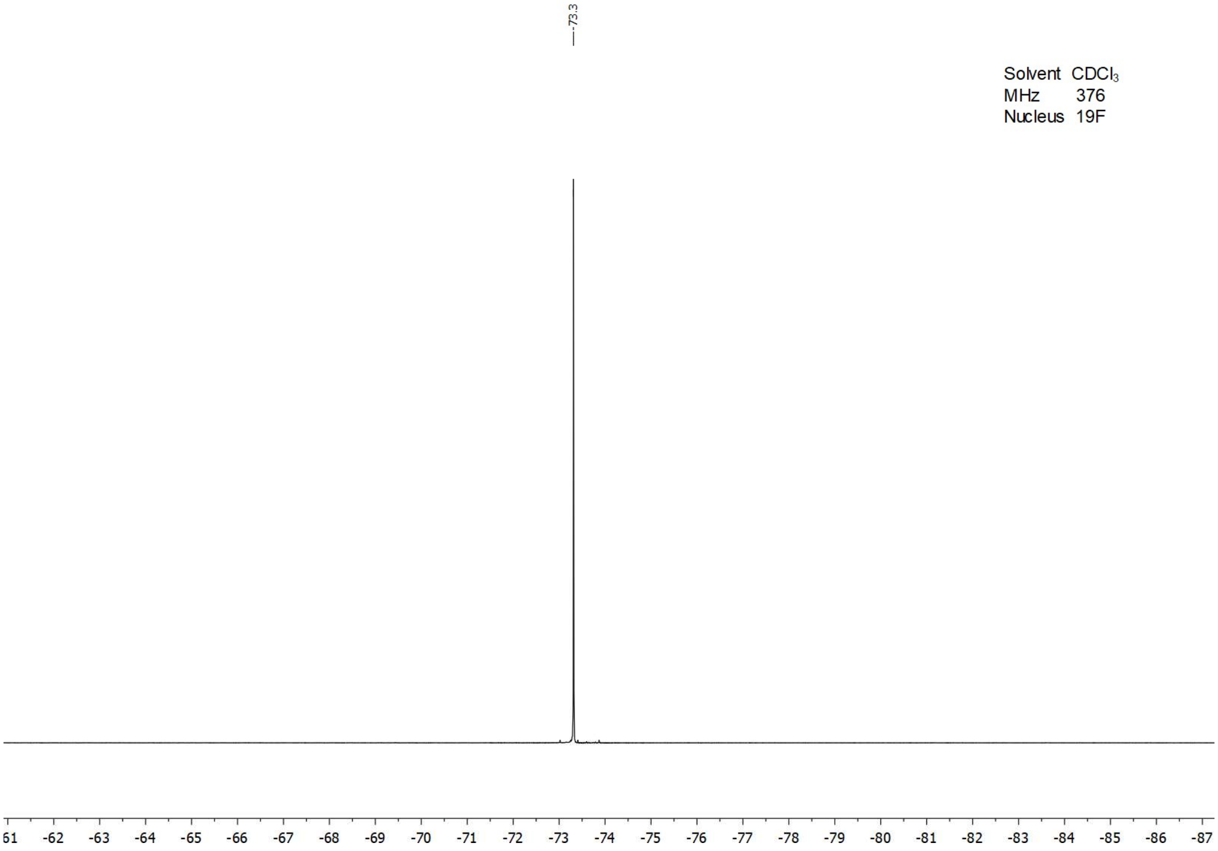
-73.3
Solvent CDCl3
MHz 376
Nucleus 19F
61 -62 -63 -64 -65 -66 -67 -68 -69 -70 -71 -72 -73 -74 -75 -76 -77 -78 -79 -80 -81 -82 -83 -84 -85 -86 -87

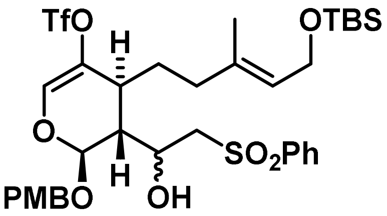
TfO
H
OTBS
O
SO2Ph
H
PMBO
OH

19a/b

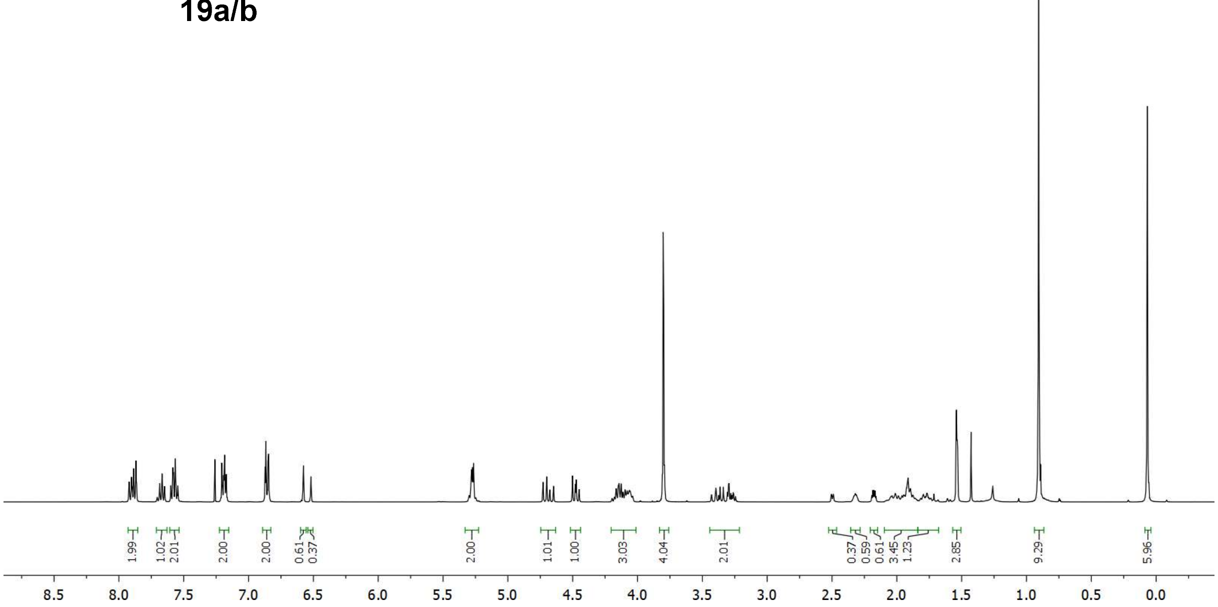
Solvent CDCl3
MHz 400
Nucleus 1H
1.99
1.02
2.01
2.00
2.00
0.61
0.37
2.00
1.01
1.00
3.03
4.04
2.01
0.37
0.59
0.61
3.45
1.23
2.85
9.29
5.96
8.5
8.0
7.5
7.0
6.5
6.0
5.5
5.0
4.5
4.0
3.5
3.0
2.5
2.0
1.5
1.0
0.5
0.0

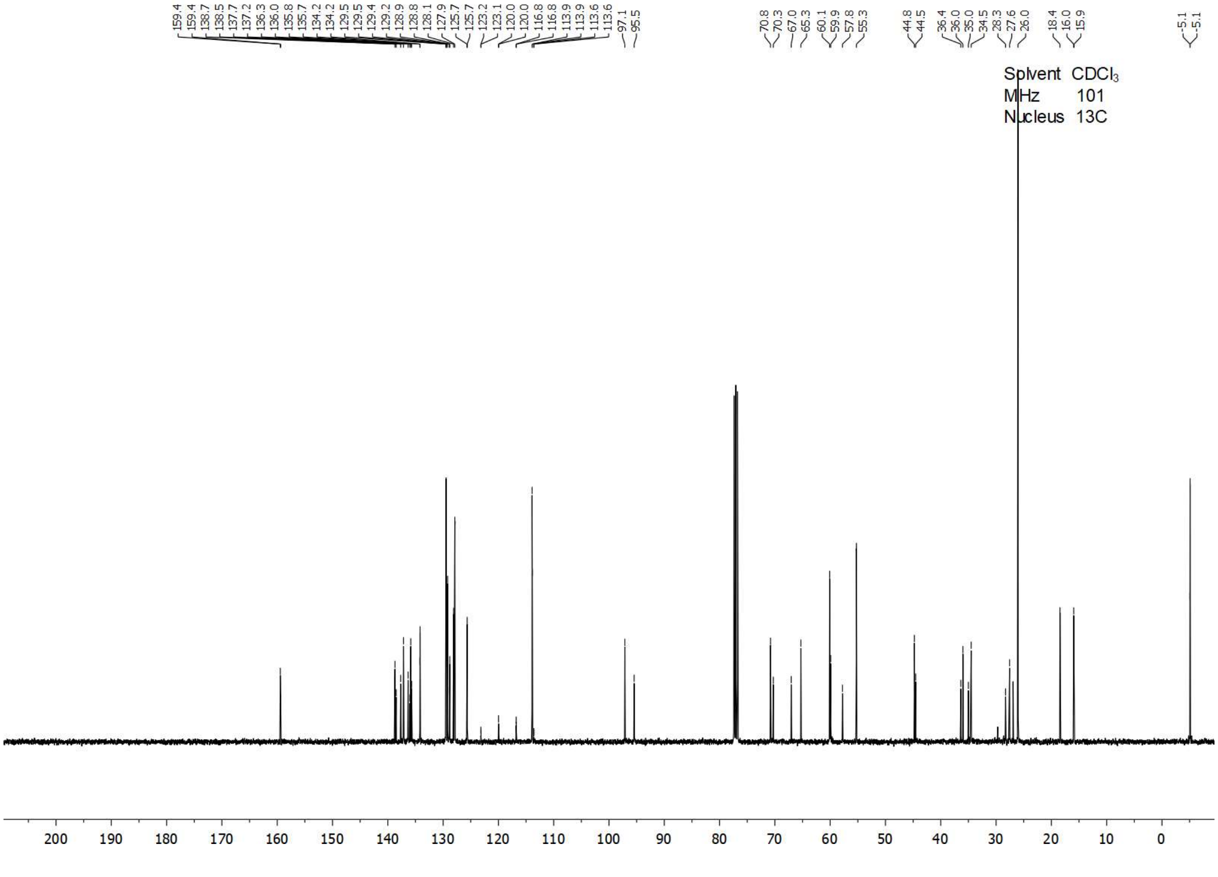
159.4
159.4
138.7
138.5
137.7
137.2
136.3
136.0
135.8
135.7
134.2
134.2
129.5
129.5
129.4
129.2
128.9
128.8
128.1
127.9
125.7
125.7
123.2
123.1
120.0
120.0
116.8
116.8
113.9
113.9
113.6
97.1
95.5
70.8
70.3
67.0
65.3
60.1
59.9
57.8
55.3
44.8
44.5
36.4
36.0
35.0
34.5
28.3
27.6
26.0
18.4
16.0
15.9
-5.1
-5.1
Solvent CDCl3
MHz 101
Nucleus 13C
200
190
180
170
160
150
140
130
120
110
100
90
80
70
60
50
40
30
20
10
0

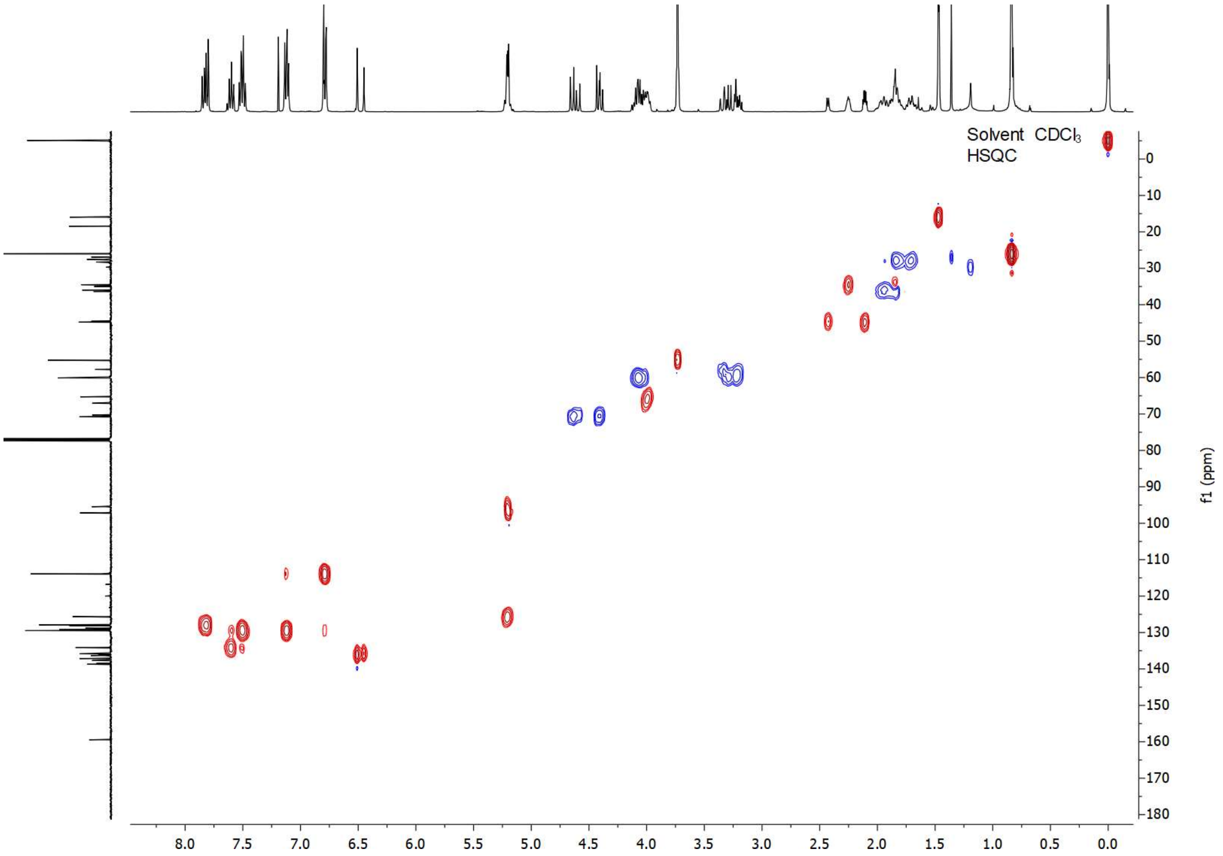
Solvent CDCl3
HSQC
f1 (ppm)

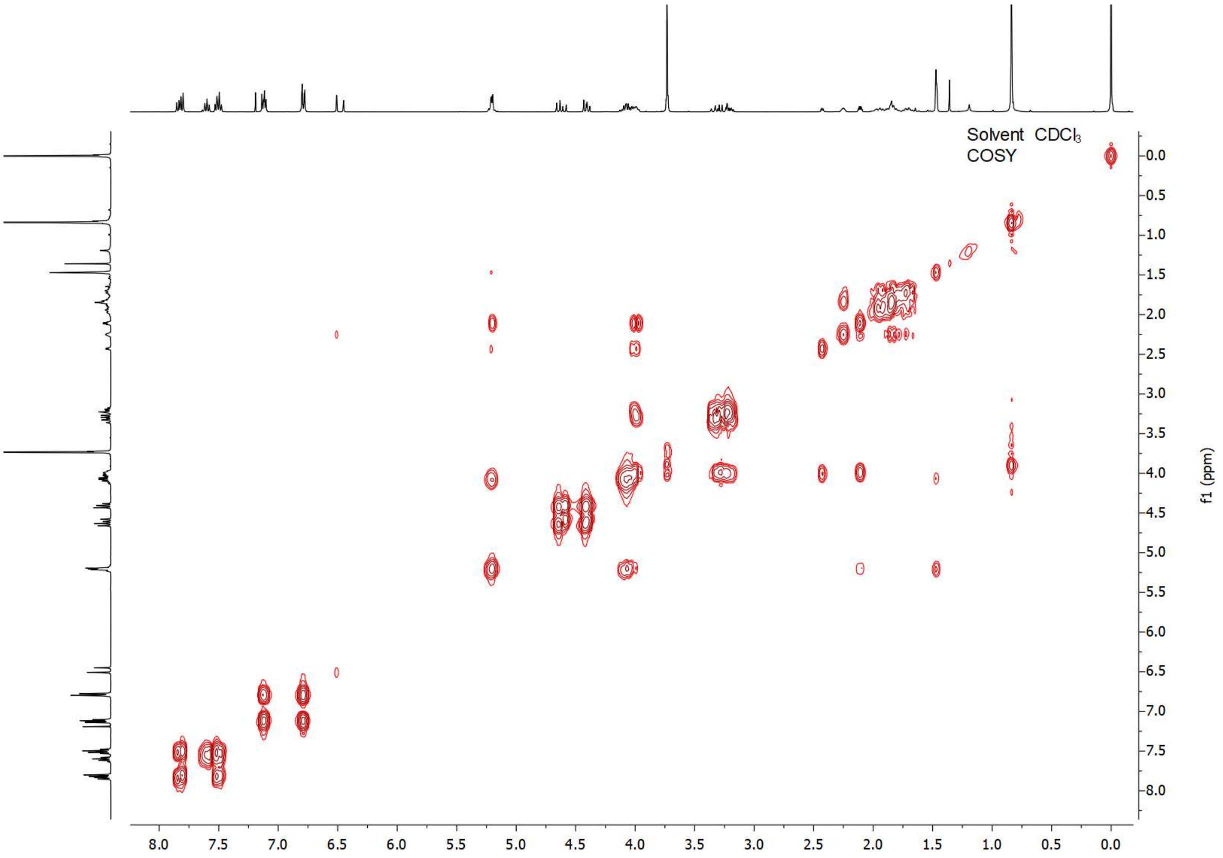
Solvent CDCl3
COSY
f1 (ppm)

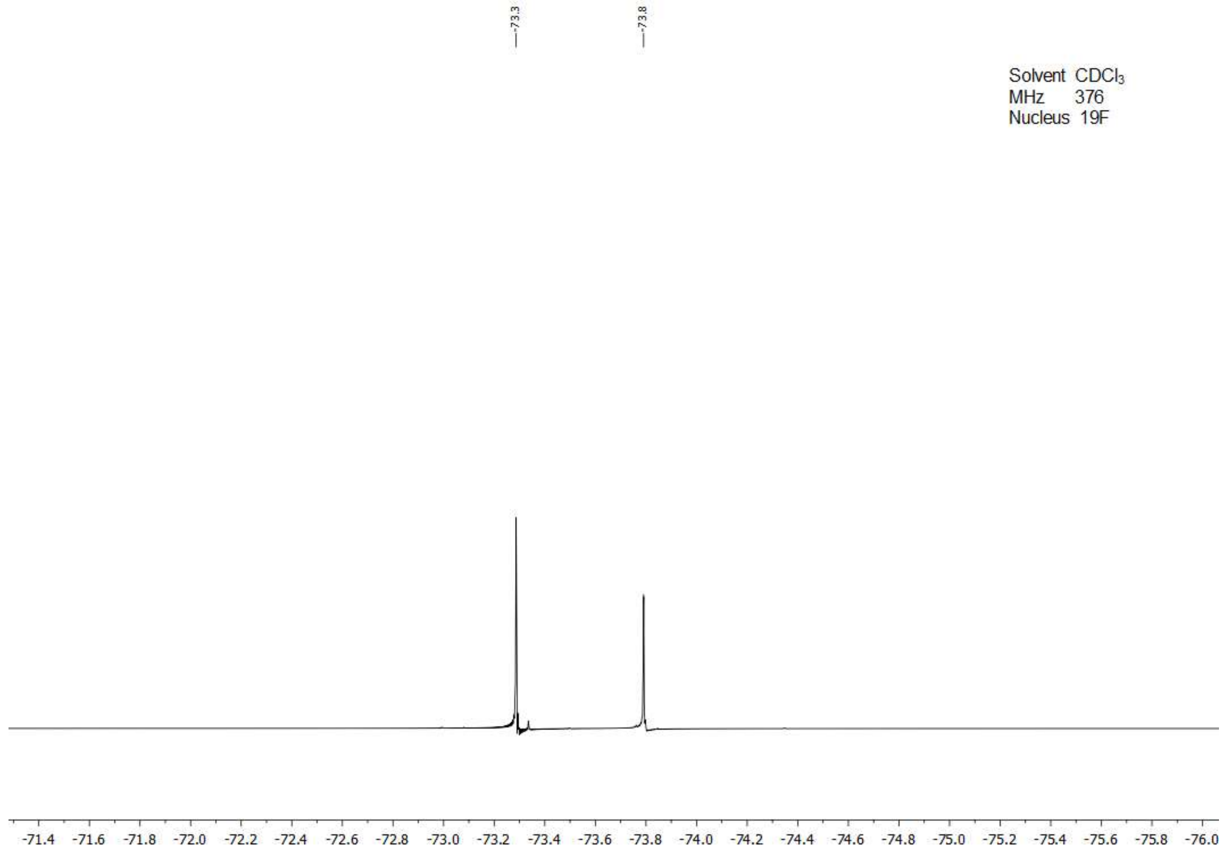

-73.3
-73.8
Solvent CDCl3
MHz 376
Nucleus 19F
-71.4 -71.6 -71.8 -72.0 -72.2 -72.4 -72.6 -72.8 -73.0 -73.2 -73.4 -73.6 -73.8 -74.0 -74.2 -74.4 -74.6 -74.8 -75.0 -75.2 -75.4 -75.6 -75.8 -76.0

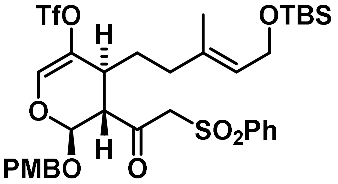
TfO
H
OTBS
O
SO2Ph
H
PMBO
O

S10

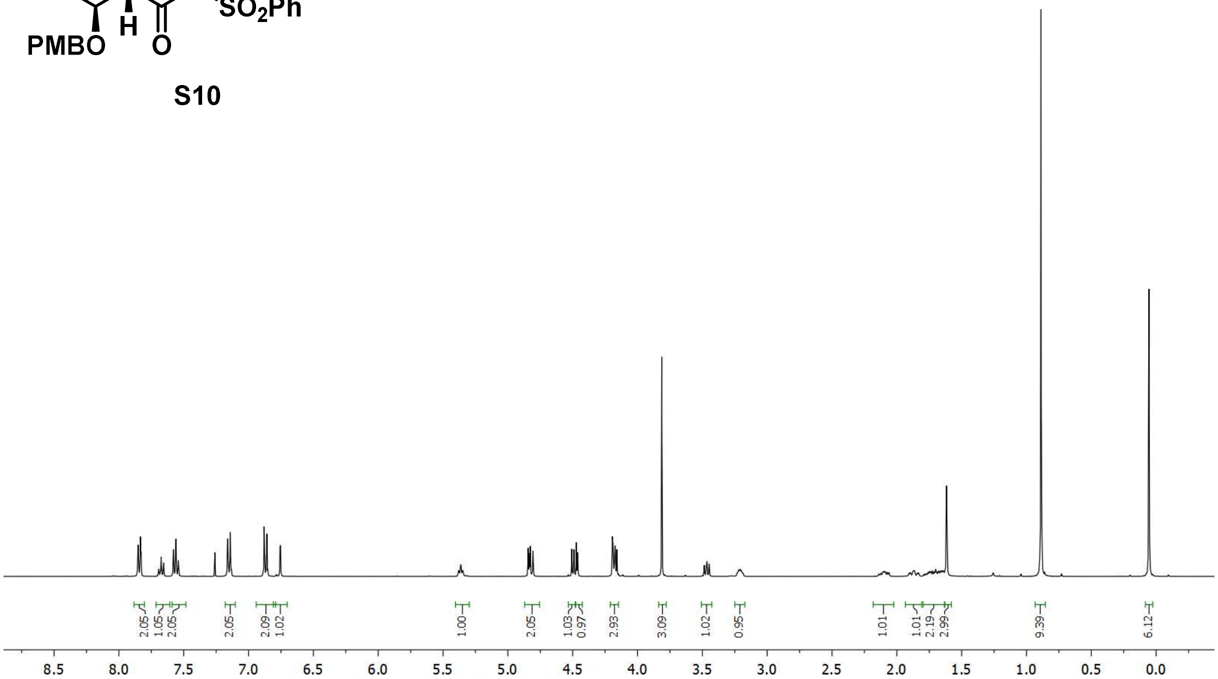
Solvent CDCl3
MHz 400
Nucleus 1H
2.05
1.05
2.05
2.05
2.09
1.02
1.00
2.05
1.03
0.97
2.93
3.09
1.02
0.95
1.01
1.01
2.19
2.99
9.39
6.12
8.5 8.0 7.5 7.0 6.5 6.0 5.5 5.0 4.5 4.0 3.5 3.0 2.5 2.0 1.5 1.0 0.5 0.0

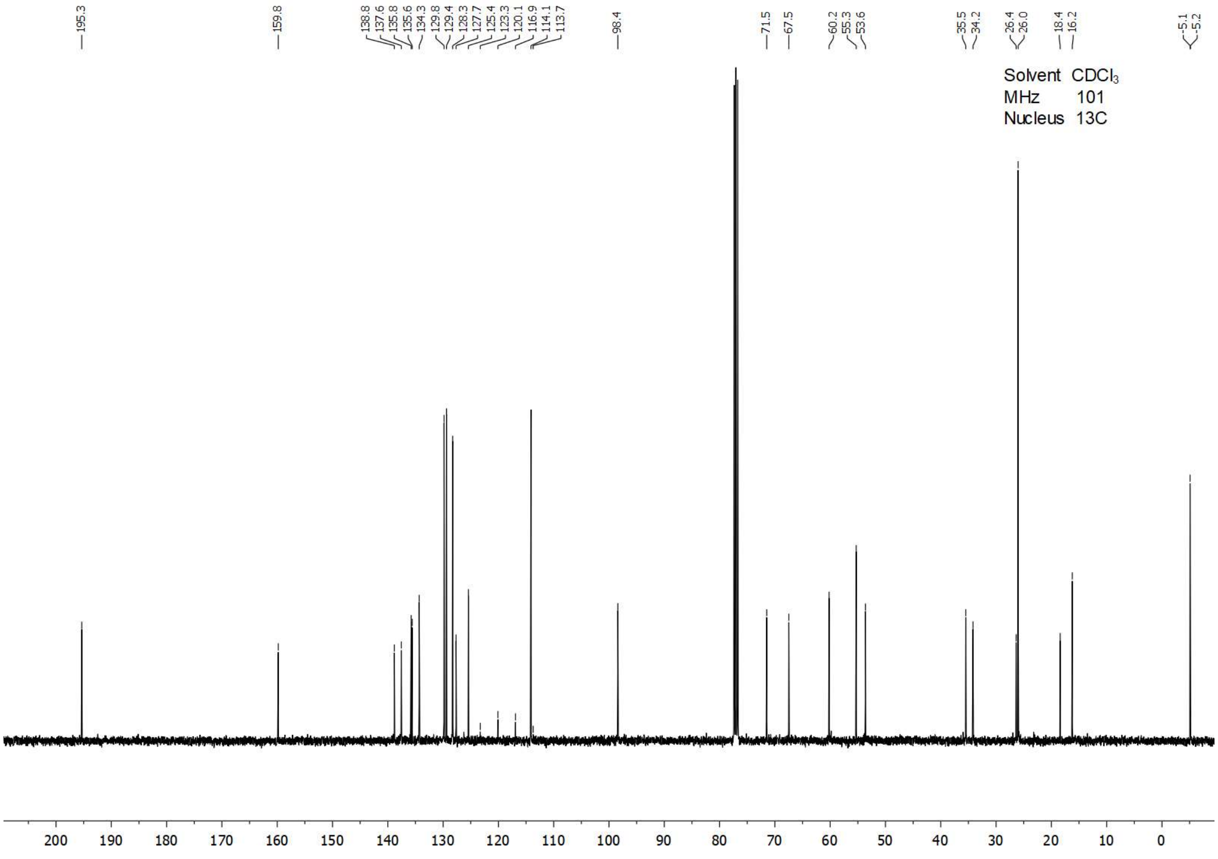
195.3
159.8
138.8
137.6
135.8
135.6
134.3
129.8
129.4
128.3
127.7
125.4
123.3
120.1
116.9
114.1
113.7
98.4
71.5
67.5
60.2
55.3
53.6
35.5
34.2
26.4
26.0
18.4
16.2
-5.1
-5.2
Solvent CDCl3
MHz 101
Nucleus 13C
200 190 180 170 160 150 140 130 120 110 100 90 80 70 60 50 40 30 20 10 0

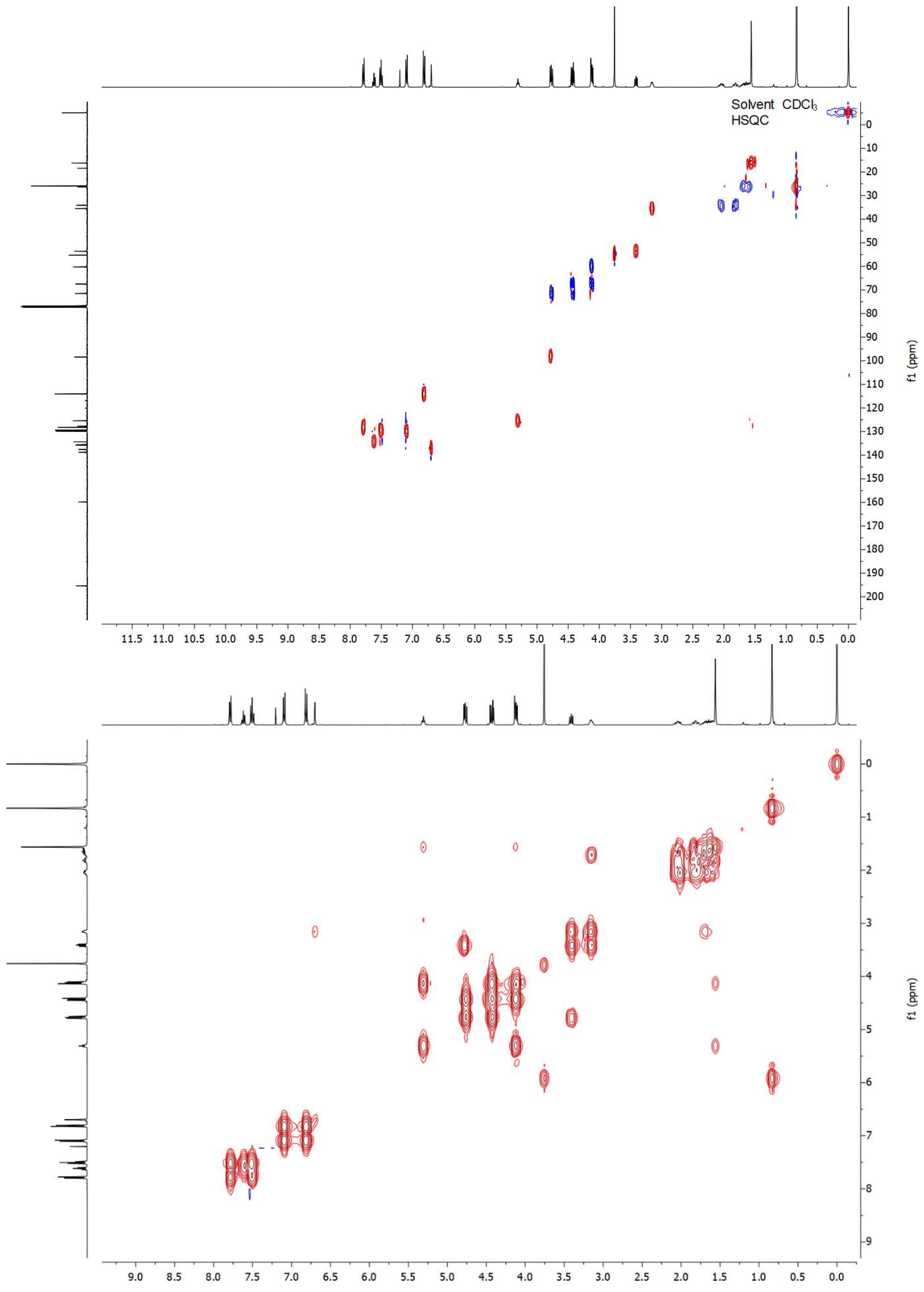
Solvent CDCl3
HSQC
f1 (ppm)
f1 (ppm)

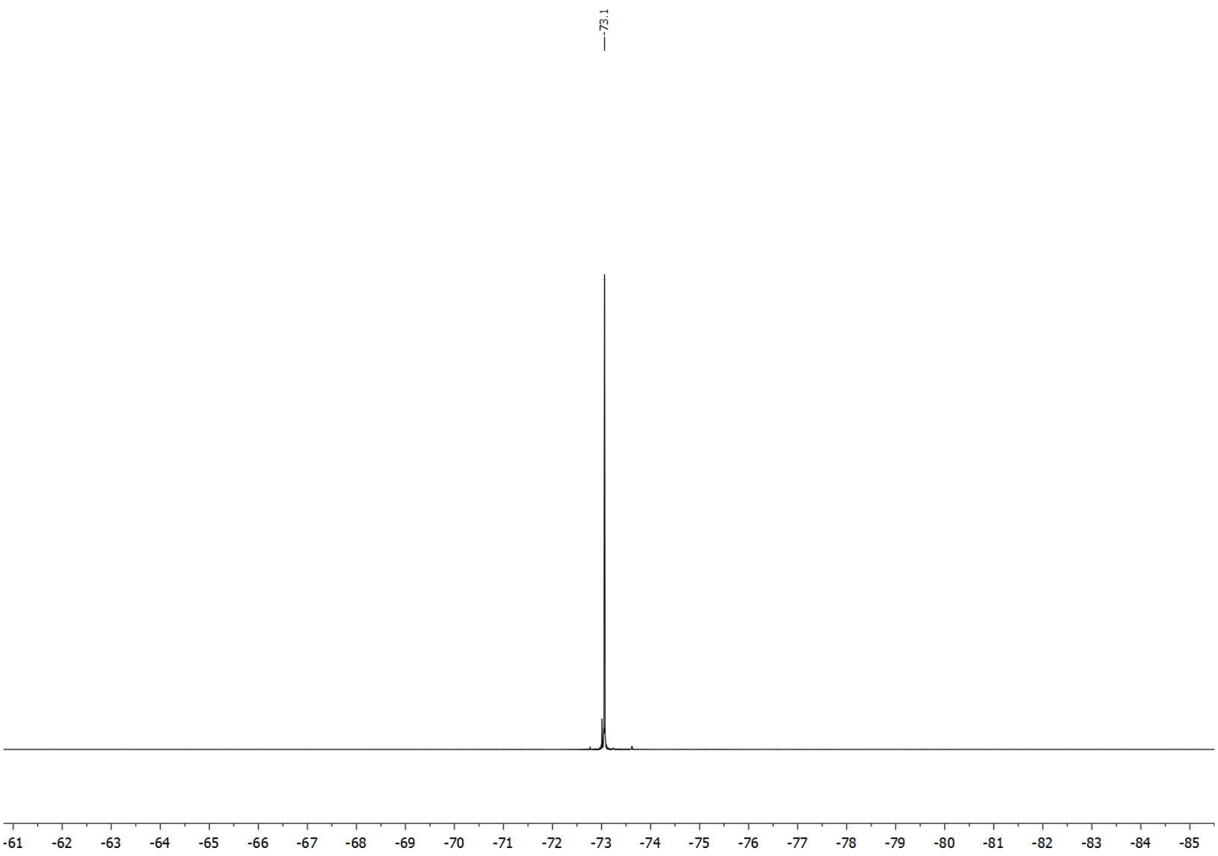
-73.1
-61 -62 -63 -64 -65 -66 -67 -68 -69 -70 -71 -72 -73 -74 -75 -76 -77 -78 -79 -80 -81 -82 -83 -84 -85

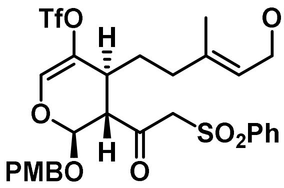
TfO
OH
H
O
SO2Ph
H
PMBO
O

S11

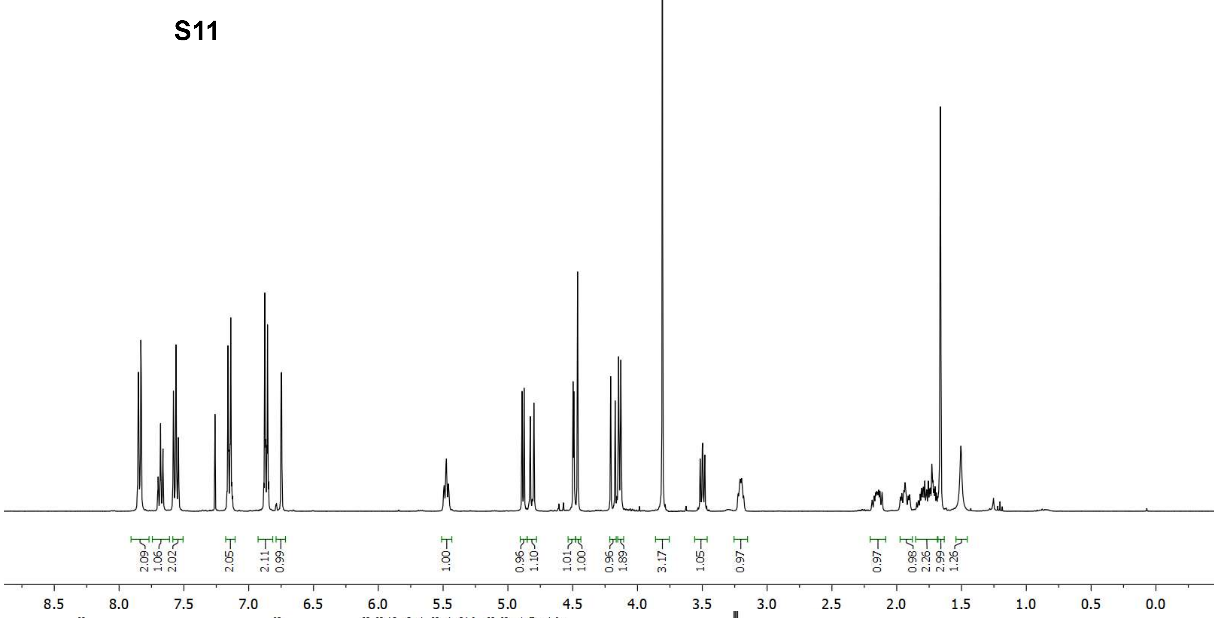
Solvent CDCl3
MHz 400
Nucleus 1H
2.09
1.06
2.02
2.05
2.11
0.99
1.00
0.96
1.10
1.01
1.00
0.96
1.89
3.17
1.05
0.97
0.97
0.98
2.26
2.99
1.26
8.5
8.0
7.5
7.0
6.5
6.0
5.5
5.0
4.5
4.0
3.5
3.0
2.5
2.0
1.5
1.0
0.5
0.0

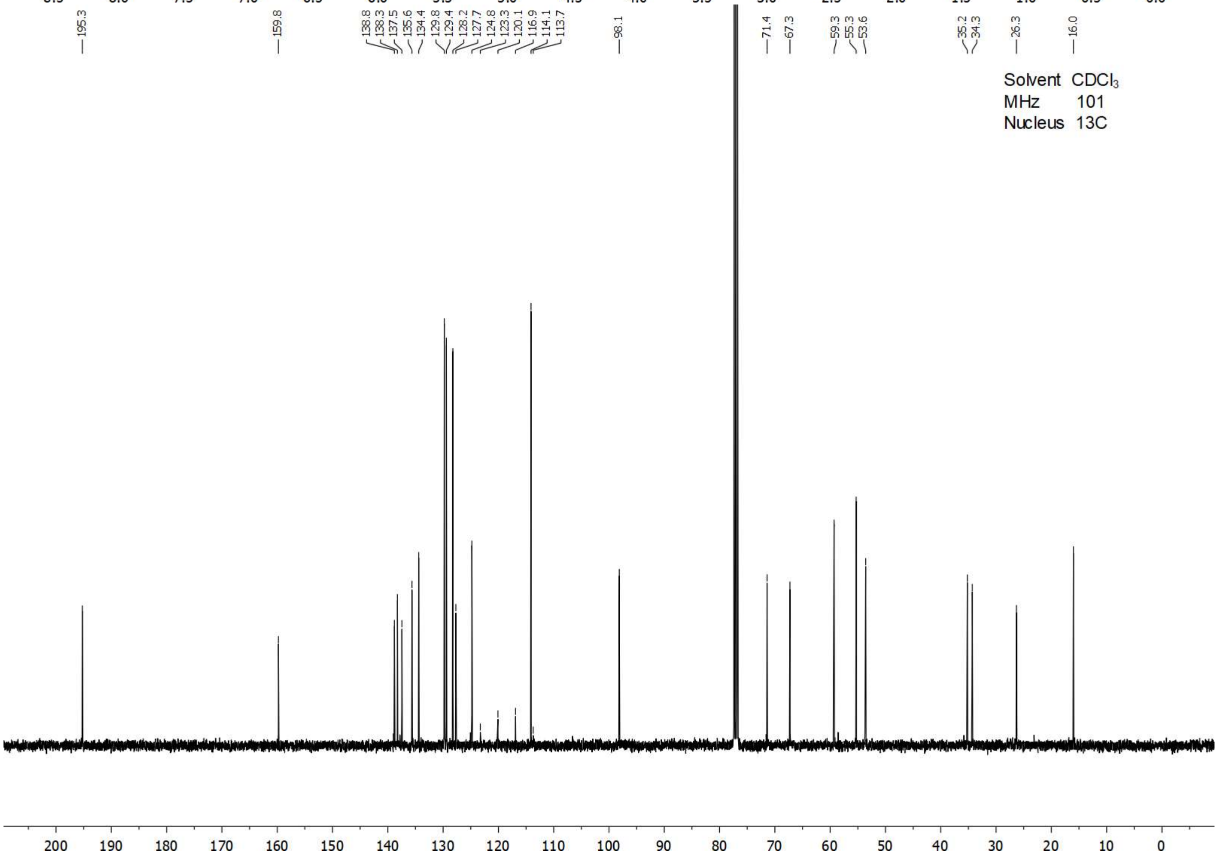
195.3
159.8
138.8
138.3
137.5
135.6
134.4
129.8
129.4
128.2
127.7
124.8
123.3
120.1
116.9
114.1
113.7
98.1
71.4
67.3
59.3
55.3
53.6
35.2
34.3
26.3
16.0
Solvent CDCl3
MHz 101
Nucleus 13C
200
190
180
170
160
150
140
130
120
110
100
90
80
70
60
50
40
30
20
10
0

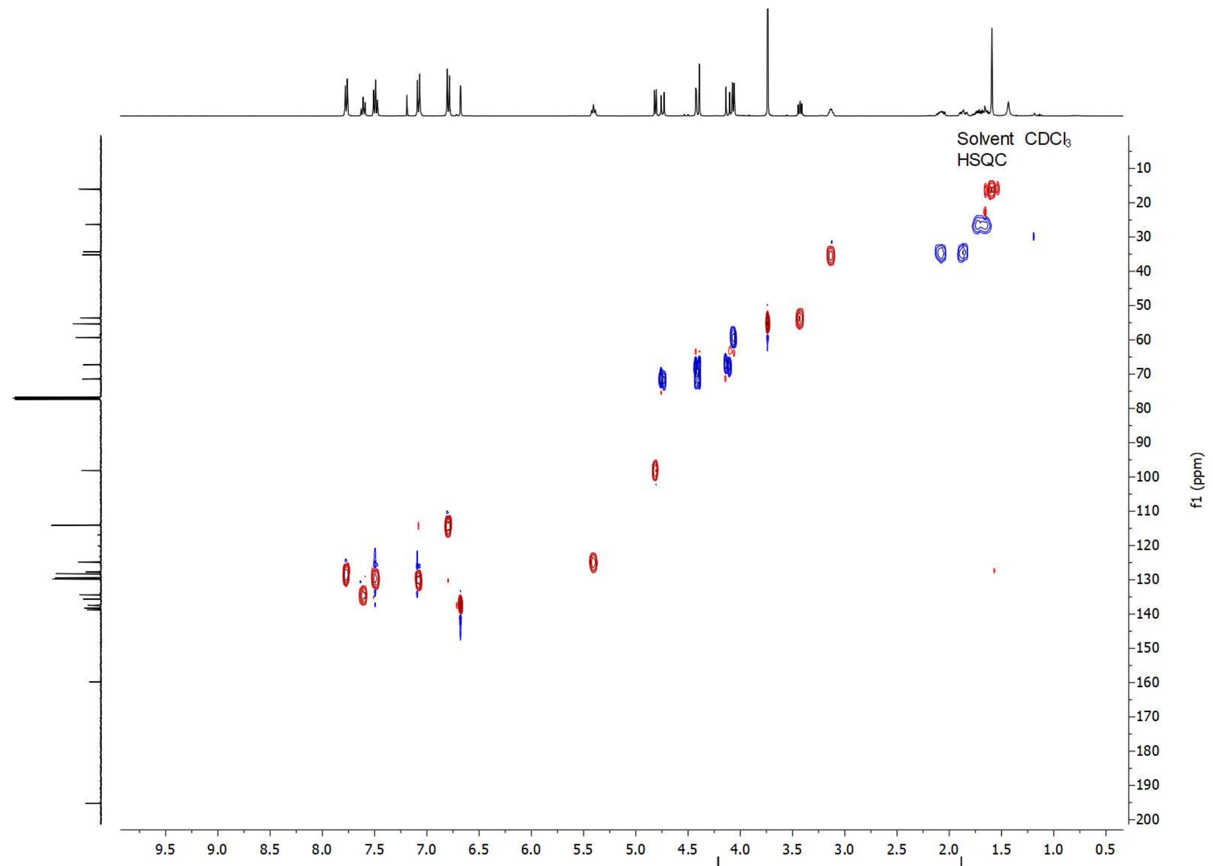

Solvent CDCl3
HSQC
f1 (ppm)

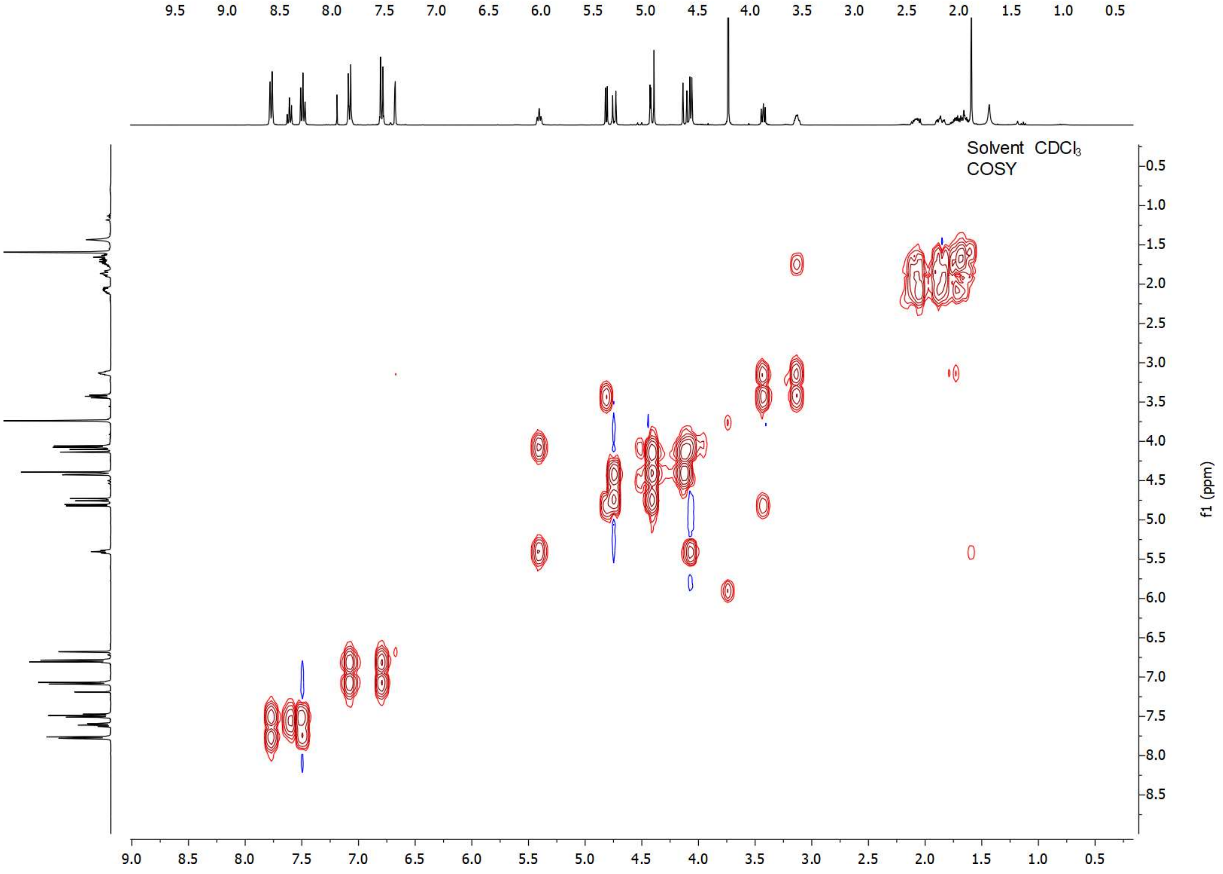

Solvent CDCl3
COSY
f1 (ppm)

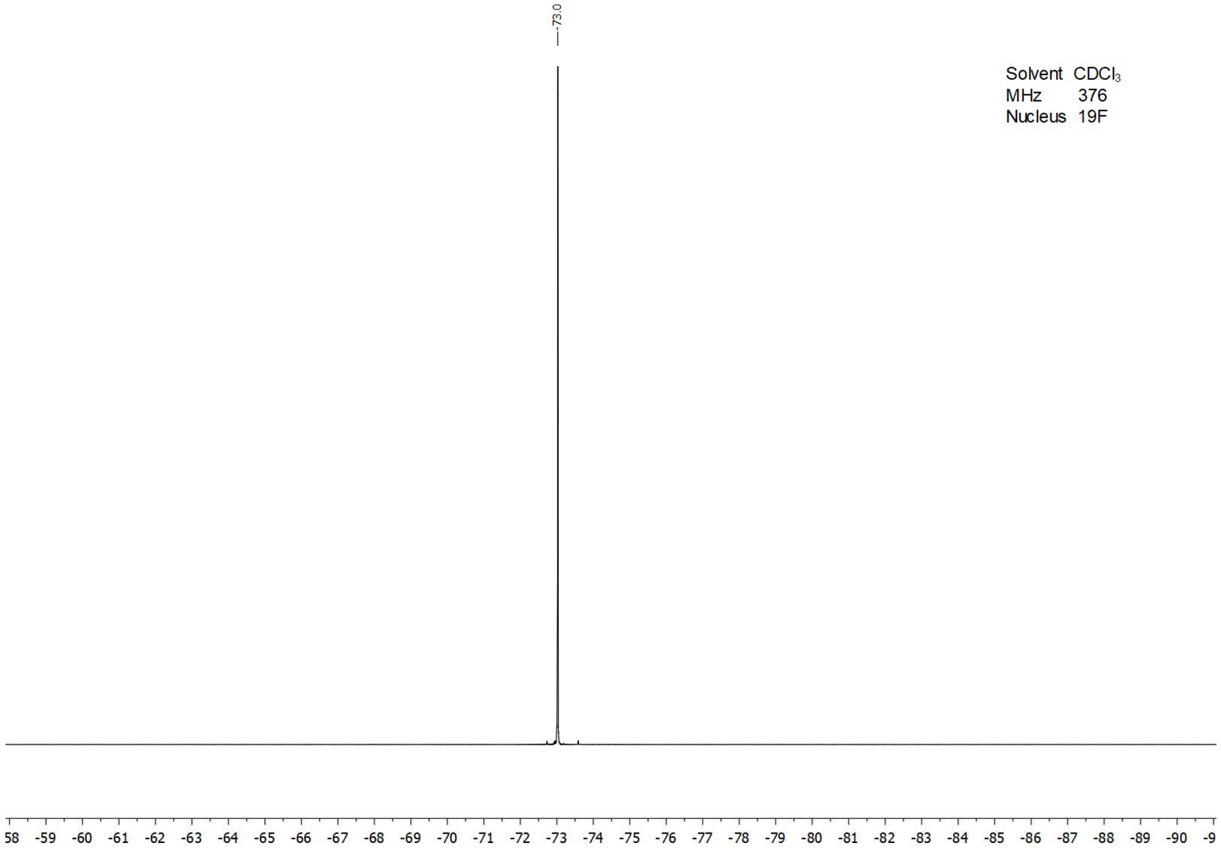

-73.0
Solvent CDCl3
MHz 376
Nucleus 19F
58 -59 -60 -61 -62 -63 -64 -65 -66 -67 -68 -69 -70 -71 -72 -73 -74 -75 -76 -77 -78 -79 -80 -81 -82 -83 -84 -85 -86 -87 -88 -89 -90 -9

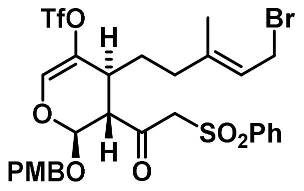
TfO
H
Br
O
SO2Ph
H
PMBO
O

8

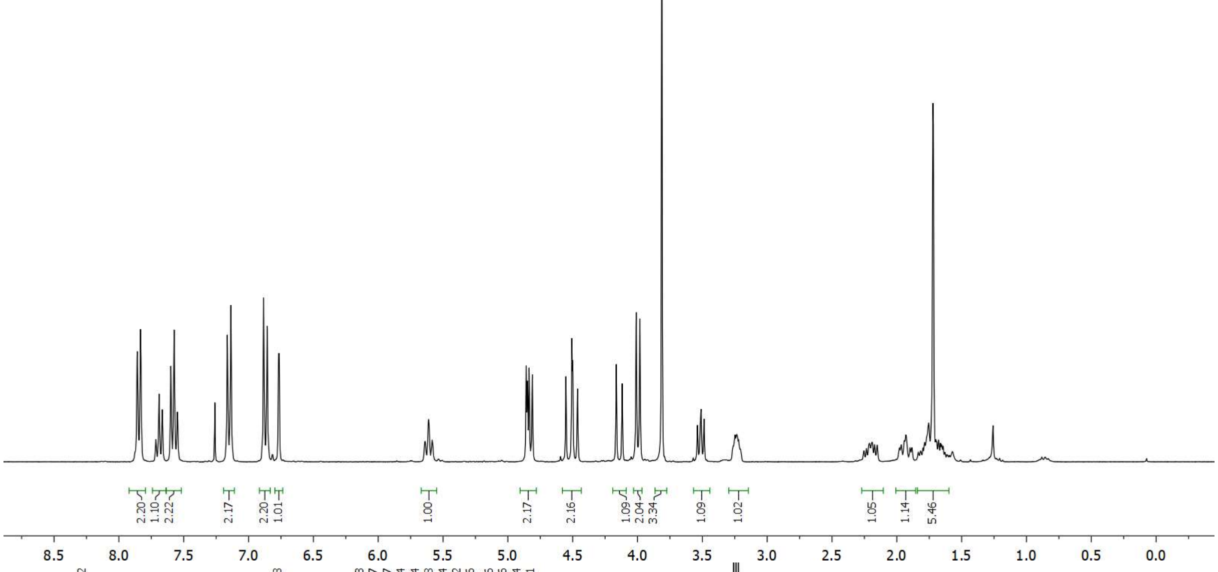
Solvent CDCl3
MHz 400
Nucleus 1H
2.20
1.10
2.22
2.17
2.20
1.01
1.00
2.17
2.16
1.09
2.04
3.34
1.09
1.02
1.05
1.14
5.46
8.5
8.0
7.5
7.0
6.5
6.0
5.5
5.0
4.5
4.0
3.5
3.0
2.5
2.0
1.5
1.0
0.5
0.0

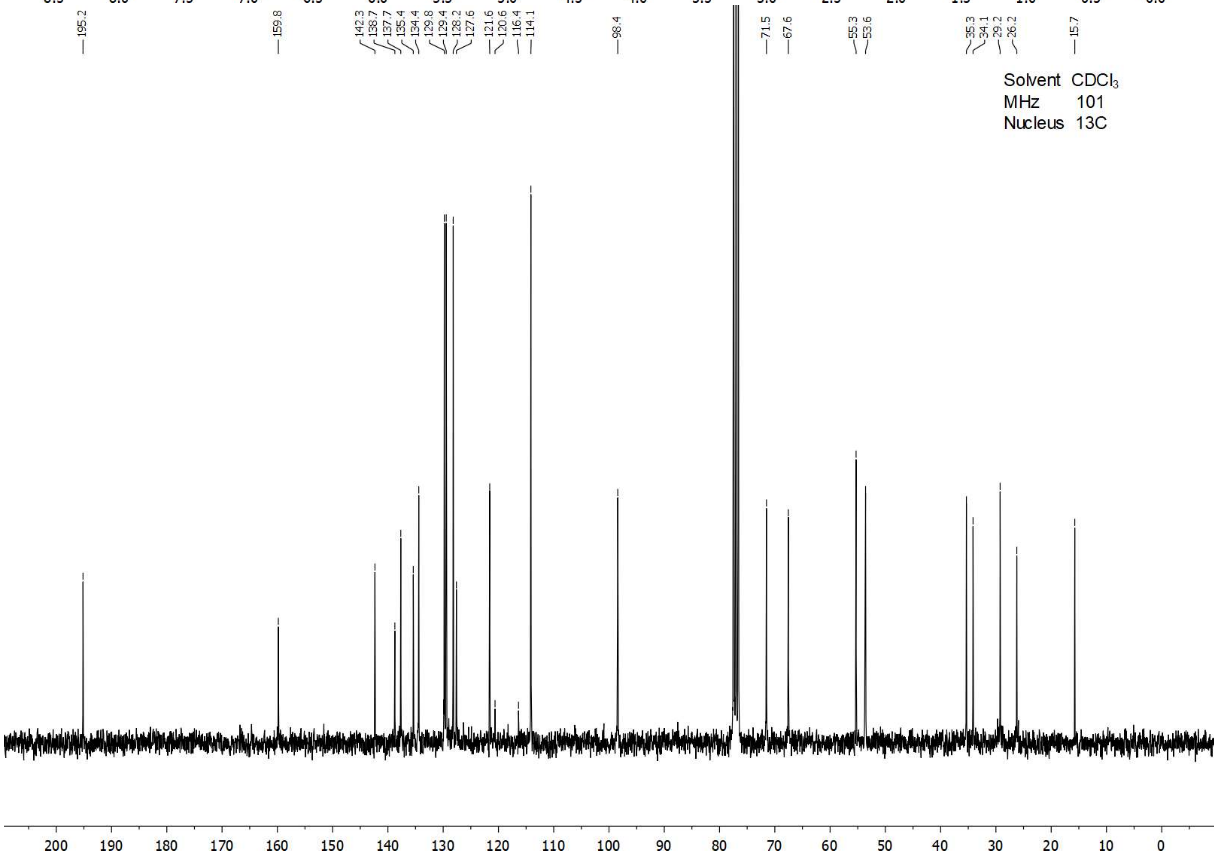
195.2
159.8
142.3
138.7
137.7
135.4
134.4
129.8
129.4
128.2
127.6
121.6
120.6
116.4
114.1
98.4
71.5
67.6
55.3
53.6
35.3
34.1
29.2
26.2
15.7
Solvent CDCl3
MHz 101
Nucleus 13C
200
190
180
170
160
150
140
130
120
110
100
90
80
70
60
50
40
30
20
10
0

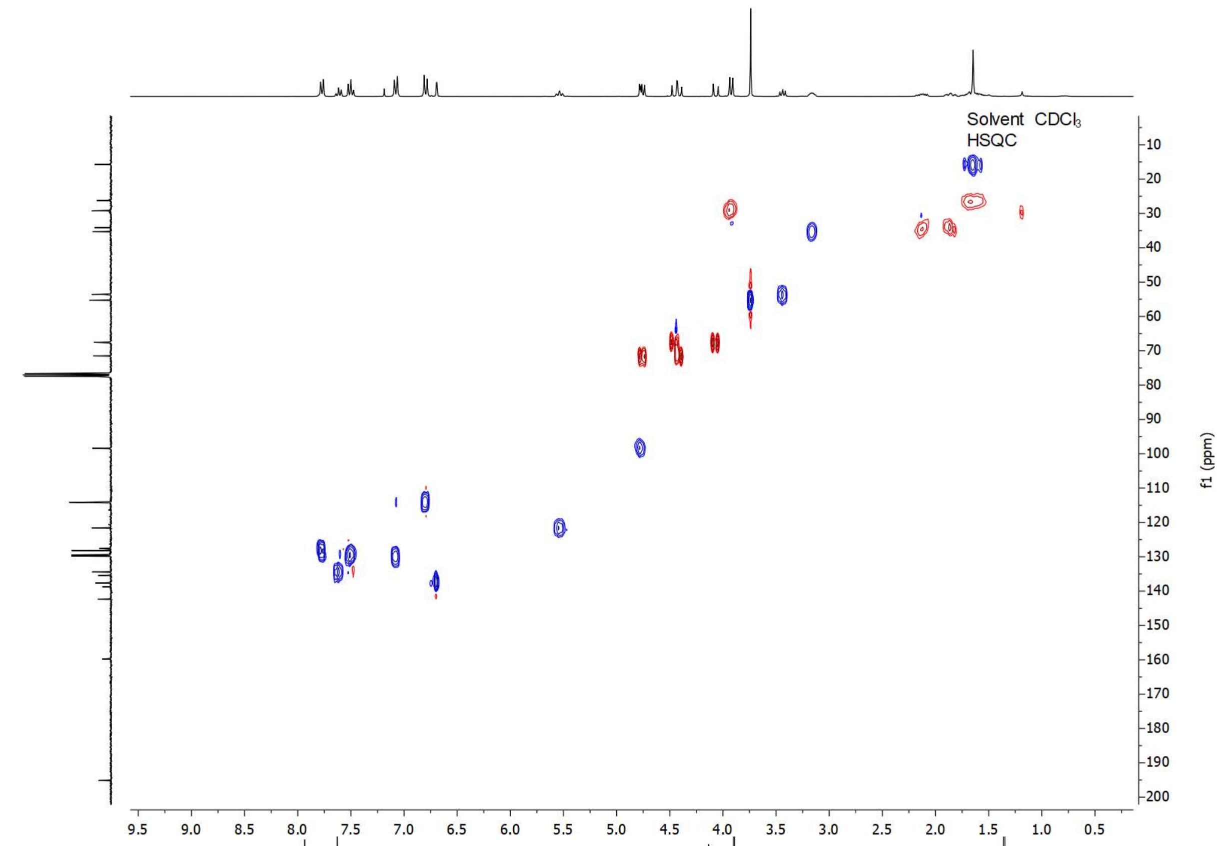
Solvent CDCl3
HSQC
f1 (ppm)

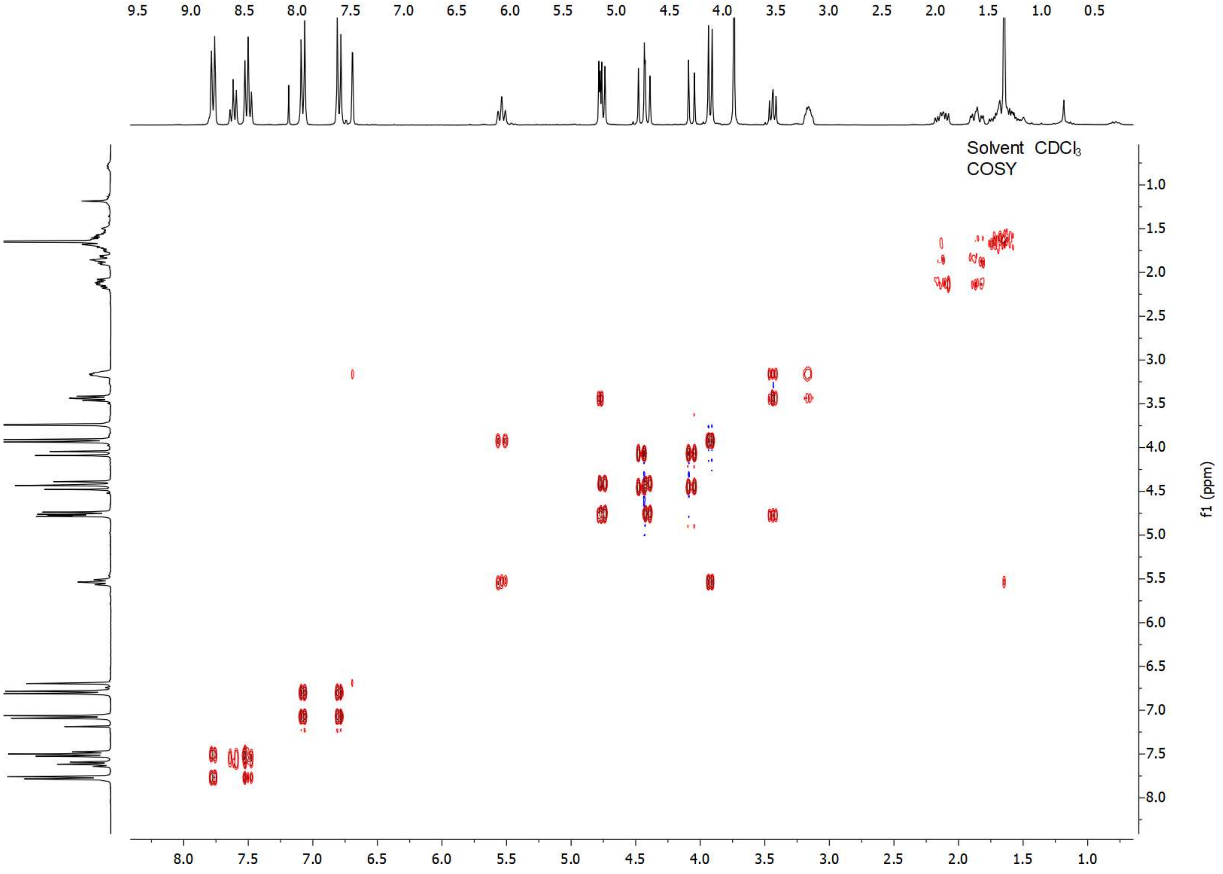
Solvent CDCl3
COSY
f1 (ppm)

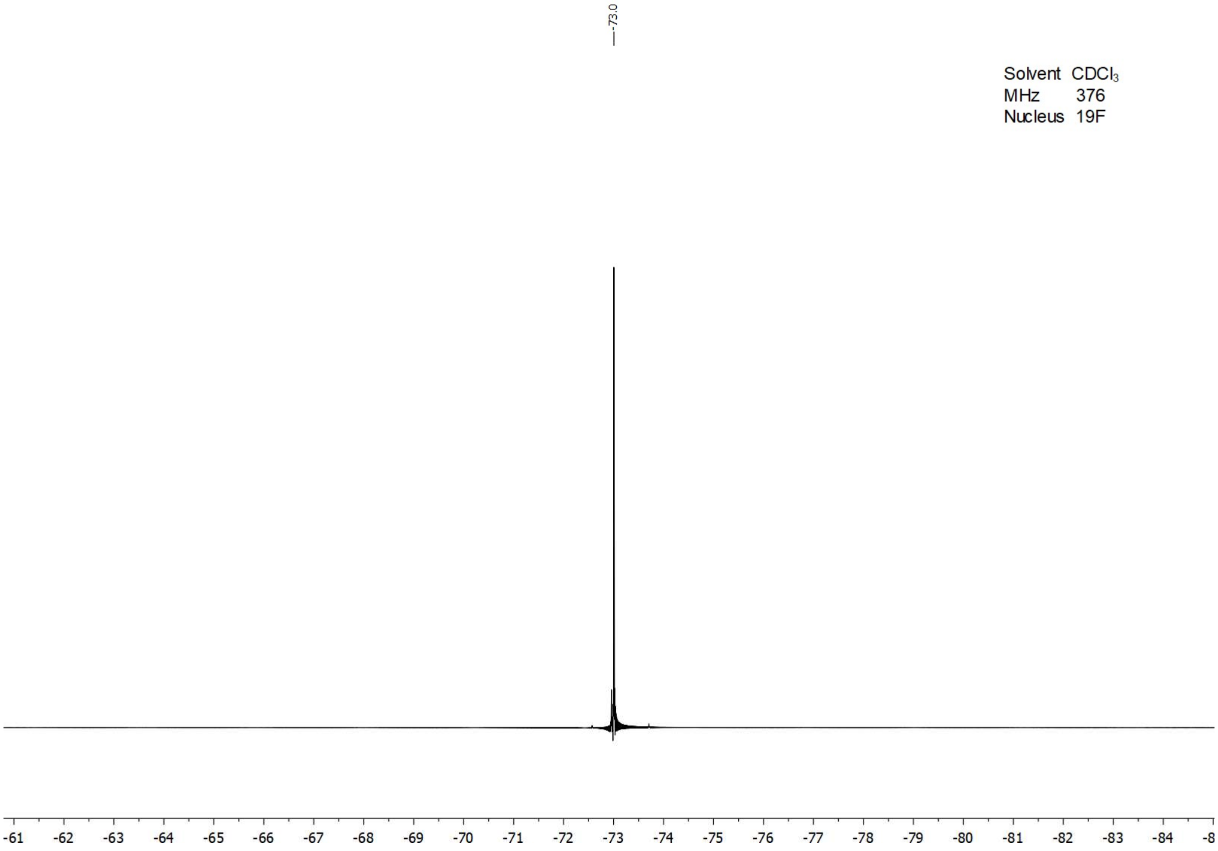
-73.0
Solvent CDCl3
MHz 376
Nucleus 19F
-61 -62 -63 -64 -65 -66 -67 -68 -69 -70 -71 -72 -73 -74 -75 -76 -77 -78 -79 -80 -81 -82 -83 -84 -8

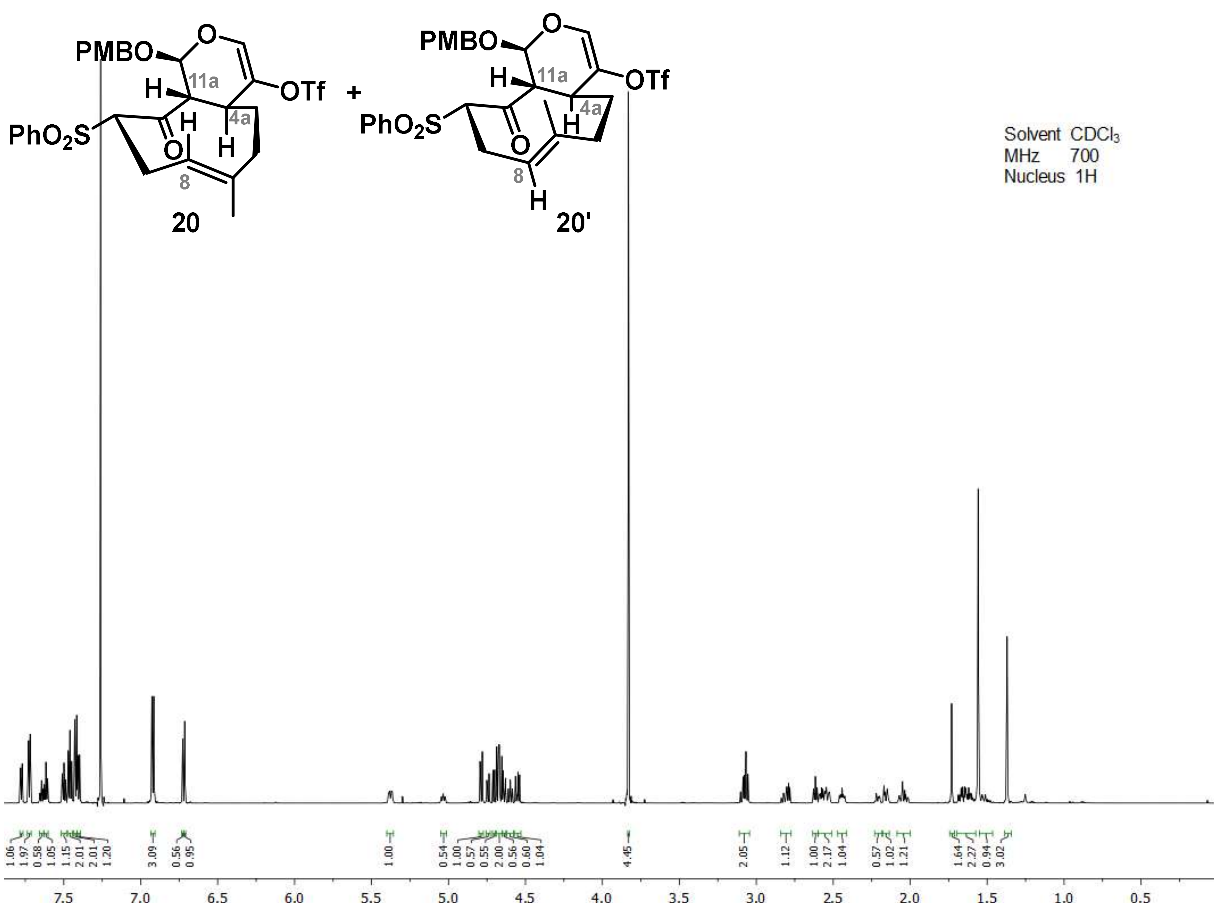
PMBO
OTf
PhO2S
11a
4a
8
20
20'
Solvent CDCl3
MHz 700
Nucleus 1H

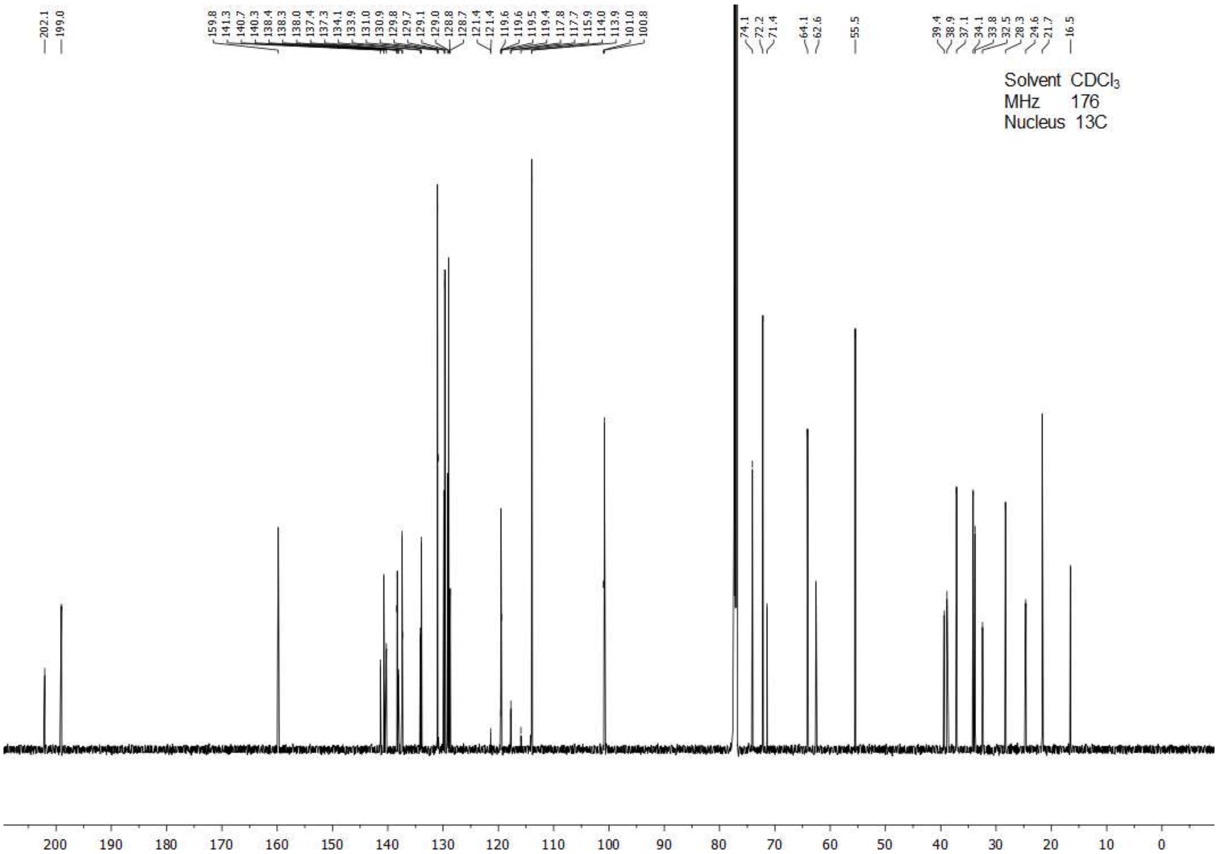
Solvent CDCl3
MHz 176
Nucleus 13C

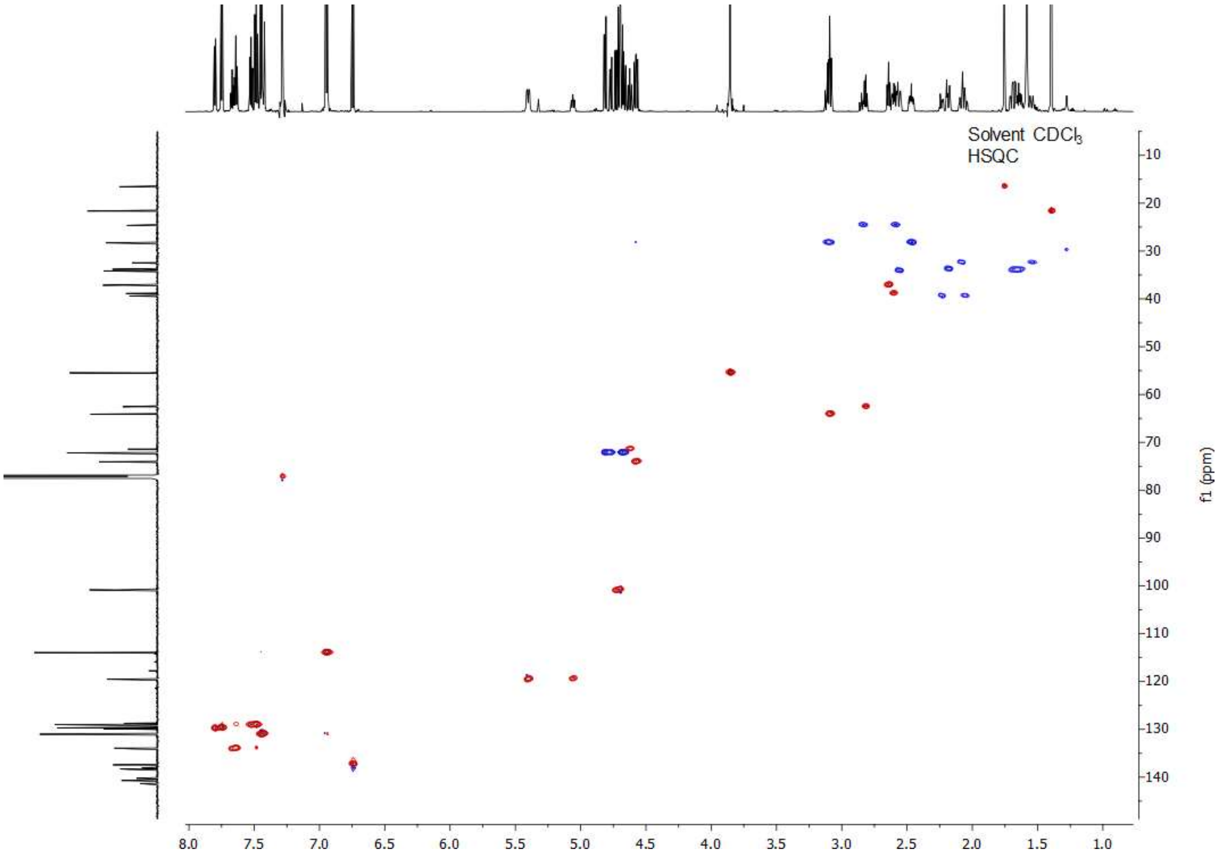

Solvent CDCl3
HSQC
f1 (ppm)

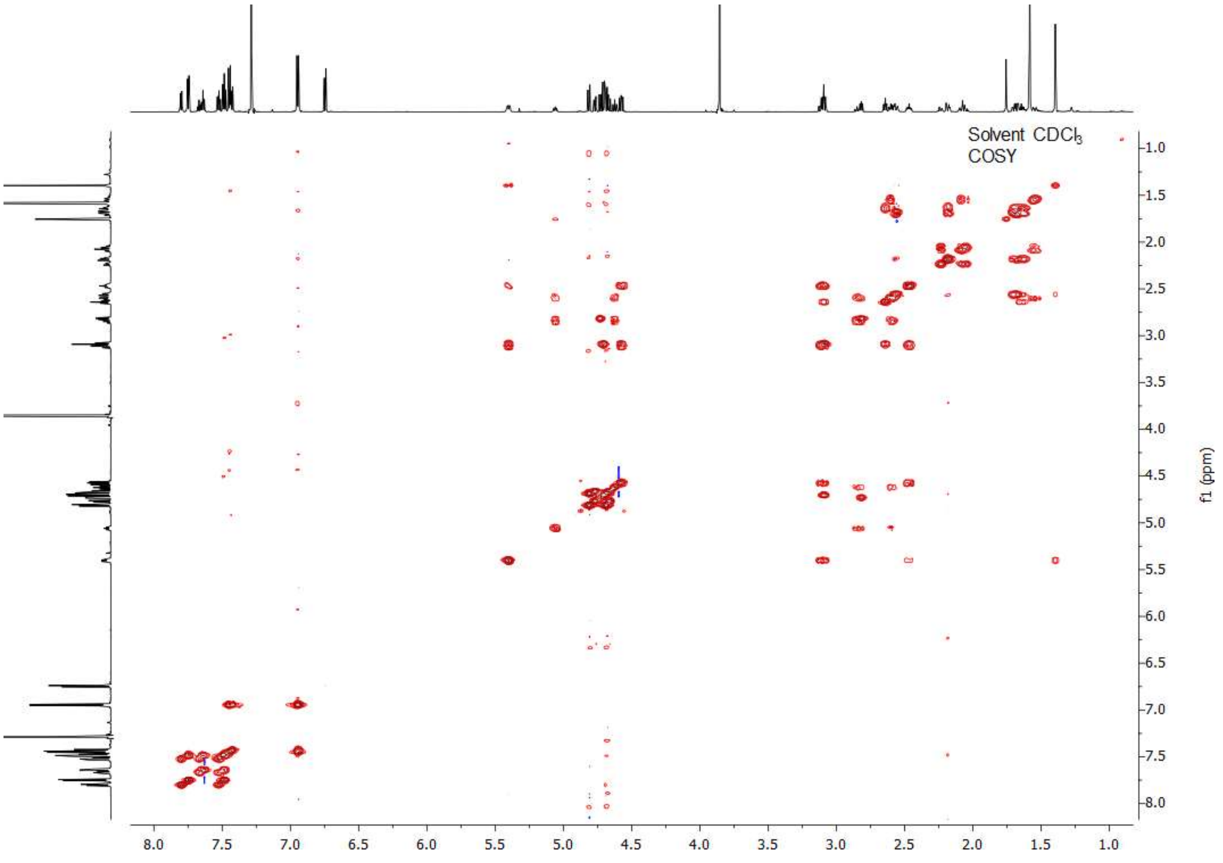

Solvent CDCl3
COSY
f1 (ppm)

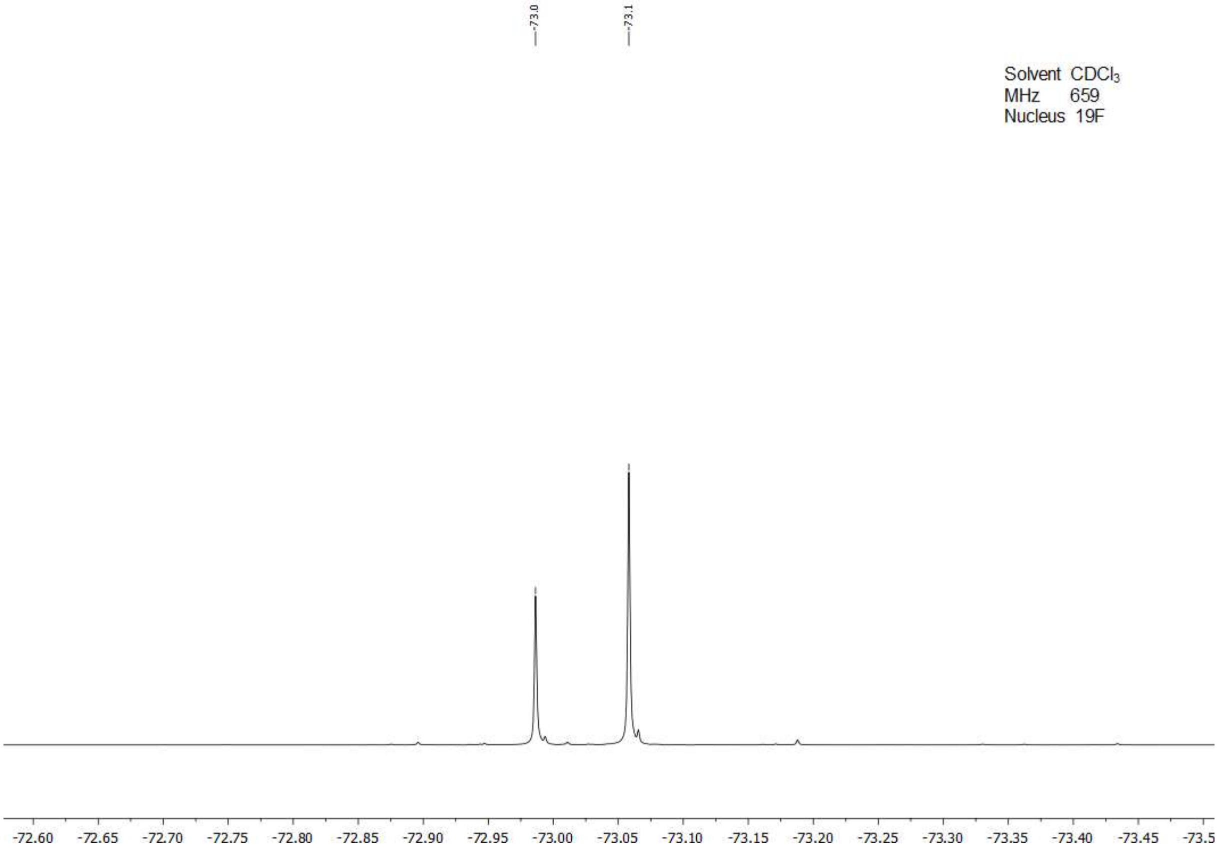
-73.0
-73.1
Solvent CDCl3
MHz 659
Nucleus 19F
-72.60 -72.65 -72.70 -72.75 -72.80 -72.85 -72.90 -72.95 -73.00 -73.05 -73.10 -73.15 -73.20 -73.25 -73.30 -73.35 -73.40 -73.45 -73.5

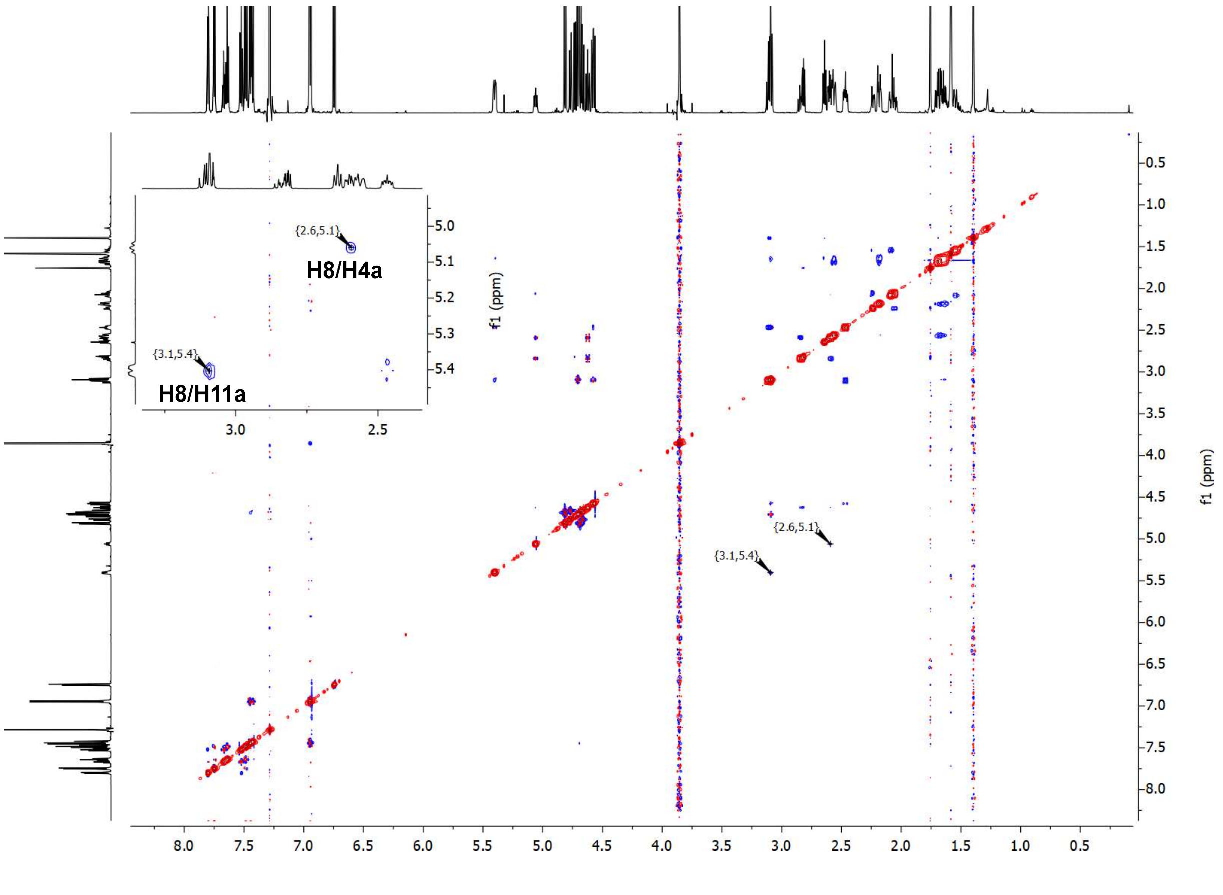
{2.6,5.1}
H8/H4a
{3.1,5.4}
H8/H11a
f1 (ppm)
f1 (ppm)

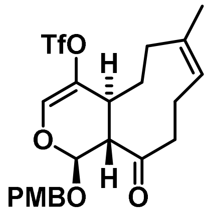

21

Solvent CDCl$_3$
MHz 400
Nucleus 1H

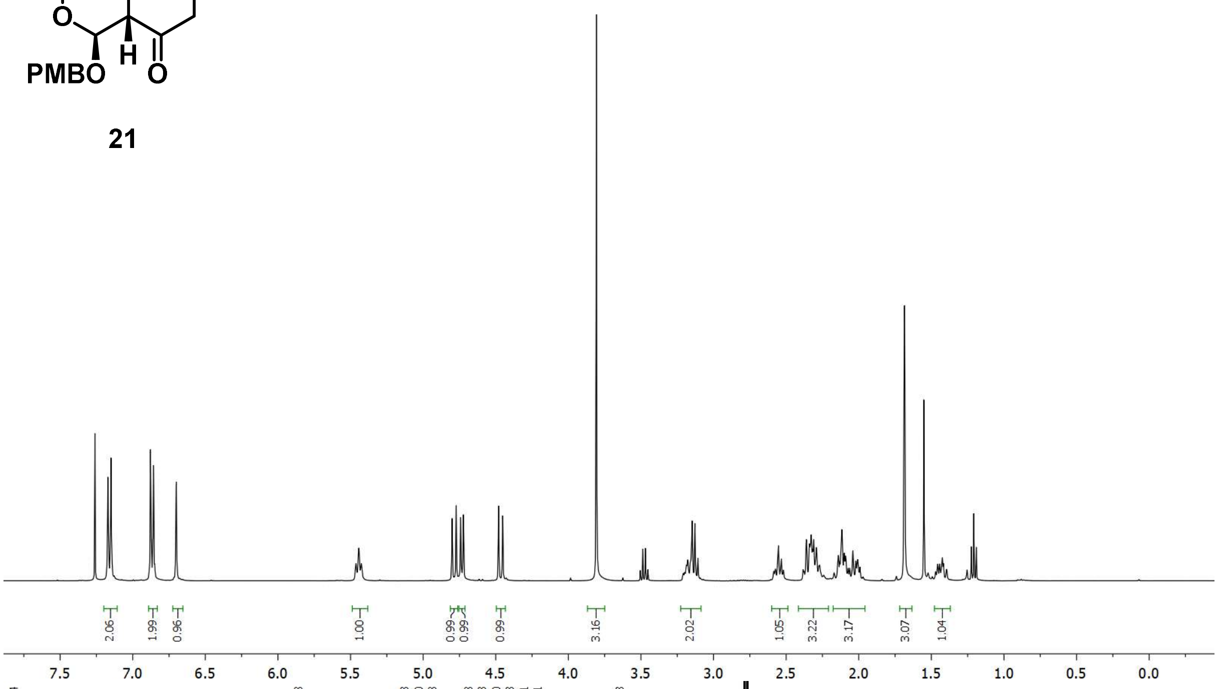

Solvent CDCl$_3$
MHz 101
Nucleus 13C

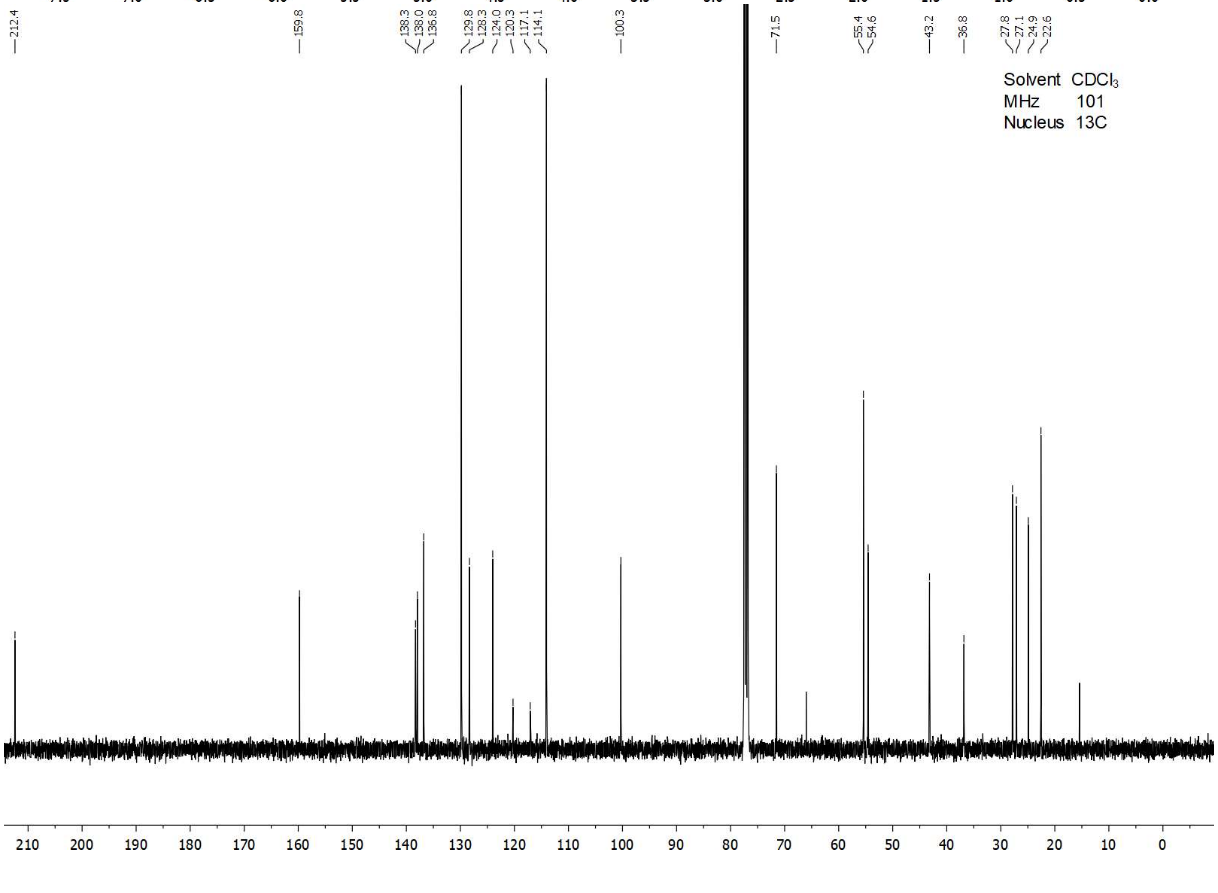

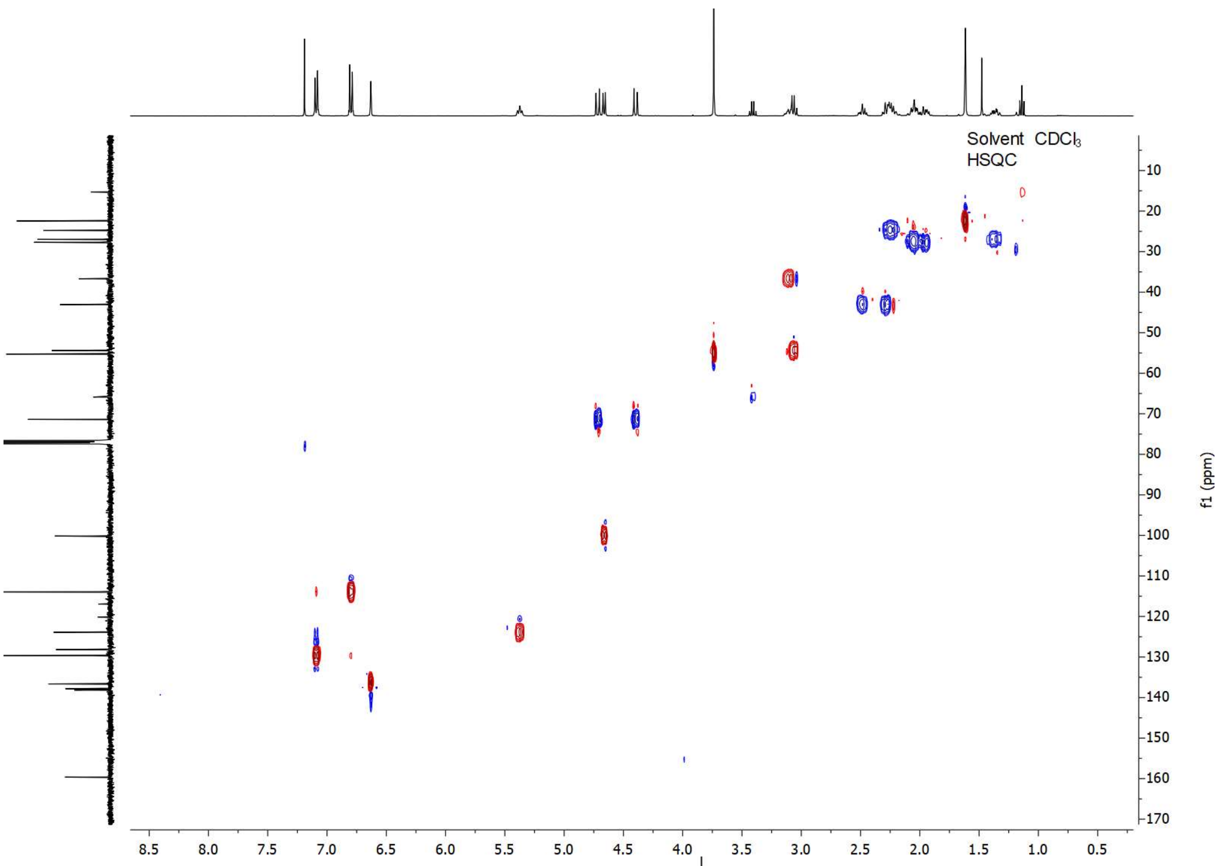
Solvent CDCl3
HSQC
f1 (ppm)

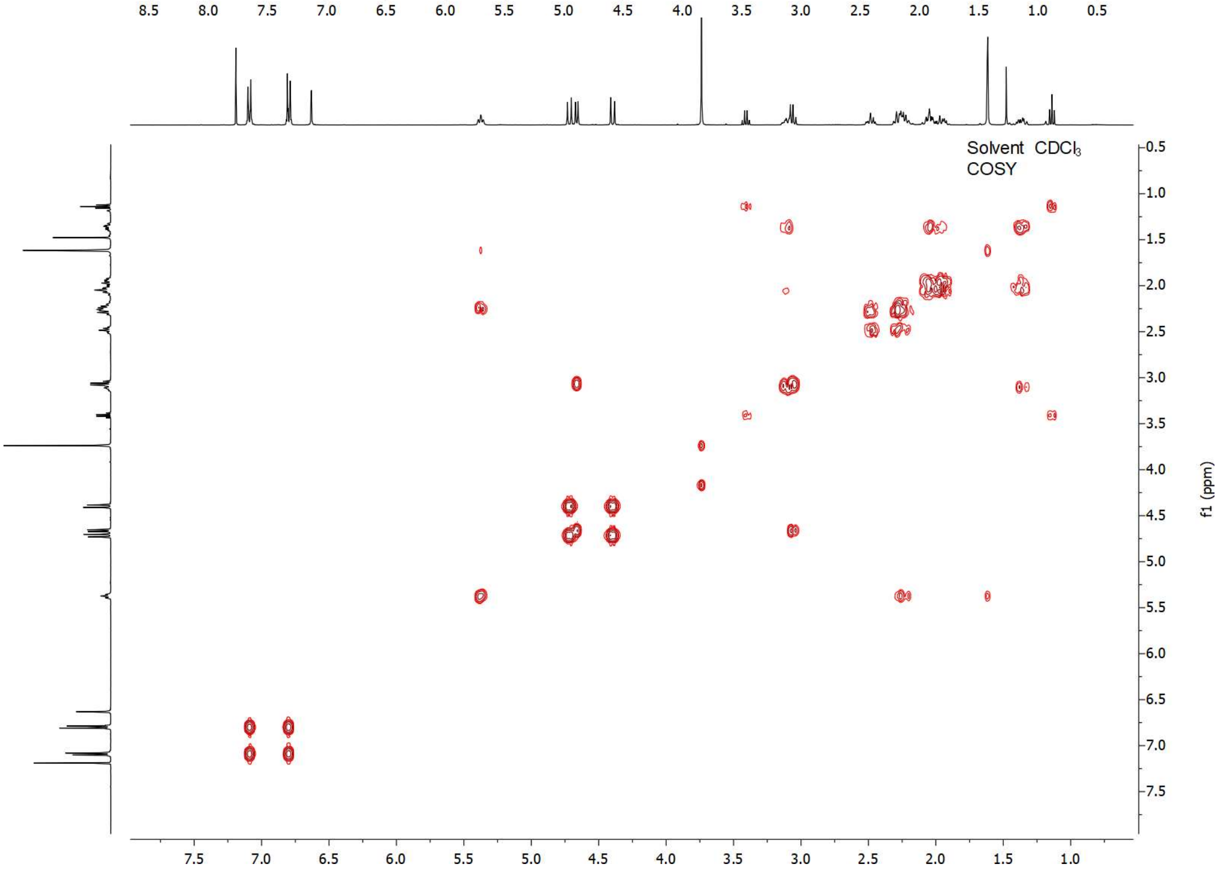
Solvent CDCl3
COSY
f1 (ppm)

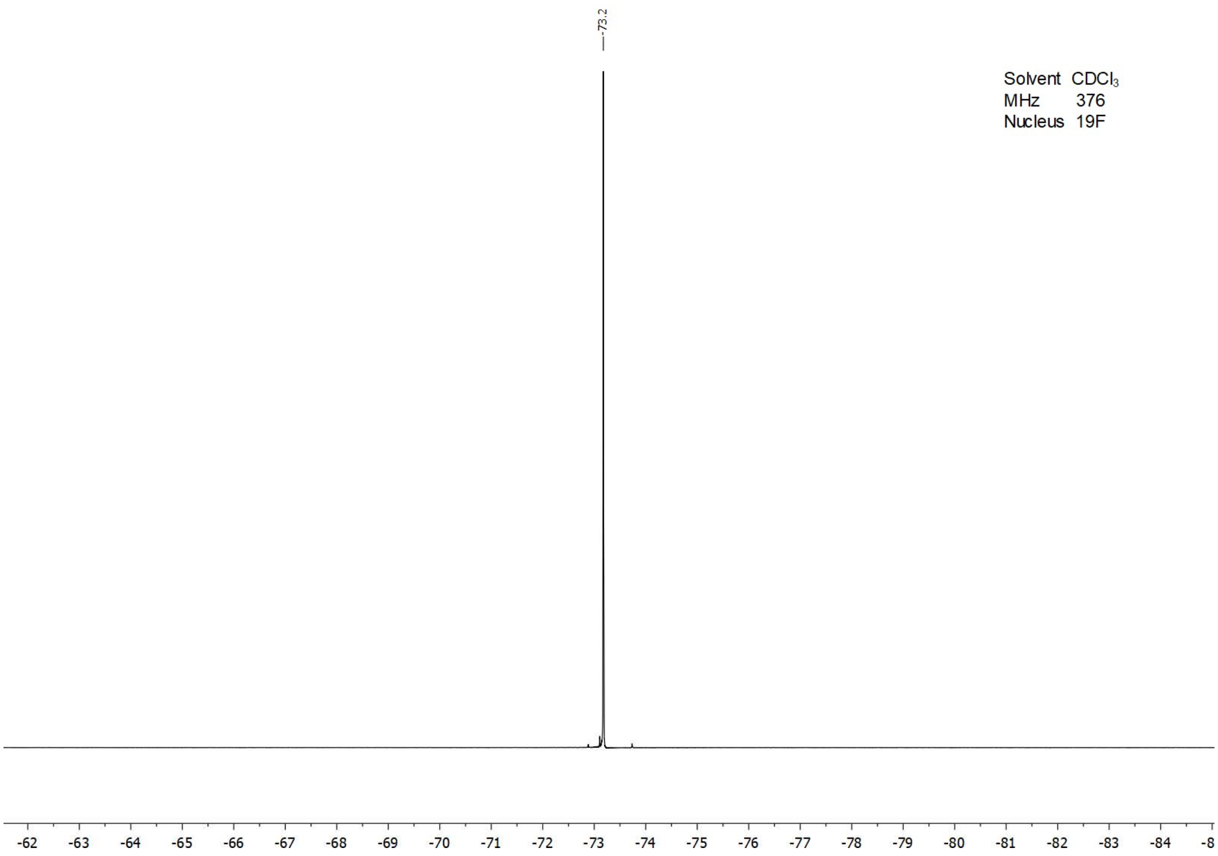
-73.2
Solvent CDCl3
MHz 376
Nucleus 19F
-62 -63 -64 -65 -66 -67 -68 -69 -70 -71 -72 -73 -74 -75 -76 -77 -78 -79 -80 -81 -82 -83 -84 -8

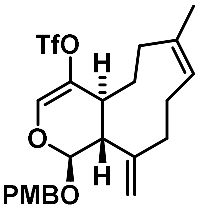

S12

Solvent CDCl$_3$
MHz 400
Nucleus 1H

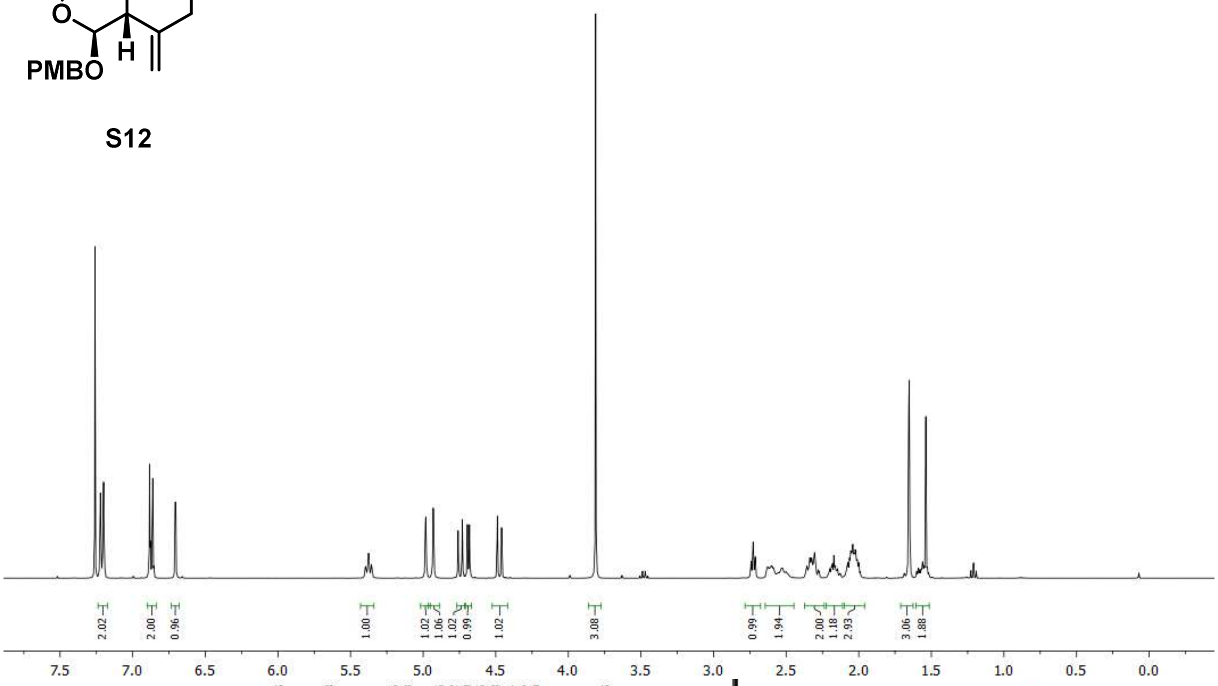

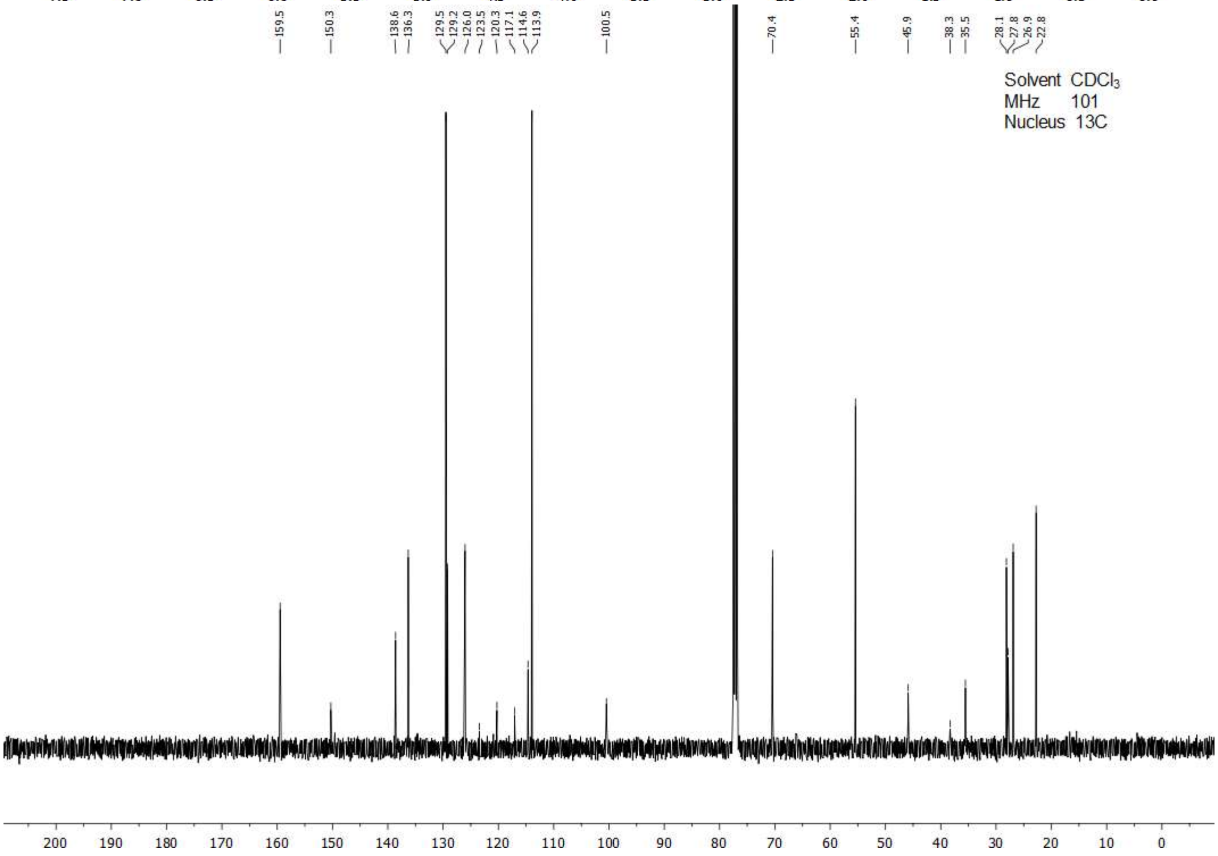

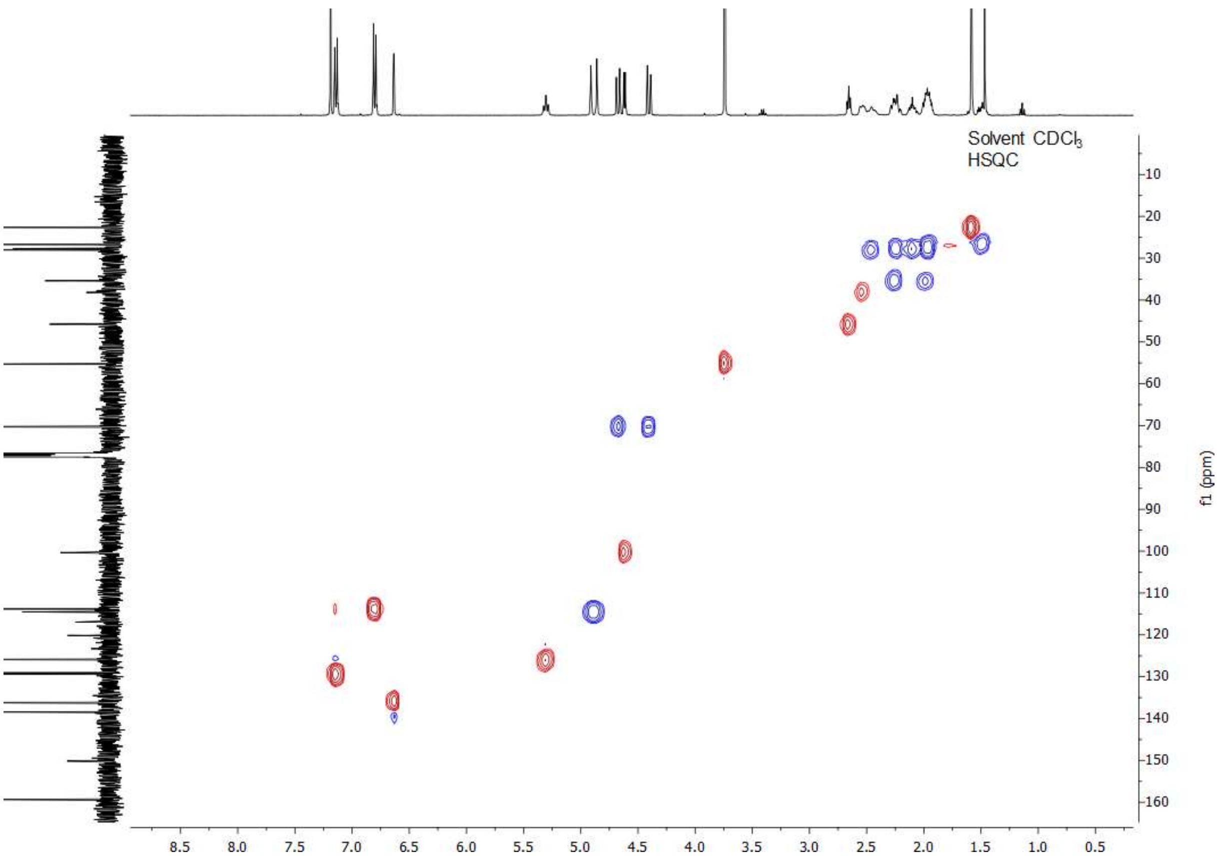
Solvent CDCl3
HSQC
f1 (ppm)

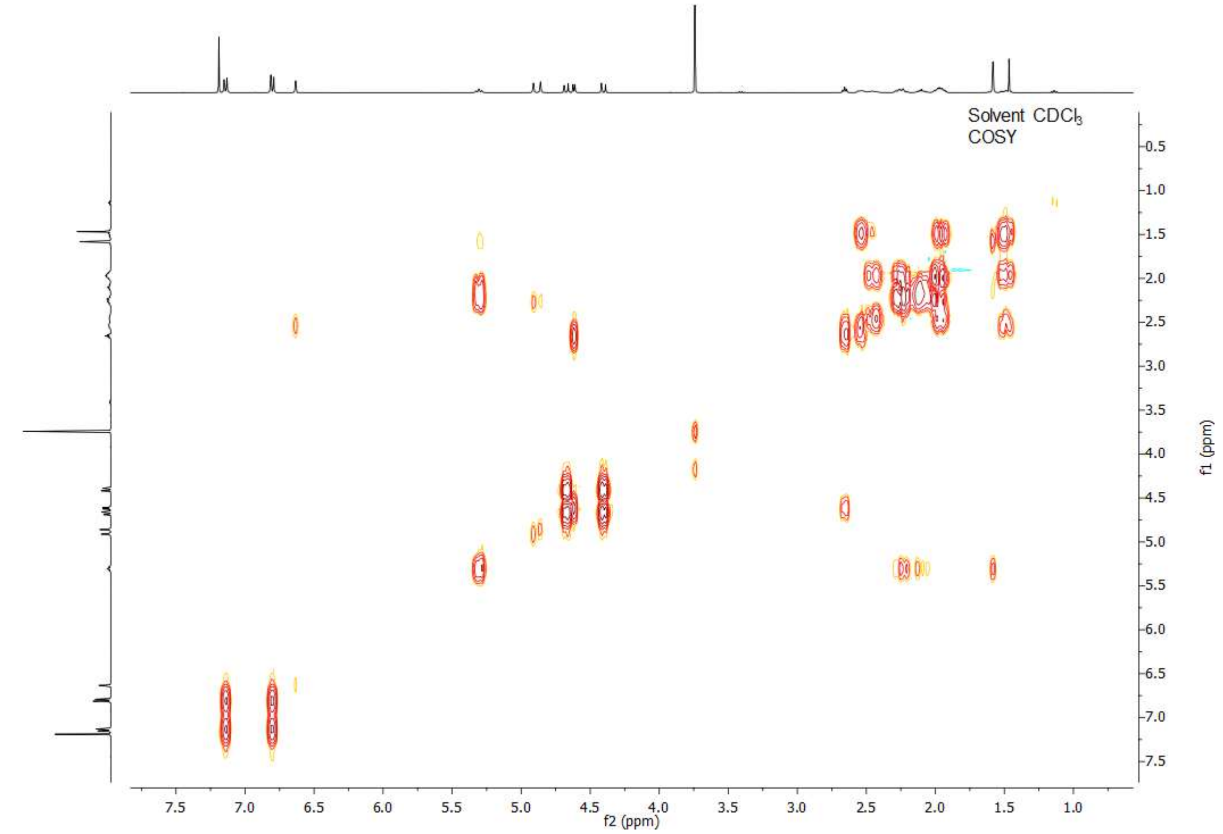
Solvent CDCl3
COSY
f2 (ppm)
f1 (ppm)

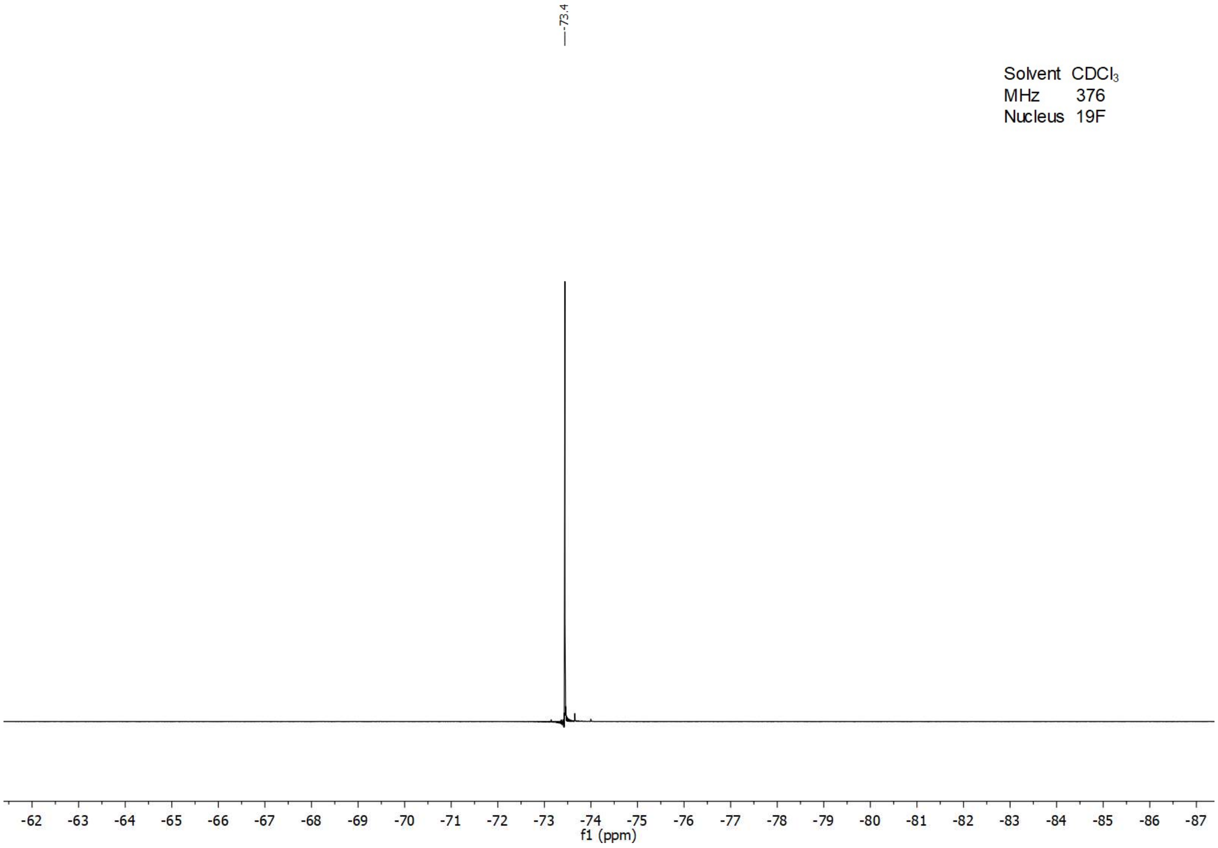
-73.4
Solvent CDCl3
MHz 376
Nucleus 19F
-62 -63 -64 -65 -66 -67 -68 -69 -70 -71 -72 -73 -74 -75 -76 -77 -78 -79 -80 -81 -82 -83 -84 -85 -86 -87
f1 (ppm)

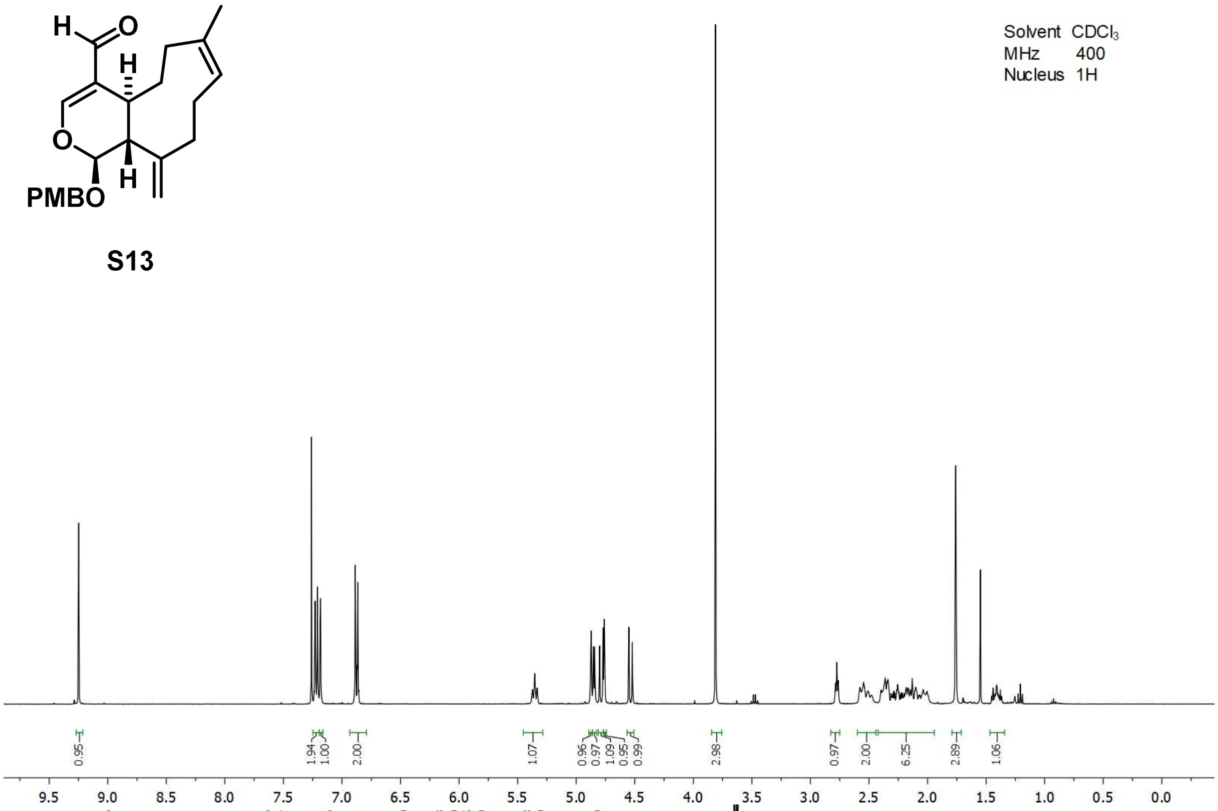
H
O
H
O
H
PMBO
S13
Solvent CDCl3
MHz 400
Nucleus 1H
0.95
1.94
1.00
2.00
1.07
0.96
0.97
1.09
0.95
0.99
2.98
0.97
2.00
6.25
2.89
1.06

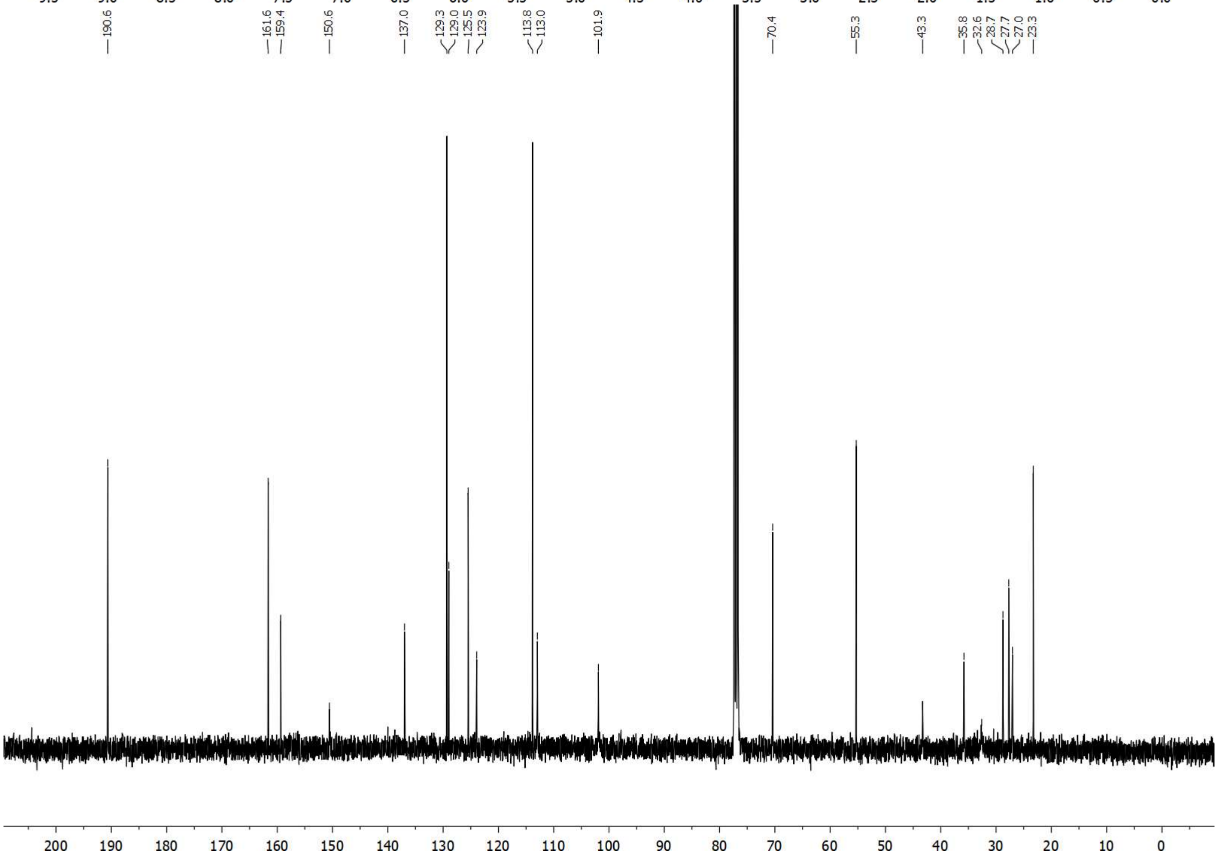
190.6
161.6
159.4
150.6
137.0
129.3
129.0
125.5
123.9
113.8
113.0
101.9
70.4
55.3
43.3
35.8
32.6
28.7
27.7
27.0
23.3

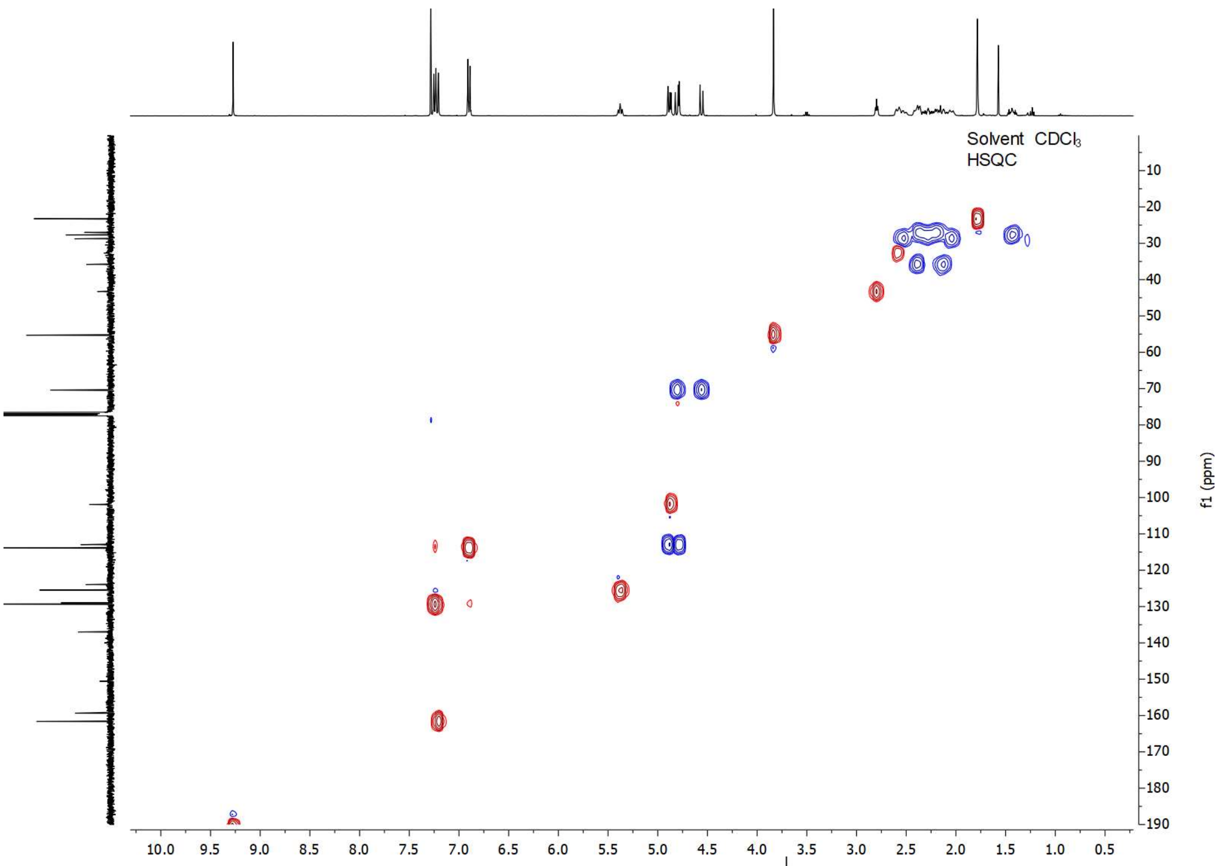
Solvent CDCl3
HSQC
f1 (ppm)

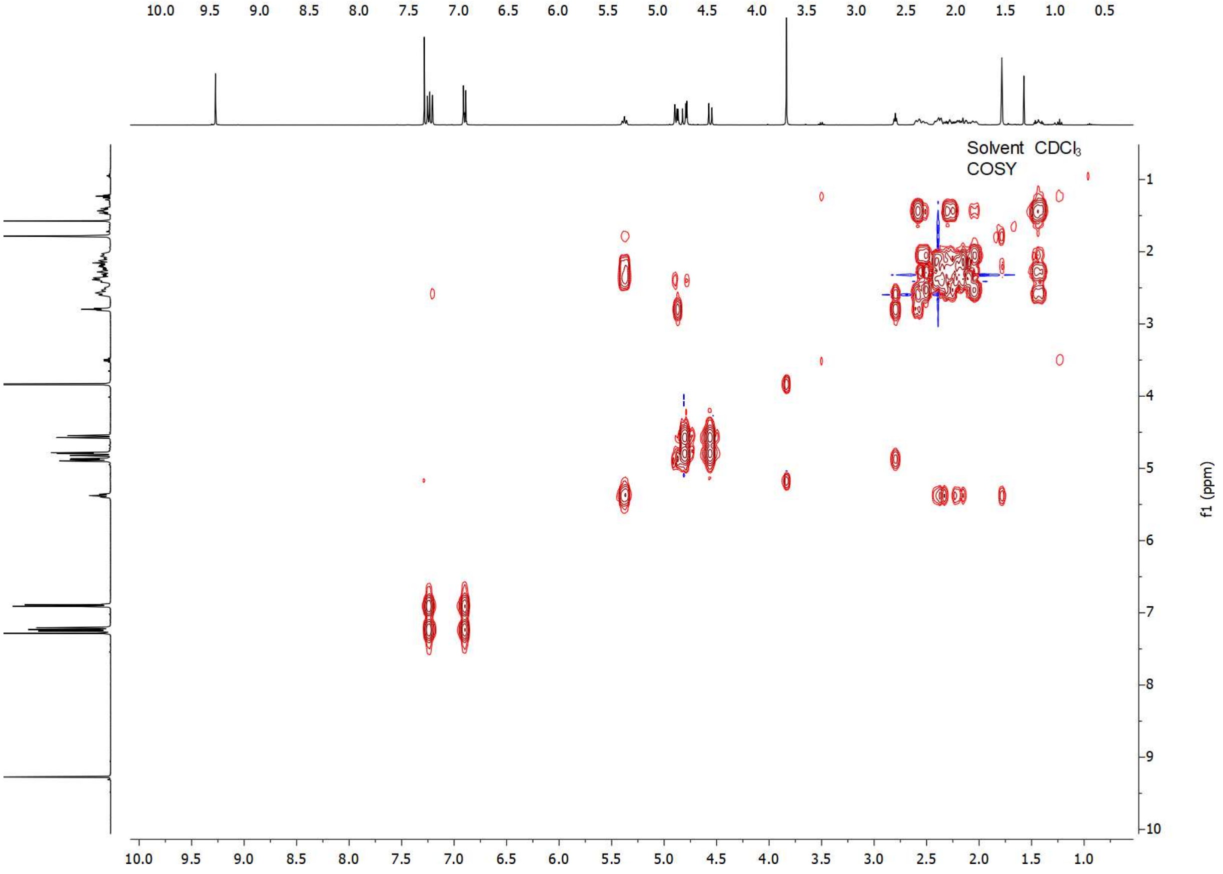
Solvent CDCl3
COSY
f1 (ppm)

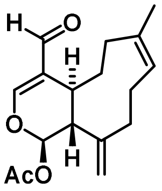

S15

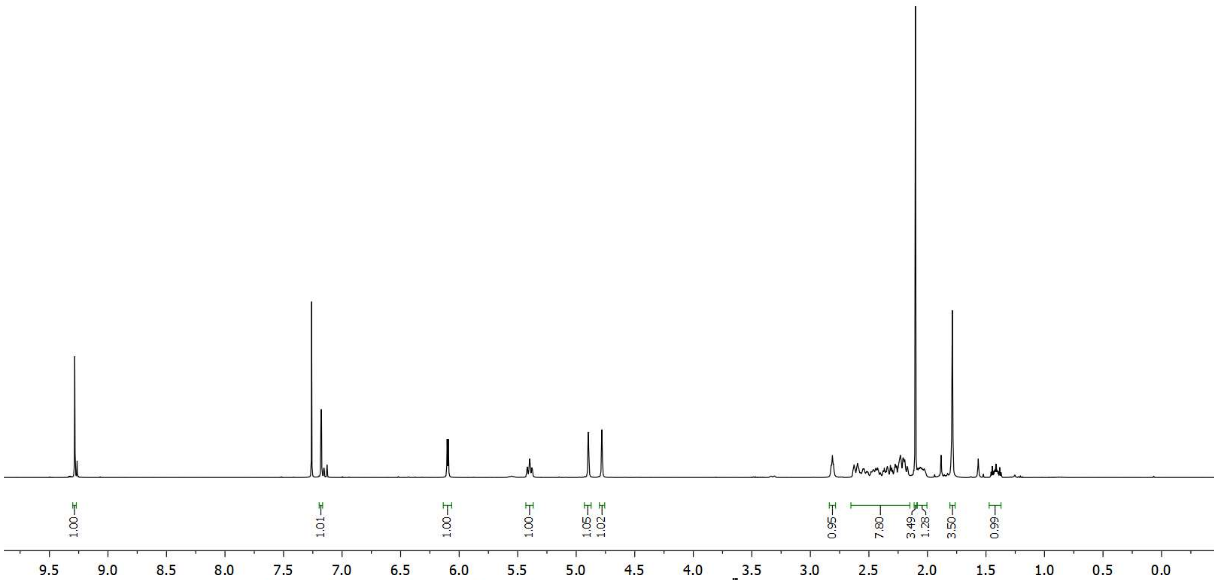

Solvent CDCl3
MHz 400
Nucleus 1H
1.00
1.01
1.00
1.00
1.05
1.02
0.95
7.80
3.49
1.28
3.50
0.99
9.5
9.0
8.5
8.0
7.5
7.0
6.5
6.0
5.5
5.0
4.5
4.0
3.5
3.0
2.5
2.0
1.5
1.0
0.5
0.0

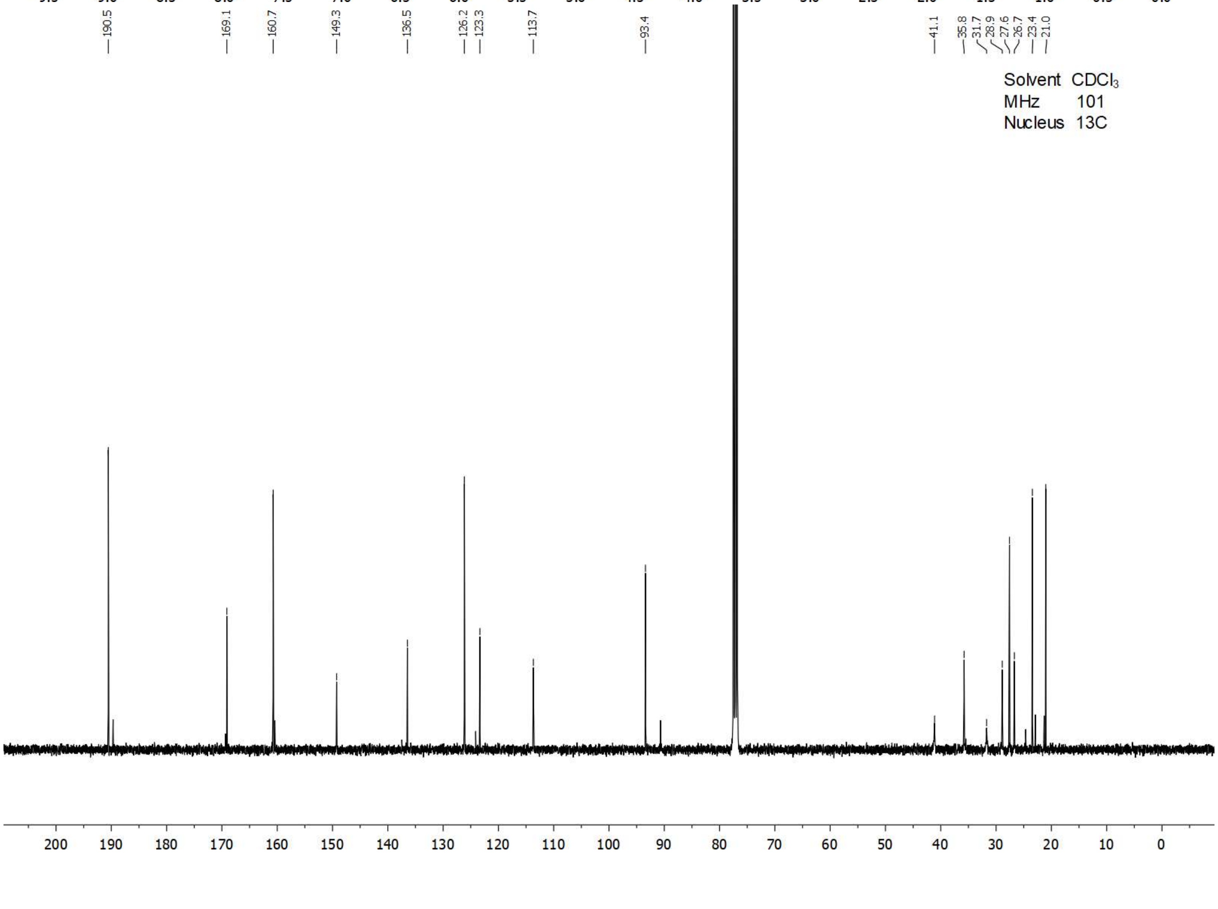

190.5
169.1
160.7
149.3
136.5
126.2
123.3
113.7
93.4
41.1
35.8
31.7
28.9
27.6
26.7
23.4
21.0
Solvent CDCl3
MHz 101
Nucleus 13C
200
190
180
170
160
150
140
130
120
110
100
90
80
70
60
50
40
30
20
10
0

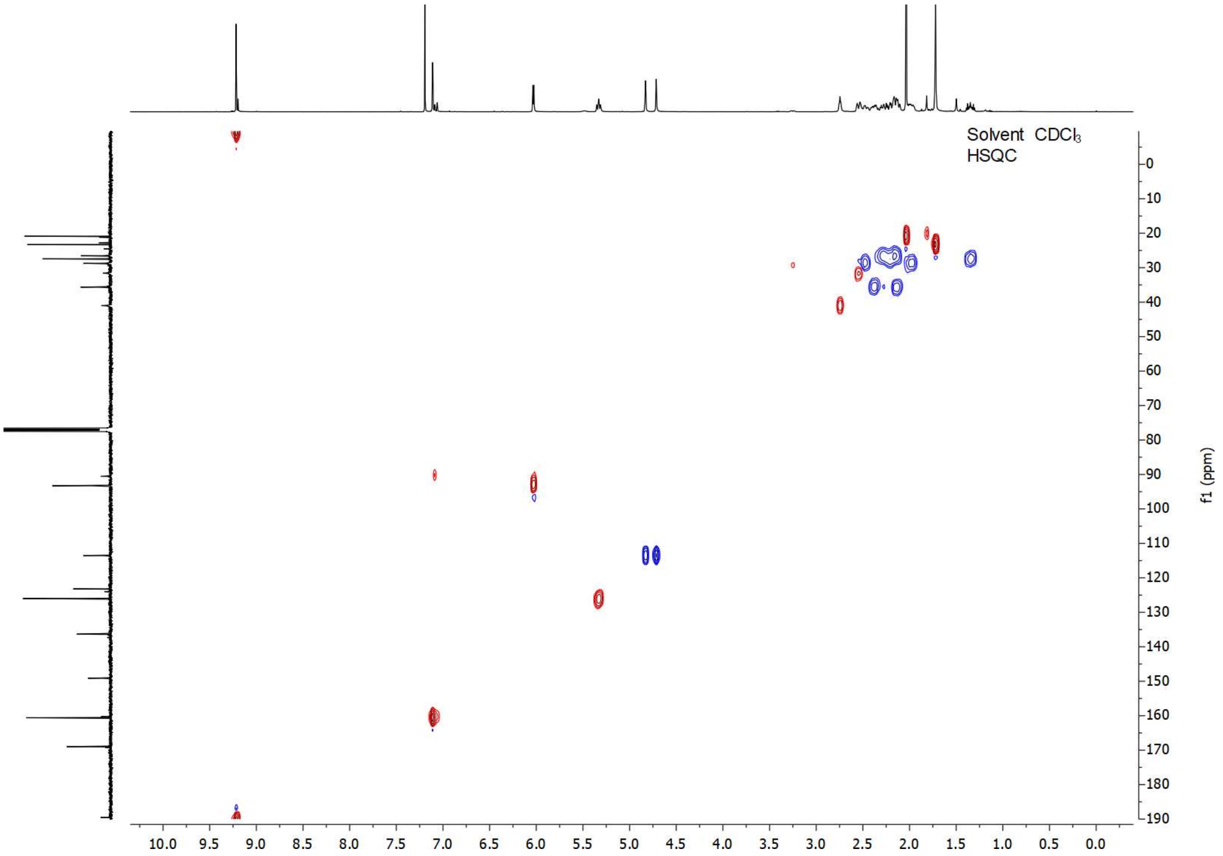
Solvent CDCl3
HSQC
f1 (ppm)

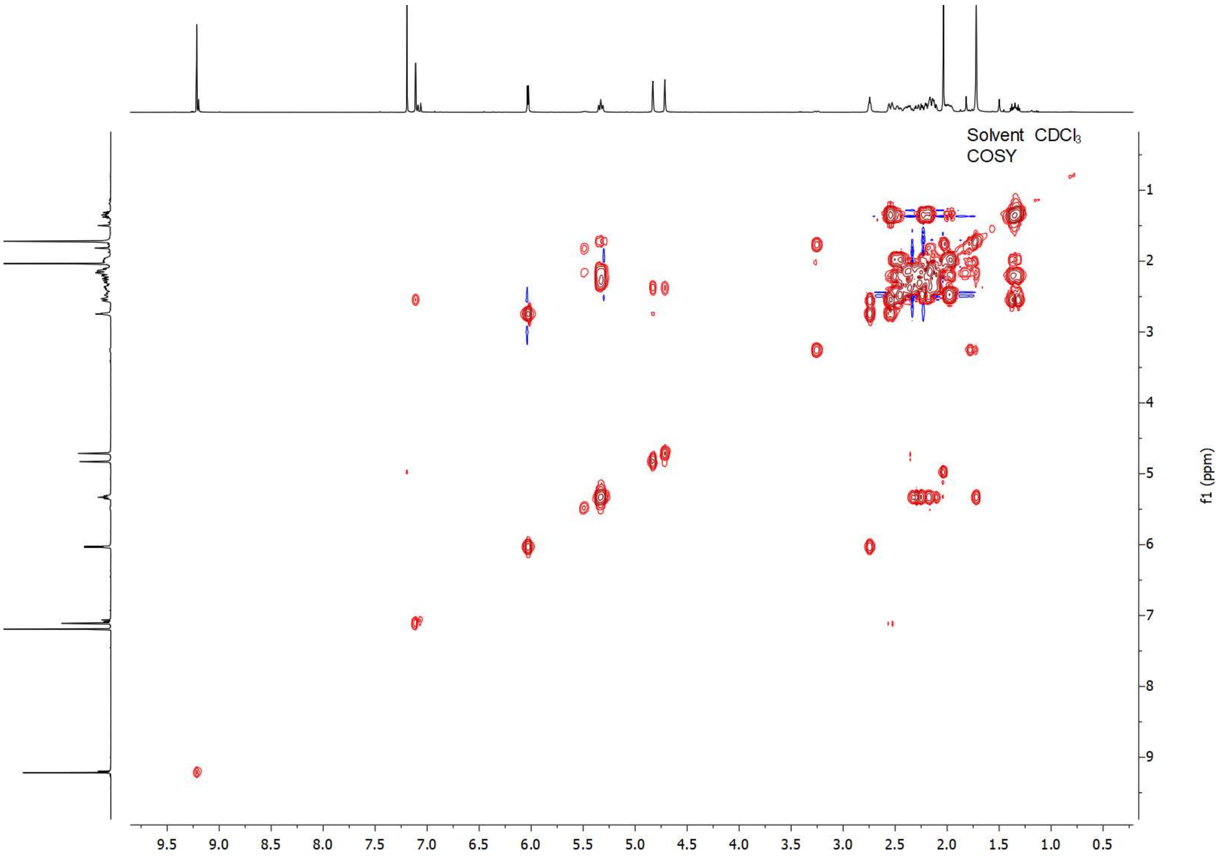
Solvent CDCl3
COSY
f1 (ppm)

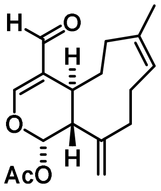
H
O
H
O
H
AcO

22

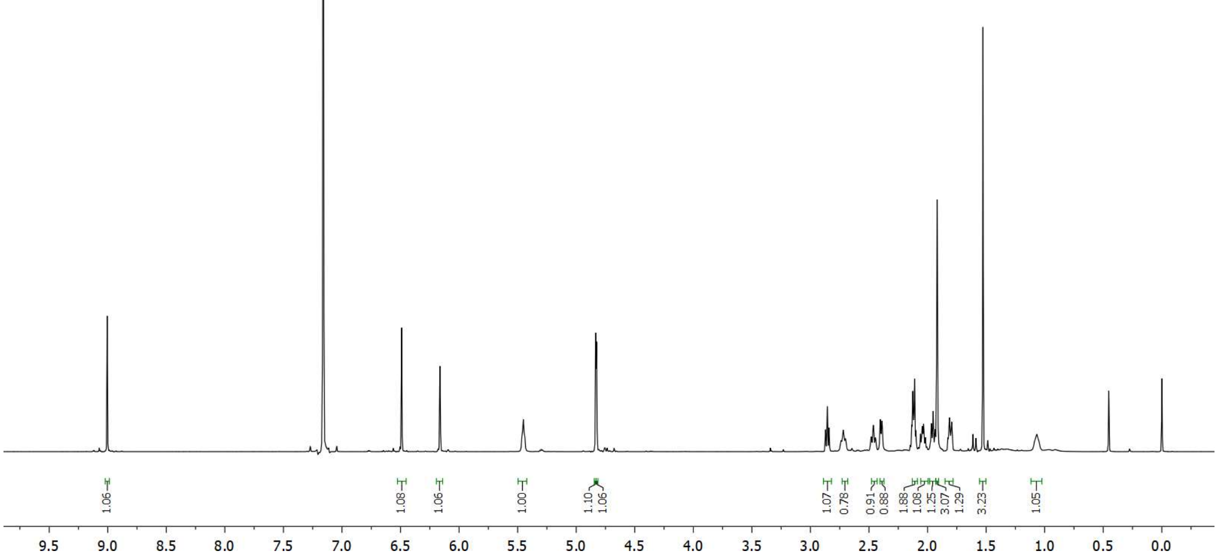
Solvent C6D6
MHz 700
Nucleus 1H
Temp 70 °C
1.06
1.08
1.06
1.00
1.10
1.06
1.07
0.78
0.91
0.88
1.88
1.08
1.25
3.07
1.29
3.23
1.05
9.5 9.0 8.5 8.0 7.5 7.0 6.5 6.0 5.5 5.0 4.5 4.0 3.5 3.0 2.5 2.0 1.5 1.0 0.5 0.0

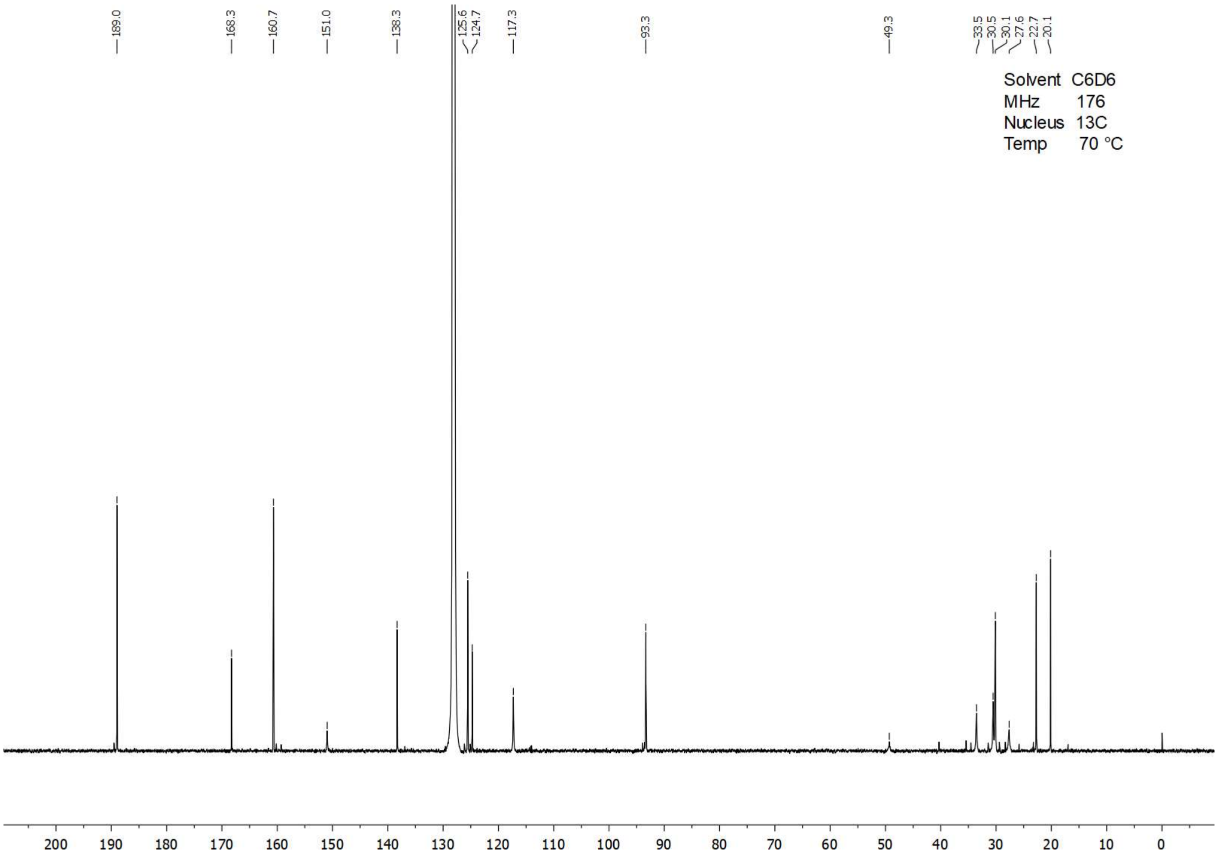
189.0
168.3
160.7
151.0
138.3
125.6
124.7
117.3
93.3
49.3
33.5
30.5
30.1
27.6
22.7
20.1
Solvent C6D6
MHz 176
Nucleus 13C
Temp 70 °C
200 190 180 170 160 150 140 130 120 110 100 90 80 70 60 50 40 30 20 10 0

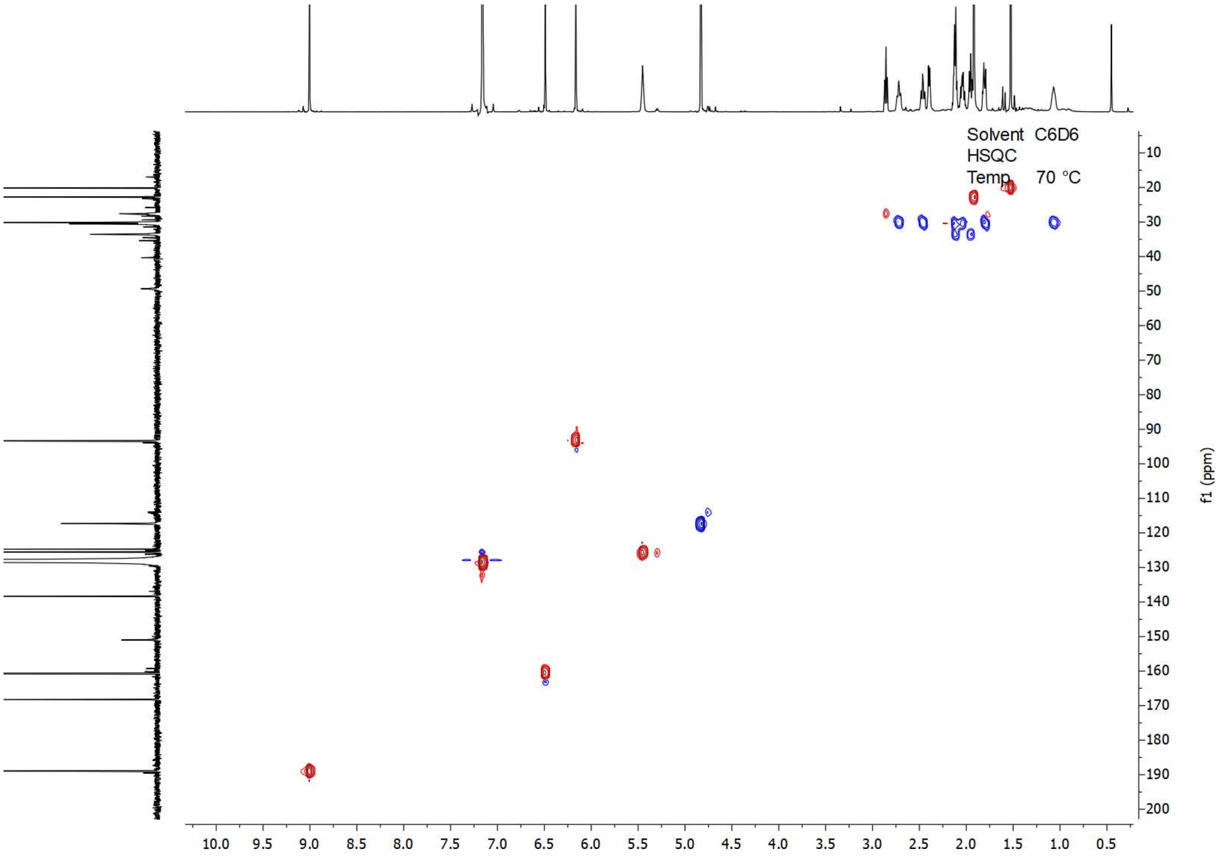
Solvent C6D6
HSQC
Temp 70 °C
f1 (ppm)

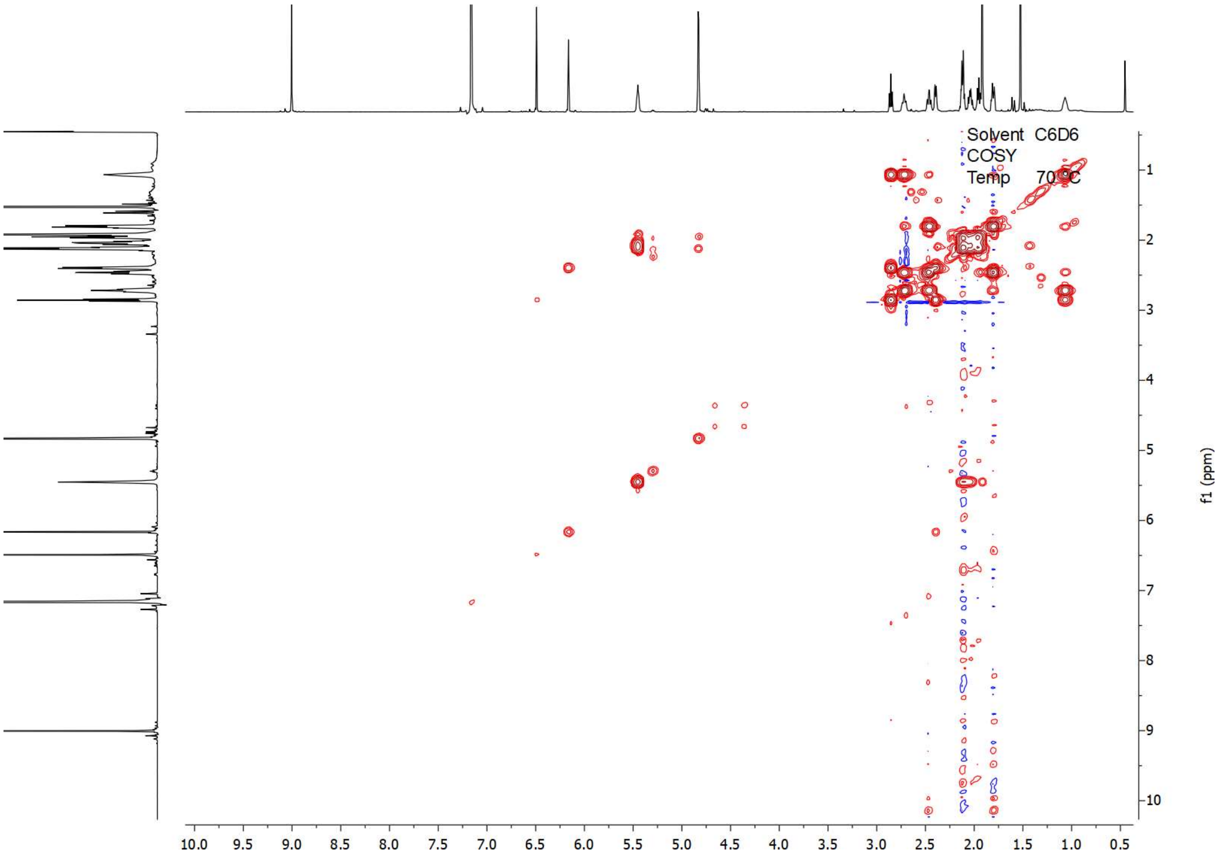
Solvent C6D6
COSY
Temp 70 °C
f1 (ppm)

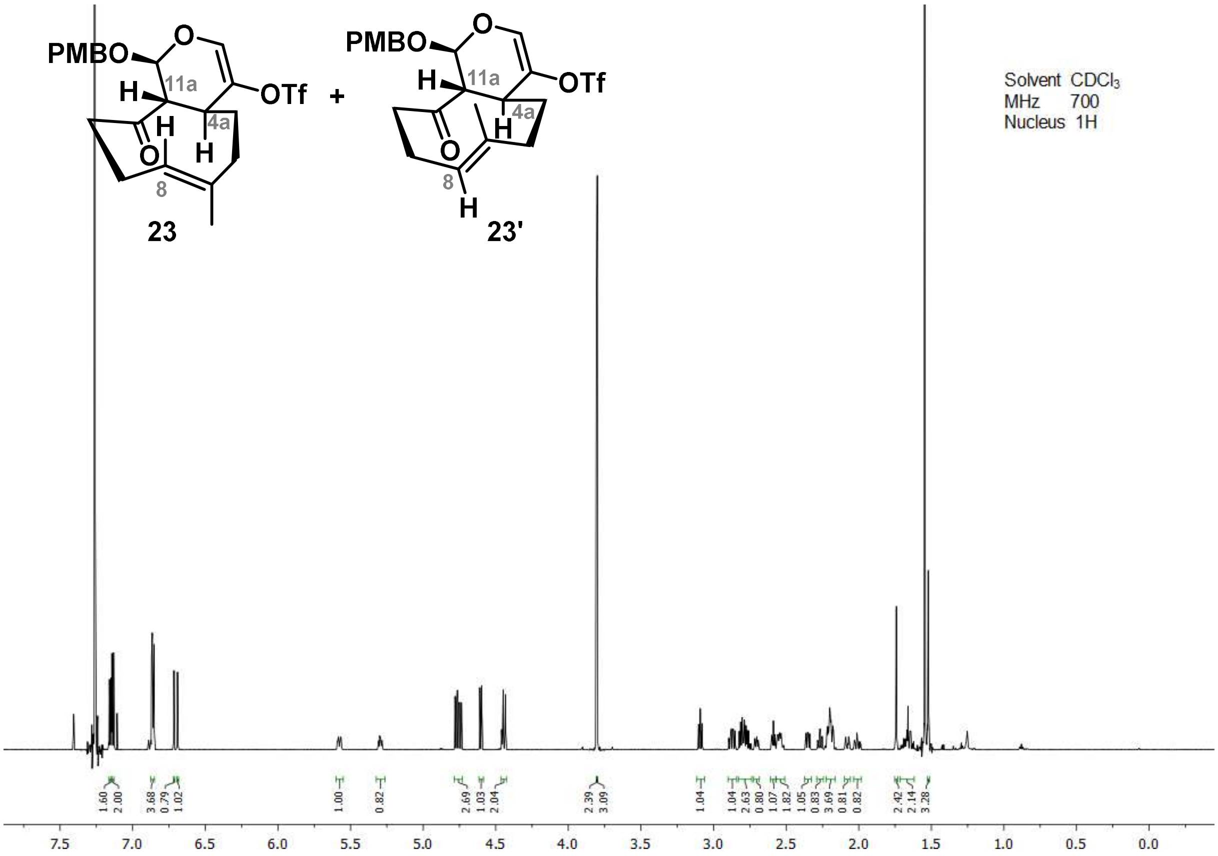
PMBO
O
OTf
H
11a
4a
H
H
O
8
23
+
PMBO
O
OTf
H
11a
4a
H
O
8
H
23'
Solvent CDCl3
MHz 700
Nucleus 1H
1.60
2.00
3.68
0.79
1.02
1.00
0.82
2.69
1.03
2.04
2.39
3.09
1.04
1.04
2.63
0.80
1.07
1.82
1.05
0.83
3.69
0.81
0.82
2.42
2.14
3.28
7.5
7.0
6.5
6.0
5.5
5.0
4.5
4.0
3.5
3.0
2.5
2.0
1.5
1.0
0.5
0.0

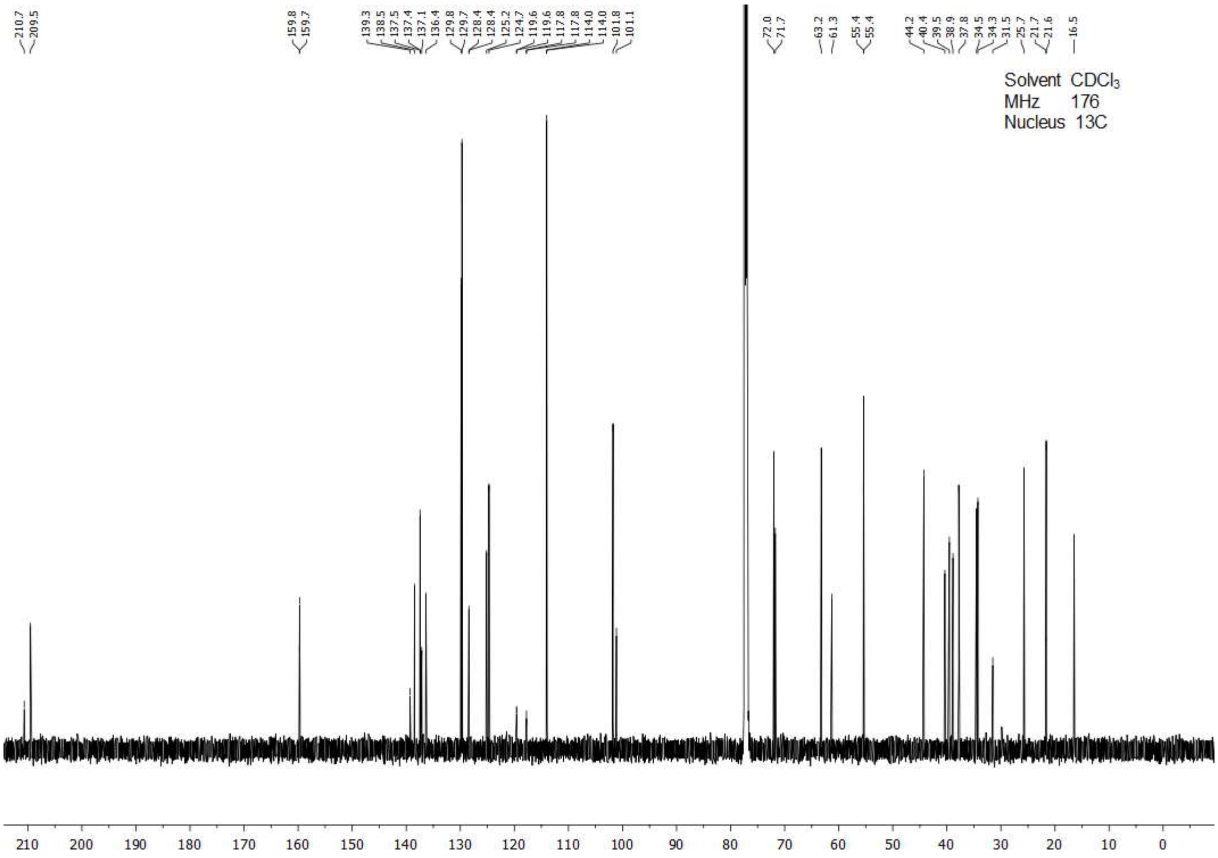
210.7
209.5
159.8
159.7
139.3
138.5
137.5
137.4
137.1
136.4
129.8
129.7
128.4
128.4
125.2
124.7
119.6
119.6
117.8
117.8
114.0
114.0
101.8
101.1
72.0
71.7
63.2
61.3
55.4
55.4
44.2
40.4
39.5
38.9
37.8
34.5
34.3
31.5
25.7
21.7
21.6
16.5
Solvent CDCl3
MHz 176
Nucleus 13C
210
200
190
180
170
160
150
140
130
120
110
100
90
80
70
60
50
40
30
20
10
0

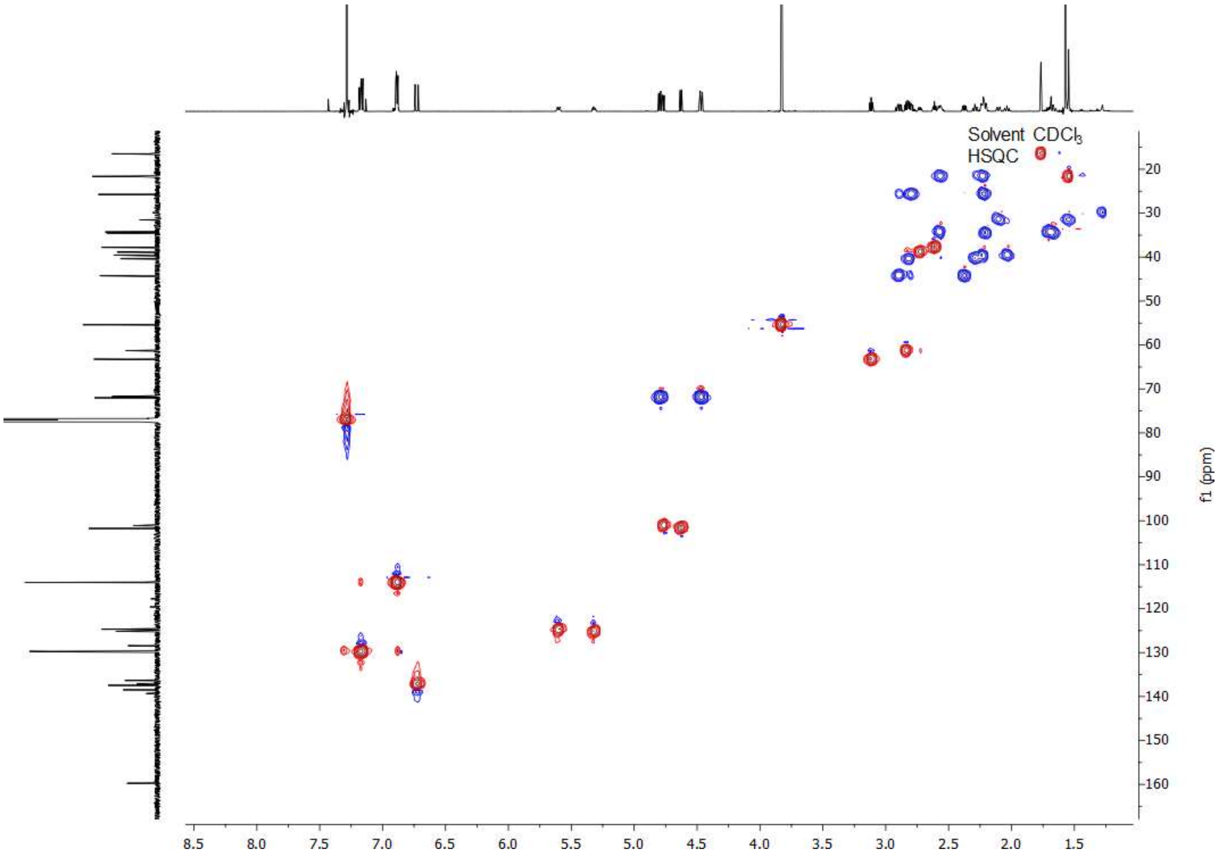
Solvent CDCl3
HSQC
f1 (ppm)

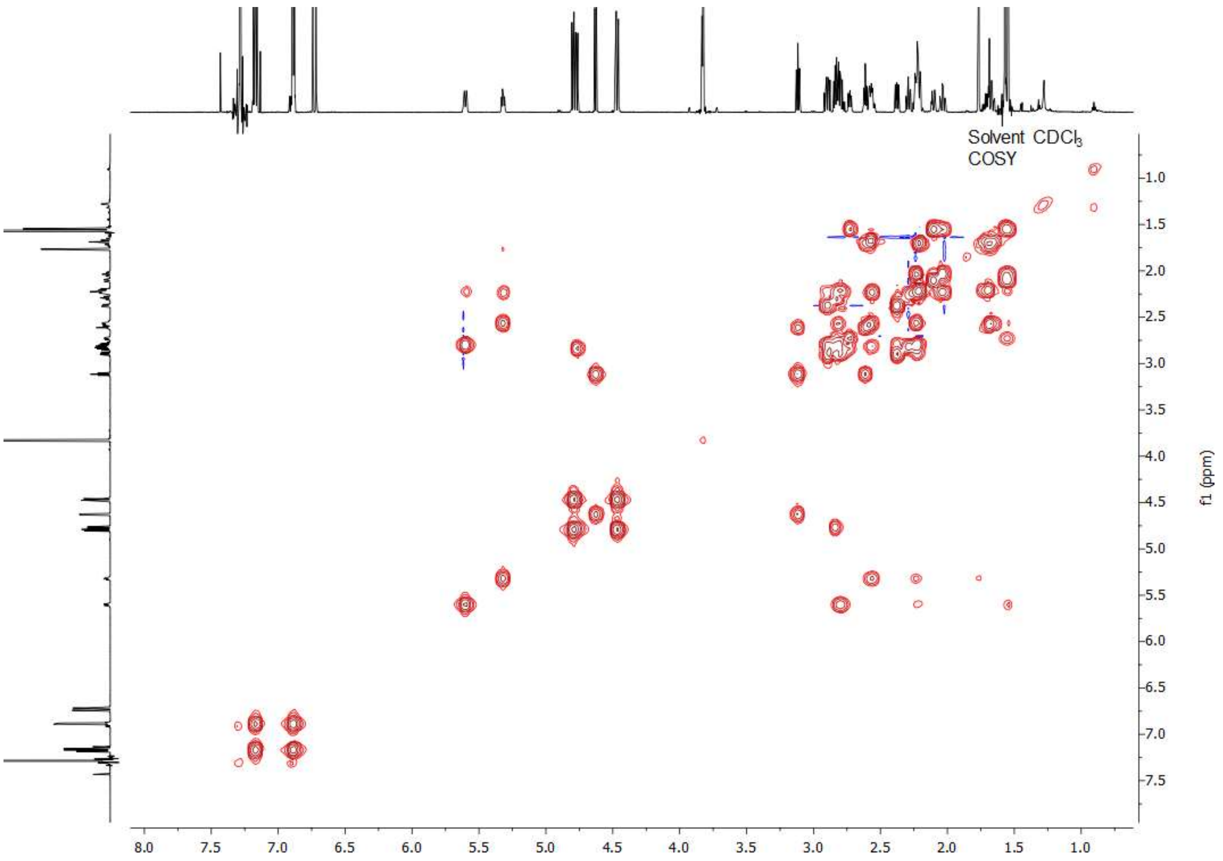
Solvent CDCl3
COSY
f1 (ppm)

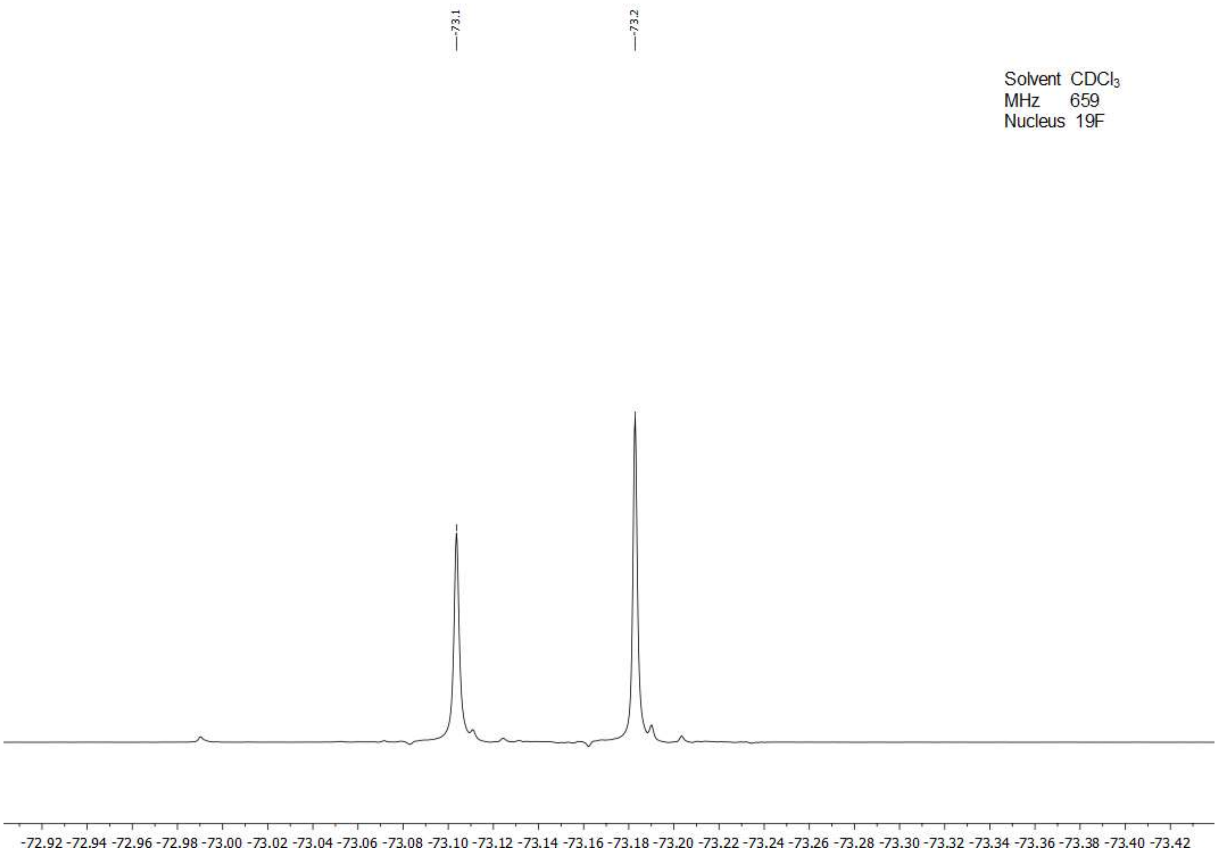

—73.1
—73.2
Solvent CDCl3
MHz 659
Nucleus 19F
-72.92 -72.94 -72.96 -72.98 -73.00 -73.02 -73.04 -73.06 -73.08 -73.10 -73.12 -73.14 -73.16 -73.18 -73.20 -73.22 -73.24 -73.26 -73.28 -73.30 -73.32 -73.34 -73.36 -73.38 -73.40 -73.42

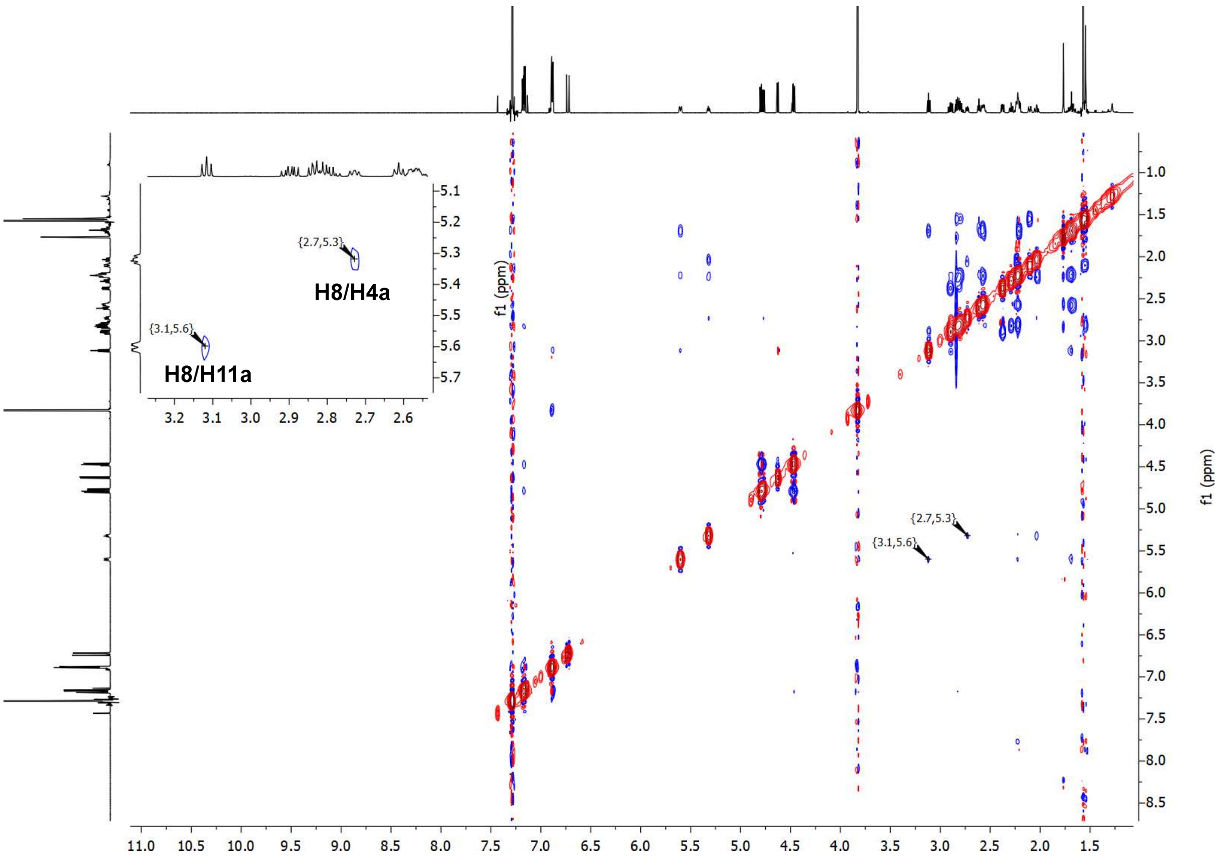

{2.7,5.3}
{3.1,5.6}
H8/H4a
H8/H11a
f1 (ppm)

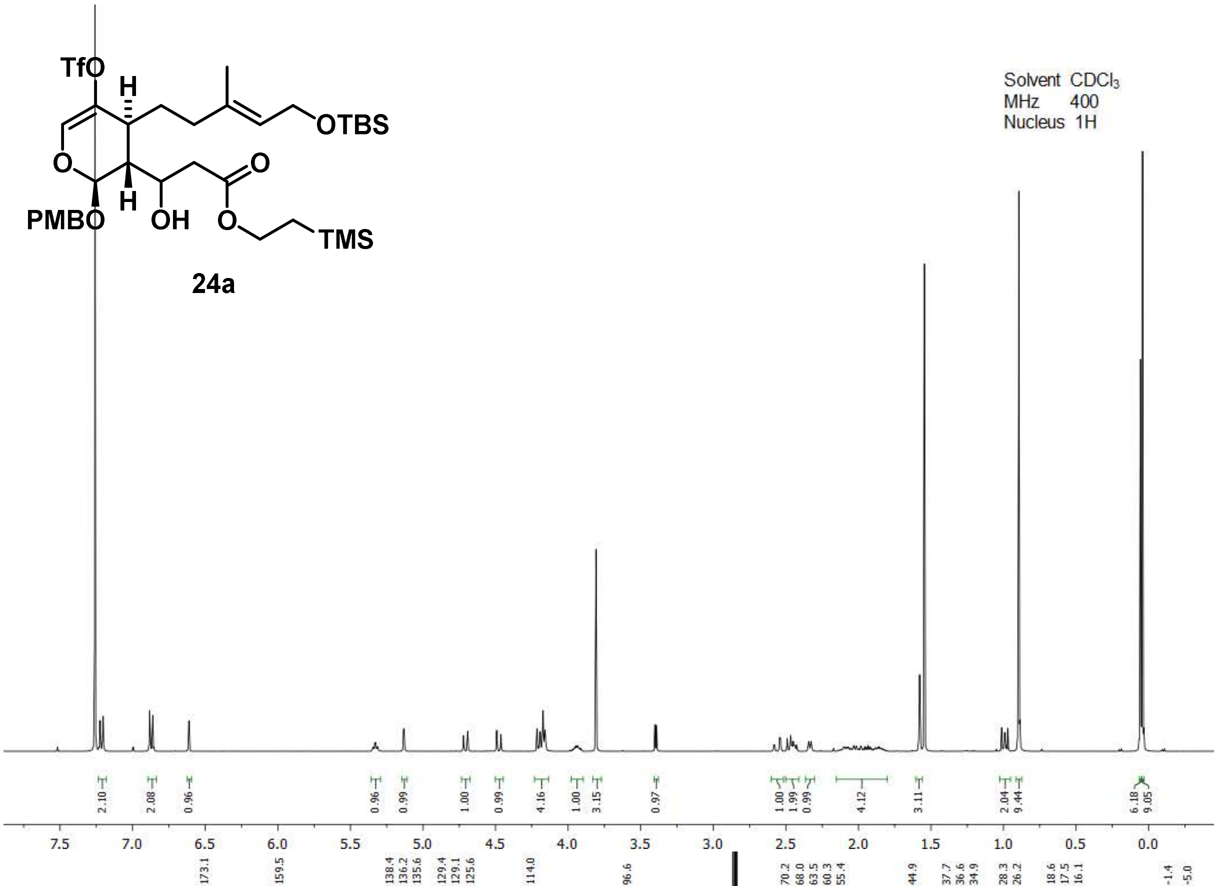
TfO
H
OTBS
O
O
H
PMBO
OH
O
TMS

24a

Solvent CDCl$_3$
MHz 400
Nucleus 1H

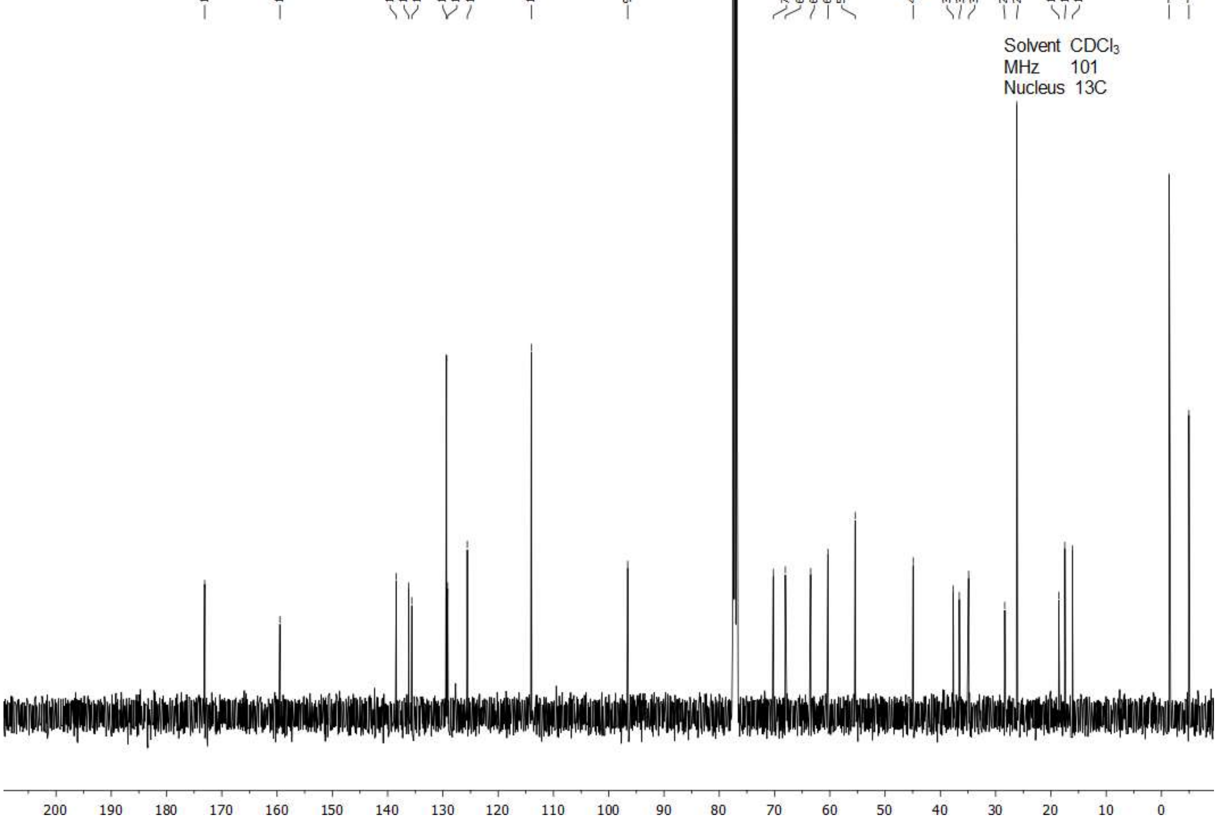
Solvent CDCl$_3$
MHz 101
Nucleus 13C

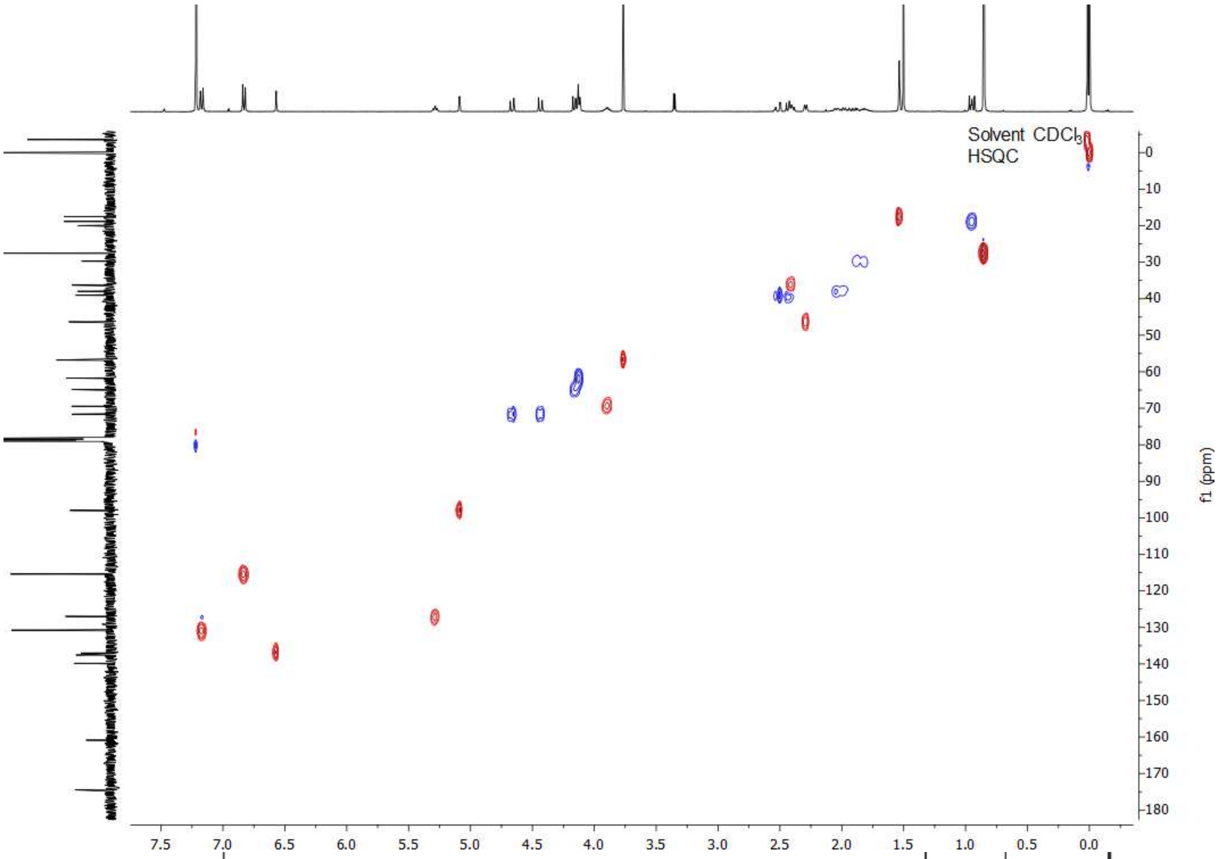
Solvent CDCl3
HSQC
f1 (ppm)

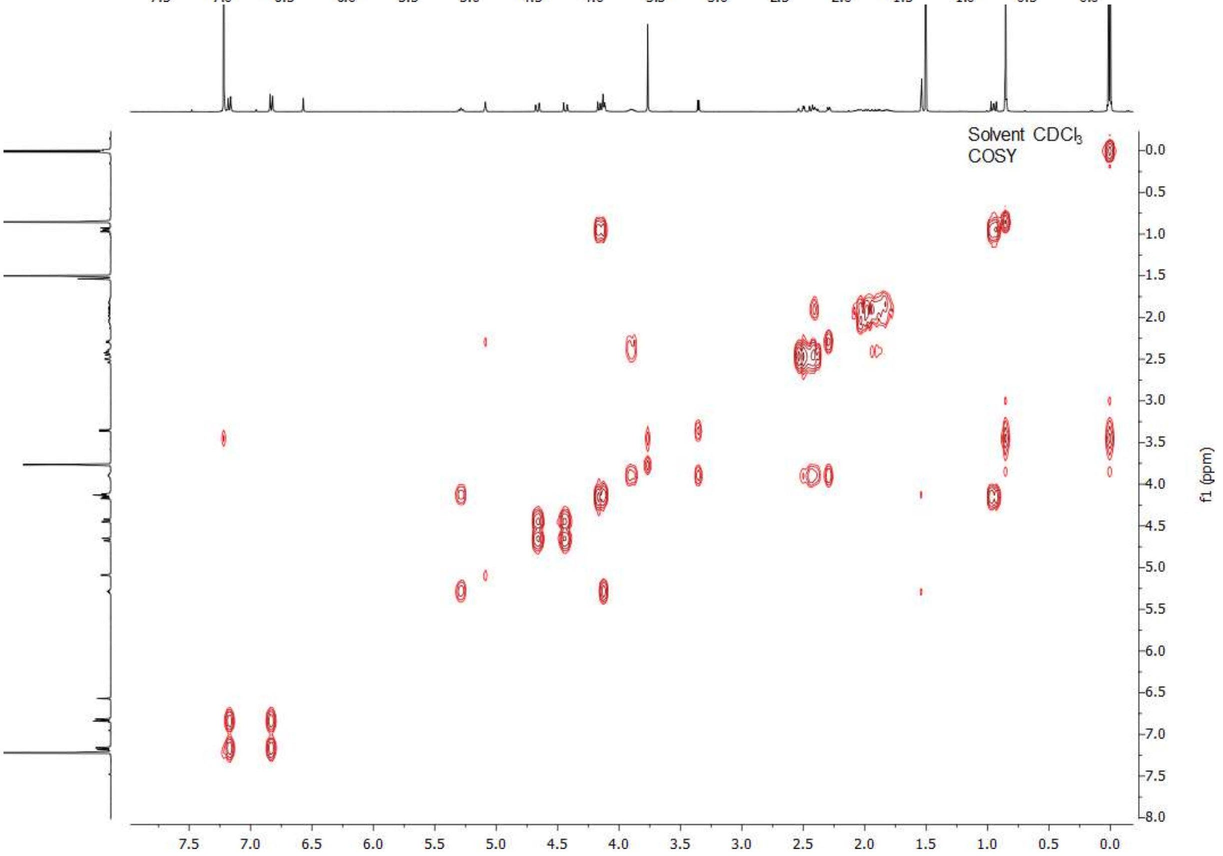
Solvent CDCl3
COSY
f1 (ppm)

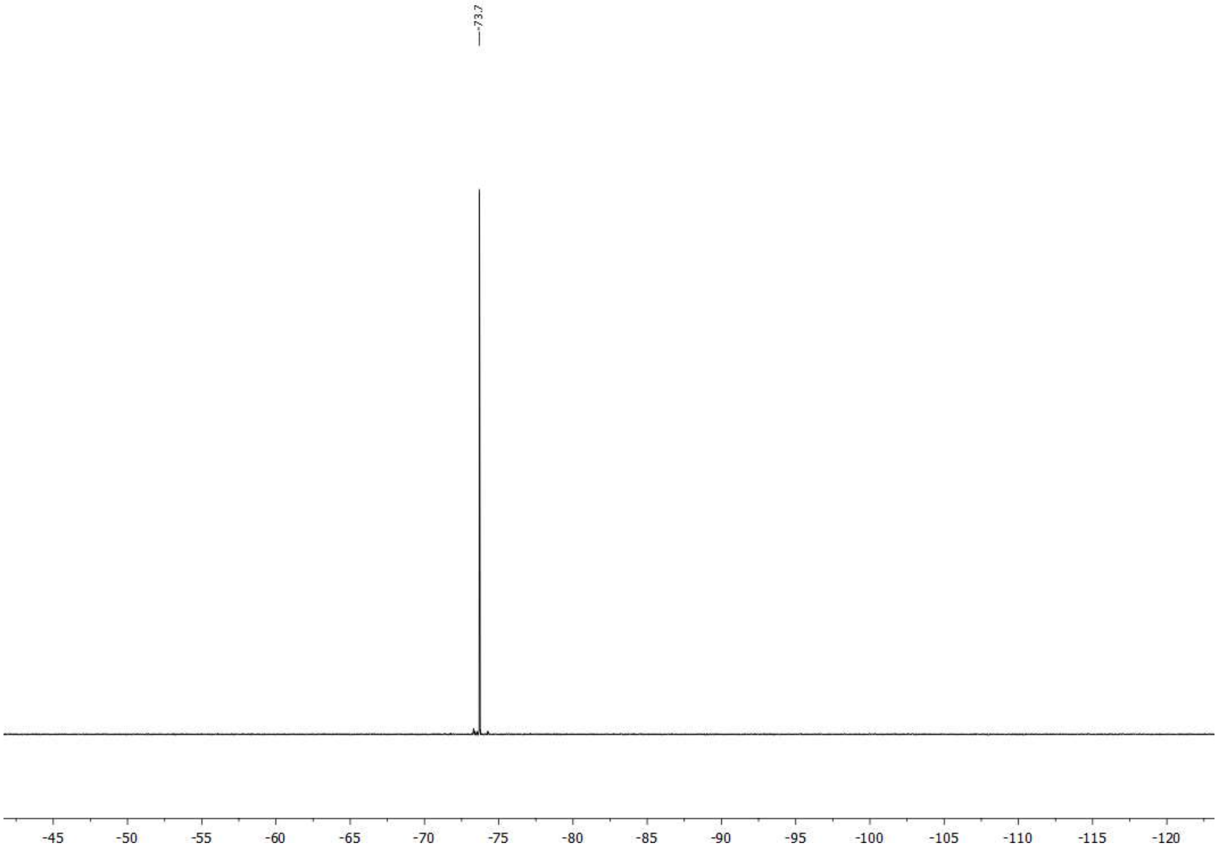
-73.7
-45
-50
-55
-60
-65
-70
-75
-80
-85
-90
-95
-100
-105
-110
-115
-120

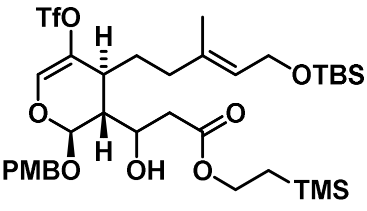
TfO
H
OTBS
O
O
PMBO
H
OH
O
TMS

24b

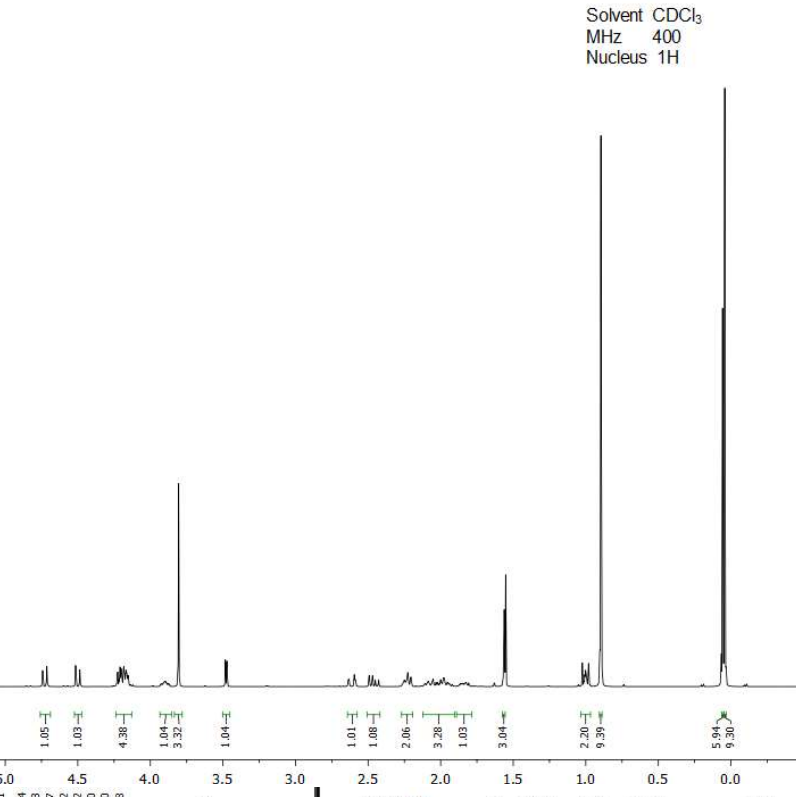
Solvent CDCl$_3$
MHz 400
Nucleus 1H

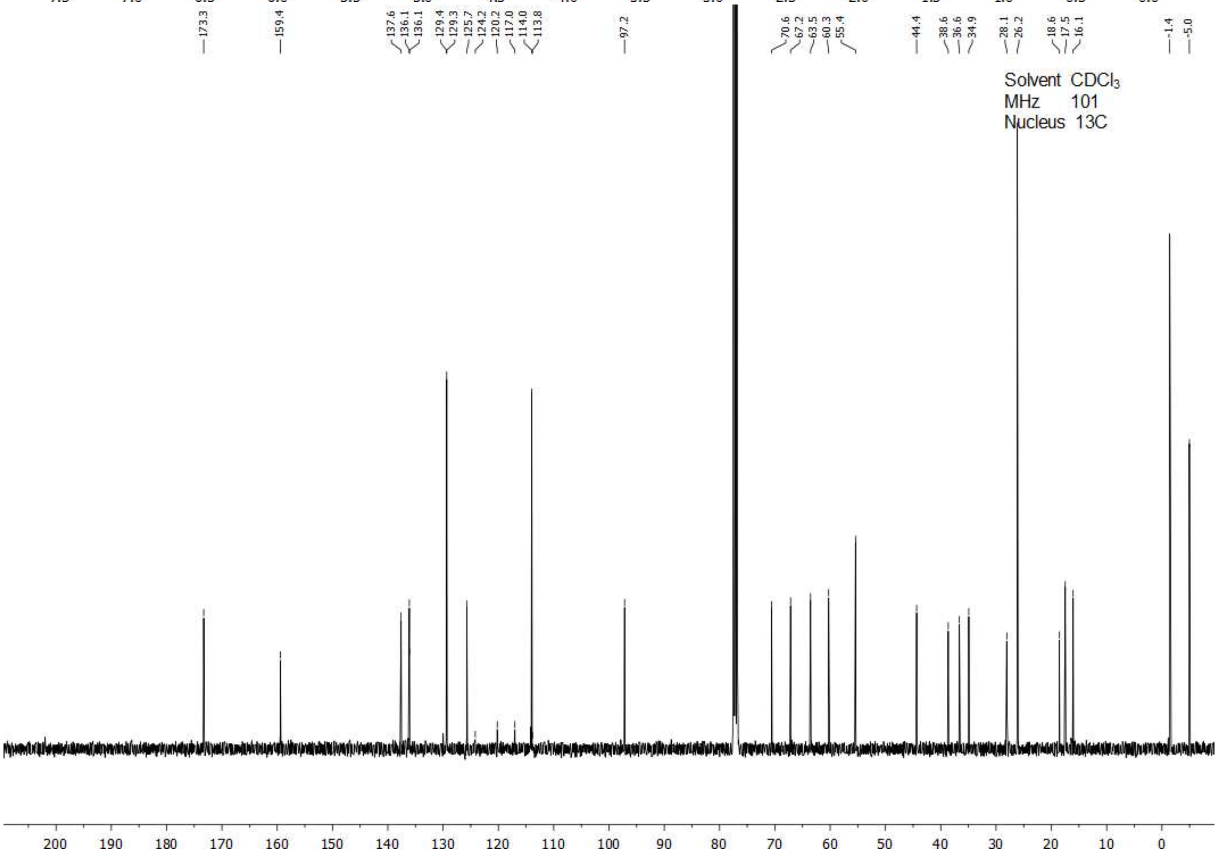
Solvent CDCl$_3$
MHz 101
Nucleus 13C

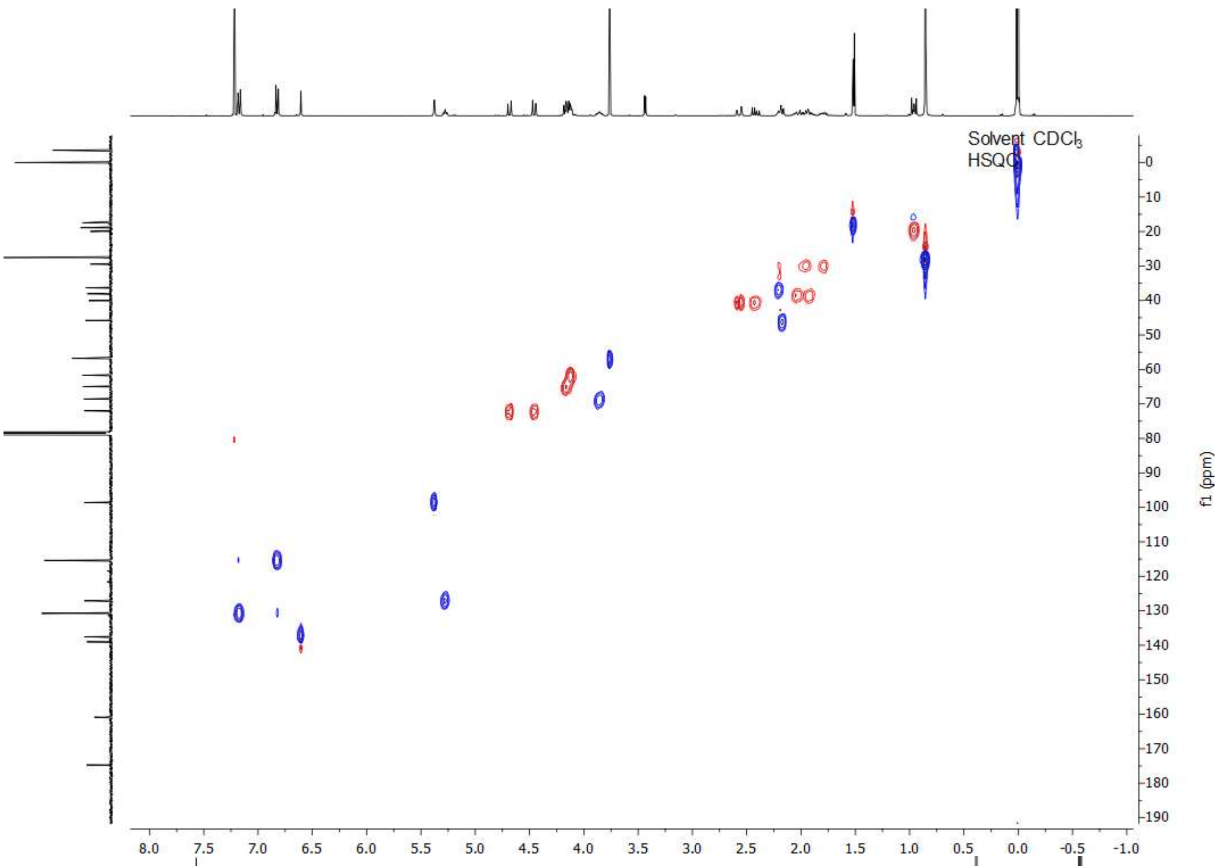
Solvent CDCl3
HSQC
f1 (ppm)

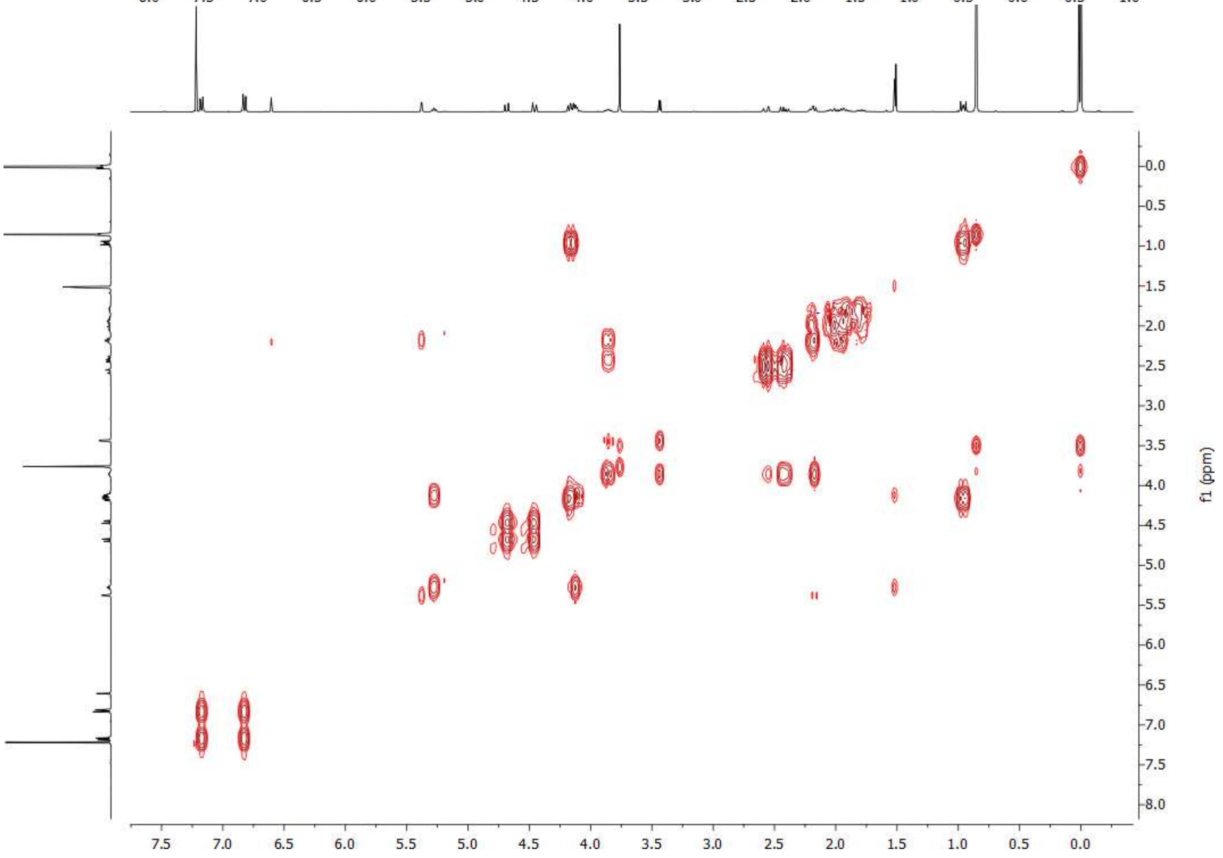
f1 (ppm)

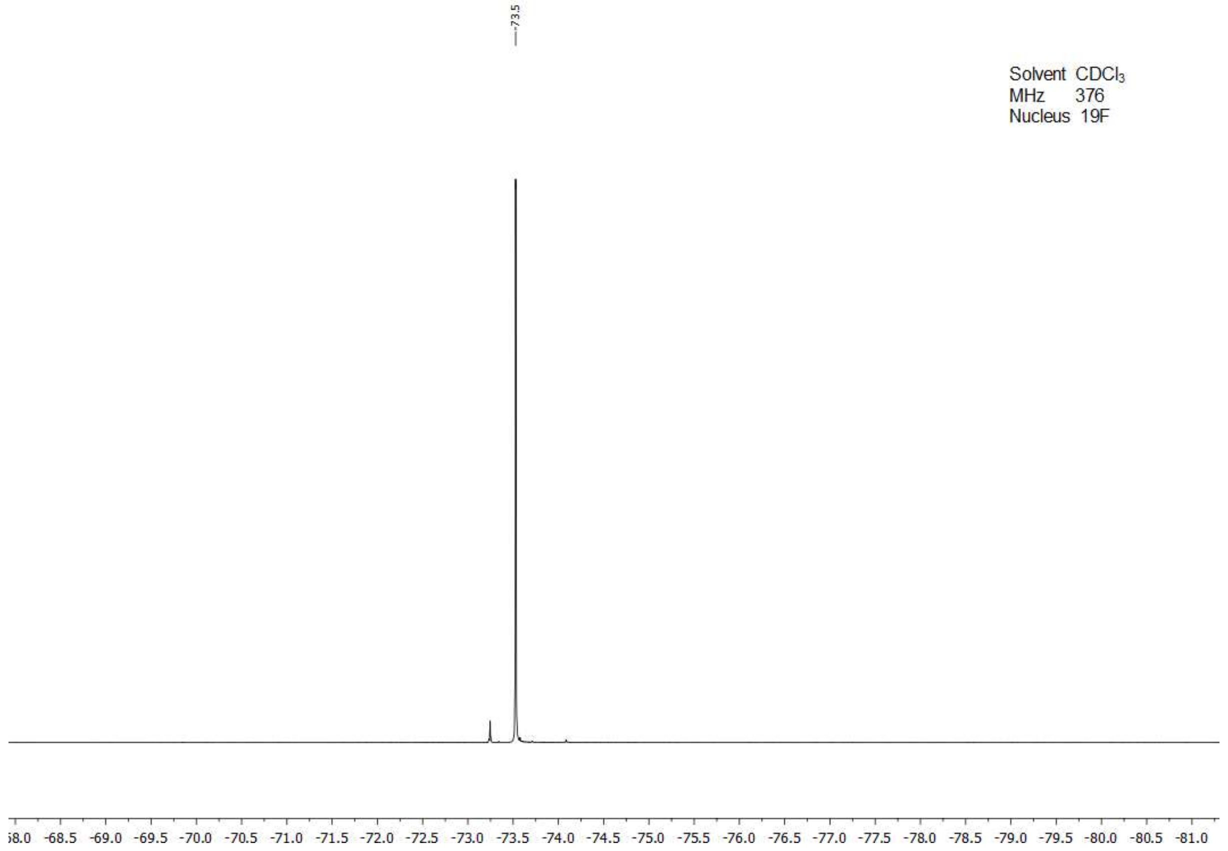

-73.5
Solvent CDCl3
MHz 376
Nucleus 19F
-68.5 -69.0 -69.5 -70.0 -70.5 -71.0 -71.5 -72.0 -72.5 -73.0 -73.5 -74.0 -74.5 -75.0 -75.5 -76.0 -76.5 -77.0 -77.5 -78.0 -78.5 -79.0 -79.5 -80.0 -80.5 -81.0

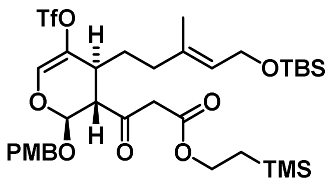
TfO
H
OTBS
O
O
H
PMBO
O
O
TMS

S18

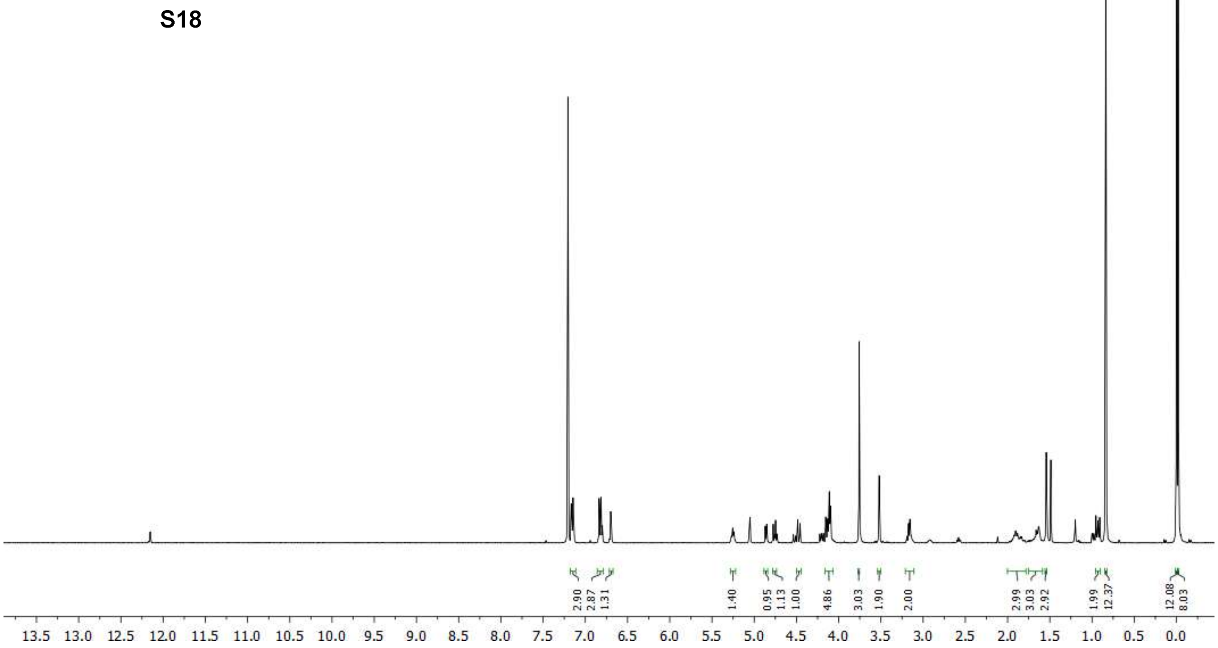
Solvent CDCl3
MHz 400
Nucleus 1H
2.90
2.87
1.31
1.40
0.95
1.13
1.00
4.86
3.03
1.90
2.00
2.99
3.03
2.92
1.99
12.37
12.08
8.03

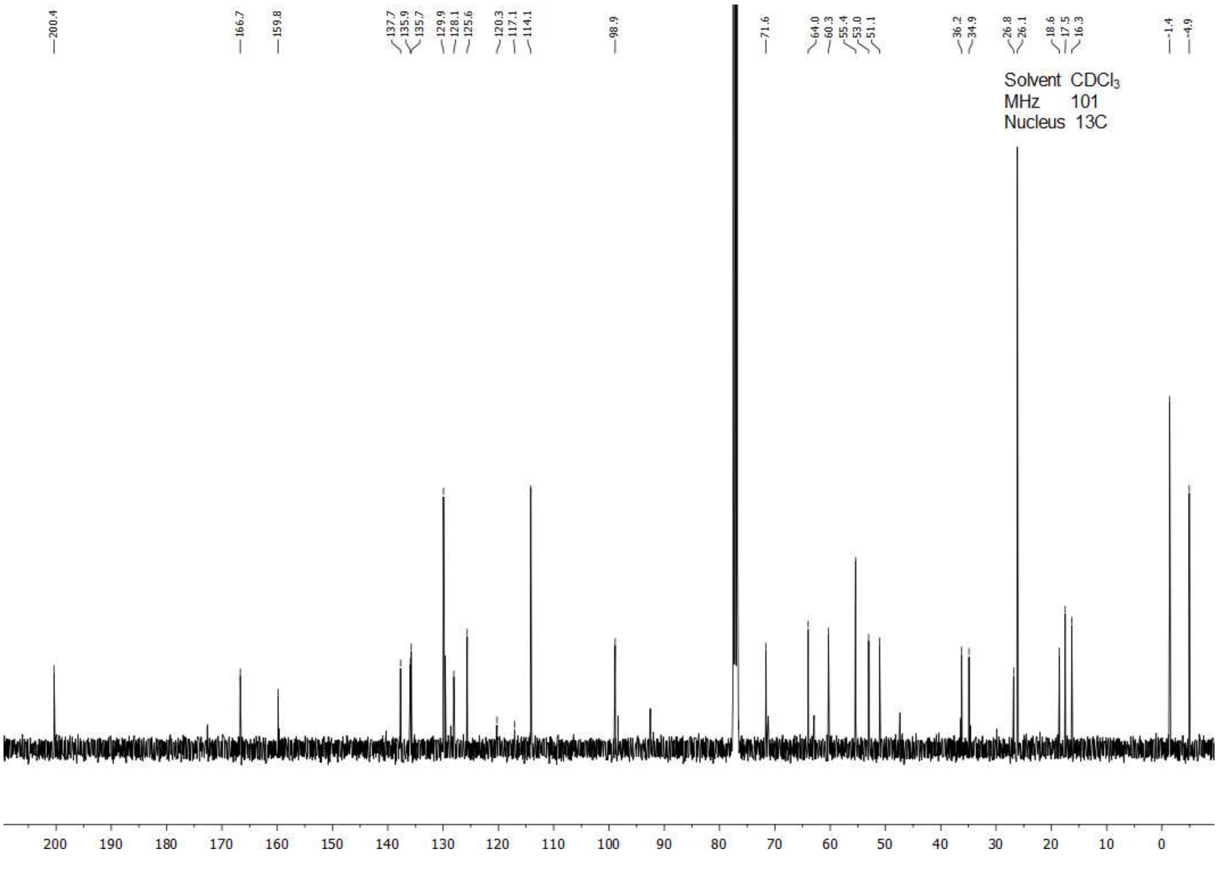
200.4
166.7
159.8
137.7
135.9
135.7
129.9
128.1
125.6
120.3
117.1
114.1
98.9
71.6
64.0
60.3
55.4
53.0
51.1
36.2
34.9
26.8
26.1
18.6
17.5
16.3
-1.4
-4.9
Solvent CDCl3
MHz 101
Nucleus 13C

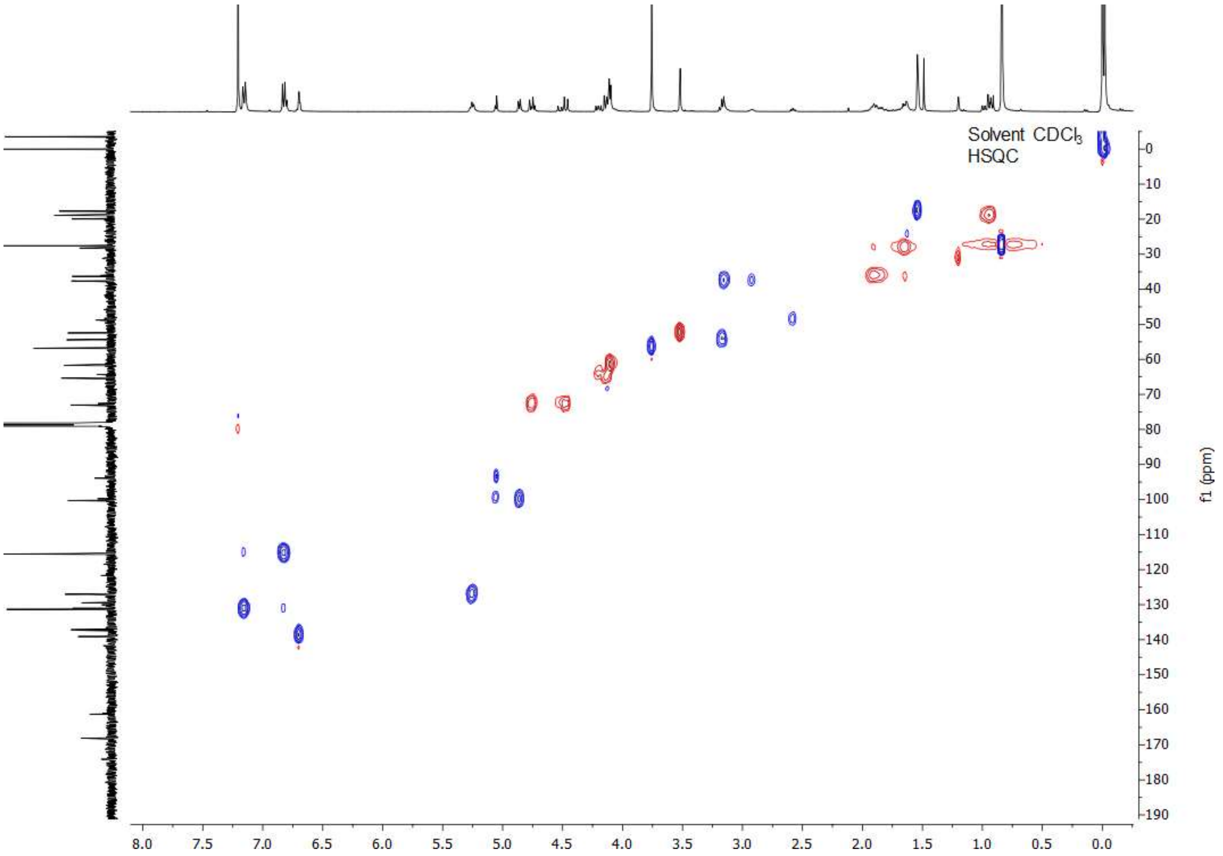
Solvent CDCl3
HSQC
f1 (ppm)

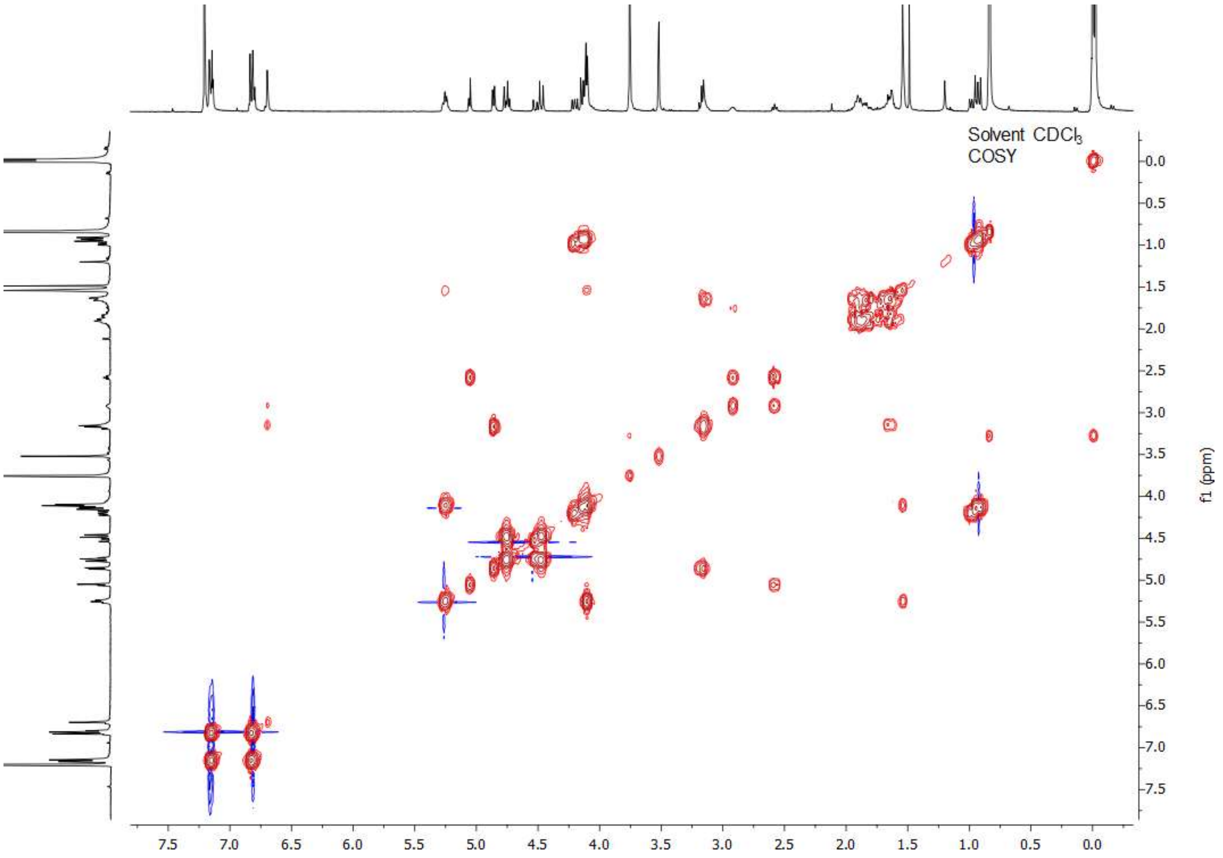
Solvent CDCl3
COSY
f1 (ppm)

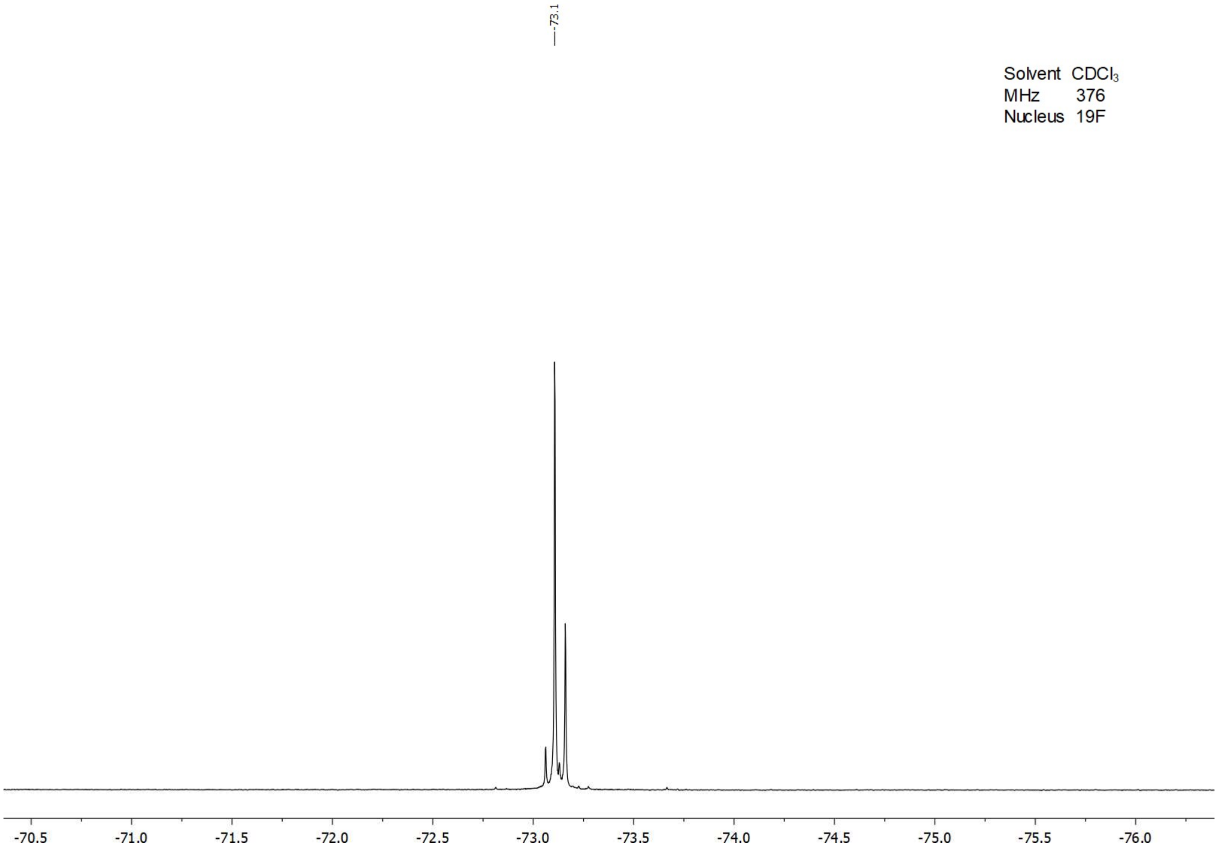

-73.1
Solvent CDCl3
MHz 376
Nucleus 19F
-70.5
-71.0
-71.5
-72.0
-72.5
-73.0
-73.5
-74.0
-74.5
-75.0
-75.5
-76.0

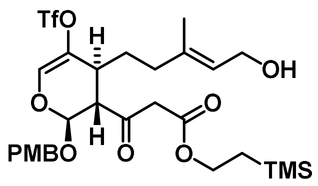
TfO
H
OH
O
O
PMBO
H
O
O
TMS

S19

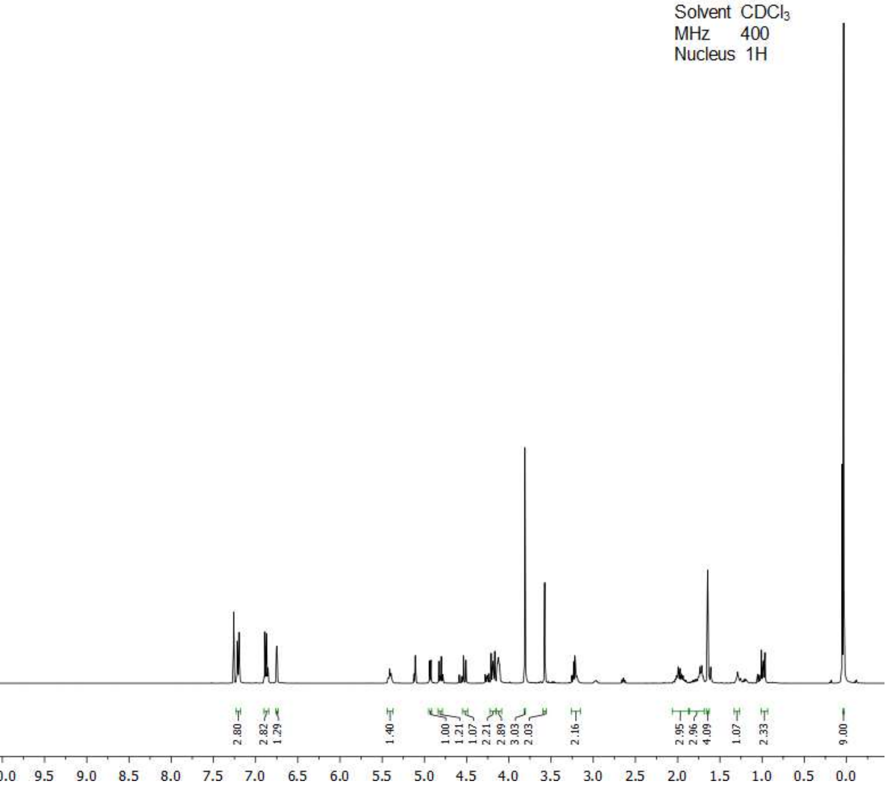
Solvent CDCl3
MHz 400
Nucleus 1H
2.80 2.82 1.29 1.40 1.00 1.21 1.07 2.21 2.89 3.03 2.03 2.16 2.95 2.96 4.09 1.07 2.33 9.00
13.5 13.0 12.5 12.0 11.5 11.0 10.5 10.0 9.5 9.0 8.5 8.0 7.5 7.0 6.5 6.0 5.5 5.0 4.5 4.0 3.5 3.0 2.5 2.0 1.5 1.0 0.5 0.0

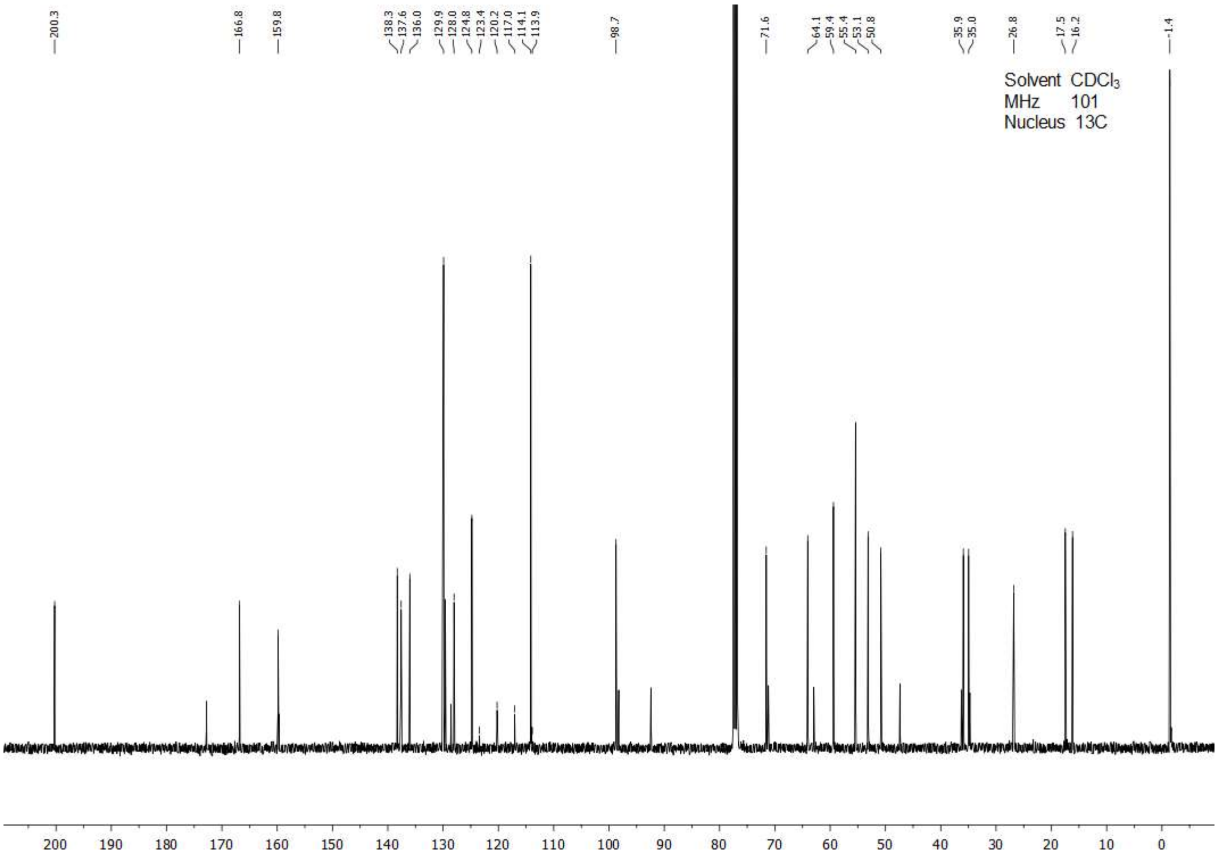
200.3
166.8
159.8
138.3 137.6 136.0 129.9 128.0 124.8 123.4 120.2 117.0 114.1 113.9
98.7
71.6
64.1 59.4 55.4 53.1 50.8
35.9 35.0
26.8
17.5 16.2
-1.4
Solvent CDCl3
MHz 101
Nucleus 13C
200 190 180 170 160 150 140 130 120 110 100 90 80 70 60 50 40 30 20 10 0

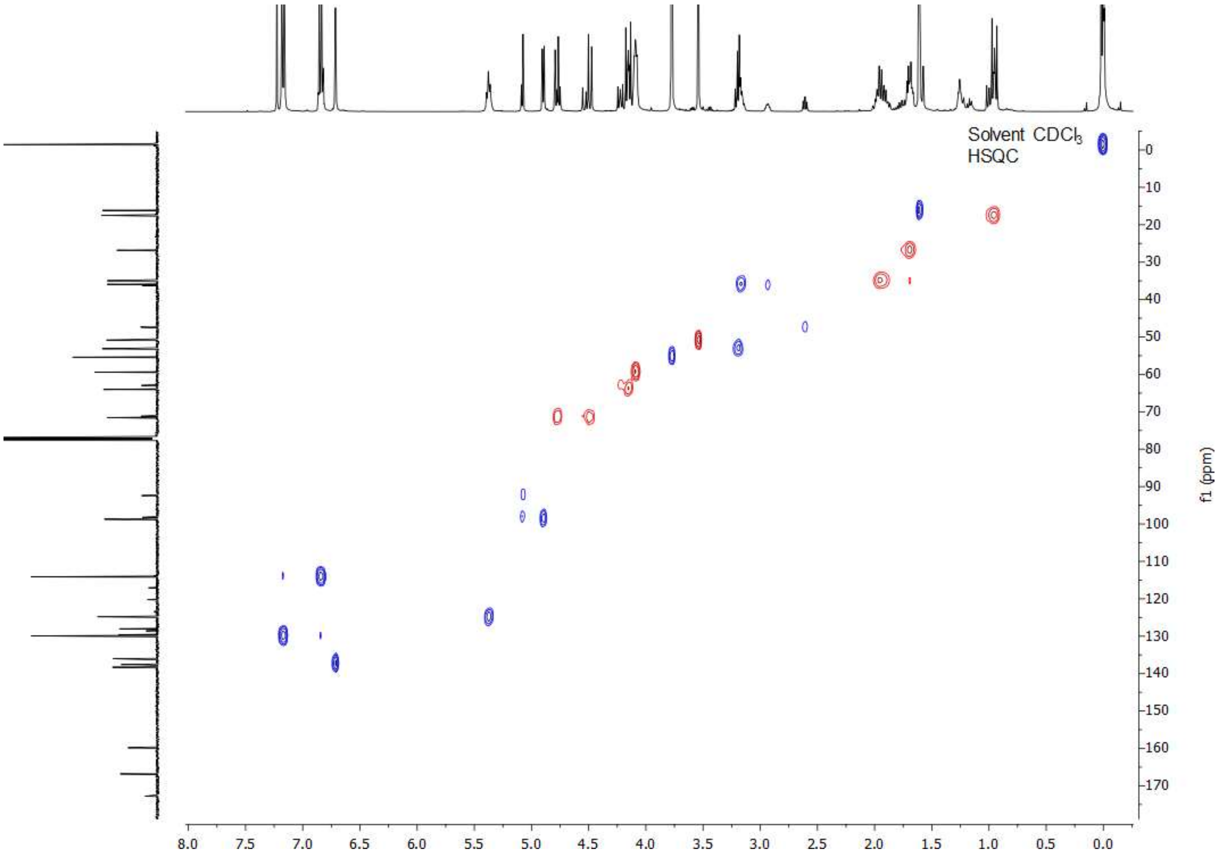

Solvent CDCl3
HSQC
f1 (ppm)

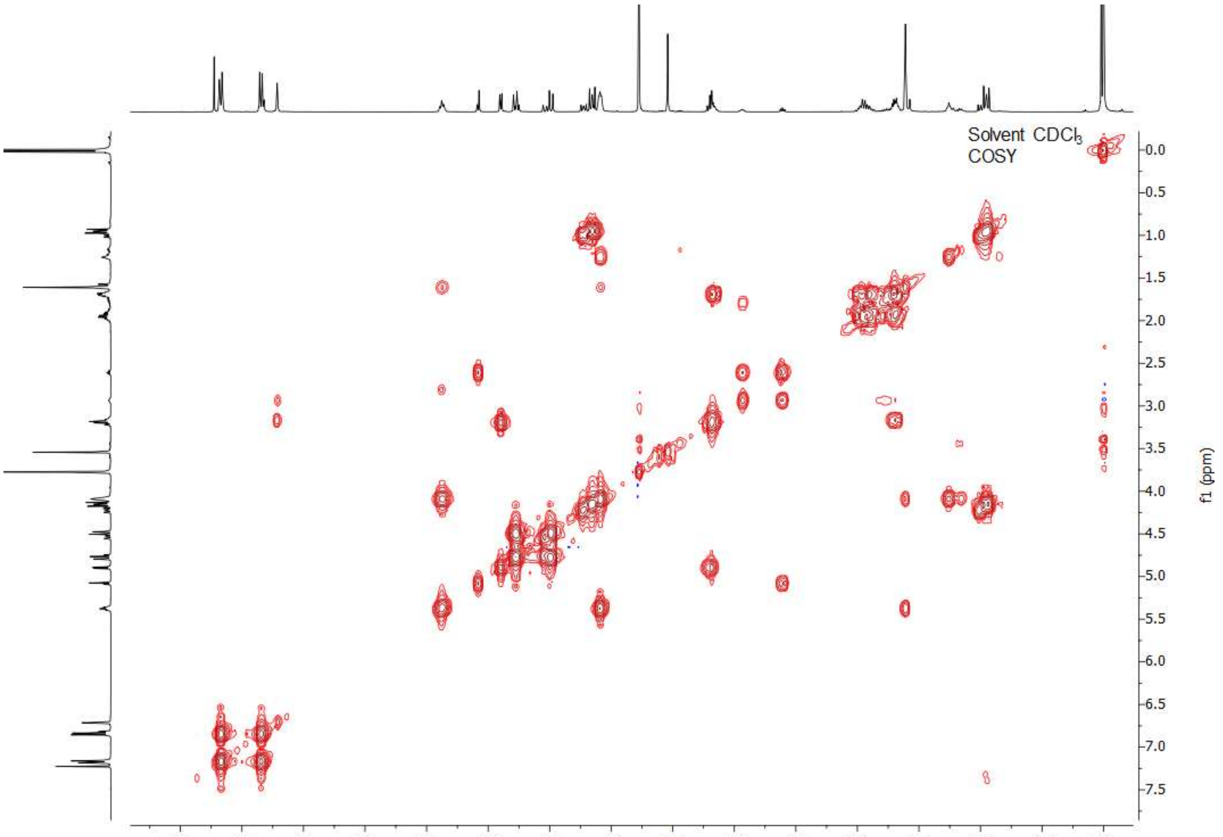

Solvent CDCl3
COSY
f1 (ppm)

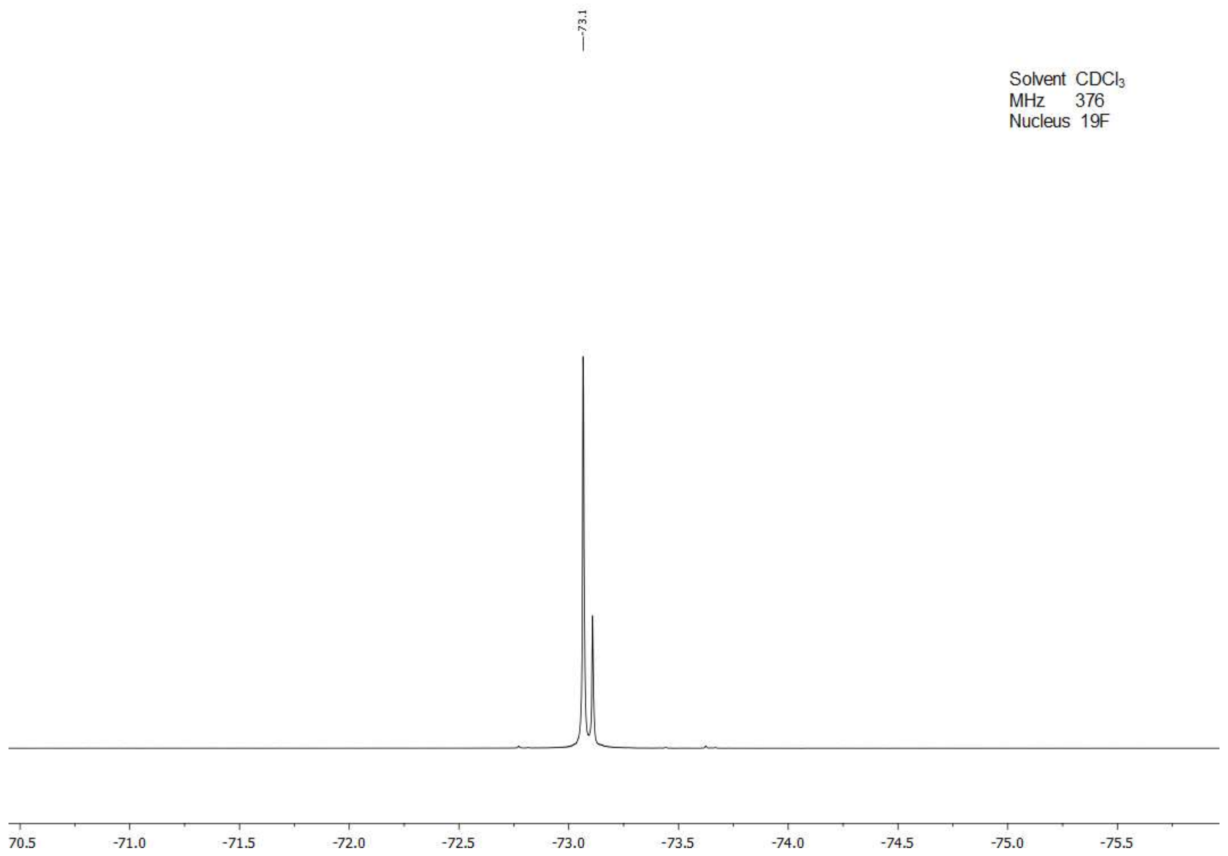

-73.1
Solvent CDCl3
MHz 376
Nucleus 19F
70.5
-71.0
-71.5
-72.0
-72.5
-73.0
-73.5
-74.0
-74.5
-75.0
-75.5

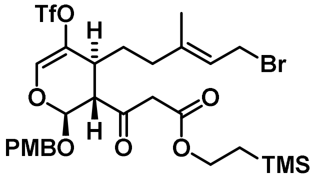
TfO
H
Br
O
O
PMBO
H
O
O
TMS

9

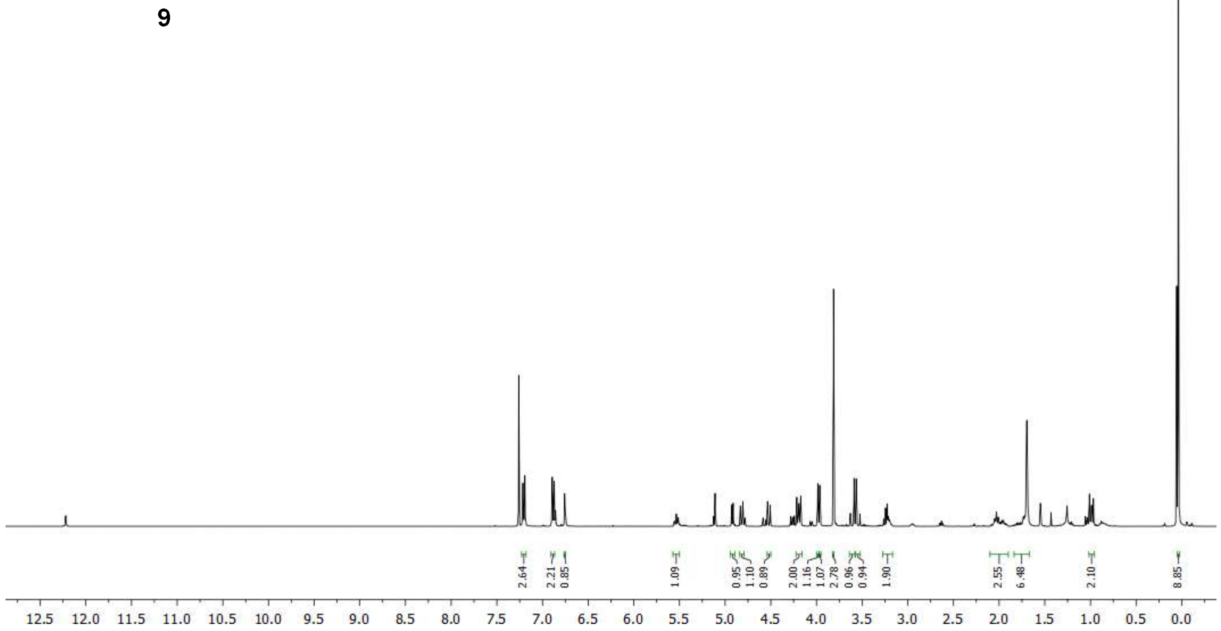
Solvent CDCl3
MHz 400
Nucleus 1H
2.64
2.21
0.85
1.09
0.95
1.10
0.89
2.00
1.16
1.07
2.78
0.96
0.94
1.90
2.55
6.48
2.10
8.85
12.5 12.0 11.5 11.0 10.5 10.0 9.5 9.0 8.5 8.0 7.5 7.0 6.5 6.0 5.5 5.0 4.5 4.0 3.5 3.0 2.5 2.0 1.5 1.0 0.5 0.0

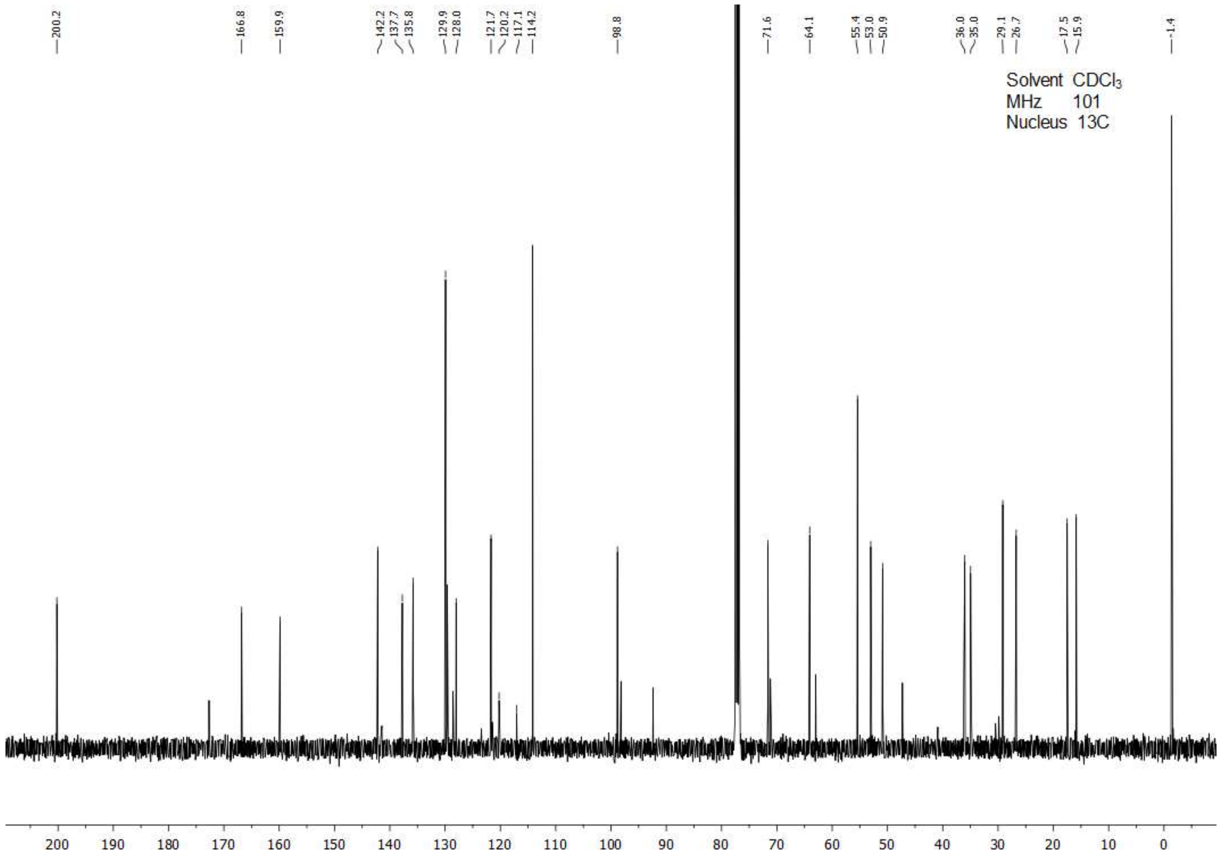
200.2
166.8
159.9
142.2
137.7
135.8
129.9
128.0
121.7
120.2
117.1
114.2
98.8
71.6
64.1
55.4
53.0
50.9
36.0
35.0
29.1
26.7
17.5
15.9
-1.4
Solvent CDCl3
MHz 101
Nucleus 13C
200 190 180 170 160 150 140 130 120 110 100 90 80 70 60 50 40 30 20 10 0

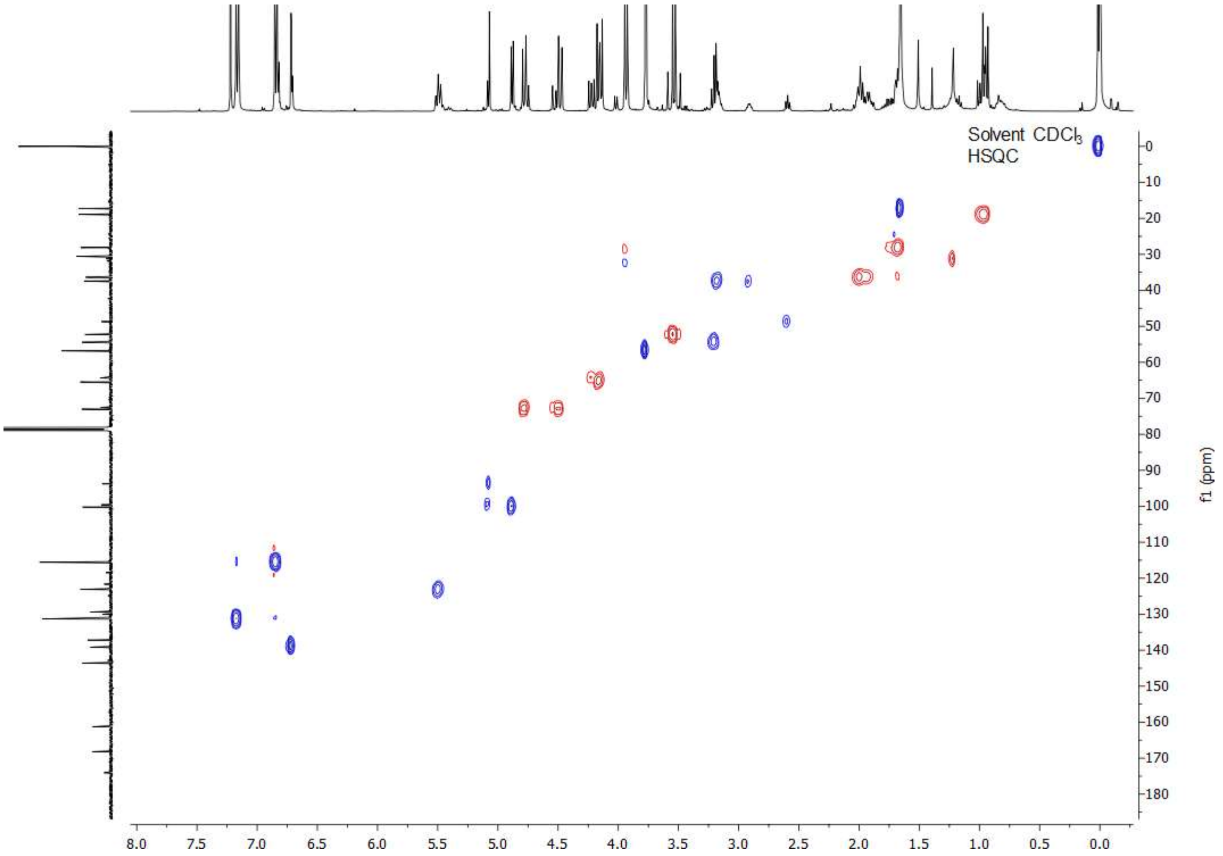
Solvent CDCl3
HSQC
f1 (ppm)

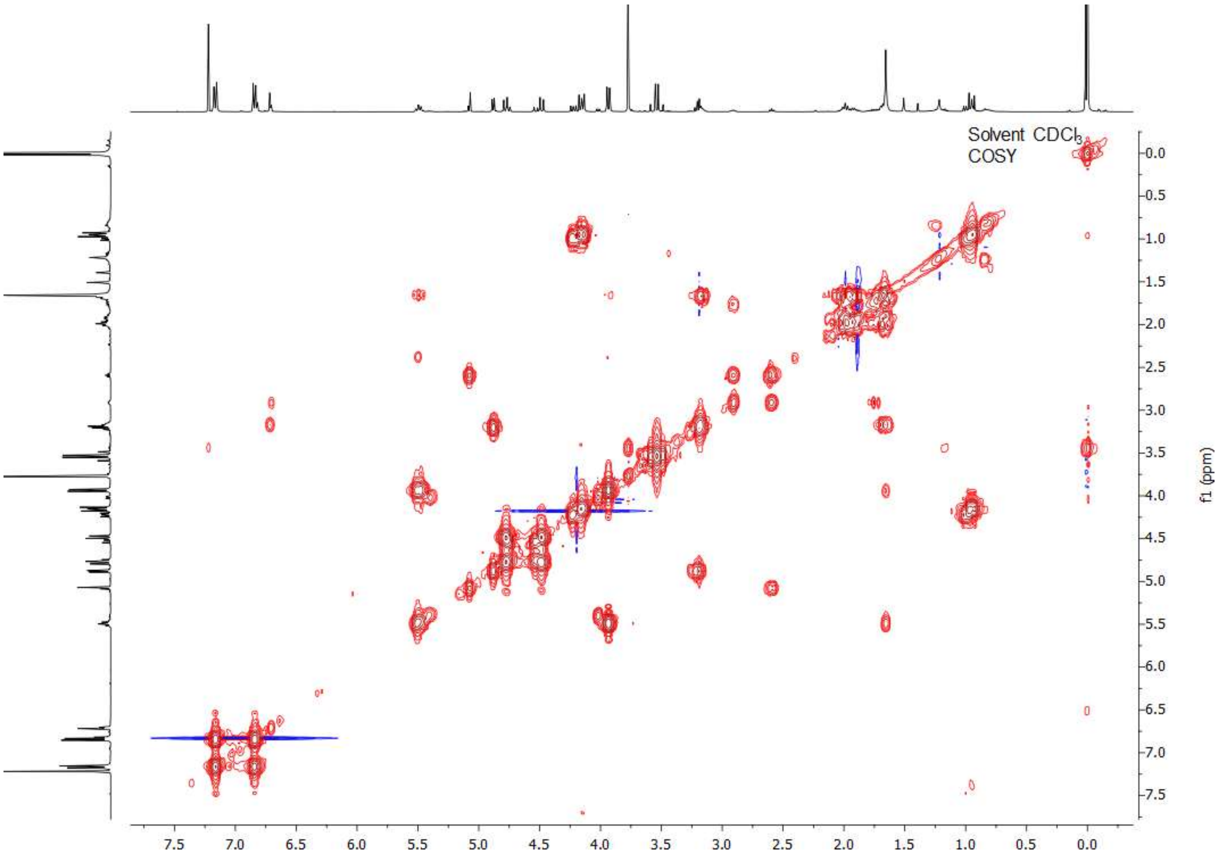
Solvent CDCl3
COSY
f1 (ppm)

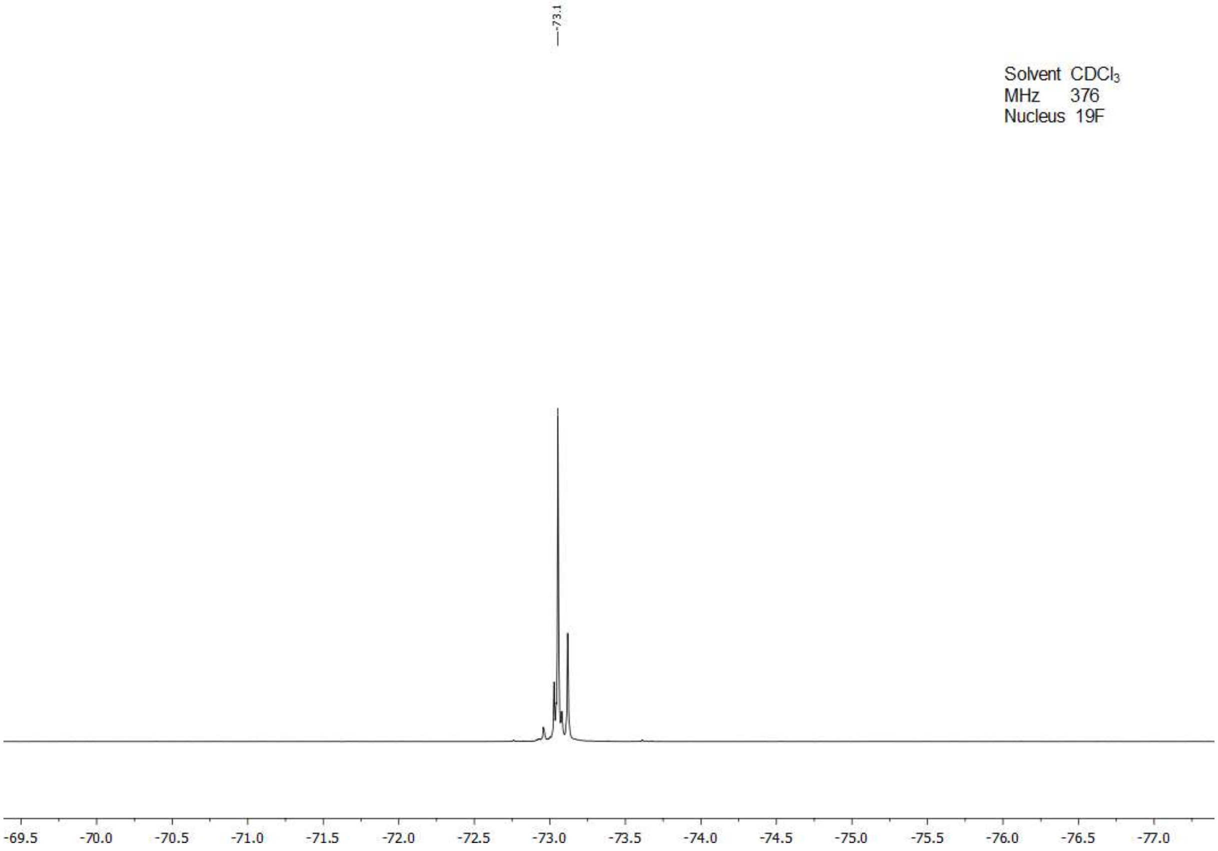

—73.1
Solvent CDCl3
MHz 376
Nucleus 19F
-69.5 -70.0 -70.5 -71.0 -71.5 -72.0 -72.5 -73.0 -73.5 -74.0 -74.5 -75.0 -75.5 -76.0 -76.5 -77.0

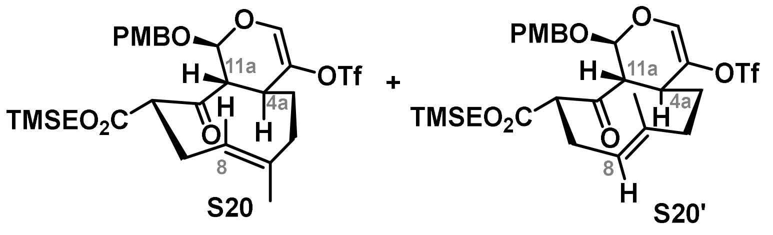
PMBO
O
OTf
H
11a
4a
TMSEO2C
8
S20
+
S20'

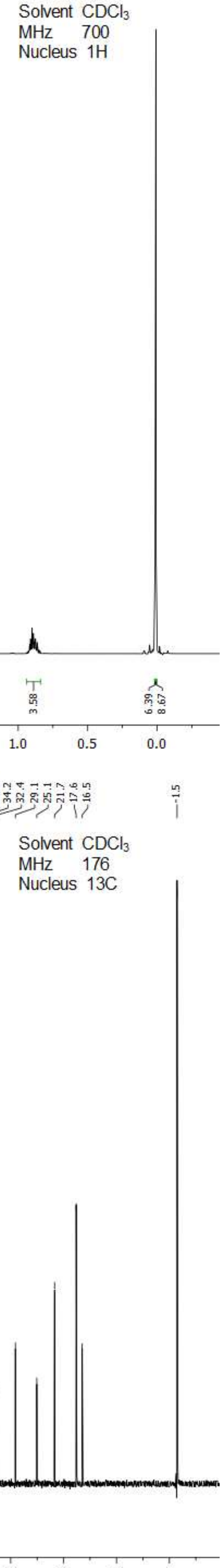
Solvent CDCl3
MHz 700
Nucleus 1H
Solvent CDCl3
MHz 176
Nucleus 13C

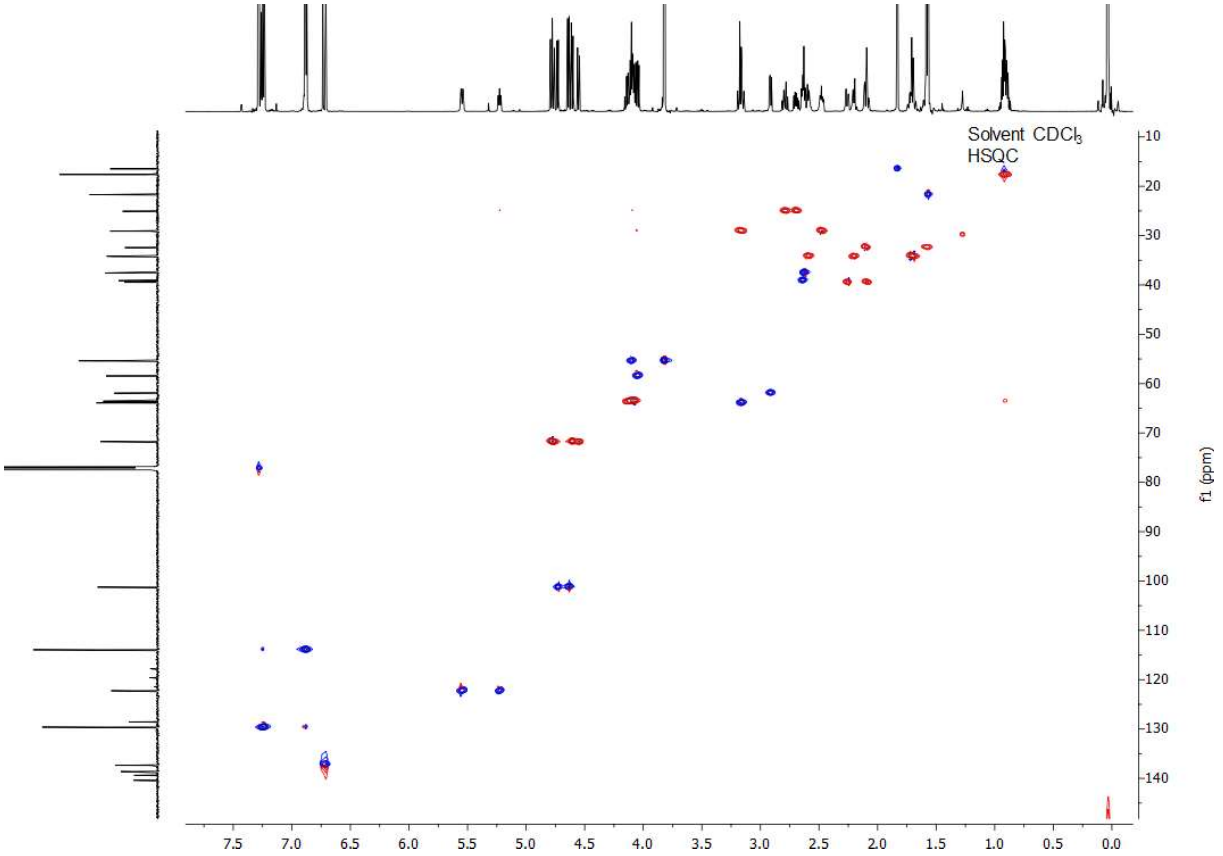
Solvent CDCl3
HSQC
f1 (ppm)

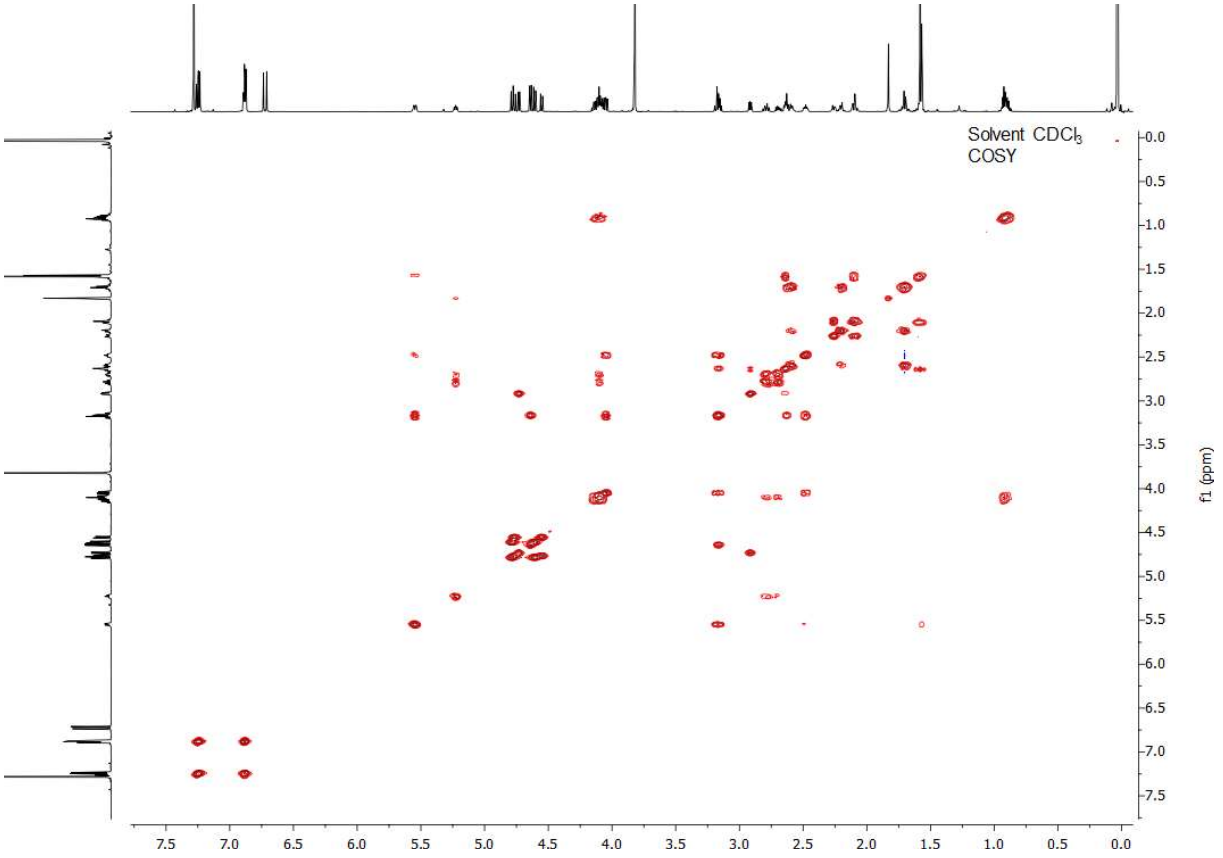
Solvent CDCl3
COSY
f1 (ppm)

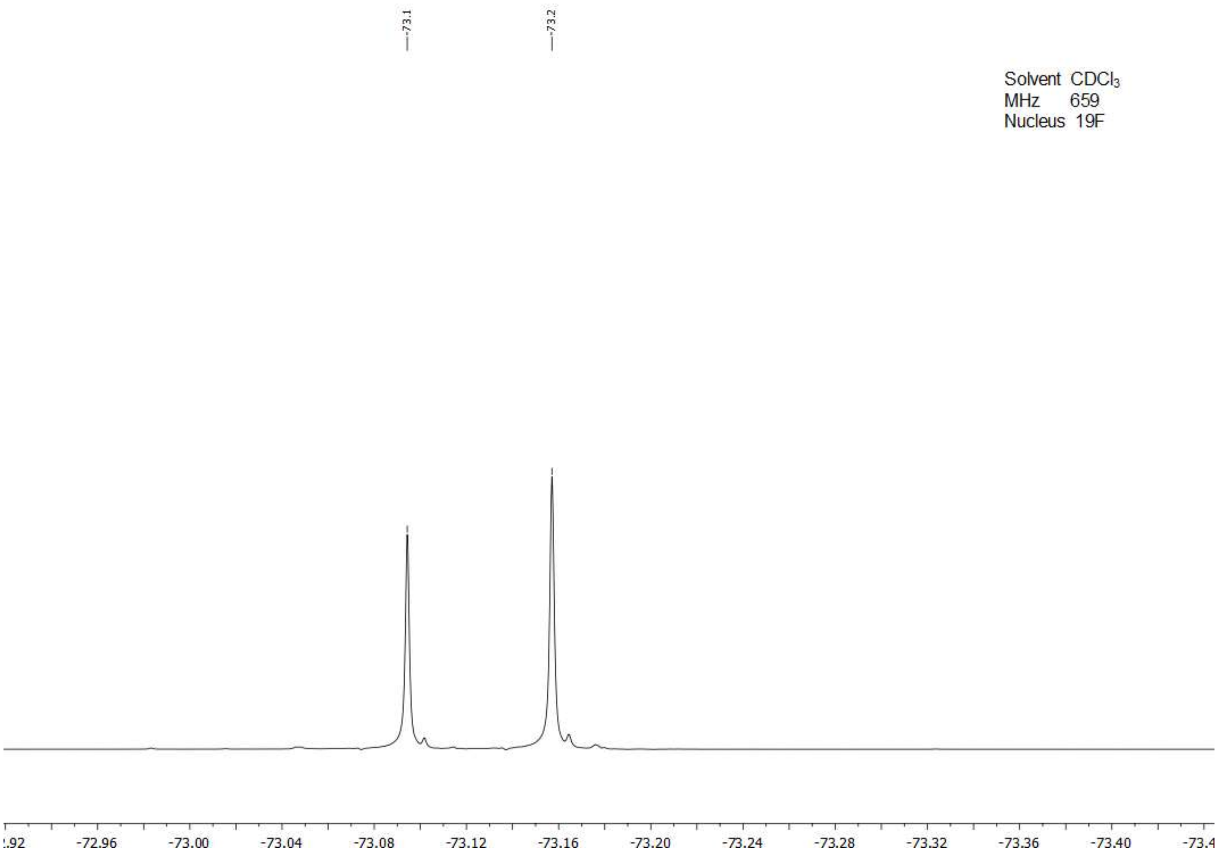
-73.1
-73.2
Solvent CDCl3
MHz 659
Nucleus 19F
-72.96
-73.00
-73.04
-73.08
-73.12
-73.16
-73.20
-73.24
-73.28
-73.32
-73.36
-73.40

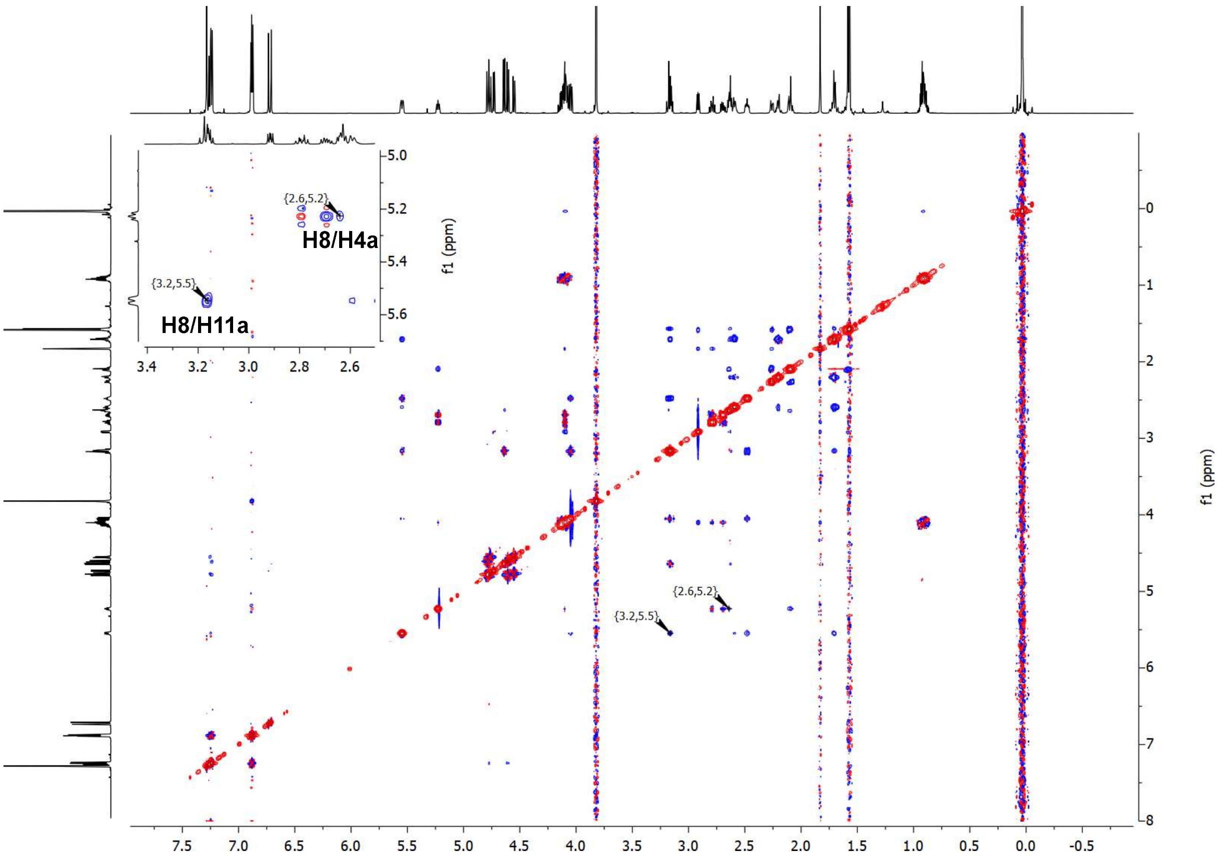
{2.6,5.2}
H8/H4a
{3.2,5.5}
H8/H11a
f1 (ppm)
f1 (ppm)

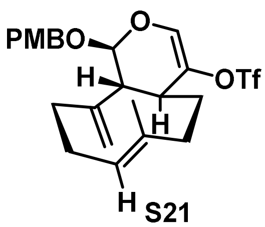

S21

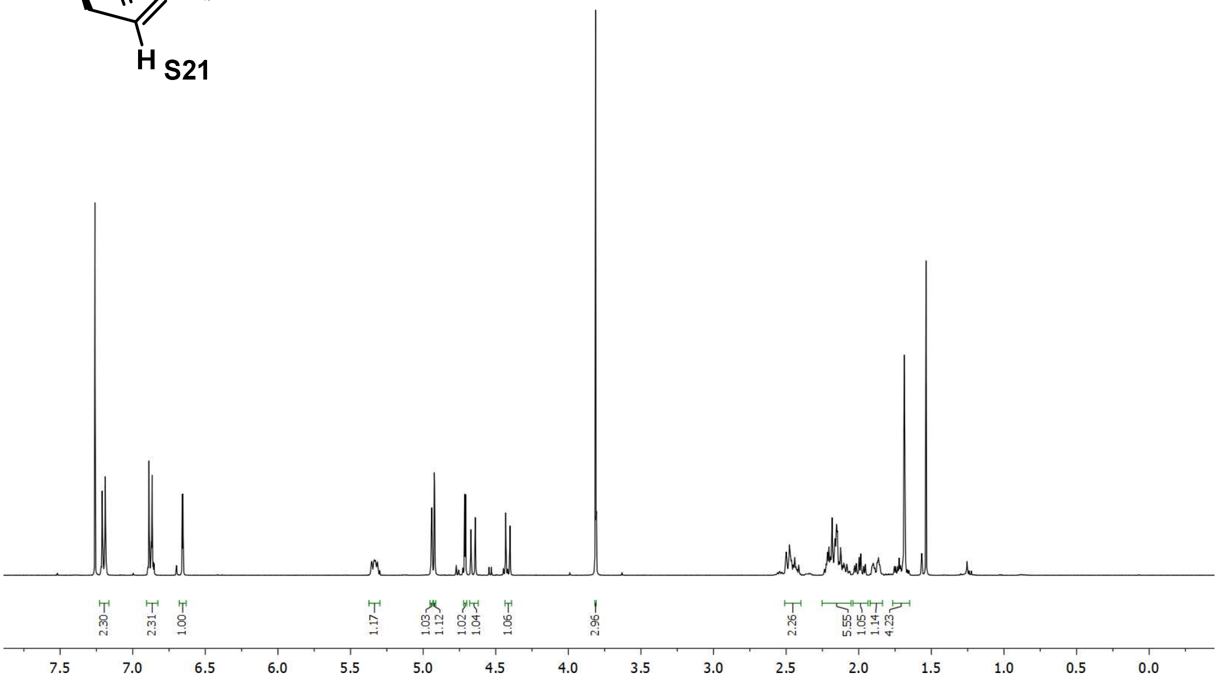
Solvent CDCl3
MHz 400
Nucleus 1H

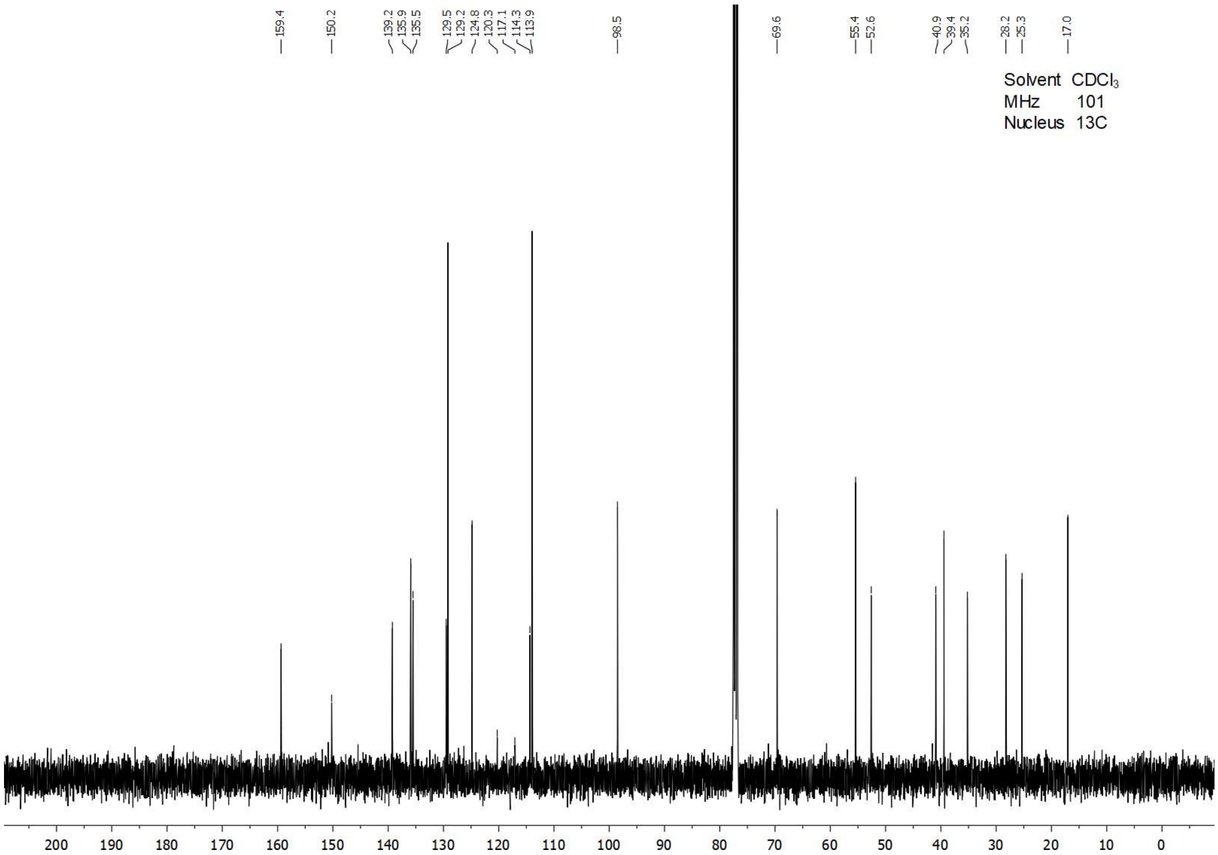
159.4
150.2
139.2
135.9
135.5
129.5
129.2
124.8
120.3
117.1
114.3
113.9
98.5
69.6
55.4
52.6
40.9
39.4
35.2
28.2
25.3
17.0
Solvent CDCl3
MHz 101
Nucleus 13C

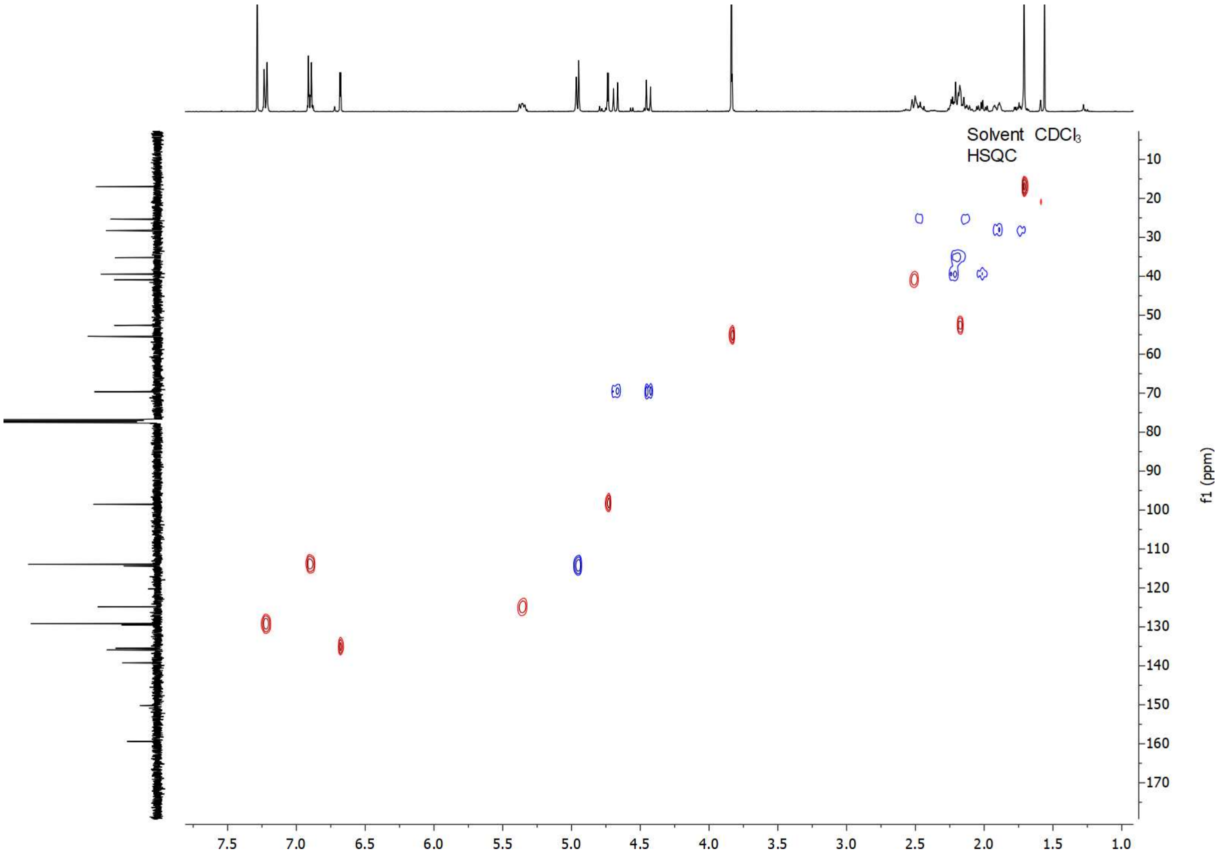
Solvent CDCl3
HSQC
f1 (ppm)

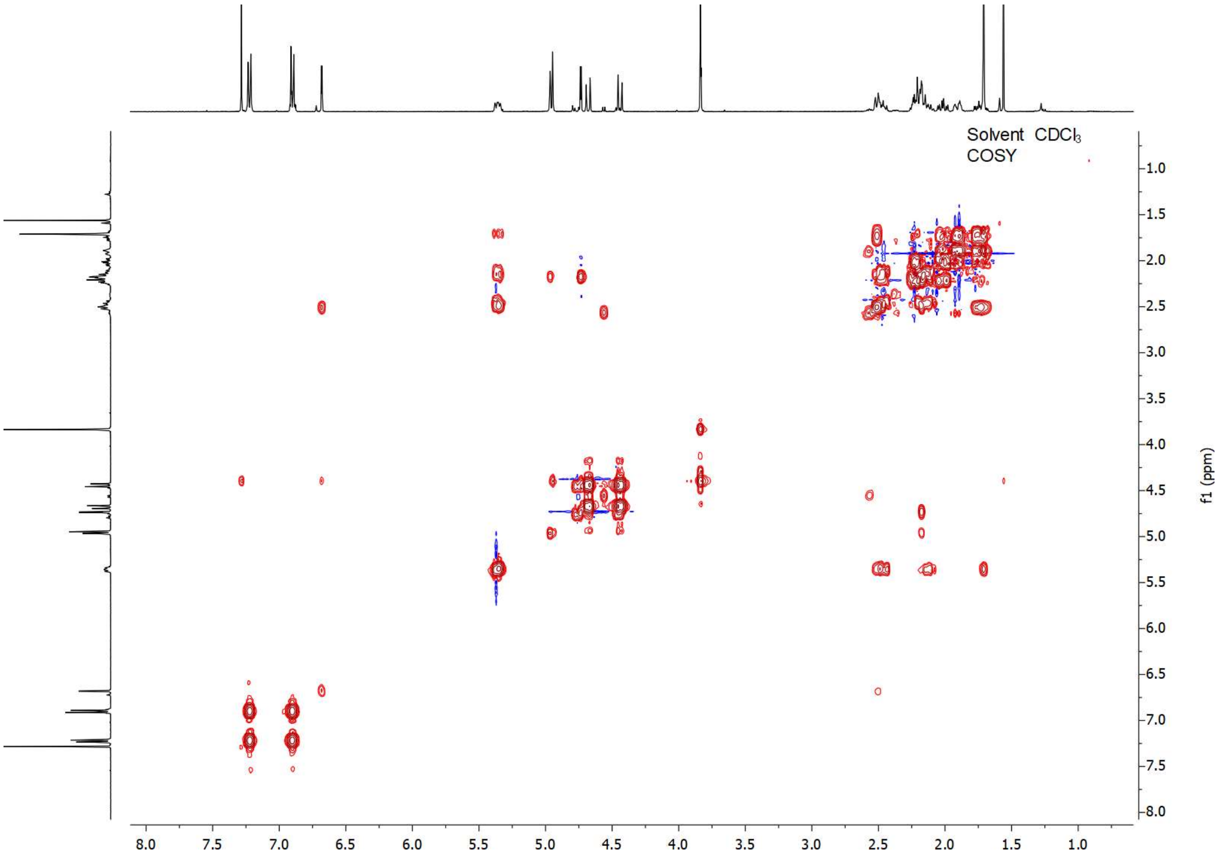
Solvent CDCl3
COSY
f1 (ppm)

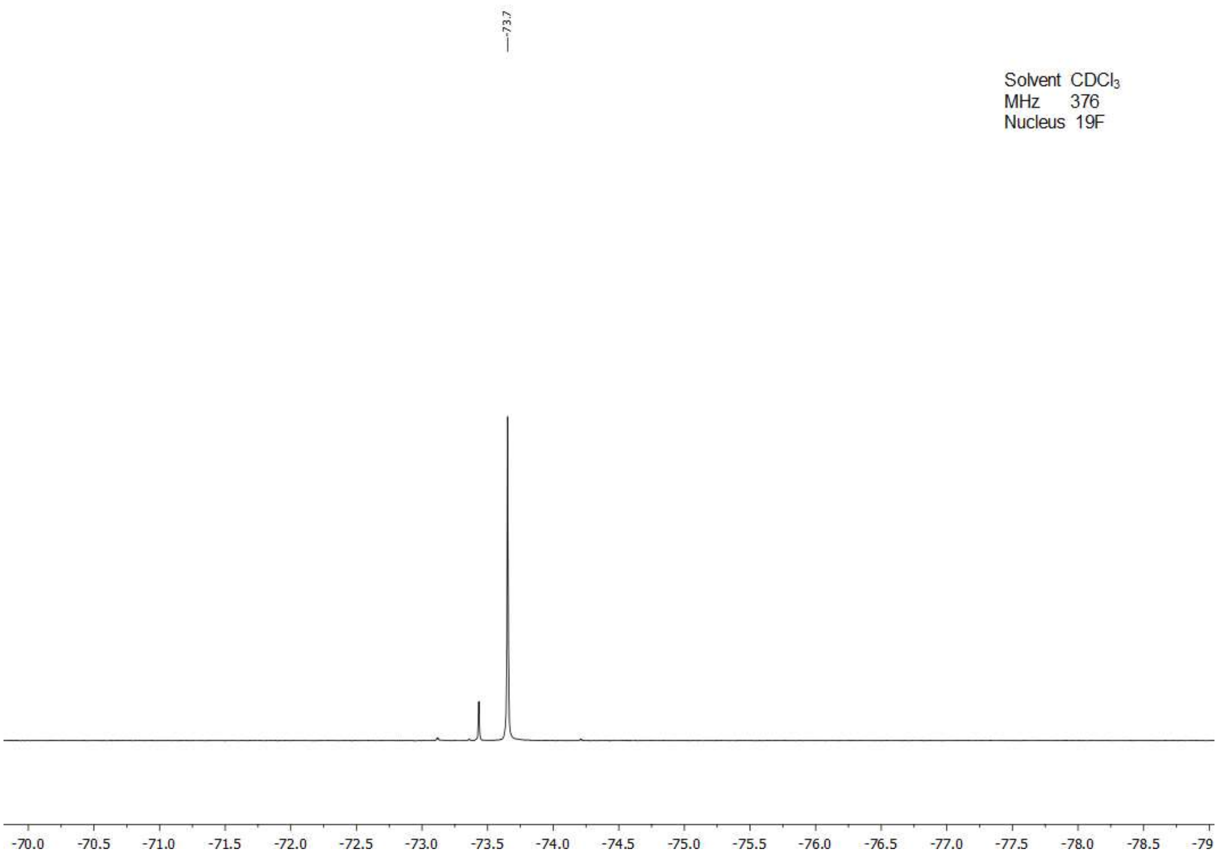
-73.7
Solvent CDCl3
MHz 376
Nucleus 19F

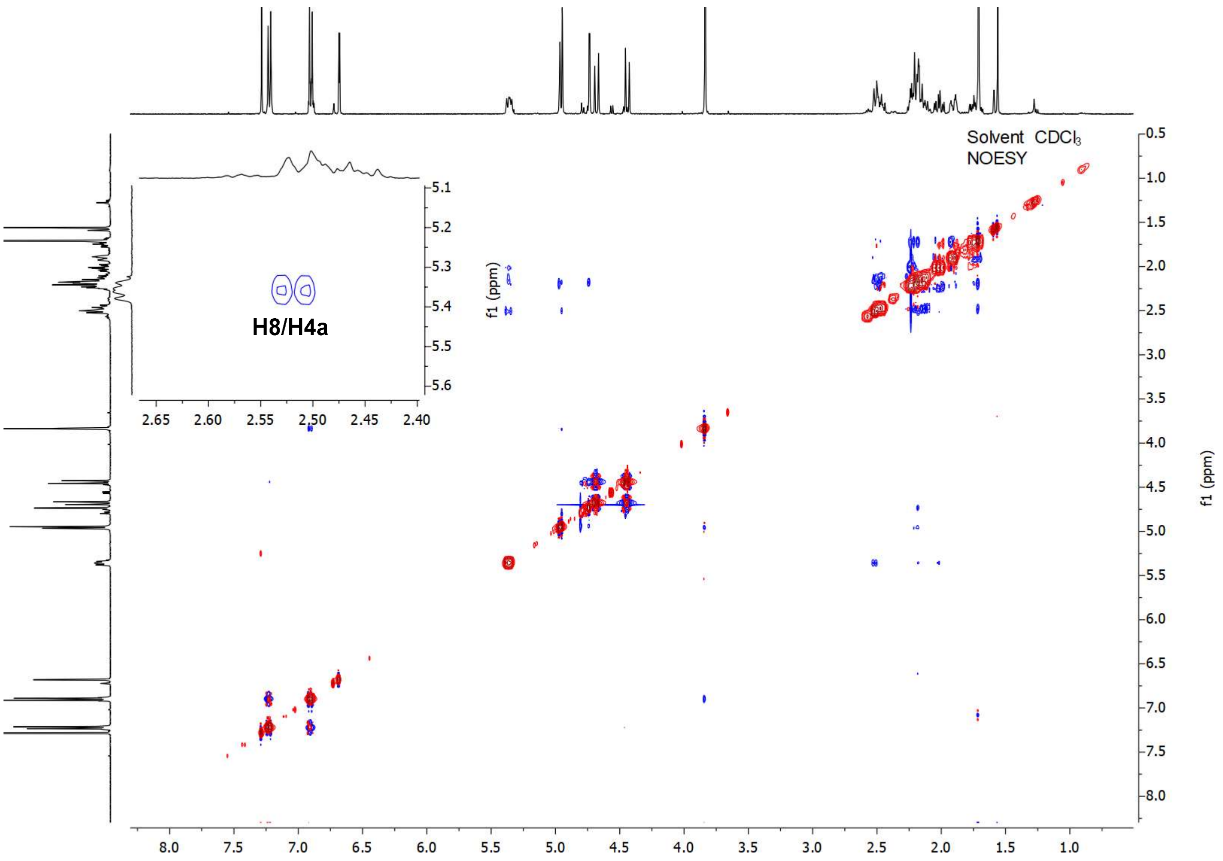
Solvent CDCl3
NOESY
H8/H4a
f1 (ppm)

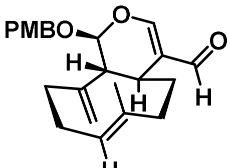
PMBO
O
H
H
H
H

25

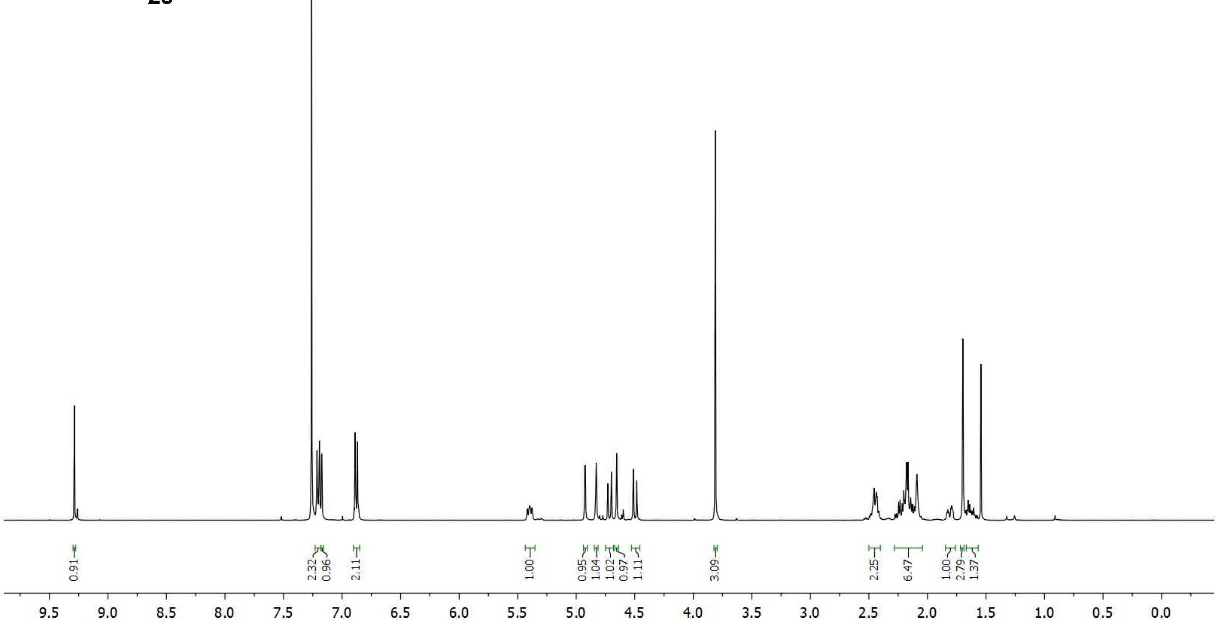
Solvent CDCl$_3$
MHz 400
Nucleus 1H
0.91
2.32
0.96
2.11
1.00
0.95
1.04
1.02
0.97
1.11
3.09
2.25
6.47
1.00
2.79
1.37
9.5 9.0 8.5 8.0 7.5 7.0 6.5 6.0 5.5 5.0 4.5 4.0 3.5 3.0 2.5 2.0 1.5 1.0 0.5 0.0

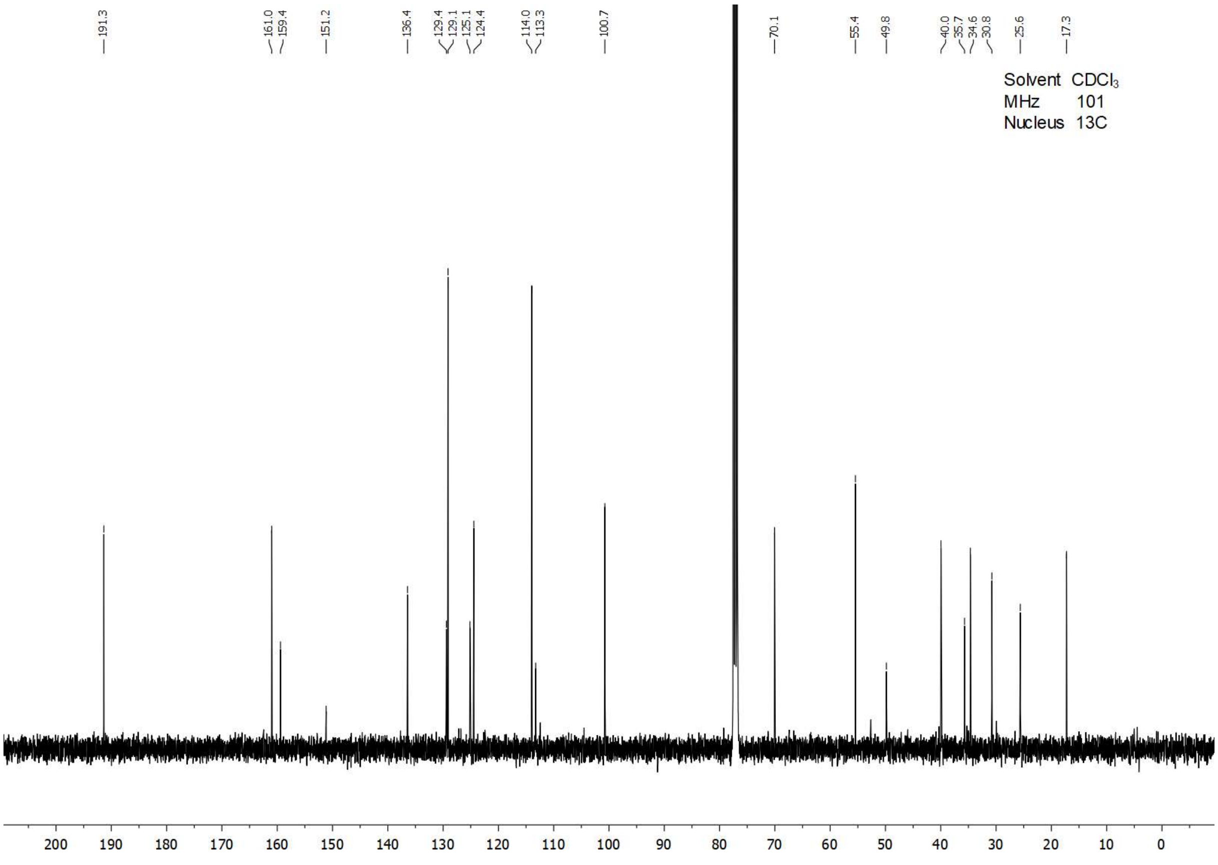
191.3
161.0
159.4
151.2
136.4
129.4
129.1
125.1
124.4
114.0
113.3
100.7
70.1
55.4
49.8
40.0
35.7
34.6
30.8
25.6
17.3
Solvent CDCl$_3$
MHz 101
Nucleus 13C
200 190 180 170 160 150 140 130 120 110 100 90 80 70 60 50 40 30 20 10 0

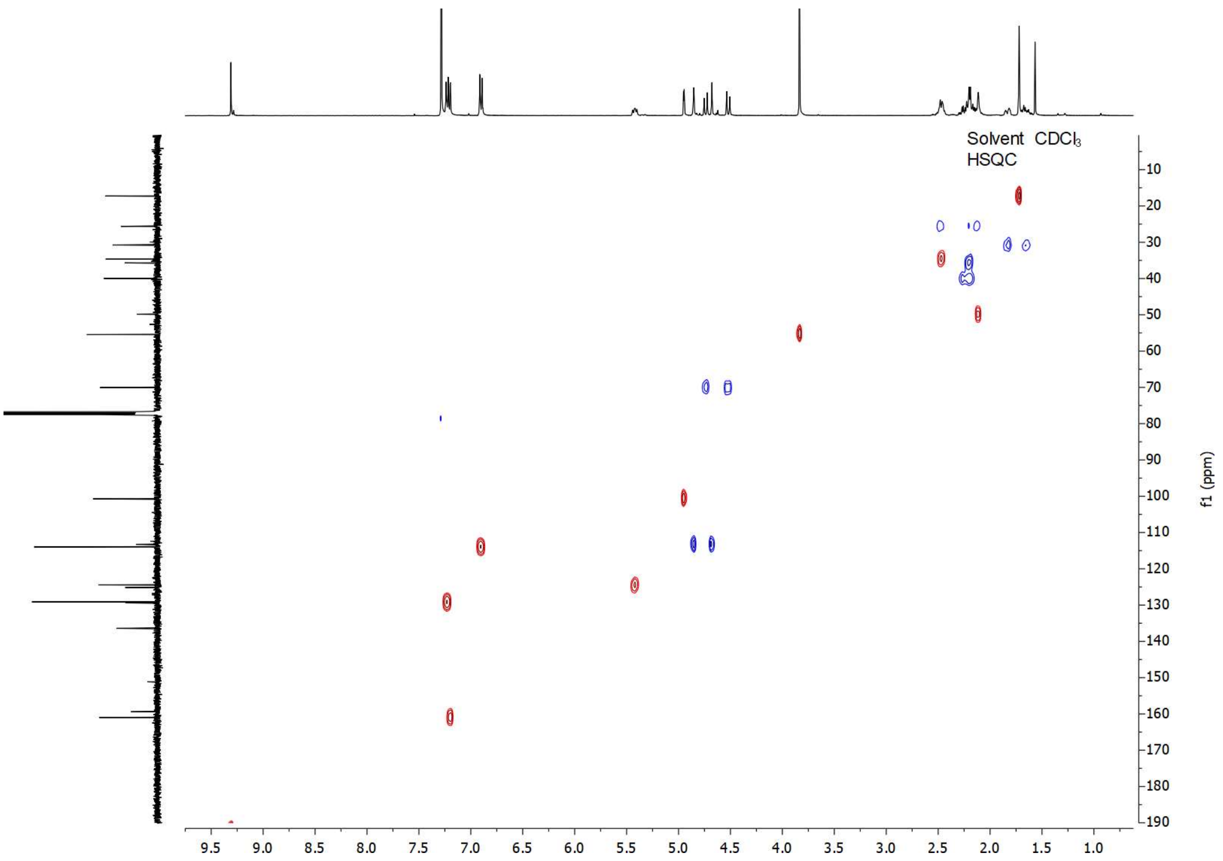

Solvent CDCl3
HSQC
f1 (ppm)

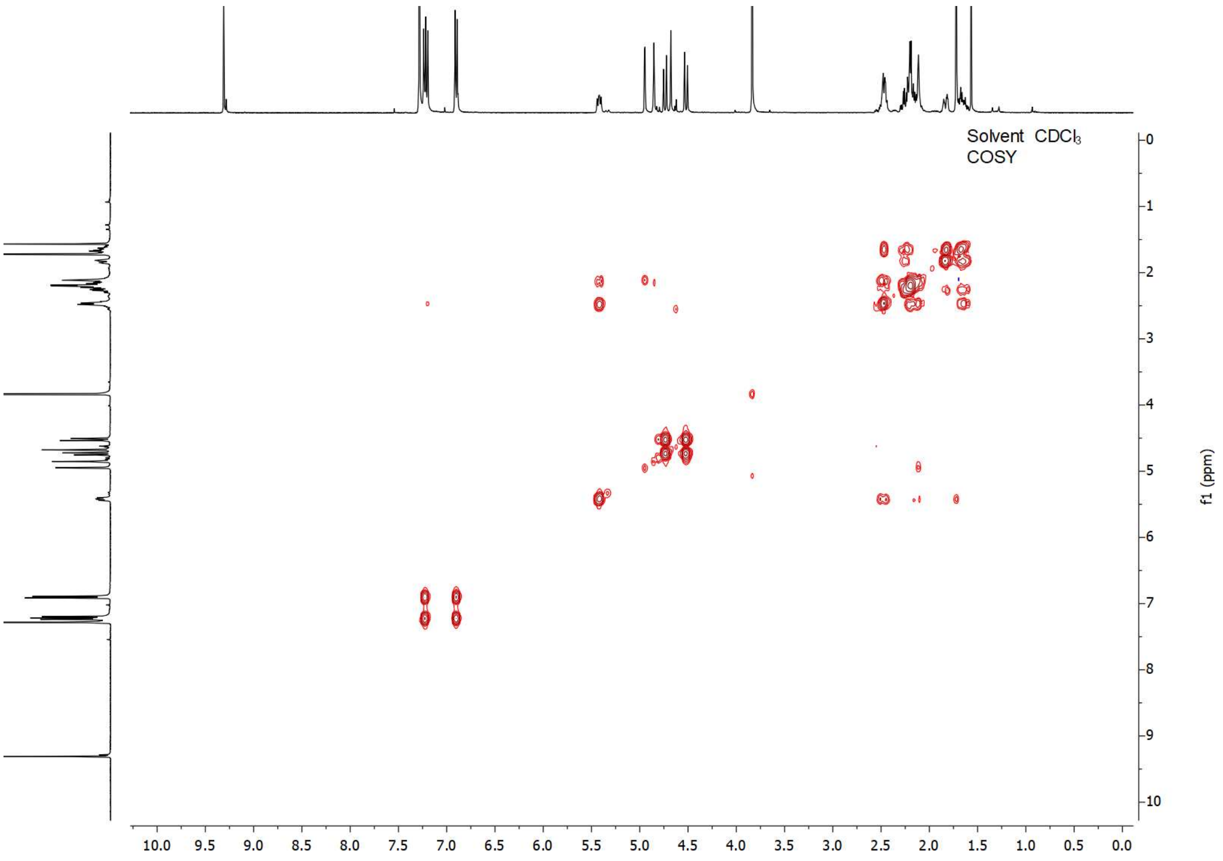

Solvent CDCl3
COSY
f1 (ppm)

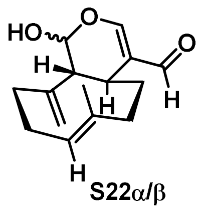

S22α/β

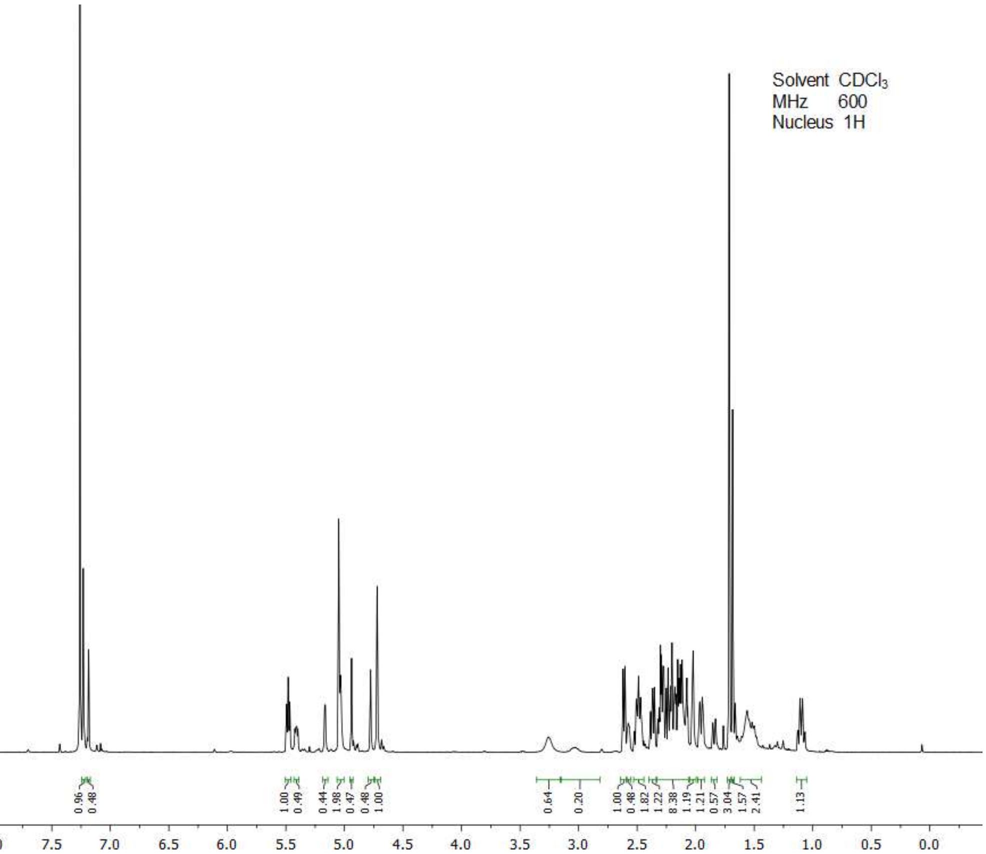
Solvent CDCl3
MHz 600
Nucleus 1H

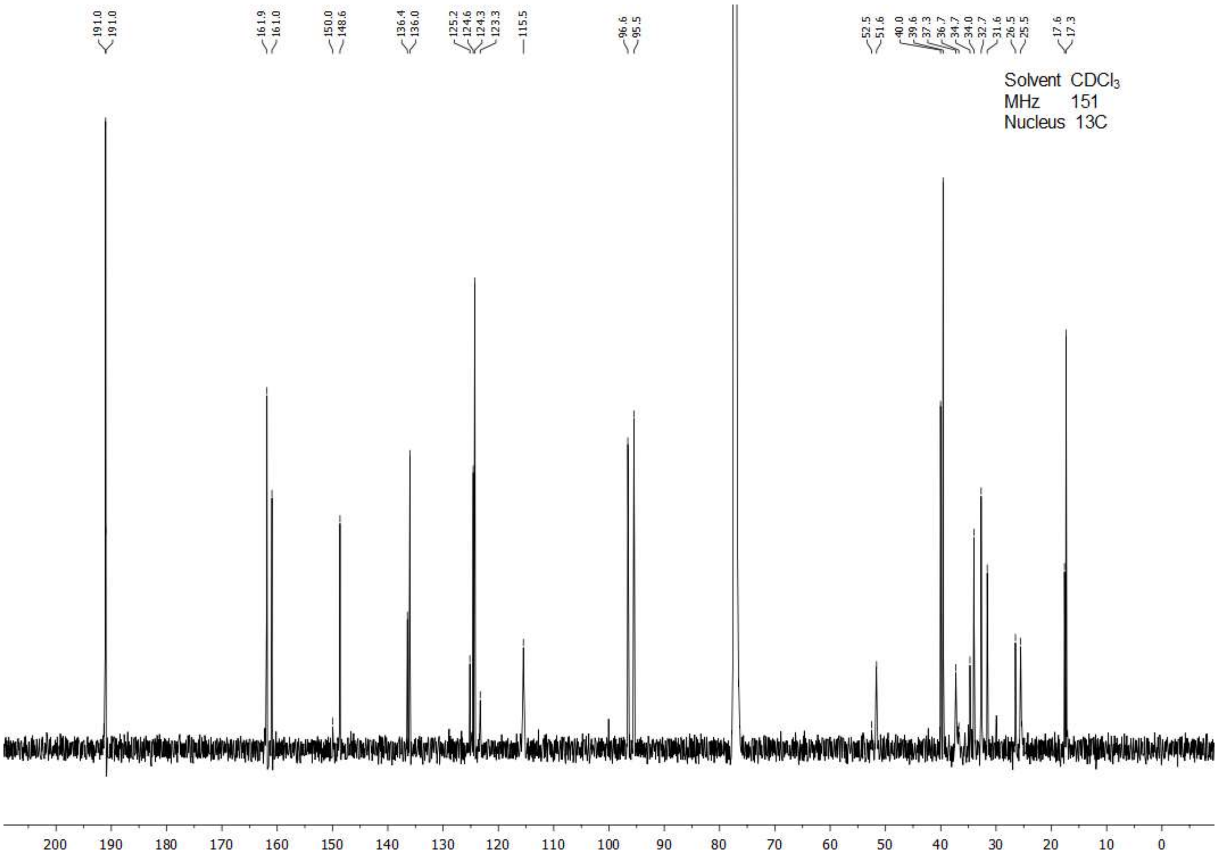
Solvent CDCl3
MHz 151
Nucleus 13C

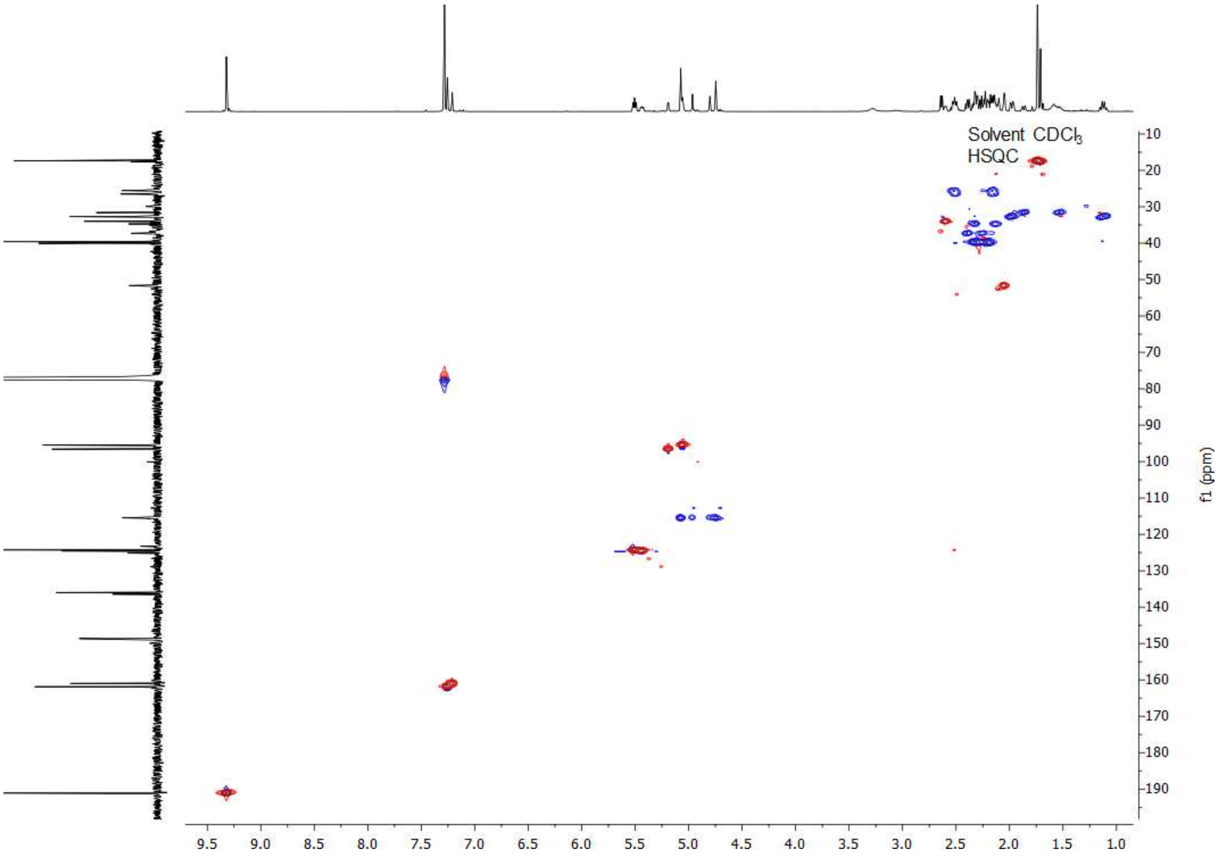
Solvent CDCl3
HSQC
f1 (ppm)

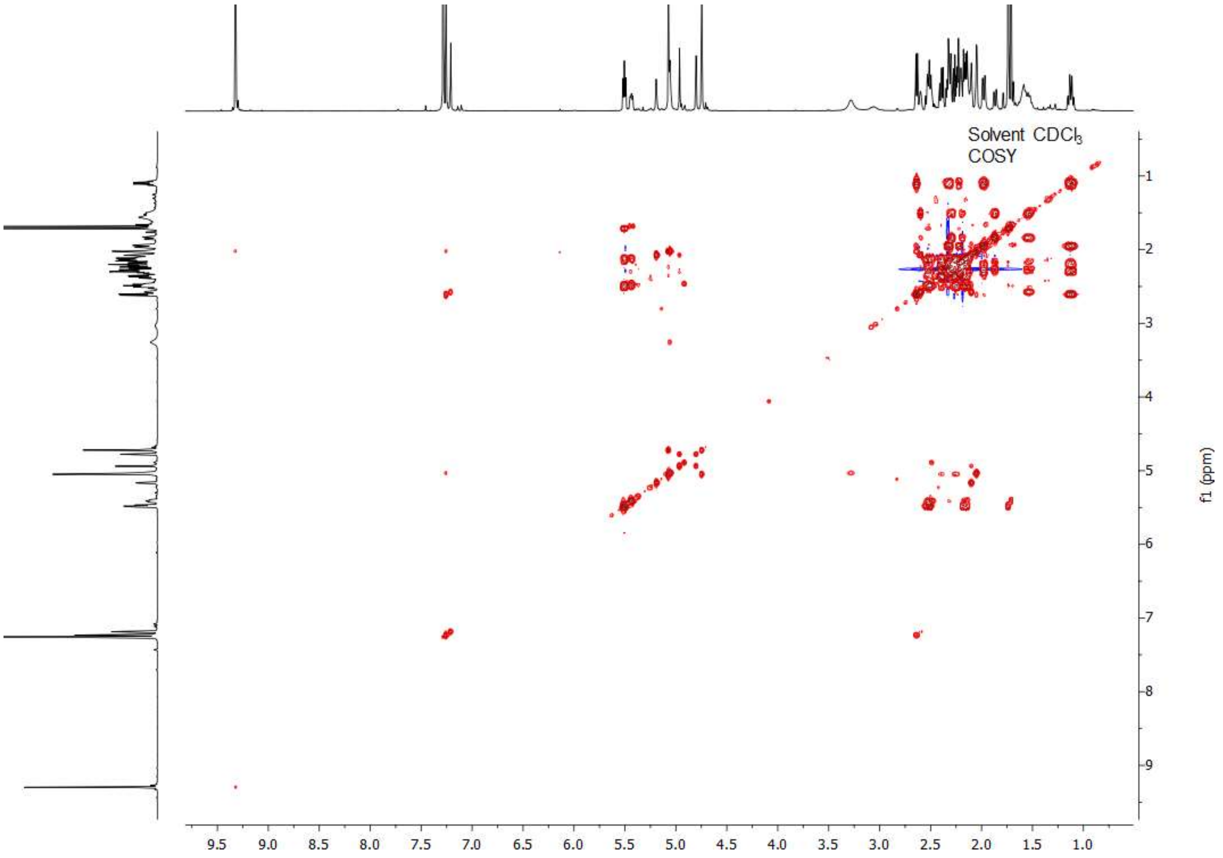
Solvent CDCl3
COSY
f1 (ppm)

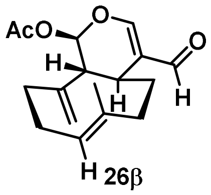

26β

Solvent CDCl$_3$
MHz 400
Nucleus 1H

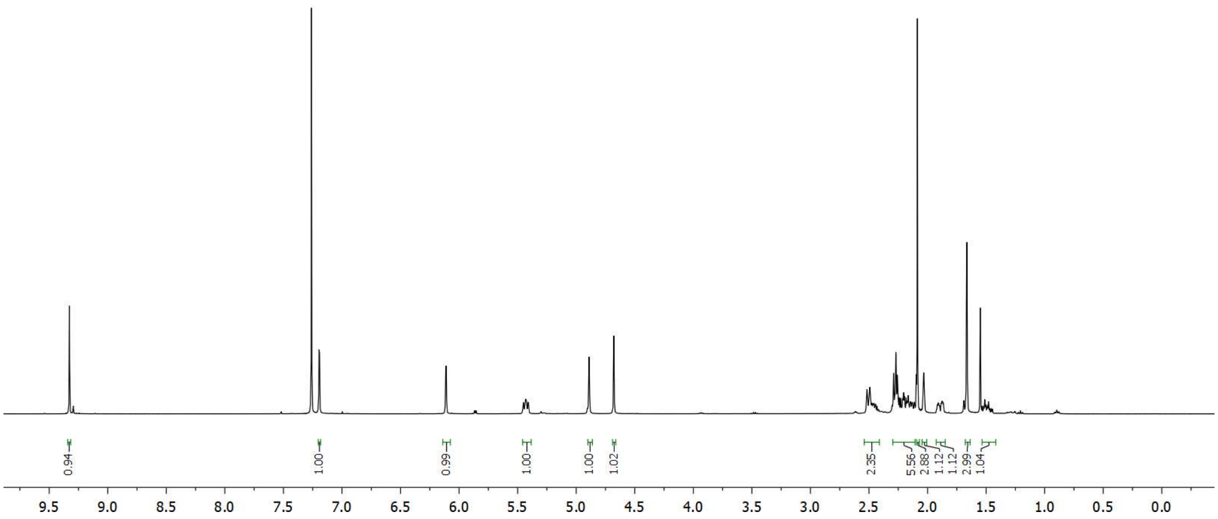

Solvent CDCl$_3$
MHz 101
Nucleus 13C

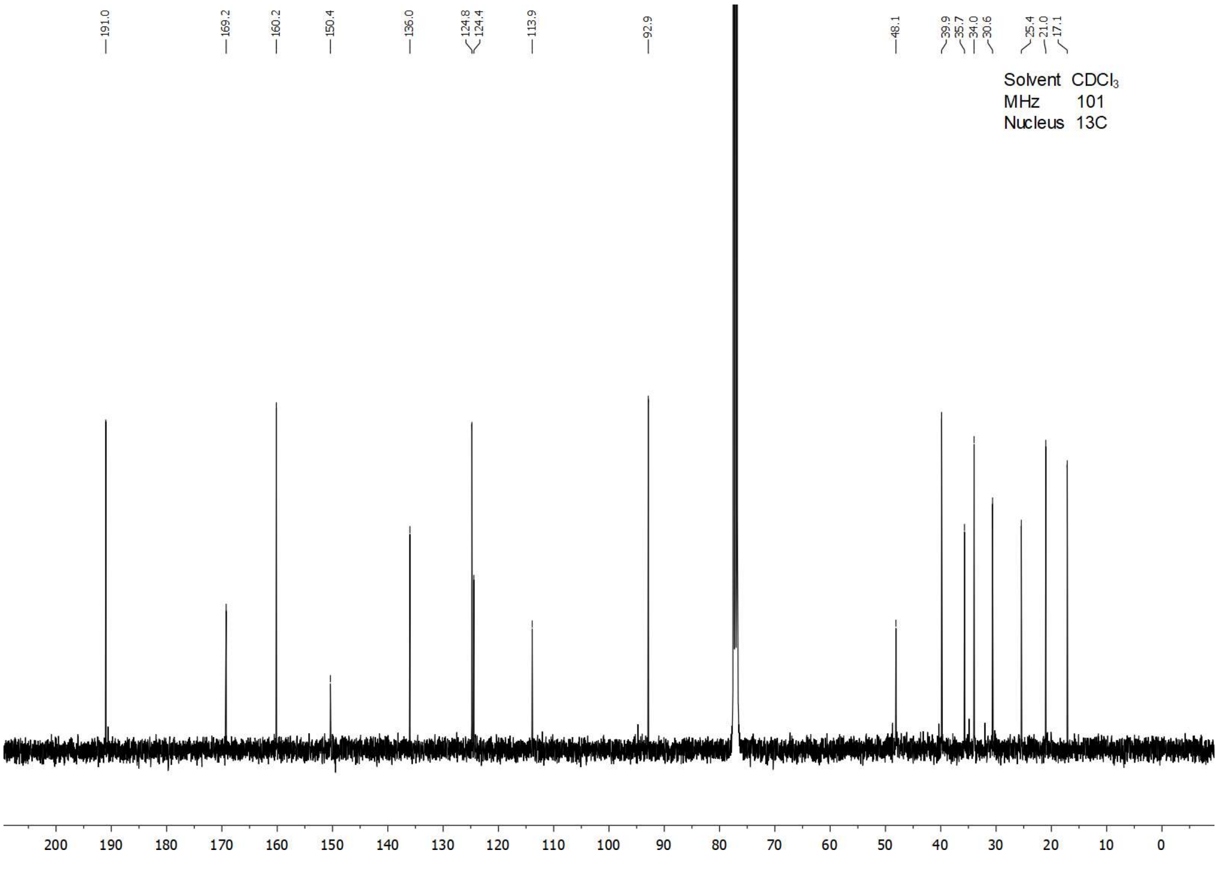

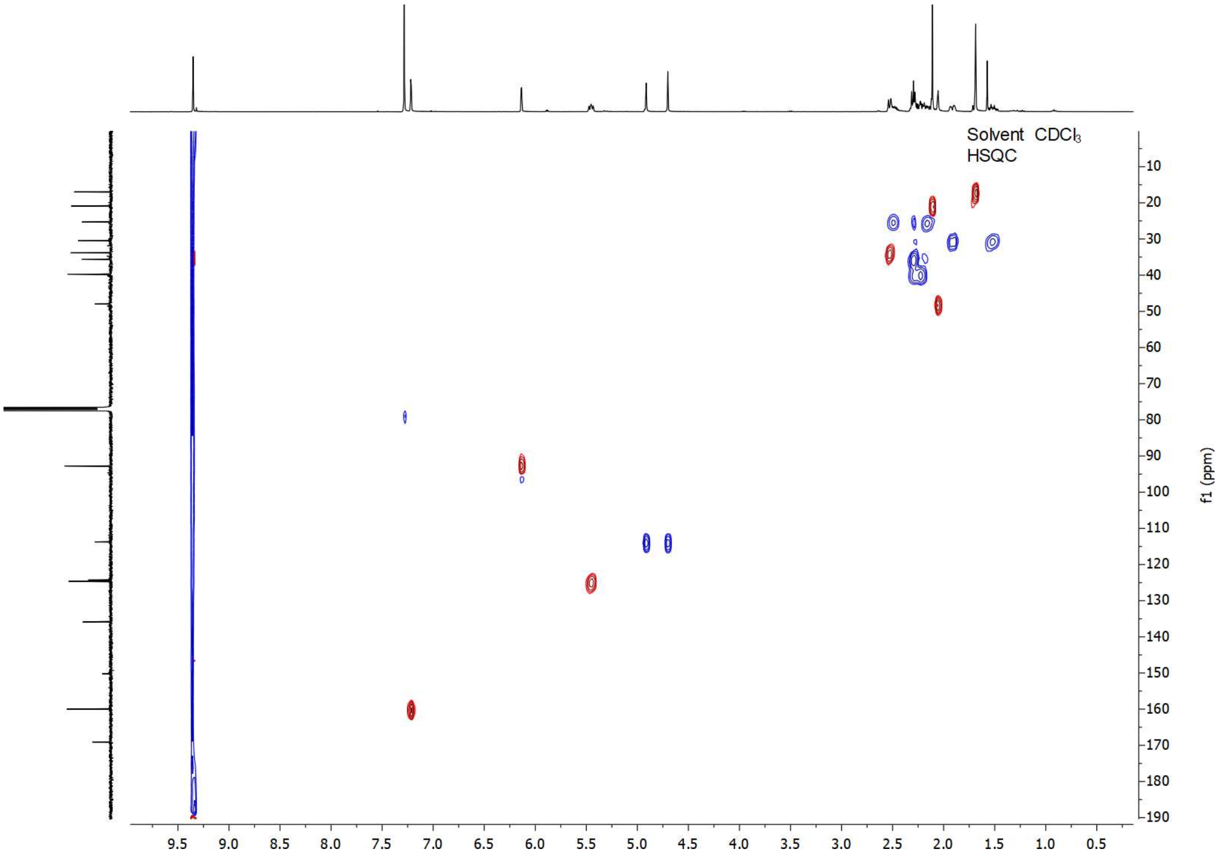
Solvent CDCl3
HSQC
f1 (ppm)

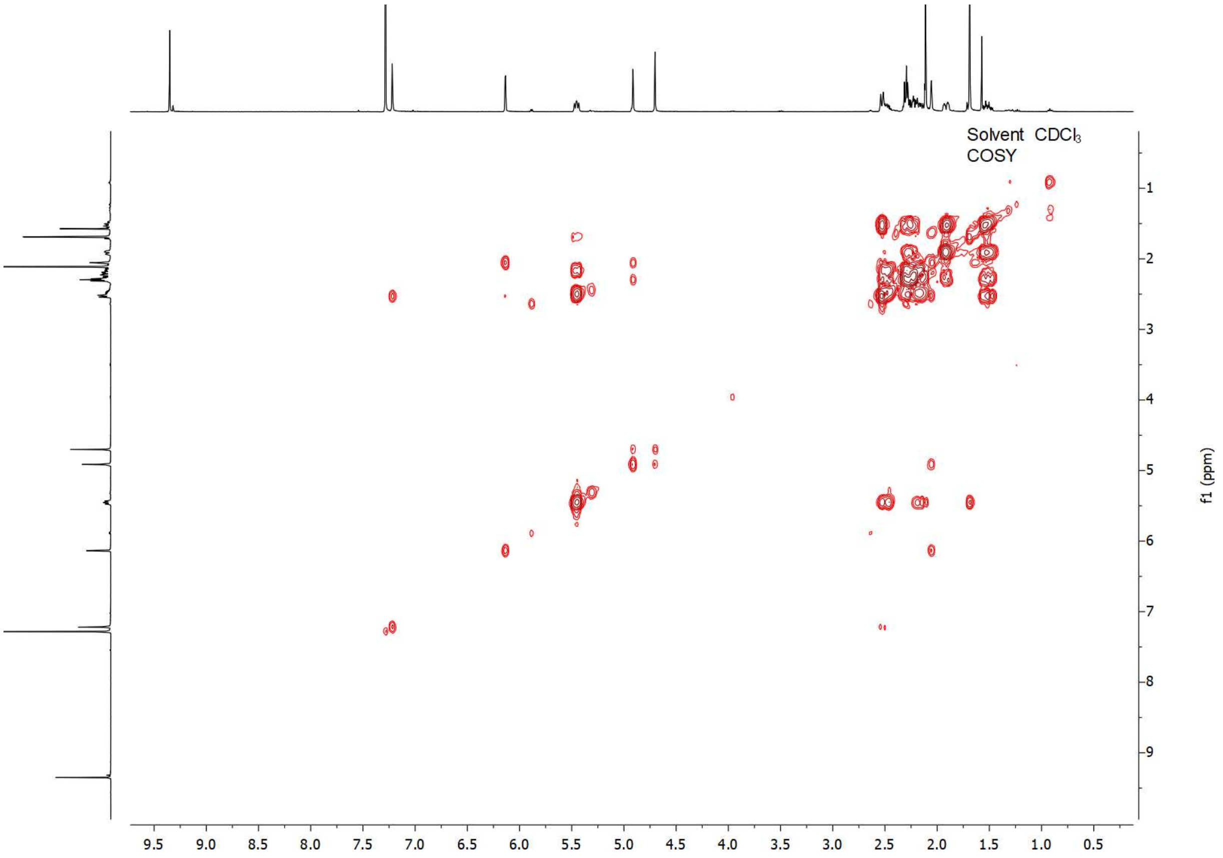
Solvent CDCl3
COSY
f1 (ppm)

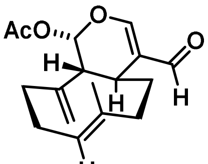

26α

Solvent C6D6
MHz 700
Nucleus 1H
Temp 70 °C

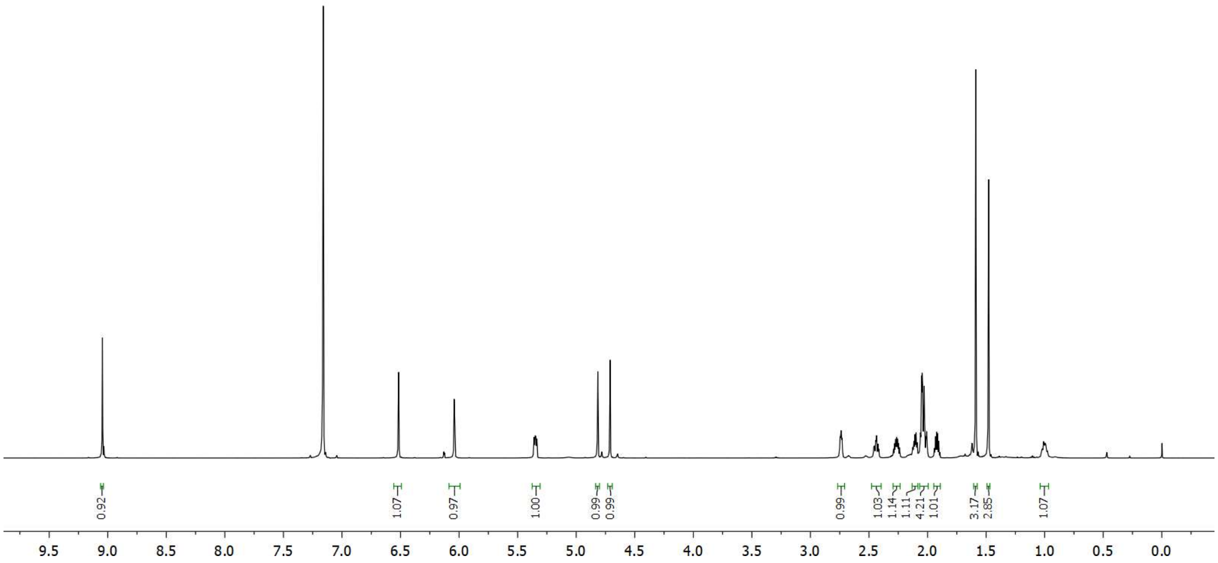

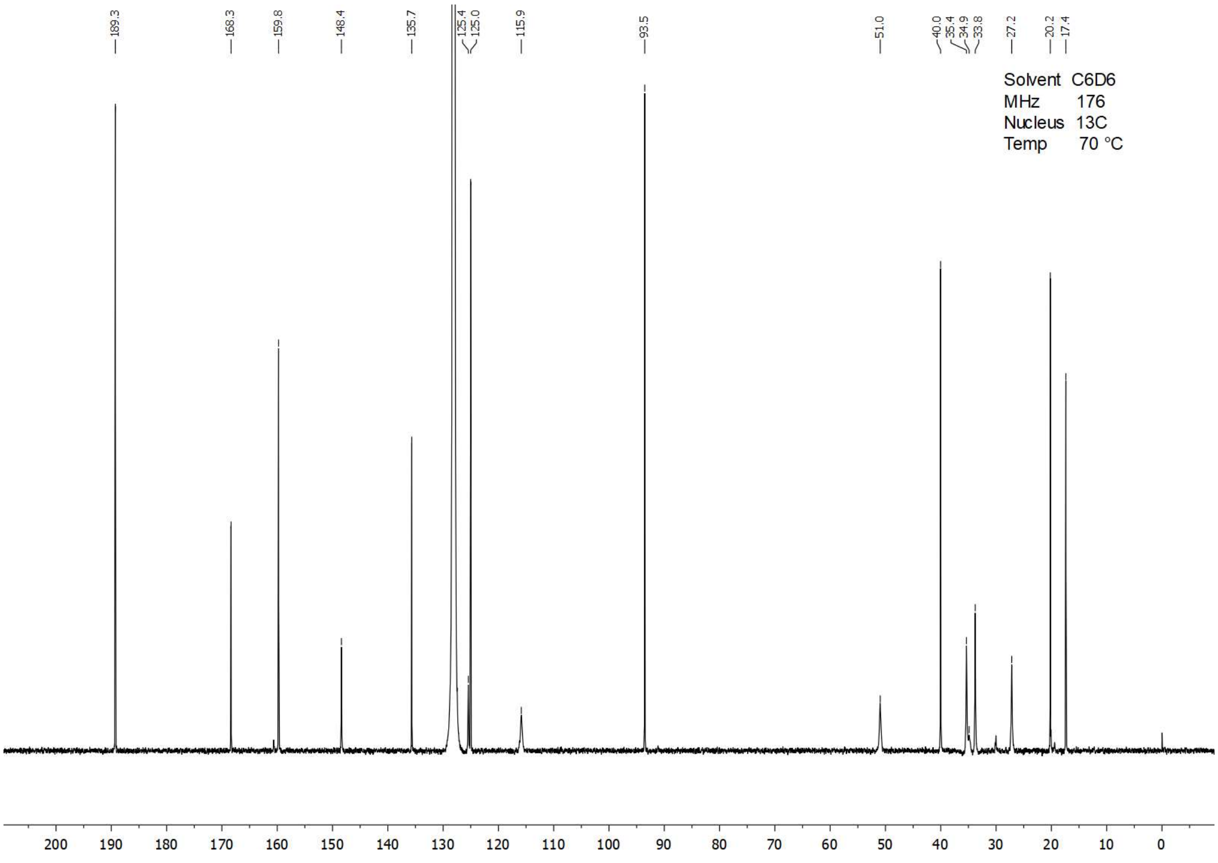

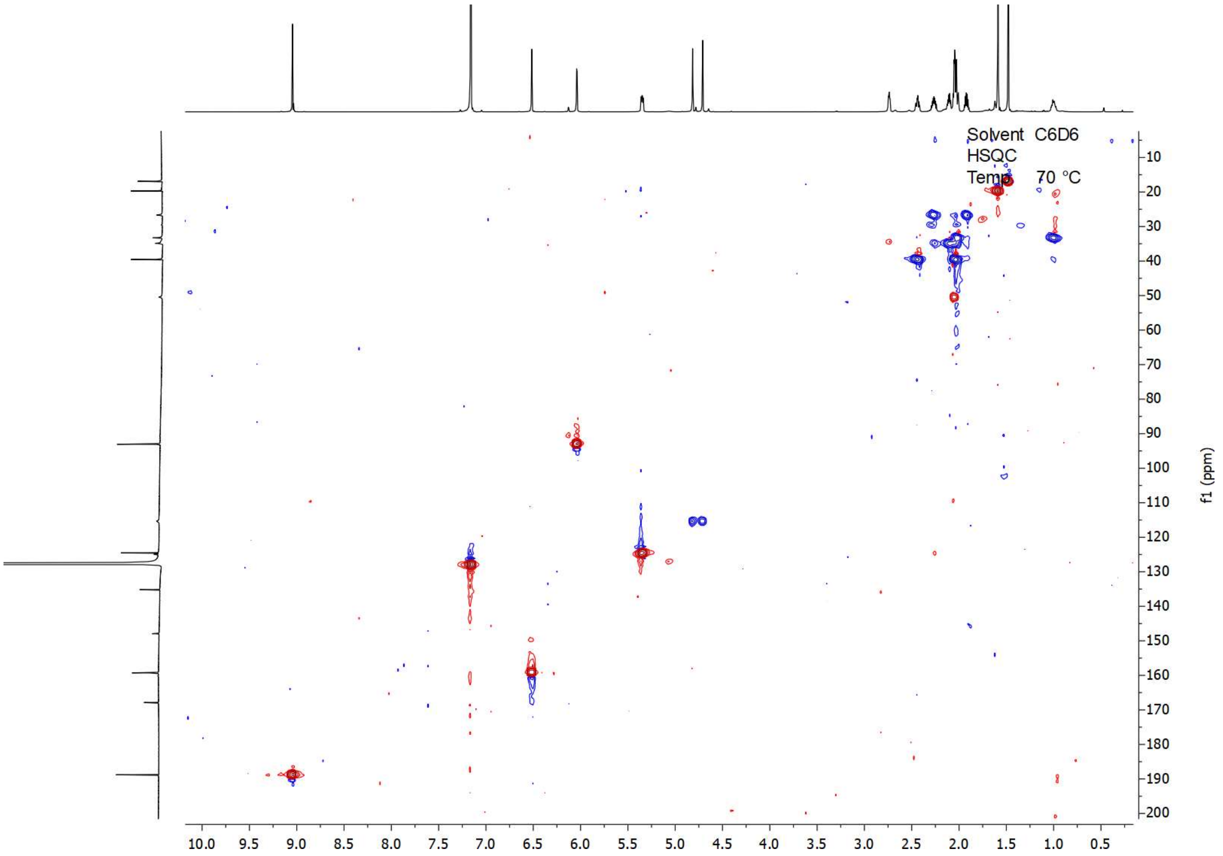

Solvent C6D6
HSQC
Temp 70 °C
f1 (ppm)

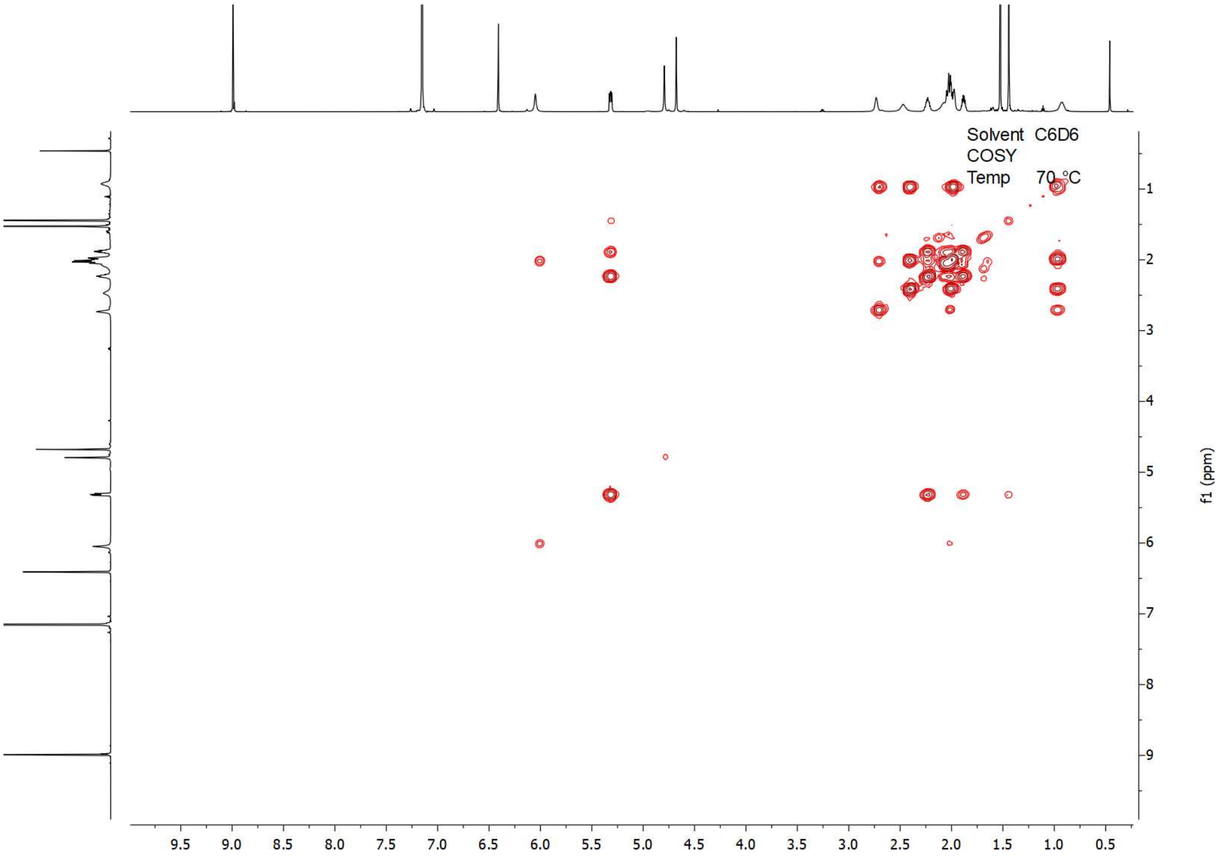

Solvent C6D6
COSY
Temp 70 °C
f1 (ppm)

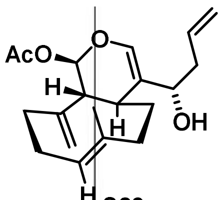
AcO
O
H
H
OH
H

S23a

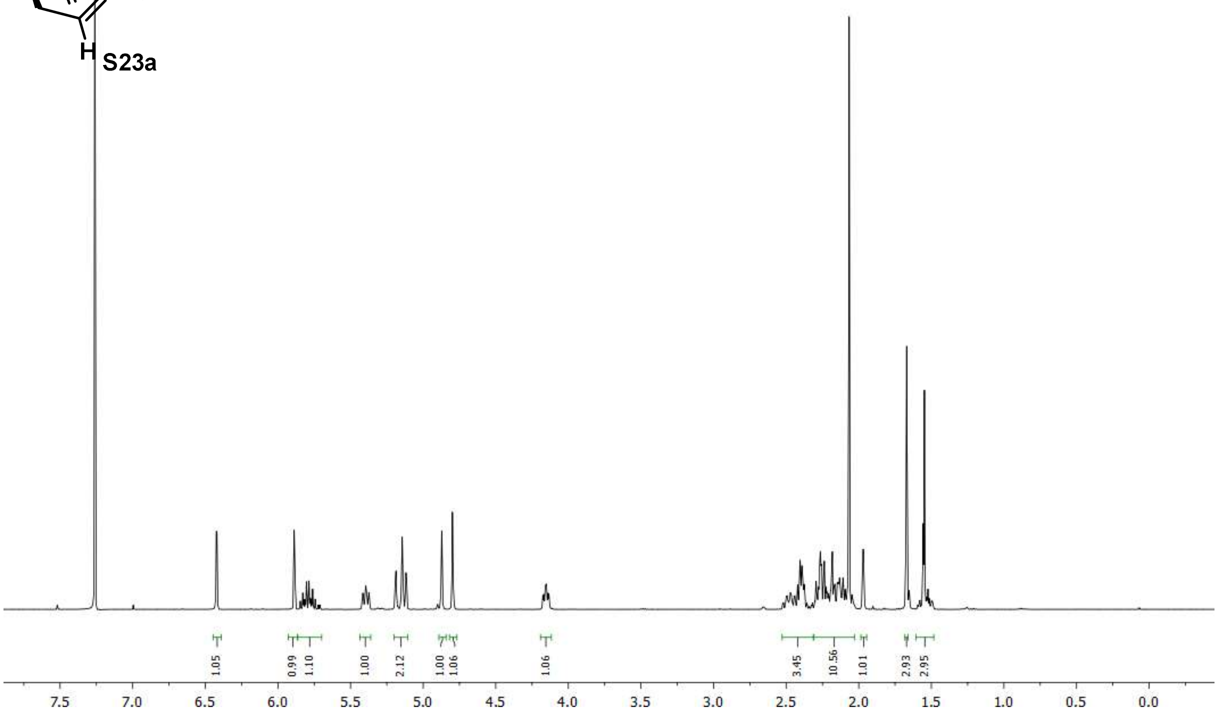
Solvent CDCl3
MHz 400
Nucleus 1H
1.05
0.99
1.10
1.00
2.12
1.00
1.06
1.06
3.45
10.56
1.01
2.93
2.95
7.5 7.0 6.5 6.0 5.5 5.0 4.5 4.0 3.5 3.0 2.5 2.0 1.5 1.0 0.5 0.0

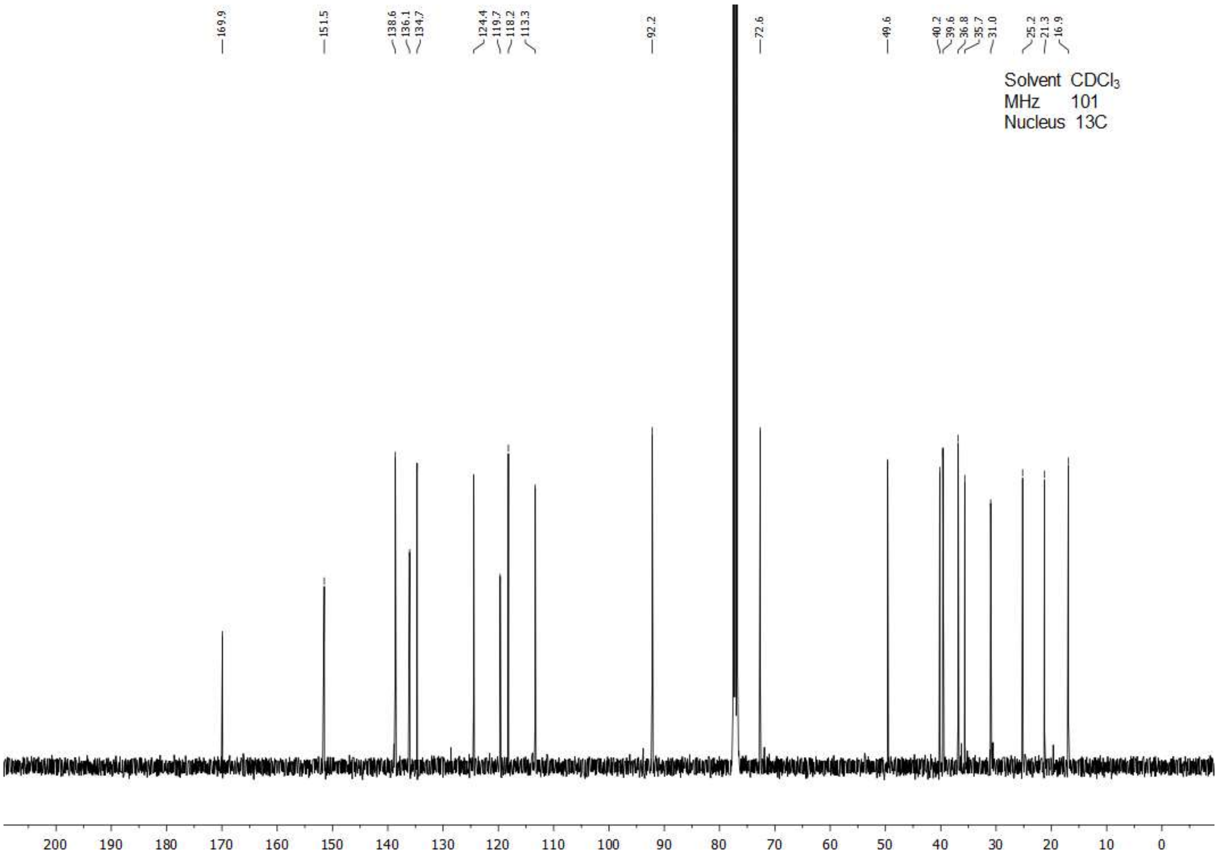
169.9
151.5
138.6
136.1
134.7
124.4
119.7
118.2
113.3
92.2
72.6
49.6
40.2
39.6
36.8
35.7
31.0
25.2
21.3
16.9
Solvent CDCl3
MHz 101
Nucleus 13C
200 190 180 170 160 150 140 130 120 110 100 90 80 70 60 50 40 30 20 10 0

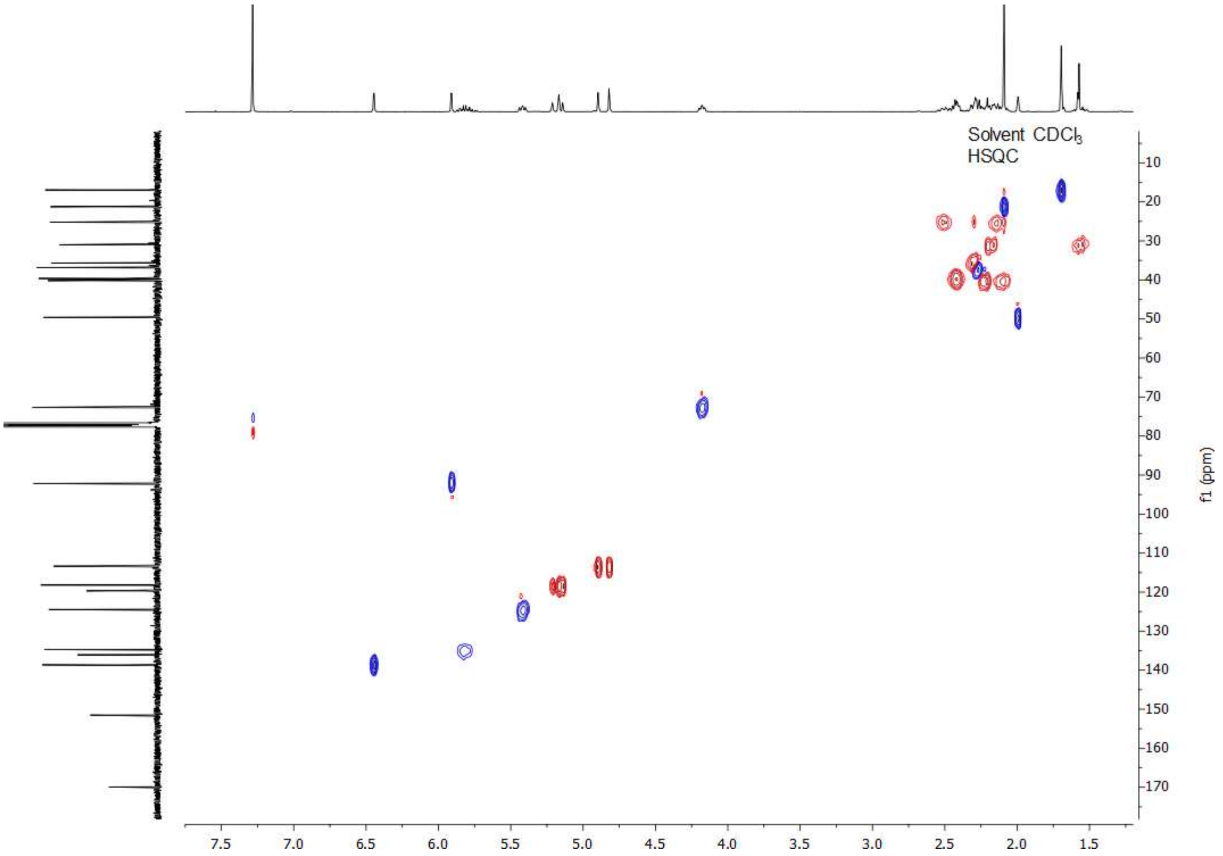
Solvent CDCl3
HSQC
f1 (ppm)

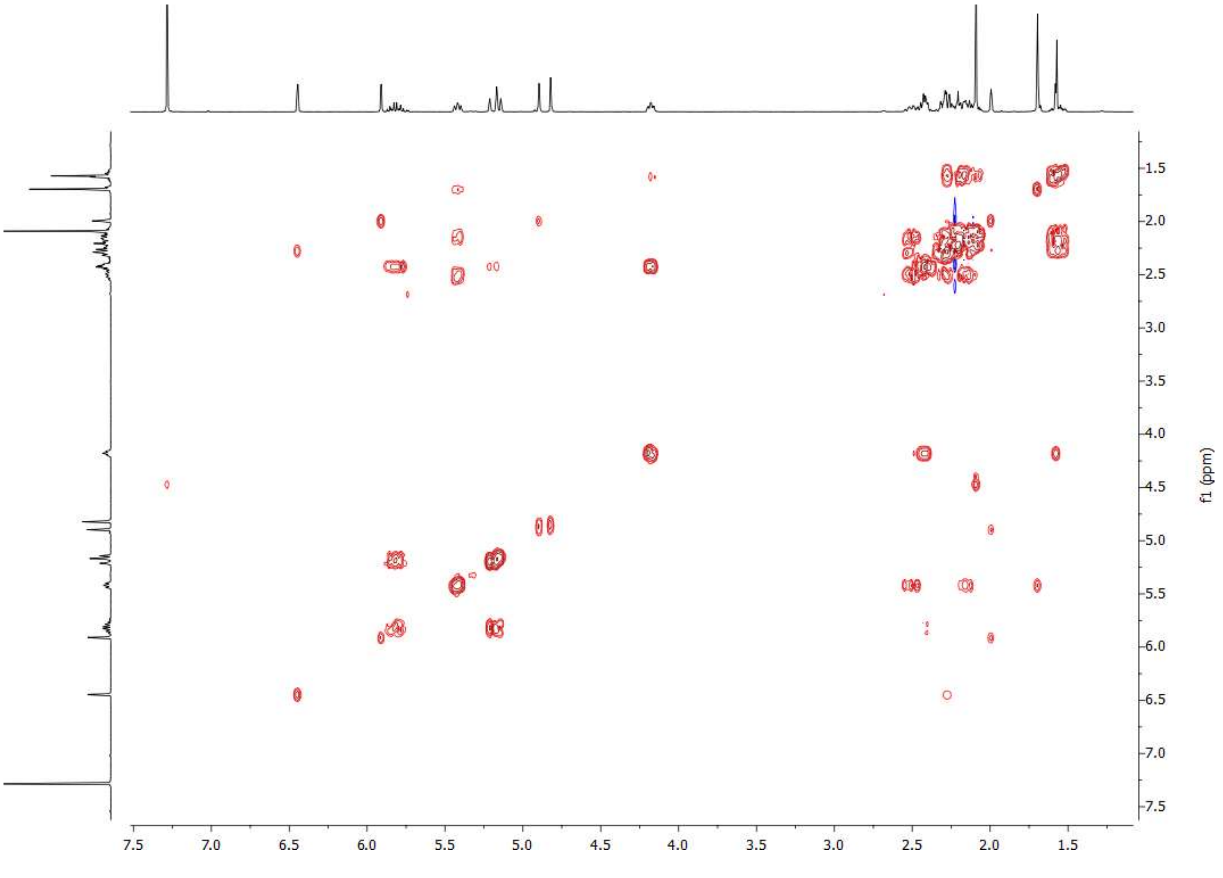
f1 (ppm)

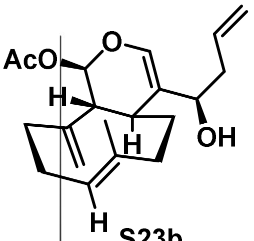

S23b

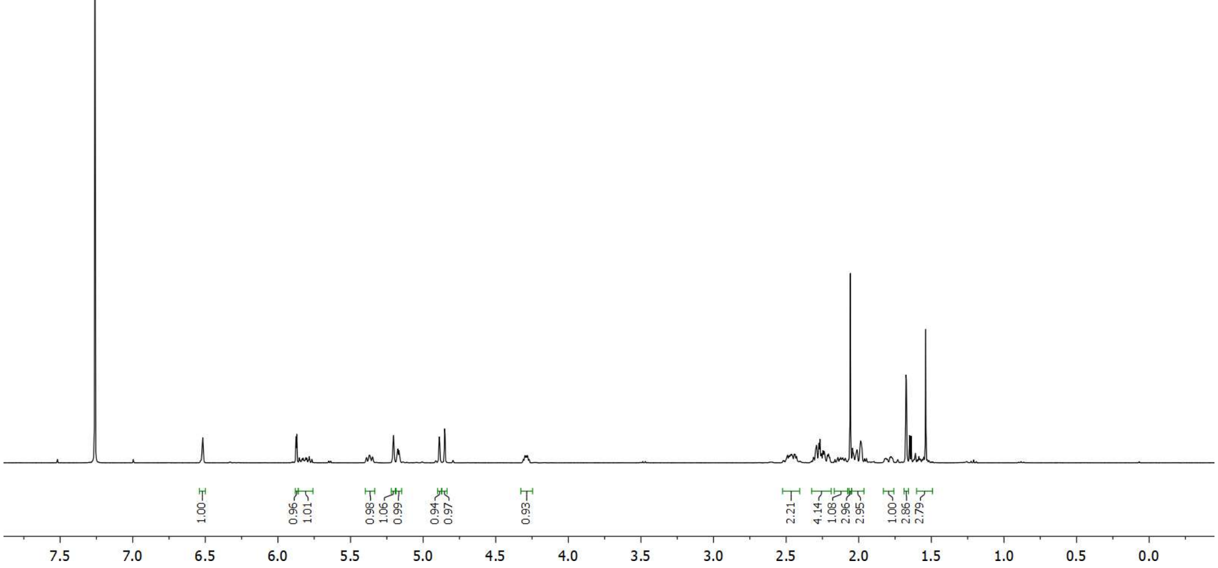
Solvent CDCl$_3$
MHz 400
Nucleus 1H
1.00
0.96
1.01
0.98
1.06
0.99
0.94
0.97
0.93
2.21
4.14
1.08
2.96
2.95
1.00
2.86
2.79
7.5 7.0 6.5 6.0 5.5 5.0 4.5 4.0 3.5 3.0 2.5 2.0 1.5 1.0 0.5 0.0

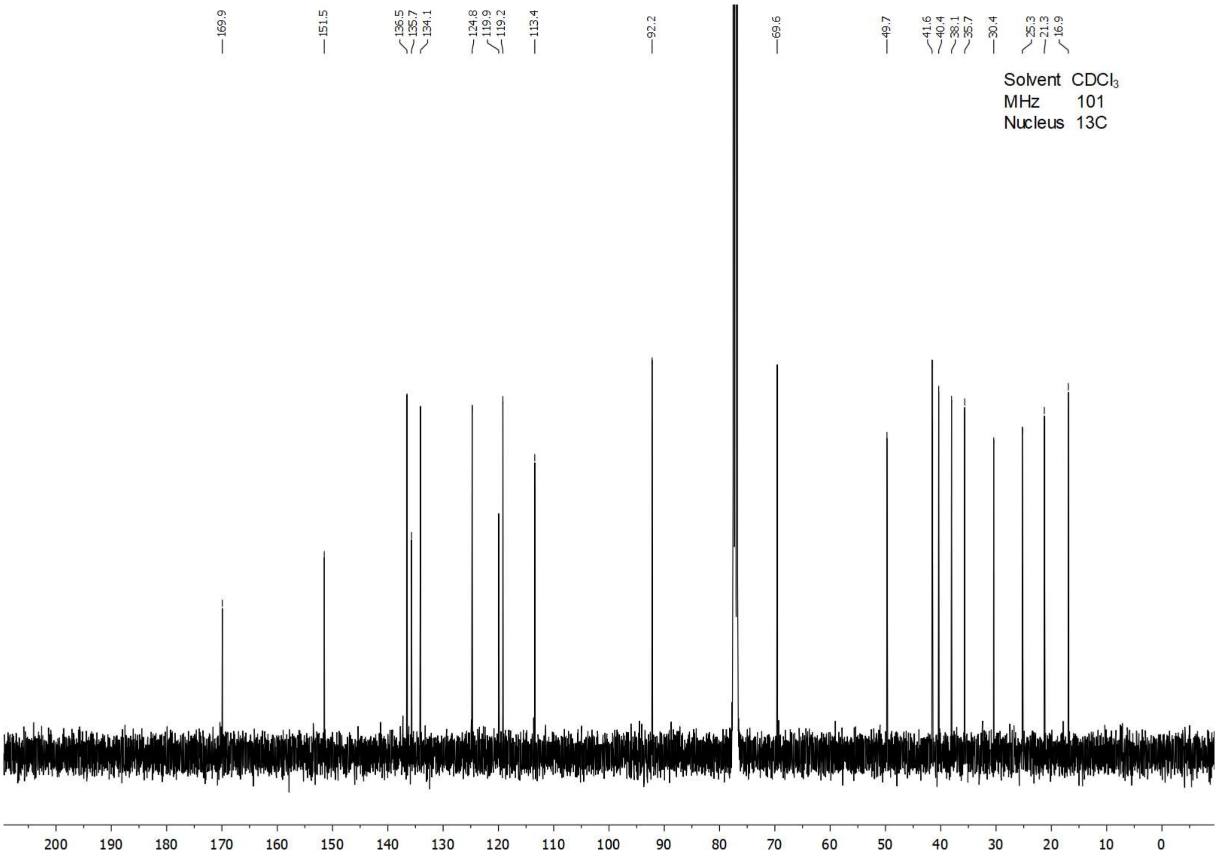
169.9
151.5
136.5
135.7
134.1
124.8
119.9
119.2
113.4
92.2
69.6
49.7
41.6
40.4
38.1
35.7
30.4
25.3
21.3
16.9
Solvent CDCl$_3$
MHz 101
Nucleus 13C
200 190 180 170 160 150 140 130 120 110 100 90 80 70 60 50 40 30 20 10 0

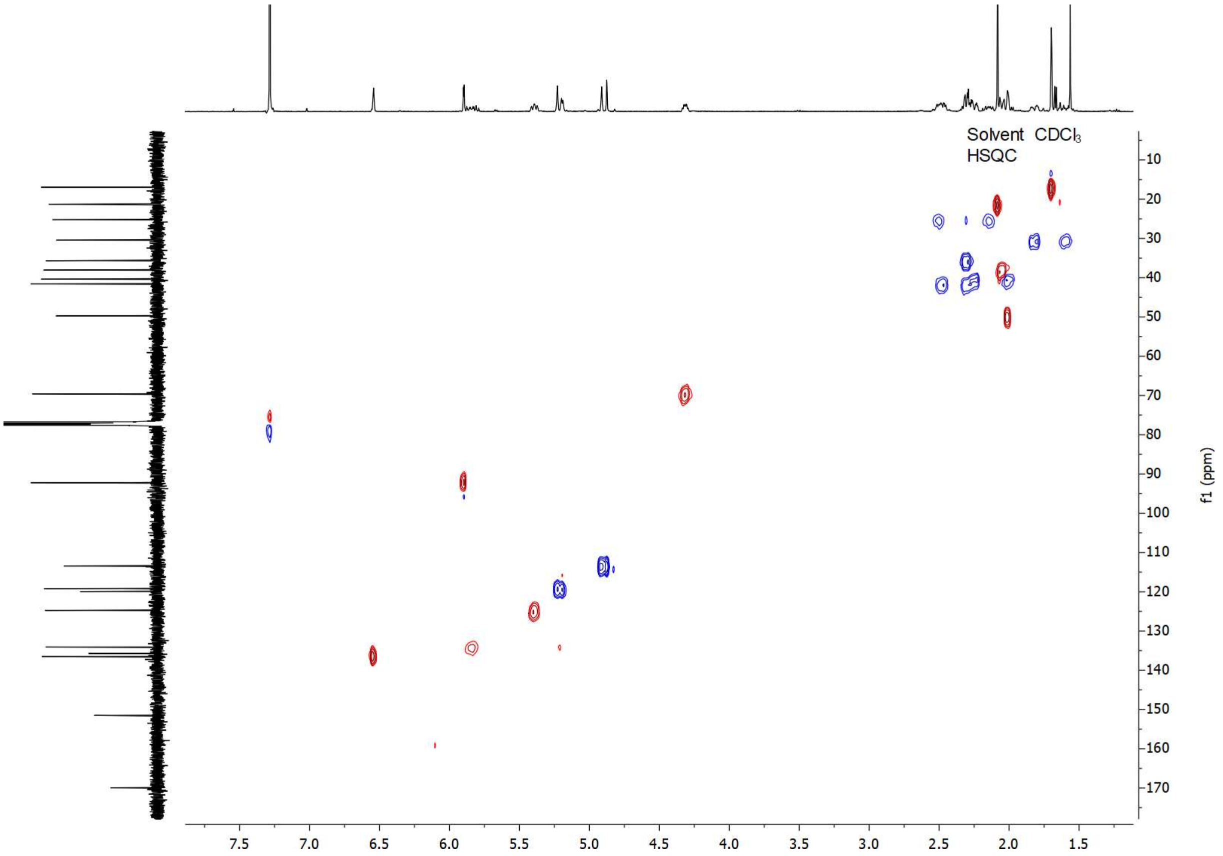
Solvent CDCl3
HSQC
f1 (ppm)

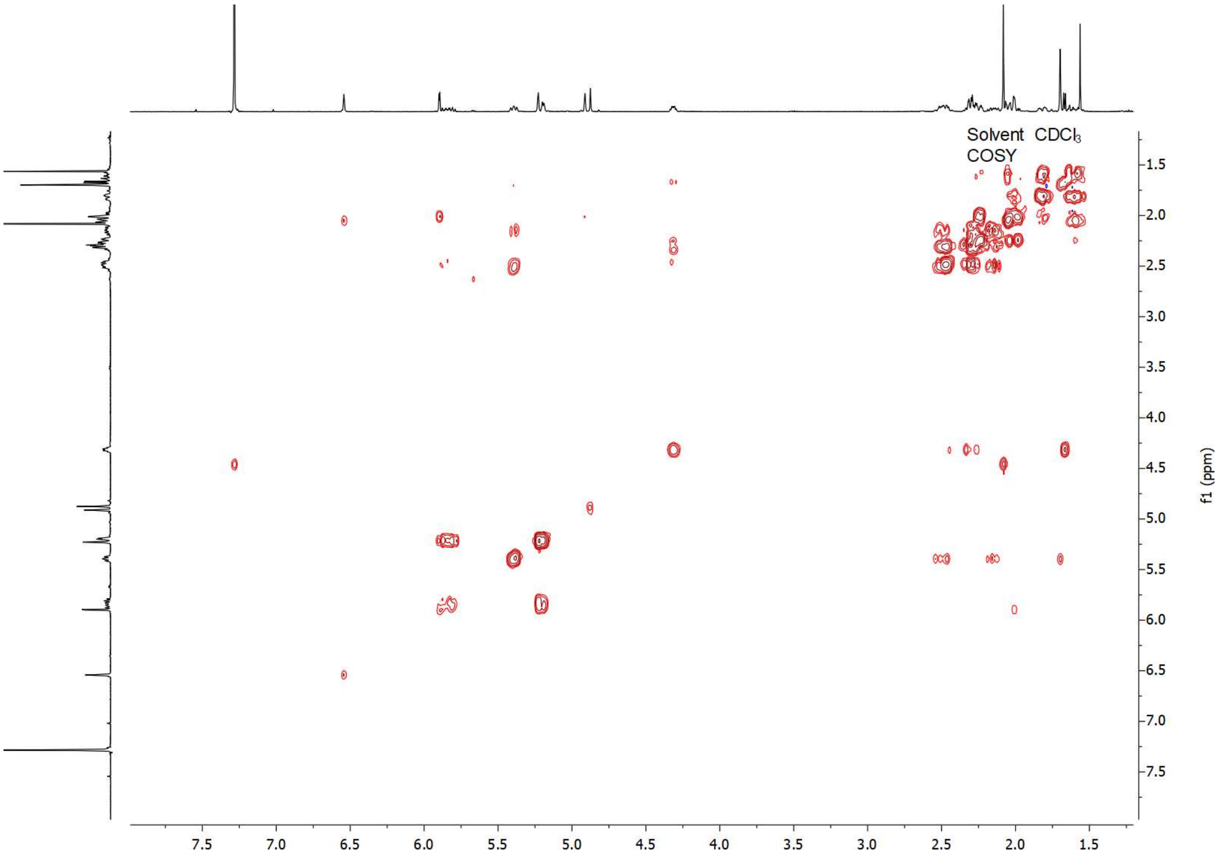
Solvent CDCl3
COSY
f1 (ppm)

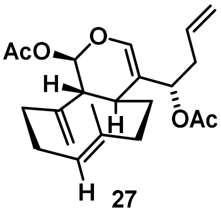

27

Solvent CDCl$_3$
MHz 400
Nucleus 1H

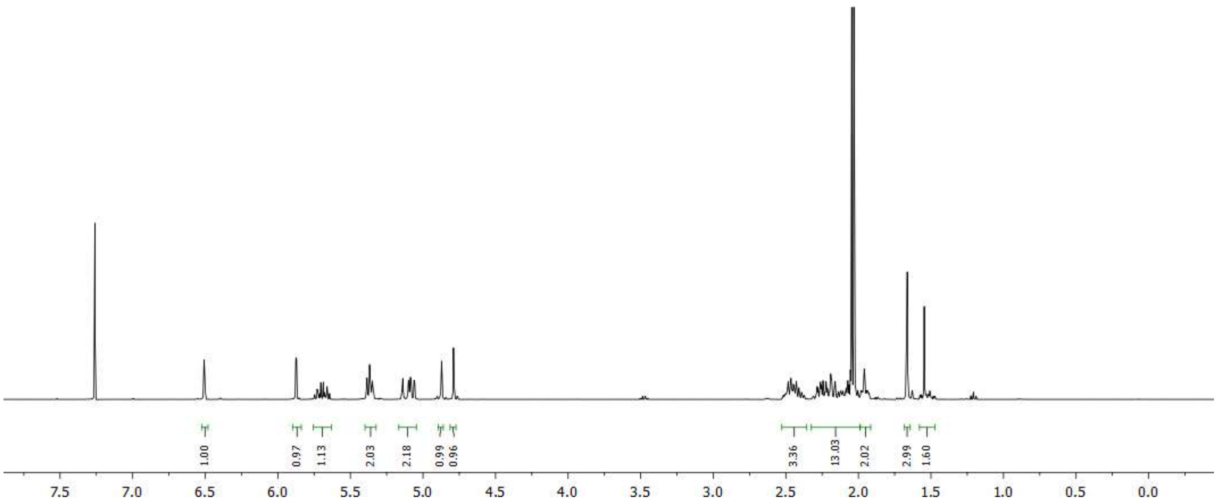

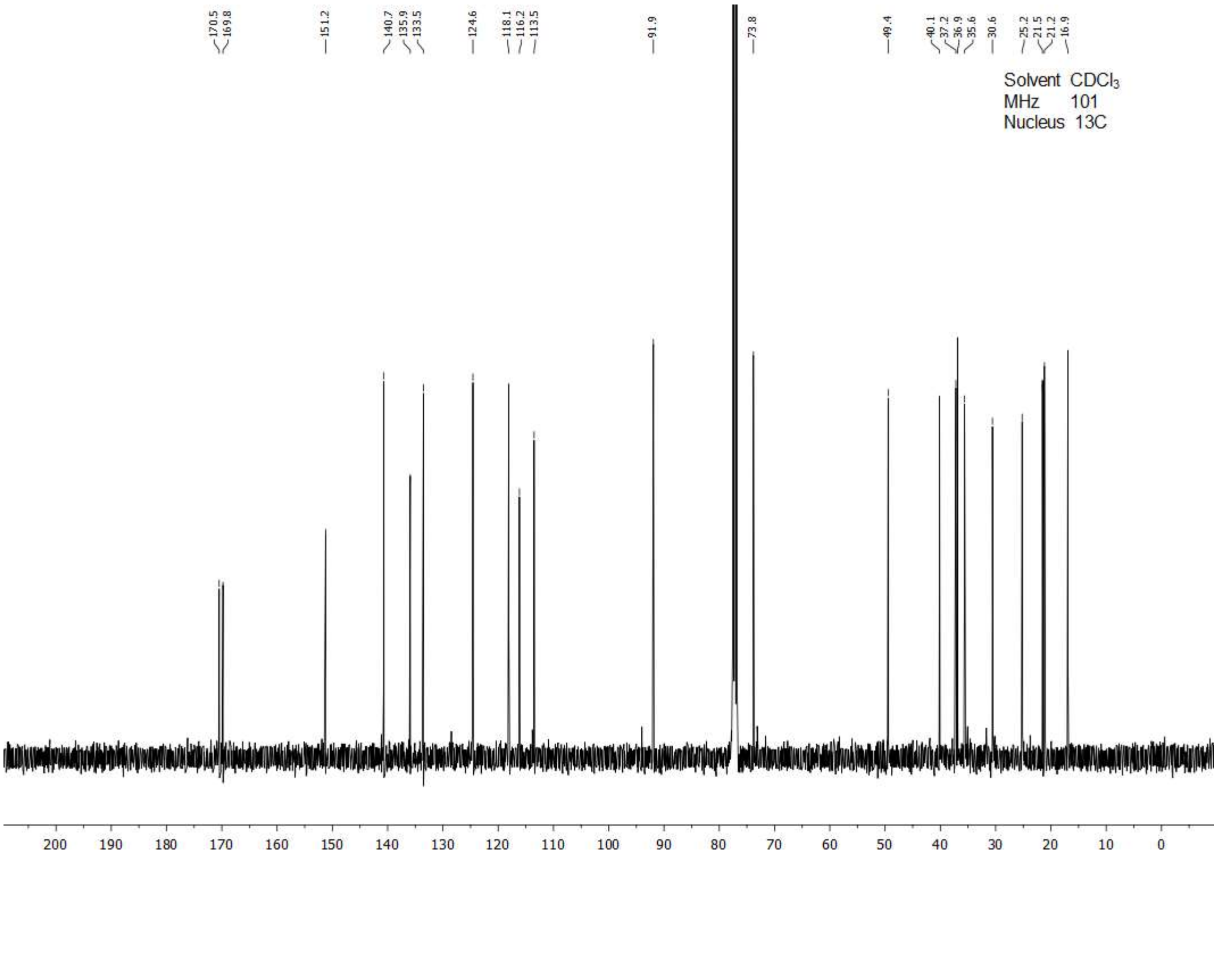

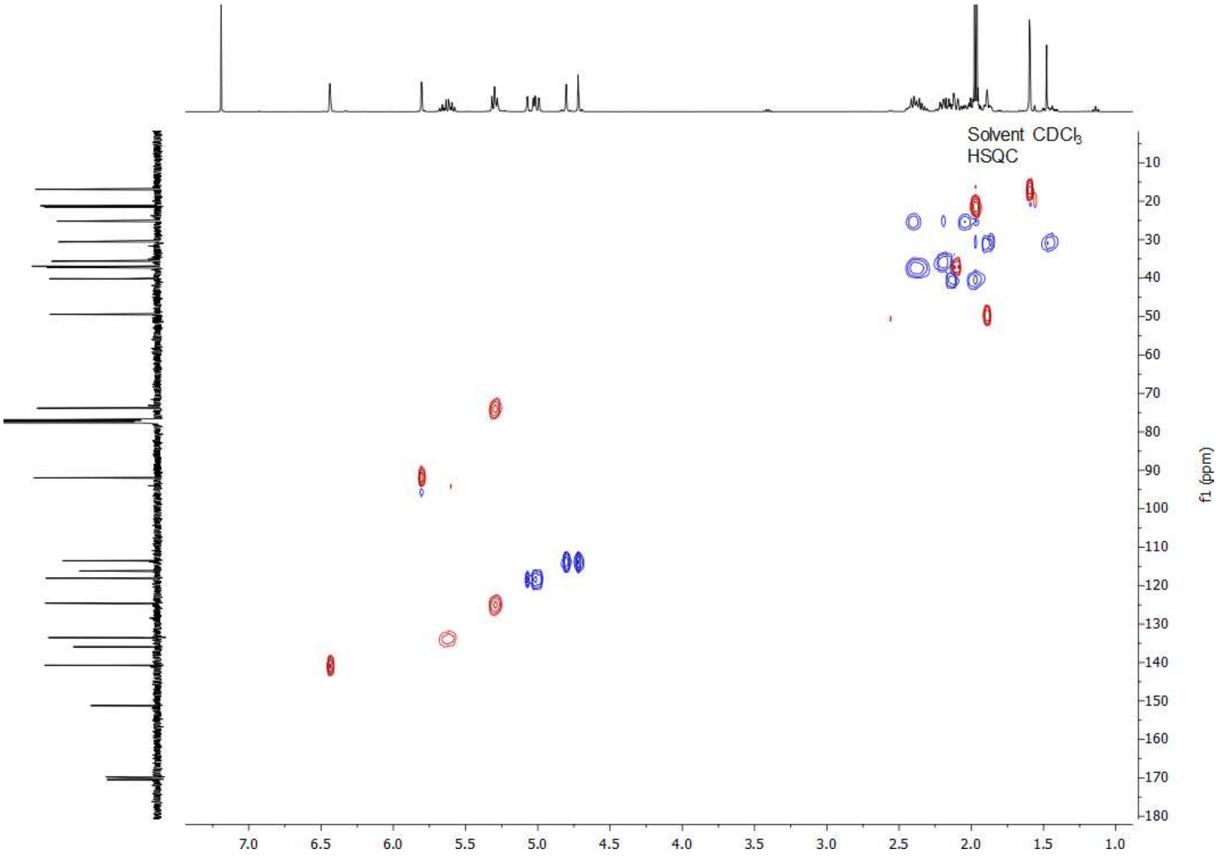
Solvent CDCl3
HSQC
f1 (ppm)

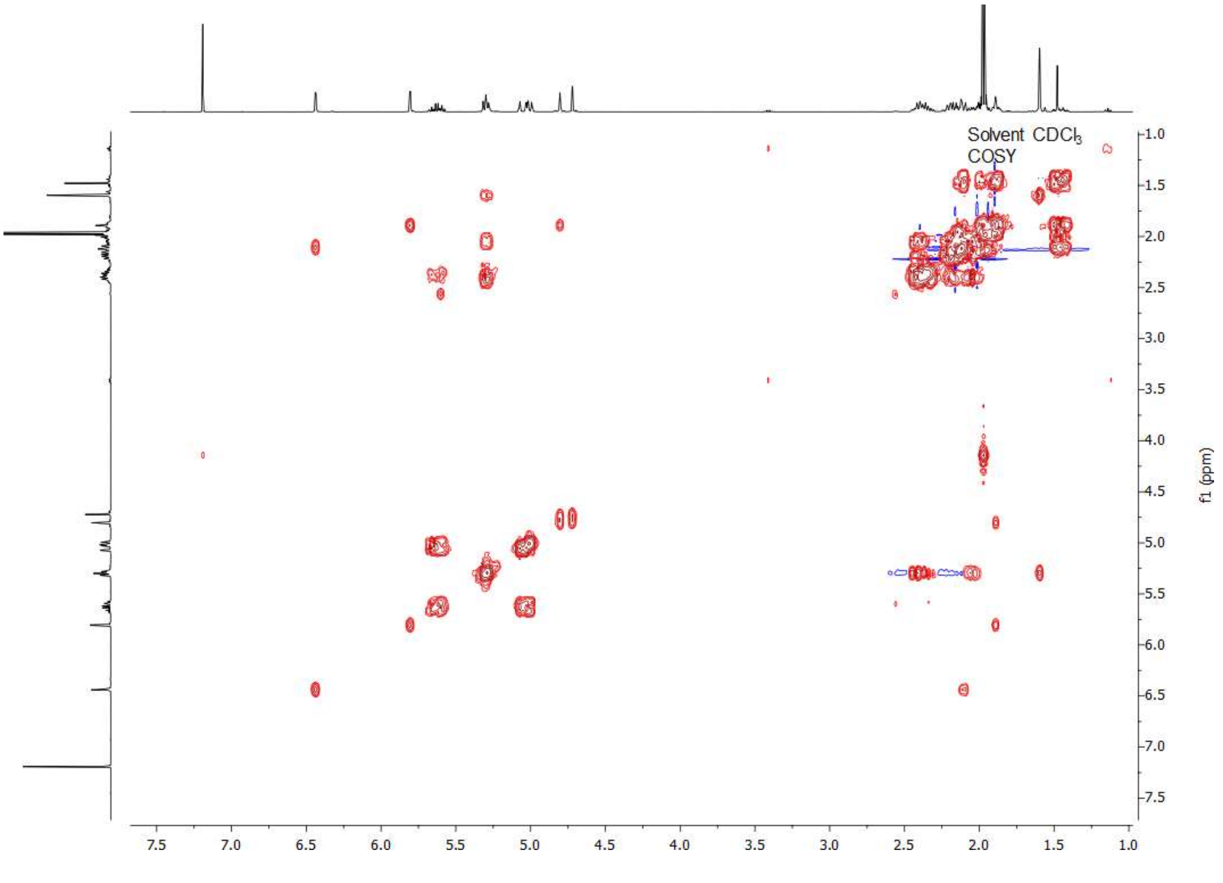
Solvent CDCl3
COSY
f1 (ppm)

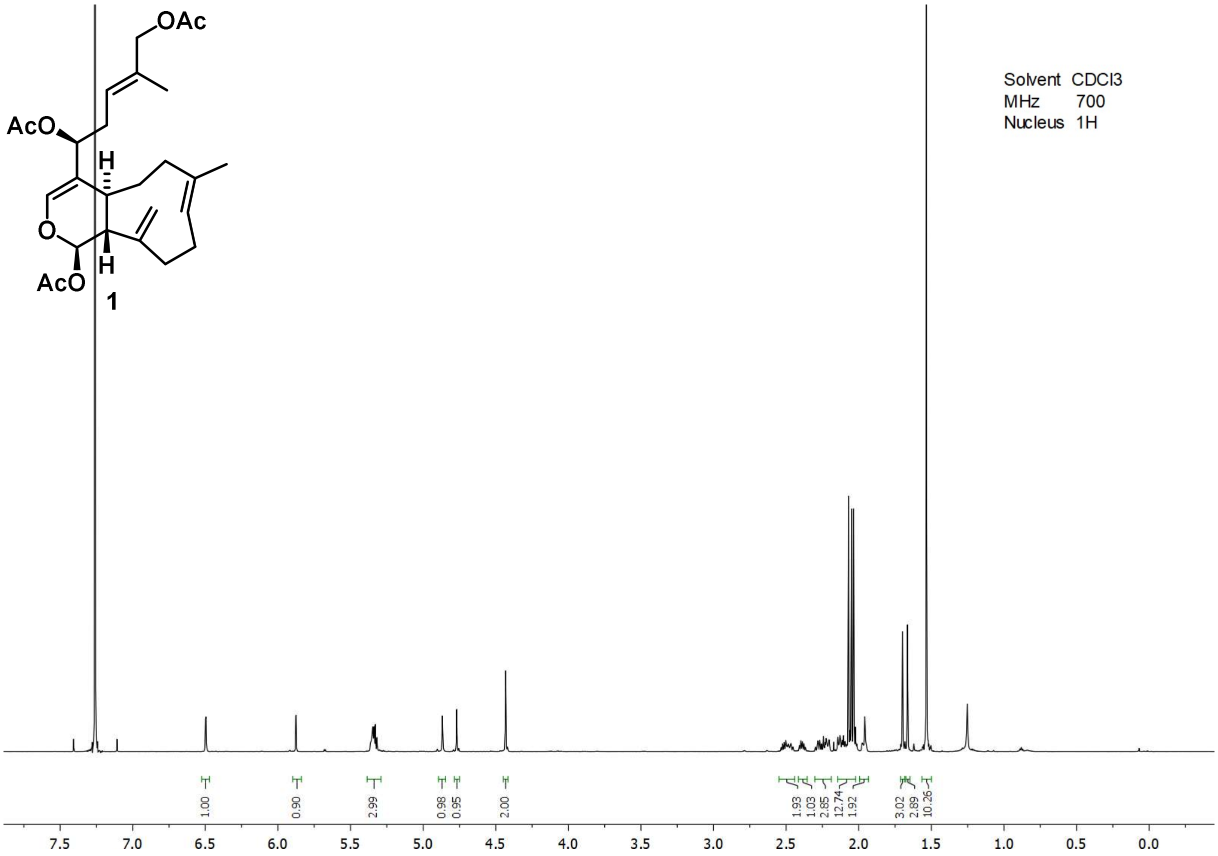
OAc
AcO
H
O
H
AcO
1
Solvent CDCl3
MHz 700
Nucleus 1H
1.00
0.90
2.99
0.98
0.95
2.00
1.93
1.03
2.85
12.74
1.92
3.02
2.89
10.26
7.5 7.0 6.5 6.0 5.5 5.0 4.5 4.0 3.5 3.0 2.5 2.0 1.5 1.0 0.5 0.0

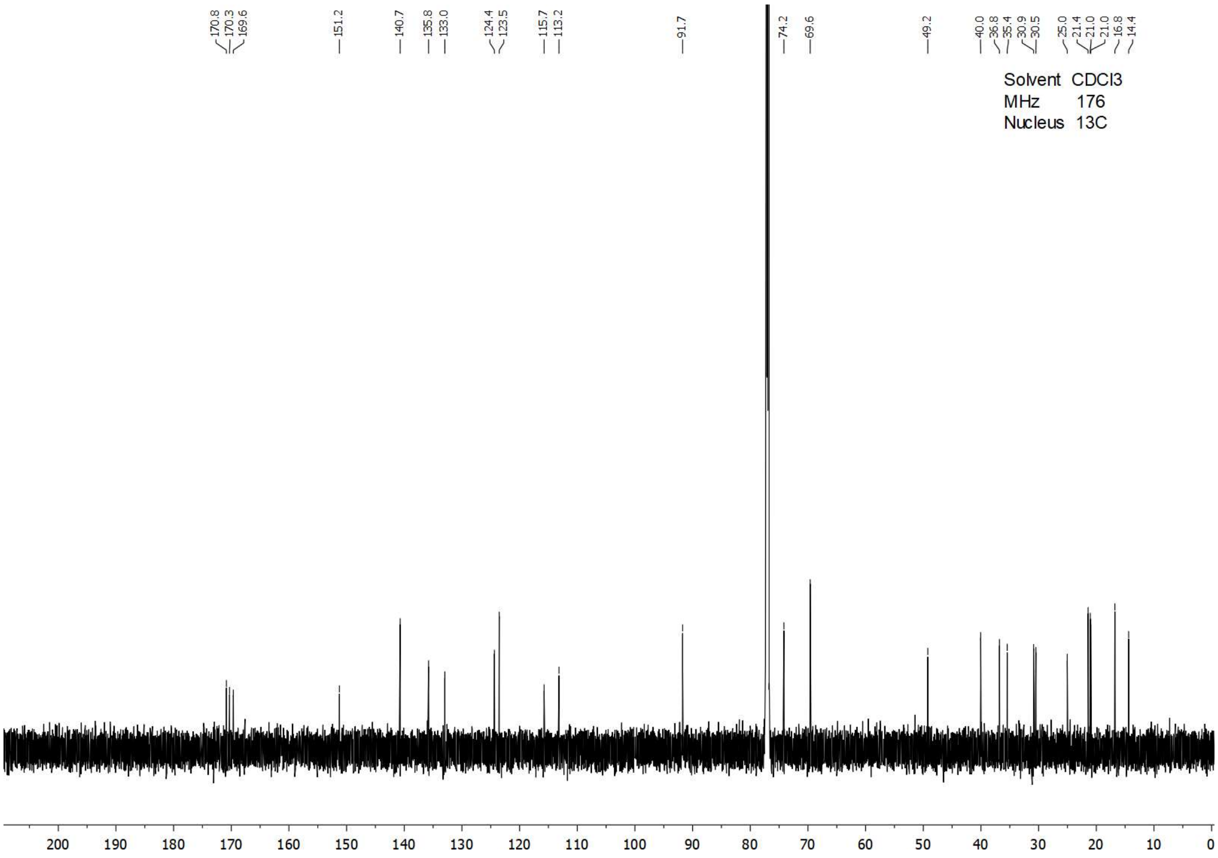
170.8
170.3
169.6
151.2
140.7
135.8
133.0
124.4
123.5
115.7
113.2
91.7
74.2
69.6
49.2
40.0
36.8
35.4
30.9
30.5
25.0
21.4
21.0
21.0
16.8
14.4
Solvent CDCl3
MHz 176
Nucleus 13C
200 190 180 170 160 150 140 130 120 110 100 90 80 70 60 50 40 30 20 10 0

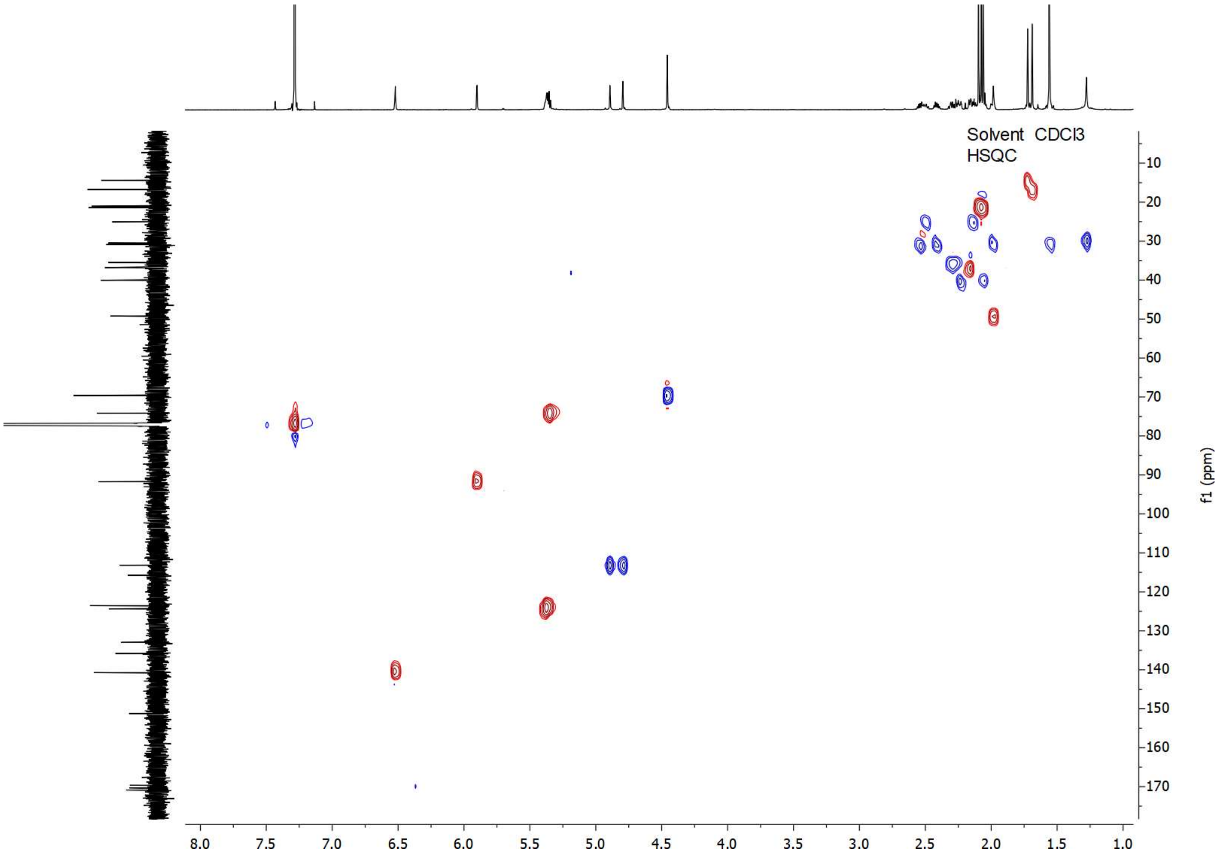
Solvent CDCl3
HSQC
f1 (ppm)

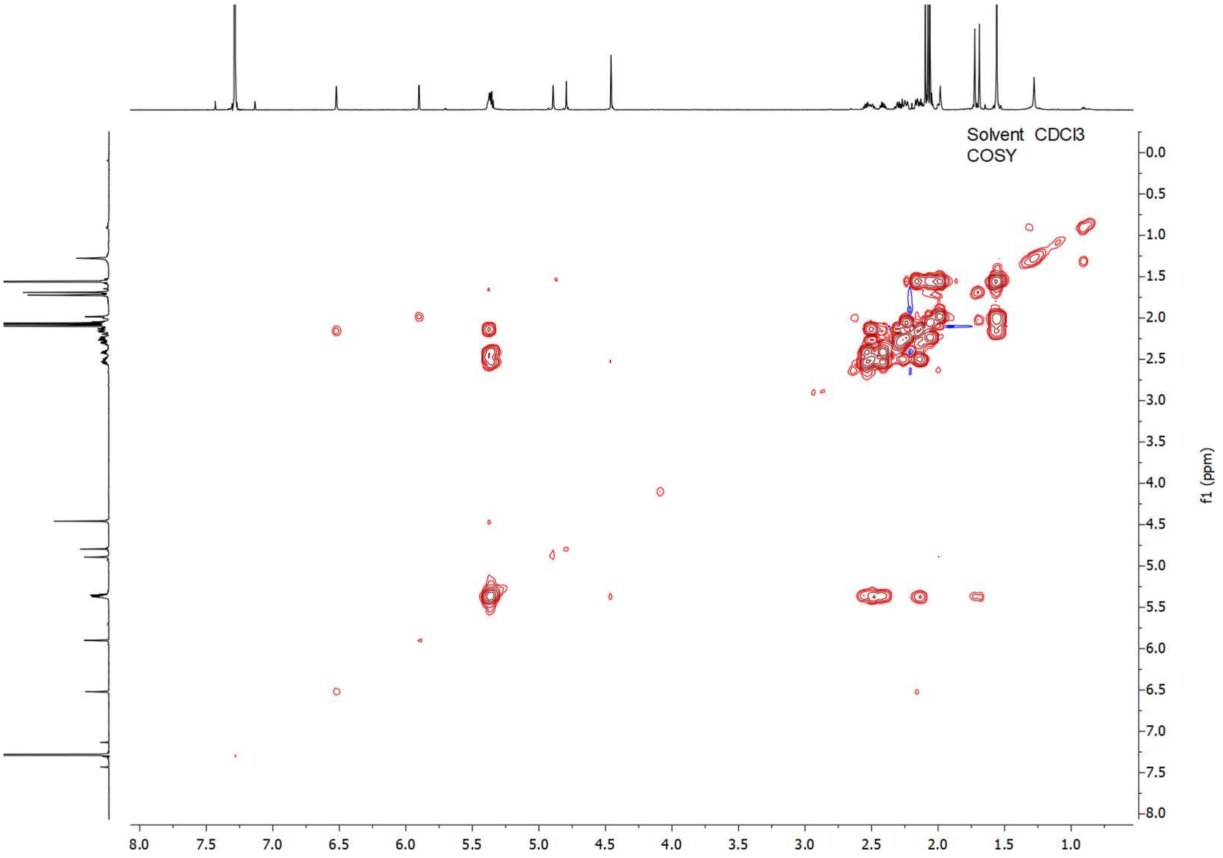
Solvent CDCl3
COSY
f1 (ppm)

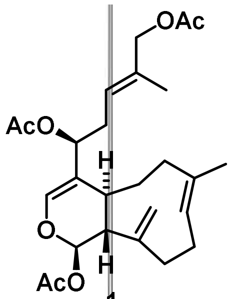
OAc
AcO
H
O
H
AcO

1

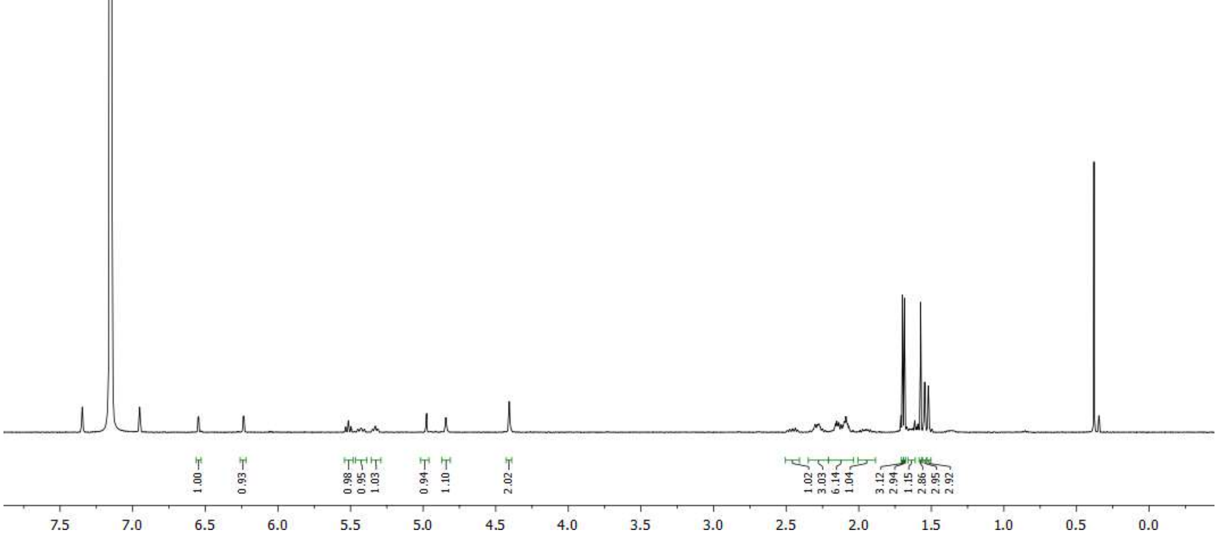
Solvent C6D6
MHz 700
Nucleus 1H
7.5 7.0 6.5 6.0 5.5 5.0 4.5 4.0 3.5 3.0 2.5 2.0 1.5 1.0 0.5 0.0

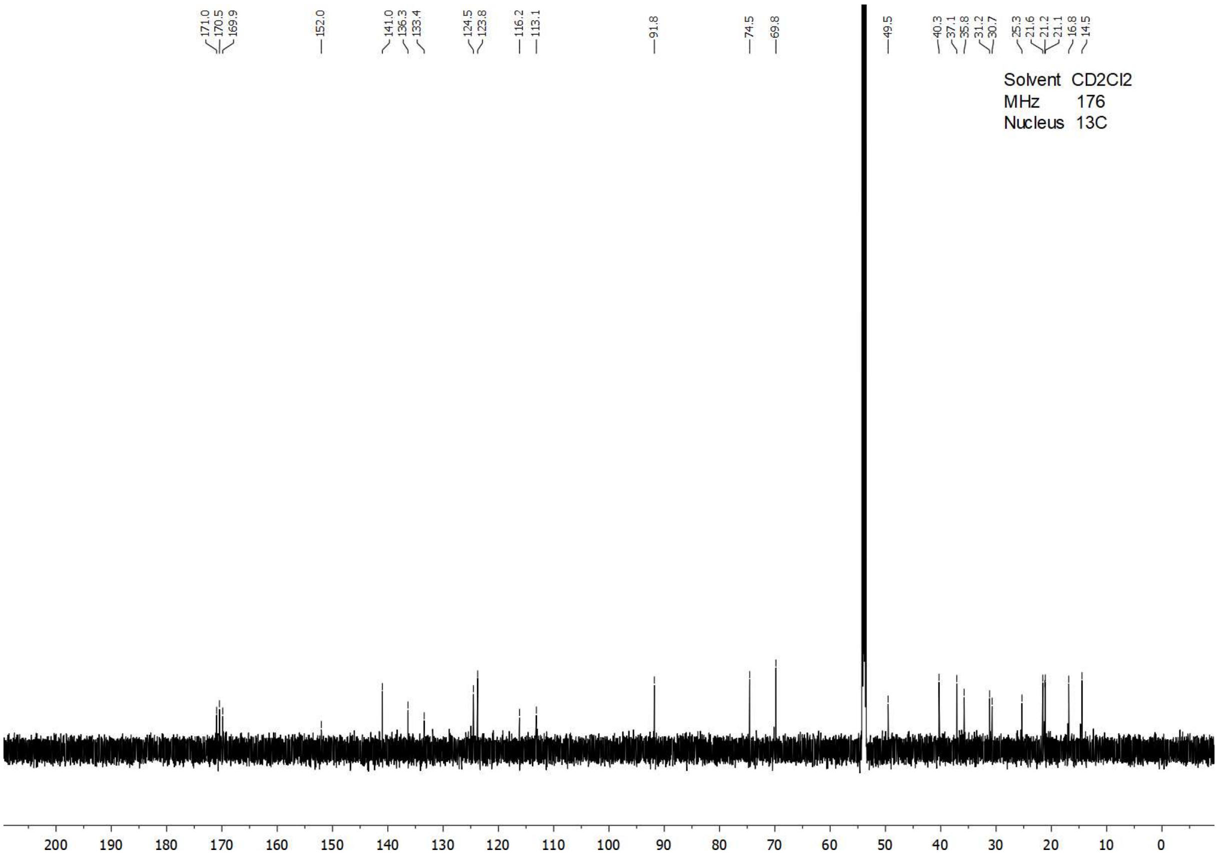
171.0
170.5
169.9
152.0
141.0
136.3
133.4
124.5
123.8
116.2
113.1
91.8
74.5
69.8
49.5
40.3
37.1
35.8
31.2
30.7
25.3
21.6
21.2
21.1
16.8
14.5
Solvent CD2Cl2
MHz 176
Nucleus 13C
200 190 180 170 160 150 140 130 120 110 100 90 80 70 60 50 40 30 20 10 0

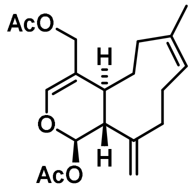
AcO
H
O
H
AcO

S24

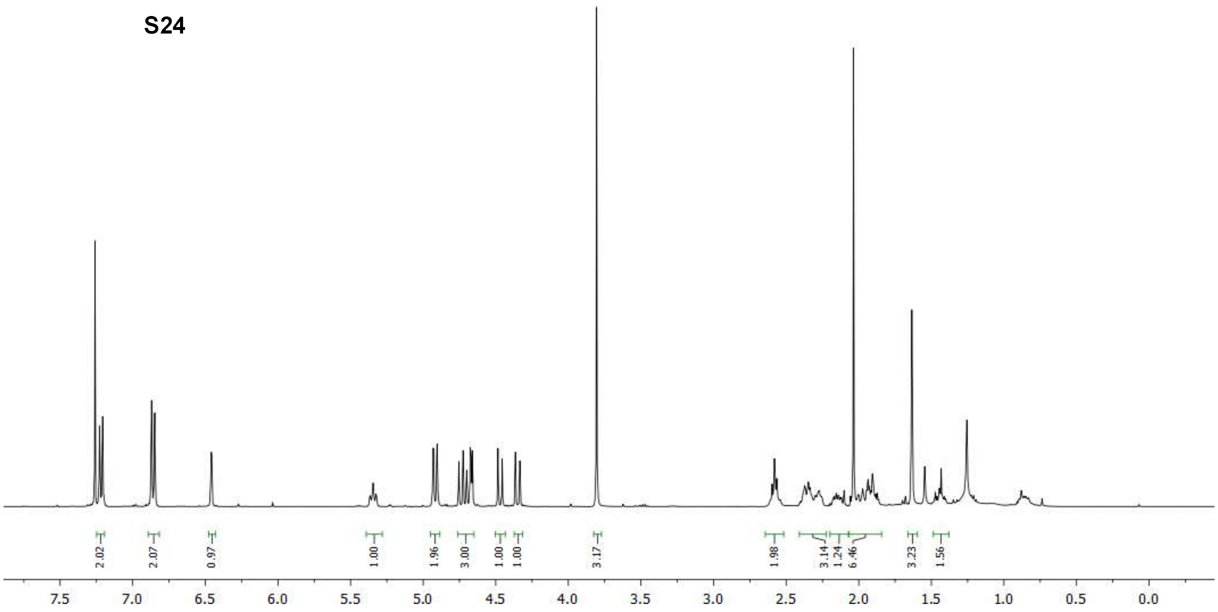
Solvent CDCl3
MHz 400
Nucleus 1H
2.02
2.07
0.97
1.00
1.96
3.00
1.00
1.00
3.17
1.98
3.14
1.24
6.46
3.23
1.56
7.5
7.0
6.5
6.0
5.5
5.0
4.5
4.0
3.5
3.0
2.5
2.0
1.5
1.0
0.5
0.0

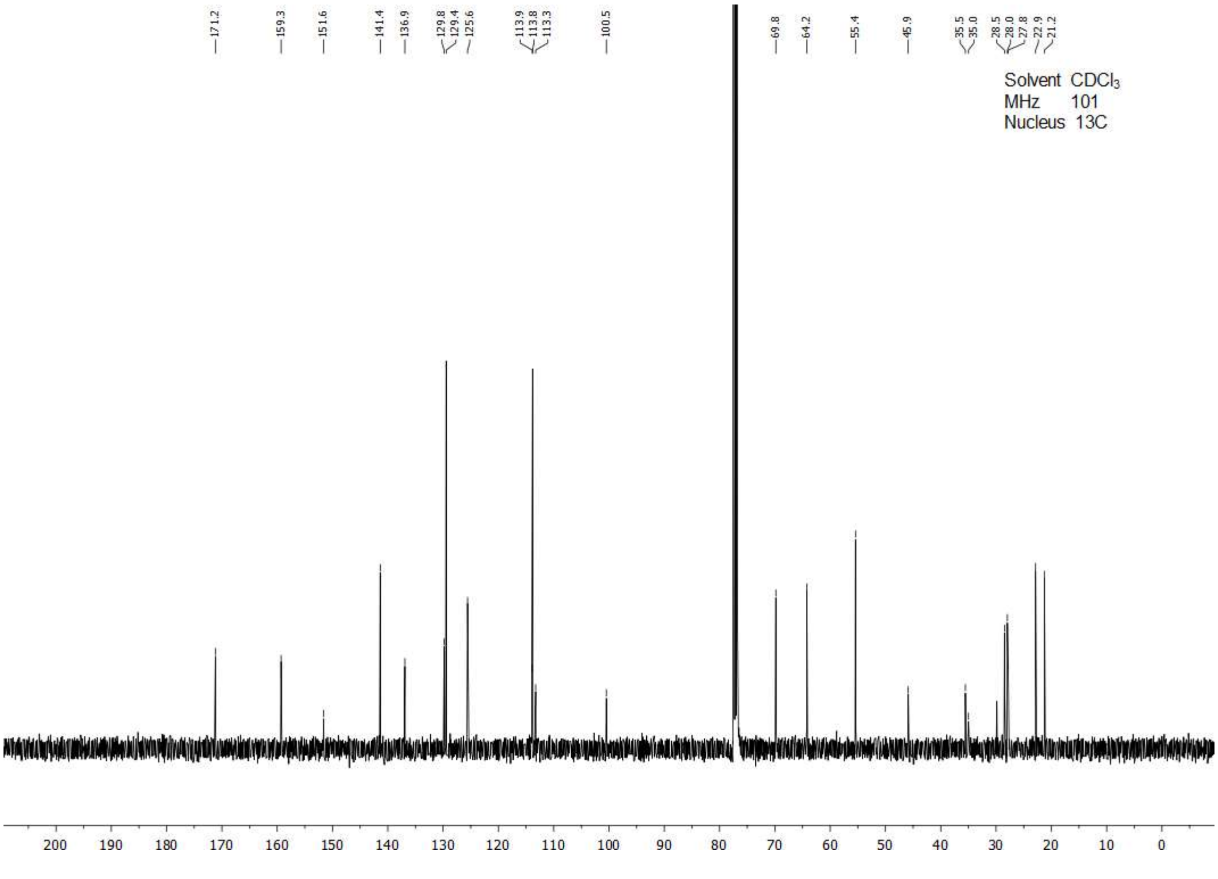
171.2
159.3
151.6
141.4
136.9
129.8
129.4
125.6
113.9
113.8
113.3
100.5
69.8
64.2
55.4
45.9
35.5
35.0
28.5
28.0
27.8
22.9
21.2
Solvent CDCl3
MHz 101
Nucleus 13C
200
190
180
170
160
150
140
130
120
110
100
90
80
70
60
50
40
30
20
10
0

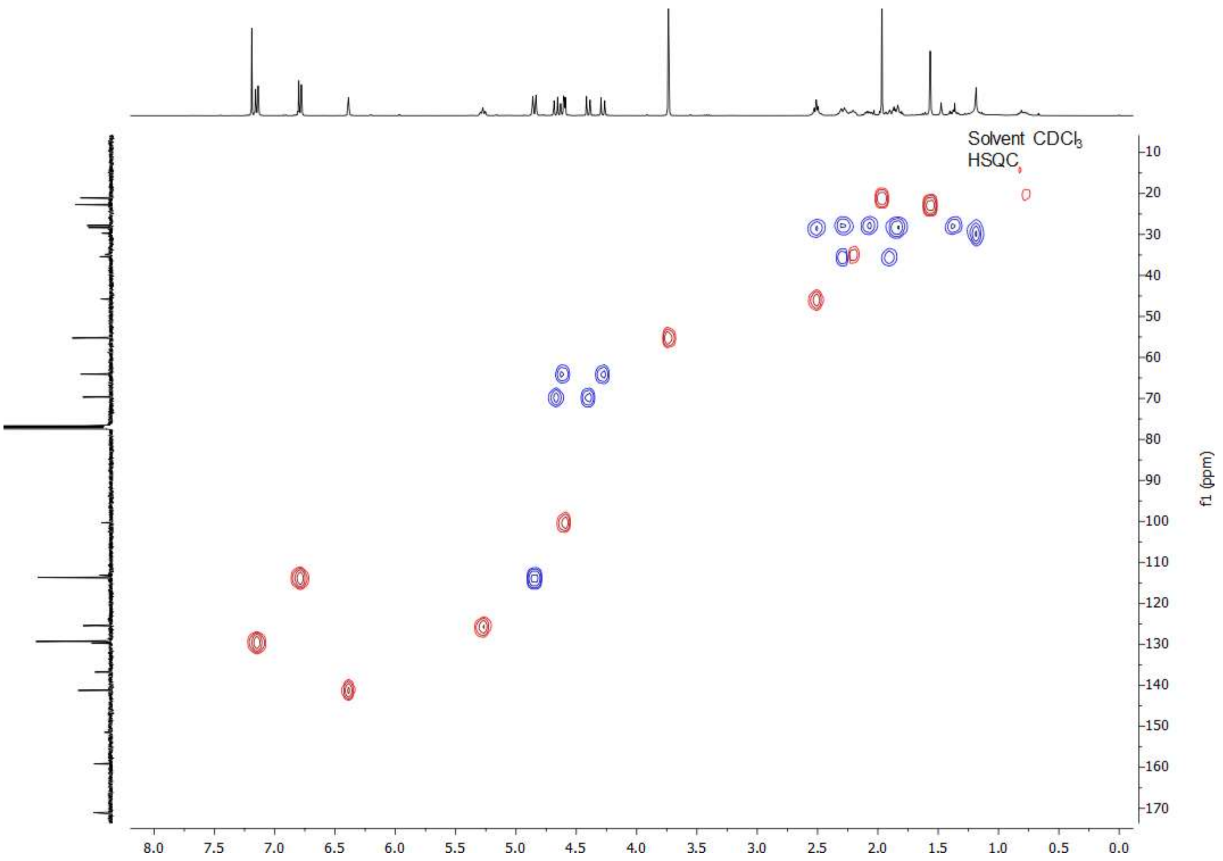

Solvent CDCl3
HSQC
f1 (ppm)

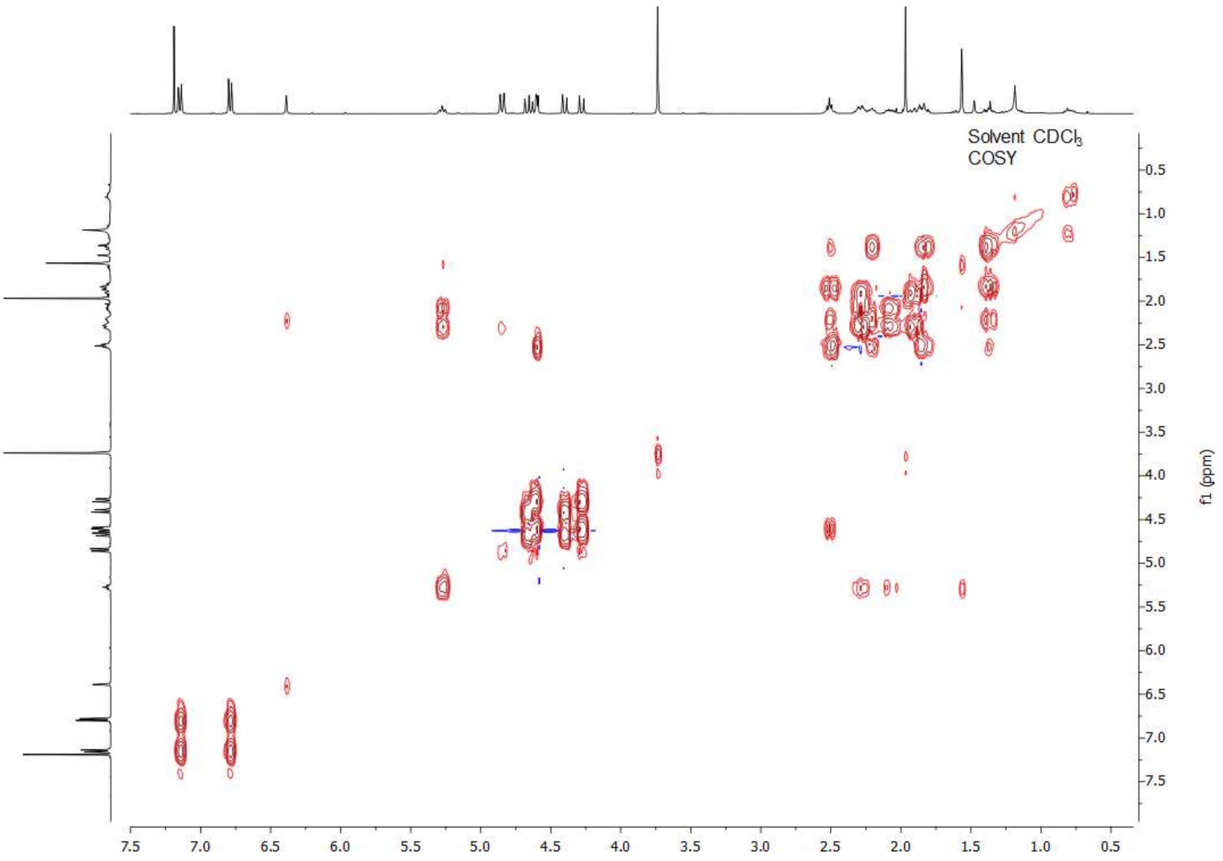

Solvent CDCl3
COSY
f1 (ppm)

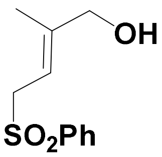

S32

Solvent CDCl3
MHz 400
Nucleus 1H

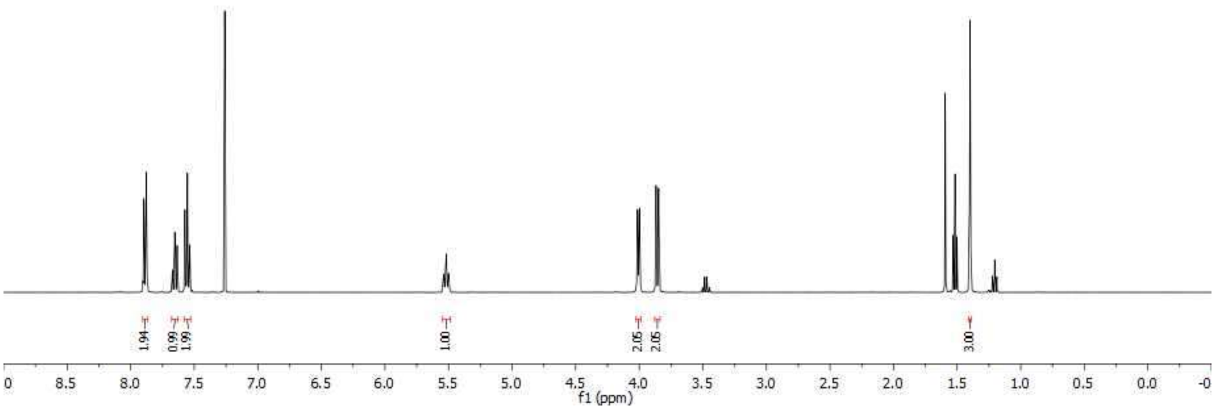

Solvent CDCl3
MHz 101
Nucleus 13C

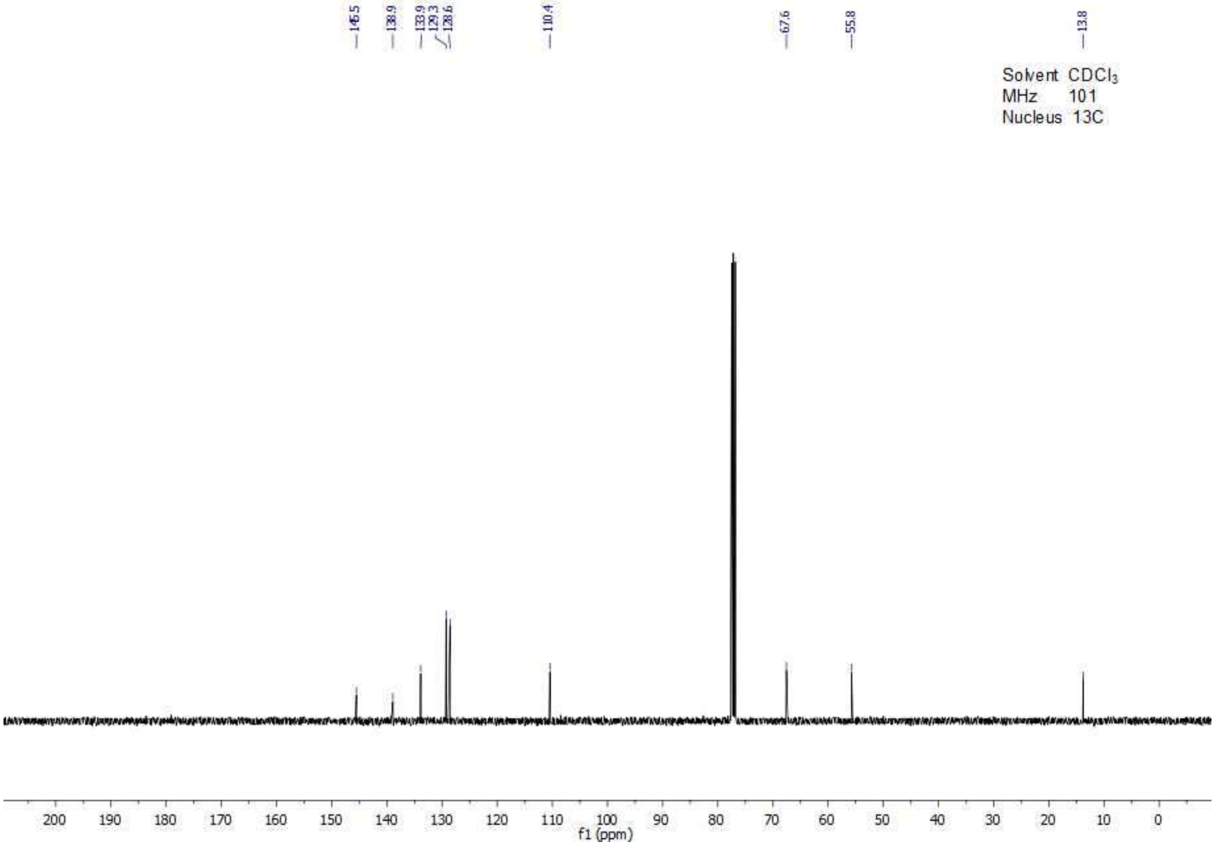

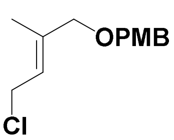

S33

Solvent CDCl$_3$
MHz 400
Nucleus 1H

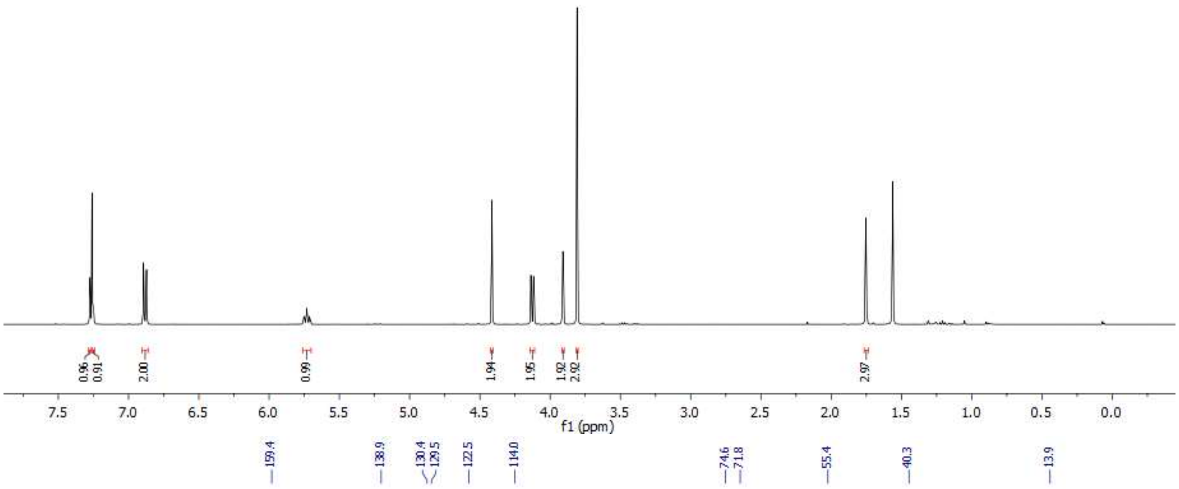

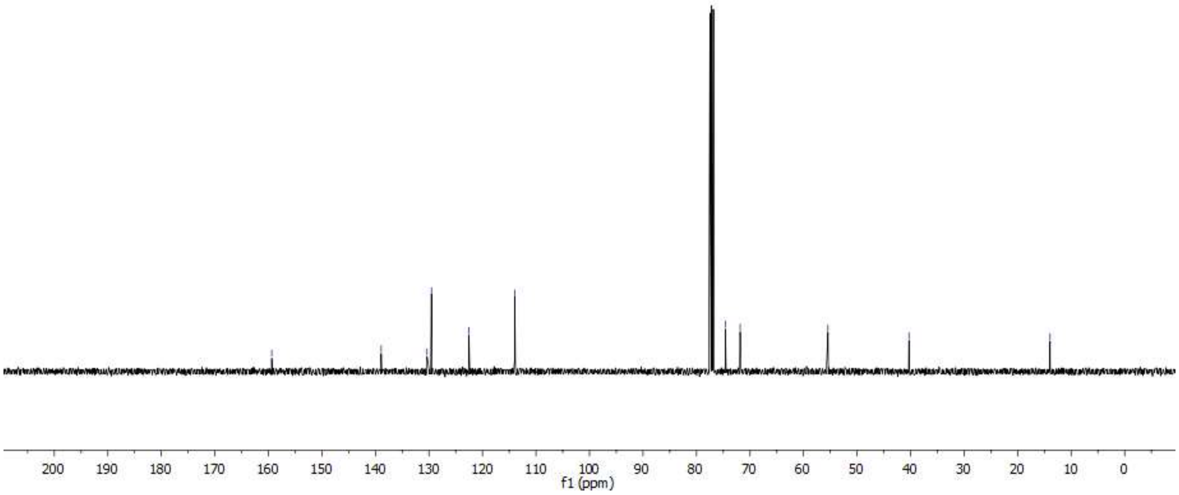

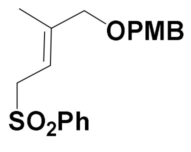

S32

Solvent $CDCl_3$
MHz 400
Nucleus 1H

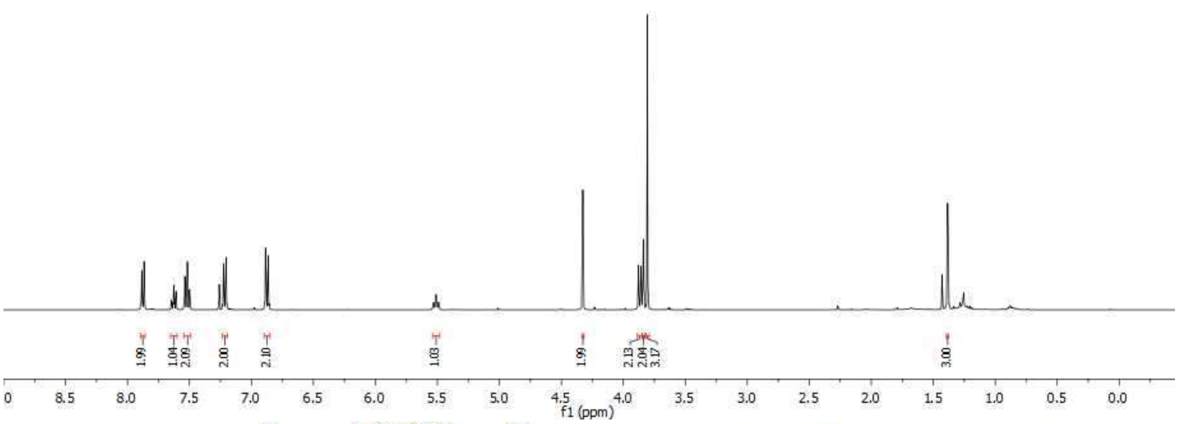

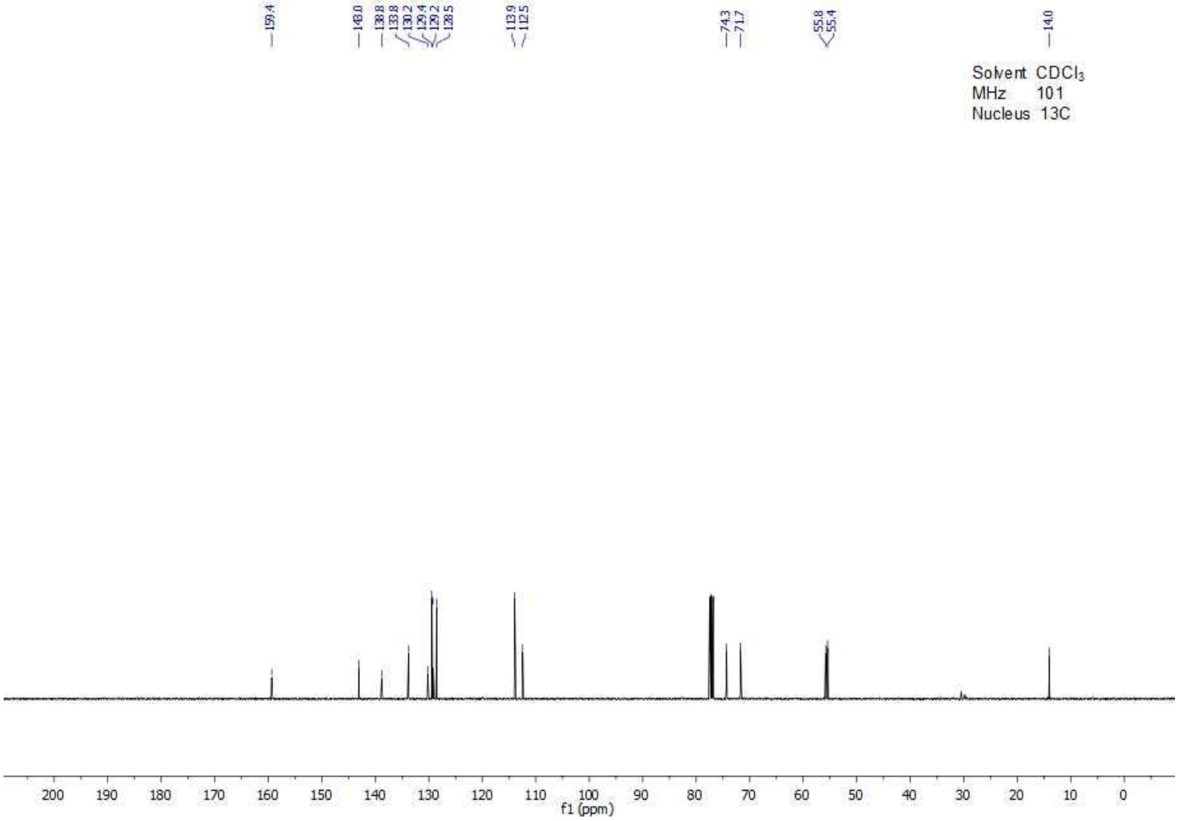

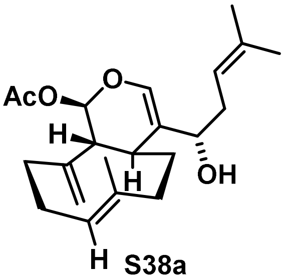

S38a

Solvent CDCl$_3$
MHz 400
Nucleus 1H

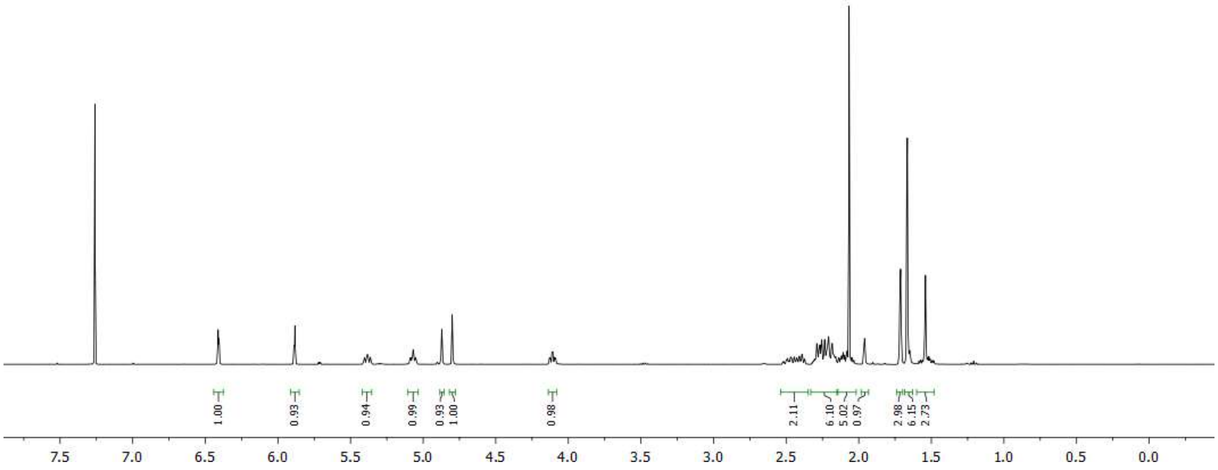

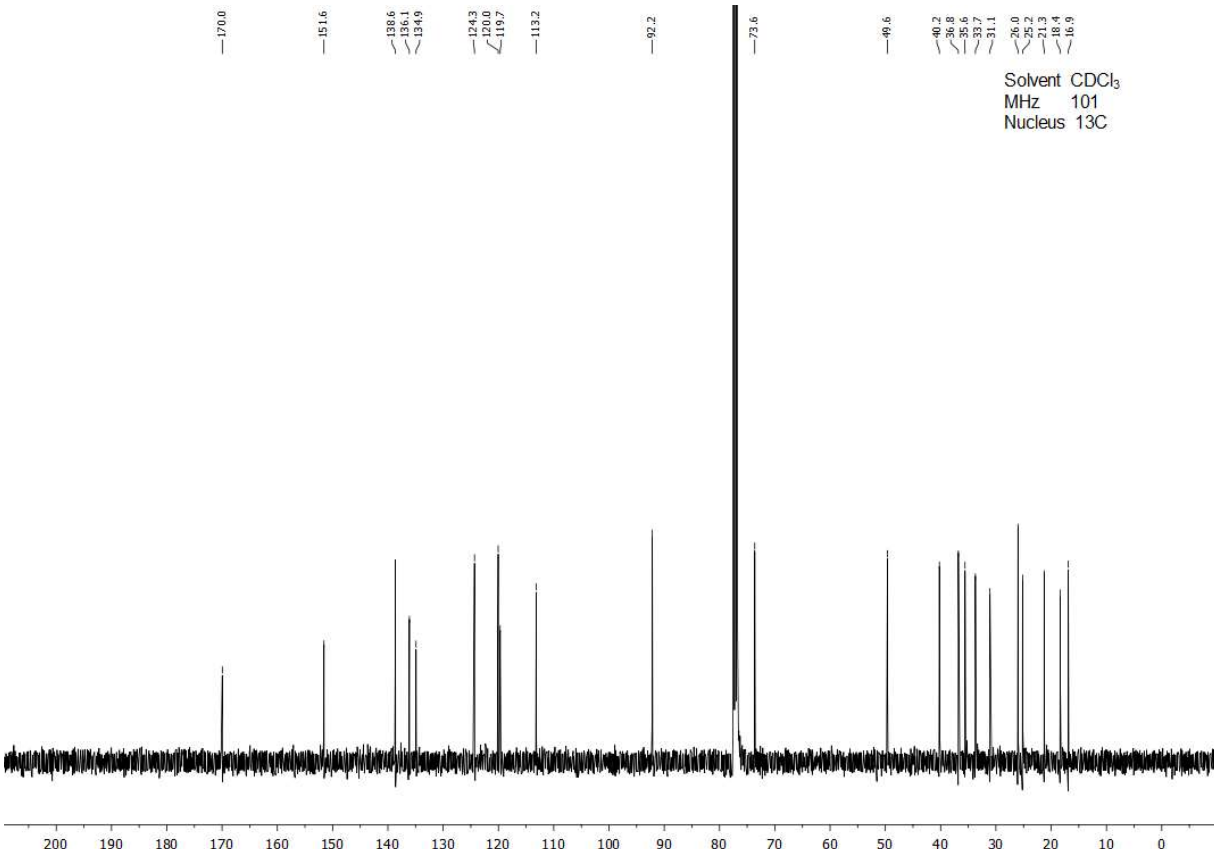

Solvent CDCl$_3$
MHz 101
Nucleus 13C

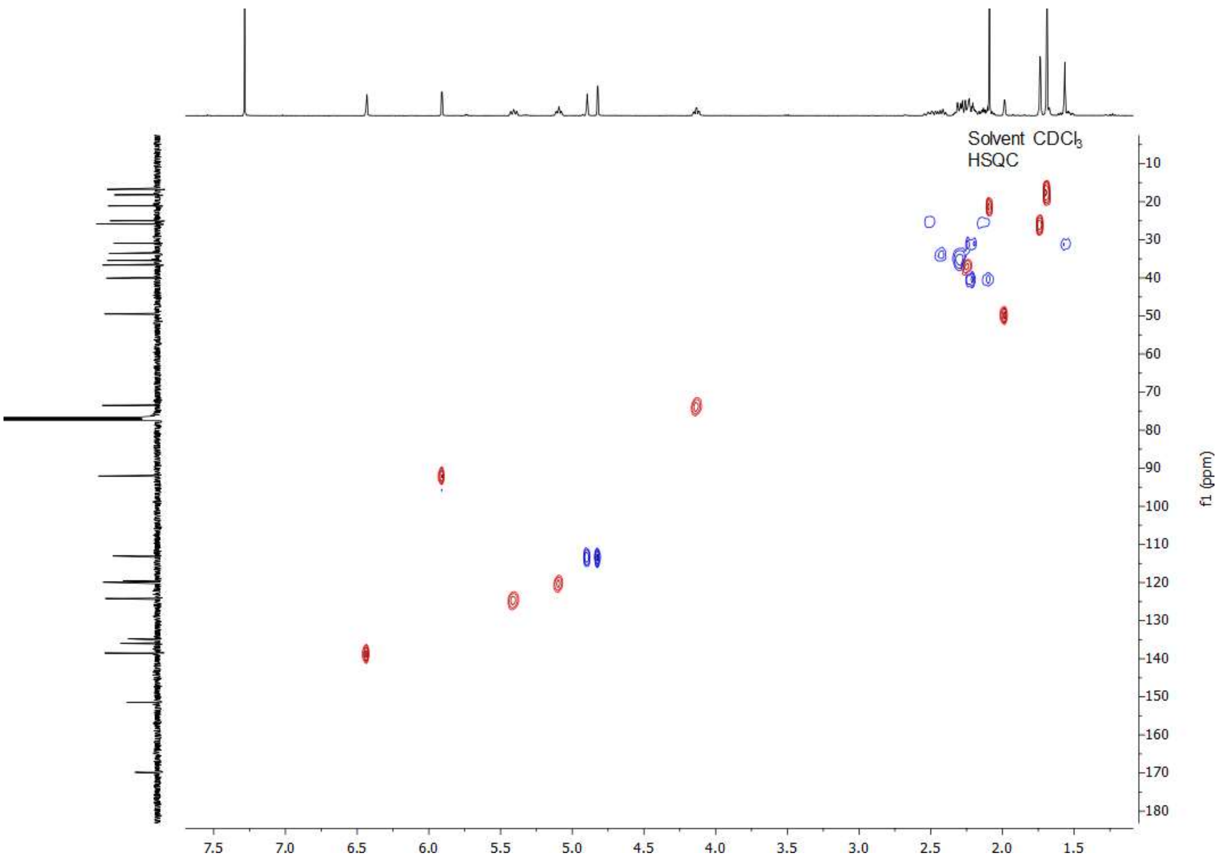
Solvent CDCl3
HSQC
f1 (ppm)

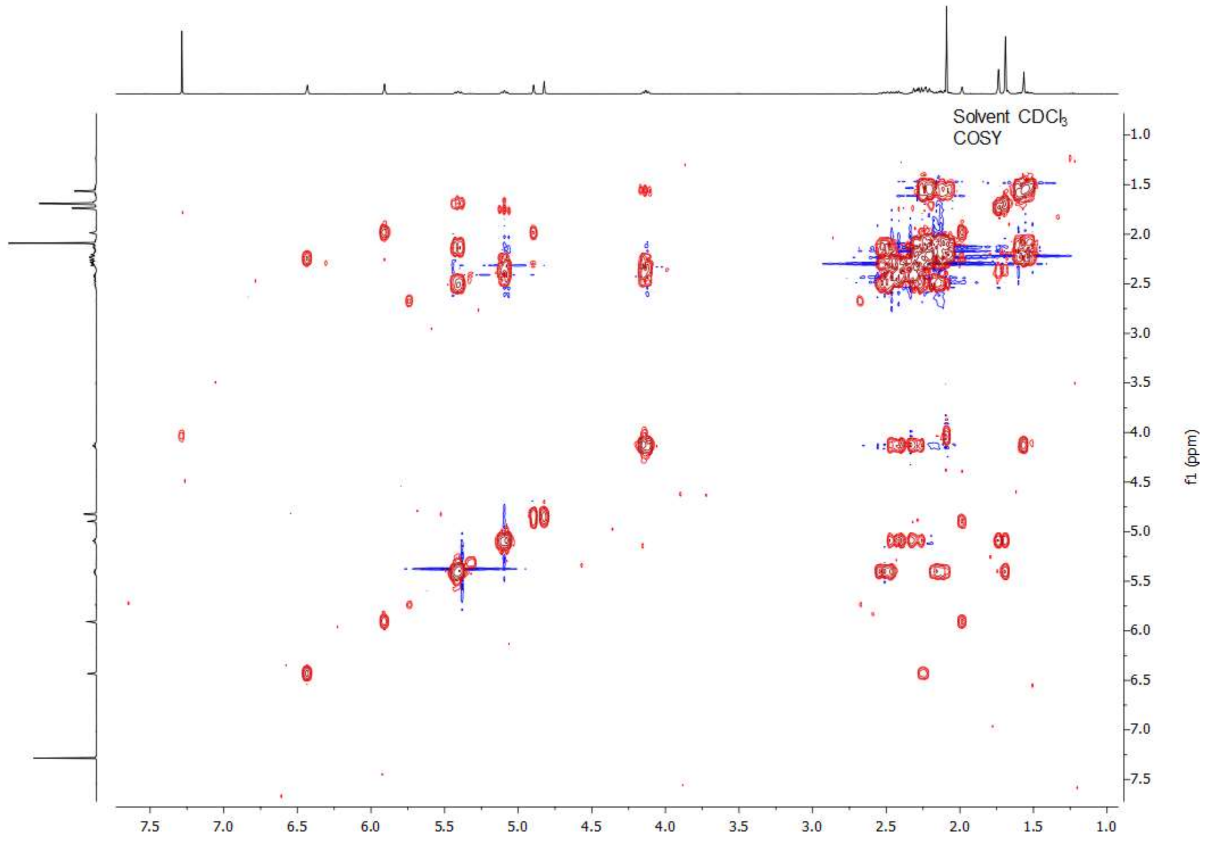
Solvent CDCl3
COSY
f1 (ppm)

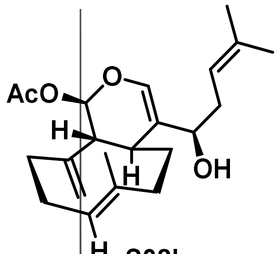
AcO
O
H
H
OH
H

S38b

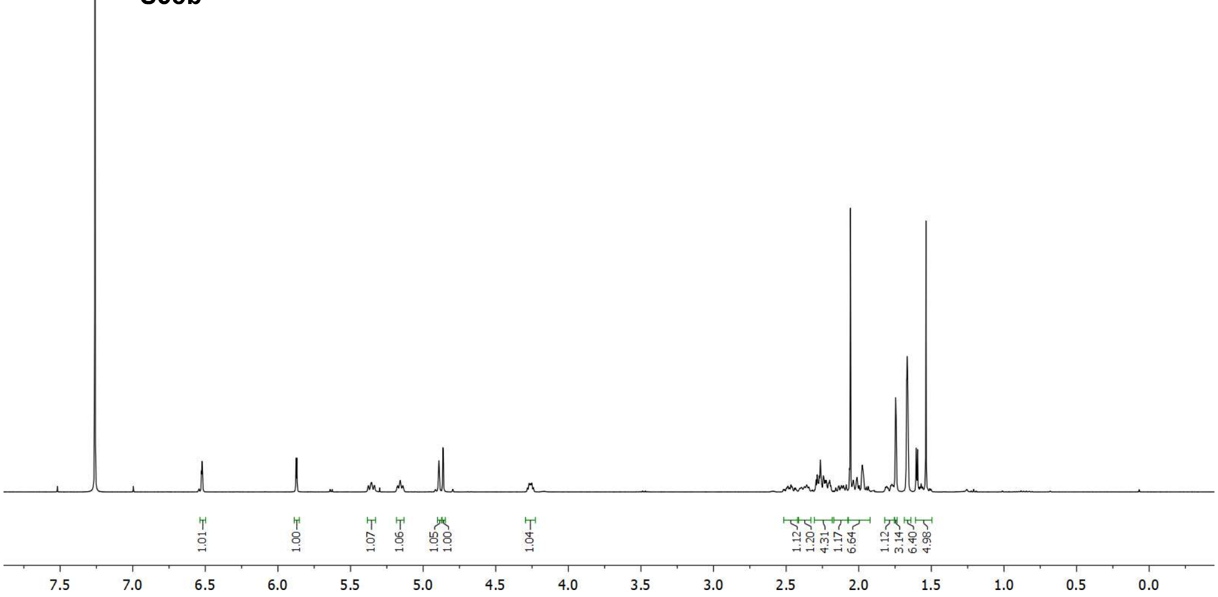
Solvent CDCl3
MHz 400
Nucleus 1H
1.01
1.00
1.07
1.06
1.05
1.00
1.04
1.12
1.20
4.31
1.17
6.64
1.12
3.14
6.40
4.98
7.5 7.0 6.5 6.0 5.5 5.0 4.5 4.0 3.5 3.0 2.5 2.0 1.5 1.0 0.5 0.0

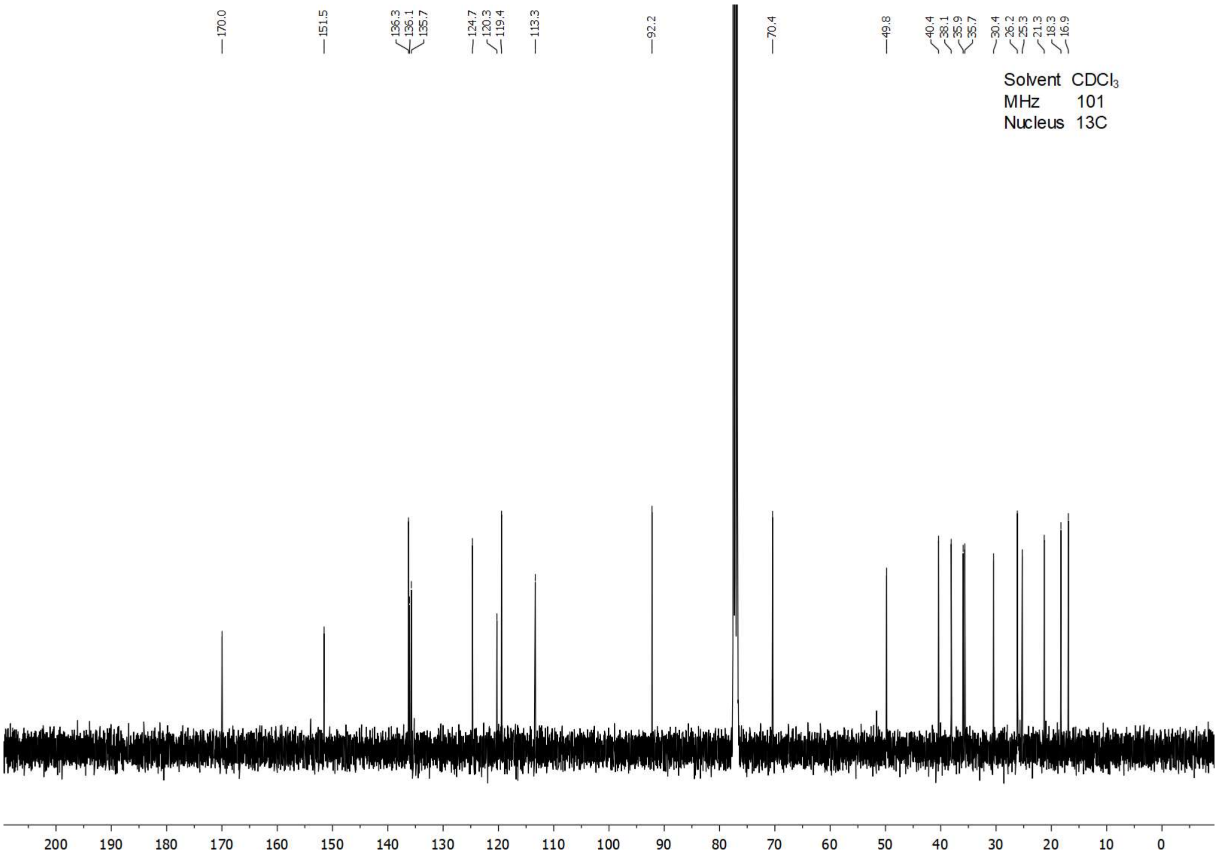
170.0
151.5
136.3
136.1
135.7
124.7
120.3
119.4
113.3
92.2
70.4
49.8
40.4
38.1
35.9
35.7
30.4
26.2
25.3
21.3
18.3
16.9
Solvent CDCl3
MHz 101
Nucleus 13C
200 190 180 170 160 150 140 130 120 110 100 90 80 70 60 50 40 30 20 10 0

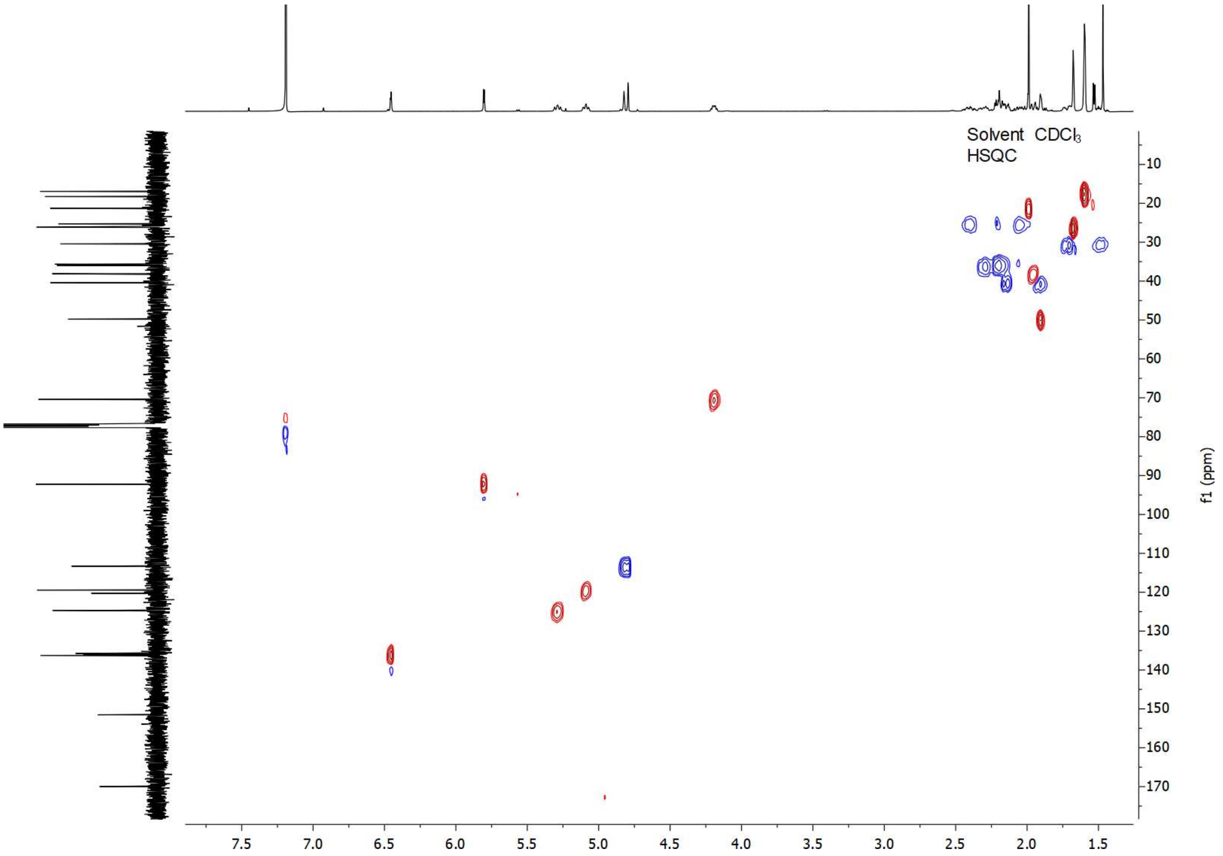

Solvent CDCl3
HSQC
f1 (ppm)

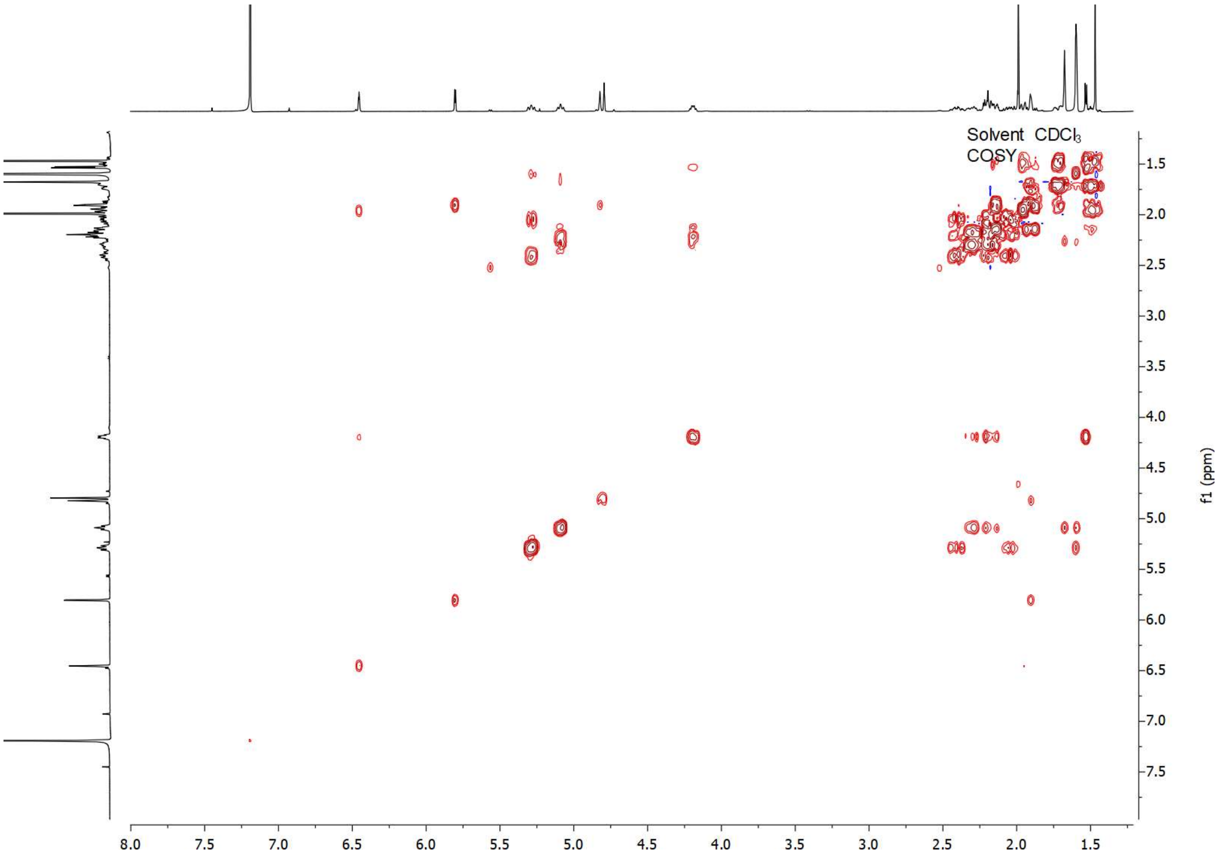

Solvent CDCl3
COSY
f1 (ppm)

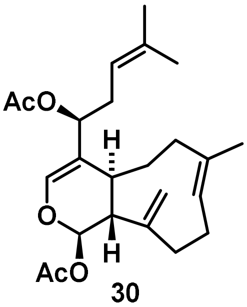

30

Solvent $CDCl_3$
MHz 400
Nucleus 1H

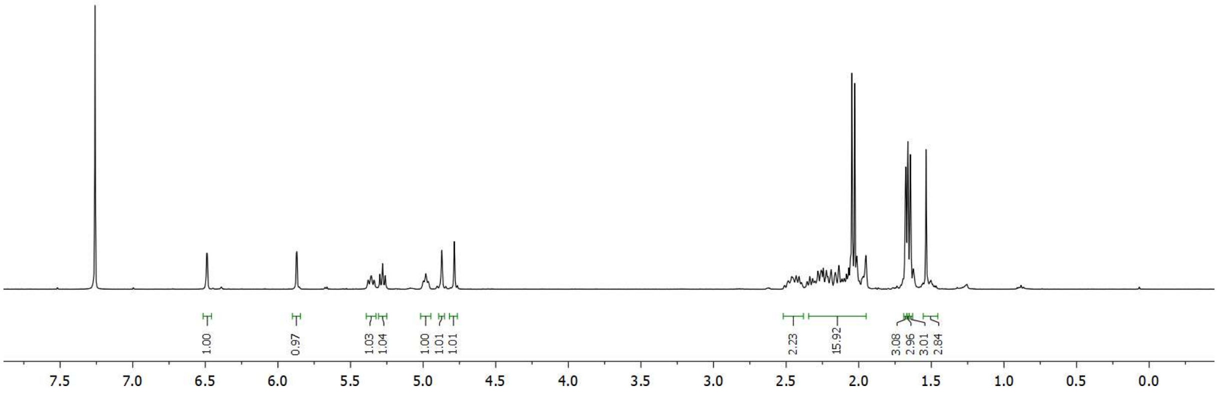

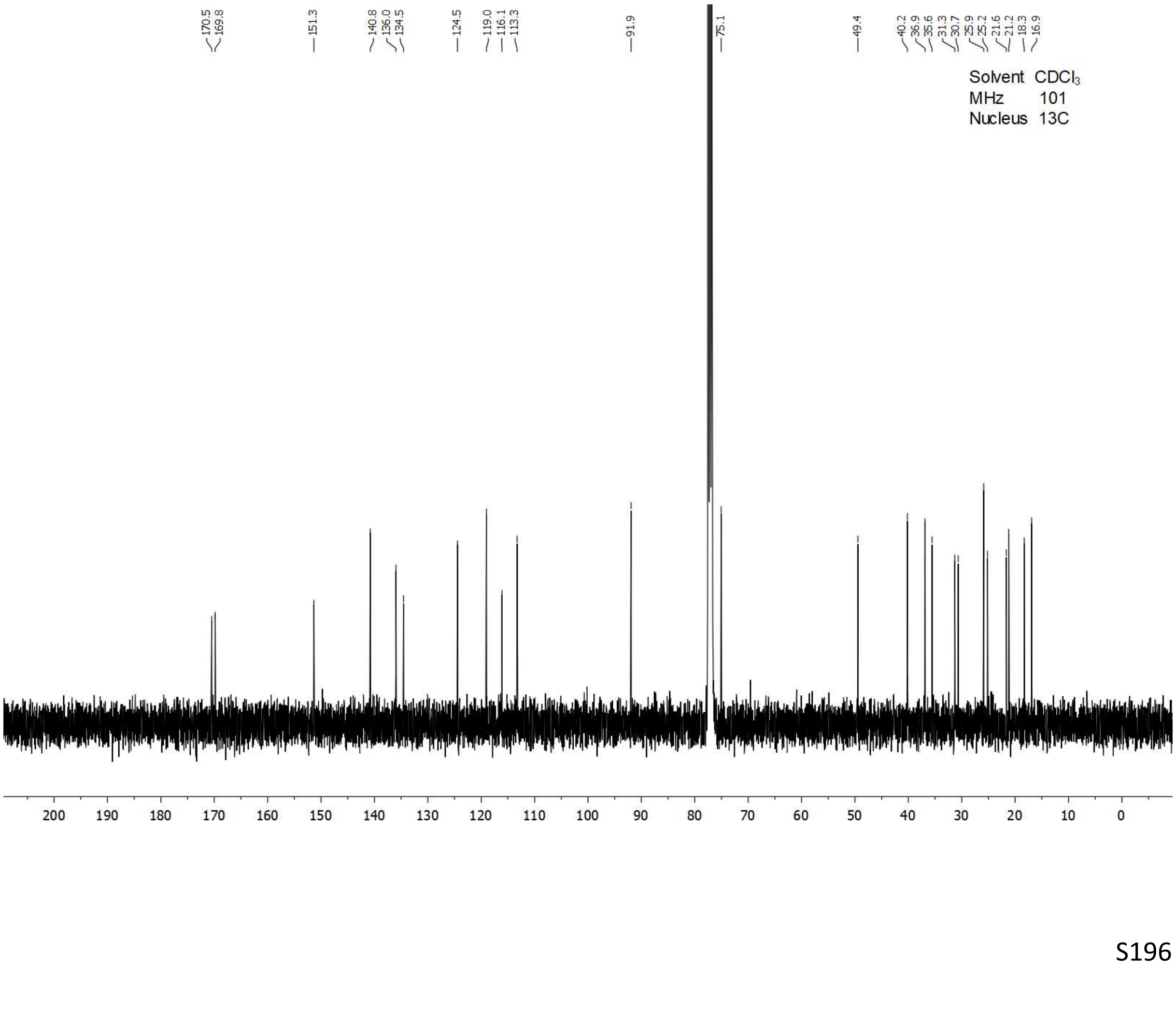

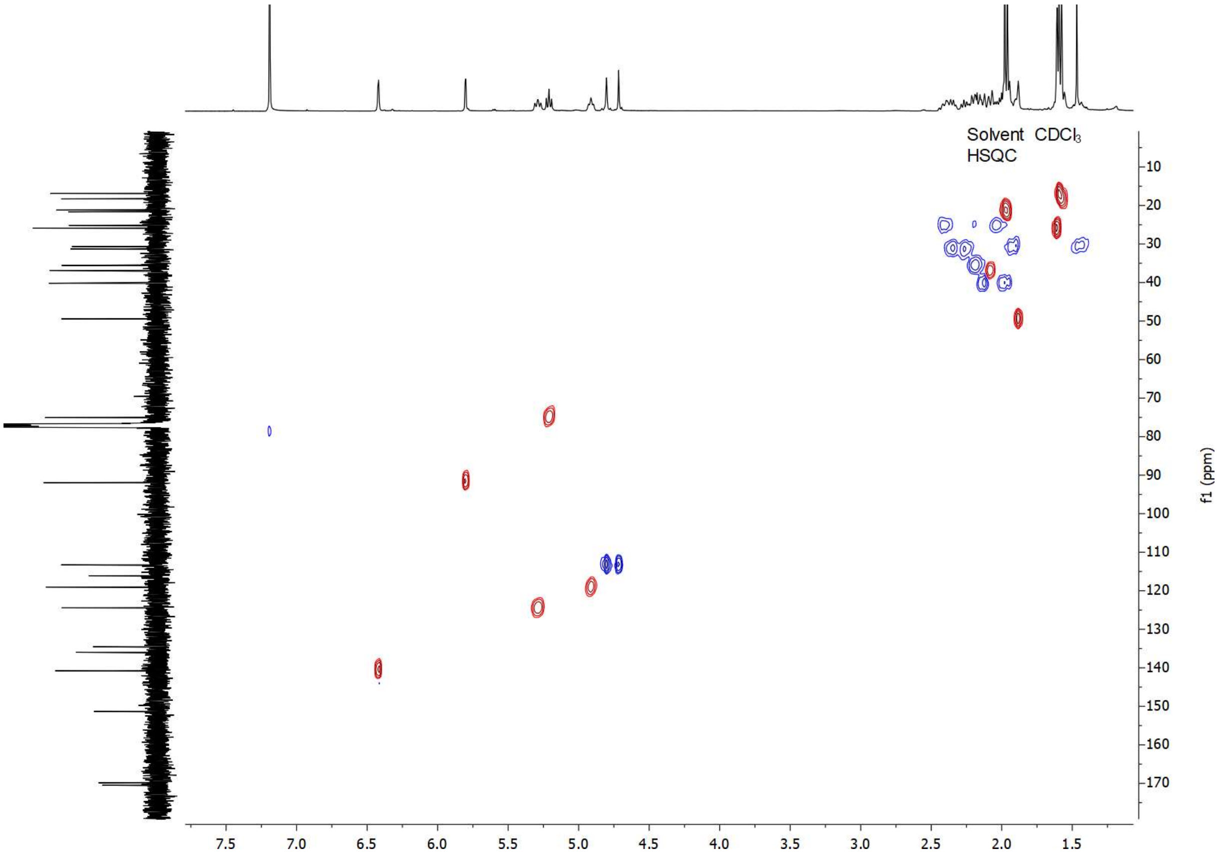
Solvent CDCl3
HSQC
f1 (ppm)

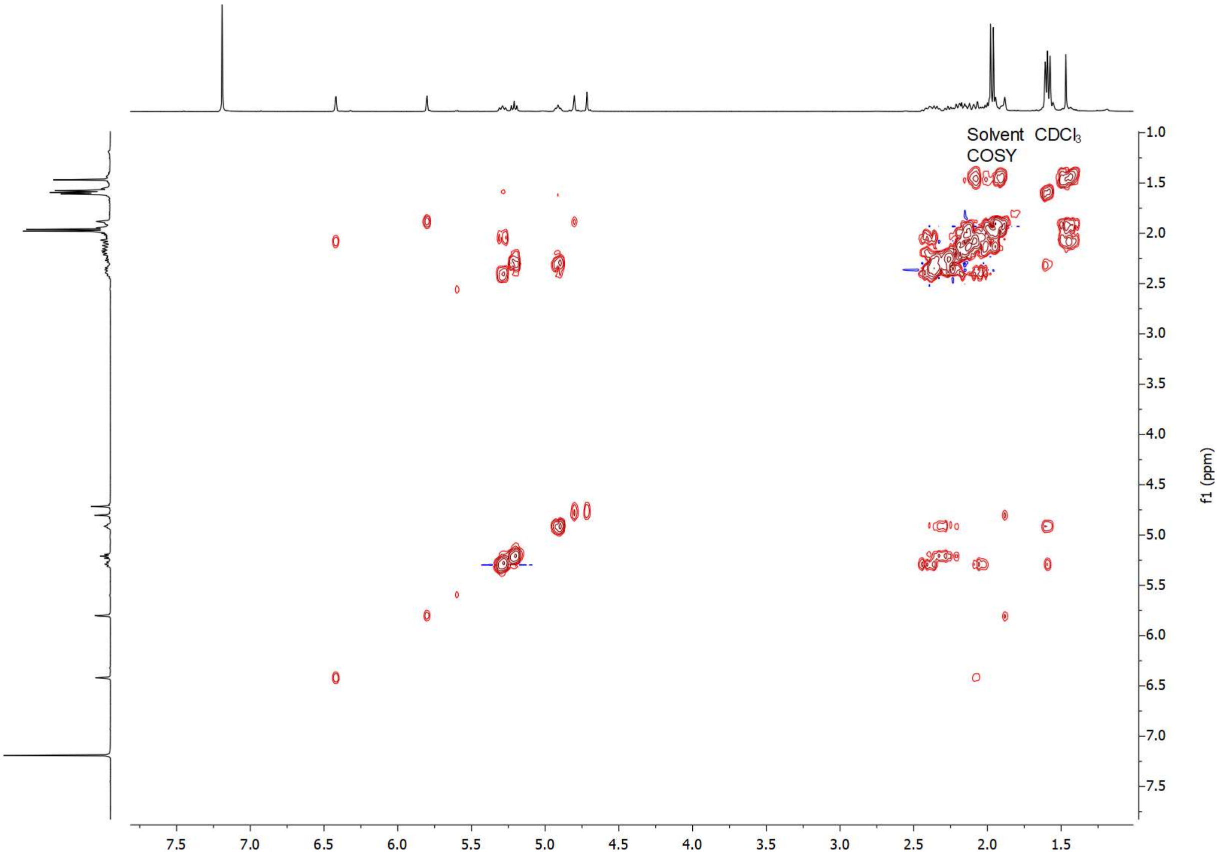
Solvent CDCl3
COSY
f1 (ppm)

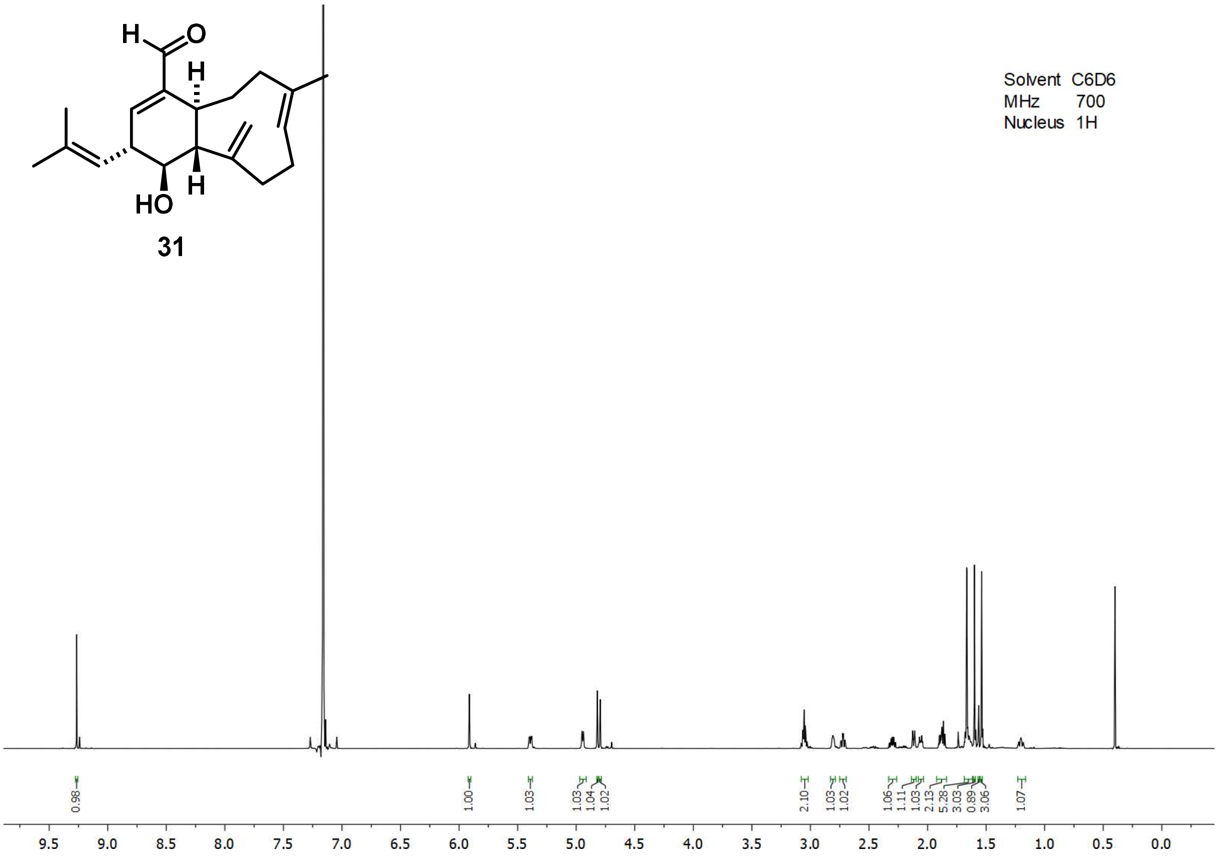
H
O
H
H
HO
31
Solvent C6D6
MHz 700
Nucleus 1H
0.98
1.00
1.03
1.03
1.04
1.02
2.10
1.03
1.02
1.06
1.11
1.03
2.13
5.28
3.03
0.89
3.06
1.07
9.5 9.0 8.5 8.0 7.5 7.0 6.5 6.0 5.5 5.0 4.5 4.0 3.5 3.0 2.5 2.0 1.5 1.0 0.5 0.0

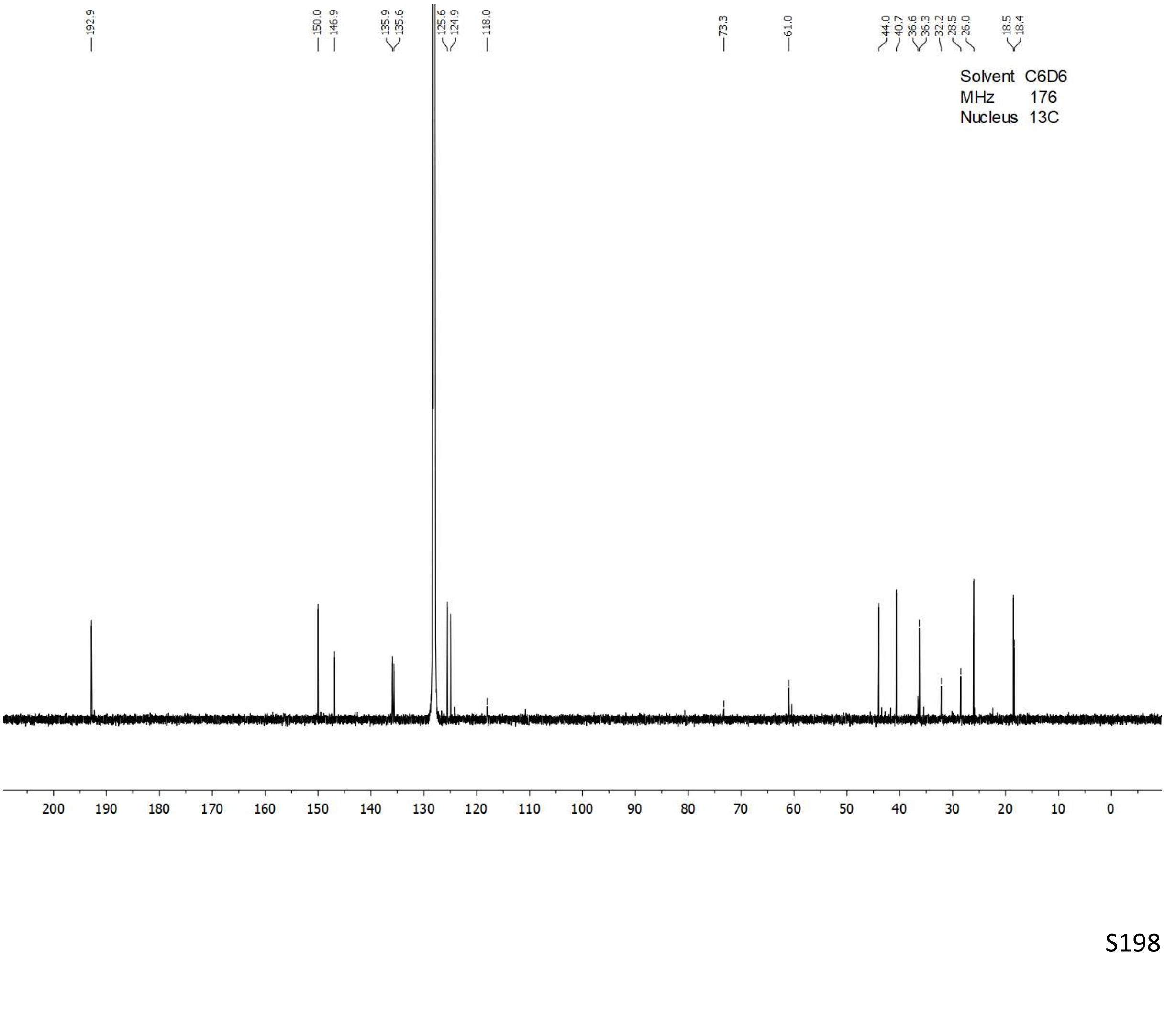
192.9
150.0
146.9
135.9
135.6
125.6
124.9
118.0
73.3
61.0
44.0
40.7
36.6
36.3
32.2
28.5
26.0
18.5
18.4
Solvent C6D6
MHz 176
Nucleus 13C
200 190 180 170 160 150 140 130 120 110 100 90 80 70 60 50 40 30 20 10 0

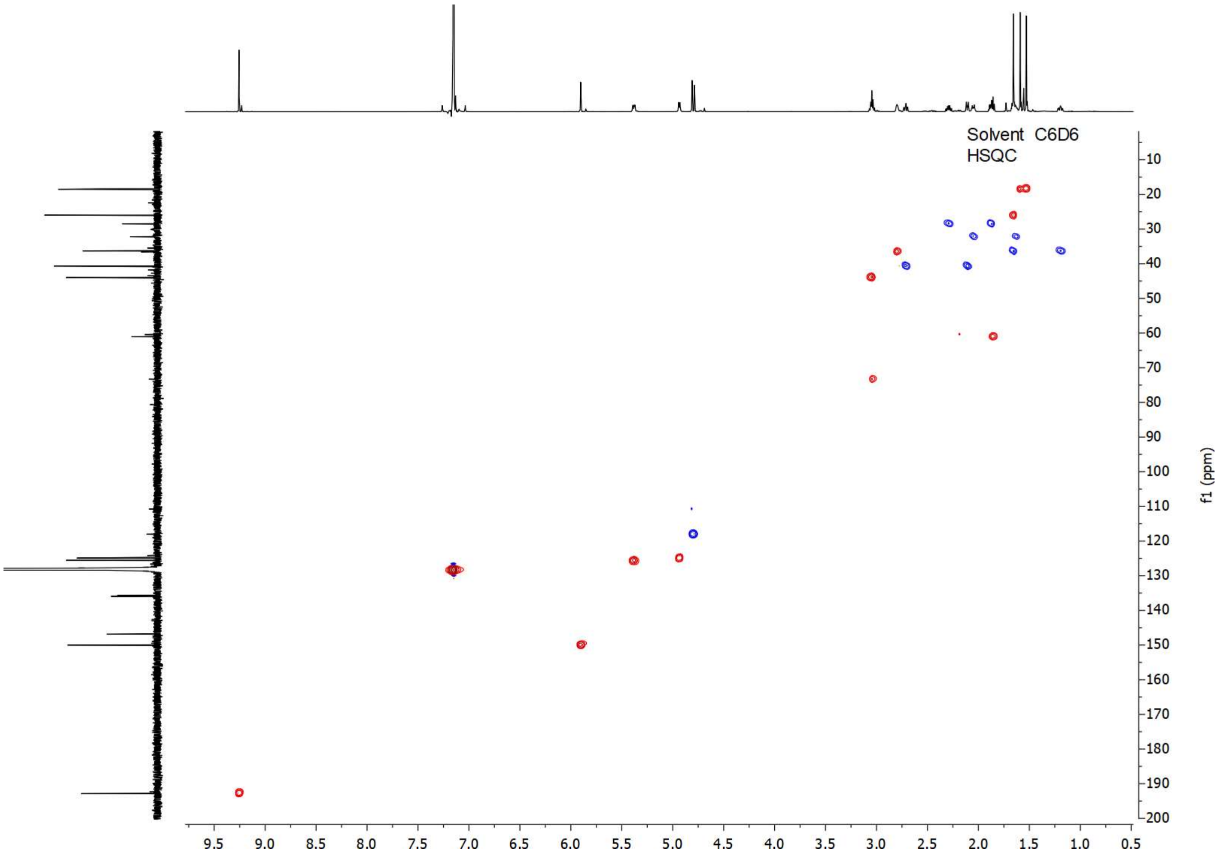
Solvent C6D6
HSQC
f1 (ppm)

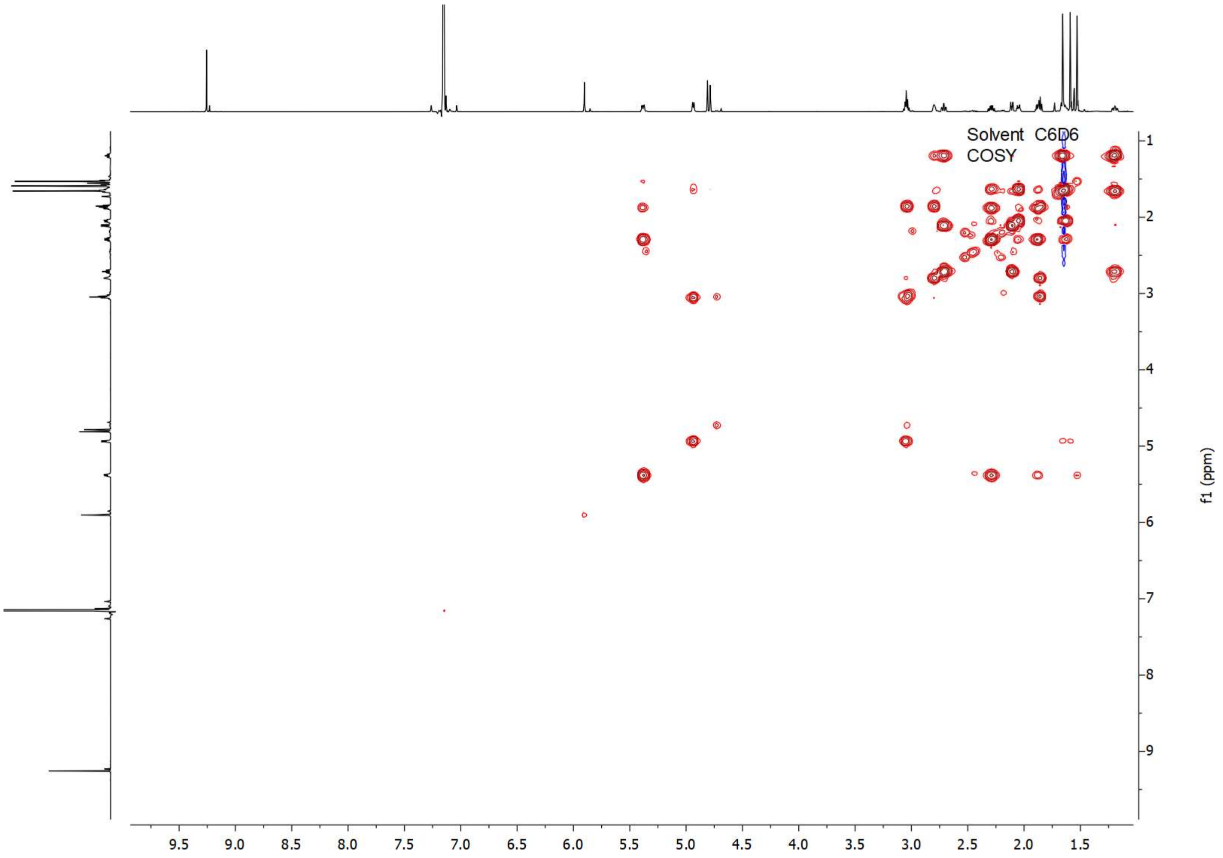
Solvent C6D6
COSY
f1 (ppm)

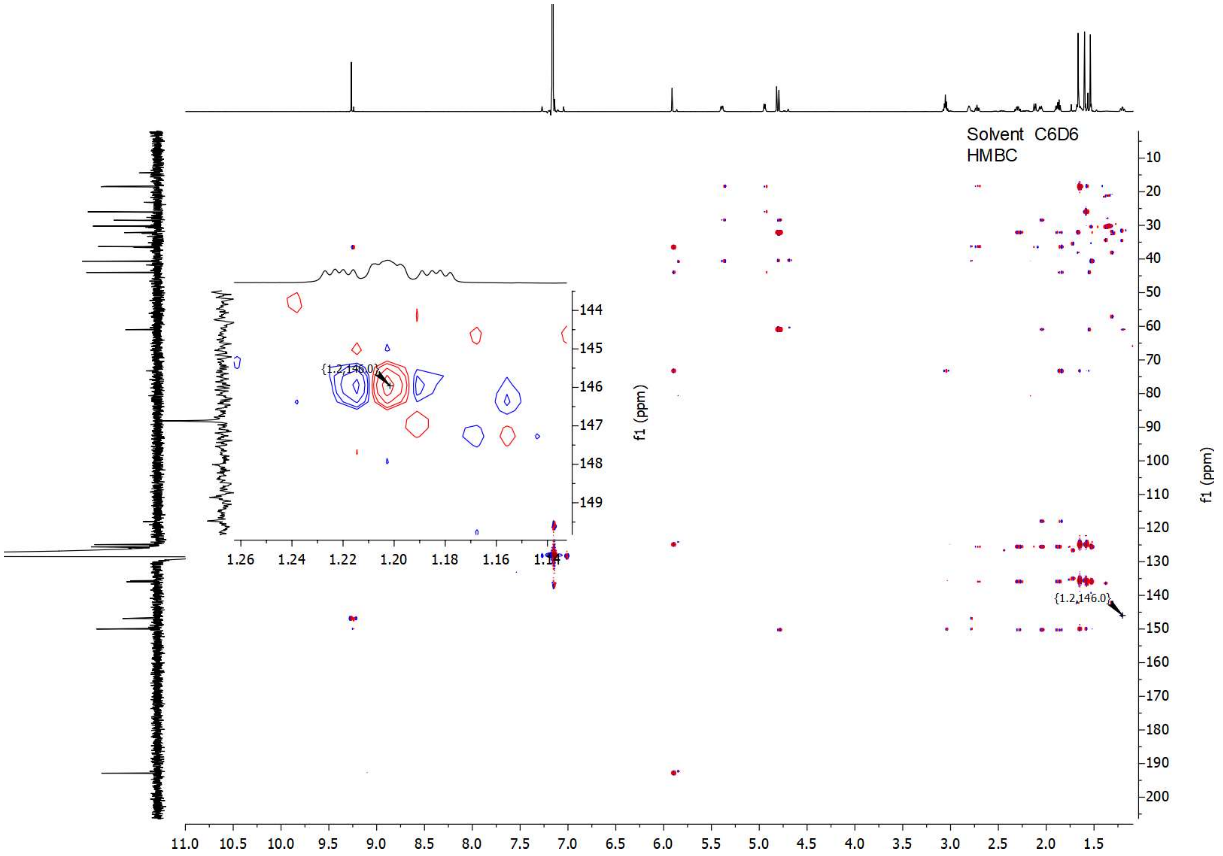
Solvent C6D6
HMBC
f1 (ppm)
{1.2,146.0}
